# Author Correction: A scope of prebiotic neat reaction conditions and the mechanism of urea-assisted phosphorylations of alcohols

**DOI:** 10.1038/s41467-026-69220-7

**Published:** 2026-02-03

**Authors:** Anastasiia Shvetsova, Lynda Merzoud, Augustin Lopez, Elodie Fromentin, Anne Baudouin, Henry Chermette, Isabelle Daniel, Michele Fiore, Peter Strazewski

**Affiliations:** 1 Institut de Chimie et Biochimie Moléculaires et Supramoléculaires, UMR5246, CNRS, Université Claude Bernard Lyon 1, Villeurbanne, France; 2 Laboratoire de Géologie de Lyon–Terre, Planètes et Environnement, UMR5276, CNRS, Université Claude Bernard Lyon 1, Villeurbanne, France; 3 Institut de Chimie Analytique, UMR5280, CNRS, Université Claude Bernard Lyon 1, Villeurbanne, France

**Keywords:** Origin of life, Geochemistry

Correction to: *Nature Communications* 10.1038/s41467-025-63307-3, published online 8 October 2025

In the version of this article initially published, there were errors in Fig. 4a, Int-1, where in the lower right-hand structure, the oxygen atom originally appeared as “OH”; in Fig. 4b, following the second reaction arrow, where H_3_ in “NH_3_” was mistakenly color-tagged in bold blue, and in Fig. 4b, Nucleophile trap, where CONH_2_ originally was written “CONH_3_.” The figure is now amended in the HTML and PDF versions of the article.

