# Supplementary material for: A scope of prebiotic neat reaction conditions and the mechanism of urea-assisted phosphorylations of alcohols
Source: Nat Commun. 2025 Oct 8;16:8929. doi: 10.1038/s41467-025-63307-3 (PMC12508118; doi:10.1038/s41467-025-63307-3)
Supplement: Supplementary file 1 — Supplementary Information [file 41467_2025_63307_MOESM1_ESM.pdf]

Supplementary Information

A scope of prebiotic neat reaction conditions and the mechanism of urea-assisted phosphorylations of alcohols

Anastasiia Shvetsova,[1,2] Lynda Merzoud,[3] Augustin Lopez,[1] Elodie Fromentin,[1] Anne Baudouin,[1] Henry Chermette,[3] Isabelle Daniel,[2] Michele Fiore[1] & Peter Strazewski[1]✉

[1] Institut de Chimie et Biochimie Moléculaires et Supramoléculaires, UMR5246 (CNRS), Université Claude Bernard Lyon 1, bât. Edgar Lederer, 1 rue Victor Grignard, F-69622 Villeurbanne, France.
[2] Laboratoire de Géologie de Lyon–Terre, Planètes et Environnement, UMR5276 (CNRS), Université Claude Bernard Lyon 1, France.
[3] Institut de Chimie Analytique, UMR5280 (CNRS), Université Claude Bernard Lyon 1, France.

✉email : strazewski@univ-lyon1.fr

This PDF file (231 pages) includes:

☛ **Table of contents** for Sections 1–9 (with internal links to the sub-sections)

☛ **Materials and Methods** (Section 1)

☛ **Design of experiments** (Section 2)

☛ **Study of the reaction mechanism by quantum theoretical methods** (Section 3)

☛ **Identification of the reaction products by NMR spectroscopic and mass spectrometric methods** (Section 4)

☛ **Kinetic study of urea-assisted phosphorylation reactions** (Section 5)

☛ **Tracing stable isotopes in volatiles evaporating from ‘dry’ phosphorylation reactions** (Section 6)

☛ **A scope of phosphorylation reactions in prebiotic conditions** (Section 7)

☛ **Vesiculation of crude phosphorylation mixtures** (Section 8)

☛ **Supplementary References** 1–47 (Section 9)

All sections contain Supplementary Notes and Discussions, and some contain Supplementary Conclusions:

☛ **Conclusion on the mechanism** (3.4)

☛ **Supplementary conclusion on the urea-assisted phosphorylation of glycerol (5) and MPG (6)** (5.4)

☛ **Conclusion on tracing stable isotopes in volatiles and non-volatiles formed during the urea-assisted neat phosphorylation reactions** (6.3)

☛ **Supplementary conclusion on nucleoside phosphorylation** (7.5.8)

☛ **Conclusion on the reactivity of pyruvic acid (16)** (7.11.1)

Supplementary Figures and Supplementary Tables:

☛ **Supplementary Fig. 1** to ☛ **Supplementary Fig. 223**

☛ **Supplementary Table 1** to ☛ **Supplementary Table 67**

Table of contents

1. Materials and Methods 5

1.1. Chemicals, solvents and general equipment 5

1.2. NMR equipment, signal description, and quantification methods 9

1.2.1. Sample preparation for NMR spectroscopic analysis 10

1.3. MS and LC-MS material, equipment and methods 10

1.3.1. Sample preparation for MS analysis (LC-MS, LRMS, HRMS) of the crude reaction mixtures, trap 1 and trap 2 contents 11

1.4. HPLC equipment, material and method for the analysis of MPG phosphorylation 11

1.5. Quantum theoretical methods 11

1.6. Synthesis methods 12

1.6.1. Synthesis of racemic mono-palmitoylglycerol (MPG) **6** 12

1.6.2. NMR and MS characterisation of MPG (**6**) 13

1.6.3. Synthesis and characterisation of racemic *bis*-tridecanoyl and dioleoyl glycerol 14

1.6.3.1. Synthesis of TBDMS-*rac*-BTG (**20**) 14

1.6.3.2. Characterisation of TBDMS-*rac* BTG (**20**) 14

1.6.3.3. Synthesis of *rac*-BTG (**7**) 15

1.6.3.4. Characterisation of *rac*-BTG (**7**) 16

1.6.4. Synthesis of $NaH_2P[^{18}O_4]$ (**$[^{18}O_4]P_i$**) 18

1.6.4.1. $H_3P[^{18}O_4]$ 18

1.6.4.2. $NaH_2P[^{18}O_4]$ 19

2. Design of experiments 21

2.1. Carousel™ reactions 21

2.2. Experimental set-up for tracing stable isotopes in urea-assisted phosphorylation reactions of glycerol and racemic monopalmitoylglycerol 21

3. Study of the reaction mechanism by quantum theoretical methods 23

3.1. State of the art and objectives concerning the reaction mechanism of the urea-assisted phosphorylation of alcohols 23

3.2. Associative (organo-catalytic) versus dissociative (eliminative) phosphate activation 24

3.3. Thermodynamic preferences and kinetic competition 25

3.4. Conclusion on the mechanism 26

4. Identification of the reaction products by NMR spectroscopic and mass spectrometric methods 29

4.1. Urea-assisted glycerol (**5**) phosphorylation 31

4.1.1. Two persistent organic phosphorylated and carbonylated minor reaction products **36**

4.1.1.1. Proton-decoupled heteronuclear multiple-bond correlation and J-modulated spin-echo ^{13}C nuclear magnetic resonance spectroscopies 39
4.1.2. High-resolution mass spectrometry and hydrophilic-ion liquid chromatography 42
4.2. Urea-assisted phosphorylation of MPG (**6**) 45
4.2.1. ^{1}H and ^{31}P NMR and ^{1}H-^{31}P HMBC spectroscopies 45
4.2.2. Reversed-phase high-performance liquid chromatography coupled to high-resolution mass spectrometry (RP-HPLC-HRMS) 48
4.3. Inorganic cyanate (NCO^-) and carbamoyl phosphate (**CP_i**) 49
5. Kinetic study of urea-assisted phosphorylation reactions 52
5.1. Phosphorylation of glycerol (**5**) 52
5.1.1. Thermal stability of urea in solution 52
5.1.2. Glycerol phosphorylation mixtures containing [$^{15}N_2$]urea 57
5.2. Phosphorylation of racemic monopalmitoylglycerol (**6**) 67
5.3. Loss of residual water in 'dry' conditions 76
5.4. Supplementary conclusion on the urea-assisted phosphorylation of glycerol (**5**) and MPG (**6**) 76
6. Tracing stable isotopes in volatiles evaporating from 'dry' phosphorylation reactions 78
6.1. Trap 1: Gaseous nucleophiles (ammonia, water) trapped as benzamide and benzoic acid 78
6.2. Trap 2: CO_2 trapped as benzoic acid (benzamide from trapping HNCO not found by LC-MS) 97
6.3. Conclusion on tracing stable isotopes in volatiles and non-volatiles formed during the urea-assisted neat phosphorylation reactions 109
7. A scope of phosphorylation reactions in prebiotic conditions 111
7.1. Dependence on condensing agent of phosphorylation of glycerol (**5**) 111
7.2. Dependence on condensing agent (catalyst, liquidiser) of phosphorylation of MPG (**6**) 113
7.3. Phosphorylation of MPG (**6**) assisted by urea and cyanamide studied by HPLC. 116
7.4. Dependence of phosphorylation of glycerol and MPG on phosphorous source 118
7.4.1. Effect of protonation degree of sodium orthophosphate on phosphorylation of glycerol (**5**) 118
7.4.2. Experiments with cyclic trimetaphosphate (**cTMP**) 119
7.4.3. Experiments with sodium thiophosphate (**SP_i**) 124
7.4.3.1. Competition between **SP_i** and **[$^{18}O_4$]P_i** in urea-assisted phosphorylations of glycerol and MPG 131
7.4.4. Involving natural minerals and their analogues in phosphorylation of glycerol and MPG 136
7.5. Phosphorylation of nucleosides 138
7.5.1. Phosphorylation of adenosine in the presence of water or formamide (**3a**) 138
7.5.2. Experiments with different condensing agents (cyanamide **1** and urea **2a**) 140

7.5.3. Temperature dependence of urea- and cyanamide-assisted adenosine phosphorylation **151**

7.5.4. Effect of urea excess on phosphorylation of nucleosides **152**

7.5.5. Experiments with **cTMP** as a phosphorous source **160**

7.5.6. Urea-assisted phosphorylation of equimolar ‘dry’ mixtures of mononucleotides without and with complementary nucleosides, and of all ribonucleosides **161**

7.5.6.1. HPLC-HRMS analysis of urea-assisted phosphorylation of a dry equimolar mixture of all ribonucleosides **167**

7.5.7. Urea-assisted phosphorylation of a mixture of ribonucleosides in the presence of valine **173**

7.5.8. Supplementary conclusion on nucleoside phosphorylation **174**

7.6. One-pot experiment with glycerol, the ribonucleosides, C_{10}:C_{11}:C_{12}:C_{13} alkanoic acids and urea as a condensing agent **174**

7.6.1. NMR analyses of products **175**

7.6.2. MS analysis of products **180**

7.7 Phosphorylation of 2,3-*bis*-(tridecanoyl)glycerol: BTG (**7**) **187**

7.8 Phosphorylation of 2,3-dioleoylglycerol: DOG (**8**) **191**

7.9 Phosphorylation of dodecan-1-ol (**9**) **194**

7.9.1 NMR and HRMS analyses of urea- and cyanamide-assisted reaction mixtures containing dodecan-1-ol (**9**), its phosphate esters and other amphiphilic products **195**

7.9.2 Other phosphate sources and reaction conditions for dodecan-1-ol (**9**) **202**

7.10 Phosphorylation of geraniol (**10**) **212**

7.11 Phosphorylation of pyruvic acid (**16**) **217**

7.11.1 Conclusion on the reactivity of pyruvic acid (**16**) **225**

8. Vesiculation of crude phosphorylation mixtures 228

9. Supplementary References 229

1. Materials and Methods

1.1. Chemicals, solvents and general equipment

➢ 1-Dodecanol (98 %), urea (99.5 %), formamide (99 %), benzoyl chloride (99 %), acetic acid (≥ 99 %), 4-dimethylaminopyridine (DMAP) (99 %), phenyl magnesium bromide (1.6 M PhMgBr in cyclopentyl methyl ether), tetrahydrofuran (99.5 %), toluene (99.85 %), cytidine (99.5 %), uridine (99.5 %), ammonium magnesium phosphate hexahydrate (98 %) and 4-(dimethylamino)pyridine (99 %) were purchased from *ACROS Organics* (*Thermo-Fisher Scientific,* Inc.). Geraniol (97 %), sodium cyclic trimetaphosphate (≥ 99 %) and adenosine (99 %) were purchased from *Alfa Caesar*. Palmitoyl chloride (98 %), guanosine (≥ 98 %), thymidine (99 %) and triethylamine *tris*-hydrofluoride (37 % HF) were purchased from *Fluka BioChemika* (*Honeywell International* Inc.). [^{13}C]Urea (98 %), [^{15}N]formamide (≥ 98 %) and [^{15}N]acetamide (≥ 98 %) were purchased from *Cambridge Isotope Laboratories,* Inc. (*CIL*). [^{15}N]$_2$Urea (≥ 98 %) was purchased from *CIL* and *Sigma-Aldrich*. Sodium dihydrogen phosphate (≥ 99 %), sodium thiophosphate tribasic hydrate (≥ 90 %), sodium phosphate dibasic (≥ 99 %), sodium phosphate tribasic dodecahydrate (≥ 98 %), N-methylacetamide (≥ 99 %), N-methylformamide (99 %), cyanamide (99 %), pyruvic acid (98 %), glycerol (≥ 99.5 %), acetamide (99 %), adenosine 5′-diphosphate sodium salt (≥ 95 %), L-alanine (≥ 99 %), L-valine (≥ 99 %), D-valine (≥ 98 %), cyclohexylammonium phosphoenolpyruvate (≥ 97 %) and dilithium carbamoyl phosphate hydrate (≥ 85 %) were purchased from *Sigma-Aldrich* (*Sigma Aldrich Chimie* S.a.r.l., *Merck* KGaA). Adenosine 5'-monophosphate disodium salt (≥ 98%), adenosine 5'-triphosphate disodium salt hydrate (≥ 97 %), guanosine 5'-monophosphate disodium salt (≥ 98 %), uridine 5'-monophosphate disodium salt (≥ 98 %) and cytidine 5'-monophosphate disodium salt (≥ 95 %) were purchased from *Carbosynth* (*Biosynth Carbosynth* Ltd). Palmitic acid (> 99.5 %), oleic acid (> 85.0 %), oleoyl chloride (> 80.0 %) and N-(3-dimethylaminopropyl)-N′-ethylcarbodiimide hydrochloride (> 98.0 %) were purchased from *Tokyo Chemical Industry* Co. (*Tokyo Kasei Kogyo*). Tridecanoic acid (95.0 %) and DL-1,2-isopropylideneglycerol (98 %) were purchased from *Fluorochem,* Ltd. Calcium pyrophosphate (> 96 %) was purchased from *Thermo-Fisher Scientific*. A crystalline mixture of 1- and 2-glyceryl phosphate (≈ 6:4 mol/mol) was produced by *Prolabo*, *Rhône-Poulenc*. A piece of natural vivianite was purchased from *Du Minéral au Bijou* <hosmalin-mineraux.fr>.

➢ Anhydrous toluene, dichloromethane (CH_2Cl_2 = DCM) and tetrahydrofuran (THF) were produced using a solvent drying apparatus (*Innovative Technology* Ltd, China) and the needed volumes of solvents were collected under a constant argon flow. All deuterated solvents were purchased from *Eurisotop* (Saint-Aubin Cedex, France). HPLC solvents were purchased from *Thermo-Fischer Scientific* (mass spectrometry grade). Thin-layer chromatography (TLC) was carried out on aluminium sheets coated with silica gel 60 F254 (*Merck*). TLC plates were inspected by UV light (λ = 254 nm) and developed by treatment with a mixture of 10 % H_2SO_4 in EtOH/H_2O (1:1 *v/v*) followed by heating.

➢ Melting points were measured in a capillary in heated oil on a *Büchi 510* apparatus.

Supplementary Table 1: Chemical substances used in this work. Reduced-nitrogen compounds **1-4c**, alcohols **5-16** (pyruvic acid **16** can react as enol tautomer **16a**), reference compounds: glyceryl phosphate (**5a**), alkanoic acids (**6a**, **7abcd**), 1-dodecyl phosphate (**9b**), nucleoside mono-, di- and triphosphates (**11abc**, **12a**, **13a**, **14a**), phosphoenol pyruvate (PEP, **16b**), educts (**17**-**21**), amino acids (**22abc**), inorganic phosphate sources, chemical reagents.

Compound number/code	Compound name (composition)	Structure	Molecular weight [g/mol]	Monoisotopic molecular mass* [Da]
**1**	Cyanamide	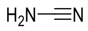	42.04	42.0218
**2a**	Urea	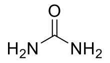	60.06	60.0324
**2b**	[$^{15}N_2$]Urea	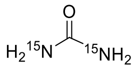	62.04	62.0264
**2c**	[^{13}C]Urea	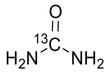	61.05	61.0357

**3a**	Formamide	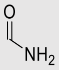	45.04	45.0215
**3b**	$[^{15}N]$Formamide	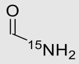	46.03	46.0185
**3c**	N-Methylformamide	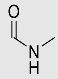	59.07	59.0371
**4a**	Acetamide	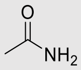	59.07	59.0371
**4b**	$[^{15}N]$Acetamide	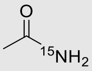	60.06	60.0341
**4c**	N-Methylacetamide	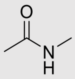	73.09	73.0528
**5**	Glycerol	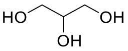	92.09	92.0473
**5a**	Sodium glyceryl phosphate	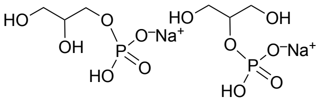	194.05	171.0064
**6**	*rac*-mono-Palmitoylglycerol (MPG)	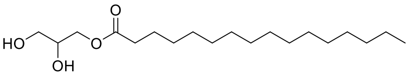	330.51	330.2770
**6a**	Palmitic acid (C16:0)	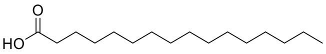	256.43	256.2402
**7**	*rac*-1,2-*bis*-Tridecanoyl glycerol (BTG)	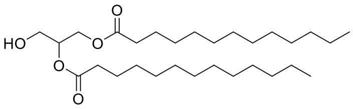	484.76	484.4128
**7a**	Tridecanoic acid	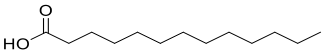	214.35	214.1933
**7b**	Dodecanoic acid	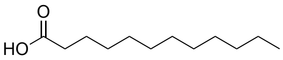	200.32	200.1776
**7c**	Undecanoic acid	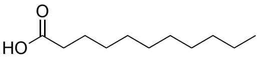	186.29	186.1620
**7d**	Decanoic acid	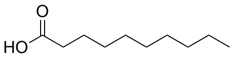	172.27	172.1463
**8**	*rac*-1,2-Dioleoylglycerol (DOG)	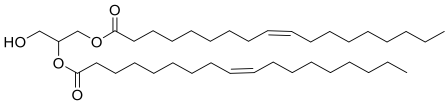	621.00	620.5380
**9**	Dodecan-1-ol	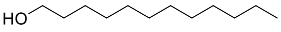	186.34	186.1984
**9b**	Sodium 1-dodecyl phosphate	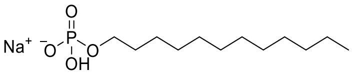	288.38	266.1647
**10**	Geraniol	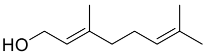	154.25	154.1358
**11**	Adenosine	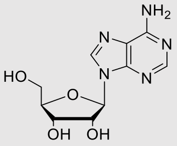	267.25	267.0968
**11a**	Adenosine 5'-monophosphate disodium salt	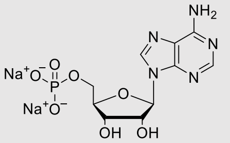	391.19	345.0485
**11b**	Adenosine 5′-diphosphate sodium salt	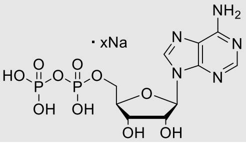	427.20	424.0076

**11c**	Adenosine 5'-triphosphate disodium salt hydrate	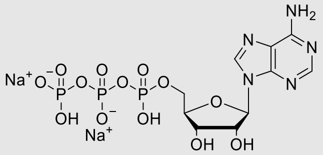	551.14	502.9666
**12**	Guanosine	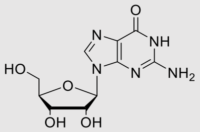	283.24	283.0917
**12a**	Guanosine 5'-monophosphate disodium salt	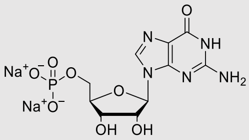	407.18	361.0434
**13**	Cytidine	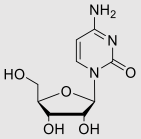	243.22	243.0855
**13a**	Cytidine 5'-monophosphate disodium salt	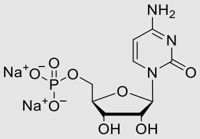	367.16	321.0373
**14**	Uridine	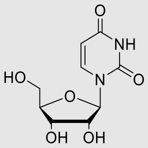	244.20	244.0695
**14a**	Uridine 5'-monophosphate disodium	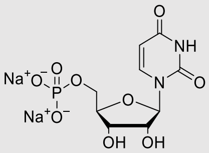	368.15	322.0213
**15**	Thymidine	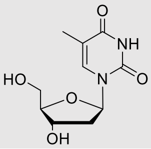	242.23	242.0903
**16**	Pyruvic acid	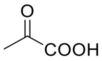	88.06	88.0160
**16b**	Cyclohexylammonium phosphoenolpyruvate	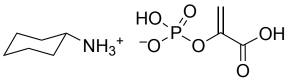	267.22	166.9751 ($C_3H_4O_6P^-$)
**17**	α,β-Isopropylidene-DL-glycerol	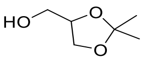	132.16	132.0786
**18**	*rac*-(2,2-Dimethyl-1,3-ioxolan-4-yl)methyl palmitate	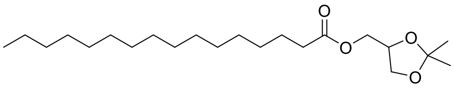	370.57	370.3083
**19**	*tert*-Butyldimethylsilyl-glycerol	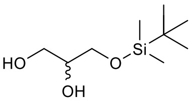	221.39	221.1573
**20**	*tert*-Butyldimethylsilyl-*rac*-1,2-*bis*-tridecanoyl glycerol	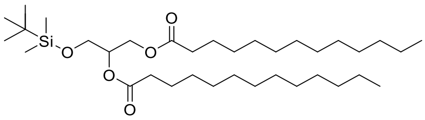	614.06	613.5227

**21**	*tert*-Butyldimethylsilyl-*rac*-1,2-dioleoylglycerol	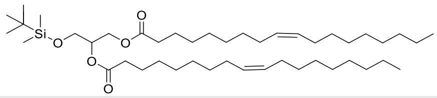	750.30	749.6479
**22a**	L-Alanine	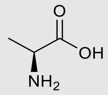	89.09	89.0477
**22b**	L-Valine	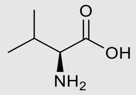	117.15	117.0790
**22c**	D-Valine	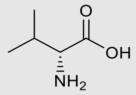	117.15	117.0790
**P$_i$**	NaH_2PO_4	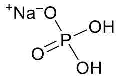	119.98	94.9551
**[$^{18}O_4$]P$_i$**	$NaH_2P[^{18}O_4]$	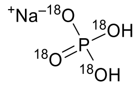	127.98	102.9720
**P$_i$** (dibasic)	Na_2HPO_4	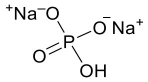	141.96	94.9551
**P$_i$** (tribasic)	$Na_3PO_4 \cdot 12\ H_2O$	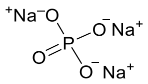	163.94 (anhydrous)	94.9551
**CP$_i$**	Dilithium carbamoylphosphate hydrate	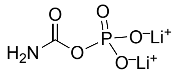	152.88 (anhydrous)	138.9682 ($CH_2NPO_5^{2-}$)
**cTMP**	Trisodium cyclic trimetaphosphate	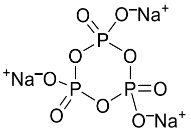	305.89 (anhydrous)	236.8772 ($P_3O_9^{3-}$)
**SP$_i$**	Trisodium thiophosphate monohydrate ($Na_3SPO_3 \cdot H_2O$)	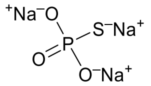	180.01 (anhydrous)	110.9322 (PSO_3^{3-})
**Vivianite**	Ferrous phosphate octahydrate ($Fe^{2+}{}_3(PO_4)_2 \cdot 8\ H_2O$)	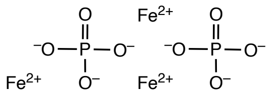	506.61	94.9551 (PO_4^{3-})
**Struvite (synthetic)**	Ammonium magnesium phosphate hexahydrate ($NH_4MgPO_4 \cdot 6\ H_2O$)	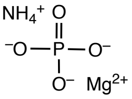	245.41	94.9551 (PO_4^{3-})
**Canaphite analog**	Calcium pyrophosphate ($Ca_2P_2O_7$)	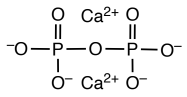	254.05 (anhydrous)	173.9141 ($P_2O_7^{4-}$)
**$Et_3N \cdot HF$**	Triethylamine *tris*-hydrofluoride	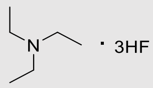	161.21	102.2005 (Et_3NH^+)
**DMAP**	4-(Dimethylamino)pyridine	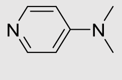	122.17	122.0844
**EDC·HCl**	N-(3-Dimethylaminopropyl)-N'-ethylcarbodiimide hydrochloride	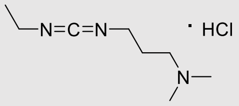	191.70	156.1495 ($C_8H_{18}N_3^+$)

* for salts: molecular mass of the larger ion

1.2. NMR equipment, signal description, and quantification methods

NMR spectra were recorded in $CDCl_3$, fully deuterated dimethyl sulfoxide (DMSO-*d*6) and D_2O on *Bruker Avance 300*, *400* and *500* spectrometers equipped with a standard BBFO probe. ^1H were recorded with 2 sec relaxation time and excitation sculpting when presaturation was needed. ^{31}P nuclei through qNMR spectra were performed using 128 scans, inverse gated decoupling with 90-degree pulses and 60 sec relaxation time (longer than measured on $\mathbf{SP_i}$ having the slowest relaxation time) to ensure quantitative measurement of all ^{31}P nuclei. ^{31}P-^1H HMBC spectra were run with the standard pulse program from *Bruker*, using 512 increments with 100 ppm spectral width in F1, 4 scans and 1.6 sec relaxation delay giving 1 h experiment time. ^{15}N experiments on enriched compounds were run using a 90 degrees single pulse, with 30 sec relaxation delay. Additional $^{31}P\{^{13}C,^1H\}$, $^{13}C\{^{31}P,^1H\}$ JMOD NMR, together with ^{31}P-^{13}C and ^{31}P-^{15}N HMBC spectra, were taken on a *Bruker Avance NEO 600* MHz spectrometer equipped with a quadruple resonance (^1H, ^{13}C, ^{31}P, ^{15}N) inverse cryoprobe (QCI 5 mm) at the *Institut de Chimie de Toulouse, Université Paul Sabatier*.

Chemical shifts of solvents ($CDCl_3$: δ_H = 7.26 and δ_C = 77.23 ppm; DMSO-*d*6: δ_H = 2.50 ppm (quintet, $J_{H,D}$ = 1.9 Hz) and δ_C = 40.0 ppm (septet, $J_{C,D}$ = 32 Hz); D_2O: δ_H = 4.65) served as internal references. Signal shapes and multiplicities are abbreviated as br (broad), s (singlet), d (doublet), t (triplet), q (quartet), quint (quintet) and m (multiplet). Where possible, a scalar coupling constant J is given in Hertz (Hz). ^{31}P NMR chemical shifts were referenced to NaH_2PO_4 (δ_P = 0.00 ppm). Although ^{13}C NMR chemical shifts were usually referenced to DMSO (δ_C = 40.0 ppm), in the case of spectra taken in H_2O/D_2O the signal of urea (**2a**) at δ_C = 160.50 ppm was considered as an internal reference.

The quantification of products was tested and performed using 1D qNMR methods with ^1H and ^{31}P nuclei. The quantities of the main groups of phosphorylated products were estimated using relative quantification, compared to the signal of inorganic phosphate by $^{31}P\{^1H\}$ qNMR. In most experiments, phosphorylated organic molecules were produced as a result of synthesis and were not initially present in the mixture. Therefore, the quantity of inorganic phosphate at the end of the reaction corresponded to its unreacted leftovers (if present). In conclusion, the relative quantities of organic molecules resulted from the conversion of inorganic phosphate into them.

The regions of interest were integrated and compared to the integral of inorganic phosphate, with the total sum set to 100 %. Due to the large number of different experiments, we decided not to use an internal standard and instead relied on a relative quantification technique. This approach facilitated the comparison of different experimental conditions and the ratios of initial reactants.

The results of this quantification obtained from $^{31}P\{^1H\}$ NMR spectra included only phosphorylated organic products and do not contain all other possible by-products of the reaction. Multiplicity analysis revealed that the majority of the signals appeared as singlets in $^{31}P\{^1H\}$ NMR spectra, thus, each corresponded to a single molecule available for integration, except for di-, tri-, and polyphosphate products. In other words, we define throughout this work "Conversion % $\mathbf{P_i}$" as the percentage of the inorganic phosphorous source $\mathbf{P_i}$, $\mathbf{SP_i}$, $\mathbf{PP_i}$ (canaphite analogue) or **cTMP** transformed into organic phosphates of any kind (acyclic phosphate monoesters and diesters, cyclic diesters, organic diphosphates and triphosphates etc). In the time-dependent studies of the urea-assisted phosphorylation of glycerol (**5**) and MPG (**6**) we name this conversion "$\mathbf{P_i}$ consumption" to distinguish it from the degradation of urea and the formation and degradation of carbamates and cyclic carbonates as detected by ^{13}C NMR spectroscopy.

The signal-to-noise ratio (SNR) of qNMR spectra was systematically evaluated and measured using the MNova software for experiments of the kinetic study of urea-assisted phosphorylations, as well as all other ^{31}P qNMR of the phosphorylation of **5** and **6** (Section 7.1-7.4). The kinetics results are presented in Section 5 and were incorporated in the corresponding calculations, figures, and tables. Because of the large amount of data and number of signals in the qNMR spectra concerning Sections 7.1-7.4, there the SNR of the largest signal (< 10,000) and that of $\mathbf{P_i}$ were added as Supplementary Dataset files named after the corresponding figure numbers. Whenever this SNR was very low for the lipophilic samples due to aggregation or phase separation issues, we noted it in the supplementary text.

With the exception of the mixtures containing dodecan-1-ol (**9**, Section 7.9), all other ^{1}H NMR spectra appeared to be too complex for their reliable interpretation and quantification of the components by qNMR. In the kinetic study (Section 5), the relative quantities of glyceryl carbamates, cyclic carbonates, and other reaction by-products were evaluated based on the urea consumption, the ^{13}C NMR signal of which was never crowded or overlapped by others, and the isotopologs could be easily deconvoluted. This approach, however, was difficult or impossible to apply when calculating conversions relative to any starting alcohol other than **9**, because of overlapping signals in the region of interest of their, both, ^{13}C or ^{1}H NMR signals.

1.2.1. Sample preparation for NMR spectroscopic analysis

The NMR sample preparation for the crude mixtures depended on the starting alcohol that was used in the reaction. In cases where the initial alcohol was liquid (glycerol, geraniol, dodecanol and DOG) and the crude mixture had low heterogeneity, fewer preparation steps were needed than for MPG and BTG that were difficult to dissolve even in DMSO-*d*6. In the case of nucleosides, the only solvent used was D_2O. Work-up of the crude reaction mixtures obtained using **5, 8-10 and 16** as a starting molecule: the whole reaction mixture was dissolved in 1000 µL DMSO-*d*6, vortexed at a medium rate for 20-30 seconds and centrifuged for 120 seconds at ~ 2000 x g. The final extract was filtered off and then placed in an NMR tube for analysis. Work-up of the crude reaction mixtures obtained using compounds **6 and 7** as a starting molecule: to about 50 mg of the reaction mixture were added 1000 µL DMSO-*d*6, the mixture was sonicated for 60 seconds, vortexed at a medium rate for 20-30 seconds and centrifuged for 120 seconds at ~ 2000 x g. The final extract was filtered off and then placed in an NMR tube for analysis. Work-up of the crude reaction mixtures obtained using compounds **11-15** as a starting molecule: about 50 mg of reaction mixture were added 1000 µl D_2O, the mixture was sonicated for 60 seconds, vortexed at a medium rate for 20-30 seconds and centrifuged for 120 seconds at ~ 2000 x g. The final extract was filtered off and placed in an NMR tube for analysis.

1.3. MS and LC-MS material, equipment and methods

MS-quality ultra-pure water (*Elga*®) and the following chemicals were used: acetonitrile ≥ 99.9 % *Optima* LC/MS grade, methanol ≥ 99.9 % *Optima* LC/MS grade, formic acid 99.0 % *Optima* LC/MS grade, DCM > 99.8 % for HPLC stabilised with amylene.

For direct injection analyses, samples were diluted 100 times in a solvent mixture (46.1 % MeOH, 38.4 % DCM, 15.4 % ultra-pure water and 0.1 % formic acid) and injected at 10 µL/min using a syringe pump. The low-resolution mass spectrometry (LRMS) analyses were performed using an ion trap (*AmaZon SL*, *Bruker*) equipped with an electrospray ion source (ESI) operated in positive or negative ion mode. The capillary voltage was set to ± 4500 V, the nebulizer gas was set to 3.4-4.8 bar (50-70 psi), the dry heater was set to 200 °C and the dry gas was set to 4-9 L/min, *m/z* range 50-1000 Da. The data were processed by the *DataAnalysis 5.2* software from Bruker. The high-resolution mass spectrometry (HRMS) analyses were performed using a high-resolution hybrid quadrupole-time of flight mass spectrometer (*Impact II*, Bruker) also equipped with an electrospray ionization source (ESI). The source parameters and the processing software were the same as mentioned above. The calibration of high-resolution data was performed with a sodium formate solution.

The liquid chromatography-high resolution mass spectrometry (LC-HRMS) analyses were performed by a UHPLC system (*Ultimate 3000*, *Thermo Scientific*) coupled with the hybrid Quadrupole – Time-of-Flight (Q-TOF) mass spectrometer (*Impact II, Bruker*). The samples were not diluted before injection. The injection volume was adjusted to the concentration of the sample and was in general 0.5 µL. The columns, eluents and gradients used were 1) reversed-phase ultrahigh-performance chromatography (RP-UHPLC) for mixtures containing **5**: *Luna Omega* Polar™ C18, 50 x 2.1 mm (1.6 µm), A = 0.1 % formic acid in ultra-pure water, B = 0.1 % formic acid in acetonitrile/methanol (ACN/MeOH) 1:1, ambient temp., flow rate 0.5 ml/min, 0-5 min 0 % B, 5-10 min 0-100 % B, 10-12 min 100 % B, 12.0-12.2 min 100-0 % B, 12.2-15 min 0 % B ; 2) RP-HPLC for mixtures containing nucleosides and nucleotides (**11-14a**): *Luna Omega* Gemini™ C18, 250 x 4.6 mm (5 µm), A = 5 mM ammonium acetate pH 8.0,

B = ACN/A 95:5, ambient temp., flow rate 1 ml/min, 0-2 min 2 % B, 2-29 min 2-20 % B, 29-30 min 20-80 % B, 30-34.5 min 80 % B, 34.5-35 min 98-2 % B, 35-45 min 2 % B; 3) hydrophilic ion liquid chromatography (HILIC) for mixtures containing glycerol (**5**) and glyceryl phosphates: *Luna Omega* Sugar™, 100 x 2.1 mm (3 µm), A = 8 mM ammonium acetate in ultra-pure water, B = ACN, isocratic elution A/B = 55:45, ambient temp., flow rate 0.5 ml/min.

1.3.1. Sample preparation for MS analysis (LC-MS, LRMS, HRMS) of the crude reaction mixtures, trap 1 and trap 2 contents

Sample preparation for MS analyses of the crude extracts obtained from reactions with starting molecules **5** to **16**: about 1-2 mg of the solid reaction mixture was dissolved in methanol or water (**11-15** in water); sonicated for 60 seconds, centrifuged for ~ 2000 x g for 120 seconds; if necessary, filtered through organic filter *Millipore* 0.45 µm PTFE (hydrophobic) and the final extract collected for analysis.

Analysis of trap 1 (Supplementary **Fig. 12**) consisted of studying the solution and precipitate separately that accumulated by the end of the reaction. In order to collect the precipitate, all the content of trap 1 was filtered under vacuum through a fritted glass (porosity 4) and dried for at least 24 h. A part of the powder (1-2 mg) was dissolved in methanol (1 ml), filtered off and placed in a vial for MS analysis. Samples of the separated solution (2-3 ml filtrate) were evaporated and the residue was dissolved in methanol (1 ml) filtered off and placed in a vial for MS analysis.

For analysing the content of trap 2 (Supplementary **Fig. 12**) a 1-2 ml sample was taken, usually shortly before the end of the experiment, while applying an enhanced argon flow in order to avoid CO_2 contamination from the air. The first step was to quench unreacted PhMgBr and protonate benzoate and other organic salts with an excess formic acid (1-2 ml). Then, the solution was evaporated and the residue was dissolved in MS-grade methanol (1 ml), filtered off and placed in a vial for MS analysis.

1.4. HPLC equipment, material and method for the analysis of MPG phosphorylation

All high-performance liquid chromatography (HPLC) analyses were performed using reversed-phase elution on a LC20 system from *Shimadzu* (high-pressure mixing double-pump system; max. flow rate 20 ml/min for each pump). The system was equipped with a DGU-20A3R degasser (*Shimadzu*, up to 10 ml/min per line), separate analytical and preparative capillary lines, two separate injectors from *Rheodyne* (100 µL and 2 ml loops). For each analysis 5 µL were commonly injected. The eluted compounds were observed by ultraviolet (UV) detection using two wavelengths at a time (SPD-20A, *Shimadzu*), fluorescence detection (RF-20A XS, *Shimadzu*) and by laser-driven evaporative light scattering detection (ELSD, *Sedex 90 LT*™, *Sedere*®). The three detectors could be used separately, pairwise or all together in the order mentioned above.

Crude mixtures produced in phosphorylation reactions such as lipids were analysed using a phenyl-hexyl column (*Kinetex*™, 2.6 µm, 100 × 3 mm, *Phenomenex*®). The lipids were detected by ELSD on a device operating at 40°C and a gain of 10 or 11. Phases A (0.1% formic acid in H_2O) and B (0.1% HCOOH in acetonitrile (ACN) were used in a linear binary gradient from A/B 50:50 to 100% B at 0.8 ml/min in 10 min at 25°C.

1.5. Quantum theoretical methods

It is well established that density functional theory (DFT) predicts molecular structures and harmonic vibrational frequencies of substantially higher accuracy than obtained via Hartree-Fock (HF) calculations[1,2]. All calculations in this work were performed with the *Gaussian09* suit of programs[3]. In the present work, Truhlar's Minnesota functional M06-2X[4] in combination with the 6-31G(d,p) basis set[5] were used to fully optimise the geometries of reactants and products, and also were used to search for all possible geometries of transition states and intermediates. The M06-2X functional is highly nonlocal with double exchange (2X) and has been used successfully for a combination of main group thermochemistry, kinetics, and noncovalent interactions[6,7]. The performance of the method and basis set has been validated by the comparison to the energies of single points calculated with the most accurate CCSD method[8] and the more extended 611++G(2p,2d) basis set[9]: M06-2X/6-311++ G(2d,2p)/

/M06-2X/6-31G(d,p) and CCSD/6-311++G(2d,2p)//M06-2X/6-31G(d,p). The differences of single-point energies between both methods using the extended basis set were very small; the CCSD energies were higher by 3.7-13.6 % for the intermediate states and 1.3-12.2 % for the transition states. Vibrational frequency calculations were carried out to ensure that the geometries obtained from M06-2X/6-31G(d,p) were indeed local minimal or saddle points on the potential energy surfaces and to determine the zero-point vibrational and thermal corrections to the Gibbs free energies. The Intrinsic Reaction Coordinate (IRC)[10] calculations were performed to confirm the correct connections between reactants, transition states, intermediates, and products on the potential energy surfaces.

1.6. Synthesis methods

1.6.1. Synthesis of racemic mono-palmitoylglycerol (MPG) 6

1) Synthesis of rac-(2,2-dimethyl-1,3-dioxolan-4-yl)methyl palmitate (**18**) following a published procedure[11]:

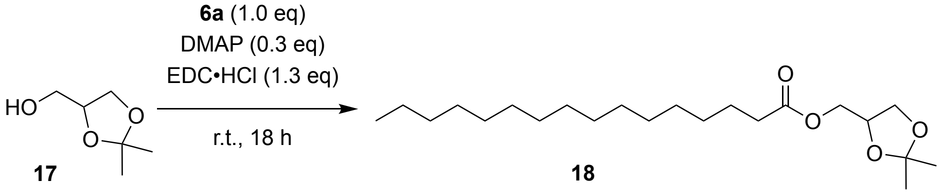

3.7 g α,β-isopropylidene-DL-glycerol (28.6 mmol, **17**) were dissolved in dry dichloromethane (75 ml DCM) and 7.3 g palmitic acid (28.6 mmol, **6a**) were added. The solution was cooled to 0 °C using an ice bath, DMAP (1.0 g, 8.6 mmol) and EDC·HCl (7.1 g, 37.2 mmol) were added together. The resulting solution was left under vigorous stirring at ambient (room) temperature (r.t.) for 18 h. A quantity of 150 ml of a saturated $NaHCO_3$ solution was added to quench the reaction. The product was extracted with DCM (2 x 100 ml), the combined organic phases were dried over anhydrous $MgSO_4$ and the crude material obtained after evaporation of the solvent was chromatographed over SiO_2 with petrol ether-ethyl acetate (PE:EtOAc 99:1 to 85:15 *v/v*) yielding **18** as a white powder (9.99 g, 94 %).

2) Synthesis of rac-1-palmitoyl-glycerol (**6**)*:*

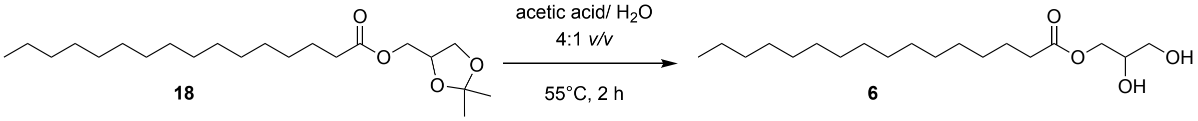

Racemic (2,2-dimethyl-1,3-dioxolan-4-yl)methyl palmitate (9.99 g, 26.9 mmol, **18**) was dissolved in a mixture of acetic acid and water (AcOH/H_2O 100:25 ml) and kept under vigorous stirring at 55 °C for 2 h. The solution was cooled to r.t. and 150 ml of saturated $NaHCO_3$ were added dropwise until the solution was neutralised. The product was then extracted with EtOAc (2 x 100 ml). The combined organic phases were washed with brine (100 ml) and dried over anhydrous $MgSO_4$. The resulting solution was evaporated, yielding MPG as a white powder (8.90 g, 99 %, **6**), mp. 65-68 °C. The product was characterised by ^{1}H NMR spectroscopy and LRMS (Supplementary **Fig. 1**-Supplementary **Fig. 2**).

1.6.2. NMR and MS characterisation of MPG (6)

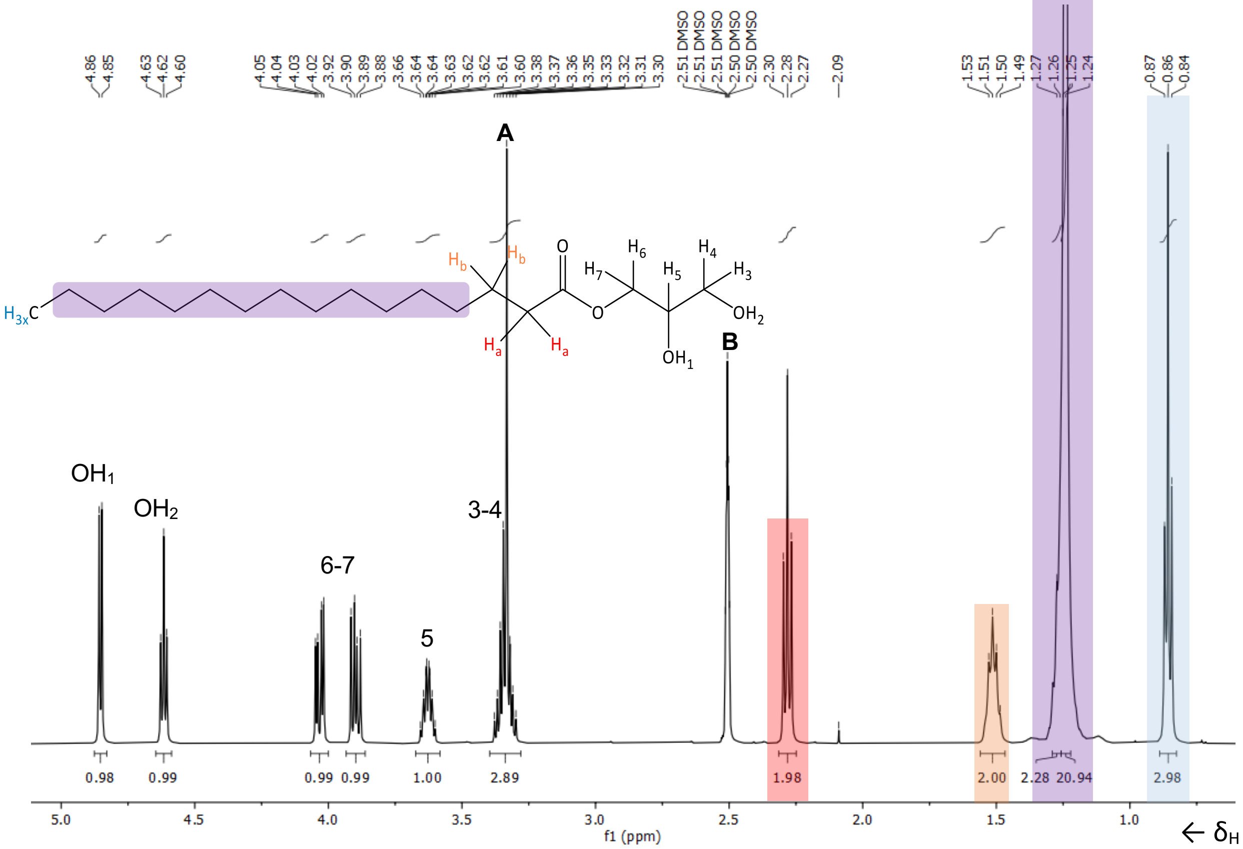

Supplementary Fig. 1. ^{1}H NMR (500 MHz, DMSO-*d*6) 0.5 mM MPG (**6**): δ_H = 4.85 (*d*, J = 5.2 Hz, 1H), 4.62 (*t*, J = 5.7 Hz, 1H), 4.03 (*dd*, J = 11.1, 4.2 Hz, 1H), 3.90 (*dd*, J = 11.1, 6.5 Hz, 1H), 3.66-3.60 (*m*, 1H), 3.38-3.30 (*m*, 3H), 2.28 (*t*, J = 7.4 Hz, 2H), 1.53-1.49 (*m*, 2H), 1.27-1.24 (*m*, 24H), 0.86 (*t*, J = 6.8 Hz, 3H). **A** = HDO (br s), **B** = DMSO-*d*5 (quint).

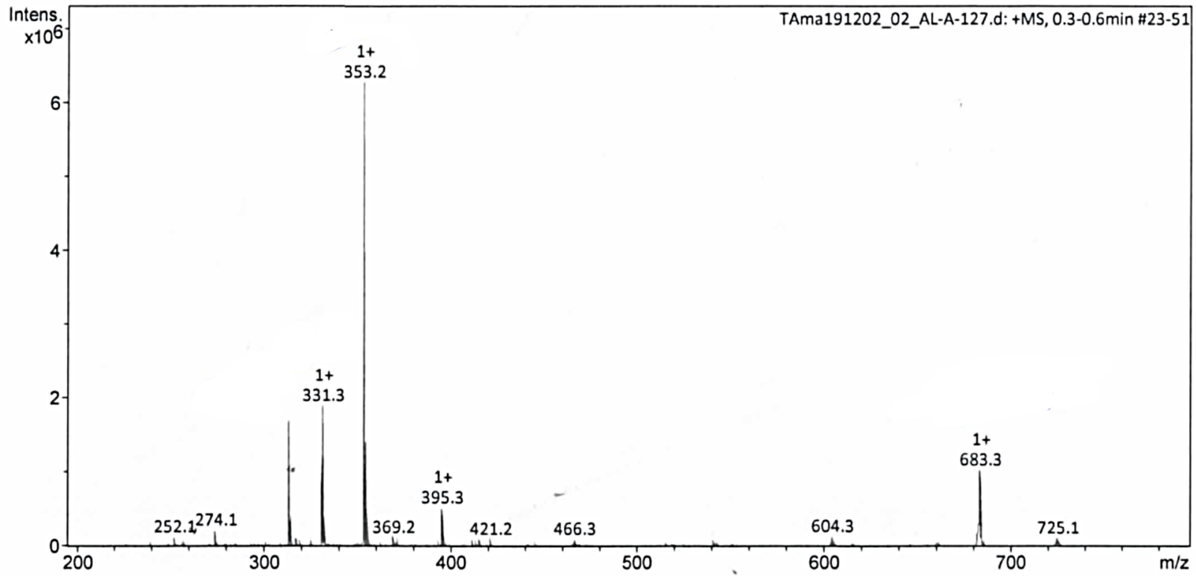

Supplementary Fig. 2. Low-resolution positive ion-mode mass spectrum of MPG (**6**), *m/z*: 331.3 (**6**) $[M+H]^+$; 353.2 $[M+Na]^+$; 683.3 $[2M+Na]^+$.

1.6.3. Synthesis and characterisation of racemic *bis*-tridecanoyl and dioleoyl glycerol

Racemic BTG (**7**) and DOG (**8**) were obtained following published procedures[11,12] described below.

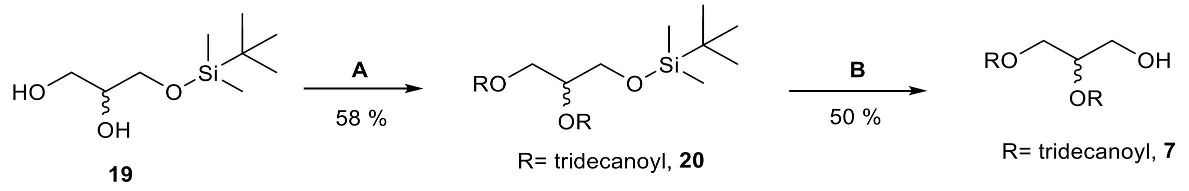

Supplementary Fig. 3. Synthesis of **7**. **A** – **19** (1 eq), **7a** (2.1 eq), DMAP (2 eq), EDC·HCl (2.2 eq); **B** – $Et_3N{\cdot}HF$ (5 eq).

1.6.3.1. Synthesis of TBDMS-*rac*-BTG (20)

In a 250 ml argon-flushed round bottom flask containing (**19**, 3 g, 0.013 mol) and **7a** (7 g, 0.03 mol) in dry DCM (50 ml) were added DMAP (0.4 g, 0.03 mol) and EDC·HCl (5 g, 0.04 mol) at 0 °C. The solution, once reached r.t., was stirred for 16 h after which thin-layer-chromatography (TLC) using diethyl ether and cyclohexane (Et_2O:Cy 8:1 *v/v*) showed complete consumption of **19**. 75 ml of water were added and the organic phase was extracted twice. The organic phases were dried over dry anhydrous Na_2SO_4 and the solvent was evaporated. Product **20** was isolated through flash chromatography (Et_2O:Cy 1:9 *v/v* to 3:7), $[\alpha]_D^{25}$ = 0.00 (*c* 0.1, $CHCl_3$), purity see Supplementary **Fig. 4**-Supplementary **Fig. 5**.

1.6.3.2. Characterisation of TBDMS-*rac*-BTG (20)

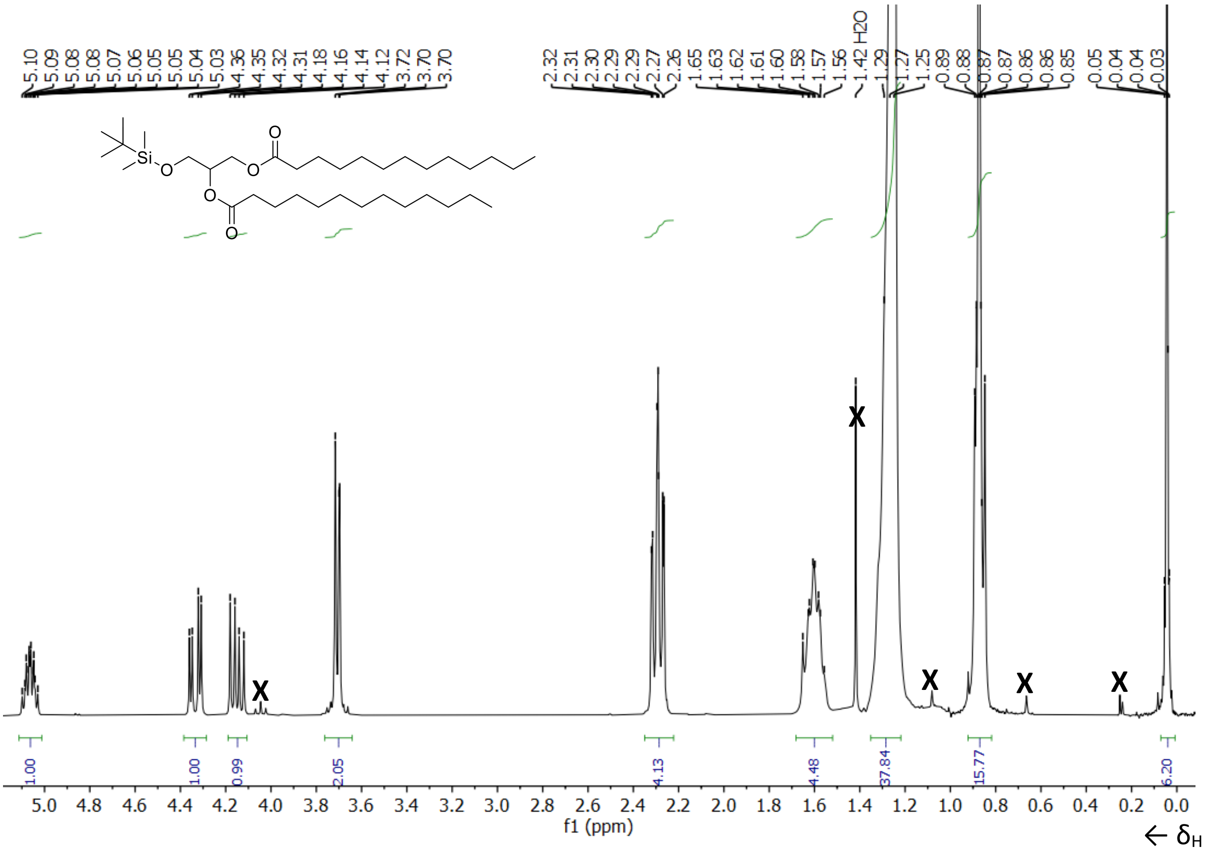

Supplementary Fig. 4. ^{1}H NMR (300 MHz, $CDCl_3$) TBDMS-*rac*-BTG (**20**): δ_H = 5.05 (*d quint*, 1H, J = 5.4, 2.5 Hz, C(2)H); 4.33 (*dd*, J = 11.9, 3.7 Hz, 1H, C(3)H_b), 4.15 (*dd*, J = 11.9, 6.3 Hz, 1 H, C(3)H_a), 3.66 (*d*, J = 5.7 Hz, 2 H, C(1)H2), 2.30 + 2.29 (2 *t*, J = 7.6 Hz, $\Delta\delta_H$ = 1.4 Hz, 4 H, 2 x C$\underline{H_2}$COOR), 1.65-1.68 (*m*, 4 H, 2 x C$\underline{H_2}$$CH_2$COOR), 1.20-1.35 (*m*, 36 H, 18 x CH_2), 0.85-0.89 (*m*, 15 H, 5 x CH_3 *t*Bu–Si, Me–Si), 0.04 (*m*, 6 H, 2 x C$\underline{H_3}$$(CH_2)_{11}$). **X** = impurities.

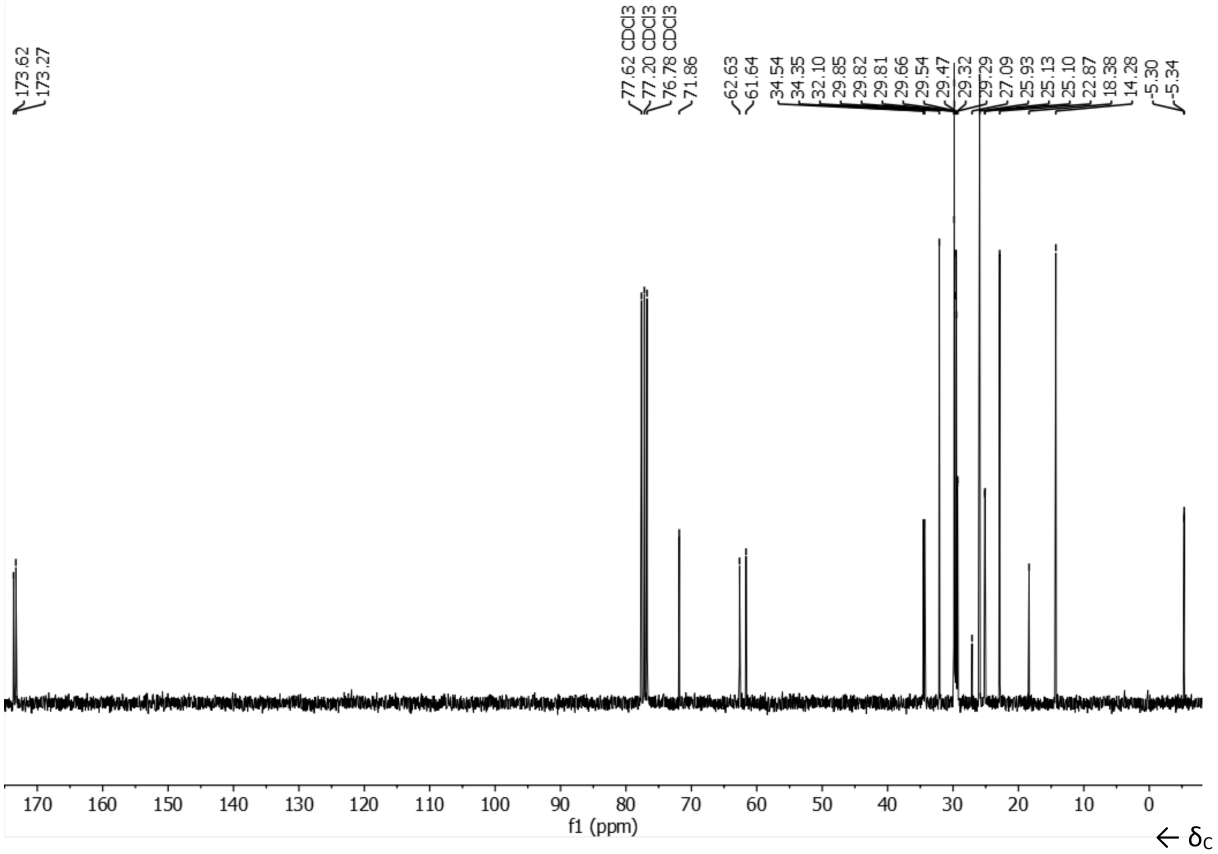

Supplementary Fig. 5. ^{13}C NMR (75.5 MHz, $CDCl_3$) TBDMS-*rac*-BTG (**20**): δ_C = –5.34, –5.30 (MeSi), 14.1 (CH_3), 18.2 (Cq ^tBuSi), 22.7 (CH_2), 25.0 (CH_2), 25.7 (3 x CH_3), 29.1-29.7 (series of CH_2), 32.0 (CH_2), 34.1 (CH_2), 34.4 (CH_2), 61.5 ($C(1)H_2$), 62.4 (C(2)H), 71.7 ($C(3)H_2$), 173.1 (C=O), 173.3 (C=O).

1.6.3.3. Synthesis of *rac*-BTG (7)

In a 250 ml argon-flushed round bottom flask containing 3.3 g **20** (0.01 mol) in a dry mixture of tetrahydrofuran and acetonitrile (THF/ACN 50 ml, 1:1 *v/v*) were slowly added 2 ml (5 eq) of $Et_3N \cdot HF$. The obtained pale-yellow solution was stirred overnight. TLC (Cy/AcOEt 2:1 *v/v*) showed complete consumption of the starting material. A saturated $NaHCO_3$ solution (160 ml) was added together with 50 ml DCM. The organic phase was extracted twice and the water phases were washed with more DCM. The combined organic phases were dried over anhydrous Na_2SO_4 and the solvent was evaporated. Flash chromatography using a step-gradient (AcOEt/Cy 1:9 to 3:7 *v/v*) furnished a mixture of 3.4 g 92 %-pure **7** (70 % yield) and 0.3 g Et_3NHF (according to ^1H NMR 22 mol% with respect to pure **7**) as a white solid, mp. 50-55 °C. A small fraction (60 mg) contained racemic mono-tridecanoyl glycerol (MTG) together with BTG. The main fraction of product **7** was characterised by polarimetry, $[\alpha]_D^{25}$ = 0.00 (*c* 0.1, $CHCl_3$), ^1H, ^{13}C NMR spectroscopies and LRMS (Supplementary **Fig. 6**-Supplementary **Fig. 8**).

1.6.3.4. Characterisation of *rac*-BTG (7)

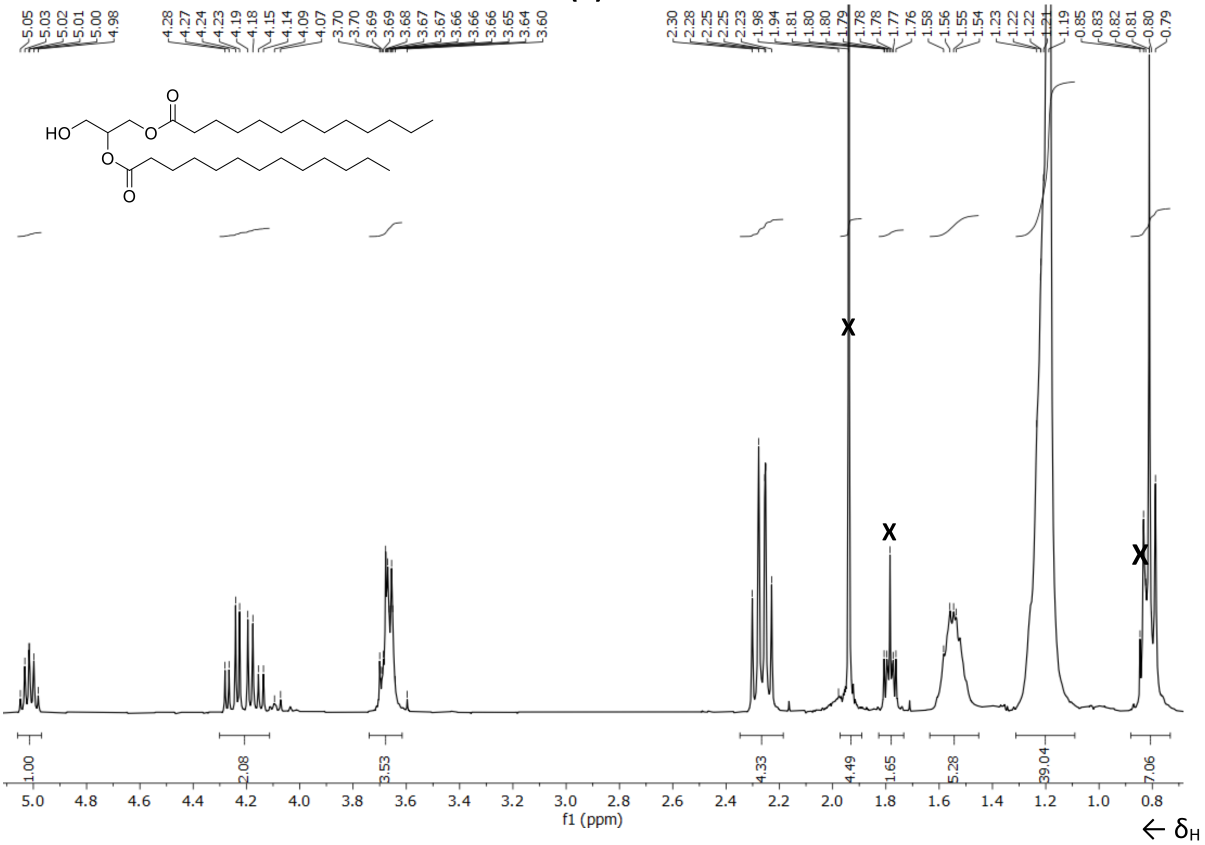

Supplementary Fig. 6. ^{1}H NMR (300 MHz, $CDCl_3$) BTG (**7**): δ_H = 5.02 (*dt*, 1H, J = 5.1, 0.5 Hz, C(2)H), 4.25 (*dd*, 1 H, J = 11.9, 4.5 Hz, C(1)H_b), 4.16 (*dd*, 1 H, J = 11.9, 5.6 Hz, C(1)H_a), 3.70 + 3.66 (*d* + br *s*, 3 H, J = 5.7 Hz, C(3)H_2 + OH), 2.28 + 2.25 (2 *t*, J = 7.3 Hz, 4 H, 2 x C$\underline{H}_2$COOR), 1.63-1.48 (*m*, 4 H, C$\underline{H}_2$$CH_2$COOR), 1.19 (*m*, 36 H, 18 x CH_2), 0.81 (2 *t*, J = 6.7 Hz, 6 H, 2 x CH_3), **X** – 1.94 (Et_3N$\underline{H}$F), 1.78 ((CH_3C$\underline{H}_2$)$_3$NHF, 0.83 (C$\underline{H}_3$$CH_2$)$_3$NHF.

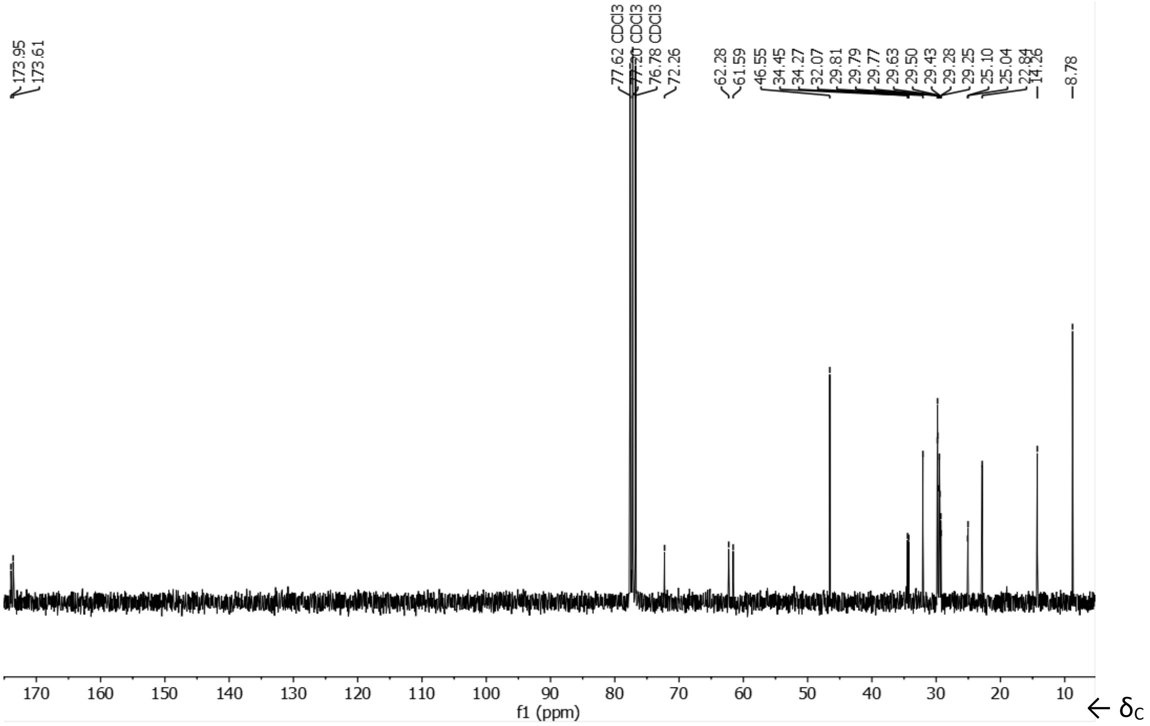

Supplementary Fig. 7. ^{13}C NMR (75.5 MHz, $CDCl_3$) BTG (**7**): δ_C = 14.2 (CH_3), 22.5 (CH_2), 27.0 (CH_2), 29.2 (CH_2), 29.2-29.8 (series of CH_2), 32.0 (CH_2), 34.2 (CH_2), 61.4 (C1), 62.0 (C2), 72.1 (C3), 173.5 and 173.8 (C=O).

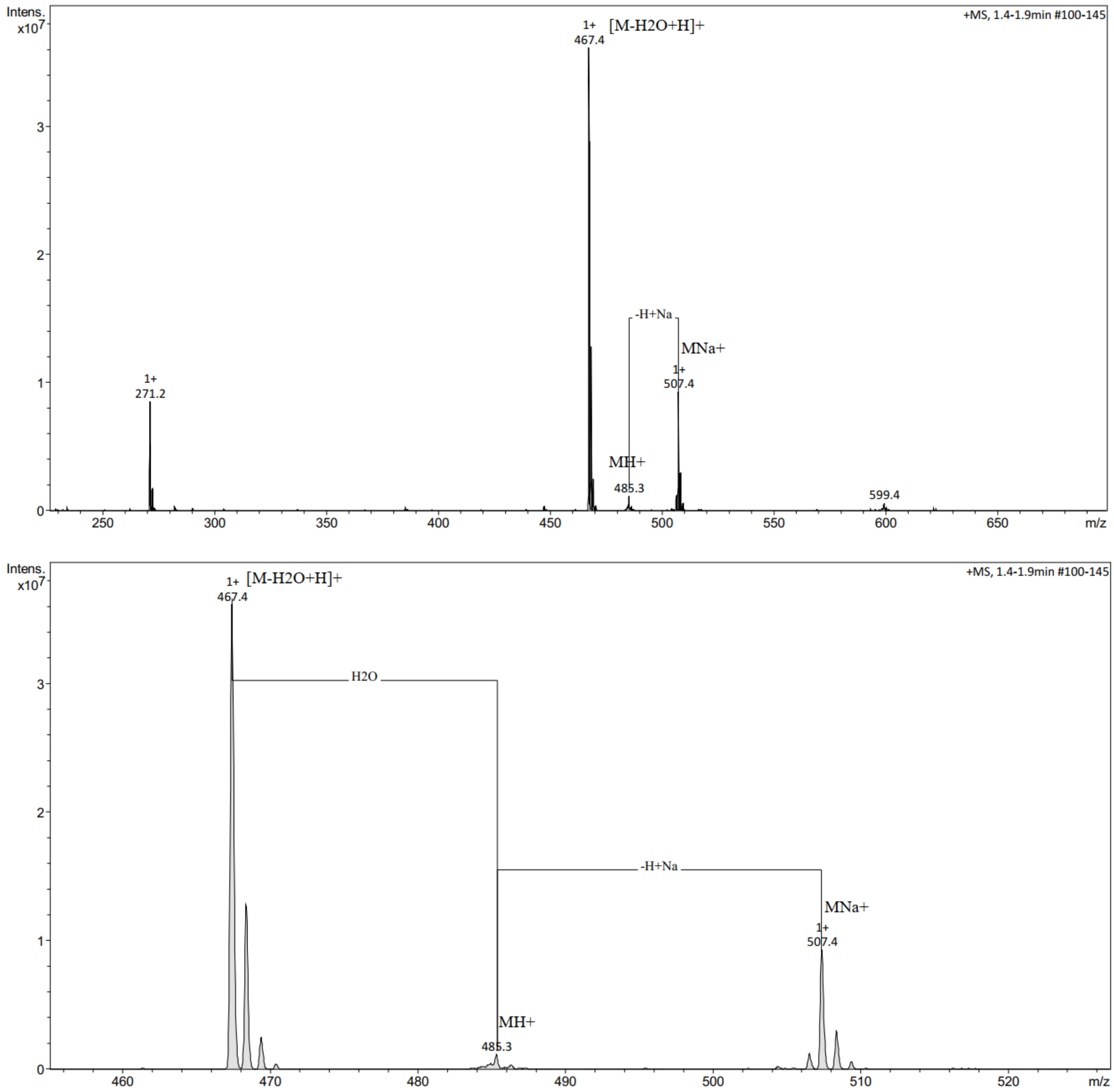

Supplementary Fig. 8. Low-resolution positive-ion mode mass spectrum of BTG (**7**). *m/z* 458.3 $[M+H]^+$, 467.4 $[M–H_2O+H]^+$, 507.4 $[M+ Na]^+$, 599.4 $[2M+Na]^+$. *m/z* 271.2 $[MTG]^+$ (*rac*-mono-tridecanoyl glycerol).

1.6.4. Synthesis of $NaH_2P[^{18}O_4]$ ($[^{18}O_4]P_i$)

1.6.4.1. $H_3P[^{18}O_4]$

5.00 g of normalised water nominally 97 %-enriched in ^{18}O {$H_2[^{16}O]$ monoisotopic mass = 18.0106 g/mol; $H_2[^{18}O]$ monoisotopic mass = 20.0148 g/mol; average molecular weight (0.03 x 18.0106 + 0.97 x 20.0148; MW = 19.9575 g/mol}, hence (5.00 g =) 250.5324 mmol water were placed in a thick-walled tube-shaped glassware flask (24 cm long x 4 cm diameter) equipped with a small magnetic stirrer (tare: 241.6 g). To this stirred water 10.4043 g PCl_5 (1/5 equiv., 49.90 mmol, MW = 208.24 g/mol) were added portion wise (approx. 250 mg per portion). During the addition, HCl fog developed in the flask which was periodically blown away with a short gentle argon stream. Care was taken that the upper half of the glassware remained at ambient temperature and that the portions were added slower during the second half (especially towards the end) of the PCl_5 addition, since the hydrolysis became more and more violent and HCl, perhaps Cl_2 and water (vapor, droplets) developed. Once that all the PCl_5 was added (after 25 minutes), the resulting colourless solution was weighed (220.4 g total weight of the flask corresponding to 5.8 g of the solution) and, after 45 min at rt (since the beginning of PCl_5 addition), the liquid was heated to 70° C (reached at 90 min total time). HCl bubbles developed continuously even when stirring was stopped for a moment. After 90 min at 70 °C the flask was cooled and weighed (total time 135 min) and the net weight was 4.928 g (expected 5.238 g = 100 %). The solution was cooled down and sampled (50 µl + 1 ml ACN) for ESI-HRMS. According to the negative-ion mode MS it contained residual amounts $H_2P[^{18}O_2]Cl_2$ (as *m/z* $P[^{18}O_2]Cl_2^-$) that could be ignored, since the neutralisation to $NaH_2P[^{18}O_4]$ was made 16 hours later during which the rest could hydrolyse to $H_3P[^{18}O_4]$. Detected isotope abundances:

Supplementary Table 2. Isotopolog ratio of phosphoric acid of two batches. Both products were used in the series of experiments.

Isotopolog	Ratio in a first batch, %	Ratio in a second batch, %
$H_3P[^{18}O_4]$	93.8	81.5
$H_3P[^{16}O^{18}O_3]$	6.0	15.8
$H_3P[^{16}O_2{}^{18}O_2]$	0.2	2.7

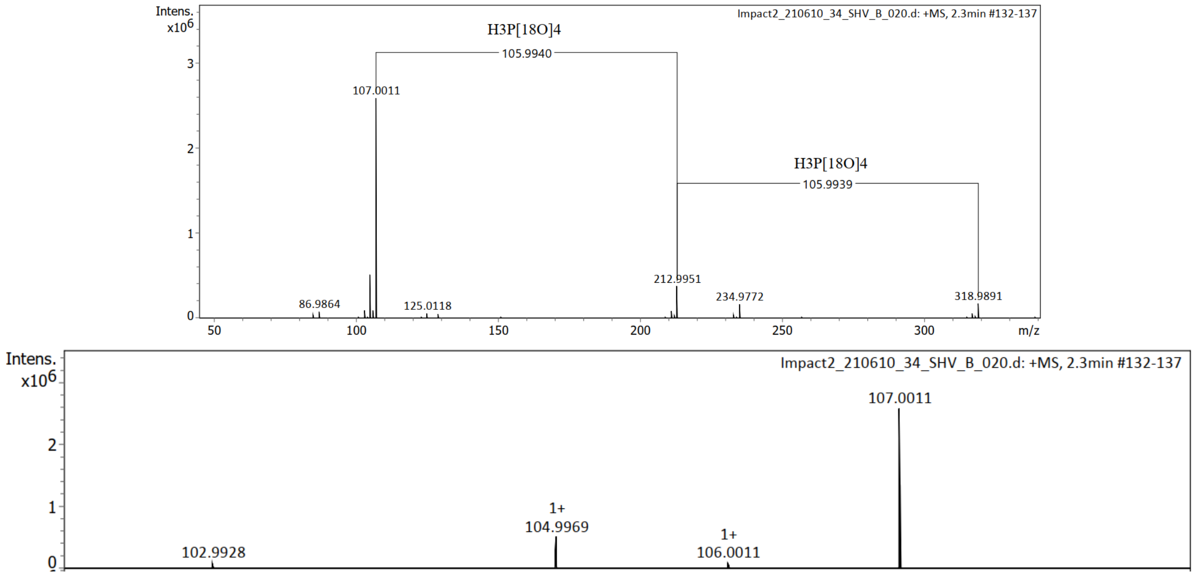

Supplementary Fig. 9. High-resolution positive-ion mode mass spectrum of $H_3P[^{18}O_4]$ (second batch).
A – Total ion spectrum: Phosphoric acid monomer, dimer and trimer (gas phase adducts).
B – Zoom into the main signal cluster from which the isotopolog ratios were determined (Supplementary **Table 2**): *m/z* 107.001 $[H_4P^{18}O_4]^+$, 104.9969 $[H_4P^{16}O^{18}O_3]^+$, 102.9928 $[H_4P^{16}O_2{}^{18}O_2]^+$, 212.9951 $[2M+H]^+$, 318.9891 $[3M+H]^+$.

1.6.4.2. $NaH_2P[^{18}O_4]$

A solution prepared by suspending 2.60 gr (24.0 mmol) of Na_2CO_3 in 8 ml of water (natural isotope abundance) was placed in a double neck 25 ml-round bottom flask equipped with a magnetic stirrer and a calibrated pH meter. The solution was placed in a thermostatic bath. The full amount $H_3P[^{18}O_4]$, prepared as described above, was transferred in a plastic syringe and was then slowly added (~ 50 µl/min) to the well stirred Na_2CO_3 suspension. The initial pH of the suspension was 11.03. The pH was continuously monitored. The pH changes from basic to moderately acidic provoked changes in the physical appearance of the solution/suspension, from milky to transparent until showing a new precipitation, probably of a mixture of Na_3PO_4, Na_2HPO_4 and/or NaH_2PO_4 that redissolved almost completely upon further addition of H_3PO_4. Once all the content of the syringe was added, the measured pH was 2.91 (= the pH value of a saturated commercial NaH_2PO_4 solution).

The obtained solution was evaporated under reduced pressure and the obtained residue was dried at 70 °C for 2 hours (weight loss 2.0 gr), at 105-150 °C for 4 hours, at 105 °C for 2 hours after which another weight loss of 1.9 % was detected. After the transfer of the salt to another vial, 4.998 g highly enriched $NaH_2P[^{18}O_4]$ could be used for further experimentation. A dry sample was analysed by ESI-HRMS to determine the isotope abundancies (Supplementary **Table 3**, Supplementary **Fig. 10**).

Supplementary Table 3. Isotopolog ratio of $NaH_2P[^{18}O_4]$ of two batches. Both products were used in the series of experiments.

Isotopolog	Ratio in a first batch, %	Ratio in a second batch, %
$NaH_2P[^{18}O_4]$	93.7	90.0
$NaH_2P[^{16}O^{18}O_3]$	6.1	9.2
$NaH_2P[^{16}O_2{}^{18}O_2]$	0.2	0.8

In much the same manner we have prepared 10 % ^{18}O-enriched NaH_2PO_3 from weakly enriched water containing 10 mol% $H_2[^{18}O]$. This compound was used for preliminary experiments in the presence of dodecan-1-ol (**9**).

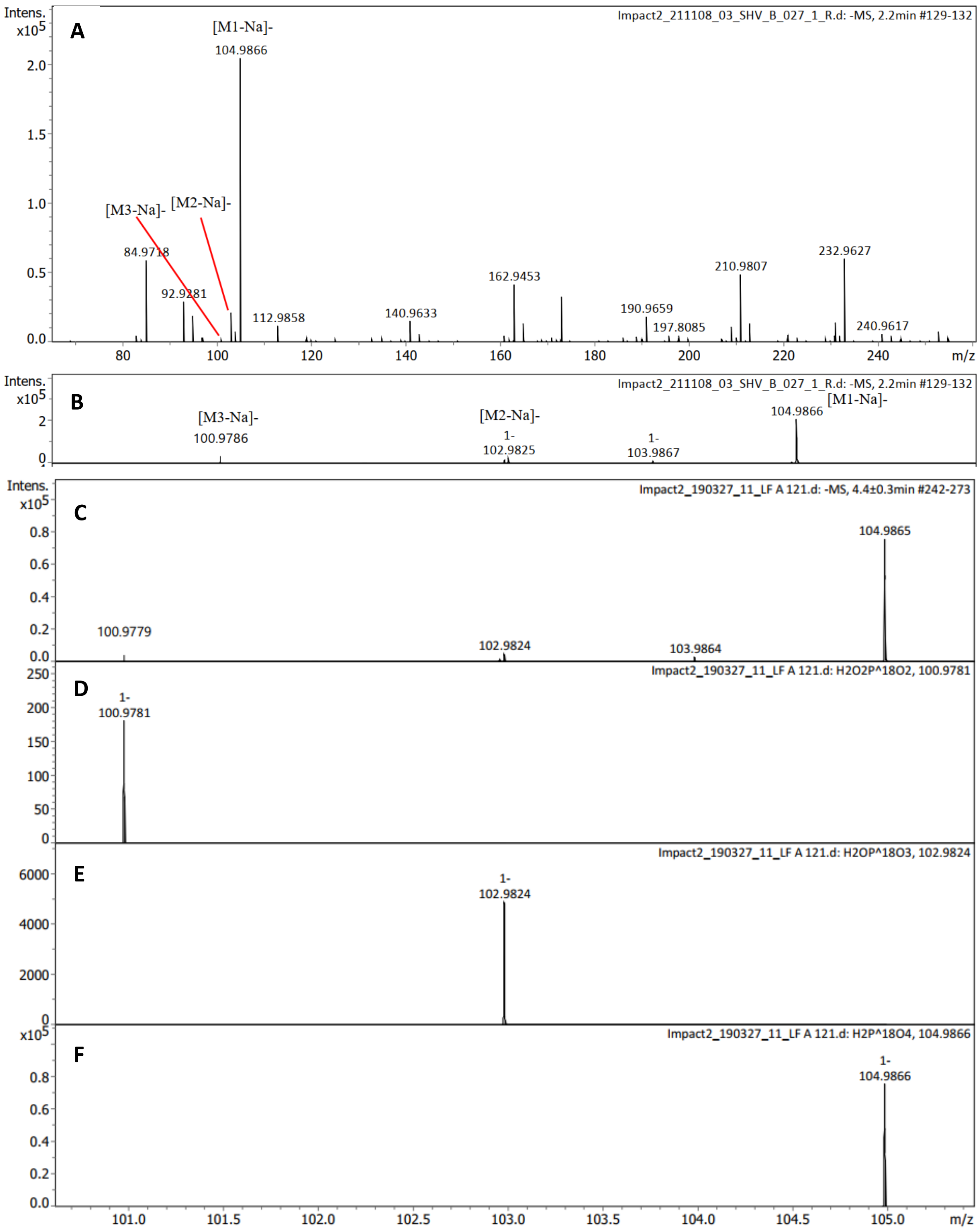

Supplementary Fig. 10. High-resolution negative-ion mode mass spectra of $NaH_2P[^{18}O_4]$.

A – Total ion spectrum (second batch).

B – Zoom into the main signal (second batch) from which the isotopolog ratios were determined (Supplementary **Table 3**), *m/z* 104.9866 $[H_2P^{18}O_4]^-$, 102.9825 $[H_2P^{16}O_1{}^{18}O_3]^-$, 100.9786 $[H_2P^{16}O_2{}^{18}O_2]^-$

C – Zoom into the main signal (first batch) from which the isotopolog ratios were determined (Supplementary **Table 3**), *m/z* 104.9865 $[H_2P^{18}O_4]^-$, 102.9824 $[H_2P^{16}O_1{}^{18}O_3]^-$, 100.9779 $[H_2P^{16}O_2{}^{18}O_2]^-$

D-**F** – Simulated mono-isotopic mass spectra of $[H_2P^{16}O_2{}^{18}O_2]^-$, $[H_2P^{16}O_1{}^{18}O_3]^-$ and $[H_2P^{18}O_4]^-$.

2. Design of experiments

2.1. Carousel™ reactions

"Carousel reactions" on a scale of 0.5-1.0 mmol alcohol were carried out using a *Carousel 12 Plus*™ Reaction Station (*R. B. Radley* & Co Ltd) depicted in Supplementary **Fig. 11**. Reactions were carried out at 60, 75, and 115 °C without using a reflux head or inert atmosphere with a time scale from 0.25 to 120 hrs. The final reaction temperature was reached incrementally with constant moderate interfusion of the contents using small magnetic stirrers. All phosphorylation reactions and control experiments were carried out in 50, 100, 250 ml-round bottom flask or in carousel glass tubes. All phosphorylation reactions under 'dry' (neat) conditions were in a solid or mixed solid-liquid state throughout the experiment. Control reactions under 'wet and evaporating' (first solubilised) conditions were initially clear aqueous solutions that eventually evaporated to the solid state. Stirring or swaying visibly worked only during the first 1-2 days and halted eventually owing to the mechanical resistance of the solid state of the mixtures. The time scale of 5 days was chosen to capture the formation of all main phosphorylation products along with by-products of urea degradation. The extended duration allowed us to track the accumulation and degradation of many late products beyond the degradation of urea and complete consumption of the initial inorganic phosphate source.

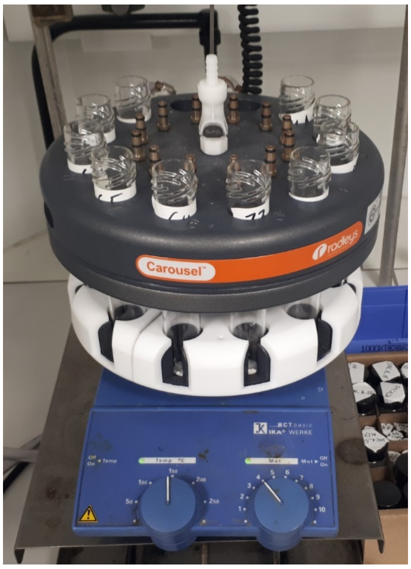

Supplementary Fig. 11. *Radley's Carousel 12 Plus*™ "Reaction Station" for studying the scope of prebiotic reactions.

2.2. Experimental set-up for tracing stable isotopes in urea-assisted phosphorylation reactions of glycerol and racemic monopalmitoylglycerol

In order to study the mechanism of urea-assisted phosphorylation reactions of glycerol and racemic monopalmitoylglycerol the experimental set-up was used as shown in Supplementary **Fig. 12**. The installation contained the reaction mixture in a round bottom flask placed in an oil bath on an electric heating plate, under steady relatively slow stirring. The reactor was connected to two traps with pipes under a slow argon flow (initially 1-2 bubbles in 10 seconds). Trap 1 was at room temperature filled with 0.1 mM solution of benzoyl chloride in dry toluene. Trap 2 contained 0.1 mM solution phenyl magnesium bromide, made of a 1.6 M PhMgBr stock solution in cyclopentyl methyl ether and diluted with anhydrous tetrahydrofuran (THF). Trap 2 was placed in a *KGW Isotherm™* Dewar bottle with an aluminium jacket filled with acetone and dry ice at temperatures between –78 and –50 °C. The Dewar was refilled every 12-16 hours with fresh portions of dry ice. Such a condition was needed to slow down the formation of by-products from the reaction of PhMgBr with THF as well as the phenylation of benzoate to benzophenone and trityl alcohol (Supplementary **Fig. 82**). The argon flow allowed to push forward the gasses that were produced in the phosphorylation reaction.

We then began to submit glycerol and MPG to the same prebiotic phosphorylation conditions as in the Carousel but used ^{13}C-labelled urea and ^{18}O-labelled sodium dihydrogenphosphate (3 to 6 mmoles of each reactant in equimolar ratio). The open reactor was connected to the aforementioned gas flow system that would allow to free the reaction mixture from all generated gasses through a gentle stream of argon gas. The gasses would bubble through a first trap 1 to efficiently trap any nucleophiles (NH_3, H_2O). Therein, gaseous, acidic electrophiles (CO_2, HNCO) could perhaps reside for some time but would eventually continue to be transported to electrophile trap 2. All products in the reactor, trap 1 (solution and precipitate) and trap 2 have been analysed by electrospray-ionisation (low- or high-resolution, HR/LR) mass spectrometry (ESI-MS) and HPLC-UV or UHPLC-ESI-MS. Depend-

ing on the experiment, the sampling of traps was made every 1-2 days (Supplementary **Fig. 54** and Supplementary **Fig. 71**). Extracts of the crude reaction mixtures were additionally analysed by ^{1}H and ^{31}P NMR spectroscopy, i.e., proton-coupled, proton-decoupled $^{31}P\{^{1}H\}$ and ^{31}P-^{1}H HMBC.

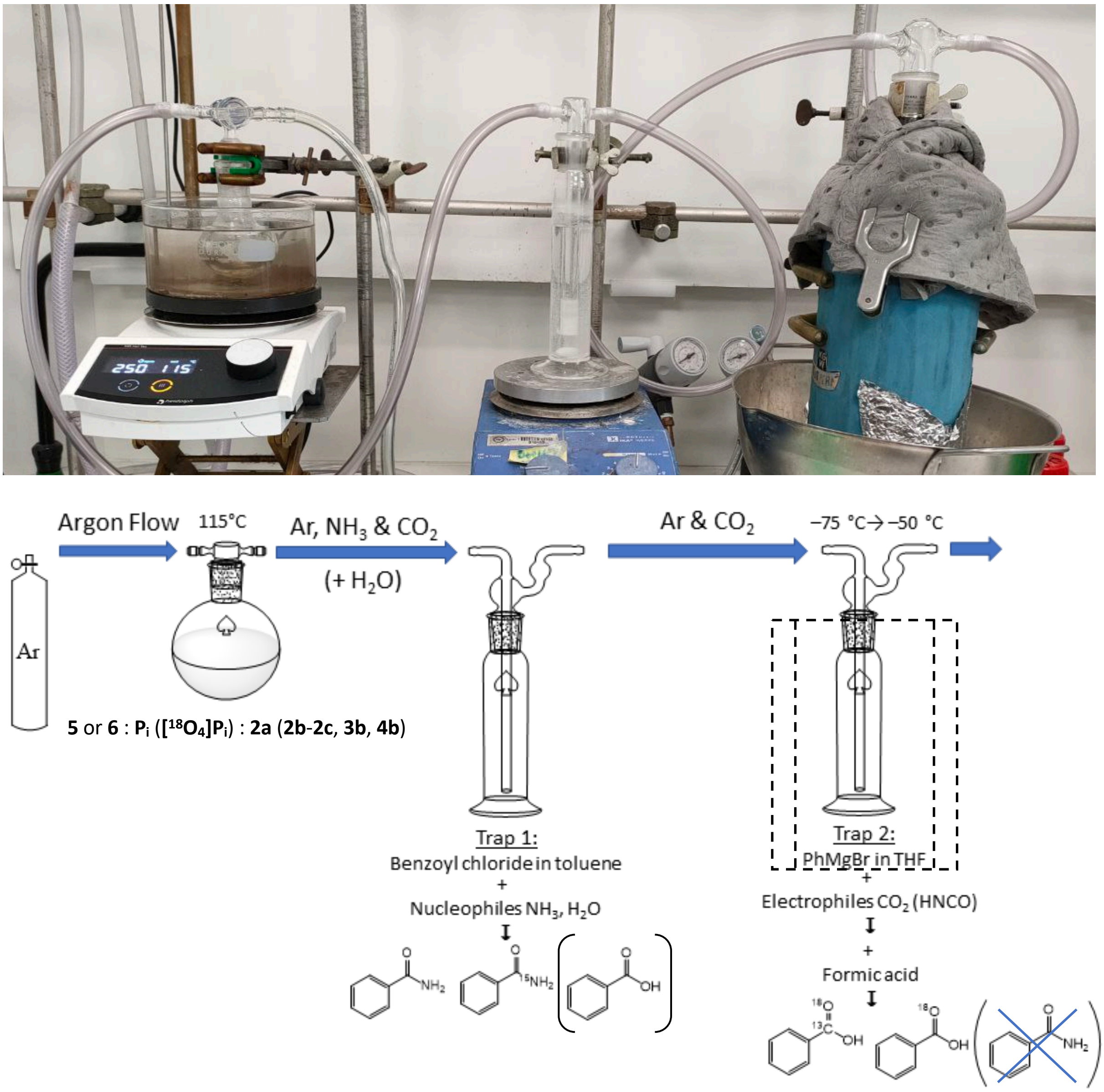

Supplementary Fig. 12. Experimental set-up for tracing stable isotopes in volatiles produced in prebiotic reactions. **A** – Photography of the laboratory installation. **B** – Schematised set-up and expected reactions in traps 1 and 2. In parentheses: molecules found in very low amounts (H_2O trapped as benzoic acid in trap 1) or not at all (HNCO to be trapped as benzamide in trap 2).

3. Study of the reaction mechanism by quantum theoretical methods

3.1. State of the art and objectives concerning the reaction mechanism of the urea-assisted phosphorylation of alcohols

A priori, two different pathways for the urea-assisted alcohol phosphorylation reaction are possible and have been proposed in the literature. Each of these pathways needs an activation of inorganic orthophosphate (not condensed di- or tri-, not mono- or diamido- and not thiophosphate) to make it amenable to a subsequent alcoholysis. This second alcoholysis step could in principle pass through several different pathways, too, but systematic calculations have shown that the most probable ones are those where both oxygen atoms, those of the apical leaving and incoming groups of a penta-coordinated phosphorous, are still pretty strongly dissociated from the central phosphorous atom being sufficiently stabilised as monomeric (if not free) metaphosphate PO_3^- in all tested trigonal-bipyramidal transition states, especially those having weakly basic leaving groups[13-16]. The question whether the urea-assisted activation of inorganic phosphate (the first step) would be 'dissociative' or 'associative' still hovers in the unknown, although the former, chemically more intuitive option[17-19] has been increasingly challenged and propagated in the past few years by the latter, where urea was assumed to catalytically provoke the direct dehydration of water from a supposed rare tautomer of the inorganic orthophosphate anion, $^{2-}O_3P–OH_2^+$, owing to its hypothetical organo-catalytic association of the phosphorous atom with the oxygen atom of urea[20,21] or formamide[22]. Both respective leaving groups that accompany the alcoholysis (the second step), being inorganic carbamic acid (**CA$_i$**) and urea (**2**), are good (fast) leaving groups as they are only weakly basic (predicted pK_a (**CA$_i$**-H$^+$) = 3.92 to 5.89 in water) or, respectively, not at all (pK_a (**2**-H$^+$) ≈ 0.1 in water).

We explored theoretically, by means of first principles quantum chemical techniques, the reaction mechanism by which inorganic dihydrogenphosphate is activated by urea to be then transformed with the addition of methanol to methyl phosphate. We envisaged two environments; one took into account our prebiotic reaction conditions, but as if there were absolutely no residual water in the milieu (solid at ambient temperature). Hence, one dihydrogenphosphate anion was placed, accompanied by three molecules of urea in an energy-minimalised initial state denoted U3W0. The other environment took into account the fact that we measured, even after drying our starting mixtures through extensive lyophilisation, a residual water content (Supplementary **Table 25**). As a kind of another extreme, we therefore placed one dihydrogenphosphate anion accompanied by three urea and two 'assisting' water molecules to begin in an energy-minimalised initial state denoted U3W2. All stationary points were optimised using Truhlar's Minnesota M06-2X/6-31G(d,p) model. Our initial calculations failed to identify any transition state (single-imaginary vibrational frequency) that would lead to the aforementioned hypothetical rare tautomer $^{2-}O_3P–OH_2^+$ irrespective of the presence or absence of two assisting water molecules (U3W0 or U3W2); this tautomer simply cannot exist. Instead we have found two chemically different reaction pathways, both, in the presence and absence of additional water molecules: Pathway 1_U3W0, Pathway 1_U3W2, Pathway 2_U3W0 and Pathway 2_U3W2, see Supplementary **Fig. 14**-Supplementary **Fig. 15** for the graphical representation of the reaction pathways and Supplementary **Table 4**-Supplementary **Table 7** for the relative Gibbs free energies of the stationary states. In all four pathways we assumed that the methanol molecule did only loosely interact with the reactants at the beginning of the potential energy surface. We checked this with a calculation of the optimised initial state in the presence of methanol and compared the energy with respect to the initial state in the absence of methanol plus the energy of a free methanol molecule. The changes were tiny, which supported our approximation to introduce the methanol at a later stage in the potential energy surface.

To ascertain a convincing strategy that would consist of a good balance between calculation time and accuracy of the results, we first focussed exclusively on the Pathway 1_U3W0 and compared methods and basis sets (**Supplementary Figure 13**). Single-point calculations of the energies of the geometries that were found at the M06-2X/6-31G(d,p) level of theory were calculated using the extended 6-311++G(2d,2p) basis set, in order to allow to take more accurately into account electron

correlation, polarization, and charge delocalisation of the anionic cluster. Next, we evaluated the performance of the M06-2X against the CCSD methods using the diffuse 6-311++G(2d,2p) basis set. The obtained results showed that the energy differences between M06-2X and CCSD were relatively small. Hence, when combined with a sufficiently extensive basis, M06-2X provides an excellent agreement with high-level ab initio CCSD for the relative energies of stable intermediates. This validated our option to use the M06-2X functional to describe the aforementioned four pathways and compare the relative Gibbs free energies of the stationary states (intermediate and transition states) at the M06-2X/6-311++G(2d,2p) level of theory (Fig. 4 and Sections 3.2 to 3.4).

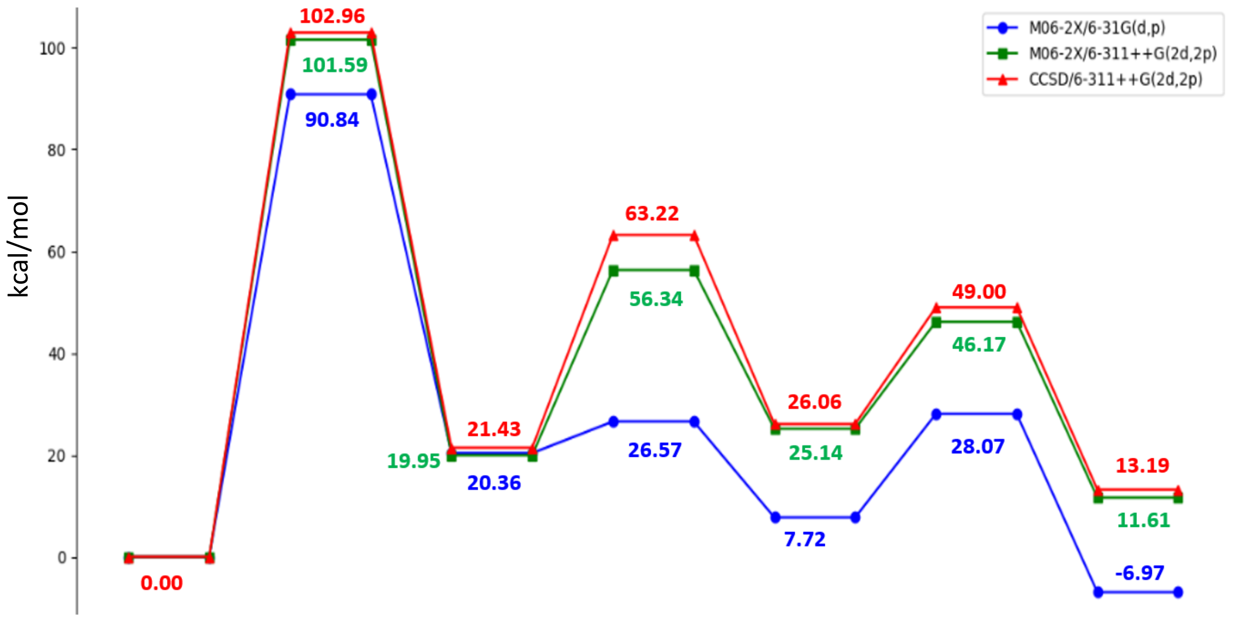

Supplementary Figure 13. Associative phosphate activation pathway P1_U2W3 starting from one inorganic dihydrogenphophate anion and three molecules of urea (U3) in absence of water molecules (W0) calculated at M06-2X/6-31G(d,p) (**blue**), M06-2X/6-311++G(2d,2p)//M06-2X/6-31G(d,p) (**green**) and CCSD/6-311++G(2d,2p)//M06-2X/6-31G(d,p) (**red**) levels of theory; y-axis: relative Gibbs free energies in [kcal/mol]. See Supplementary **Fig. 14** for atomic details.

3.2. Associative (organo-catalytic) versus dissociative (eliminative) phosphate activation

As can be seen in Supplementary **Fig. 14**, the associative pathway consists of the urea-provoked dehydration of phosphate, that is, a pathway where urea acts overall as a catalyst (is not consumed during the course of the reaction). The first transition state, in which one molecule of urea is deprotonated to the isourea anion $H_2N\text{-}C(O^-)\text{=}NH$ to immediately give carbamimidic phosphoric anhydride $HN\text{=}C(NH_2)OPO_2(OH)^-$ and one water molecule, is the key step that determines the reaction kinetics in, both, the U3W0 and U3W2 milieus, see Pathway 1 in Supplementary **Fig. 14** (unlike the ball-and-stick models, the key molecular structures are shown, for more clarity, without assisting urea and water molecules). This water molecule originates from a proton of the aforementioned catalytic urea molecule and a hydroxyl anion HO^- that was eliminated from the dihydrogen phosphate anion $P(OH)_2O_2^-$ transiently creating metaphosphoric acid $PO_2(OH)$, see [first transition state **TS-1**]$^{\#}$. The recalculated Gibbs free energies of the stationary states shown in Supplementary **Fig. 14** are more accurate (solid lines). The presence of two assisting water molecules (added right from the start) lowers the activation energy of the transition state **TS-1** by 35 kcal/mol (from 101 to 66 kcal/mol) and influences less the rest of the potential energy surface. The formation of metaphosphate and the regeneration of urea costs 22 (in U3W2) and 36 kcal/mol activation. After adding a molecule of methanol, 21 and 25 kcal/mol activation barriers are needed for the methanolysis step in U3W0 and, respectively, U3W2. The final state containing all recovered urea molecules along with methyl phosphate and water

has been found to be endergonic with respect to the initial state by $\Delta_r G° \approx +7$ to +12 kcal/mol under U3W0 and U3W2 conditions (rightmost levels compared to leftmost initial Gibbs free energy level).

This associative dehydration pathway seems at a first sight too slow to be competitive with the dissociative phosphate activation pathway, as illustrated in Supplementary **Fig. 15** denoted Pathway 2 (again the key molecular structures are shown without assisting urea and water molecules for greater clarity). In that second pathway, one of three urea molecules first tautomerises in an assisting-urea-mediated fashion to another, less costly zwitterionic isourea tautomer $HN{=}C(O^-){-}NH_3^+$ which is more rapidly achieved than the phosphate-associated isourea anion on Pathway 1. In the U3W0 milieu roughly 46 kcal/mol less activation energy is spent when compared to that of the organo-catalytic Pathway 1. On Pathway 2 **TS-1_U3W0** needs 56 kcal/mol for a direct N-to-N migration of one proton on the same urea molecule. **TS-1_U3W2** needs a mere 32 kcal/mol for this tautomerisation which involves a water molecule that relays the proton from one N atom to the other N of the same urea molecule (compare the leftmost orange with leftmost blue shadowed boxes in Supplementary **Fig. 15**). This zwitterionic isourea tautomer is predisposed to dissociate into ammonia NH_3 and isocyanic acid HNCO, which costs another 14-19 kcal/mol activation in the absence or, respectively, presence of assisting water molecules and sums up to an overall trajectory-determining transition state of roughly 37 kcal/mol in the U3W2 milieu (**TS-2** in Supplementary **Fig. 15**) compared to 60 kcal/mol on the corresponding organo-catalytic pathway.

Ammonia and isocyanic acid are in biased equilibrium with ammonium cyanate, disfavoured in the U3W0 milieu by –10 kcal/mol with respect to $NH_4^+[NCO]^-$ and by about –8 kcal/mol in the U3W2 environment. Interestingly, the difference in pK_a values $\Delta pK_a = 5.54$ resulting from those of isocyanic (3.68)[23] and ammonium (9.24) acids translates to a standard Gibbs free energy difference $\Delta G°_{HNCO+NH3\rightarrow NH4NCO} = -0.44$ kcal/mol at 298 K, which corresponds to an almost 350-thousand-fold excess cyanate over isocyanic acid in water. Thereafter, dihydrogen phosphate adds to isocyanate, at a non-rate-determining 28 kcal/mol activation cost for U3W0 and only 25 kcal/mol for U3W2, to produce inorganic carbamoyl phosphate $H_2NC(O)OP(OH)O_2^-$ (**CP$_i$**) as the activated phosphate intermediate, see the formation of **Int-4** for U3W0 and U3W2 in Supplementary **Fig. 15**. In the U3W0 environment the presence of ammonia and an added molecule of methanol (**Int-5**) is only slightly less stable than the initial state, whereas in the U3W2 milieu the same activated intermediate state **Int-5** (comprising **CP$_i$**) is exergonic by almost –7 kcal/mol when compared to its initial state.

3.3. Thermodynamic preferences and kinetic competition

Of note for all steps after the phosphate activation, first the kinetics. Intriguingly, the methanolysis step of **CP$_i$** forward from **Int-5** on Pathway 2 (Supplementary **Fig. 15**) needs about 12 kcal/mol higher activation than that of the carbamimidic phosphoric anhydride generated on Pathway 1 (the attack of methanol on metaphosphate forward from **Int-2**, Supplementary **Fig. 14**), viz. 33 kcal/mol (U3W0) and 37 kcal/mol (U3W2) on Pathway 2 versus 21 kcal/mol (U3W0) and 25 kcal/ mol (U3W2) on Pathway 1. In other words, alcoholysis itself is kinetically favoured on the associative phosphate dehydration pathway compared to the overall kinetically more favourable dissociative phosphate activation pathway. This enhances the chance for the reaction passing through two competing mechanisms should there be a possibility, which has not been accounted for in our initial state, of accelerating the deprotonation of one urea molecule, viz. an isourea anion-hydrogenphosphate complex (corresponding to **TS-1** in Supplementary **Fig. 14**), to give more rapidly the carbamimidic phosphoric anhydride than in this calculation (**Int-1** in Supplementary **Fig. 14**).

Second, the thermodynamics: like the diluted aqueous hydrolysis equilibrium state of phosphoric monoesters[24], the most stable 'final' product state in neat conditions is disfavoured in the organo-catalytic phosphate dehydration, Pathway 1. Methyl phosphate, water and urea are endergonic with respect to their corresponding initial state by about +11.6 (U3W0) and +6.7 kcal/mol (U3W2) on Pathway 1 (Supplementary **Fig. 14**). This is to be taken with a margin, since the effect of adding one molecule of methanol in the middle of Pathway 1 (tested to have a very small effect on the initial state) may not be completely nil. On Pathway 2, however, the molecular cluster containing methyl

phosphoric acid and inorganic carbamate **CA$_i$** is favoured by –6.3 and –11.7 kcal/mol (to be taken with a margin, for the same reason). Hence, using our initial states (but see the note above) the dissociative phosphate activation pathway is not only kinetically favoured over the associative urea-catalysed phosphate dehydration pathway. The urea-assisted methylation of inorganic phosphate by methanol is also thermodynamically more favoured when urea degrades (Pathway 2) than when it is regenerated (Pathway 1).

Interestingly, and irrespective of the content of residual water molecules in the initial state (U3W0 or U3W2), the most stable product state on Pathway 2 does not (yet) contain the methyl phosphate anion $CH_3OP(OH)O_2^-$ (unlike the product state on Pathway 1) but methyl phosphoric acid $CH_3OP(OH)_2O$, inorganic carbamate H_2NCOO^- (**CA$_i$**) and ammonia NH_3 (**Int-7** in Supplementary **Fig. 15**). The production of carbon dioxide, the methyl phosphate anion and a second molecule of ammonia leads through zwitterionic carbamic acid $H_3^+NCOO^-$ (**CA$_i$** in **Int-8**) and is in both milieus a 16-17 kcal/mol-uphill process, but only in a closed equilibrium system. In a naturally open system both CO_2 and NH_3 will eventually evaporate and, in doing so, drive the reaction to completeness by means of the Le Châtelier Principle.

3.4. Conclusion on the mechanism

Our calculations have shown that urea's interactions with water or methanol are relatively weak when compared to those with inorganic dihydrogenphosphate (**P$_i$**). The dissociative Pathway 2 clarifies how **P$_i$** accelerates as an "acid-base catalyst" the degradation of urea to carbon dioxide and ammonia, viz. through catalysing the formation of a zwitterionic tautomer of urea predisposed to break one carbon-nitrogen bond. However, urea is known to degrade through hydrolysis and in the absence of inorganic phosphate[17], albeit much more slowly, to the same inorganic products. So, with residual water present in the milieu, both urea-assisted phosphorylation mechanisms (Pathways 1 and 2) might compete with one another mostly at the beginning of the reaction and on a kinetic level. In addition, both mechanisms could be extended to the synthesis of phosphoric acid diesters (acyclic or cyclic, both found experimentally) in replacing **P$_i$** with an organic phosphoric monoester $ROPO_2(OH)^-$. In both mechanisms, the addition of the phosphoric acid monoester instead of an alcohol should result in the synthesis of organic pyro-(di)phosphates (phosphoric anhydrides sometimes found experimentally).

We haven't systematically tested other urea- and water-containing initial states (UxWy). For example, we did not test initial conditions in which the number of **P$_i$** molecules would equal that of urea molecules (U1), albeit most experiments were carried out at an equimolar ratio. Neither did we model any environment made of molecules in an explicit solid state that would constrain the mobility, thus, further enhance the kinetic barriers of the reactive complexes, nor did we calculate the potential energy surface beginning with a complex where one of the urea molecules was already tautomerised to charge-neutral isourea right from the start. This could be the case when cyanamide was hydrolysed to first generate this rare isourea tautomer, before it would spontaneously tautomerise to urea[25], or else, associate its oxygen atom with the phosphorous atom of a nearby **P$_i$**. We therefore cannot be entirely certain that the deprotonation of urea to the isourea anion attacking the phosphate (similar to **TS-1** on Pathway 1) would not be able to more fiercely compete with the tautomerisation of urea to zwitterionic urea and its later elimination of ammonia to give an intermediate state containing isocyanate (**Int-1** and **Int-3** on Pathway 2); the more so, as the alcoholysis on Pathway 1 seems kinetically somewhat favoured over that on Pathway 2. We asked ourselves, could the direct urea-assisted dehydration of two **P$_i$** ions to give **PP$_i$** (sometimes found experimentally) be totally excluded, and is the passage through several phosphorylation mechanisms at a time possible? Only experiments, especially those using stable isotopes, could answer this (see Section **6**).

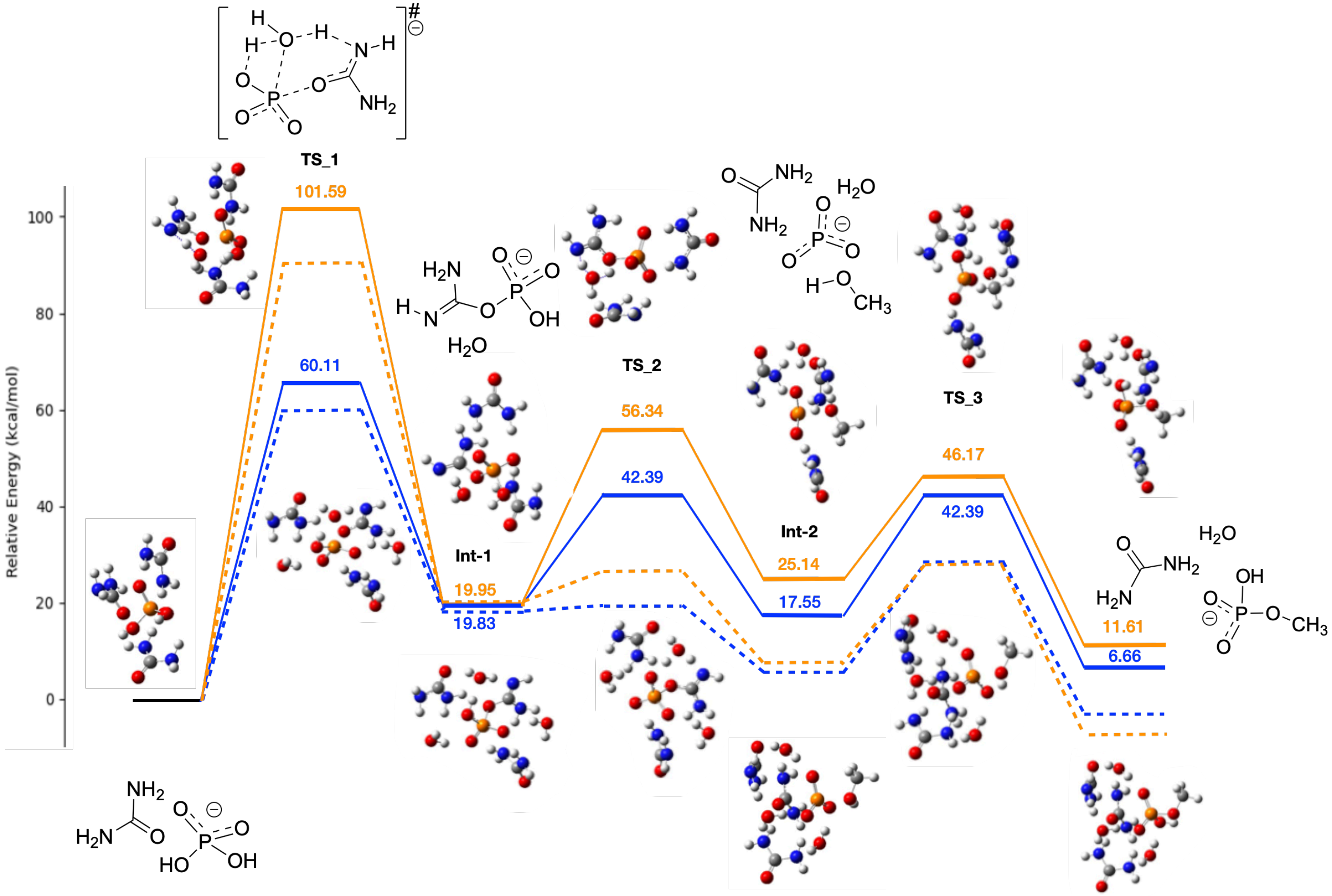

Supplementary Fig. 14. Associative phosphate dehydration pathway (denominated **Pathway 1**) starting from one inorganic dihydrogenphosphate anion and three molecules of urea (U3) in the absence, **Pathway 1_U3W0** (orange), and presence **Pathway 1_U3W2** (blue)of two 'assisting' water molecules (W0, W2). The atom coordinates were energy-minimised at the M06-2X/6-31G(d,p) level of theory (list of coordinates: cf. Supplementary Dataset). The relative Gibbs free energies [kcal/mol] were calculated at, both, the M06-2X/6-31G(d,p)//M06-2X/6-31G(d,p) (dotted lines, relative Gibbs free energy values in Supplementary **Table 4**-Supplementary **Table 5**) and the M06-2X/6-311G++(2d,2p)//M06-2X/6-31G(d,p) levels (continuous lines, values shown). Methanol was added to **Int-2** (its energy was zeroed). The transition and intermediate state ball-and-stick models are shown above (Pathway 1_U3W0) and below (Pathway 1_U3W2). Both end with the product complex containing methyl phosphate, three recovered urea molecules and at least one (cleaved) water molecule. The chemical structures are depicted, for more clarity, without 'assisting' urea and water molecules.

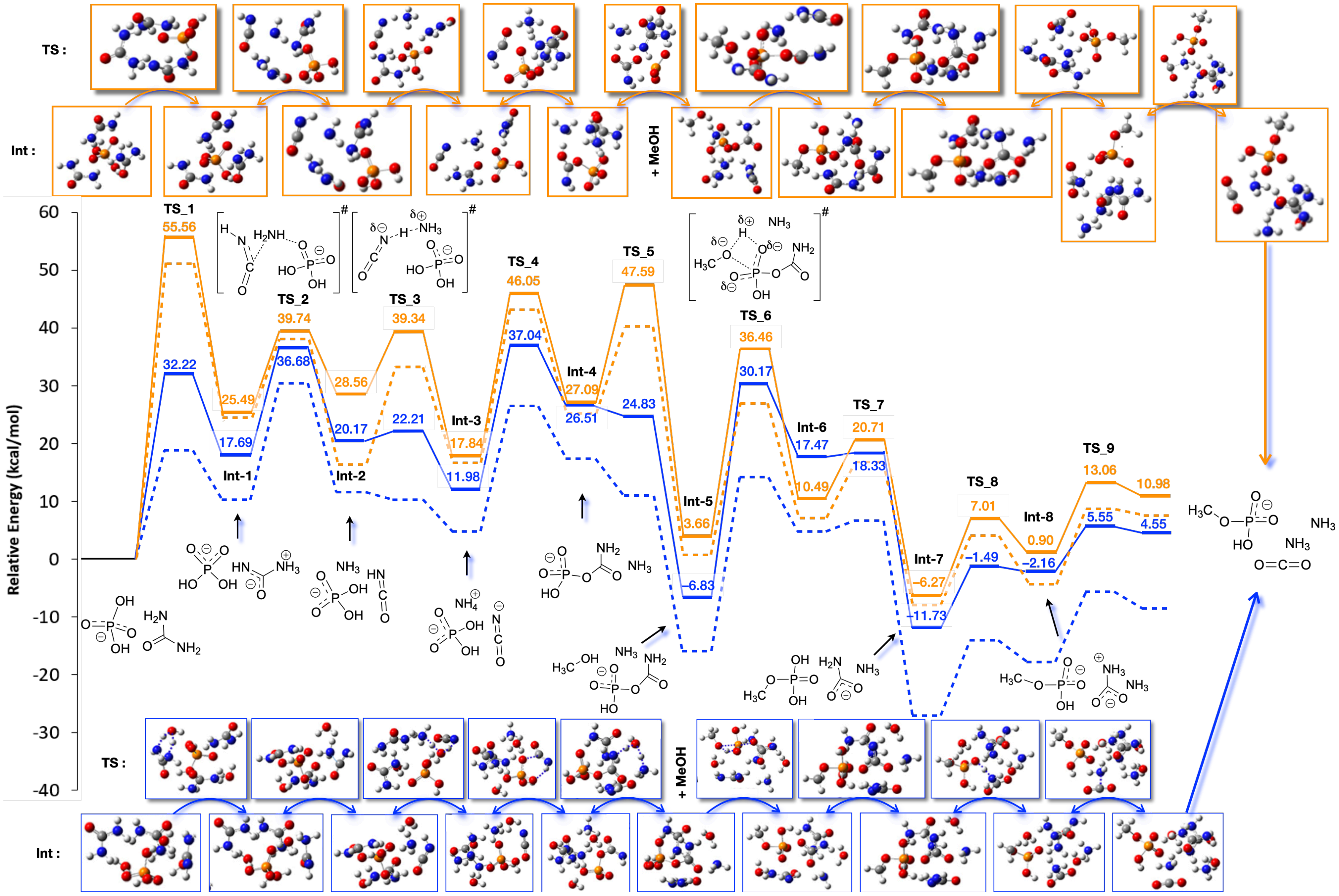

Supplementary Fig. 15. Dissociative phosphate activation pathway in urea without and with residual water: 8 intermediate states **Int-1** to **Int-8** and 9 transition states **TS-1** to **TS-9** on **Pathway 2_U3W0** (**orange**) and **Pathway 2_U3W2** (**blue**). The atom coordinates were energy-minimised at the M06-2X/6-31G(d,p) level of theory (models in boxes, coordinates see Supplementary Dataset). The relative Gibbs free energies [kcal/mol] were calculated, both, at the M06-2X/6-31G(d,p)//M06-2X/6-31G(d,p) (dotted lines, values see Supplementary **Table 6**-Supplementary **Table 7**) and the M06-2X/6-311G++(2d,2p)//M06-2X/6-31G(d,p) levels (continuous lines, values shown). Methanol was added to **Int-5**. Shadowed boxes: transistion states, other boxes: intermediates states. **TS-2** on the blue pathway is the trajectory-determining transition state.

Supplementary Table 4. Effect of basis set on the relative Gibbs free energies given in kcal/mol of the **P1-U3W0** (associative phosphate dehydration, Pathway 1, only urea, no water). The calculation was carried out at the M06-2X/6-31G(d,p) and M06-2X/6-311++G(2d,2p) levels of theory.

	**P1-U3W0**		
	**M06-2X/ 6-31G(d,p)**	**M06-2X/ 6-311++G(2d,2p)**	**Δ[6-31G(d,p) – 6-311++G(2d,2p)]**
**1a**	0.00	0.00	0.00
**TS_1a-2a**	90.84	101.59	-10.75
**2a**	20.36	19.95	0.41
**TS_2a-3a**	26.57	56.34	-29.77
**3a**	7.72	25.14	-17.42
**TS_3a-4a**	28.07	46.17	-18.10
**4a**	-6.97	11.61	-18.58

Supplementary Table 5. Effect of basis set on the relative Gibbs free energies given in kcal/mol of the **P1-U3W2** (associative phosphate dehydration, Pathway 1, urea and water). The calculation was carried out at the M06-2X/6-31G(d,p) and M06-2X/6-311++G(2d,2p) levels of theory.

	**P1-U3W2**		
	**M06-2X/ 6-31G(d,p)**	**M06-2X/ 6-311++G(2d,2p)**	**Δ [6-31G(d,p) – 6-311++G(2d,2p)]**
**1a**	0.00	0.00	0.00
**TS_1a-2a**	60.11	65.84	-5.73
**2a**	18.66	19.83	-1.17
**TS_2a-3a**	19.71	42.39	-22.68
**3a**	5.86	17.55	-11.69
**TS_3a-4a**	28.79	42.39	-13.60
**4a**	-2.92	6.66	-9.58

Supplementary Table 6. Effect of basis set on the relative Gibbs free energies given in kcal/mol of the **P2-U3W0** (dissociative phosphate activation, Pathway 2, only urea, no water). The calculation was carried out at the M06-2X/6-31G(d,p) and M06-2X/6-311++G(2d,2p) levels of theory.

	**P2-U3W0**		
	**M06-2X/ 6-31G(d,p)**	**M06-2X/ 6-311++G(2d,2p)**	**Δ [6-31G(d,p) – 6-311++G(2d,2p)]**
**1b**	0	0.00	0.00
**TS_1b-2b**	51.36	55.56	-4.20
**2b**	24.66	25.49	-0.83
**TS_2b-3b**	38.11	39.74	-1.63
**3b**	16.45	28.56	-12.11
**TS_3b-4b**	33.22	39.34	-6.12

**4b**	16.6	17.84	-1.24
**TS_4b-5b**	43.3	46.05	-2.75
**5b**	25.21	27.09	-1.88
**TS_5b-6b**	40.13	47.59	-7.46
**6b**	0.64	3.66	-3.02
**TS_6b-7b**	26.9	36.46	-9.56
**7b**	7.18	10.49	-3.31
**TS_7b-8b**	16.74	20.71	-3.97
**8b**	-10.05	-6.24	-3.81
**TS_8b-9b**	4.01	7.01	-3.00
**9b**	-4.41	0.90	-5.31
**TS_9b-10b**	8.67	13.06	-4.39
**10b**	7.59	10.98	-3.39

Supplementary Table 7. Effect of basis set on the relative Gibbs free energies given in kcal/mol of the **P2-U3W2** (dissociative phosphate activation, Pathway 2, urea and water). The calculation was carried out at the M06-2X/6-31G(d,p) and M06-2X/6-311++G(2d,2p) levels of theory.

	**P2-U3W2**		
	**M06-2X/ 6-31G(d,p)**	**M06-2X/ 6-311++G(2d,2p)**	**Δ [6-31G(d,p) – 6-311++G(2d,2p)]**
**1b**	0	0.00	0.00
**TS_1b-2b**	18.8	32.22	-13.42
**2b**	10.15	17.69	-7.54
**TS_2b-3b**	30.39	36.68	-6.29
**3b**	11.46	20.17	-8.71
**TS_3b-4b**	10.19	22.21	-12.02
**4b**	4.71	11.98	-7.27
**TS_4b-5b**	26.38	37.04	-10.66
**5b**	17.32	26.51	-9.19
**TS_5b-6b**	10.91	24.83	-13.92
**6b**	-16.04	-6.83	-9.21
**TS_6b-7b**	14.18	30.17	-15.99
**7b**	4.71	17.47	-12.76
**TS_7b-8b**	6.64	18.33	-11.69
**8b**	-27.19	-11.73	-15.46
**TS_8b-9b**	-14.06	-1.49	-12.57
**9b**	-17.9	-2.16	-15.74
**TS_9b-10b**	-5.72	5.55	-11.27
**10b**	-8.58	4.55	-13.13

4. Identification of the reaction products by NMR spectroscopic and mass spectrometric methods

Starting from the principal reaction of this work, glycerol (**5**):urea (**2a**):NaH_2PO_4 (**P**$_i$), we describe here the diversity and nature of the phosphorylated products of this reaction. In order to understand the main processes going on during the reaction, and having in mind the limited natural availability of **P**$_i$, we chose the molar ratio of the starting molecules **5**:**2a**:**P**$_i$ as 1:1:1. After heating the neat compounds for 120 hours at 115 °C, we obtained a complex mixture of organic products. It was analysed by NMR spectroscopic and MS methods. For a deeper understanding of the product structures and later illuminating the reaction mechanism, we used labelled [$^{15}N_2$]urea (**2b**), [^{13}C]urea (**2c**) and $NaH_2P[^{18}O_4]$ (**[$^{18}O_4$]P**$_i$) and tracked the fate of the enriched stable ^{15}N, ^{13}C and ^{18}O isotopes in some of the products.

4.1. Urea-assisted glycerol (5) phosphorylation

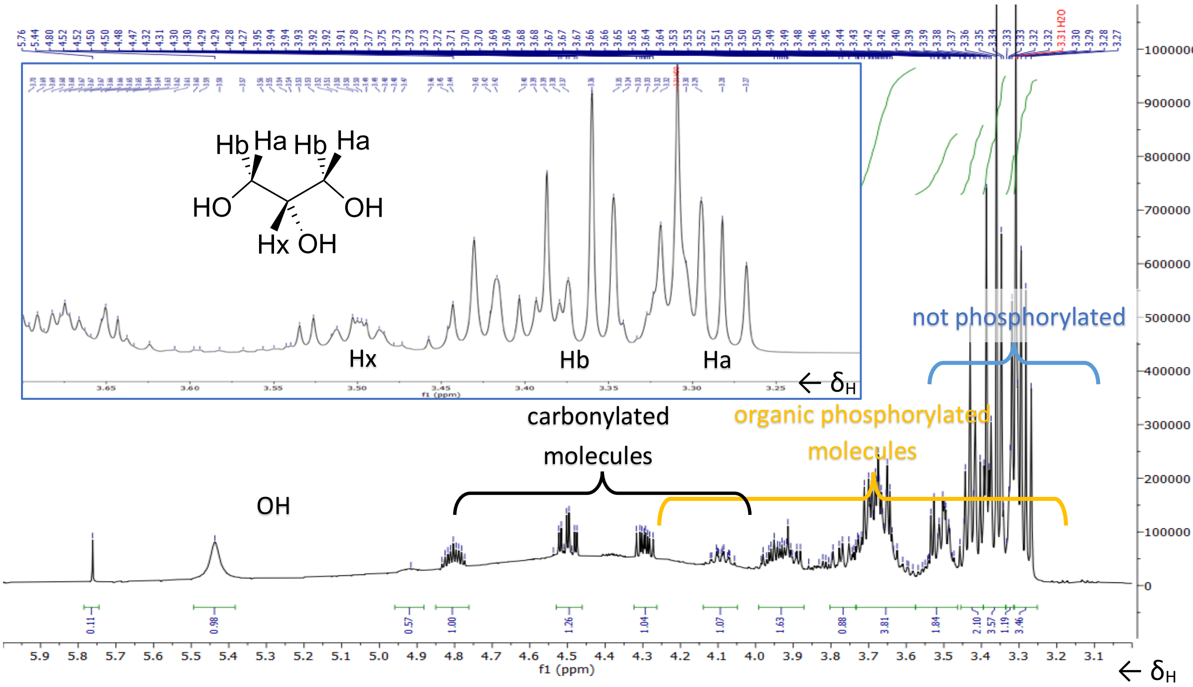

Supplementary Fig. 16. ^{1}H NMR spectrum (500 MHz) of the DMSO-*d*6 extract of a mixture of glycerol (**5**), natural isotope abundance-urea (**2a**) and NaH_2PO_4 (**P**$_i$) (1:1:1, 0.5 mmol each) after heating it neat for 120 h at 115 °C. **Framed insert**: zoom of the mainly not phosphorylated spectral region δ_H = 3.20 - 3.60 ppm shows more than the ABX system (dd_{Ha}, dd_{Hb}, tt_{Hx}) of the unreacted starting glycerol (**5**) only.

According to 2D spectral analyses (Supplementary **Fig. 18**-Supplementary **Fig. 20**), the ^{1}H resonances at δ_H = 3.25-3.55 ppm show protons from the unreacted glycerol (*dd* δ_{Ha}, *dd* δ_{Hb}, *tt* δ_{Hx}) as well as other not phosphorylated glycerol derivatives. These not phosphorylated, charge-neutral side-products cannot be detected in mass spectra of mixtures that contain many different ionic phosphate esters (cf. Supplementary **Fig. 27** and Supplementary **Fig. 28**). δ_H = 3.30-4.30 ppm are glyceryl protons from phosphorylated organic products (Supplementary **Fig. 20A**) and δ_H = 4.30-4.95 ppm from organic carbonylated products (carbamate or cyclic carbonate) and the intense AB part at δ_H = 3.51 and 3.66 ppm (2 *dd*) from the main carbonylated product (Ha', Hb' cf. Supplementary **Fig. 17**).

In this ^{1}H NMR spectrum the baseline, between δ_H = 4.10 and 5.20 ppm, is elevated due to the NH_2 groups of urea and carbamates. The broad signal at δ_H = 5.48 might be from OH groups. Using the MNova tool *Global Spectral Deconvolution* we were able to extract the broad signal of the NH_2 groups of urea and glyceryl carbamates to generate spectra with a corrected baseline, the C-H signal areas of which could be more reliably integrated (Supplementary **Fig. 17**). Comparing the (upper) ^{1}H NMR

spectrum with the one obtained in experiments using [^{13}C]urea we note in the latter a doubling of multiplicity (splitting) of the signals in the range of organic carbonylated products (Supplementary **Fig. 17**, lower spectrum). The ^{1}H NMR spectrum after using [$^{15}N_2$]urea (not shown) shows the same multiplicities in this region as those of the unlabelled mixture. This confirms the presence of one ^{13}C carbonyl atom next to these protons.

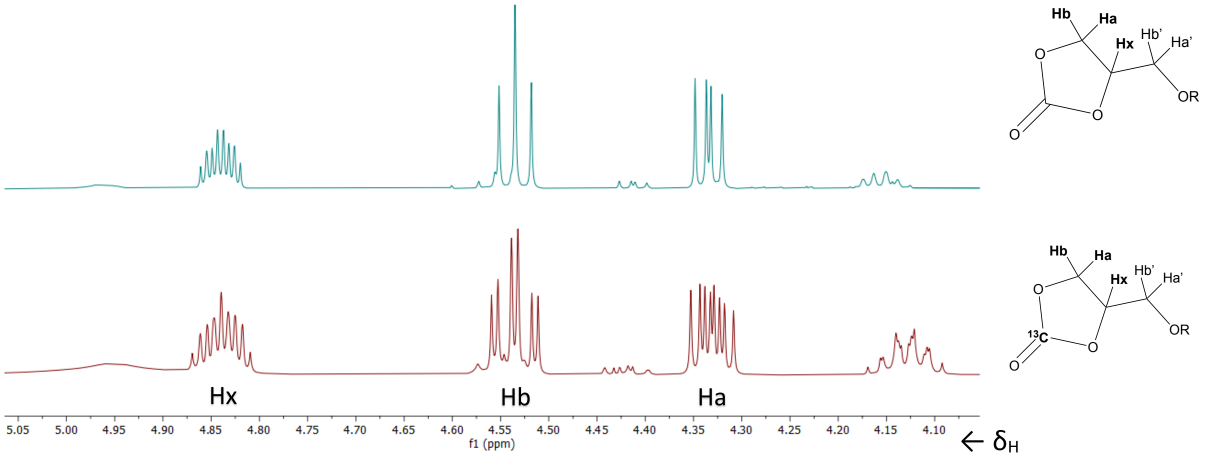

Supplementary Fig. 17. Zoom on baseline-flattened ^{1}H NMR spectra (*Global Spectra Deconvolution*, MNova) of DMSO-*d6* extracts of two 0.5 mmol scale equimolar 'dry' (neat) mixtures of glycerol (**5**), natural isotope abundance-urea (**2a**) or [^{13}C]urea (**2c**) and NaH_2PO_4 (**P**$_i$) after heating at 115 °C for 120 hours. **Upper spectrum** (**5**:**2a**:**P**$_i$ 1:1:1, Supplementary **Fig. 16**, 500 MHz): δ_H = 4.83 (*ddd*, J = 8.8, 6.0, 3.1 Hz, 1H, Hx), 4.53 (*t*, J = 8.4 Hz, 1Hb), 4.33 (*dd*, J = 8.1, 6.1 Hz, 1H, Ha), 4.17-4.07 (*m*, 1H, Ha). **Lower spectrum** (**5**:**2c**:**P**$_i$ 1:1:1, 400 MHz): δ_H = 4.83 (apparent *tt* (*dddd*), J = 9.1, 3.1 Hz, 1H, Hx), 4.53 (*td*, J = 8.3, 2.6 Hz, 1H, Hb), 4.33 (*ddd*, J = 8.1, 5.8, 3.8 Hz, 1H, Ha). R = H or PO_2OH^-.

From ^{1}H-^{1}H COSY (Supplementary **Fig. 18**) we conclude that the main signals at δ_H = 4.83, 4.53, 4.33, 3.51 and 3.66 ppm belong to glyceryl-1,2-cyclic carbonate where the resonances for Hx, Hb and Ha take 10.7 % of the total area integral in the baseline-flattened ^{1}H NMR spectrum, which means that this compound (Hx, Hb, Ha, Hb', Ha') is present in almost 18 %.

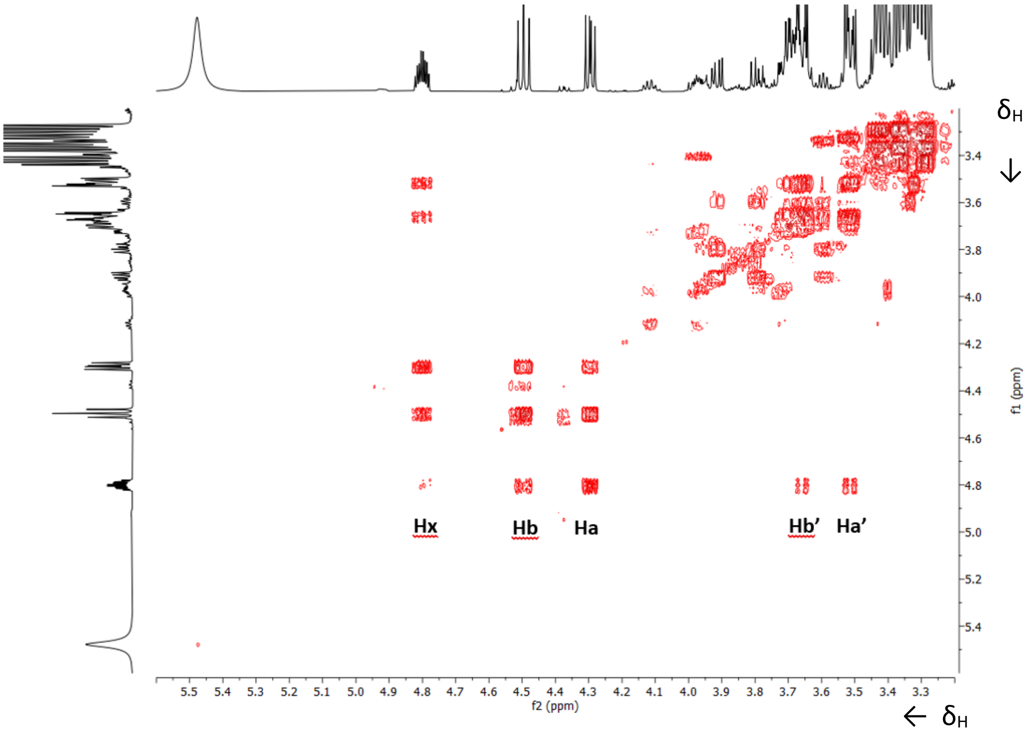

Supplementary Fig. 18. ^{1}H-^{1}H COSY (500 MHz) of the DMSO-*d6* extract of a mixture of glycerol (**5**), natural isotope abundance-urea (**2a**) and NaH_2PO_4 (**P**$_i$) (1:1:1, 0.5 mmol each) after heating it neat for 120 h of at 115 °C.

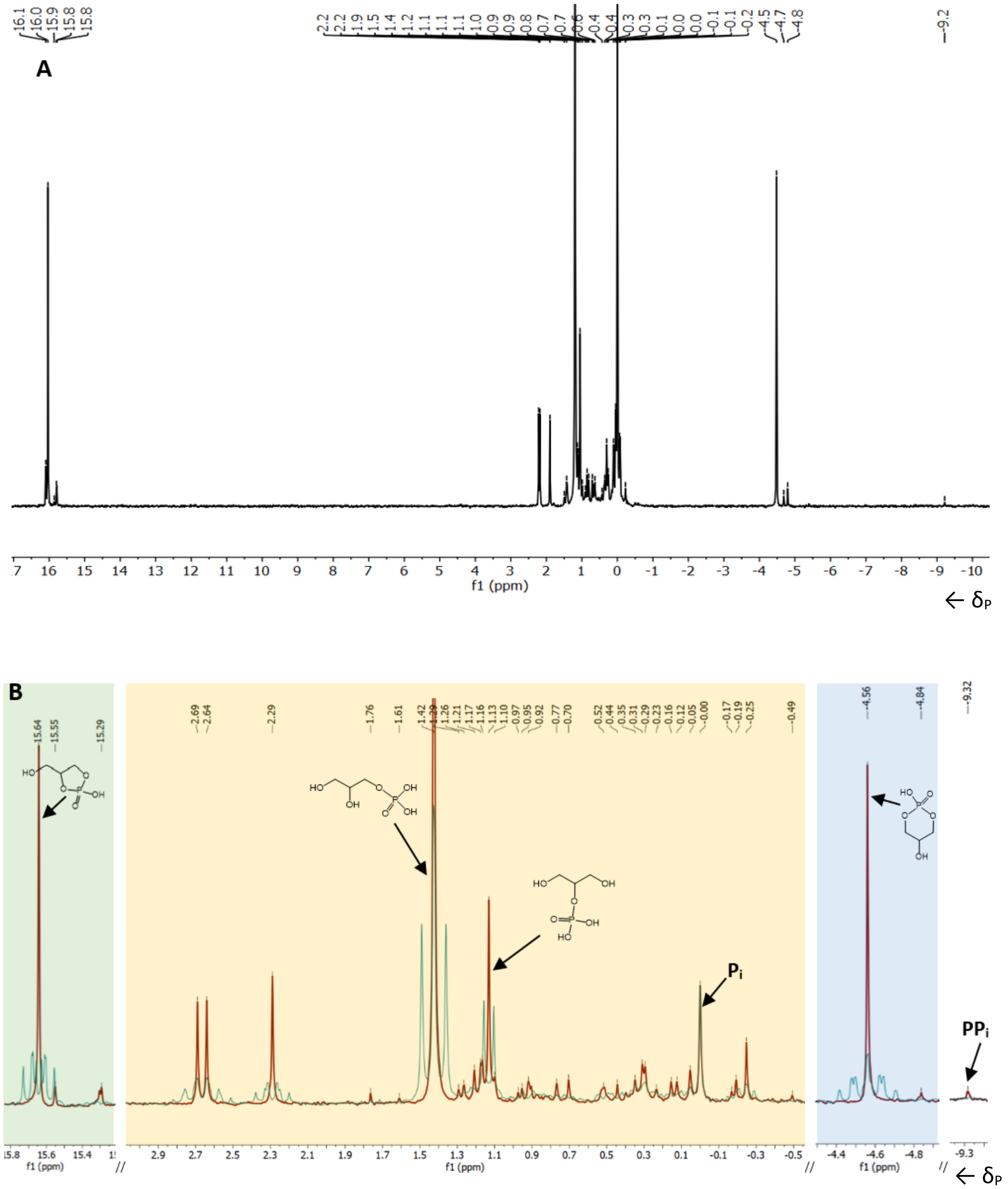

Supplementary Fig. 19. **A** – $^{31}P\{^{1}H\}$ NMR (202.5 MHz, DMSO-*d*6) of the extract of a neat 0.5 mmol scale equimolar mixture of glycerol (**5**), natural isotope abundance-urea (**2a**) and NaH_2PO_4 (**P**$_i$) (1:1:1) after 120 h of heating at 115 °C. **B** – excerpts of **A** (**brown**) superimposed with ^{1}H-coupled ^{31}P NMR spectrum (**turquoise**) of the same mixture. Pale green, ochre and pale blue zones colour coded as in Supplementary **Fig. 20A**.

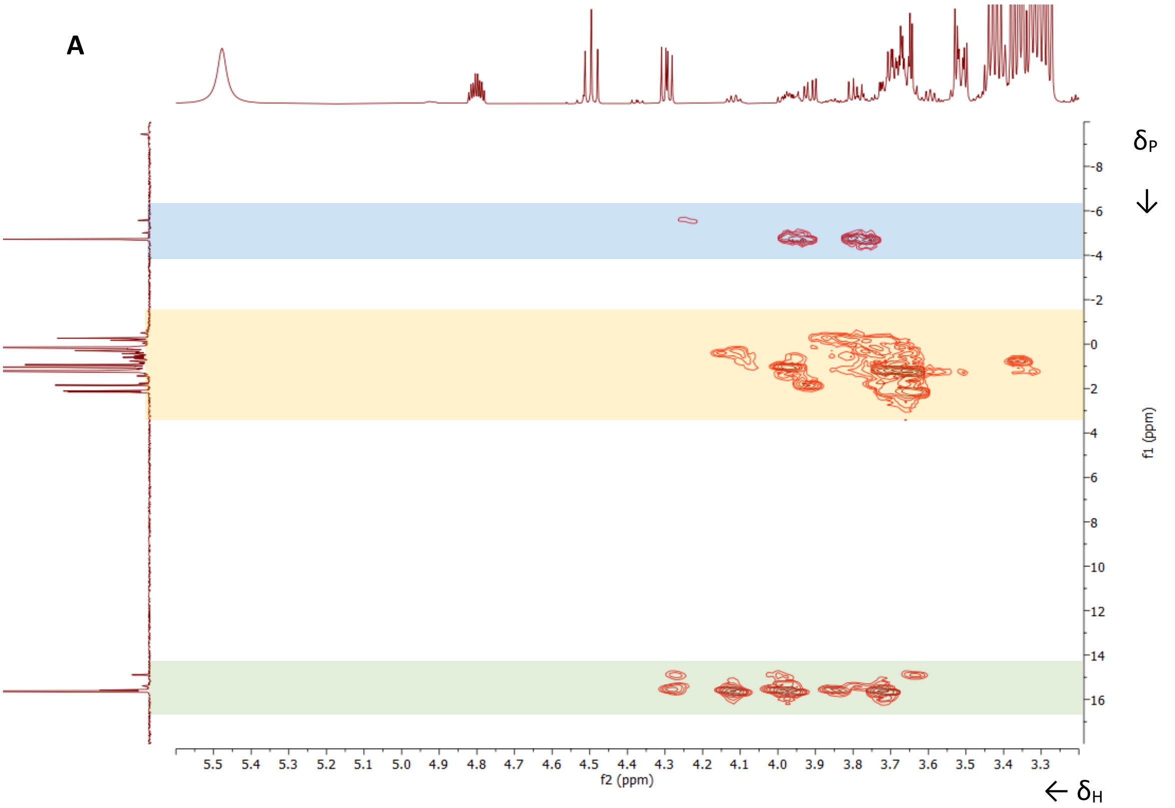

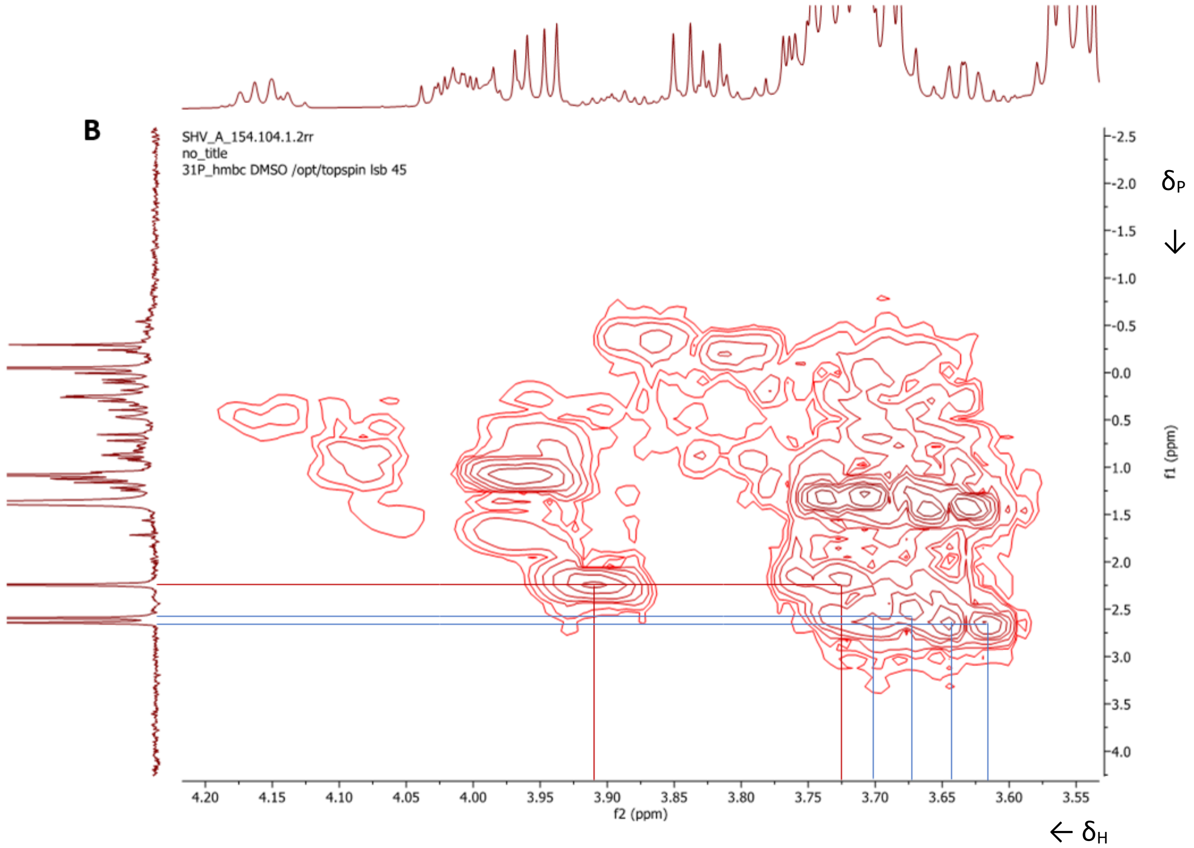

Supplementary Fig. 20. ^{1}H-^{31}P HMBC spectrum (500 MHz for ^{1}H: horizontal axis, baseline-flattened, 202.5 MHz for $^{31}P\{^{1}H\}$: vertical axis) of the DMSO-*d6* extract of a neat 0.5 mmol scale mixture of glycerol (**5**), natural isotope abundance-urea (**2a**) and NaH_2PO_4 (**P**$_i$) (1:1:1 molar ratios) after 120 h of heating at 115 °C. Correlation of signals ^{1}H and ^{31}P seen as cross-spots representing organic products in the reaction mixture. **A** – All ^{31}P signals. **B** – Acyclic organic phosphate esters.

We studied in detail the multiplicity of the ^{1}H-coupled ^{31}P NMR resonances to confirm the immediate through-bond neighborships of the ^{31}P atoms. Proton-coupled triplets indicate acyclic primary phosphate esters $RCH_2OP(O)_3^{-2}$, doublets indicate secondary phosphate esters $R_2CHOP(O)_3^{-2}$ (Supplementary **Fig. 21C**) and singlets originate from inorganic ortho- (**P$_i$**) and pyrophosphates (**PP$_i$**). Cyclic phosphate esters produce ^{1}H-^{31}P couplings to give either a *ddd* ^{31}P multiplicity for five-membered rings or a *tt(d)* ^{31}P multiplicity for symmetrical 6-membered rings provided that the long-range doublet can be resolved (Supplementary **Fig. 21A** and **B**).

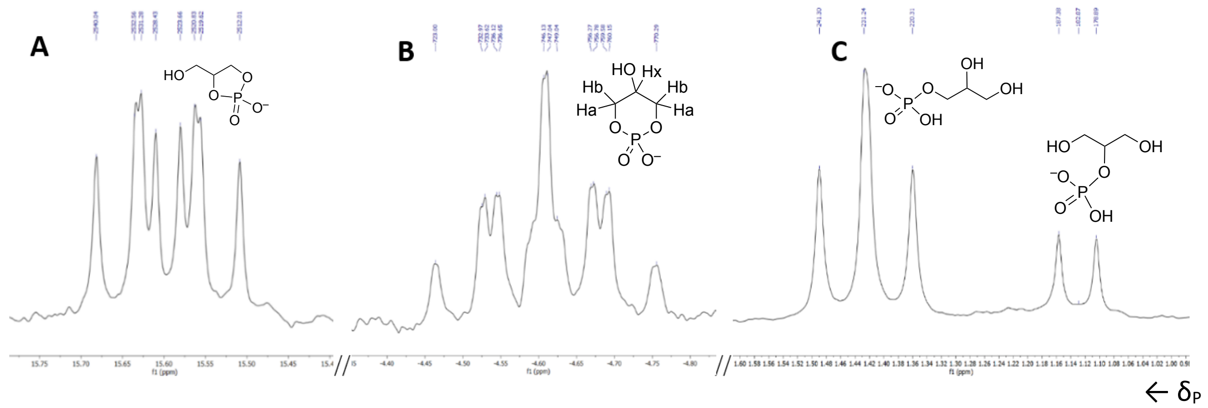

Supplementary Fig. 21. ^{31}P NMR spectra (^{1}H-coupled, 202.5 MHz, DMSO-*d*6), selected signals from Supplementary **Fig. 19** of the extract of a 0.5 mmol scale mixture of glycerol (**5**), natural isotope abundance-urea (**2a**) and NaH_2PO_4 (**P$_i$**) (1:1:1) after 120 h of heating at 115 °C. **A** – zoom of the 5-membered ring cyclic phosphate; δ_P = 15.64 ppm (*ddd,* $^{3}J_{H,P}$ = 11.6 Hz, $^{3}J_{H,P}$ = 8.7 Hz, $^{3}J_{H,P}$ = 7.6 Hz). **B** – zoom on 6-membered ring cyclic phosphate; δ_P = –4.56 ppm (*ttd,* $^{3}J_{Ha,P}$ = 13.4 Hz, $^{3}J_{Hb,P}$ = 10.2 Hz, $^{4}J_{Hx,P}$ = 0.7 Hz). **C** – zoom on acyclic organic phosphates (1.60-0.9 ppm); δ_P (**GlO1P**) = 1.43 (*t*, $^{3}J_{H,P}$ = 10.6 Hz), δ_P (**GlO2P**) =1.13 (*d*, $^{3}J_{H,P}$ = 8.5 Hz).

Following these ^{1}H-coupled ^{31}P multiplicities, the observed organic and inorganic products can be divided in several groups: the signal at δ_{Pi} = 0.00 ppm is due to inorganic ortho-phosphate PO_4^{-3} (**P$_i$**) as a residual part of the initial phosphorus source that remained unreacted and δ_{PPi} = – 9.32 ppm is inorganic pyrophosphate $P_2O_7^{-4}$ (**PP$_i$**) as a by-product of the reaction. Non-terminal polyphosphate (**P$_{ni}$**) signals appear as ^{1}H-decoupled ^{31}P{^{1}H} triplets at –18 to –30 ppm, the corresponding terminal signals as ^{31}P{^{1}H} doublets at –8 to –12 ppm, both are rarely observed, mostly in nucleoside phosphorylations in water. The exact chemical shifts δ_{Pi}, δ_{PPi} and δ_{Pni} depend on the proton concentration of the solution, the more basic the more deshielded: δ_{Pi} mono-basic (around –0.4 ppm) < di-basic < tri-basic (up to +3.4 ppm). Organic products such as cyclic organic 5-membered ring phosphates (**5cGlP**) $\delta_{5\text{-mr}}$ = 12-20 ppm (pale green zone), 6-membered rings (**6cGlP**) $\delta_{6\text{-mr}}$ = –4 to –7 ppm (pale blue zone) and acyclic primary and secondary monophosphates (**GlO1P** and, respectively, **GlO2P**) usually resonate at $\delta_{acyclic}$ = –1 to +2 ppm (ochre zone). Conferring to ^{31}P{^{1}H} signals of mixtures containing dinucleoside phosphodiesters (cf. yellow zones in the ^{31}P{^{1}H} NMR spectra shown **Section 7.5**), those of diglyceryl phosphodiesters (**[Gl]$_2$P**) could be tentatively expected to resonate at higher field of this range; however, neither ^{1}H-coupled ^{31}P signals (turquoise line spectrum in Supplementary **Fig. 19**) nor ^{31}P DOSY spectra (not shown) were conclusive at this stage.

4.1.1. Two persistent organic phosphorylated and carbonylated minor reaction products

In all DMSO-*d*6 solutions containing urea-assisted phosphorylation mixtures of glycerol we have unfailingly observed other ^{31}P resonances, never observed as such in D_2O nor in DMSO-*d*6 extracts of phosphorylations of glycerol in the absence of urea, except with hot **cTMP** (Section 7.4.2). These resonances are shifted downfield with respect to all other acyclic product ^{31}P signals. They belong to two phosphorylated compounds, appear in changing concentrations and precise resonance frequencies with reaction time passing and show complex multiplicities in their proton-coupled ^{31}P signals in the region between δ_P = 1.9 and 2.9 ppm (Supplementary **Fig. 19B**). One such glyceryl phosphate produces in proton-decoupled ^{31}P{^{1}H} NMR spectra two quite strongly deshielded peaks (δ_P = 2.35-2.45 ppm) that seem to be a ^{31}P-^{31}P doublet but are in fact two singlets. We have discovered in different experiments (different batches, different reaction time periods) that not only their chemical shift varied with respect to that of **P$_i$**, which was expected from slight differences in pH (proton abundance). The difference in their relative chemical shifts $\Delta\delta_P$ varied as well, see Supplementary **Fig. 22A**, Supplementary **Fig. 45**, Supplementary **Fig. 46**, and Supplementary **Table 13**. However, the ratio of their proton-decoupled ^{31}P{^{1}H} peak areas was exactly 1:1 in all cases. In proton-coupled ^{31}P NMR spectra they split into two quintets (2 x *quint*) with the same coupling constant each or, when the $\Delta\delta_P$ happened to equal their coupling constant, they appear as an apparent sextet (Supplementary **Fig. 22B** and **C**). The other deshielded phosphorylated glyceryl derivative resonates as an ordinary ^{31}P{^{1}H} singlet, always slightly more shielded than the double-resonance (δ_P = 2.0-2.2 ppm), and is in all fully coupled ^{31}P NMR spectrum a doublet of triplets (*dt*) or it coalesces into an apparent quartet (Supplementary **Fig. 22B** and **D**). Noteworthy is that the fully coupled ^{31}P resonance patterns for both compounds differ in their appearance depending on whether we used for the phosphorylation experiment natural isotope abundance-urea (**2a**), [$^{15}N_2$] (**2b**) or [^{13}C]urea (**2c**). The most marked differences were observed when [^{13}C]urea (**2c**) was added: at lower field strength best visible in the more upfield signal (being a ^{1}H-decoupled singlet, lines **1**-**2** in Supplementary **Fig. 22B**) and at slightly higher field strength best visible in the more downfield signal (resonating at two frequencies in the ^{1}H-decoupled spectrum, lines **1** and **3**, Supplementary **Fig. 22B**). Minute barely significant differences are also observable in the both signals when [$^{15}N_2$]urea (**2b**) was used (lines **1**, **4**, **5**, Supplementary **Fig. 22B**). We ascribed those *a priori* to the ^{15}N isotope effect either of carbamoyl groups (if present) or ammonium ions, thus, the volatile counter cations of the organic phosphates, the other one being Na^+.

This meant that these minor compounds contained, apart from a phosphate group, also a carbonyl function in both molecules. A chemical explanation could be the presence of carbamates or cyclic carbonates in the acyclic glyceryl phosphate molecules. To confirm the theory, we conducted an experiment on commercial glyceryl phosphate (**5a**) with natural isotope abundance urea (**2a**) in the absence and presence of inorganic phosphate (Supplementary **Fig. 23**).

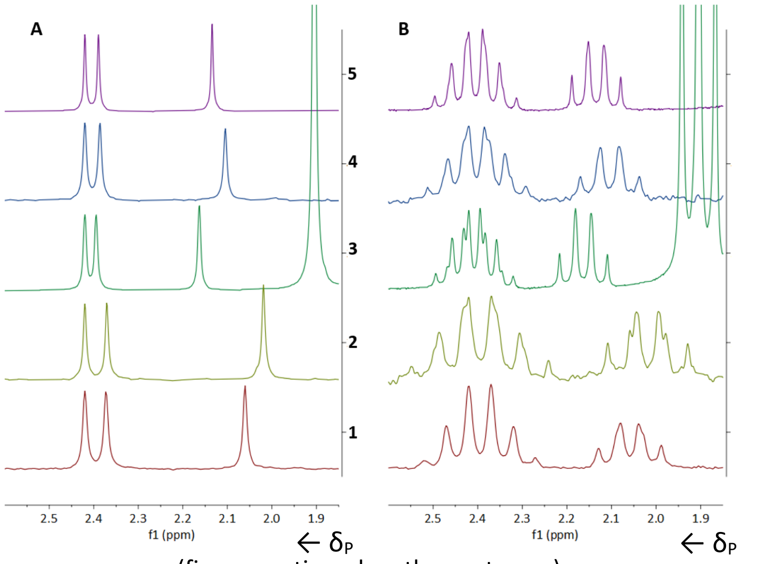

(figure continued on the next page)

C

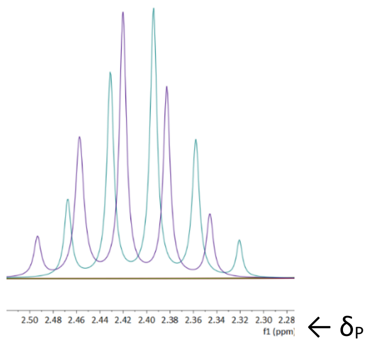

D

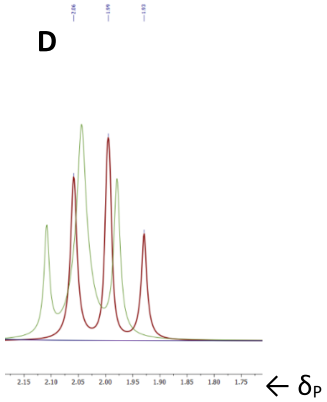

Supplementary Fig. 22. ^{31}P NMR spectra in DMSO-*d*6, selected signals (δ_P = 2.7-1.7, cf. Supplementary **Fig. 19**) at 202.5 MHz and 242.9 MHz. The stacked spectra are aligned on the peak maximum of the most downfield quintet (δ_P = 2.42).

A – ^{31}P{^{1}H} NMR (^{1}H-decoupled)

B – ^{31}P NMR (fully coupled)

C – Deconvoluted 2 x *quint* from **B** line **3**

D – Deconvoluted *dt* from **B** line **2**

Line 1 – unlabelled (202.5 MHz, DMSO-*d*6): **5**:**2a**:**P$_i$** (1:1:1, 30 mmol each): δ_P = 2.42, 2.37 (2 *quint*, $^3J_{H,P}$ = 9.5 Hz each), 2.06 (*dt*, $^3J_{H,P}$ = 8.7, 9.7 Hz). **Line 2** – ^{13}C-labelled: (202.5 MHz, DMSO-*d*6): **5**:**2c**:**P$_i$** (1:1:1, 3 mmol each): δ_P = 2.42, 2.37 (*2 quint,* $^3J_{H,P}$ = 10.3 Hz each), 2.02 (*dt,* $^3J_{H,P}$ = 10.2, 8.1 Hz). **Line 3** – ^{13}C-labelled: (242.9 MHz, DMSO-*d*6): **5**:**2c**:**P$_i$** (1:1:1, 1 mmol each): δ_P = 2.42, 2.39 (2 *quint*, $^3J_{H,P}$ = 8.9 Hz each), 2.16 (*dt*, $^3J_{H,P}$ = 8.8, 8.5 Hz). **Line 4** – ^{15}N-half labelled, half unlabelled: (202.5 MHz, DMSO-*d*6) **5**:**2a**:**2b**:**P$_i$** (1:0.5:0.5:1, 1mmol each): δ_P = 2.42, 2.39 (2 *quint*, $^3J_{H,P}$ = 9.3 Hz each), 2.10 (*dt*, $^3J_{H,P}$ = 9.0, 8.5 Hz). **Line 5** – ^{15}N-labelled: (242.9 MHz, DMSO-*d*6) **5**:**2b**: **P$_i$** (1:1:1, 1 mmol each), δ_P = 2.42, 2.39 (2 *quint*, $^3J_{H,P}$ = 9.2 Hz each), 2.13 (*dt*, $^3J_{H,P}$ = 9.0, 8.5 Hz).

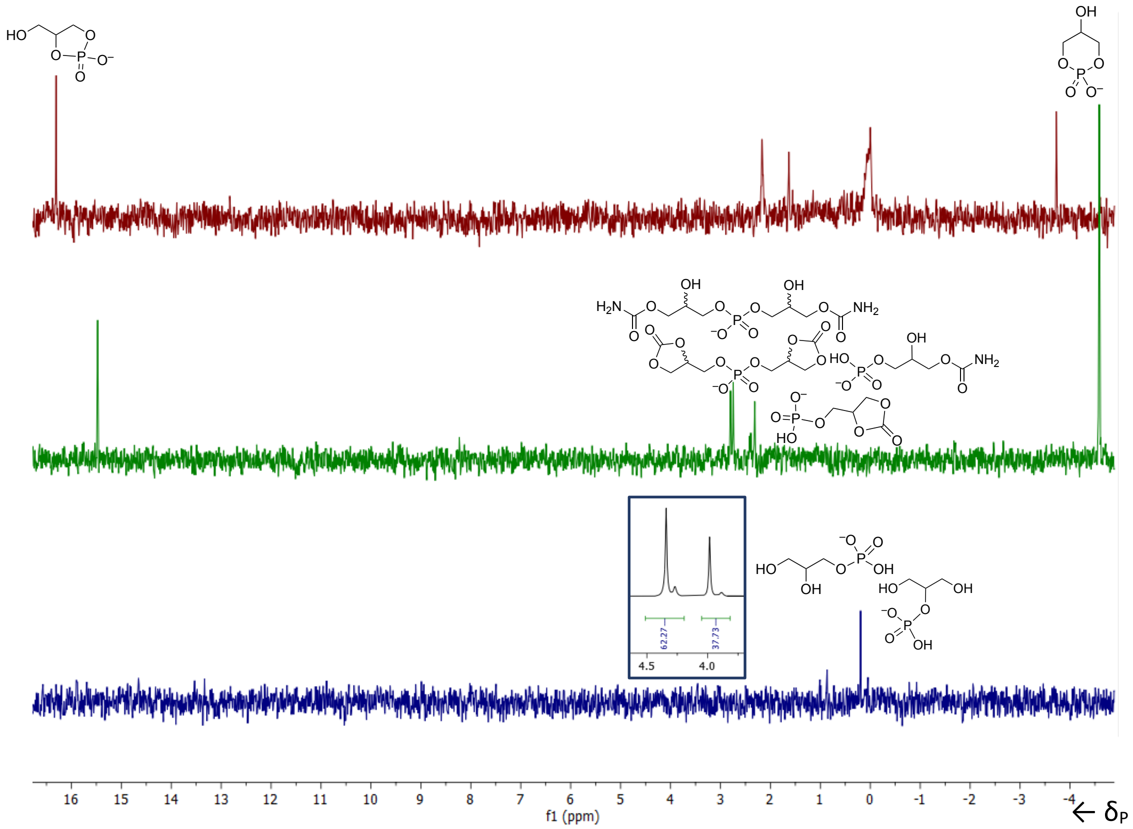

Supplementary Fig. 23. ^{31}P{^{1}H} NMR (202.5 MHz, DMSO-*d*6) of the extract of 0.5 mmol scale mixture of: **lower spectrum** – Reference spectrum in DMSO-*d*6 of commercial glyceryl phosphate (**5a**) without heating (framed insert: same in D_2O); **middle spectrum** – **5a**:**2a** (1:1) after 96 hours of heating neat at 115 °C; **upper spectrum** – **5a**:**2a**:**P$_i$** (1:1:1) after 96 hours of heating at 115 °C.

The lower spectrum in Supplementary **Fig. 23** shows the $^{31}P\{^1H\}$ NMR reference spectrum of the starting molecules **5a** in DMSO-*d*6. A better spectrum was obtained in D_2O revealing a 63:37 ratio for glyceryl-1- and glyceryl-2-phosphates by peak area integration (see insert).

After heating a neat equimolar mixture of **5a** and urea for 4 days at 115 °C, the DMSO-*d*6 extract showed in the $^{31}P\{^1H\}$ NMR spectrum that the starting molecules were absent. Instead, 15.9 % 5-membered ring cyclophosphate (δ_P = 15.5 ppm) was observed, that could have cyclized from both **5a** isomers, and 53.5 % 6-membered ring cyclophosphate (δ_P = –4.6 ppm), that could cyclize only from glyceryl-1-phosphate (middle spectrum of Supplementary **Fig. 23**). In addition, more than three resonances appeared in between those of the cyclic phosphates quite strongly downfield between 2.2 and 3.0 ppm. These are likely to be due to the carbamoylation and/or carbonylation of the glyceryl phosphates **5a**. We assign (see later for the justification) the double-resonances at the downfield-end of acyclic phosphate region (δ_P = 2.9-2.4 ppm) to diastereoisomeric dicarbonyl and/or dicarbamoyl diglyceryl phosphate diesters (17.6 %, double-resonance at $\delta_P \approx 2.9$) and the single resonance to 1,2-cyclic carbonyl or 1-carbamoylglyceryl-3-phosphate or other acyclic phosphates (14.0 % at $\delta_P \approx 2.45$).

In the upper spectrum of Supplementary **Fig. 23**, the broad peak at 0 ppm (53.1%) is due to unreacted inorganic phosphate $\mathbf{P_i}$ encompassing the signal of unreacted **5a**. The main organic products are 9.6 % 5-membered ring cyclophosphate (δ_P = 16.3 ppm), 8.7 % 6-membered ring cyclophosphate (δ_P = –3.5 ppm) and two signals in the normal acyclic glyceryl phosphate region (28.4 % at $\delta_P \approx$ 2.2-1.5 ppm). In the presence of $\mathbf{P_i}$ added to **5a** and urea the above mentioned double-resonances were not detected.

Subsequently, the solution giving spectrum 2 of Supplementary **Fig. 23** was heated to 40 and 60°C, in order to observe the temperature effect on the ^{31}P NMR signals, and then D_2O was added (Supplementary **Fig. 24**). The spectra 1-3 of Supplementary **Fig. 24** show that, upon heating, the signal width and the difference in the chemical shift $\Delta\delta_P$ of the double-resonances became smaller (★ major at 3.15-3.00 ppm, minor: see arrows). In the presence of 10 % *v/v* D_2O in DMSO-*d*6 the double-resonance coalesced. In a similar experiment as shown in the spectrum of Supplementary **Fig. 19A**, obtained after heating a neat equimolar mixture of glycerol (**5**), natural isotope abundance-urea (**2a**) and NaH_2PO_4 (1:1:1) for 120 hours at 115 °C, we diluted a 0.5 ml-DMSO-*d*6 extract with 0.5 ml D_2O and the same coalescence of the corresponding signals, that is, of the double-resonance at δ_P = 2.64 and 2.69 ppm in Supplementary **Fig. 19**, was observed (not shown).

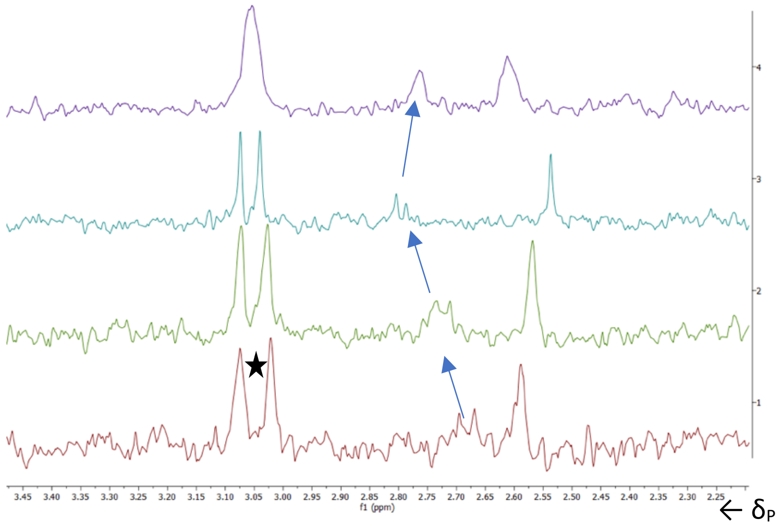

Supplementary Fig. 24. Stacked $^{31}P\{^1H\}$ NMR spectra at 202.5 MHz of selected signals at 2.2-3.5 ppm from spectrum 2 of Supplementary **Fig. 23** (DMSO-*d*6) of the experiment crude mixture starting from commercial glyceryl phosphate (**5a**) and natural isotope abundance-urea (**2a**) (1:1) after 96 h of heating at 115 °C. The spectra are aligned on the most downfield peak at δ_P = 3.07 of the larger double-resonance (star). Lane **1**: spectrum made at room temperature. Lane **2**: spectrum made at 40 °C. Lane **3**: spectrum made at 60 °C. Lane **4**: spectrum made at room temperature after adding 0.1 ml of D_2O to the stock solution (DMSO-*d*6 : D_2O ≈ 9:1 *v/v*). The arrows indicate a minor double-resonance that coalesces in the presence of D_2O, too.

4.1.1.1. Proton-decoupled heteronuclear multiple-bond correlation and J-modulated spin-echo ^{13}C nuclear magnetic resonance spectroscopies

For a better understanding of the molecular structure of these downfield-shifted phosphoglyceryl carbonyl compounds, as they were recurrently produced in all urea-assisted glycerol phosphorylations under 'dry' conditions, we analysed the DMSO-*d*6 extracts by NMR spectroscopy equipped with a quadruple resonance inverse cryoprobe (QCI). In principle, the carbonyl group could either be attached to an oxygen of the glycerol backbone or directly bound to the phosphate group which would imply the formation of carbamoyl phosphates or phosphoryl carbonates. Yet another possibility would be the formation of carbonyl phosphoramidates and those bearing a cyclic carbonate elsewhere in the molecule. These possibilities can be distinguished by using labelled urea and measuring any $^{1\text{-}3}$J couplings in the reaction mixture and correlations between ^{31}P, ^{15}N and ^{13}C by means of proton-decoupled HMBC spectroscopy.

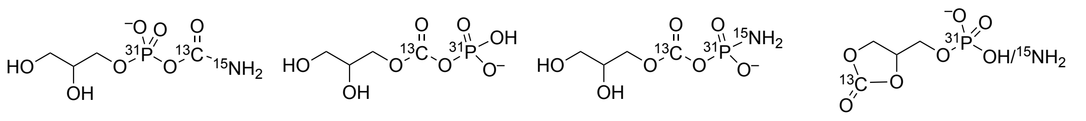

carbamoyl phosphate   phosphoryl carbonate   carbonyl phosphoramidate   cyclic carbonyl phosph(ate/oramidate)

DMSO-*d*6 extracts of neat equimolar reaction mixtures containing glycerol (**5**), [$^{15}N_2$]urea (**2b**) or [^{13}C]urea (**2c**) and inorganic phosphate (**P$_i$**), that have been heated for 96 hours at 115 °C, have failed to give evidence for the presence of carbamoyl phosphates, phosphoryl carbonates or phosphoramidates: no ^{13}C-^{31}P couplings or 2D correlations of resonances in the carbonyl ^{13}C frequency range were found whatsoever, and no ^{15}N-^{31}P correlations either (not shown). All ^{13}C-^{31}P couplings found through ^{13}C{^{31}P} decouplings and all heteronuclear cross correlations, that have been observed between these nuclei by ^{1}H-decoupled HMBC spectroscopy, were those between non-carbonyl (glyceryl) ^{13}C and ^{31}P nuclei (Supplementary **Fig. 25**). A JMOD ^{13}C NMR spectrum allows us to confirm the structure and position of phosphorus in the molecule. At a ^{13}C field strength of 150.9 MHz (600 MHz for ^{1}H) ^{13}C signals that couple with a ^{31}P nucleus (> 1.5 Hz) appear as ^{13}C-doublets. In the ^{31}P{^{1}H}-^{13}C{^{1}H} HMBC spectrum we can see short range ($^{2}J_{C,P}$) and longer range ($^{3}J_{C,P}$) correlations of carbon atoms of the glyceryl part with phosphate groups. The display of JMOD ^{13}C NMR spectra of the glyceryl C atoms allowed for the elucidation of the structures of both carbonylated phosphoglyceryl products (Supplementary **Fig. 25B**).

The ^{31}P{^{1}H}-^{13}C{^{1}H} HMBC spectra taken at $J_{C,P}$ = 5 Hz showed clearly the expected correlations for the 5-membered-ring glyceryl cyclic phosphate: δ_P = 15.9 with δ_C = 63.00 ppm (*d*, $^{2}J_{C,P}$ = 4.8 Hz, CH_2), δ_C = 65.46 ppm (br *s*, $^{3}J_{C,P}$ < 1.5 Hz, CH_2) and δ_C = 75.22 ppm (br *s*, $^{3}J_{C,P}$ < 1.5 Hz, CH), and the 6-membered-ring glyceryl cyclic phosphate (**5cGlP** and **6cGlP**, cf. Supplementary **Fig. 25A**): δ_P = –4.7 with δ_C = 64.04 ppm (*d*, $^{2}J_{C,P}$ = 4.8 Hz, CH) and δ_C = 70.09 ppm (*d*, $^{3}J_{C,P}$ = 5.7 Hz, CH_2). The known acyclic glyceryl phosphates could be more readily identified at $J_{C,P}$ = 10 Hz. The ^{31}P{^{1}H} resonance at δ_P = 1.12 ppm (triplet in proton-coupled ^{31}P) correlates with δ_C = 66.40 ppm (*d*, $^{2}J_{C,P}$ = 5.4 Hz, CH_2) and δ_C = 71.87 ppm (*d*, $^{3}J_{C,P}$ = 5.3 Hz, CH) which is correct for glyceryl-1-phosphate. The smaller ^{31}P{^{1}H} resonance at δ_P = 1.03 ppm (doublet in proton-coupled ^{31}P) correlates with δ_C = 63.03 ppm (*d*, $^{2}J_{C,P}$ = 4.8 Hz, CH_2) and δ_C = 76.87 ppm (*d*, $^{3}J_{C,P}$ = 4.9 Hz, CH) as expected from glyceryl-2-phosphate (both abbreviated **GlP**, cf. Supplementary **Fig. 25B**). In the same HMBC spectrum, the ^{31}P{^{1}H} single-resonance at δ_P = 1.38 ppm (2.29 ppm in Supplementary **Fig. 19**, between 2.0 and 2.2 ppm in Supplementary **Fig. 22A**) correlates with two different glyceryl moieties, so we should expect an asymmetric acyclic phosphodiester. This is consistent with the multiplicity of the ^{1}H-coupled ^{31}P resonance: a doublet of triplets at 202.5 MHz or a quartet at 242.9 MHz (*dt* or *q*, cf. Supplementary **Fig. 22B**), and the ^{1}H-^{31}P HMBC spectrum (red-brown correlation lines in Supplementary **Fig. 20**) indicating the connection of the phosphate with a secondary ($\delta_H \approx$ 3.91 ppm) and a primary ($\delta_H \approx$ 3.72 ppm) alcohol oxygen atom. The ^{13}C chemical shifts of one glyceryl residue: δ_C = 66.82 ppm (*d*, $^{2}J_{C,P}$ = 5.7 Hz, CH_2) and δ_C = 71.84 ppm (*d*, $^{3}J_{C,P}$ = 5.2 Hz, CH), are similar to those of 1-glyceryl phosphate and those of the other glyceryl residue: δ_C = 63.06 ppm (*d*, $^{3}J_{C,P}$ = 4.2 Hz, CH_2) and δ_C = 77.48 ppm (*d*, $^{2}J_{C,P}$ = 5.1 Hz, CH), resemble 2-glyceryl phosphate although both carbon atoms that are closer to the phosphate ($^{2}J_{C,P}$ doublets) are much downfield-shifted when compared to the main glyceryl phosphates, by $\Delta\delta_{CH2}$ = 0.42 ppm and $\Delta\delta_{CH}$ = 0.61 ppm.

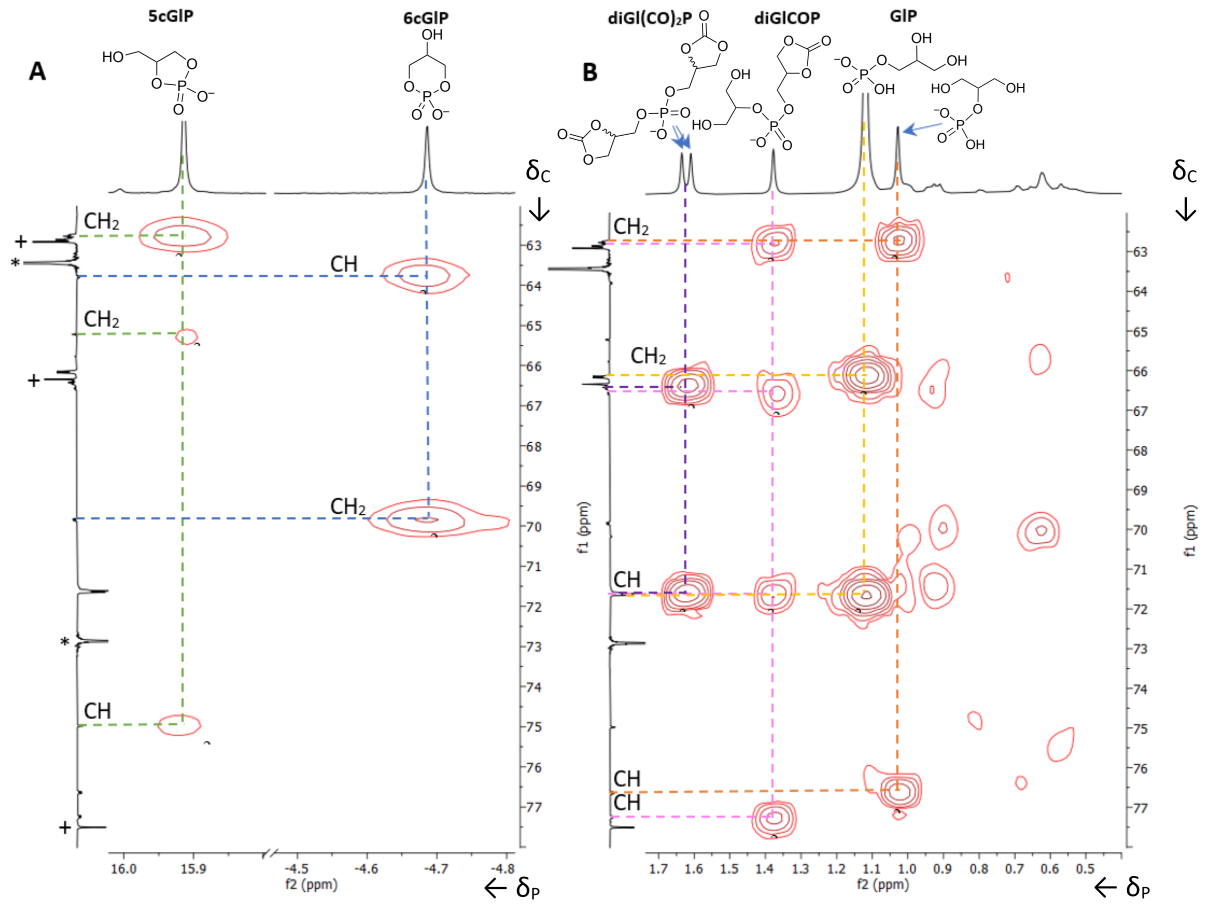

Supplementary Fig. 25. $^{31}P\{^1H\}$-$^{13}C\{^1H\}$ HMBC spectra (242.9 MHz for $^{31}P\{^1H\}$ [horizontal axis], 150.9 MHz for ^{13}C [vertical axis, JMOD], DMSO-*d*6) of the extract from a neat mixture of glycerol (**5**), [^{13}C]urea (**2c**) and NaH_2PO_4 (**P**$_i$) (1:1:1, 0.5 mmol each) after heating for 96 hours at 115 °C. **A** – ^{13}C-middle field (62-78 ppm)/^{31}P-low-field (15.8-16.0 ppm) and high-field (–4.5 to –4.8 ppm) excerpts of HMBC taken at $J_{C,P}$ = 5 Hz; * = glycerol; **+** = glyceryl-1,2-cyclic carbonate. **B** – ^{13}C-middle field (62-78 ppm)/^{31}P-middle-field (0.4-1.7 ppm) excerpt of HMBC taken at $J_{C,P}$ = 10 Hz. Correlation of ^{13}C and ^{31}P signals appear as cross-spots through 2-3 consecutive chemical bonds ($^{2-3}J_{C,P}$) in the molecules produced during the urea-assisted phosphorylation reaction. The C-P coupling ranges (^2J or ^3J) indicated on cross-spots are consistent with the multiplicity and coupling constant $J_{H,P}$ of the corresponding ^1H-coupled ^{31}P NMR resonances (Supplementary **Fig. 20B** and Supplementary **Fig. 21C**).

Taken together and keeping in mind that the ^1H-coupled ^{31}P *dt* and *q* multiplicities are not visibly influenced by the presence of ^{15}N isotopes (Supplementary **Fig. 22B** lanes 1, 4 and 5) but strongly by the presence of ^{13}C carbonyls (Supplementary **Fig. 22B** lanes 1, 2 and 3), this molecule most likely is 1,3-dihydroxypropan-2-yl ((2-oxo-1,3-dioxolan-4-yl)methyl) phosphate or, in more descriptive words, a 5-membered ring 1,2-cyclic carbonate of a diglyceryl phosphodiester one glyceryl of which is linked through the secondary and the other through the primary position (**diGlCOP**, cf. Supplementary **Fig. 25B**). The mono-anionic mass peak *m/z* = 271.0224 Dalton is indeed present in the negative ion mode mass spectrum of the methanolic extract of the unlabelled sample (Supplementary **Fig. 27**) and the signature of 1,2-cyclic carbonates is visible in the ^1H NMR spectrum of the mixture (Supplementary **Fig. 16**-Supplementary **Fig. 17**). The other 6-membered ring isomer (1,3-cyclic carbonate) or the variant containing two cyclic 1,2- and 1,3-carbonates cannot be categorically excluded, however, any acyclic carbonate (containing one to three carbonic acid monoester groups) seems too prone to decarboxylate after many days at 115 °C, and no MS evidence supports any of these alternatives.

Following the same pattern, we conclude that the $^{31}P\{^1H\}$ double-resonance at (here) δ_P = 1.63 and 1.61 ppm (2.69 and 2.64 ppm in Supplementary **Fig. 19**, around 2.4 ppm in Supplementary **Fig. 22A**) is due to an acyclic phosphate ester group linked to the primary position of glycerol, thus, correlating with a $^{13}C\{^{31}P\}$ double-resonance at δ_C = 66.64 and 66.68 ppm (2 *d*, $^2J_{C,P}$ = 5.7 Hz each, 2 x

CH_2) and with δ_C = 71.84 (*d*, $^3J_{C,P}$ = 5.3 Hz, CH). The ^1H-^{31}P HMBC spectrum shows the connection of the phosphate with a primary alcohol only (^{31}P correlation with δ_H = 3.60-3.65 ppm, cf. Supplementary **Fig. 20B**). The fact that each $^{31}P\{^1H\}$ signal splits into a ^1H-coupled quintet (2 x *quint* around δ_P = 2.4 ppm in Supplementary **Fig. 22B**) means there are four magnetically equivalent protons in the vicinity of the ^{31}P nucleus indicating the connection of the phosphate with two primary alcohol oxygen atoms. The shift in coupling pattern of the corresponding ^1H-coupled ^{31}P resonances in the absence or presence of ^{13}C-labelled atoms from [^{13}C]urea indicates the presence of a carbonyl group in this molecule (lanes 1 and 3 in Supplementary **Fig. 22B**). However, the ^{31}P downfield shift of 0.2-0.4 ppm with respect to the aforementioned 1,2-cyclic carbonate **diGlCOP** (Supplementary **Fig. 22A**) suggests the presence of two carbonyl groups and the existence of only two ^{31}P-^{13}C cross correlations for this molecule gives evidence for its symmetrical constitution, which means that it is *bis*-((2-oxo-1,3-dioxolan-4-yl)methyl) phosphate, that is, the symmetric phosphodiester of two primary 3-glyceryl-1,2-cyclic carbonates (**diGl(CO)₂P**, cf. Supplementary **Fig. 25B**).

The peculiarity of this molecule is that it shows a 1:1 double-resonance in anhydrous DMSO, both in $^{31}P\{^1H\}$ and $^{13}C\{^{31}P\}$ NMR spectra, that persists at elevated temperatures up to 60 °C and coalesces to a single somewhat broad resonance only in the presence of water, which is consistent with the existence of two equally probable diastereoisomers. The mono-anionic mass peak *m/z* = 297.0017 Dalton is not present in the negative ion mode mass spectrum of the methanolic extract of the unlabelled sample (Supplementary **Fig. 27**). LC-HRMS using the HILIC column (HILIC-HRMS) of the methanolic extract of the unlabelled mixture does not show the presence of any identifiable carbamoyl or cyclic carbonate derivative of glyceryl phosphates. However, the highest intensities of the extracted peaks in both positive and negative ion mode chromatograms have the mass of diglyceryl phosphate (peak no. 4 in **A** and, respectively, peak no. 5 in **C**, cf. Supplementary **Fig. 28**). A study of kinetics of the time-dependent growth of the amounts of both carbonylated glyceryl phosphates as observed by ^1H, ^{13}C and $^{31}P\{^1H\}$ NMR spectroscopies are described in section **5**.

The remaining major (fully coupled) ^{13}C NMR resonances in this middle-field chemical shift range at 62-78 ppm do not couple or correlate by $\{^1H\}$ HMBC with the ^{31}P nucleus: three CH_2 singlets at δ_C = 63.16 ppm (small *s*, CH_2), 63.68 ppm (large *s*, CH_2) and 66.58 ppm (small *s*, CH_2) besides two CH singlets at δ_C = 73.16 ppm (medium *s*, CH) and 77.75 ppm (small *s*, CH), Supplementary **Fig. 25A**. The large/medium signals are those of glycerol (marked with an asterisk *), the small signals are from glyceryl-1,2-cyclic carbonate (marked with a **+** sign). Both compounds are readily identified in the ^1H-spectrum (Supplementary **Fig. 16**-Supplementary **Fig. 17**), the cyclic carbonate also in the $^{13}C\{^{31}P\}$ NMR spectrum of the ^{13}C-labelled sample (Supplementary **Fig. 26**). In this downfield chemical shift range all carbonyls are seen best, because they are fully enriched in this isotope. The corresponding mixture obtained after using [$^{15}N_2$]urea is of course much less sensitive but still permits the identification of otherwise not observed residual urea (triplet), glyceryl carbamate (doublet) and cyclic carbonate (singlet), see Supplementary **Fig. 26C**.

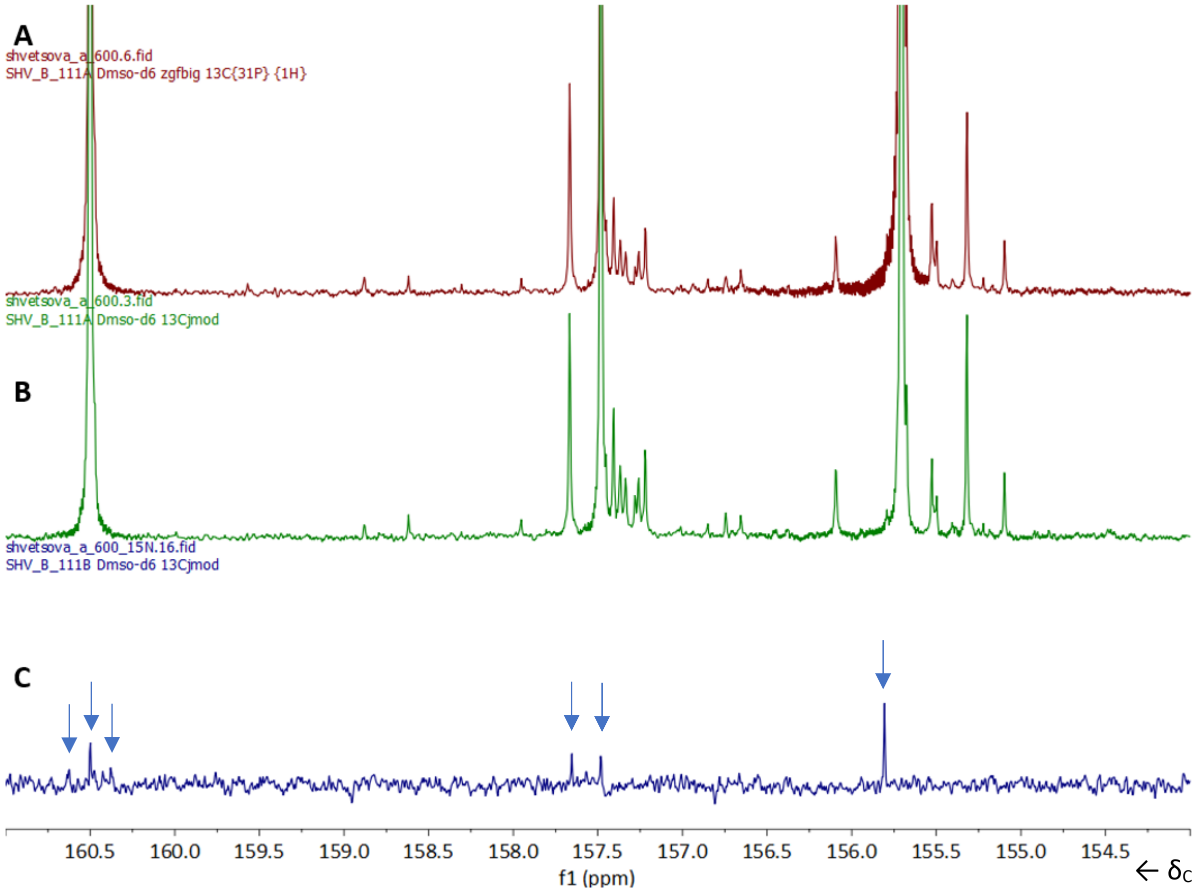

Supplementary Fig. 26. ^{13}C NMR (150.9 MHz) low-field excerpts of the DMSO-*d*6 extracts from a neat mixture of **A** (**upper spectrum**) $^{13}C\{^{31}P\}$ NMR, **B** (**middle spectrum**) ^{13}C JMOD NMR both of glycerol (**5**), [^{13}C]urea (**2c**) and NaH_2PO_4 (**P**$_i$) (1:1:1, 0.5 mmol each); and **C** (**lower spectrum**) ^{13}C JMOD NMR of glycerol (**5**), [$^{15}N_2$]urea (**2b**) and NaH_2PO_4 (**P**$_i$) (1:1:1, 0.5 mmol each) all after heating neat mixtures for 96 hours at 115 °C. Leftmost: residual urea (triplet), central: carbamates (doublet), right-hand: cyclic carbonates (singlet).

4.1.2. High-resolution mass spectrometry and hydrophilic-ion liquid chromatography

Another method that provided us with information about reaction products is mass spectrometry (MS). Due to the small size and high polarity of the glyceryl phosphates, the most optimal analysis was high-resolution (direct injection) MS in negative ion mode (Supplementary **Fig. 27**). Taking into consideration the exact masses of the compounds, we know the most probable atomic composition of the molecules but not their structure. For example, the 5- and 6-membered ring phosphates would have exactly the same mass, *m/z* 152.9958 Dalton, similar to glyceryl-1-phosphate and glyceryl-2-phosphate, *m/z* 171.0064 Dalton. Thus, in Supplementary **Fig. 27D** only the most probable isomers of the identified products are suggested.

High-resolution mass spectrometry (HRMS) also confirms the presence of diesters such as diglyceryl phosphate and diglyceryl diphosphates, along with the carbamoyl and cyclic carbonate derivatives of (mono)glyceryl phosphates suggested in the previous section. However, the mono-anionic mass peak at *m/z* 297.0017 Dalton is not present in the negative ion mode mass spectrum of the methanolic extract of the unlabelled sample (Supplementary **Fig. 27**). In none of these mass spectra could we identify *m/z* peaks of expected, not phosphorylated molecules.

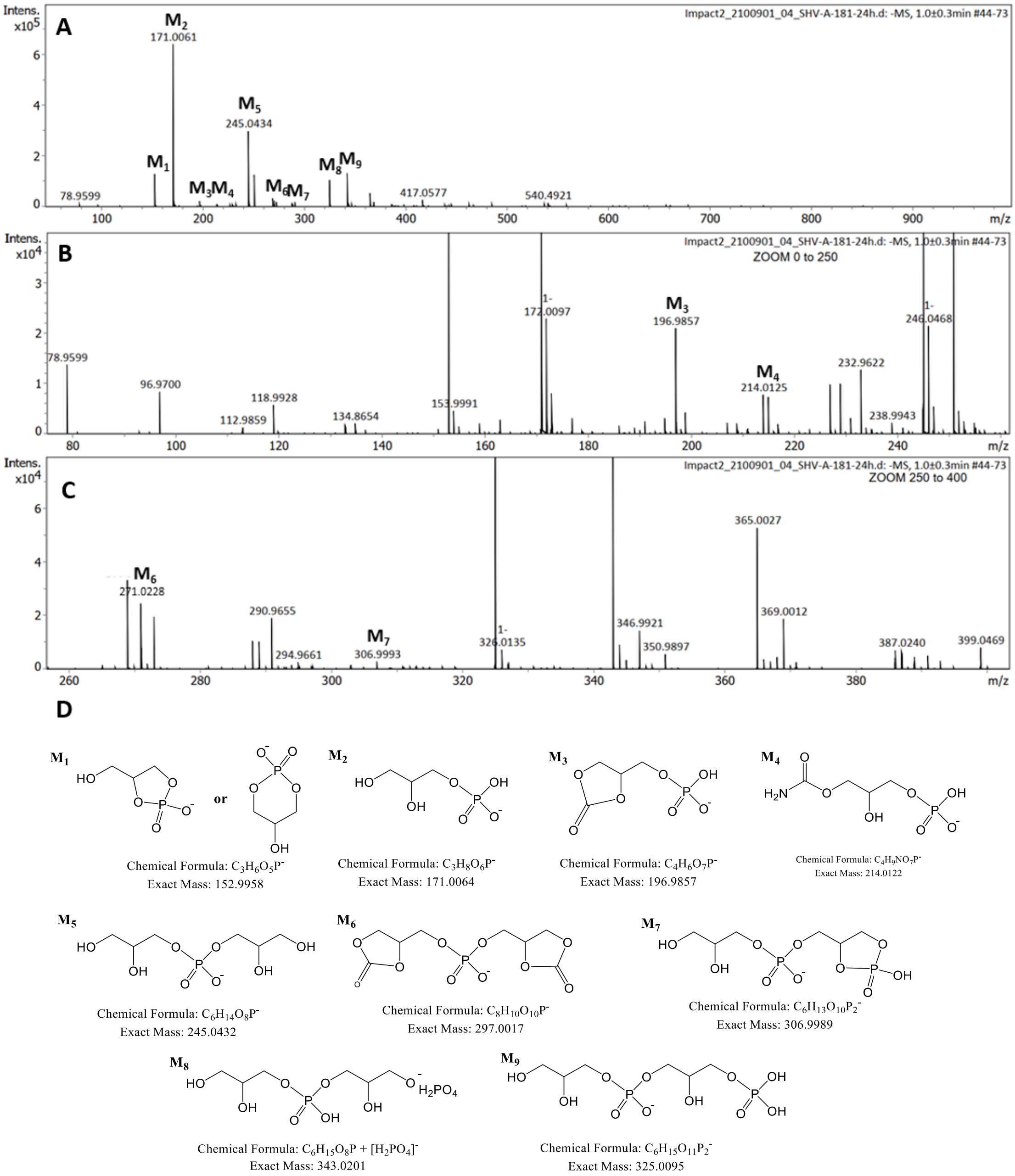

Supplementary Fig. 27. HRMS spectra (direct injection) in the negative ion mode of the methanolic extract of the crude mixture obtained from letting glycerol (**5**), urea (**2a**) and NaH_2PO_4 (**P**$_i$) (1:1:1, 0.5 mmol each) react for 24 h at 115 °C (neat). *M/z* $[H_2PO_4]^-$ 96.9696 = ESI^- common background ion. **A** – Total ion spectrum. **B**, **C** – Zoom into relevant *m/z* ranges. **D** – Assigned chemical structures (only 1-2 of several possible isomers shown) and mono-isotopic masses of the charge-neutral molecules.

We attempted to find **diGlCOP** and **diGl(CO)$_2$P** in the crude mixture by LC-HRMS using the hydrophilic-ion liquid chromatography column (HILIC-HRMS). We optimised the separation protocol after trying several possible conditions and solvent systems by using evaporative light scattering detection (HILIC-ELSD). In Supplementary **Fig. 28**, the results of the analysis in positive and negative ion modes are presented. There are similar products to the direct injection HRMS listed above, such as cyclic and acyclic mono- and diglyceryl phosphates (Supplementary **Fig. 28C**). The methanolic extract of the unlabelled mixture does not show any identifiable carbamoyl or cyclic carbonate derivatives of glyceryl phosphates. However, the highest intensities of the extracted peaks in both positive and negative ion mode chromatograms match the mass of diglyceryl phosphate (peak no. 4 in **A** and, respectively, peak no. 5 in **C**, cf. Supplementary **Fig. 28**).

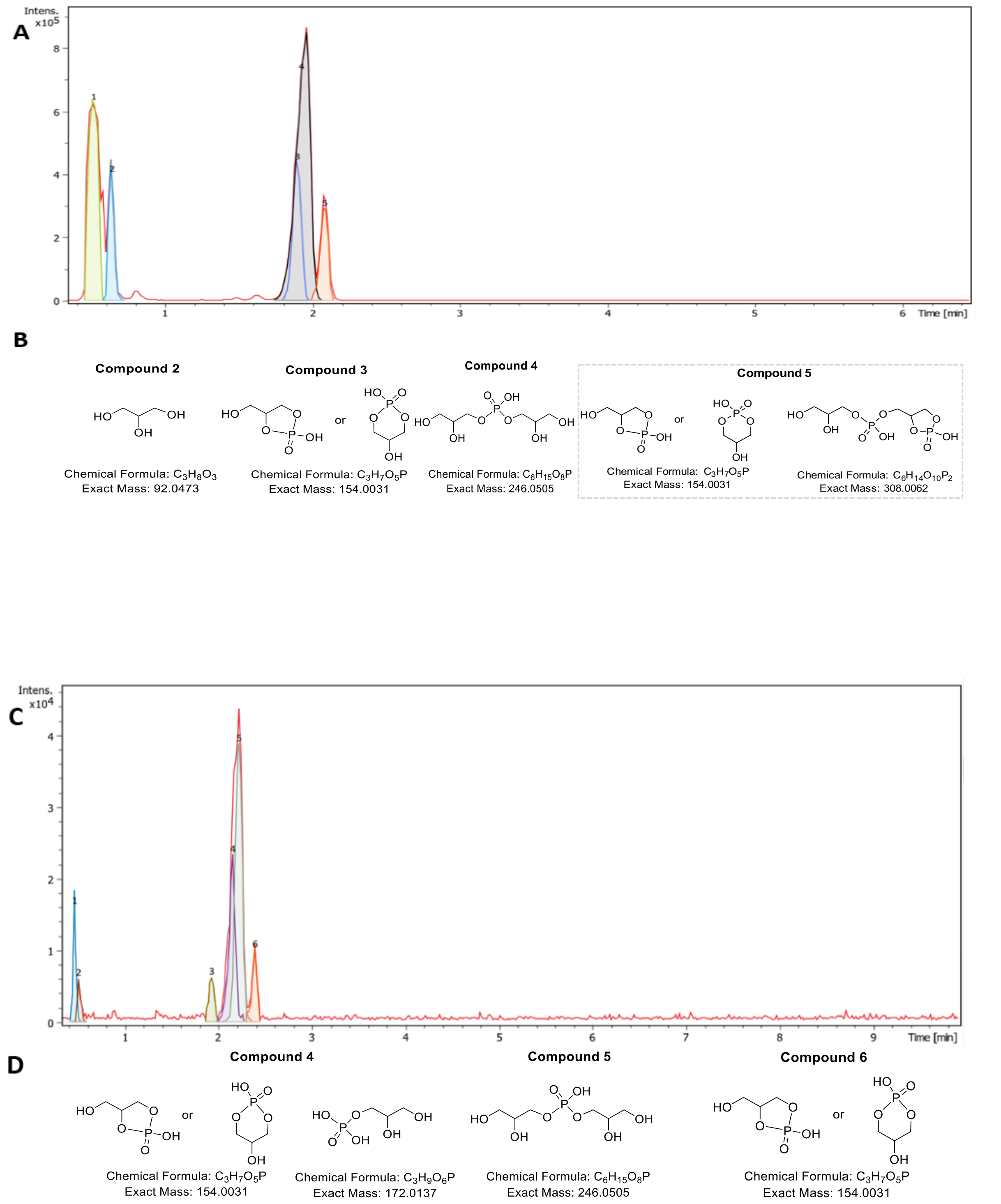

Supplementary Fig. 28. HILIC-HRMS spectra of the methanolic extract of the crude mixture obtained by letting glycerol (**5**), urea (**2a**) and NaH_2PO_4 (**P**$_i$) (1:1:1, 0.5 mmol each) react for 96 h at 115 °C (neat). **A** – Positive ion mode chromatogram and numbered ion extracts. **B** – Assigned chemical structures of extracted ion peaks found in **A** (only the most probable acyclic isomers shown), chemical formula and monoisotopic masses of the charge-neutral molecules. Peak 1 of spectrum **A** contains only sodium acetate clusters (+82n). **C** – Negative ion mode chromatogram and numbered ion extracts. **D** – Assigned chemical structures of extracted ion peaks found in **C** (only the most probable acyclic isomers shown), chemical formula and monoisotopic masses of the charge-neutral molecules. Peaks 1 and 2 of spectrum **C** are acetate adducts of a fluorinated polymer (PTFE) (from the grease used on LC pump modules) and leftovers of MS calibrant. The mass of peak 3 of spectrum **C** is closest to glyceryl methyl phosphodiester that may occur from the reaction of glyceryl phosphate with the solvent (*m/z* 186.1139 found, *m/z* 186.0293 calculated for $C_4H_{11}O_6P$) but remains unidentified.

4.2. Urea-assisted phosphorylation of MPG (6)

4.2.1. ^{1}H and ^{31}P NMR and ^{1}H-^{31}P HMBC spectroscopies

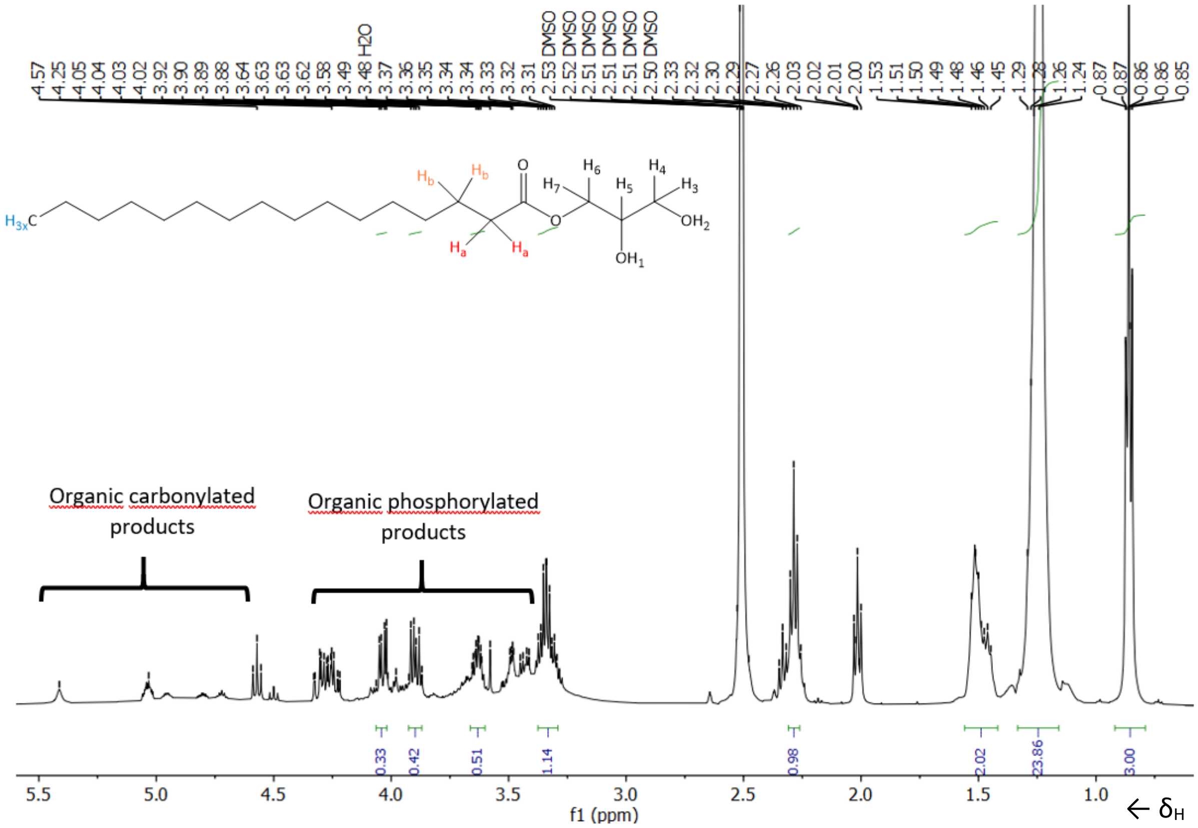

Supplementary Fig. 29. ^{1}H NMR spectrum (500 MHz, DMSO-*d6*) of the extract of the crude reaction mixture from MPG (**6**), natural isotope-abundance urea (**2a**) and NaH_2PO_4 (**P$_i$**) (1:1:1, 3 mmol each) that was heated neat for 120 hours at 115 °C : δ_H (MPG signals appear with integrated peak areas, compare with Supplementary **Fig. 1**). Signals at 3.5-4.3 ppm are due to organic products of phosphorylation, signals at 4.3-5.5 ppm contain not reacted OH groups of MPG, also 1,2-cyclic carbonates.

The ^{1}H NMR spectrum is more complicated when compared to the ^{1}H NMR spectrum of pure MPG (**6**) (the characterisation of pure MPG is described in Supplementary **Fig. 1**) due to the presence of phosphorylated and carbonylated products. Also, as a result of thermal degradation of **6**, the presence of glycerol (**5**) and considerable amounts of glyceryl phosphates are observed, as confirmed by ^{31}P NMR (Supplementary **Fig. 30**) and LC-MS (Supplementary **Fig. 33**).

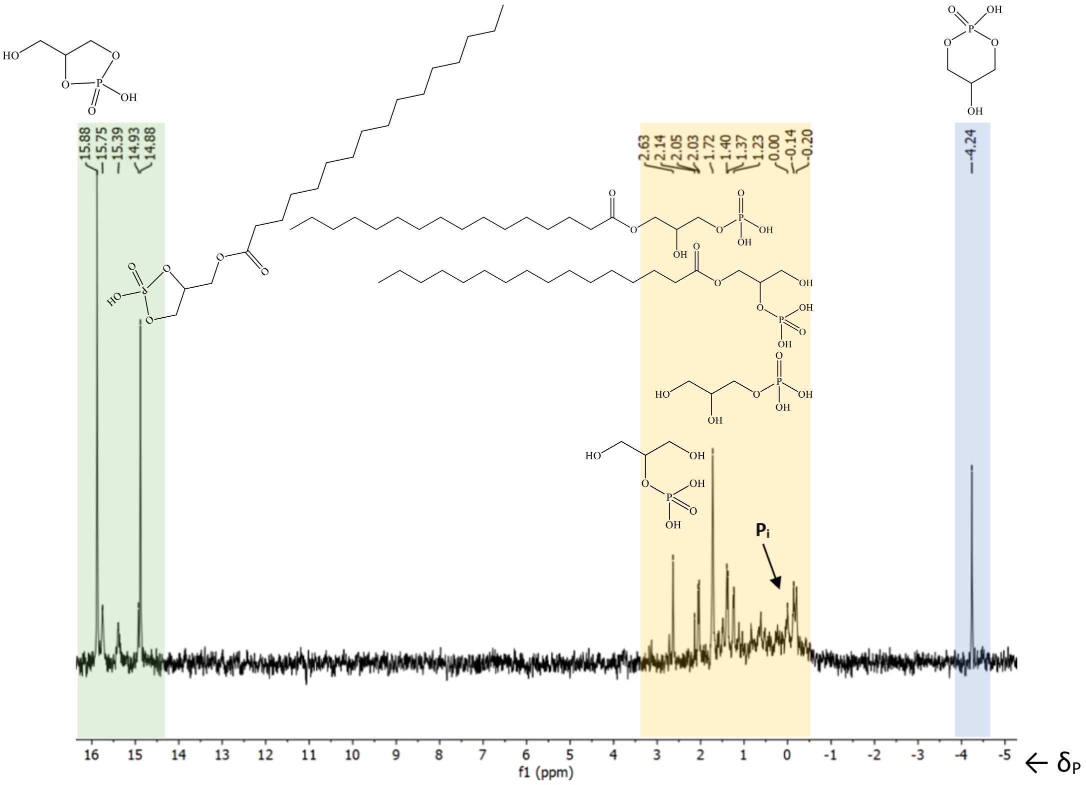

Supplementary Fig. 30. $^{31}P\{^{1}H\}$ NMR (202.5 MHz, DMSO-*d*6) of the extract of the crude reaction mixture from MPG (**6**), natural isotope-abundance urea (**2a**) and NaH_2PO_4 (**P**$_i$) (1:1:1, 3 mmol each) that was heated neat for 120 hours at 115 °C. δ_P = 15.62-14.63 (*s*, 5-membered ring cyclic phosphates containing **5cGlP** and **5cMPGP**, green), 2.38-0.46 (acyclic organo-phosphates containing **MPGP**, ochre), 0.00 (*s*, **P**$_i$), –4.49 (*s*, 6-membered ring glyceryl-1,3-cyclic phosphate **6cGlP**, blue).

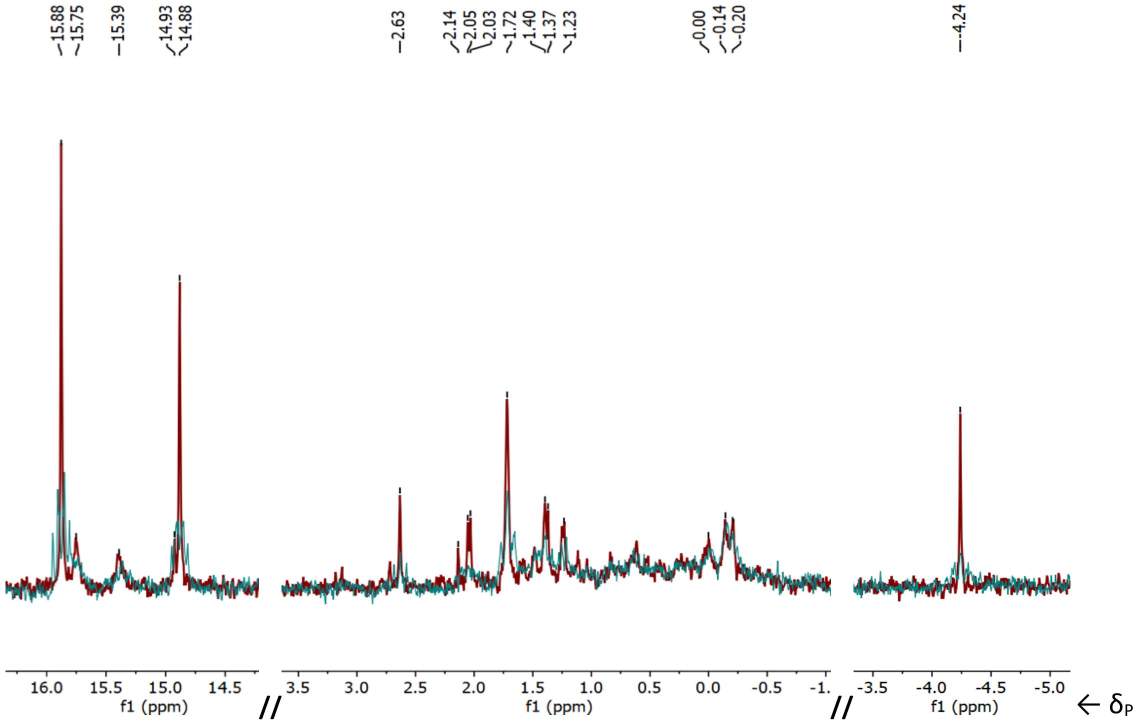

Supplementary Fig. 31. $^{31}P\{^{1}H\}$ NMR (**brown**) and ^{31}P NMR spectra (**turquoise**) (202.5 MHz) in DMSO-*d*6 of the extract of the crude reaction mixture from MPG (**6**), natural isotope-abundance urea (**2a**) and NaH_2PO_4 (**P**$_i$) (1:1:1, 3 mmol each) that was heated neat for 120 hours at 115 °C: δ_P = 15.88 (s/*ddd*, 5-membered ring cyclic phosphate **5cGlP**), 14.88 (s/*ddd*, 5-membered ring cyclic phosphate **5cMPGP**), 2.38-0.46 (acyclic organo-phosphates containing **MPGP**) including 2.05 (s/*t*, acyclic primary monoester **MPG-1P**), 1.37 (s/*d*, acyclic secondary monoester **MPG-2P**); 1.75 (s/*t*, acyclic primary monoester **Gl1P**), 0.00 (*s*, **P**$_i$); –4.24 (s/*tt(d)*, 6-membered ring cyclic phosphate **6cMPGP**).

The multiplicity pattern of organic phosphorylated products is more complicated to follow by ^{31}P NMR spectroscopy due to the lower solubility of the MPG phosphorylation reaction mixture, when compared to that of glycerol phosphorylation. Consequently, lower signal intensities and a much higher signal-to-noise ratio were observed.

However, it was possible to identify the main groups of products as shown in Supplementary **Fig. 21** and the integration of the $^{31}P\{^1H\}$ peak areas for **5cGlP** — identified through the comparison of the ^1H-^{31}P HMBC spectra of the reaction mixtures of glycerol and MPG (Supplementary **Fig. 22** and Supplementary **Fig. 32**). The main acyclic 1-glyceryl phosphate and **6cGlP** allows for a rough estimation of the amount of MPG deacylation to palmitate, glyceryl phosphates, glyceryl cyclic carbonates and glycerol. If we calculate by integrating the peak areas the relative amount of ^{31}P signals that are surely depalmitoylated, namely, the larger most downfield signal from glyceryl-1,2-cyclic phosphate (marked **x** in Supplementary **Fig. 32**) and that from glyceryl-1,3-cyclic phosphate — the 6-membered ring cyclic phosphate is expected to form due to depalmitoylation followed by cyclisation much more likely than through acyl migration followed by cyclisation — then <u>53 % depalmitoylation</u> constitutes a lower limit. This percentage could be higher if among the acyclic signals were depalmitoylated phosphates as well.

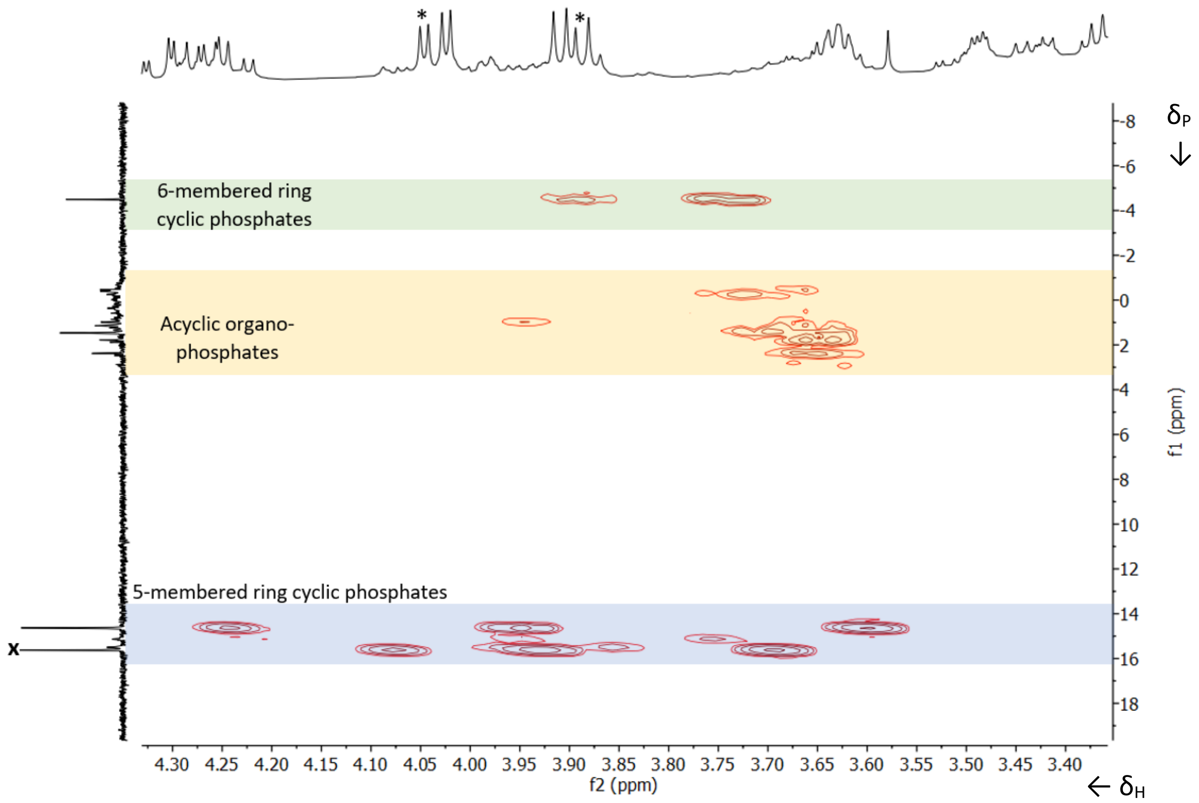

Supplementary Fig. 32. ^1H-$^{31}P\{^1H\}$ HMBC spectrum (500 MHz, ^1H [horizontal axis], 202.5 MHz $^{31}P\{^1H\}$ [vertical axis]) in DMSO-*d6* of the extract of MPG (**6**), natural isotope abundance urea (**2a**) and NaH_2PO_4 (**P**$_i$) (1:1:1, 3 mmol each) heated neat for 120 hours at 115 °C. * = **MPG** (2 *dd*), **x** = **5cGlP**.

4.2.2. Reversed-phase high-performance liquid chromatography coupled to high-resolution mass spectrometry (RP-HPLC-HRMS)

MPG (**6**), compared to glycerol (**5**), has a long chain that helps to separate molecules well without the need of using HILIC-MS. In Supplementary **Fig. 33**, we were able to identify all the products suggested by the above NMR analysis, such as 5- or 6-membered ring phosphates, acyclic glyceryl and MPG phosphates, along with carbamoyl and cyclic carbonate. Compounds (peaks) # 2, 3 and 4 contain phosphorylated MPG with an additional glycerol molecule, each compound most likely belongs to a different isomer of the same compound (Supplementary **Fig. 33B**). Other interesting products are compounds # 12-15, where we detected the presence of di-MPG phosphate and two fatty acid chains attached to one and the same phosphorylated glycerol molecule (DPG phosphate).

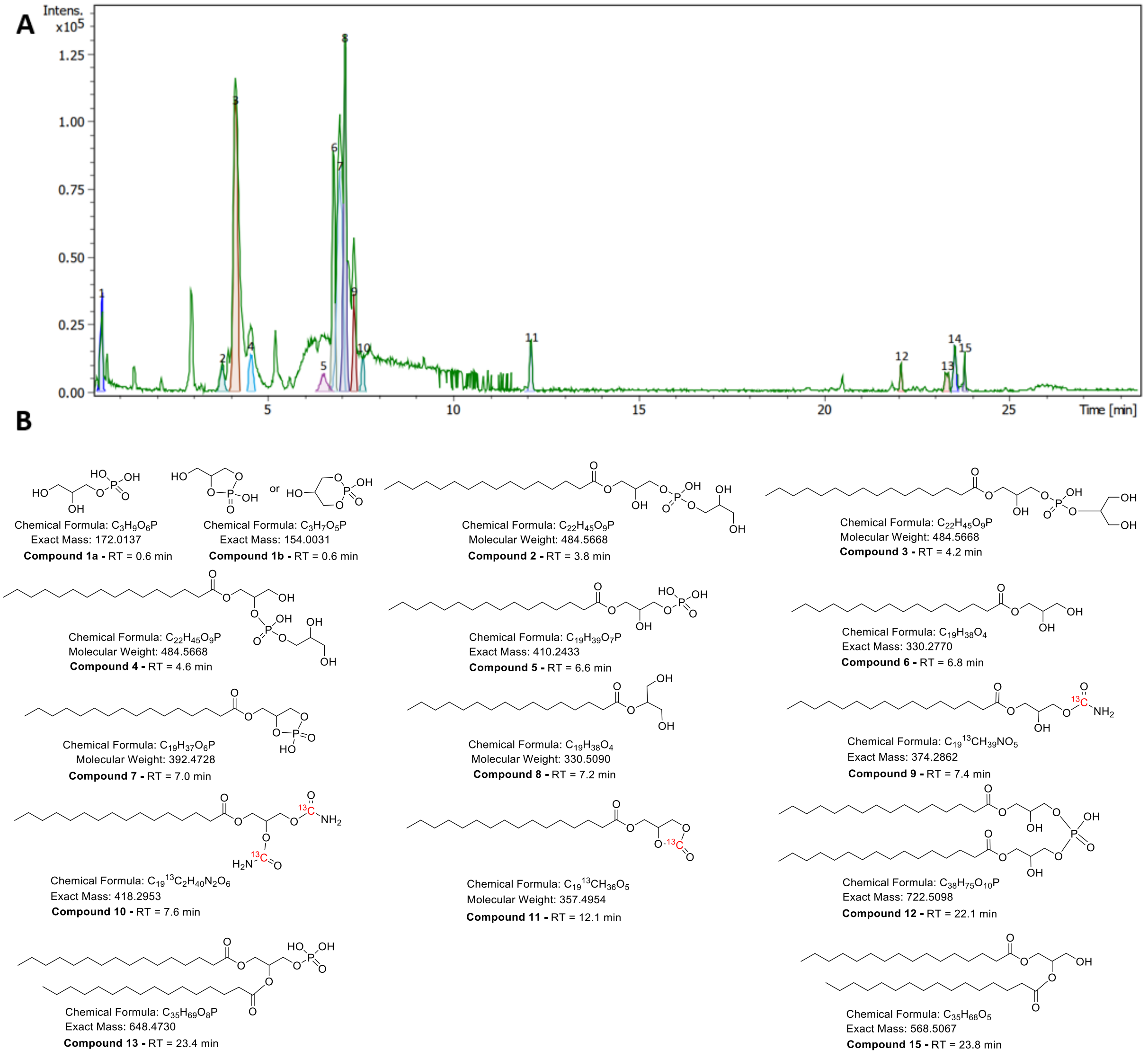

Supplementary Fig. 33. Exemplary chromatogram of a methanolic extract of the reaction mixture containing MPG (**6**), [^{13}C]urea (**2c**) and NaH_2PO_4 (**P**$_i$) (1:1:1, 0.5 mmol each) that was heated neat for 48 hours at 115 °C.
A – RP-UHPLC-HRMS chromatogram (green) with extracted negative ions (coloured, numbered peaks).
B – Chemical structures and monoisotopic masses of the detected compounds in their charge-neutral forms. Suggested molecules corresponding to groups of peaks # 2, 3, 4 (*m/z* 484.5668) and # 6, 8 (*m/z* 330.5090) have different retention times R_T but the same *m/z*, hence, are isomers. Compound # 5 shows up as very broad peak (R_T = 5.5->10 min), it is the main phosphorylated product (area under green line). Compound # 14 (R_T = 23.5 min, *m/z* 540.4903) could not be identified.

4.3. Inorganic cyanate (NCO^{-}) and carbamoyl phosphate (CP_i)

The ^{13}C chemical shift of the cyanate anion (NCO^{-}) in D_2O has been reported to resonate at δ_C = 129.43 or 129.54 ppm (s) at pH 8.5[23]. The expected ^{13}C and ^{31}P chemical shifts and multiplicities of inorganic carbamoyl phosphate (**CP_i**) in water depend on the buffer, pH and internal or external references utilised at different magnetic field strengths B_0 : δ_P = −1.41 ppm (*s*)[23] and −1.75 to −2.5 ppm (*s*)[23,27] both at B_0 = 202.5 MHz, or δ_P = −4.35 ppm (*d*, J_{CP} = 4.7 Hz) at B_0 = 121.4 MHz[28] and δ_C = 157.39 ppm (*s*) at B_0 = 125.7 MHz[23] or 156.85 ppm (*d*, J_{CP} = 4.7 Hz) at B_0 = 75.4 MHz[28]. We have never found any ^{13}C NMR peak resonating at around $\delta_C \approx$ 130 ppm in any of our DMSO-*d*6 or D_2O extracts, which would have been indicative of NCO^{-}, not in early time points of the reaction with glycerol or MPG, nor in those urea-assisted phosphorylation experiments that have been carried out with [^{13}C]urea (**5c**). Therefore, we focussed our search for an NMR spectroscopic signature for **CP_i**.

As a reference compound, we tested the commercial dibasic hydrate of the lithium salt of **CP_i** as a 0.06 mM solution in DMSO-*d*6:D_2O (5:1 *v/v*). Due to the low concentration of the solution and its poor solubility in DMSO-*d*6, we did not detect any signals by ^{13}C NMR spectroscopy. We did identify by $^{31}P\{^1H\}$ NMR spectroscopy (lower spectrum in Supplementary **Fig. 34**) two signals corresponding to **CP_i** (−1.46 ppm) and **PP_i** (−7.78 ppm).

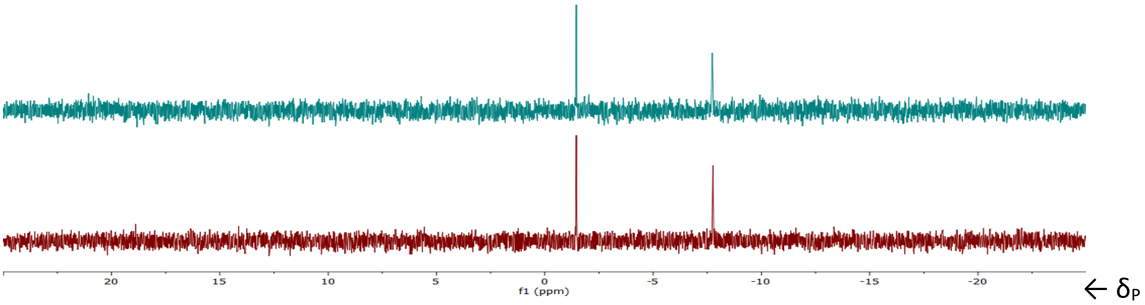

Supplementary Fig. 34. $^{31}P\{^1H\}$ (**lower spectrum**) and ^{31}P (**upper spectrum**) NMR spectra (202.5 MHz, DMSO-*d*6:D_2O 5:1 *v/v*) of 0.06 mM **CP_i** (5 mg dibasic lithium hydrate salt in 0.5-0.6 ml): δ_p = −1.46 (*s*) **CP_i** and δ_p = −7.78 (*s*) **PP_i**.

From the measured difference in decay rates of **P_i**, urea and MPG described in Section **5** (Supplementary **Fig. 47**, Supplementary **Fig. 53**, Supplementary **Table 15**, Supplementary **Table 23**) we could calculate the best range of reaction time when potentially a maximum amount **CP_i** could be present, if it formed immediately after the (early) consumption of **P_i** and urea but before the (later) consumption of considerable amounts of MPG (measured by HPLC-ELSD), which in a neat reaction mixture was most probable after 2-3 hours of heating at 115 °C.

Thus, a crude mixture of glycerol (**5**), [^{13}C]urea (**2c**) and **P_i** (1:1:1) was heated at 115°C for 2 hours. NMR acquisitions were made on "fresh" solutions of D_2O and DMSO-*d*6 prepared just before the NMR experiment made on a 600 MHz machine equipped with a QCI cryoprobe. Due to the presence of a ^{13}C-labelled carbon atom in the urea molecule, **CP_i** was expected to present itself as a doublet by, possibly both, ^{31}P NMR and, especially by ^{13}C NMR spectroscopy[28,29]. After a series of experiments, we could positively confirm the absence of an NMR signal of **CP_i** in the crude mixture after 2 hours of heating at 115 °C (Supplementary **Fig. 35**-Supplementary **Fig. 36**). There are no ^{31}P resonances of whatever multiplicity visible between δ_p = −1.46 and −4.5 ppm, not even between δ_p = −1.0 and −8.0 ppm (Supplementary **Fig. 35**)[29]! Inorganic phosphate and pyrophosphate are better soluble in water than in DMSO, thus, in the **upper** spectrum (Supplementary **Fig. 35A**) the **P_i** signal is more intense compared to that in the **middle** spectrum (Supplementary **Fig. 35B**). A **PP_i** singlet (δ_{PPi} = −8.08 ppm) is visible in water and two different PP_o resonances that could possibly belong to primary and secondary glyceryl diphosphates: δ_{PPo1} = −7.29 and −10.64 (2 x *d*, J = 20.9 Hz) and δ_{PPo2} = −6.93 and −9.95 (2 x *d*, J = 20.7 Hz). 16 scans were carried out in the spectra shown in Supplementary **Fig. 35 A** and **B**. However, when this scan number was increased to 2059 (Supplementary **Fig. 35C**), **PP_o**, **diGl(CO)$_2$P** (1.70/1.67 ppm) and **diGlCOP** (1.45 ppm) and other minor signals become visible in the DMSO-*d*6 spectrum as well.

Apart from large ^{1}H-coupled ^{13}C resonances of unreacted urea, that is, the huge singlet at 160.50 ppm in D_2O and DMSO-*d*6, the large singlet at 157.0 ppm in D_2O and 157.57 ppm in DMSO-*d*6 showing the early emergence of 1-glyceryl carbamate (compare with Supplementary **Fig. 26**) and the doublet CH and triplet CH_2 from unreacted glycerol, only tiny ^{31}P-coupled ^{13}C resonances appeared in the middle-field CH and CH_2 region from emerging 1-glyceryl phosphate and 2-glyceryl phosphate, see arrows in Supplementary **Fig. 36A**, and compare with ^{13}C JMOD NMR spectra in Supplementary **Fig. 36B**. There are no ^{31}P-^{13}C couplings visible in the carbonyl region of the fully coupled ^{13}C NMR spectrum, not even in minor peaks.

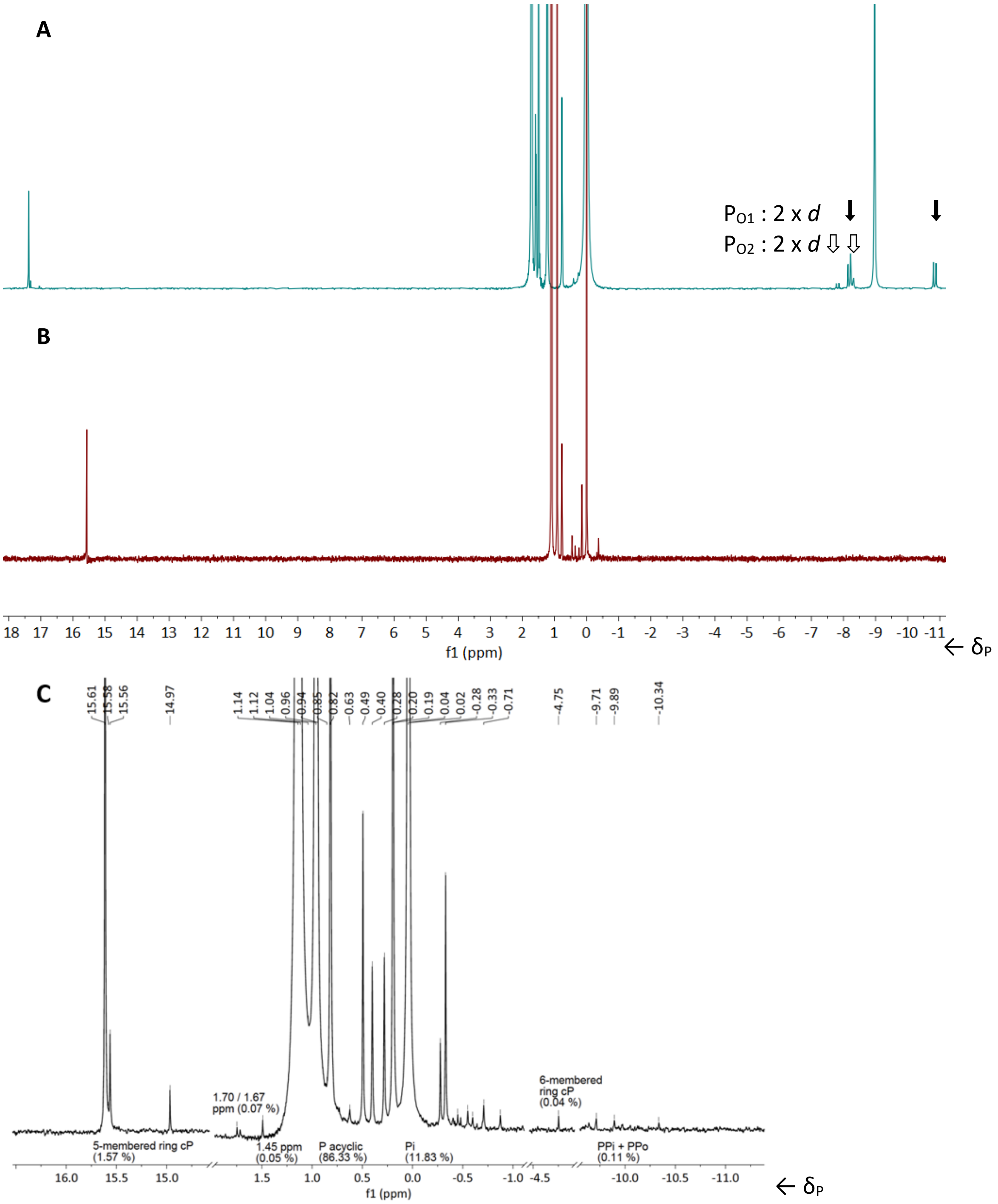

Supplementary Fig. 35. ^{31}P{^{1}H} NMR spectra (242.9 MHz) of freshly prepared extracts of a mixture of glycerol (**5**), [^{13}C]urea (**2c**) and **P**$_i$ (1:1:1, 0.5 mmol each) after 2 hrs heating neat at 115 °C. **A** (**turquoise**) – in D_2O, **B** (**brown**) and **C** (black) – in DMSO-*d*6. **A** and **B**: 16 scans, **C**: 2059 scans. All spectra referenced to internal **P**$_i$ at δ_{Pi} = 0.00 ppm.

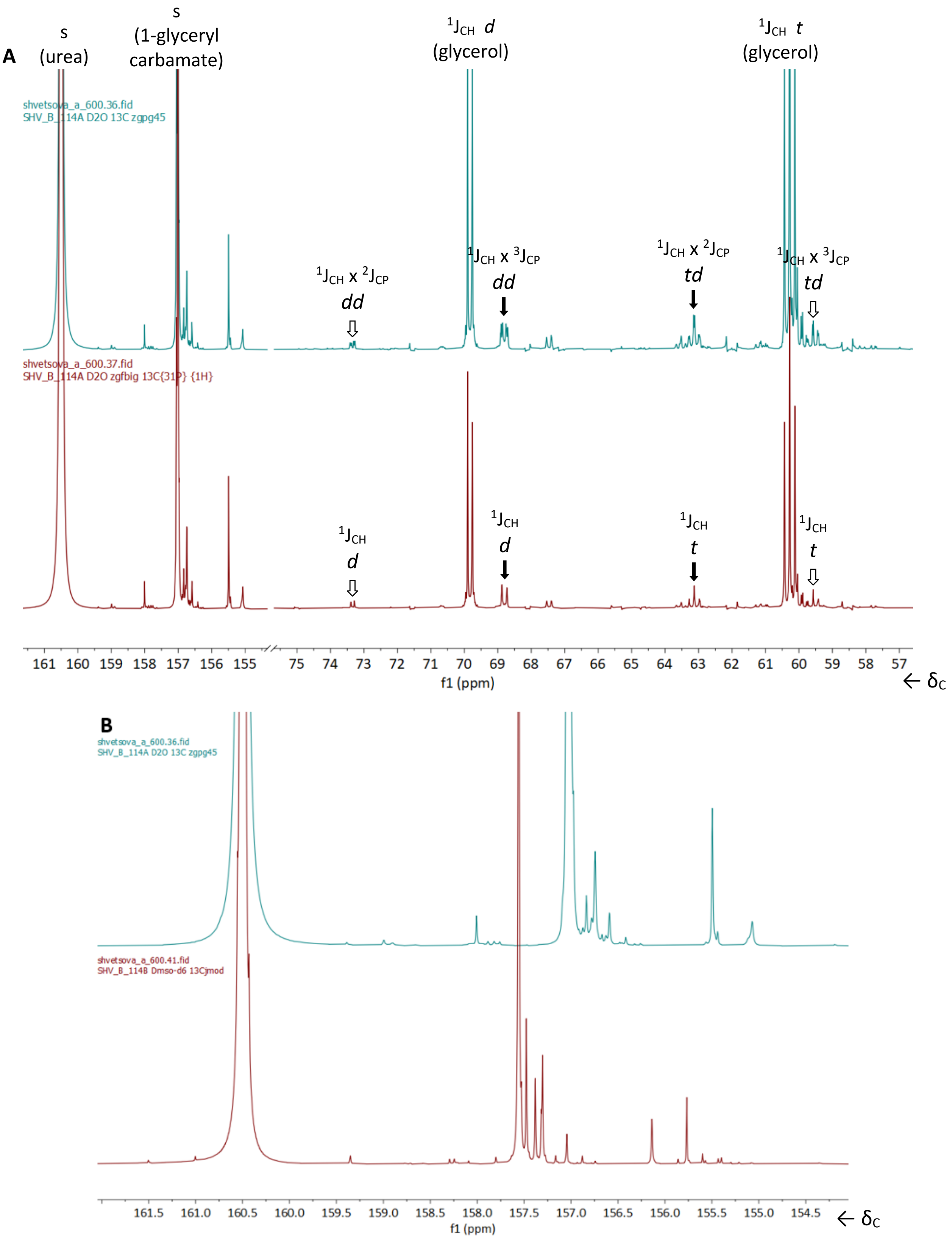

Supplementary Fig. 36. ^{1}H-coupled ^{13}C NMR spectra (150.9 MHz) of freshly prepared extracts of a mixture of glycerol (**5**), [^{13}C]urea (**2c**) and NaH_2PO_4 (**P**$_i$) (1:1:1, 0.5 mmol each) <u>after 2 hours heating neat at 115 °C</u>. **A** – NMR solvent D_2O, **lower spectrum**: ^{13}C{^{31}P} NMR ; **upper spectrum**: ^{13}C NMR. Signals showing a J_{CP} coupling are marked with their $^{1}J_{CH}$ (*t* or *d*) and a filled arrow for 1-glyceryl phosphate and an empty arrow for 2-glyceryl phosphate: δ_C = 73.2 ppm (*dd*, $^{1}J_{C(2)H}$ = 16.1 Hz, $^{2}J_{C(2)P}$ = 6.5 Hz, 2-glyceryl phosphate); 68.7 ppm (*dd*, $^{1}J_{C(2)H}$ = 21.7 Hz, $^{3}J_{C(2)P}$ = 6.9 Hz, 1-glyceryl phosphate); 63.2 ppm (*td*, $^{1}J_{C(1)H}$ = 23.0 Hz, $^{2}J_{C(1)P}$ = 5.0 Hz, 1-glyceryl phosphate), 59.5 ppm (*td*, $^{1}J_{C(1)H}$ = 23.5 Hz, $^{3}J_{C(1)P}$ = 4.2 Hz, 2-glyceryl phosphate). **B** – 163-155 ppm zoom on ^{13}C NMR, **upper spectrum**: in D_2O; **lower spectrum** – in DMSO-*d*6. Leftmost: unreacted urea, central: carbamates, right-hand: cyclic carbonates.

5. Kinetic study of urea-assisted phosphorylation reactions

5.1. Phosphorylation of glycerol (5)

5.1.1. Thermal stability of urea in solution

The thermal degradation of urea was spectroscopically best observed when natural isotope-abundance urea (**2a**) was replaced in part with pure [$^{15}N_2$]urea (**2b**, Supplementary **Fig. 37**, Supplementary **Fig. 38**) in order to follow heated solutions of equimolar mixtures by $^{13}C\{^1H\}$ NMR spectroscopy (Supplementary **Fig. 39**, Supplementary **Fig. 41**).

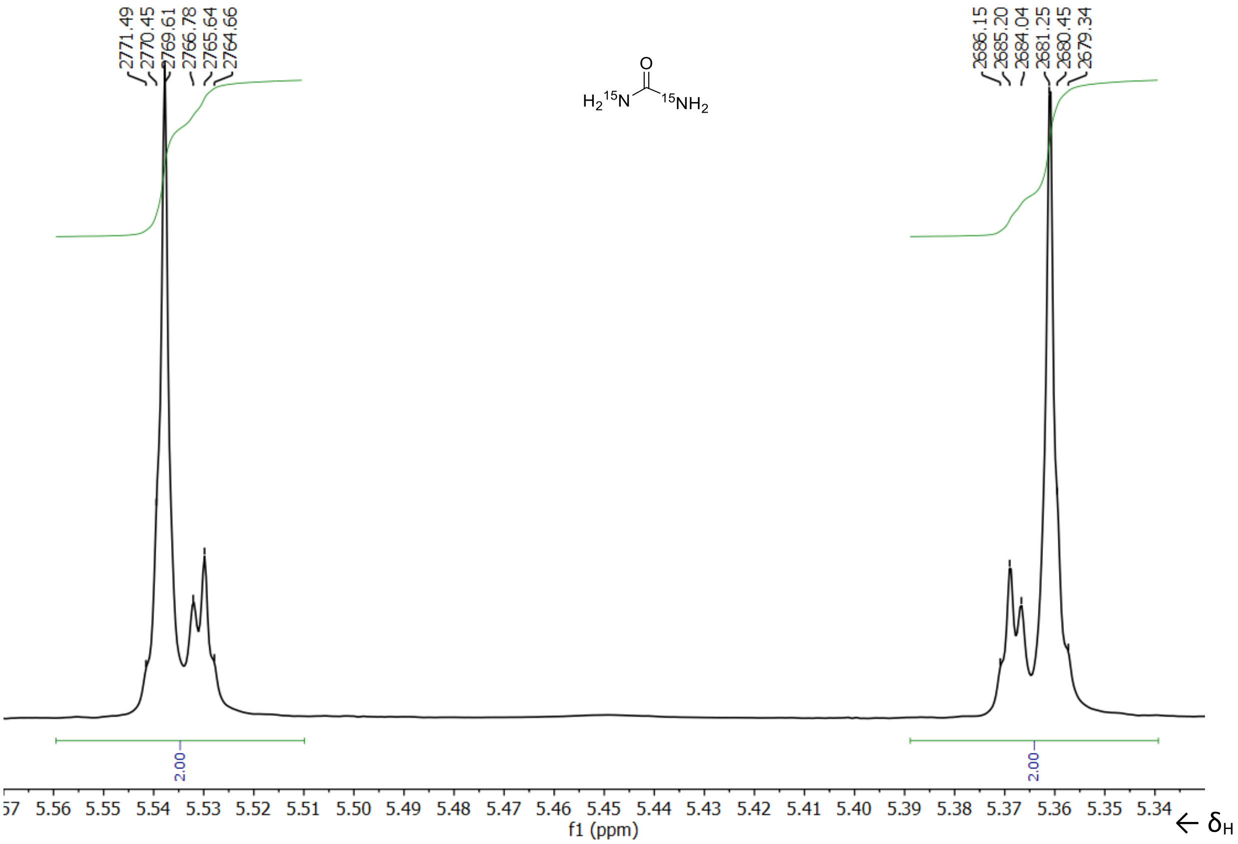

Supplementary Fig. 37. ^{1}H NMR spectrum (500 MHz, DMSO-*d*6) of 0.5 mM [$^{15}N_2$]urea **2b** (500 MHz, DMSO-*d*6), δ_H = 5.45 (*d*, $^1J_{H,N}$ = 88.5 Hz and long-range secondary-order $^3J_{H,N}$ couplings).

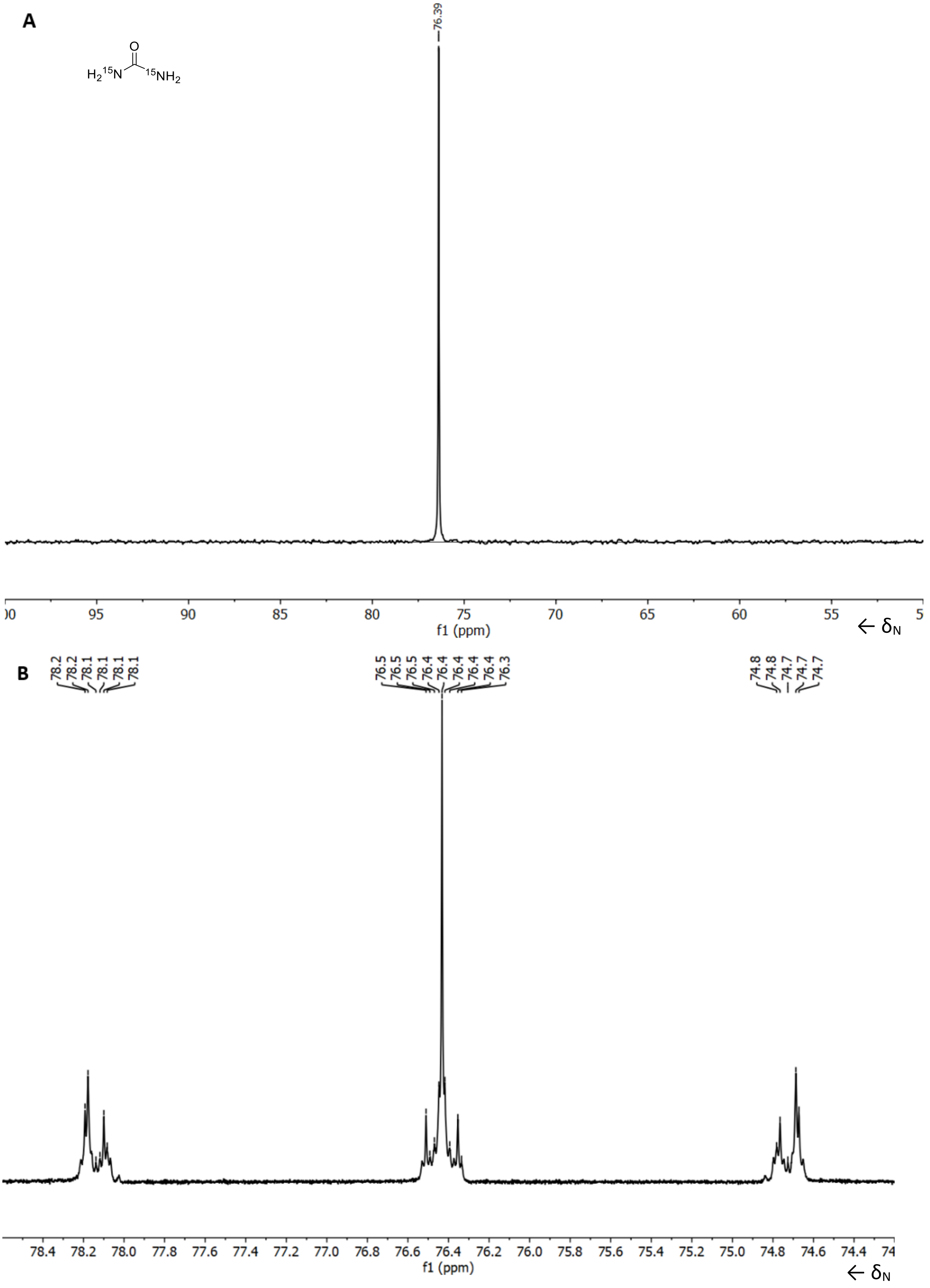

(figure continued on the next page)

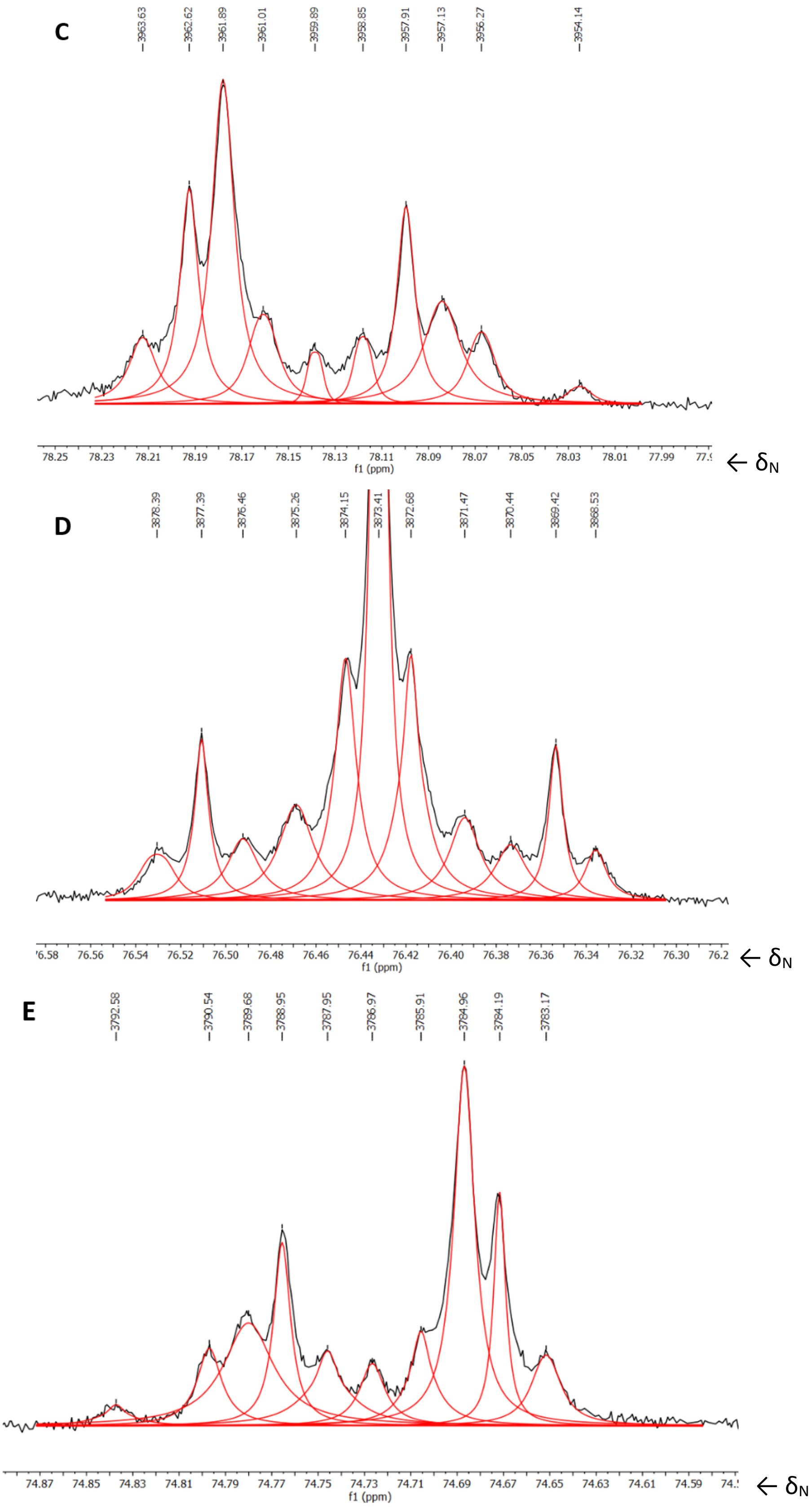

Supplementary Fig. 38. ^{15}N NMR spectrum (50.7 MHz, DMSO-*d6*) of 0.5 mM **2b. A** – Proton decoupled ^{15}N{^{1}H} NMR spectrum at full spectral width: δ_N = 76.39 ppm; **B**-**E** – Proton-coupled ^{15}N NMR spectrum (zoom); **C**-**E** – left-hand, middle and right-hand excerpts of **B** (black: spectrum, red: deconvoluted peaks): δ_N = 76.39 ppm (*t*, $^{1}J_{H,N}$ = 88.5 Hz and long-range secondary-order couplings with $^{3}J_{H,N}$ = 4.0 Hz and smaller). Deconvolution of multiplet was performed by MestreNova™ (version: 3.0.2.8161).

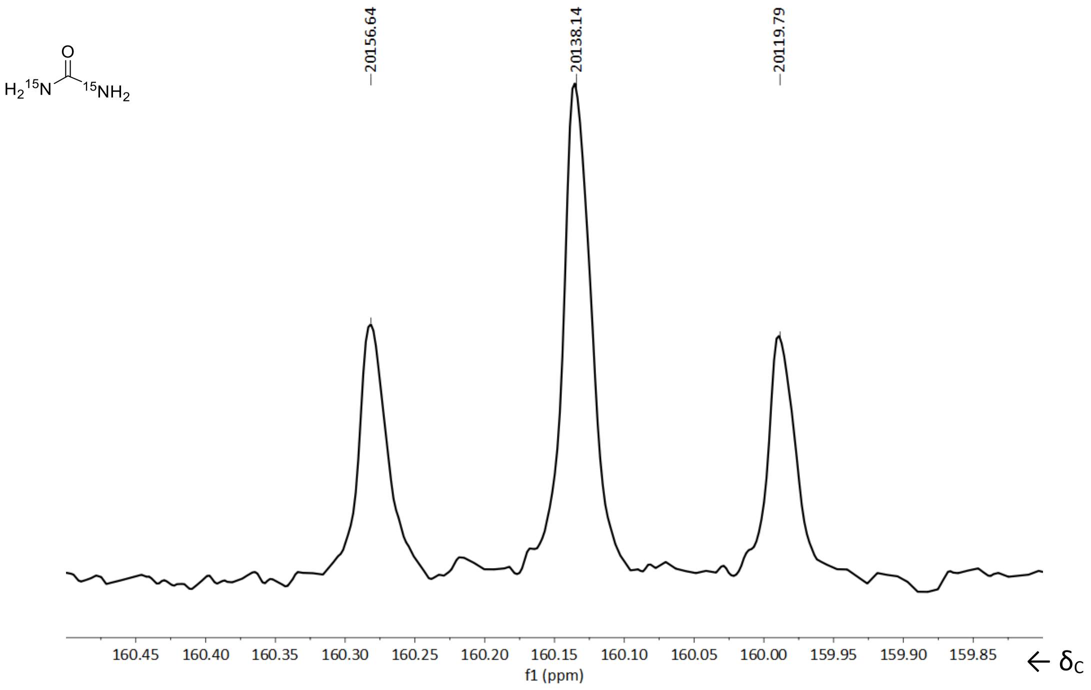

Supplementary Fig. 39. ^{13}C NMR spectrum (125.7 MHz, DMSO-*d6*) 0.5 mM [$^{15}N_2$]urea (**2b**), δ_C = 160.13 ppm (*t*, $^1J_{C,N}$ = 18.6 Hz).

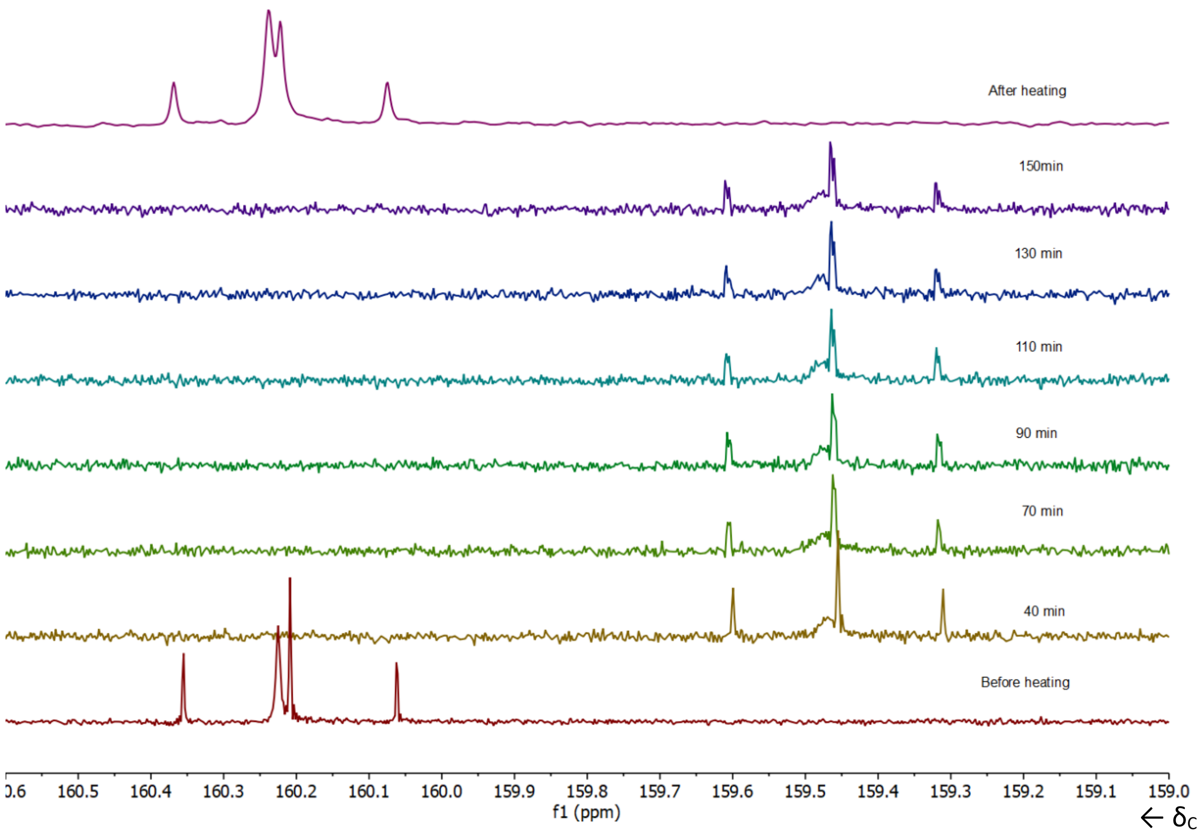

Supplementary Fig. 40. Stack of ^{13}C NMR spectra (500 MHz, DMSO-*d6*) of the equimolar mixture of natural isotope-abundance urea (**2a**) and [$^{15}N_2$]urea (**2b**) (1:1, 0.5 mmol each). The sample was heated up to 100 °C inside the NMR spectrometer and the NMR measurements were performed every 20-30 min. NMR spectra "before" and "after" (Supplementary **Fig. 41**) were made before heating and after cooling down, respectively. After 150 min of heating no significant signals of a doublet were observed.

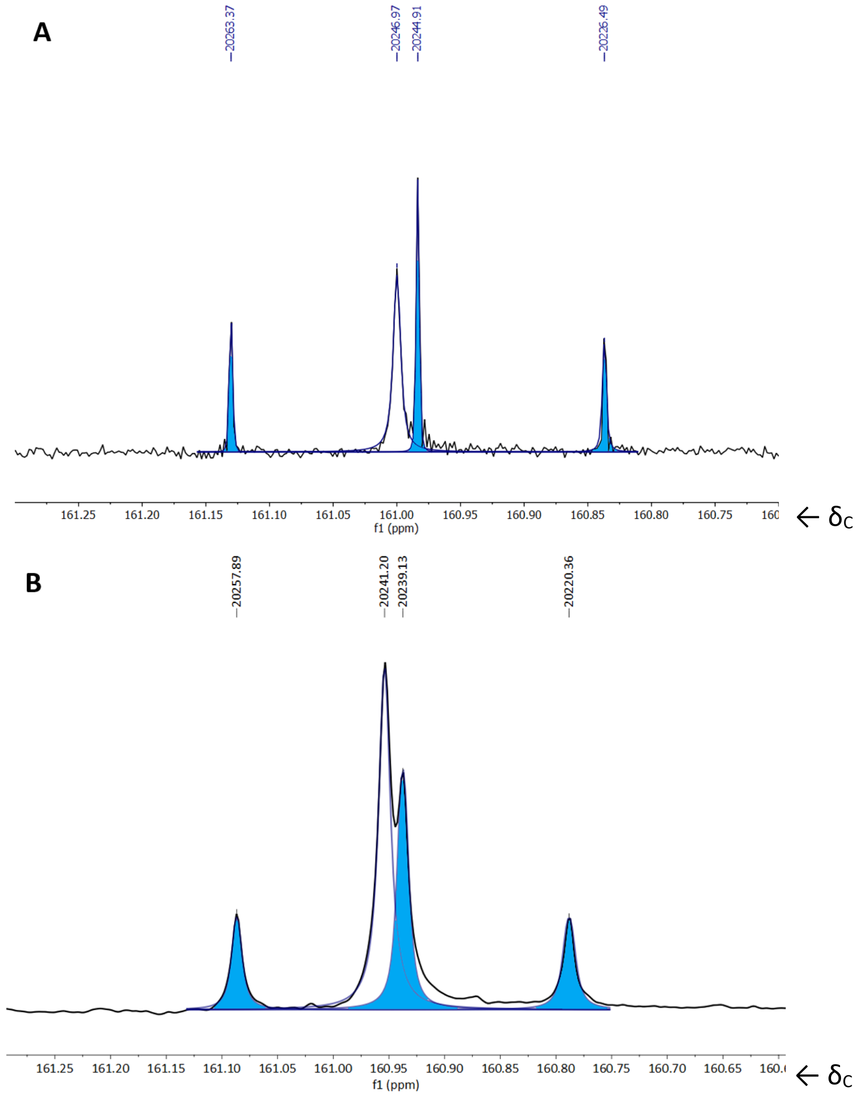

Supplementary Fig. 41. ^{13}C NMR spectra (125.7 MHz, DMSO-*d*6) of the equimolar mixture of natural isotope-abundance urea (**2a**) and [$^{15}N_2$]urea (**2b**) (1:1, 0.5 mmol each) taken both at rt: δ_C = 160.94 ppm (*t*, $^1J_{C,N}$ = 18.6 Hz, **2b**); δ_C = 160.95 ppm (*s*, **2a**). Difference in frequencies (2.07 Hz) between **2a** and **2b** is due to the isotope effect for two ^{15}N nuclei versus two ^{14}N nuclei bonded to ^{13}C. **A** – before heating; **B** – after heating for 150 min up to 100 °C. Black line: spectrum, blue lines and cyan coloured peaks from deconvolution.

Supplementary Table 8. Results from integration of ^{13}C NMR peaks of the deconvoluted urea signals of the spectra shown in Supplementary **Fig. 41** (triplet versus singlet at [160.7-160.3 ppm]) before and after heating a DMSO-*d*6 solution of the equimolar mixture of natural isotope-abundance urea (**2a**) and [$^{15}N_2$]urea (**2b**) (1:1, 0.5 mmol each).

	Ratio of triplet versus singlet before heating, %	Ratio of triplet versus singlet after heating, %
**Triplet**	51.4	52.6
**Singlet**	48.6	47.4

Simply heating a DMSO-*d*6 solution containing the 1:1 mixture of **2a** and **2b** to 100 °C for two hours (Supplementary **Fig. 40**) did not significantly change the amounts of unlabelled and labelled urea (Supplementary **Table 8**), nor did any other signal appear in this timeframe. This meant that any change that would be observed in the presence of other compounds (**P**$_i$, glycerol) could be ascribed to their chemical potential.

5.1.2. Glycerol phosphorylation mixtures containing [$^{15}N_2$]urea

The presence of equimolar amounts of urea (**2a**:**2b**), $\mathbf{P_i}$ and glycerol (**5**) in a neat mixture that was heated at 115 °C not only gave clear evidence for the accelerated degradation of urea to ammonium cyanate within the same timeframe. The spectroscopic signature of [^{14}N,^{15}N]urea appeared already very early in the reaction mixture, thus, proved that eliminated ammonia, before evaporating out of the neat mixture, could re-attack isocyanate to form half-labelled urea molecules (Supplementary **Fig. 42**-Supplementary **Fig. 43**). The time-dependent degradation of urea could also be observed by ^{1}H NMR spectroscopy at 5.4-5.7 ppm (Supplementary **Fig. 44A**).

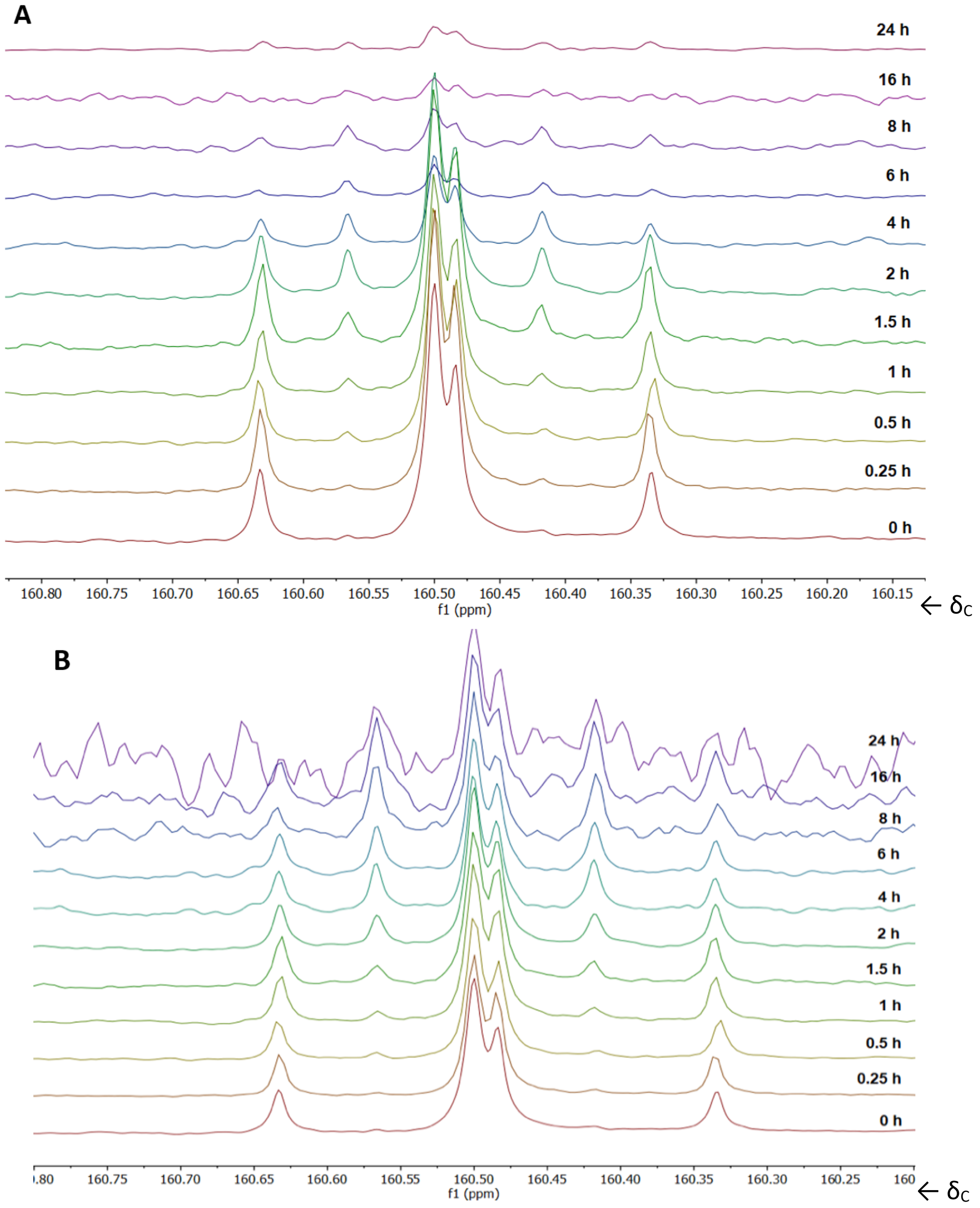

Supplementary Fig. 42. Stack of ^{13}C NMR spectra (125.8 MHz) zoomed on the urea signal of the DMSO-*d*6 extract of a 0.5 mmol scale mixture of glycerol (**5**), natural isotope-abundance urea (**2a**), [$^{15}N_2$]urea (**2b**) and NaH_2PO_4 ($\mathbf{P_i}$) (1:0.5:0.5:1 molar ratios) after heating neat for 0-24 hours at 115 °C. Spectra referenced to the urea singlet at 160.5 ppm. δ_C = 160.50 (*s*, **2a**), 160.48 (*t*, $^{1}J_{C,N}$ = 18.6 Hz, **2b**), 160.49 (*d*, $^{1}J_{C,N}$ = 18.6 Hz, [^{14}N,^{15}N]urea). **A** – Stack of NMR spectra with experimental (absolute) intensities. **B** – Intensities of spectra adjusted to the intensity of the urea singlet at 0 h illustrating (in **B**) the gradually increasing signal-to-noise ratio with time passing owing to the degradation of urea (visible in **A**).

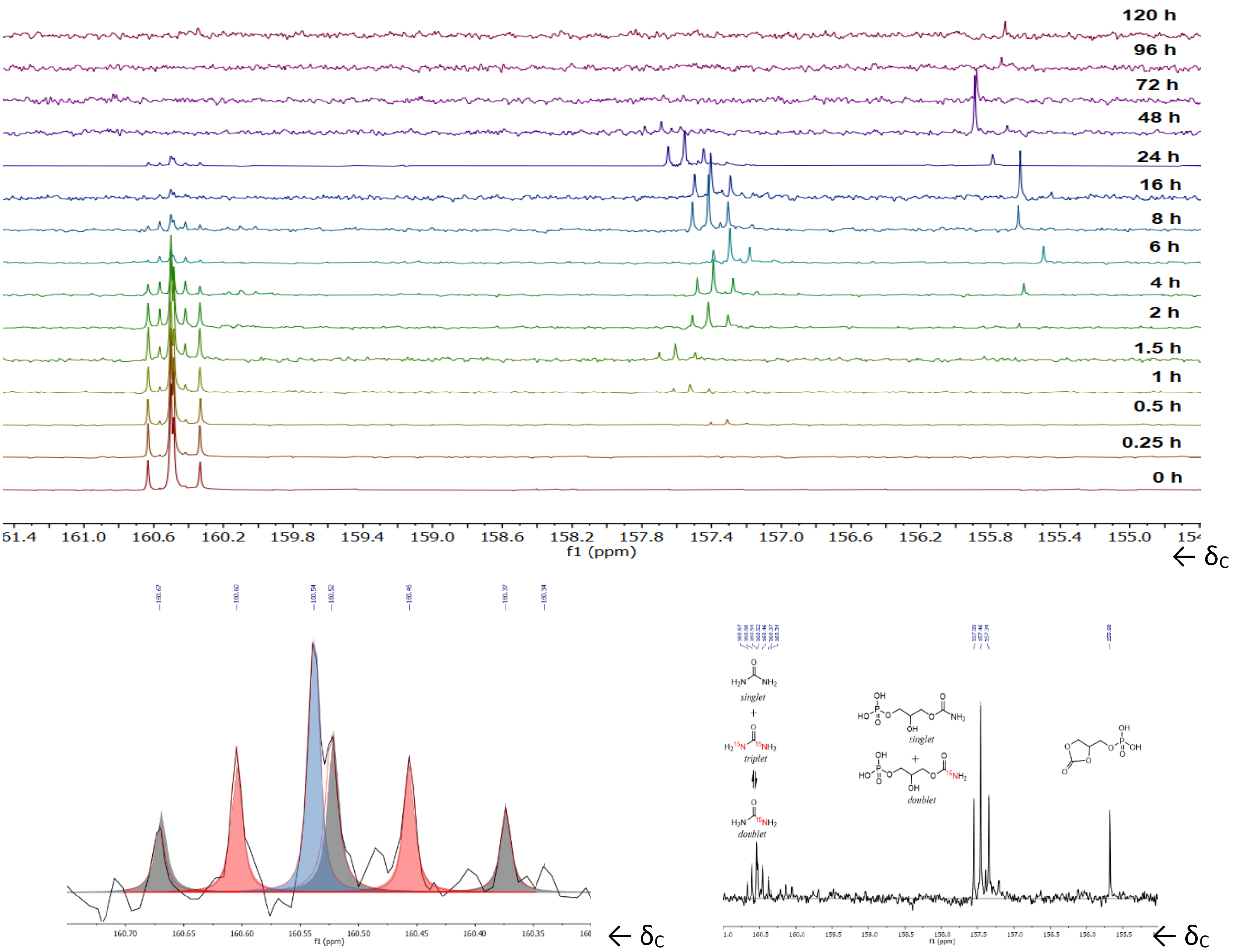

Supplementary Fig. 43. Above: Stack of ^{13}C NMR spectra (125.8 MHz) at the spectral width of carbonyl groups in the DMSO-*d6* extracts of a 0.5 mmol scale mixture of glycerol (**5**), natural isotope-abundance urea (**2a**), [$^{15}N_2$]urea (**2b**) and NaH_2PO_4 (**P**$_i$) (1:0.5:0.5:1 molar ratios) after heating neat for 0-120 hours at 115 °C. Stack referenced to the urea singlet at δ_C = 160.50 ppm. The presence of carbamate and carbonate groups confirms the interpretation of HRMS as shown in Supplementary **Fig. 27**. **Below left:** ^{13}C NMR spectrum of urea in DMSO-*d6* after heating the glycerol mixture for 8 hours. Raw spectrum (black line) overlayed with deconvoluted peaks for [$^{14}N_2$]urea singlet (blue, δ_C = 160.54 ppm), [$^{14}N,^{15}N$]urea doublet (red, δ_C = 160.53 ppm, $^1J_{C,N}$ = 18.6 Hz) and [$^{15}N_2$]urea triplet (grey, δ_C = 160.52 ppm, $^1J_{C,N}$ = 18.6 Hz). Isotope ^{13}C shielding per nitrogen atom $\Delta\delta_C(^{15}N\text{-}^{14}N)$ = 1.10 Hz. **Below right:** Same raw ^{13}C NMR 8 hours-spectrum showing additional signals for carbamate ($\delta_{Csinglet}$ = 157.46 ppm, $\delta_{Cdoublet}$ = 157.45 ppm, $^1J_{C,N}$ = 25.9 Hz) and 1,2-cyclic carbonate (δ_C = 155.68 ppm).

The time-incremental ^{13}C, ^1H and ^{31}P NMR spectra of this reaction mixture (taken from extracts of independent reaction batches for each timepoint) show the evolution of the formation of different classes of organic compounds (Supplementary **Fig. 43**-Supplementary **Fig. 45**). The ^{13}C resonance at $\delta_C \approx$ 157.4 ppm belongs to glyceryl carbamate, since it is a 1:1 mixture of a singlet and a doublet in this mixture containing ^{15}N-half-labelled urea, which is consistent with the ^{13}C NMR spectrum of the mixture that has been labelled with [^{13}C]urea (Supplementary **Fig. 26**). The singlet at > 155 ppm shows emerging glyceryl-1,2-cyclic carbonate. From ^1H-^1H COSY and ^1H-^{31}P HMBC spectra (Supplementary **Fig. 18** and Supplementary **Fig. 20**) we cannot determine with certainty whether the carbamate and 1,2-cyclic carbonate are glycerol or glyceryl phosphate derivatives. However, the rapid appearance of the first ^{31}P signals from the 1-glyceryl and 3-glyceryl phosphates after 15 minutes at 115 °C (Supplementary **Fig. 45**), as well as the equally rapid emergence of a characteristic double ^1H-*dd* system at $\delta_H \approx$ 3.8 and 3.9 ppm (Supplementary **Fig. 44A**) indicate that the major ^{13}C-carbamate and -cyclic carbonate signals are due to the corresponding primary glyceryl phosphate esters rather than glycerol without phosphate.

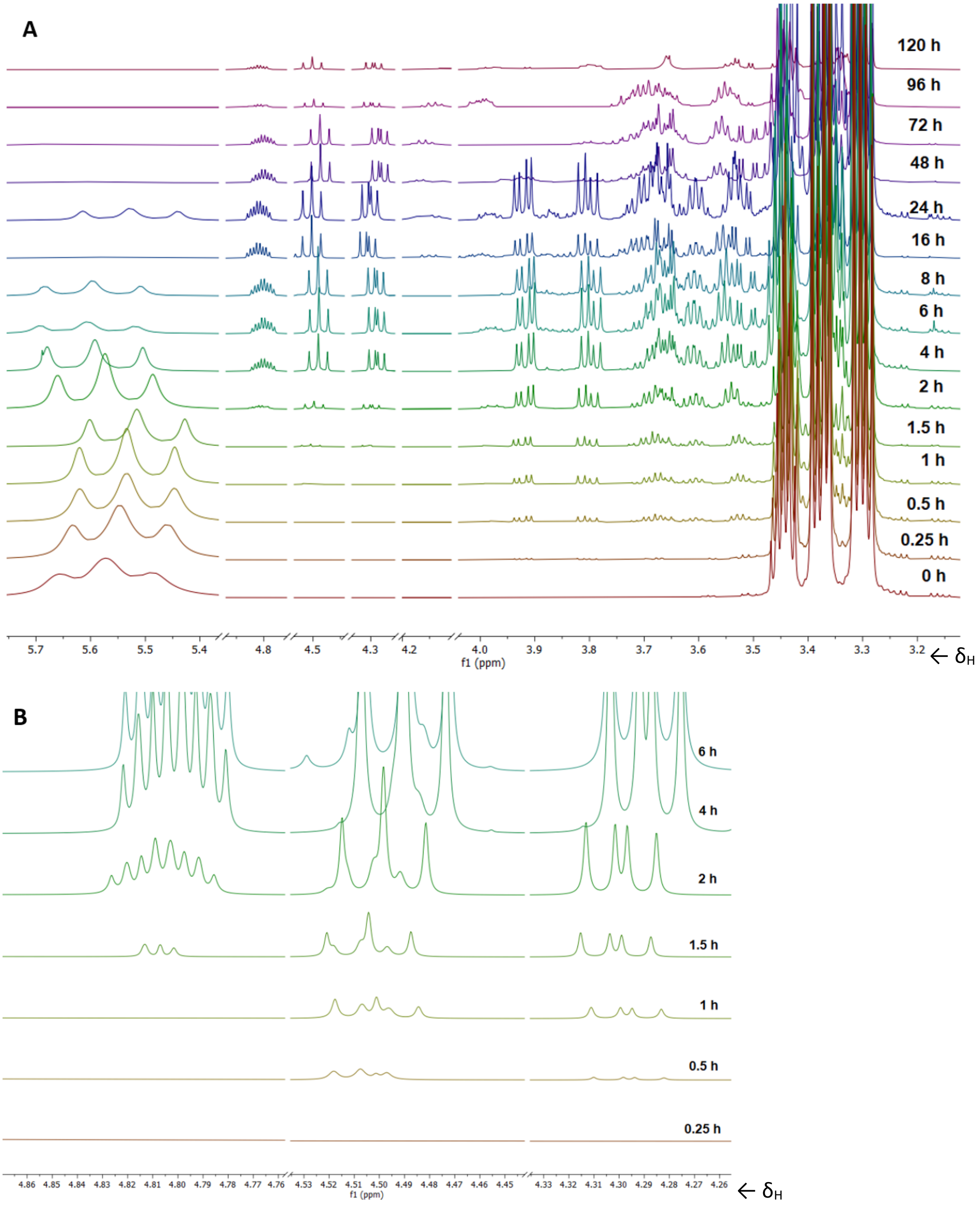

Supplementary Fig. 44. Stack of ^{1}H NMR spectra (500 MHz) of the DMSO-*d6* extract of a 0.5 mmol scale mixture of glycerol (**5**), natural isotope-abundance urea (**2a**), [$^{15}N_2$]urea (**2b**) and NaH_2PO_4 (**P**$_i$) (1:0.5:0.5:1 molar ratios) after heating neat for 0-120 hours at 115 °C. Each time-point was an individual experiment. Spectra referenced and the signal intensities adjusted to the residual *d*5-quintet of DMSO-*d6* at δ_H = 2.52 ppm (not shown). δ_H (2 h) = 5.57 (br *s*, **2a** + carbamate), 5.58 (br *d*, $^{1}J_{H,N}$ = 84 Hz, **2b** + [^{15}N]carbamate), 4.83-4.28 (*ddd*, *t*, *dd*, Hx, Hb and Ha of glyceryl-1,2-cyclic carbonate, Supplementary **Fig. 17**), 4.00-3.20 (mix of glyceryl phosphates, Hb' and Ha' of glyceryl-1,2-cyclic carbonate and glycerol). The decrease of the signal intensities in the late time points are due to the lower solubility of the reaction mixtures. **A** – Spectrum width for all signals except solvent; **B** – Zoom into 4.90-4.25 ppm at 0.25-6 hours.

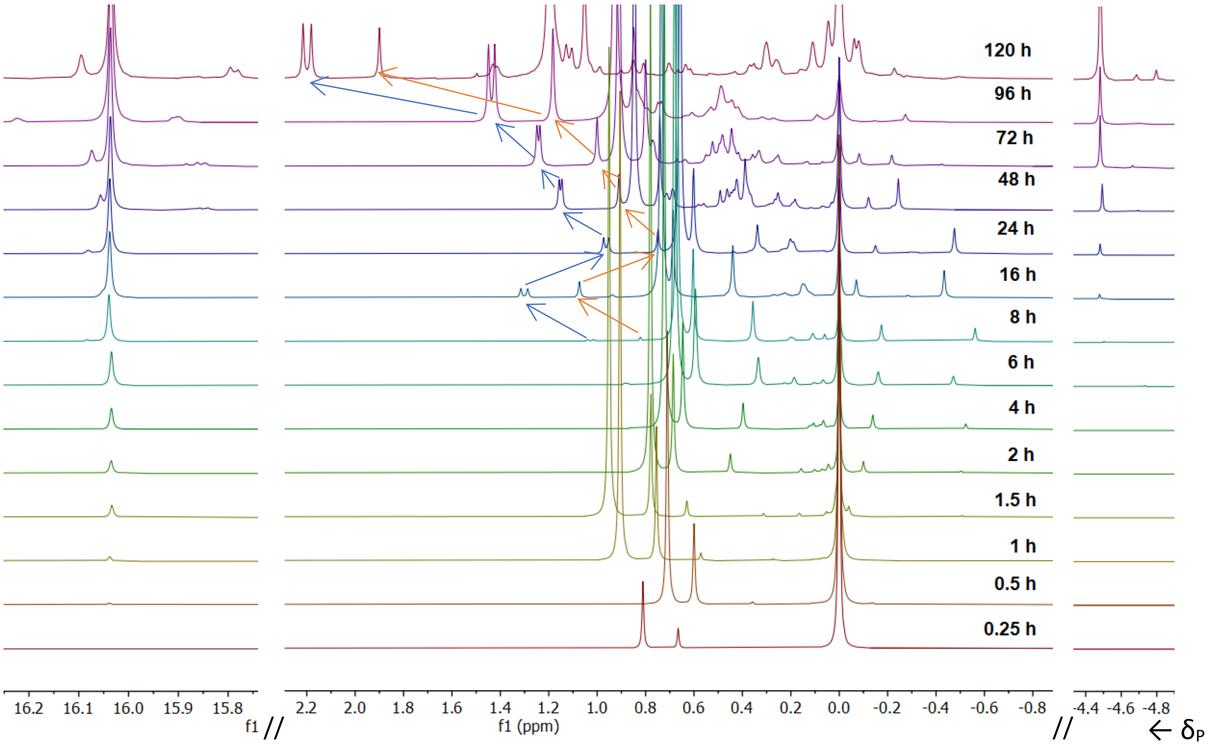

Supplementary Fig. 45. Stack of $^{31}P\{^{1}H\}$ NMR spectra (202.5 MHz) at full spectral width of the DMSO-*d6* extracts of a 0.5 mmol scale mixture of glycerol (**5**), natural isotope-abundance urea (**2a**), $[^{15}N_2]$urea (**2b**) and NaH_2PO_4 (**P**$_i$) (1:0.5:0.5:1 molar ratios) after heating neat for 0-120 hours at 115 °C. Stack referenced to the **P**$_i$ singlet at δ_{Pi} = 0.00 ppm: δ_P = 15.8-16.2 ppm (**5cGlP**), 2.2 to –0.5 ppm (**GlO1P**, **GlO2P**, **[Gl]$_2$P**; cyclic carbonates, cf. blue and orange arrows), –4.4 to –4.7 (**6cGlP**).

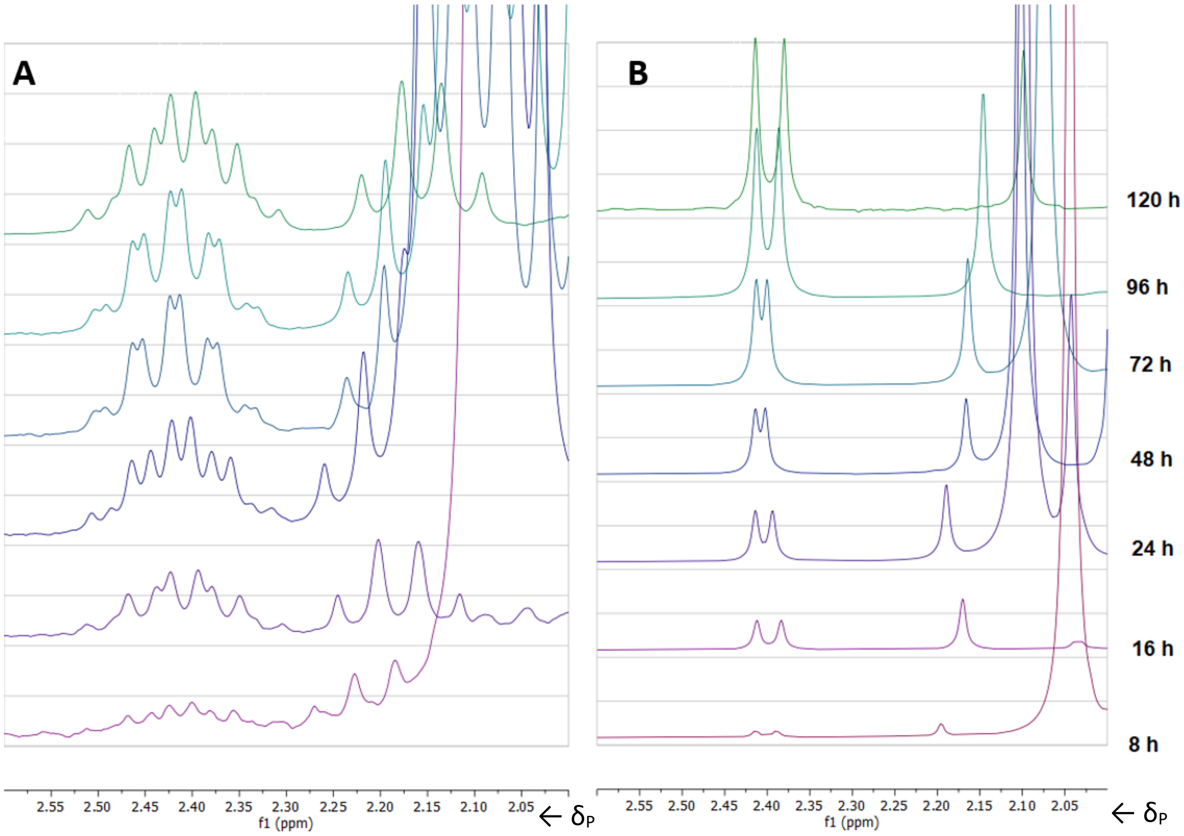

Supplementary Fig. 46. $^{31}P\{^{1}H\}$ NMR (202.5 MHz, DMSO-*d6*) of selected zoom at 2.6-2.0 ppm of Supplementary **Fig. 45**; reaction times 8-120 h, at 115 °C. Stacks centred on the quintet's most downfield maxima at δ_P = 2.42 ppm. **A** – ^{31}P NMR (^{1}H-coupled spectrum); **B** – $^{31}P\{^{1}H\}$ NMR (^{1}H-decoupled spectrum).

Supplementary Table 9. Results from integrating ^{13}C NMR peaks of the deconvoluted urea signals (triplet versus doublet versus singlet at 160.7-160.3 ppm) depending on the heating time (Supplementary **Fig. 42**). DMSO-*d*6 extracts of a 0.5 mmol scale mixture of glycerol (**5**), natural isotope-abundance urea (**2a**), [$^{15}N_2$]urea (**2b**) and NaH_2PO_4 (**P$_i$**) (1:0.5:0.5:1 molar ratios) after heating neat for 0-24 hours at 115 °C. Reactions were conducted in parallel in the carousel reactor (2.1), so each time point is an independent batch. We made a triplicate of each time point reaction. Error margins for all experiments were obtained following the signal-to-noise ratio as calculated by the MNova software, and by applying the Pythagorean theorem[30]. For triplets and doublets, the error margin was calculated from twice the signal-to-noise ratio of the highest peak.

Heating time, h	Triplet [$^{15}N_2$]urea, %	Error margin of triplet, %	Doublet [^{14}N,^{15}N]urea, %	Error margin of doublet, %	Singlet [$^{14}N_2$]urea, %	Error margin of singlet, %
0	49.5	0.66	-	-	50.5	0.35
0.25	52.8	0.75	3.0	1.14	44.2	0.32
0.5	52.8	0.83	5.3	1.16	41.9	0.32
1	52.3	1.28	7.7	1.78	40.0	0.50
1.5	49.6	1.58	11.1	1.99	39.3	0.69
2	47.1	1.26	14.6	1.26	38.3	0.48
4	43.5	2.52	25.0	2.63	31.5	0.85
6	40.5	8.11	28.7	6.94	30.8	2.44
8	39.5	8.54	30.6	7.62	30.0	2.88
16	42.1	23.60	27.2	24.70	30.8	8.34
24	44.6	24.90	30.8	29.60	24.6	7.63

Supplementary Table 10. Results from integrating ^{1}H NMR peaks of the deconvoluted 3-phosphoglyceryl-1,2-cyclic carbonate signals depending on the heating time. DMSO-*d*6 extracts of a 0.5 mmol scale mixture of glycerol (**5**), natural isotope-abundance urea (**2a**), [$^{15}N_2$]urea (**2b**) and NaH_2PO_4 (**P$_i$**) (1:0.5:0.5:1 molar ratios) after heating neat for 0-120 hours at 115 °C. The integrated area of the *t* at $\delta_H \approx 4.5$ ppm was multiplied by five to respect five proton signals of the glyceryl-1,2-cyclic carbonate (Hx, Ha, Hb, Ha', Hb', cf. Supplementary **Fig. 17**) and compared to the total integral of all the signals of ^{1}H NMR. In all cases, the S-to-N ratio was negligible and was not considered in further calculations. The first detectable peak appeared after 30 minutes of heating.

Heating time, h	0.5	1	1.5	2	4	6	8	16	24	48	72	96	120
cyclic carbonate (sum of signals), %	0.15	0.38	1.17	1.95	6.07	6.93	8.32	14.42	8.40	15.48	11.05	4.73	16.62

Supplementary Table 11. Results from integrating ^{13}C NMR peaks (Supplementary **Fig. 43**) of the carbonyl signals of urea [160.7-160.3 ppm], 3-phosphoglyceryl-1-carbamate* [157.8-157.4 ppm] and 3-phosphoglyceryl-1,2-cyclic carbonate* [156.5-155.4 ppm] depending on the heating time at 115 °C obtained from DMSO-*d*6 extracts of mixture on a 0.5 mmol scale starting from glycerol (**5**), natural isotope-abundance urea (**2a**), [$^{15}N_2$]urea (**2b**) and NaH_2PO_4 (**P$_i$**) (1:0.5:0.5:1 molar ratios). We calculated relative quantities of each compound by taking as 100 % the absolute integrated area of initial amount of urea before heating.

Heating time, h	Urea (multiplet), %	Error margin of urea, %	Carbamate (singlet and doublet), %	Error margin of carbamate, %	Cyclic carbonate (singlet), %	Error margin of cyclic carbonate, %
0	100	4.02	-	-	-	-
0.25	79.8	30.70	-	-	-	-
0.5	61.6	17.50	3.03	1.80	-	-
1	51.9	12.50	3.74	1.53	-	-
1.5	38.5	7.65	4.88	1.62	0.61	0.29
2	39.1	6.98	14.5	2.01	0.47	0.10
4	19.4	6.20	14.8	2.28	2.15	0.22
6	10.3	6.13	19.4	2.08	3.36	0.21
8	8.15	4.55	21.5	1.95	3.60	0.24
16	-	-	12.5	2.27	3.77	0.23
24	10.6	18.50	10.6	2.06	6.31	0.25
48	-	-	2.86	1.50	4.64	0.19
72	-	-	-	-	2.52	0.26
96	-	-	-	-	0.99	0.28
120	-	-	-	-	0.66	0.14

* The presence of the phosphate group is not proven but kinetically most probable.

Supplementary Table 12. Results from integrating $^{31}P\{^{1}H\}$ NMR peaks depending on the heating time at 115 °C for Supplementary **Fig. 45**. The total conversion (conversion of initial amount of **P$_i$** to phosphorylated organic products) was determined by subtraction of inorganic compound quantities (**P$_i$** and **PP$_i$**) from the sum of all integrated peak areas. The error margin was calculated from signal-to-noise ratio of the **P$_i$** peak. **GlP** comprise all acyclic organic phosphate peaks except those for **diGl(CO)$_2$P** and **diGlCOP** (Supplementary **Fig. 25**).

Heating time, h	GlP %	diGl(CO)$_2$P %	diGlCOP %	5cGlP %	6cGlP %	P$_i$ %	PP$_i$ %	Total P$_i$ conversion %	Error margin of P$_i$, %
0	-	-	-	-	-	100.00	-	-	0.023
0.25	15.47	-	-	-	-	84.53	-	15.47	0.020
0.5	43.90	-	-	0.16	-	55.94	-	44.06	0.019
1	58.51	-	-	0.34	-	41.15	-	58.85	0.019
1.5	72.09	-	-	1.18	-	26.69	0.04	73.27	0.017
2	82.12	-	-	1.45	-	16.31	0.12	83.57	0.009
4	86.14	0.26	-	2.61	0.16	10.79	0.04	89.17	0.008
6	83.01	0.65	-	2.82	0.14	13.31	0.07	86.62	0.008
8	84.11	0.67	0.78	3.33	0.25	10.76	0.10	89.14	0.007
16	80.36	1.91	1.79	6.20	0.54	8.29	0.91	90.80	0.015
24	77.31	3.36	3.10	3.91	0.07	12.14	0.11	87.75	0.019
48	71.75	3.98	3.03	6.81	1.86	11.67	0.90	87.43	0.007
72	70.36	4.92	3.69	9.25	3.41	7.05	1.32	91.63	0.010
96	66.16	8.70	5.67	9.85	3.35	5.27	1.00	93.73	0.019
120	61.41	3.91	1.85	8.25	8.19	16.23	0.16	83.61	0.035

Supplementary Table 13. Deviations of relative ^{31}P chemical shift $\Delta\delta_P$ and ^{31}P-^{1}H coupling constant (*quint* and *t*) of two compounds persistent over time: columns 4-7 for **diGl(CO)₂P** and columns 8-10 for **diGlCOP**, depending on the reaction scale and the presence of enriched stable isotopes in the starting urea molecule (columns 1-3), cf. **Section 4.1.1**. Conversion of initial amount of $\mathbf{P_i}$ to phosphorylated organic products (denoted $^{31}P\{^{1}H\}$ peak area %) was determined after subtraction of inorganic compound quantities ($\mathbf{P_i}$ and $\mathbf{PP_i}$) from the sum of all integrated peak areas. NIA = natural isotope abundance. Average deviations within peaks of a quintet or triplet.

Urea	Reaction scale, mmol	Reaction time, h	2 x *s* $^{31}P\{^{1}H\}$ area %	$\Delta\delta_P$ between both *s*, Hz	Average ^{31}P-^{1}H coupl. const. of 2x *quint*, Hz	Average deviation of coupl. const. of *quint*, Hz	*s* $^{31}P\{^{1}H\}$ area %	Average ^{31}P-^{1}H coupl. const. of *(d)t*, Hz	Average deviation of coupl. const. of *(d)t*, Hz
$^{14}N/^{15}N$ 1:1	0.5	0	-	-	-	-	-	-	-
	0.5	0.25	-	-	-	-	-	-	-
	0.5	0.5	-	-	-	-	-	-	-
	0.5	1	-	-	-	-	-	-	-
	0.5	1.5	-	-	-	-	-	-	-
	0.5	2	-	-	-	-	-	-	-
	0.5	4	0.26	4.44	-	-	-	-	-
	0.5	6	0.65	2.72	-	-	-	-	-
	0.5	8	0.67	4.94	8.97	0.346	0.78	8.67	-
	0.5	16	1.91	5.83	9.00	0.091	1.79	8.84	0.070
	0.5	24	3.36	4.12	8.63	0.065	3.10	8.42	-
	0.5	48	3.98	2.40	8.11	0.055	3.03	8.15	-
	0.5	72	4.92	2.69	8.17	0.072	3.69	8.09	-
	0.5	96	8.70	5.19	9.01	0.096	5.67	8.67	0.045
	0.5	120	3.91	6.93	9.30	0.228	1.85	9.10	0.049
NIA	0.5	120	7.26	9.38	-	-	4.67	-	-
^{13}C	3	120	6.24	8.04	10.02	0.409	4.14	10.53	0.160
NIA	3	120	3.89	8.20	9.57	0.138	2.24	9.41	0.060
NIA	10	120	12.14	9.61	10.04	0.183	6.11	9.26	0.553
NIA	30	120	10.97	9.85	10.33	0.023	6.96	9.20	0.705

Supplementary Table 14. List with the descriptions of the models of OriginPro™ software (version 9.9.0.225) used for kinetic studies of glycerol and MPG phosphorylation with urea assistance: *x* = heating time [seconds] ; *y* = relative $^{31}P\{^1H\}$,$^{13}C\{^1H\}$ or ^1H NMR peak area [%]. All y values are depicted on the fitted curves by using error margin weights implemented in the software.

Model	MnMolecular	ExpDec2	ExpGrowDec
**Application**	P_i consumption, production of [^{14}N,^{15}N]urea	Total urea decay	3-phosphoglyceryl-1-carbamate and 3-phosphoglyceryl-1,2-cyclic carbonate growth and decay
**Function**	$y = A(1 - e^{-kx})$	$y = A_1 e^{-x/t_1} + A_2 e^{-x/t_2}$	$y = \begin{cases} A_d + A_g\left(e^{\frac{-x_c}{t_g}} - e^{\frac{-x_c}{t_g}}\right) & x \leq x_c \\ A_d e^{\frac{-(x-x_c)}{t_d}} & x > x_c \end{cases}$
**Description**	Exponential function for monomolecular growth model	Double-exponential decay function with time constant parameters	Exponential functions bearing one growth and one decay step
**Optimised parameters found by error margin(*y*)-weighted fitting (Supplementary Table 9)**	*A* = amplitude of growth; *k* = apparent growth rate constant; $t_{1/2}$ = half-time growth rate	A_n = amplitude of decay; t_n = time constant; $t_{1/2\,n}$ = half-time of decay; k_n = decay rate constant.	x_c = center ("knee" in the curve); A_g (A_d) = amplitude of growth (decay); t_g (t_d) = growth (decay) time constant; k_g (k_d) = growth (decay) rate constant.
**Derived parameters***	$y_{max} = A$<br>$t_{1/2} = \frac{1}{k}$	$y_{max} = A_1 + A_2$<br>$t_{1/2_n} = t_n * \ln(2)$<br>$k_n = \frac{1}{t_n}$	$y_{max} = A_g$<br>$k_d = \frac{1}{t_d}$

* The ± values reported by Origin™ for the derived parameters *k* and $t_{1/2}$ (Fig. 4, Supplementary **Table 15**, Supplementary **Table 23**, Section 5.4) represent their standard deviations calculated from the covariance matrix during nonlinear least squares fitting, based on how sensitive the model is to changes in the data and indicate how precisely the estimates point to the original data points. See more details at https://www.originlab.com/doc/Origin-Help/NLFit-Theory#How_Origin_Fits_the_Curve

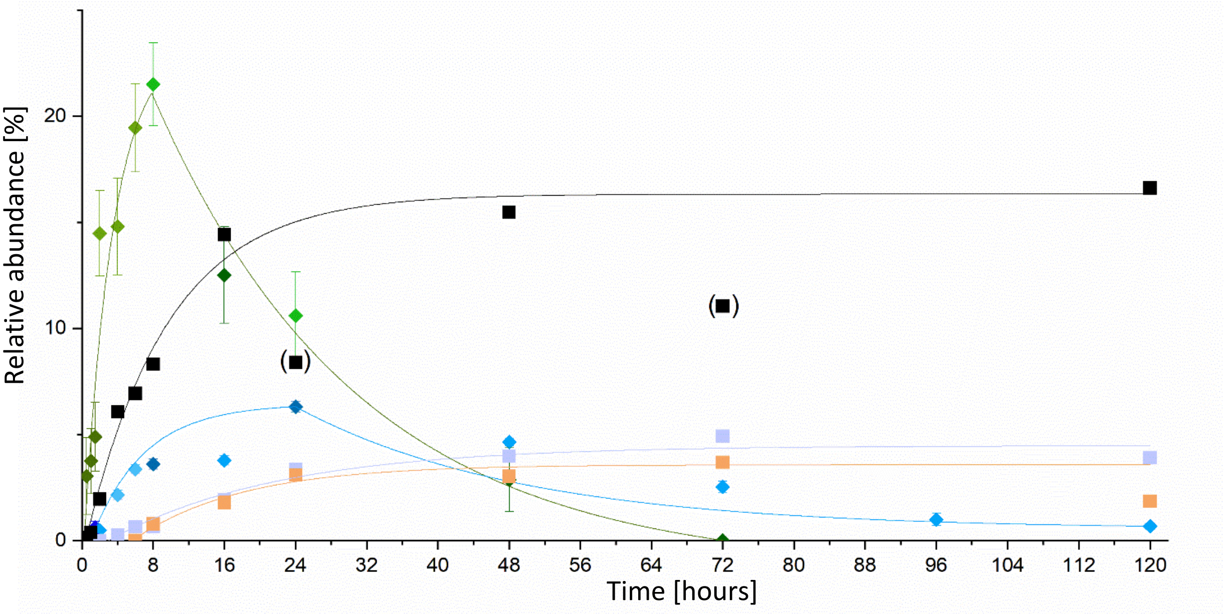

Supplementary Fig. 47. Results of kinetic study of the urea-assisted phosphorylation of glycerol (**5**). Curves show the kinetics of carbamoylation of glyceryl phosphate to: 3-phosphoglyceryl-1-carbamate (green ◆ from ^{13}C NMR, Supplementary **Table 11**), 3-phosphoglyceryl-1,2-cyclic carbonate (black ■ from ^{1}H NMR, Supplementary **Table 10**; blue ◆ from ^{13}C NMR, Supplementary **Table 11**) followed by phosphodiester formation to **diGl(CO)$_2$P** and **diGlCOP** (**pale violet** ■ and, respectively, orange ■ from ^{31}P NMR, Supplementary **Table 12**). Information used for fitting results taken from three series of independent experiments. Exponential fittings all weighted by error margins (bars shown) calculated from S-to-N ratios. In parentheses are outlier values that were not used for fitting.

Supplementary Table 15. Data obtained from kinetic studies of glycerol phosphorylation with urea assistance. Fittings performed by OriginPro™ software (version 9.9.0.225) using data from Supplementary **Table 9**-Supplementary **Table 12**. Fitted parameters: apparent kinetic rate constant ***k***, half-time **$t_{1/2}$** (for growth or decay), squared correlation coefficient **R^2**, and fitting asymptote **% max**.

Subject	Method of analysis	Model	*k* [h^{-1}]	$t_{1/2}$ [h]	R^2	% max
**P_i consumption**	$^{31}P\{^1H\}$ NMR	Growth	1.20 ± 0.10	0.57 ± 0.04	0.98	89.1
**[^{14}N,^{15}N]urea**	$^{13}C\{^1H\}$ NMR	Growth	0.27 ± 0.05	2.60 ± 0.40	0.99	35.8
**Urea decay**	$^{13}C\{^1H\}$ NMR	1st step decay	2.90 ± 2.40	0.24 ± 0.14	0.99	100*
		2nd step decay	0.28 ± 0.03	2.50 ± 0.03		
**Carbamate**	$^{13}C\{^1H\}$ NMR	Growth	0.30 ± 0.02	2.30 ± 1.60	0.97	21.5
		Decay	0.04 ± 0.02	16.00 ± 6.10		
	$^{31}P\{^1H\}$ NMR	Growth	0.05 ± 0.01	77.51 ± 16.10	0.96	4.92
**Cyclic carbonate**	$^{13}C\{^1H\}$ NMR	Growth	0.17 ± 0.23	4.10 ± 2.90	0.88	6.3
		Decay	0.04 ± 0.08	17.70 ± 13.80		
	$^{31}P\{^1H\}$ NMR	Growth	0.08 ± 0.02	52.6 ± 12.93	0.97	3.7
	^{1}H NMR	Growth	0.11 ± 0.01	29.63 ± 0.35	0.99	16.3

* In the case of calculating urea's decay, we set the integral of urea's signal to the first time point (0 hours) as the maximum amount in absolute values (100 %).

Supplementary Table 16. Results from integrating $^{31}P\{^1H\}$ NMR spectra depending on a scale of the reaction. DMSO-*d*6 extract of mixture 0.5-30 mmol scale glycerol (**5**), natural isotope-abundance urea (**2a**) and NaH_2PO_4 (**P_i**) (1:1:1). Calculation of the total conversion (conversion of initial amount of **P_i** to phosphorylated organic products) was made by subtraction of inorganic compounds quantities (**P_i** and **PP_i**) from the sum of all integrated peaks. Scales 3-30 mmoles were carried out in an open round-bottom flask (5-50 ml) with magnetic stirring and an oil heat bath; 0.5 mmol scale was performed using the carousel reactor (2.1) with small magnetic stirrers and electric heating.

Reaction scale, mmol	5cGlP, %	6cGlP, %	GlP,§ %	P_i, %	PP_i, %	Total conversion, %
**0.5**	7.14	3.63	81.07	7.82	0.34	91.84
**0.5**	8.25	8.19	67.17	16.23	0.16	83.61
**0.5**	3.99	1.07	76.59	13.8	4.55	81.65
**3***	12.08	10.05	72.58	4.88	0.41	94.71
**3**	10.40	4.57	77.76	6.86	0.41	92.73
**10**	5.42	3.32	89.17	2.09	-	97.91
**30**	4.56	6.79	83.53	5.12	-	94.88

* in this reaction [^{13}C]urea (**2c**) was used.

§ includes **diGl(CO)$_2$P** and **diGlCOP.**

5.2. Phosphorylation of racemic monopalmitoylglycerol (6)

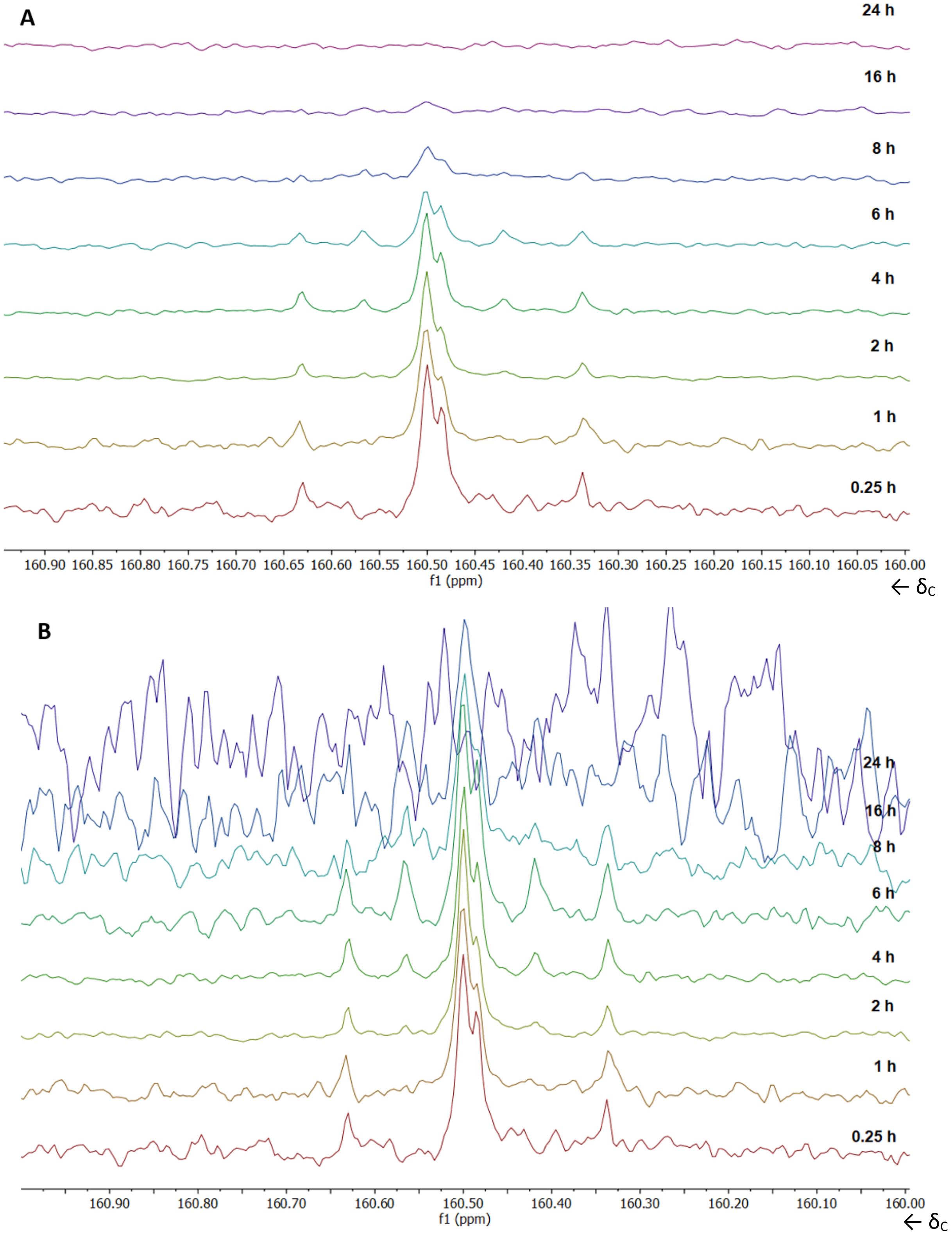

Supplementary Fig. 48. Stack of ^{13}C NMR spectra (125.8 MHz) zoomed on the urea signal of the DMSO-*d6* extract of a 0.5 mmol scale mixture of MPG (**6**), natural isotope-abundance urea (**2a**), [$^{15}N_2$]urea (**2b**) and NaH_2PO_4 (**P**$_i$) (1:0.5:0.5: 1 molar ratios) after heating neat for 0-24 hours at 115 °C. Stack was referenced to the urea singlet at 160.5 ppm: δ_C = 160.50 ppm (*s*, **2a**), 160.48 (*t*, $^1J_{C,N}$ = 18.6 Hz, **2b**), 160.49 (*d*, $^1J_{C,N}$ = 18.6 Hz, [^{14}N,^{15}N]urea). **A** – Stack of NMR spectra with experimental (absolute) intensities. **B** – Intensities of spectra adjusted to the intensity of the urea singlet at 0 h illustrating (in **B**) the gradually increasing signal-to-noise ratio with time passing owing to the degradation of urea (visible in **A**).

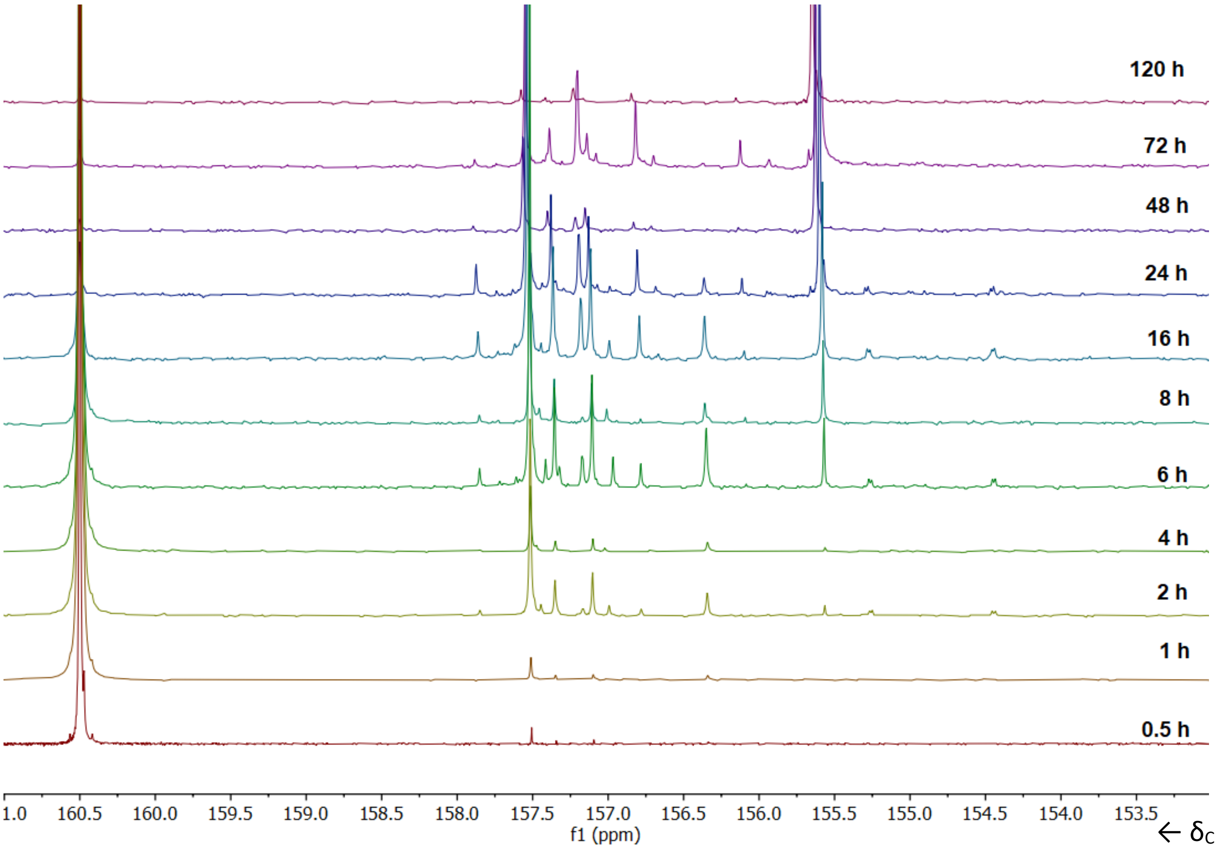

Supplementary Fig. 49. Stack of ^{13}C NMR spectra (125.8 MHz) at full spectral width of the DMSO-*d*6 extracts of a 0.5 mmol scale mixture of MPG (**6**), [^{13}C]urea (**2c**) and NaH_2PO_4 (**P**$_i$) (1:0.5:0.5:1 molar ratios) after heating neat for 0-120 hours at 115 °C: δ_C = urea 160.50 (*s*, **2c**), 157.5 (*s*, MPG carbamate), 157.3, 157.1 (2 x *s*, MPG dicarbamate), 155.6 (*s*, MPG-2,3-cyclic carbonate). Stack referenced to the urea singlet at δ_C = 160.50 ppm. Presence of carbamate and carbonate groups confirms interpretation of HRMS as shown in Supplementary **Fig. 33**.

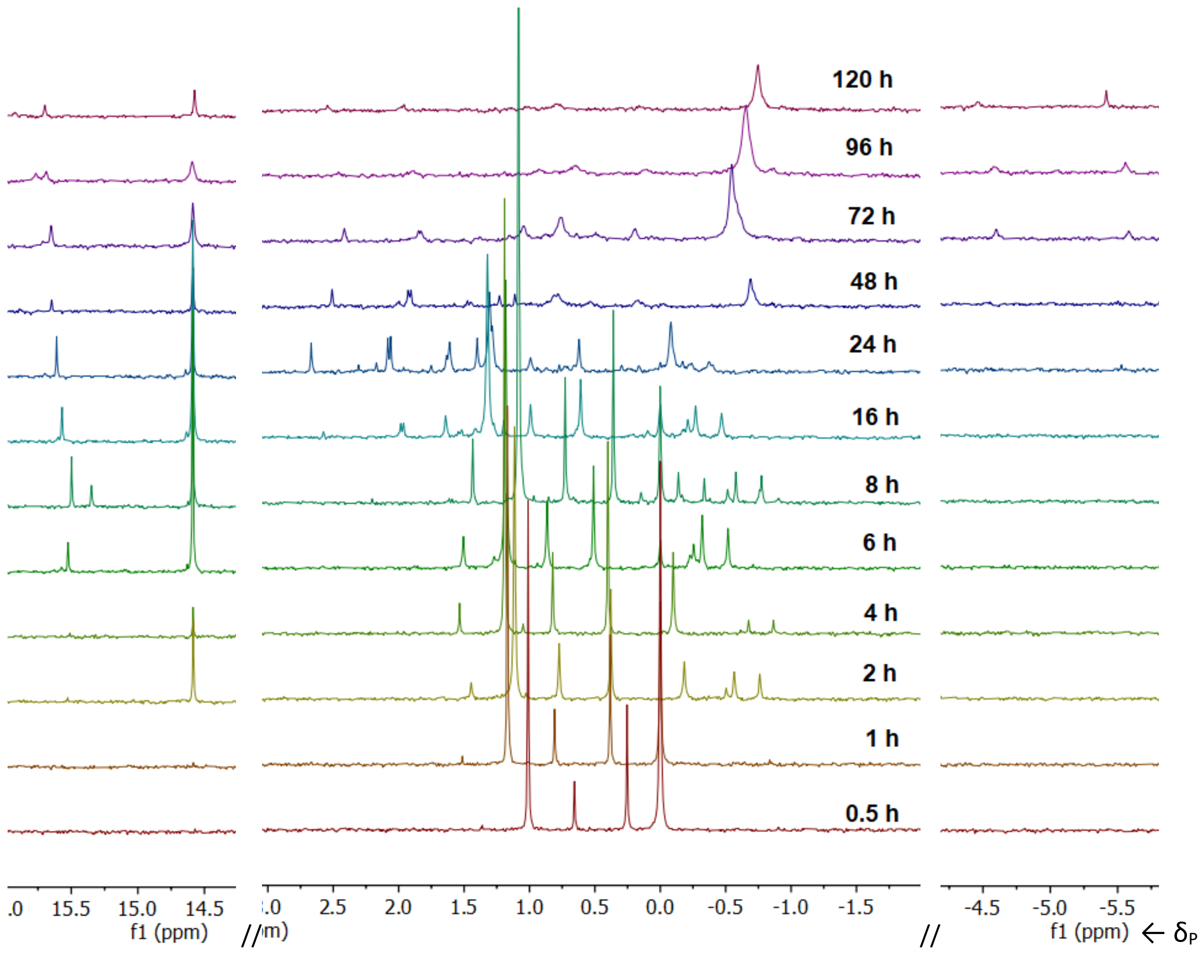

Supplementary Fig. 50. Stack of $^{31}P\{^{1}H\}$ NMR spectra (202.5 MHz) of the DMSO-*d6* extracts of a 0.5 mmol scale mixture of MPG (**6**), natural isotope-abundance urea (**2a**), $[^{15}N_2]$urea (**2b**) and NaH_2PO_4 (**P**$_i$) (1:0.5:0.5:1 molar ratios) after heating neat for 0-120 hours at 115 °C. Stack referenced to the **P**$_i$ singlet at δ_{Pi} = 0.00 ppm: δ_P = 15.5 ppm (**5cGlP**), 14.5 ppm (**5cMPGP**), from 2.5 to –1.0 ppm (**MPGO1P**, **MPGO2P** and **[MPG]$_2$P**), from –4.5 to –5.5 ppm (**6c MPGP**).

For all NMR analyses, we extracted the products from the reaction mixture as described in Materials and Methods 1.3.1. Nevertheless, we observed difficulties with the full dissolution in DMSO-*d6* of the crude mixtures after 24-48h of heating. After the centrifugation step of the sample preparation, the extracts of the late reaction mixtures were subdivided in three phases. The bottom phase was insoluble and remained as white as the initial crude mixture: the solvent was saturated with reaction products (and some part of unreacted **P**$_i$) so a part of the crude mixture remained insoluble. The middle fraction was a transparent solution that contained the extracted products and was used for NMR analysis. The top fraction was transparent but behaved like a gel and complicated the extraction process — the presence of (deacylated) palmitate could be the cause of this effect. Because of the low volume of the fraction available for NMR analysis, signal intensities were low and the signal-to-noise ratio increased in the 48-120 h spectra. To avoid these difficulties, in another series of experiments, we lowered the amount of the crude mixture, which solved the problem.

A kinetic study was also made for two 5-membered ring phosphorylated products of MPG and glycerol. After 16-48 h when urea was mostly consumed, the production of 5-membered ring MPG phosphate slowed down. Over this period of time the amount of **5cMPGP** decreased, which could be explained by facilitation of further reactions because of the presence of **5cMPGP** as a new starting material. During the same time period, the amount of **5cGlP** remained more less the same and its decay was slower than that of **5cMPGP**. The reason may be the facility of glycerol to be phosphorylated without the presence of urea. There are other signals in the 5-membered ring region, however, we do

not have enough data to make a fitting and track their growth and decay. These products could be monocyclic glyceryl diphosphate **PGl>P** and/or mono-cyclic diglyceryl diphosphate **GlPGl>P**. In Supplementary **Table 17**, the values were obtained from the integration of the $^{31}P\{^{1}H\}$ NMR peak areas (Supplementary **Fig. 50**) divided by the maximum phosphorylation of a single product being **5cMPGP** after 16 hours and taken as 100%.

Supplementary Table 17. Results from integrating $^{31}P\{^{1}H\}$ NMR peaks in the 5-membered cyclic phosphate region depending on the heating time. DMSO-*d*6 extracts of MPG (**6**), [^{13}C]urea (**2c**) and NaH_2PO_4 (**P$_i$**) 1:1:1 (0.5 mmol each) heated neat at 115 °C. Values of conversions were calculated with respect to the maximum absolute intensity of **5cMPGP** at 16 h.

**Heating time, h**		**5cGlP, %**		**5cMPGP, %**
	15.6 ppm	15.5 ppm	14.55 ppm	14.5 ppm
**0.5**	-	-	-	-
**1**	-	-	-	1.3
**2**	-	1.3	-	30.7
**4**	-	1.6	-	8.9
**6**	-	10.2	-	96.3
**8**	-	15.2	-	65.8
**16**	-	16.8	6.0	100.0
**24**	2.6	13.9	3.7	50.2
**48**	-	4.8	-	21.3
**72**	7.1	17.5	5.3	39.7
**96**	12.0	11.7	-	35.5
**120**	4.0	6.3	2.4	17.4

Supplementary Table 18. Results from integrating ^{13}C NMR peaks of the deconvoluted urea signals (triplet versus doublet versus singlet at 160.7-160.3 ppm) depending on the heating time (Supplementary **Fig. 49**). DMSO-*d*6 extracts of a 0.5 mmol scale mixture of MPG (**6**), natural isotope-abundance urea (**2a**), [$^{15}N_2$]urea (**2b**) and NaH_2PO_4 (**P$_i$**) (1:0.5:0.5:1 molar ratios) after heating neat for 0-8 hours at 115 °C. After 8 hours of heating the signal/noise ratio of urea signal became too low to identify and deconvolute its multiplicity. Reactions were conducted in parallel in the carousel reactor, so each time point is an independent batch. Error margins for all experiments were obtained following the signal-noise ratio as calculated by the MNova software, and by applying the Pythagorean theorem[30]. For triplets and doublets, the error margin was calculated from twice the signal-to-noise ratio of the highest peak.

**Heating time, h**	**Triplet [$^{15}N_2$]urea %**	**Error margin of triplet, %**	**Doublet [^{14}N,^{15}N]urea, %**	**Error margin of doublet, %**	**Singlet [$^{14}N_2$]urea, %**	**Error margin of singlet, %**
0	49.5	0.66	-	-	50.5	0.35
0.25	53.10	5.10	-	-	46.90	3.19
0.75	44.90	25.81	10.73	17.44	44.37	11.93
1	39.03	5.90	10.46	12.13	50.51	3.16
2	32.90	2.02	9.37	3.98	57.73	1.21
4	38.48	2.70	11.74	3.74	49.78	1.48
6	47.03	5.47	20.57	6.15	32.40	1.95
8	39.25	10.65	17.17	9.23	43.58	5.03

Supplementary Table 19. Results from integrating ^{13}C NMR peak areas of the carbonyl signals of urea [160.5 ppm], 3-phosphoglyceryl-1-carbamate [157.5 ppm], 3-phosphoglyceryl-1,2-cyclic carbonate [155.6 ppm] and glyceryl dicarbamate [157.3-157.1 ppm] depending on the heating time. Spectra from DMSO-*d*6 extracts of MPG (**6**), [^{13}C]urea (**2c**) and NaH_2PO_4 (**P$_i$**) 1:1:1 (0.5 mmol each) after heating neat at 115 °C. We calculated relative quantities of each compound by taking as 100 % the absolute integrated area of initial amount of urea in the beginning of the experiment. Integrations obtained from spectra of Supplementary **Fig. 49**.

Heating time h	Urea (singlet) %	Error margin of urea %	Carbamate (singlet) %	Error margin of carbamate %	Cyclic carbonate (singlet) %	Error margin of cyclic carbonate %	Dicarbamate (singlet) %	Error margin of dicarbamate %
0.5	100.00	0.06	0.62	5.77	-	-	0.14	20.62
1	99.73	0.05	1.13	3.54	-	-	0.30	14.71
4	86.28	0.06	2.49	1.46	0.13	21.19	0.43	7.24
2	73.96	0.07	7.51	0.53	0.26	10.40	1.47	2.46
6	46.15	0.11	12.82	0.28	1.34	2.25	2.61	1.39
8	22.02	0.23	4.68	0.80	1.55	1.98	0.98	3.95
16	11.95	0.47	16.20	0.29	4.33	0.91	3.17	1.50
24	1.51	2.84	9.68	0.31	8.38	0.32	2.00	1.92
48	0.37	13.23	2.34	1.66	5.17	0.68	0.59	6.92
72	0.79	6.00	0.43	0.95	23.67	0.15	1.30	4.81
120	0.40	11.15	0.33	12.59	8.85	0.37	0.23	40.32

Supplementary Table 20. Results from integrating $^{31}P\{^{1}H\}$ NMR peak areas depending on the heating time. Spectra from DMSO-*d*6 extracts of MPG (**6**), [^{13}C]urea (**2c**) and NaH_2PO_4 (**P$_i$**) 1:1:1 (0.5 mmol each) after heating neat at 115 °C. Calculation of total conversion (conversion of initial amount of **P$_i$** to phosphorylated organic products) was made by subtraction of inorganic compound quantities (**P$_i$** and **PP$_i$**) from the sum of all integrated peaks. Error margin was calculated from signal-to-noise ratio of **P$_i$** peak. Integrations obtained from spectra shown in Supplementary **Fig. 50**.

Heating time h	MPGP %	5cMPGP %	6cMPGP %	P$_i$ %	Total conversion %	Error margin of P$_i$ %
0.5	47.54	-	-	52.46	47.54	0.44
1	66.37	0.42	-	33.21	66.79	0.95
2	83.97	8.33	-	7.70	92.30	4.20
4	84.45	2.82	-	12.73	87.27	1.85
6	78.06	18.46	-	3.48	96.52	5.43
8	78.94	12.14	-	8.92	91.08	1.39
16	71.57	22.80	-	5.63	94.37	5.07
24	82.64	14.79	-	2.14	97.86	17.04
48	83.45	13.86	0.65	2.04	97.96	52.36
72	80.42	13.5	3.05	3.03	96.97	16.58
120	78.61	16.68	4.71	-	100.00	-

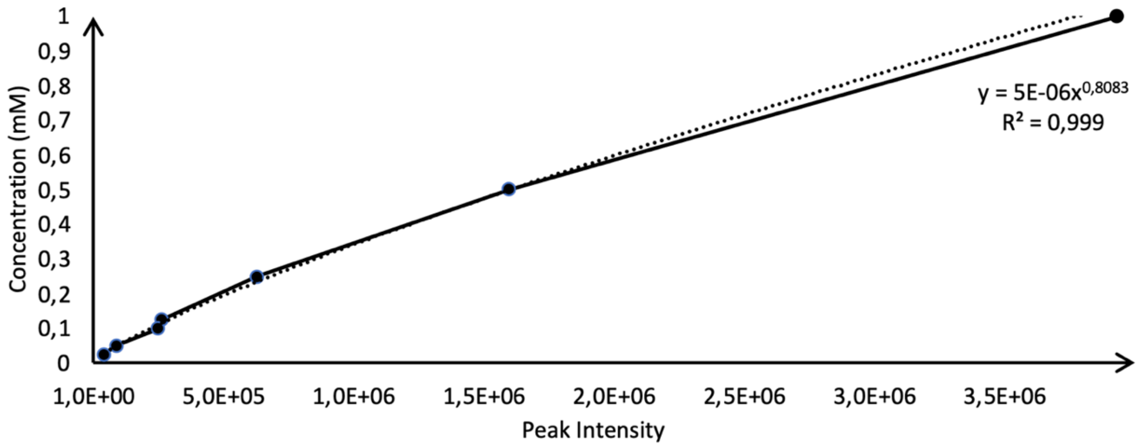

Supplementary Fig. 51. Calibration curve of pure MPG (**6**) for the determination of its concentration depending on the ELSD signal intensity in HPLC reference runs. 3.30 mg of synthesised pure MPG (**6**) was dissolved in 1 ml of MeOH. The solution was diluted in MeOH in order to obtain the appropriate concentrations (from 0.5 mM to 25 µM) and injected. An exponential trendline was used for the fitting and the obtained equation is shown in the figure.

Supplementary Table 21. Intensities of the MPG (**6**) signal by ELSD from a HPLC chromatogram depending on its concentration in MeOH.

Signal intensity (arbitrary units)	Concentration (mM)
3934728	1.00
1596149	0.50
628127	0.25
261111	0.125
249353	0.10
90330	0.05
40632	0.025

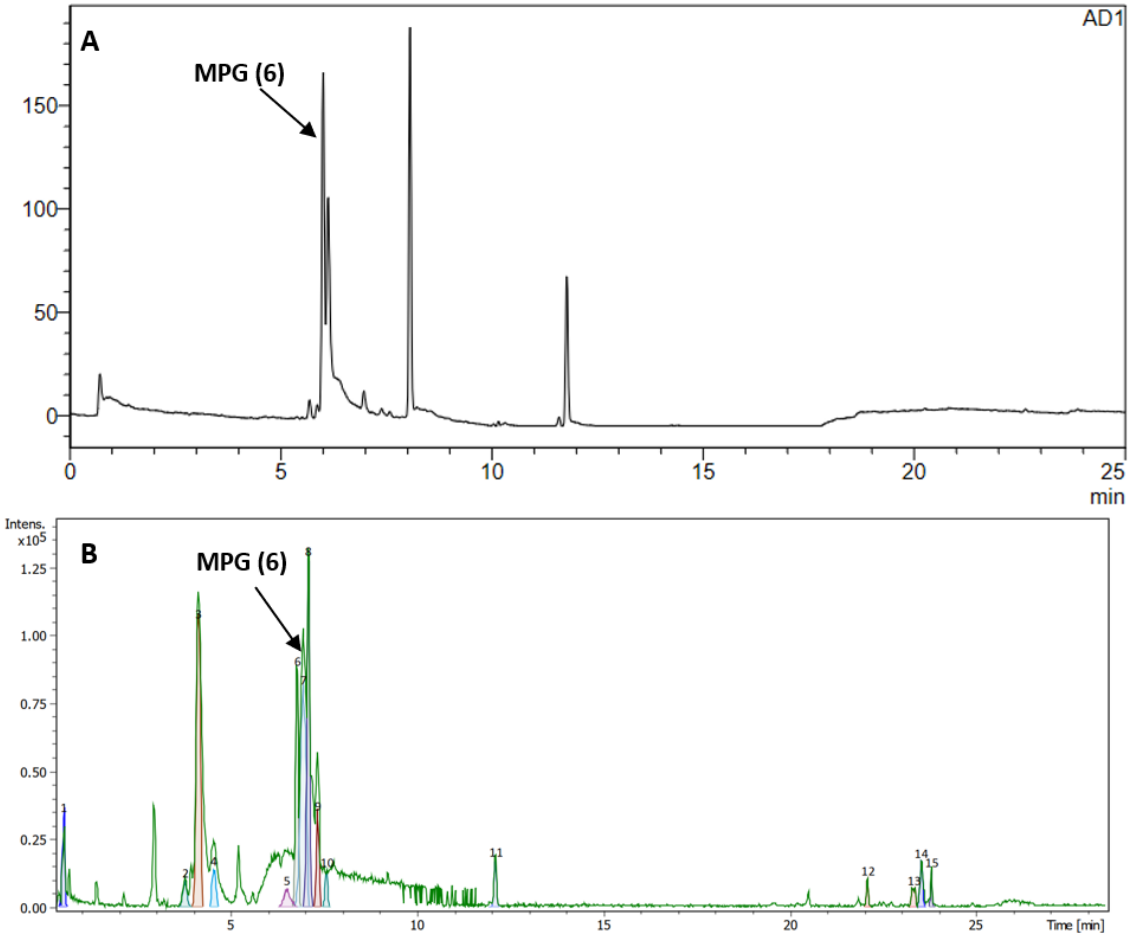

Supplementary Fig. 52. RP-UHPLC-HRMS chromatogram of a MeOH extract of a mixture of MPG (**6**), [^{13}C]urea (**2c**) and NaH_2PO_4 (**P$_i$**) (1:1:1 0;5 mmol each) after heating neat at 115 °C for 48 hours. **A** – ELSD chromatogram. **B** – Negative ion chromatogram (green, peaks slightly delayed with respect to ELSD) with extracted negative ions (coloured, numbered peaks). The chemical structures and exact masses are shown in Supplementary **Fig. 33**.

Supplementary Table 22. Results from integrating HPLC-ELSD peak areas depending on the heating time of crude mixture. MeOH extracts of mixtures of MPG (**6**), [^{13}C]urea (**2c**) and NaH_2PO_4 (**P$_i$**) 1:1:1 (0.5 mmol each) after heating neat for 0-48 hours. Calculation of total conversion (conversion of initial amount of MPG to phosphorylated organic products) was made by subtraction of residual amount of MPG (MPG signal on chromatogram) from the sum of all integrated peaks. Error margin was calculated from the signal-to-noise ratio of the MPG peak.

Heating time, h	MPG consumption, %	Error margin of MPG consumption, %
0	-	-
0.5	22.61	0.0029
1	12.06	0.00278
2	21.12	0.00437
4	37.05	0.00673
6	43.51	0.00477
16	86.36	0.04803
24	76.42	0.02490
48	78.91	0.08197

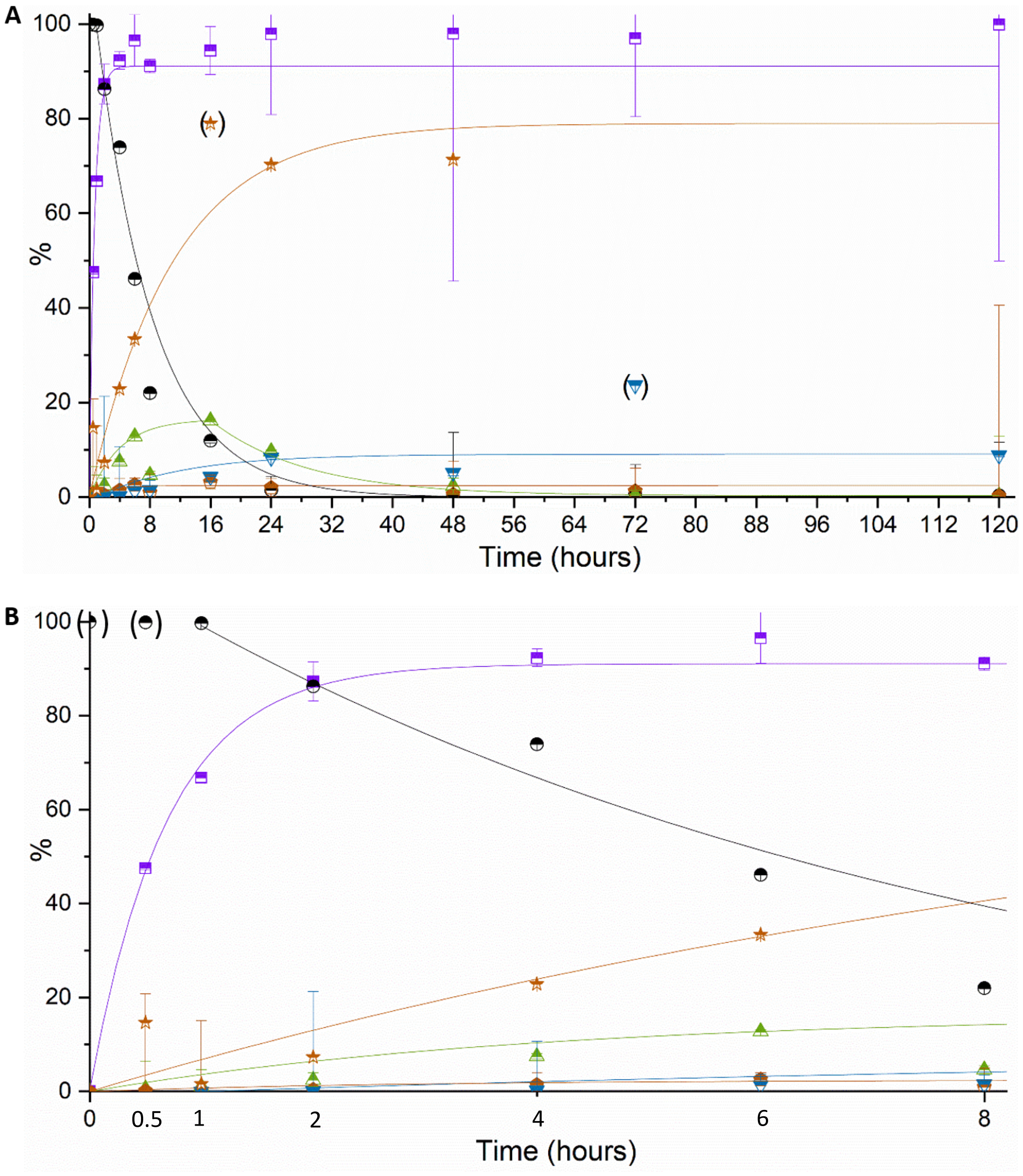

Supplementary Fig. 53. Results of kinetic study of MPG (**6**) phosphorylation through exponential fitting (cf. Supplementary **Table 14**). Curves represent fitted urea decay (black half-filled circles), MPG carbamoylation (mono-carbamate green half-filled triangles and bi-carbamate brown 'diamonds' ⯁), its cyclisation to carbonate (blue inverted half-filled triangles), **P**$_i$ consumption (violet half-filled squares) and MPG consumption (brown half-filled stars) during phosphorylation of MPG. Information used for fitting was obtained from Supplementary **Table 18**-Supplementary **Table 22** as the result of three series of independent experiments. Exponential fittings all weighted by S-to-N margins (bars shown). In parentheses are outlier values that were not used for fittings.

A – Full experimental length 0-120 hours. **B** – 0-8 hours (zoom).

Supplementary Table 23. Fitted parameters (as in Supplementary **Table 15**) from kinetic studies of urea-assisted MPG phosphorylation resulting from fittings shown in Supplementary **Fig. 53**. Fittings performed by OriginPro™ software (version 9.9.0.225) using data from Supplementary **Table 18**-Supplementary **Table 22**. Fitted parameters: apparent kinetic rate constant ***k***, half-time $\mathbf{t_{1/2}}$ (for growth or decay), squared correlation coefficient $\mathbf{R^2}$, and fitting asymptote **% max**.

Subject	Method of analysis	Model	*k* [h^{-1}]	$t_{1/2}$ [h]	R^2	% max
**P_i consumption**	$^{31}P\{^1H\}$ NMR	Growth	1.45 ± 0.04	0.5 ± 0.01	0.99	100*
**MPG consumption**	$^{13}C\{^1H\}$ NMR	Growth	0.11 ± 0.01	6.4 ± 0.5	0.99	76.1
**Urea**	$^{13}C\{^1H\}$ NMR	1st step decay	3.1 §	5.1 ± 0.14	0.94	100*
		2nd step decay	3.6 §	5.5 ± 0.29		
**Carbamate**	$^{13}C\{^1H\}$ NMR	Growth	0.25 ± 0.24	2.8 ± 2.8	0.97	16.2
		Decay	0.08 ± 0.08	8.8 ± 5.8		
**Cyclic carbonate**	$^{13}C\{^1H\}$ NMR	Growth	0.09 ± 0.02	7.9 ± 2.1	0.99	8.8
**Bicarbamate**		Growth	0.36 ± 0.25	1.9 ± 2.4	0.91	3.1

* Fixed (not fitted) parameter
§ The error margin of the fitting was 5 orders of magnitude larger than the *k* value

Supplementary Table 24. Reaction scale dependence of conversion from integrating $^{31}P\{^1H\}$ NMR peak areas. DMSO-*d*6 extracts of mixture on a 0.5-10 mmol scale of MPG (**6**), urea (**2a** or **2b** or **2c**) and NaH_2PO_4 (**P_i** or **$[^{18}O_4]P_i$**) (1:1:1 molar ratios) after 120 h of heating neat at 115°C. Calculation of total conversion (conversion of initial amount of **P_i** to phosphorylated organic products) was made by subtraction of inorganic compound quantities (**P_i**) from the sum of all integrated peaks. Scales 3-10 mmol were carried out in a round-bottom flask (5-50 ml) with magnetic stirring and an oil heat bath. The 0.5 mmol scale reactions were performed using the Carousel reactor (2.1) with small magnetic stirrers and electric heating. There is a fluctuation in the conversion of **P_i** in the series of experiments with identical reaction scale, which may be related to the solid state of the crude mixture, the use of starting molecules from different batches (MPG, labelled and unlabelled urea and **P_i**), and the generally low solubility of the mixture after heating. ***** – $[^{15}N_2]$urea (**2b**) used ; **§** – $[^{13}C]$urea (**2c**) used ; **#** – **$[^{18}O_4]P_i$** used.

Reaction scale, mmol	5cMPGP, %	6cMPGP, %	MPGP, %	P_i, %	Total conversion, %
**0.5**	11.50	4.92	36.55	47.03	52.97
**0.5***	2.38	25.21	1.93	70.48	29.52
**0.5**	12.85	29.44	9.95	47.76	52.24
**0.5§**	16.68	78.61	4.71	-	100.00
**3**	30.99	4.77	44.11	20.13	79.87
**3**	30.13	2.96	42.63	24.28	75.72
**3**	21.96	4.37	54.21	19.46	80.54
**3**	22.05	4.14	63.35	10.46	89.54
**3**	20.26	2.06	63.40	14.28	85.72
**3**	29.95	4.62	52.56	12.87	87.13
**3§#**	35.87	8.71	46.03	9.39	90.61
**3§**	23.05	2.49	59.80	14.66	85.34
**3*#**	49.04	9.76	32.84	8.36	91.64
**3***	48.71	16.79	28.92	5.58	94.42
**3§#**	37.10	6.00	51.94	4.96	95.04
**6§#**	39.51	8.21	21.20	31.08	68.92
**6§#**	42.65	8.19	42.57	6.59	93.41
**10§#**	26.95	5.19	56.18	11.68	88.32

5.3. Loss of residual water in 'dry' conditions

Supplementary Table 25. Data used for estimation of average weight loss in reactions of starting molecule (**5** or **6**), urea (**2a**, **2b** or **2c**) and NaH_2PO_4 (**P**$_i$ or **[**^{18}**O**$_4$**]P**$_i$) (1:1:1) : **m**$_{urea}$ **(g)** = initial mass of added urea; **w1 (g)** = total weight of the reaction mixture before heating; **Δw (g)** = weight difference of crude mixture before and after heating; **Δm**$_{urea}$ **(%)** = percent of urea consumed during reaction*; **m**$_{water}$ **(g)** = **Δw – m**$_{urea}$: amount of residual water; **weight % water** = **m**$_{water}$ **(g)** · 100 % / **w1 (g)**; **m**$_{water}$ **(mmol)** = **m**$_{water}$ **(g)** · 1000 / 18 g mol^{-1}: millimoles of residual water; **mol eq H**$_2$**O** = **m**$_{water}$ (mmol) / reaction scale (mmol).

Starting alcohol	Urea	P_i	Reaction scale (mmol)	m_{urea} (g)§	w1 (g)§	Δw (g)§	$Δm_{urea}$ (%)	m_{water} (g)	weight % water	m_{water} (mmol)	mol eq H_2O
Glycerol (5)	2a	P_i	30	1.81	5.53	2.66	100	0.86	15.5	47.5	1.6
	2c	P_i	3	0.18	0.46	0.31	100	0.13	28.3	7.1	2.4
	2a	P_i	3	0.18	0.47	0.06	35.4	-	-	-	-
MPG (6)	2c	$[^{18}O_4]P_i$	10	0.61	4.00	1.18	100	0.57	14.3	31.4	3.1
	2c	$[^{18}O_4]P_i$	5	0.31	2.03	0.63	100	0.33	16.3	18.2	3.6
	2b	P_i	3	0.18	1.22	0.29	100	0.10	8.2	5.8	1.9
	2c	$[^{18}O_4]P_i$	5	0.31	2.19	0.40	100	0.09	4.1	5.1	1.0
	2a	P_i	3	0.18	1.41	0.21	100	0.03	2.1	1.4	0.5
	2c	$[^{18}O_4]P_i$	3	0.18	1.36	0.21	100	0.02	1.5	1.2	0.4
	2a	P_i	3	0.18	1.34	0.20	100	0.02	1.5	1.0	0.3
	2a	P_i	3	0.18	1.34	0.19	100	0.01	0.7	0.6	0.2
	2b	$[^{18}O_4]P_i$	3	0.19	1.42	0.14	77.5	-	-	-	-
	2c	$[^{18}O_4]P_i$	3	0.19	1.42	0.14	75.7	-	-	-	-
	2a	P_i	3	0.18	1.39	0.14	76.3	-	-	-	-
	2a	P_i	3	0.18	1.44	0.09	47.8	-	-	-	-
	2c	P_i	3	0.18	1.26	0.18	96.0	-	-	-	-

* If **m**$_{urea}$ **(g)** > **Δw (g)**, **Δm**$_{urea}$ **(%)** = **Δw (g)** · 100 % / **m**$_{urea}$ **(g)** ; if **m**$_{urea}$ **(g)** < **Δw (g)**, **Δm**$_{urea}$ **(%)** = 100 %

§ Values in [g] rounded to two digits (for the sake of readability in this table), the calculations were made with the protocolled values (four digits after the comma).

5.4. Supplementary conclusion on the urea-assisted phosphorylation of glycerol (5) and MPG (6)

For all above reactions using urea in the 'dry' state, the weight losses of the crude reaction mixtures, measured after 5 days of heating at 115 °C (Supplementary **Table 25**), corresponded to the amounts of urea that was converted to degassed ammonia and carbon dioxide and, in addition, any loss of residual water due to direct evaporation and, indirectly, through hydrolysis of cyanate followed by the decarboxylation of the intermediate carbamic acid (**CA**$_i$). At all larger scales (5, 10, 30 mmoles urea and glycerol or MPG), and most of the 3 mmol-scale experiments, we thus determined typically 4 to (most often about) 15 weight percent initial water content spanning, especially in the small-scale experiments, between 0.7 w% and 28.3 w%. This corresponds to typically 1-3 mol equivalents initially present water with respect to initially added urea, covering at all tested reaction scales these were 20 mol% to 3.6 mol equivalents residual water that reacted or evaporated from the hot neat ('dry') reaction mixture being at first in a mixed solid-liquid state. However, initially added excess water, 0.5 ml H_2O/mmol **P**$_i$, thus, about 30 mol equivalents that visibly evaporated during the first 2-3 days when heated to 115 °C ('wet and evaporating', 'wet-to-dryness' conditions), did not significantly change the outcome of glycerol and MPG phosphorylations after 5 days.

In a neat equimolar mixture of all added compounds, urea, phosphate and glycerol (**5**) or MPG (**6**), the initial decay of urea was roughly twice as fast as the consumption of **P**$_i$ being the second most rapid

process: $t_{1/2\ Pi}$ = 0.57 ± 0.04 h and 0.48 ± 0.01 h, respectively. At the same time, both **P_i** and organo-phosphates efficiently promoted urea decay that slowed down after an initial very rapid phase, giving an overall $t_{1/2\ urea\ decay}$ = 0.96 h, and nitrogen exchange, $t_{1/2\ N\text{-}exchange}$ = 2.6 ± 0.4 h, through the transient formation of cyanate followed by the addition of **P_i** (transformed to monoesters), of organo-phosphate (to cyclic and acyclic diesters) and/or of H_2O (decay) or NH_3 (N-exchange). Notably, **P_i** was more effective in degrading urea than the glyceryl phosphates. This is consistent with the fact that phosphate activation by urea to the **CP_i**, or its organo-phosphoric carbamoyl analogue $ROPO_2OC(=O)NH_2$, requires protons to be consumed and evaporated as ammonia[18]. Protons are roughly twice as abundant in **P_i** than in organo-phosphates. Hence, the kinetic and spectroscopic data did suggest that **P_i** was rapidly reacting with cyanate to produce the intermediate **CP_i**, and that 1,2-diols and phosphate monoesters added to cyanate as well, albeit at 4-6-fold slower rates. Nonetheless, this was no direct proof for the subsequent alcoholysis of **CP_i** as shown in Supplementary **Fig. 15**. Notably, the experiments thus far, that is, the use of starting compounds enriched in ^{13}C, ^{15}N, and ^{18}O isotopes but analysing only the solid crude reaction mixtures obtained from experiments carried out under hot neat conditions, and open to the air, could not categorically exclude the concomitant associative water elimination (phosphate dehydration) supposedly catalysed by urea, as theoretically analysed and shown Supplementary **Fig. 14**.

6. Tracing stable isotopes in volatiles evaporating from ‘dry’ phosphorylation reactions

6.1. Trap 1: Gaseous nucleophiles (ammonia, water) trapped as benzamide and benzoic acid

Supplementary Table 26. List of experiments performed in experimental setup shown in Supplementary **Fig. 12** for the study of prebiotic reactions by tracing stable isotopes carried over in the gas phase.

Number of the experiment	Starting molecule	Condensing agent/liquidiser	P_i	Solvent	Reaction scale, mmol
**1.1**	MPG (**6**)	**2b**	**[$^{18}O_4$]P_i**	-	3
**1.2**	**6**	**2b**	**P_i**	-	3
**1.3**	**6**	**3b**	**[$^{18}O_4$]P_i**	-	3
**1.4**	**6**	**4b**	**[$^{18}O_4$]P_i**	-	3
**1.5**	**6**	**2c**	**[$^{18}O_4$]P_i**		3
**1.6**	**6**	**2c**	**[$^{18}O_4$]P_i**	-	3
**1.7**	**6**	**2c**	**[$^{18}O_4$]P_i**	-	10
**1.8**	**6**	**2c**	**[$^{18}O_4$]P_i**	-	6
**1.9**	**6**	**2c**	**[$^{18}O_4$]P_i**	-	6
**1.10**	**6**	**2c**	**[$^{18}O_4$]P_i**	H_2O	6
**1.11**	**6**	**2a**	**P_i**	H_2O	10
**1.12**	Glycerol (**5**)	**2a**	**P_i**	H_2O	10

Supplementary Table 27. Results of the study of isotope ratio of benzamide (and sometimes benzoic acid) obtained in trap 1 after 5 days in experiments **1.1**-**1.4**. The $^{14}N/^{15}N$ ratio was found by relation of absolute intensities of signals *m/z* 122.0600 ([C_7H_7NO + H]$^+$) and *m/z* 123.0571 ([$C_7H_7{}^{15}NO$ + H]$^+$) on mass spectra of the selected chromatogram peak. In all experiments there was no evidence of the presence of ^{18}O-enriched benzoic acid in trap 1, cf. Supplementary **Fig. 70**, Supplementary **Table 28** and Supplementary **Table 29**.

Experiment	Part of trap 1 studied	Isotopolog ratio of benzamide, %	
		**C_7H_7NO**	**$C_7H_7{}^{15}NO$**
**1.1**	Solution	1.9	98.1
	Precipitate	2.8	97.2
**1.2**	Solution	3.2	96.8
	Precipitate	3.0	97.0
**1.3**	Solution	8.0	92.0
**1.4**	Solution	6.0	94.0

All benzamide peaks trapped from [$^{15}N_2$]urea-assisted (**2b**) and [^{15}N]formamide-assisted (**3b**) phosphorylations of glycerol (**5**) and MPG (**6**) gave evidence for the elimination of $^{15}NH_3$ (Supplementary **Table 27**). Data of LC-MS, HRMS and LRMS analyses of the solutions and precipitates in trap 1 of experiments **1.1**-**1.12** (cf. Supplementary **Table 26**) are shown in Supplementary **Fig. 54** - Supplementary **Fig. 69**.

We also studied the possibility of the presence of ^{18}O-labelled benzoic acid in trap 1, which would provide a signature for the urea-assisted associative phosphate activation/dehydration mechanism (Supplementary **Fig. 14**). We have found small amounts of benzoic acid in experiments **1.1**, **1.3** and **1.5** (Supplementary **Fig. 70**). The deviations of the experimental from the theoretical values of the isotopolog signal intensities for natural-isotope-abundance benzoic acid are all within the experimental error margin of this analytical method, cf. Supplementary **Table 28** and Supplementary **Table 29**.

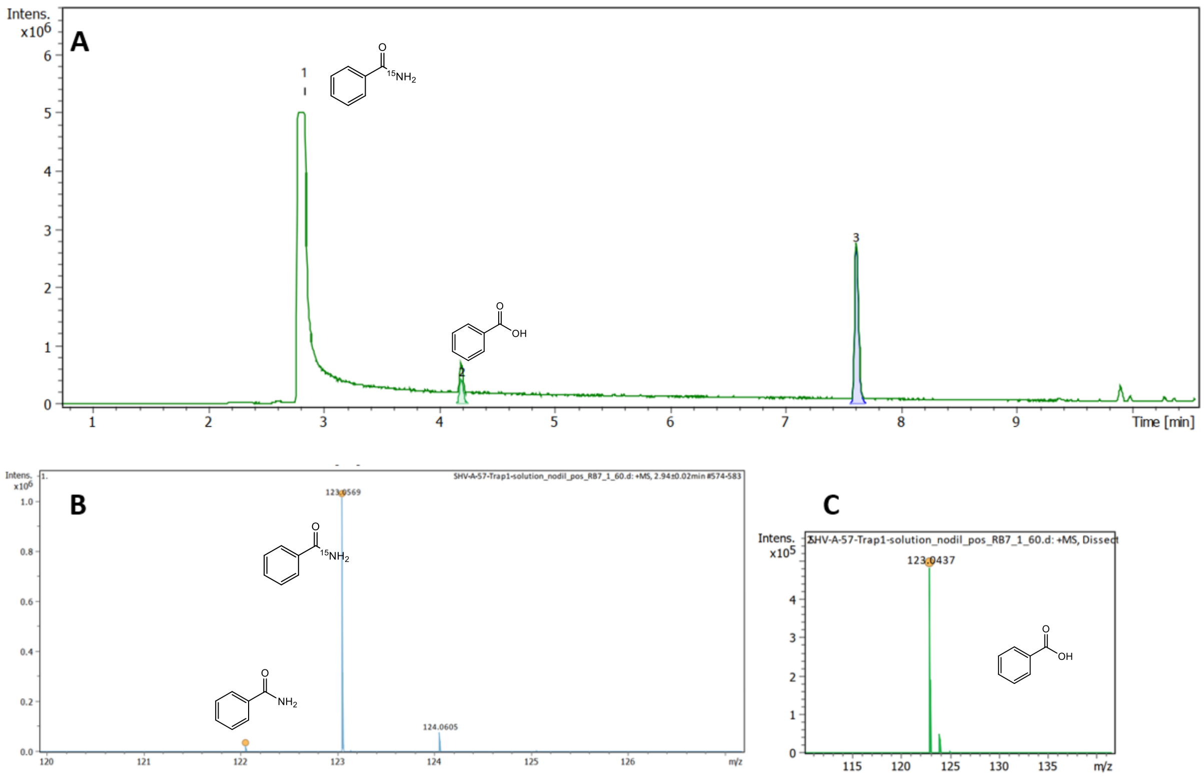

Supplementary Fig. 54. Results of HPLC-HRMS in positive-ion mode analysis of trap 1 solution of experiment **1.1** (cf. Supplementary **Table 26**).

A – Total ion chromatogram with main signals. Peak 1 at R_T = 3 min: benzamide (C_7H_7NO and $C_7H_7{}^{15}NO$); Peak 2 at R_T = 4.2 min: benzoic acid ($C_7H_6O_2$); Peak 3 at R_T = 7.6 min: contamination from vacuum filtration.

B – High-resolution mass spectrum of peak 1 (R_T = 3 min).

C – High-resolution mass spectrum of peak 2 (R_T = 4.2 min).

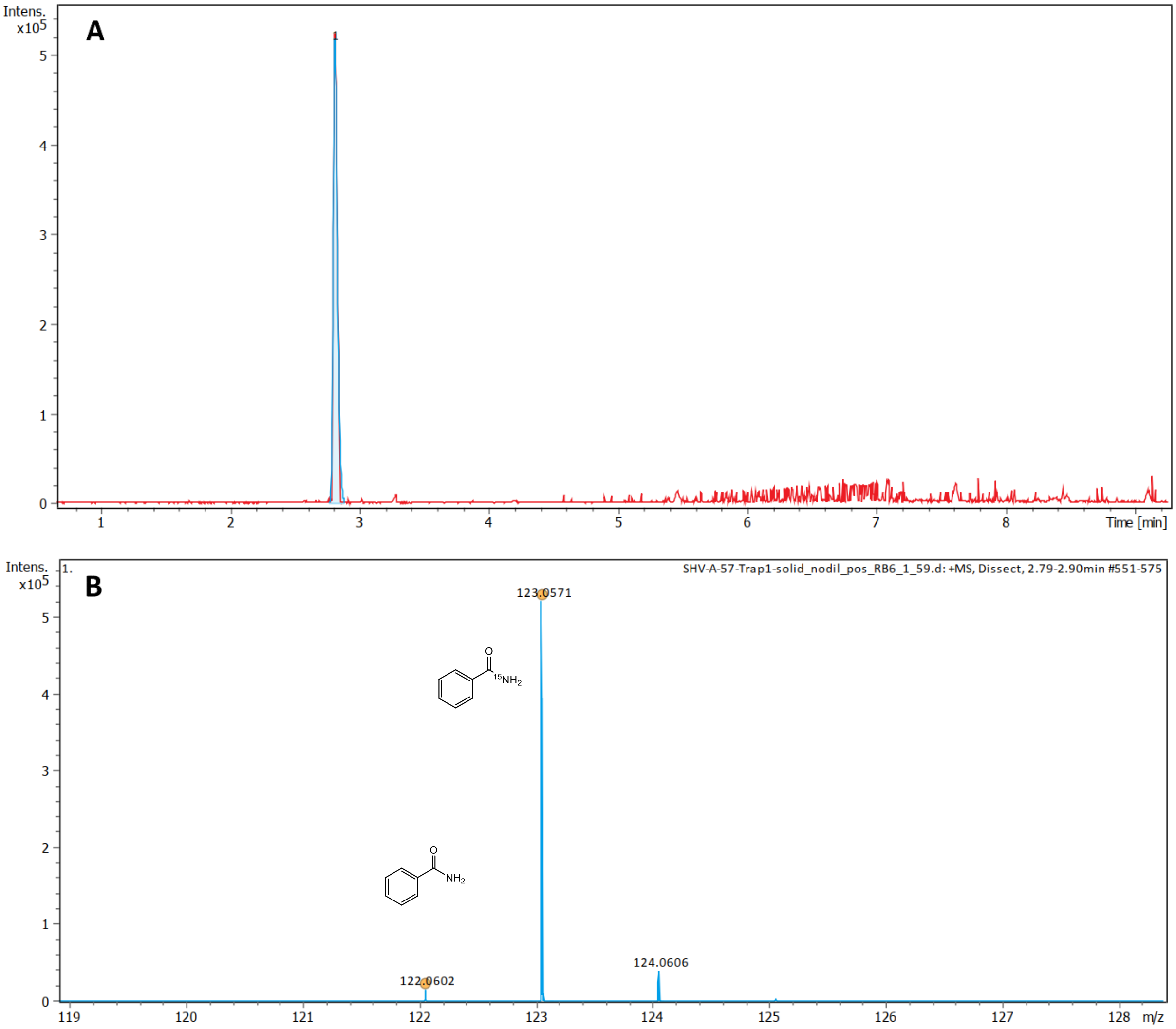

Supplementary Fig. 55. Results of LC-MS analysis of trap 1 precipitate of experiment **1.1** (cf. Supplementary **Table 26**) after 120 h of heating. **A** – chromatogram of the experiment. **B** – mass spectrum of peak #1 (R_t = 2.9 min): benzamide (C_7H_7NO and $C_7H_7{}^{15}NO$).

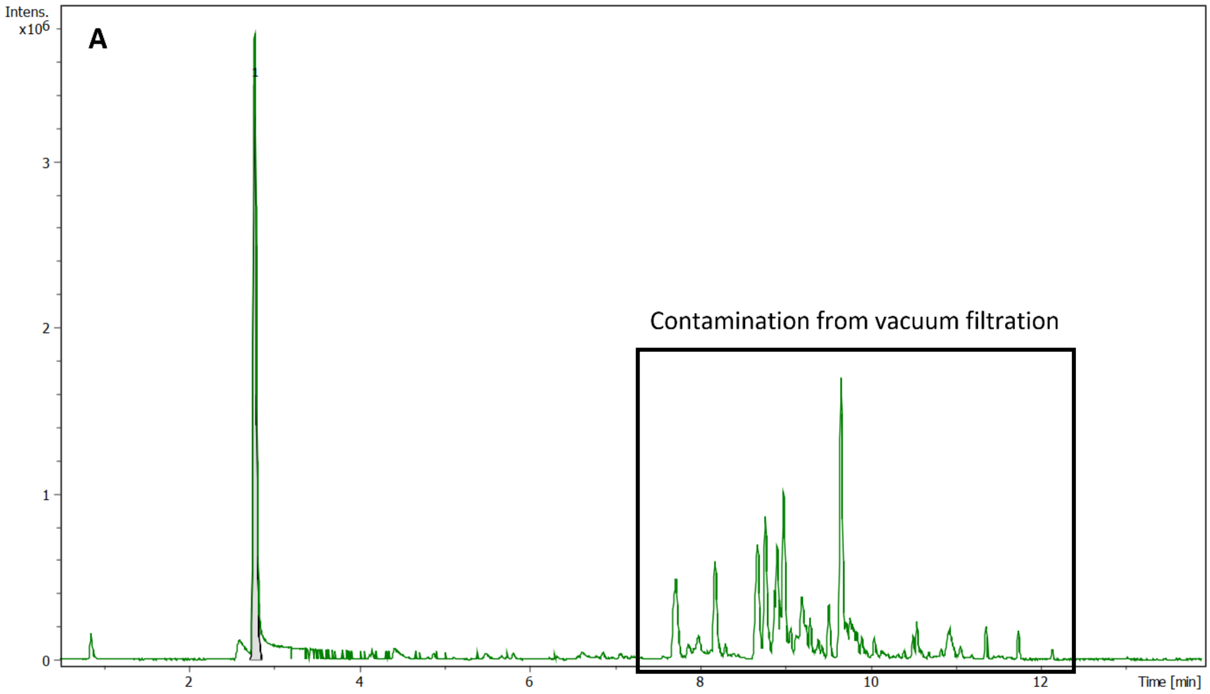

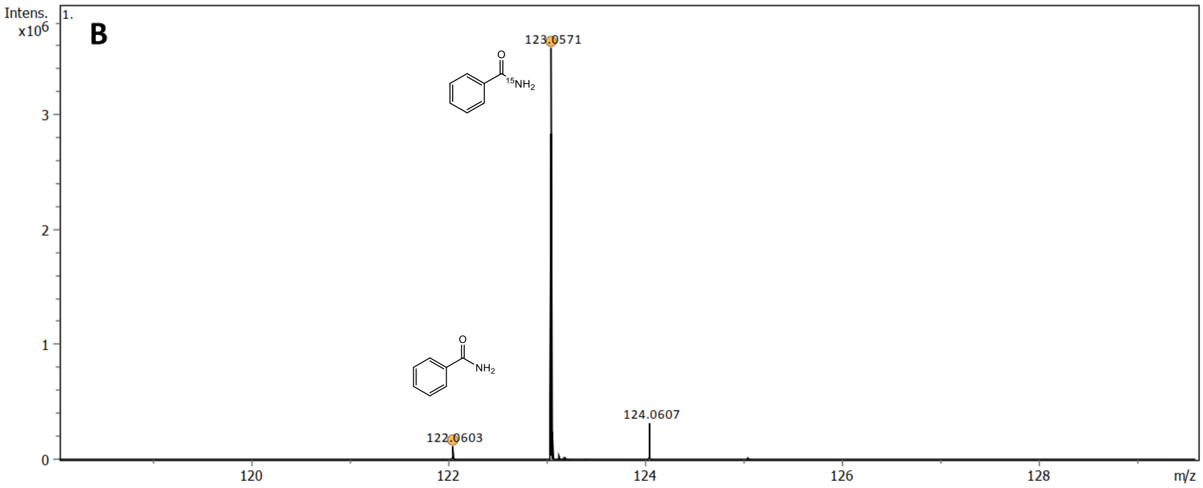

Supplementary Fig. 56. Results of LC-MS analysis of trap 1 solution of experiment **1.2** (cf. Supplementary **Table 26**) after 120 h of heating. **A** – chromatogram with main signal #1 R_t = 3 min: benzamide (C_7H_7NO and $C_7H_7{}^{15}NO$); signals after 8 min are contaminations from vacuum filtration. **B** – mass spectrum of main peak (R_t = 3 min).

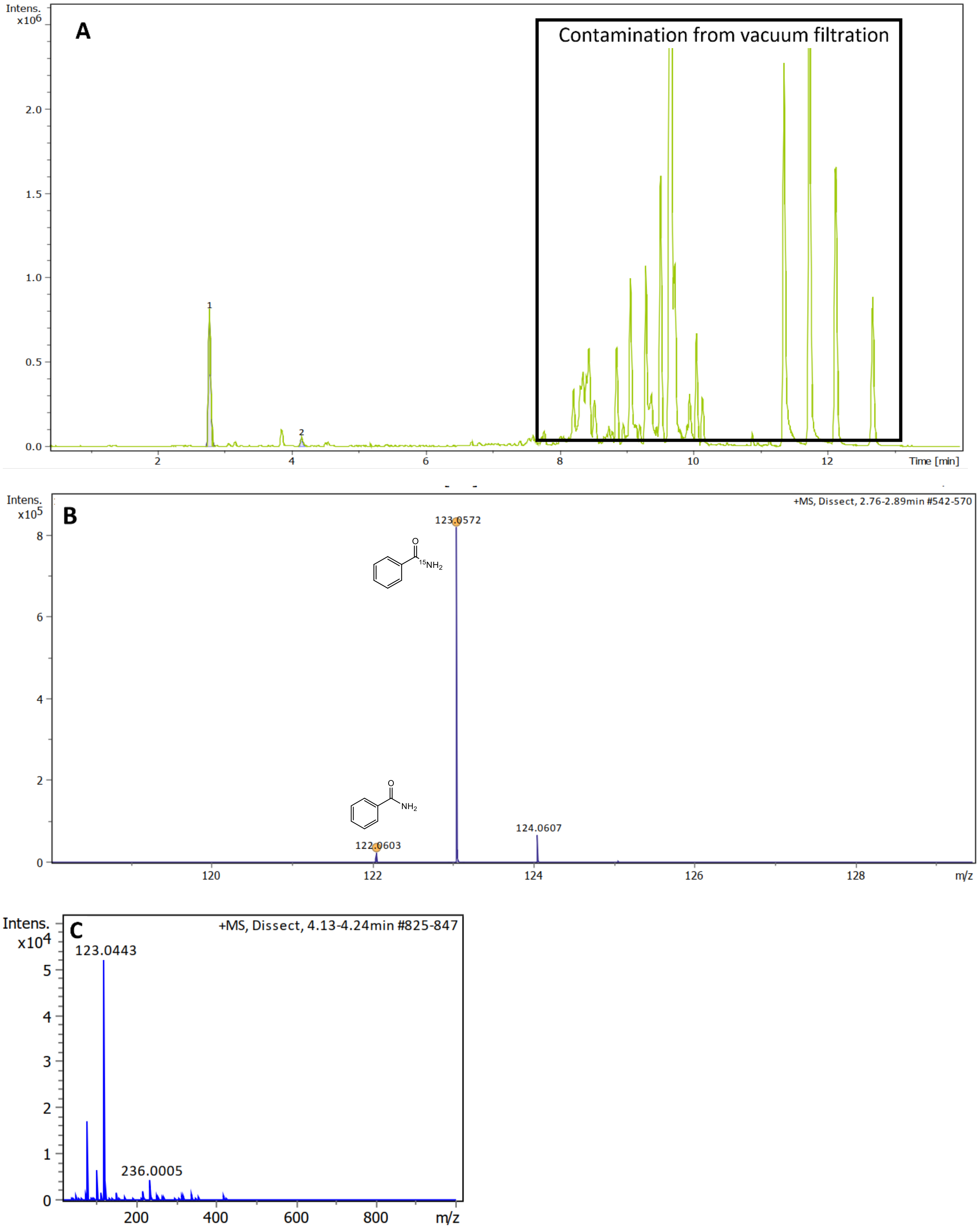

Supplementary Fig. 57. Results of LC-MS analysis of <u>trap 1 precipitate</u> of experiment **1.2** (cf. Supplementary **Table 26**) after 120 h of heating. **A** – chromatogram with main peak #1 R_t = 2.8 min: benzamide (C_7H_7NO and $C_7H_7{}^{15}NO$); #2 R_t = 4.2 min, benzoic acid ($C_7H_6O_2$). **B** – mass spectrum of peak #1 (R_t = 2.8 min). **C** – mass spectrum of peak #2 (R_t = 4.2 min).

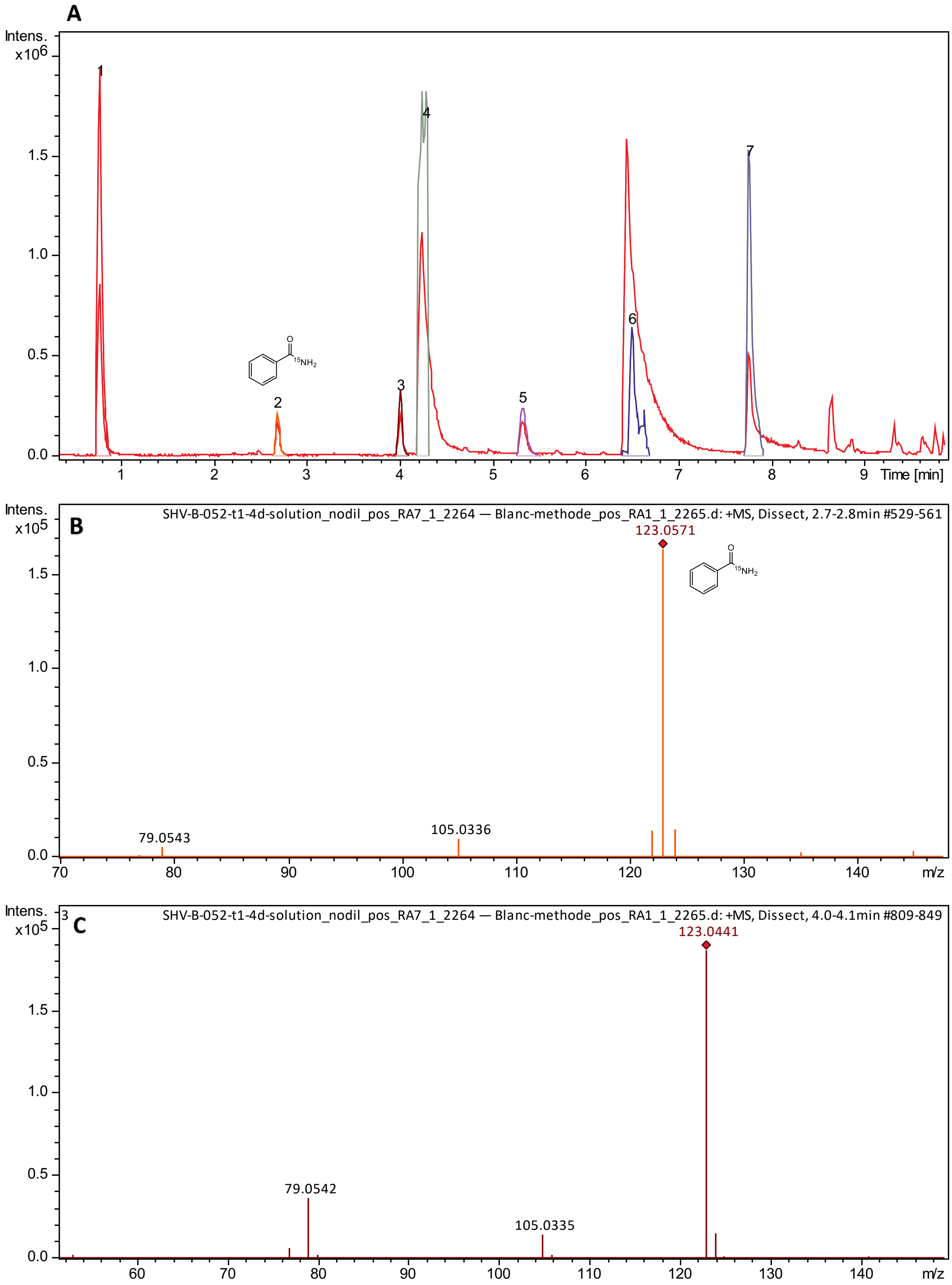

Supplementary Fig. 58. Results of LC-MS analysis of trap 1 solution of experiment **1.3** (cf. Supplementary **Table 26**) after 120 h of heating. **A** – chromatogram of the experiment, peak #2 R_t = 2.8 min: benzamide (C_7H_7NO and $C_7H_7{}^{15}NO$); #3 R_t = 4.1 min: benzoic acid ($C_7H_6O_2$). **B** – mass spectrum of peak #2 (R_t = 2.8 min). **C** – mass spectrum of peak #3 (R_t = 4.1 min).

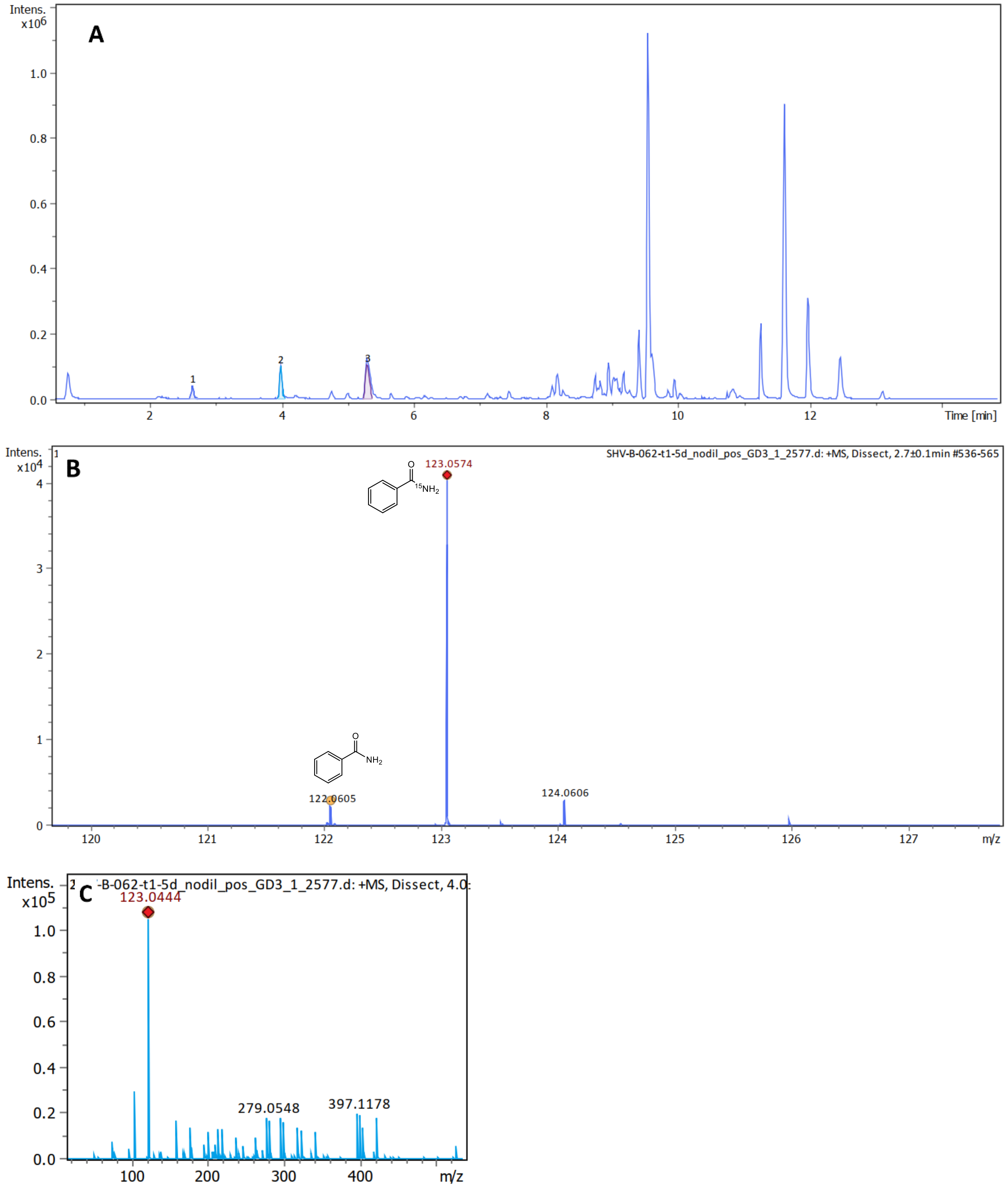

Supplementary Fig. 59. Results of LC-MS analysis of <u>trap 1 solution</u> of experiment **1.4** (cf. Supplementary **Table 26**) after 120 h of heating. **A** – chromatogram of the experiment, peak #1 R_t = 2.7 min: benzamide (C_7H_7NO and $C_7H_7{}^{15}NO$); #2 R_t = 4.0 min: benzoic acid ($C_7H_6O_2$). **B** – mass spectrum of peak #1 (R_t = 2.7 min). **C** – mass spectrum of peak #3 (R_t = 4.0 min).

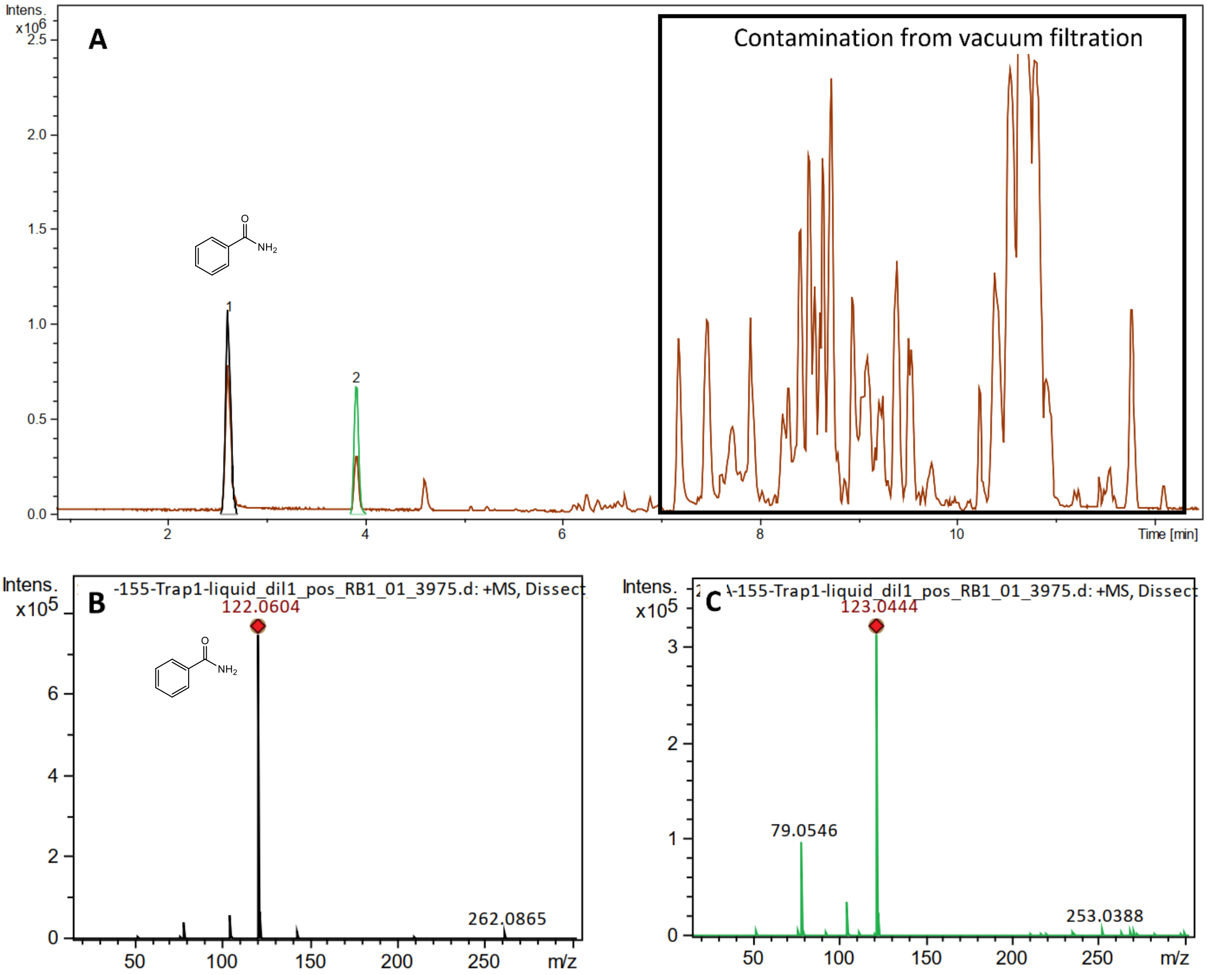

Supplementary Fig. 60. Results of LC-MS analysis of <u>trap 1 solution</u> of experiment **1.5** (cf. Supplementary **Table 26**) after 120 h of heating. **A** – chromatogram with main signals, peak #1 R_t = 2.7 min: benzamide (C_7H_7NO); #2 R_t = 3.9 min: benzoic acid ($C_7H_6O_2$). **B** – mass spectrum of peak #1 (R_t = 2.7 min). **C** – mass spectrum of peak #2 (R_t = 4.2 min).

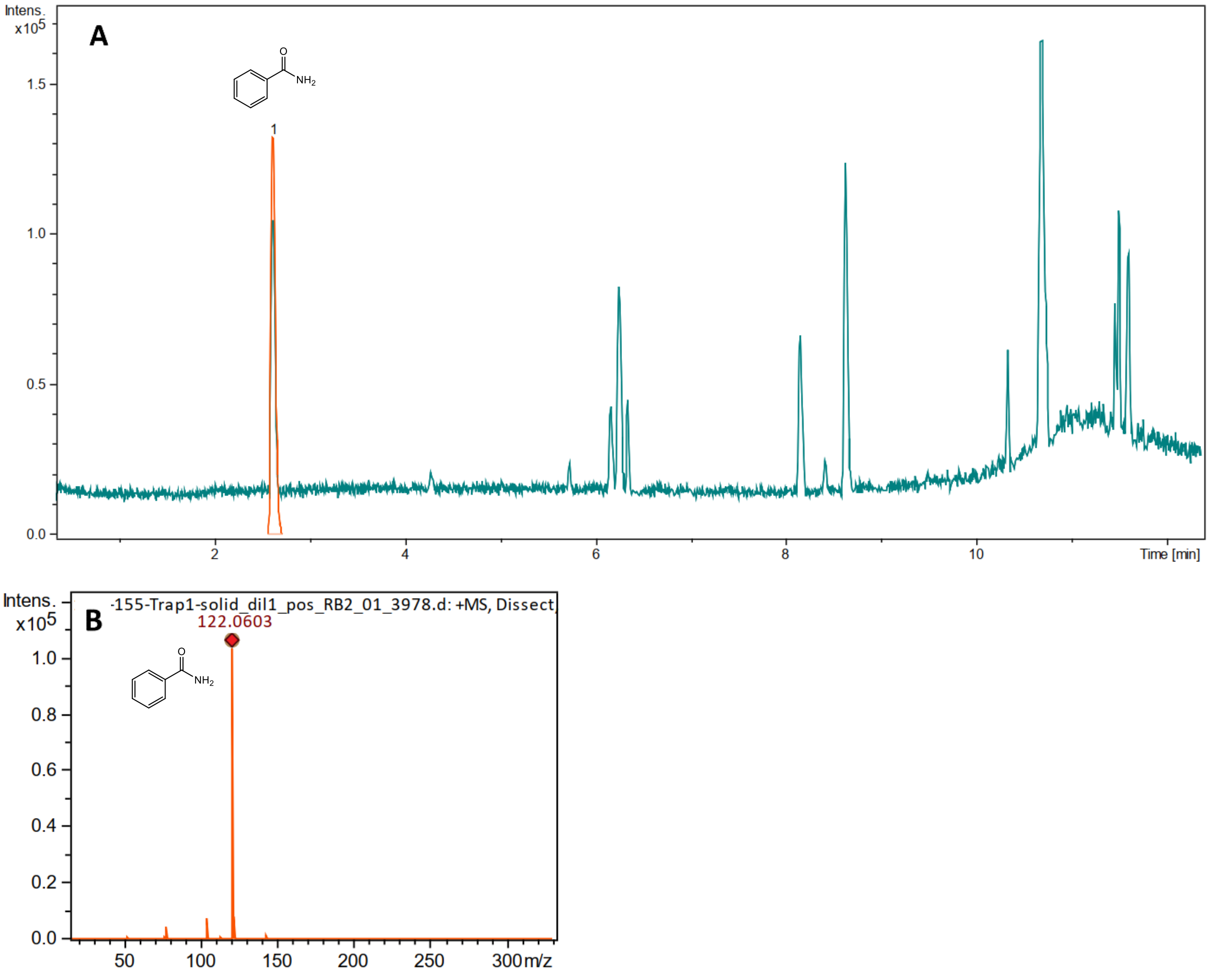

Supplementary Fig. 61. Results of LC-MS analysis of trap 1 precipitate of experiment **1.5** (cf. Supplementary **Table 26**) after 120 h of heating. **A** – chromatogram of the experiment. **B** – mass spectrum of peak #1 (R_t = 2.9 min): benzamide (C_7H_7NO).

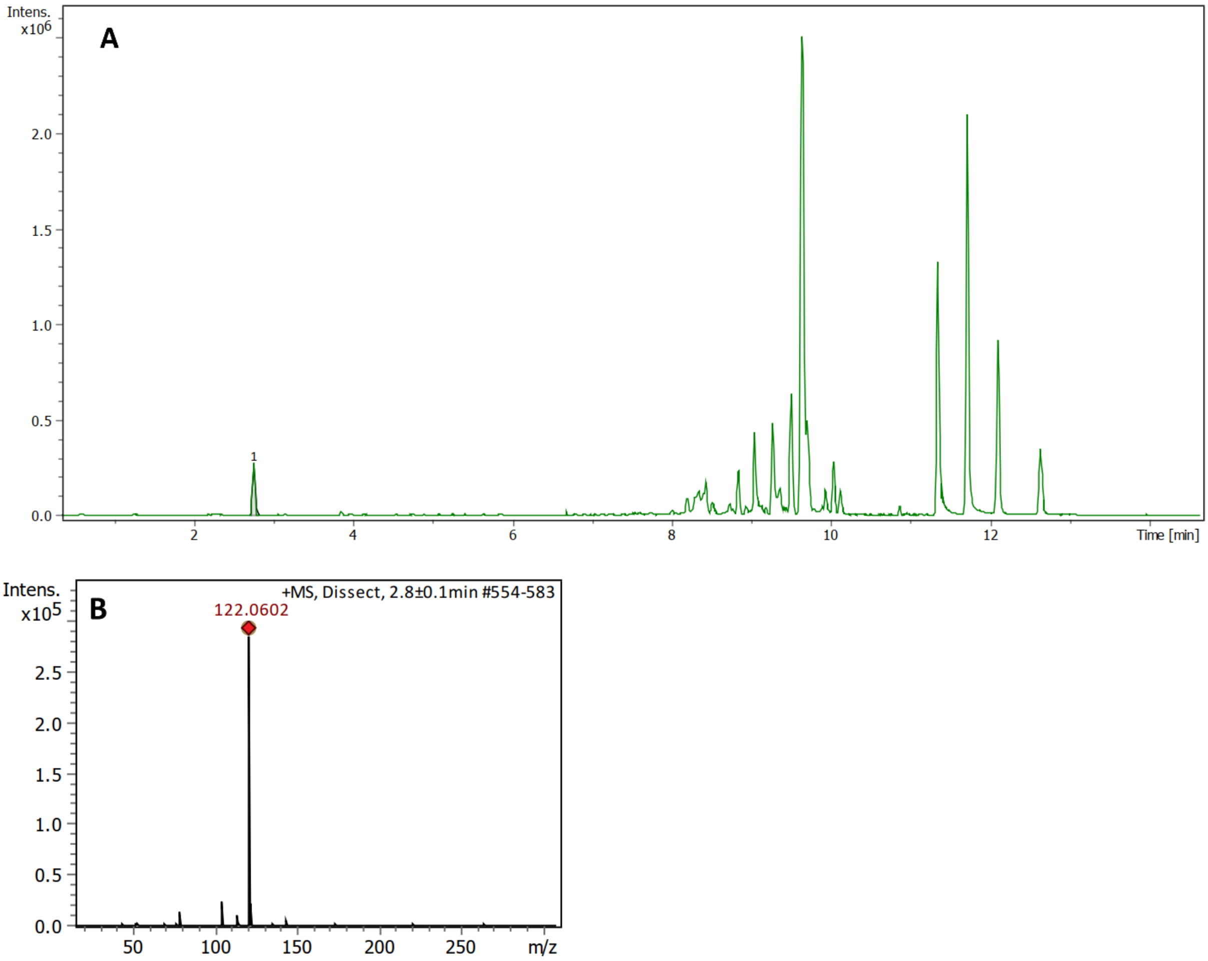

Supplementary Fig. 62. Results of LC-MS analysis of trap 1 precipitate of experiment **1.5** (cf. Supplementary **Table 26**) after 120 h of heating. **A** – chromatogram of the experiment. **B** – mass spectrum of peak #1 (R_t = 2.9 min): benzamide (C_7H_7NO).

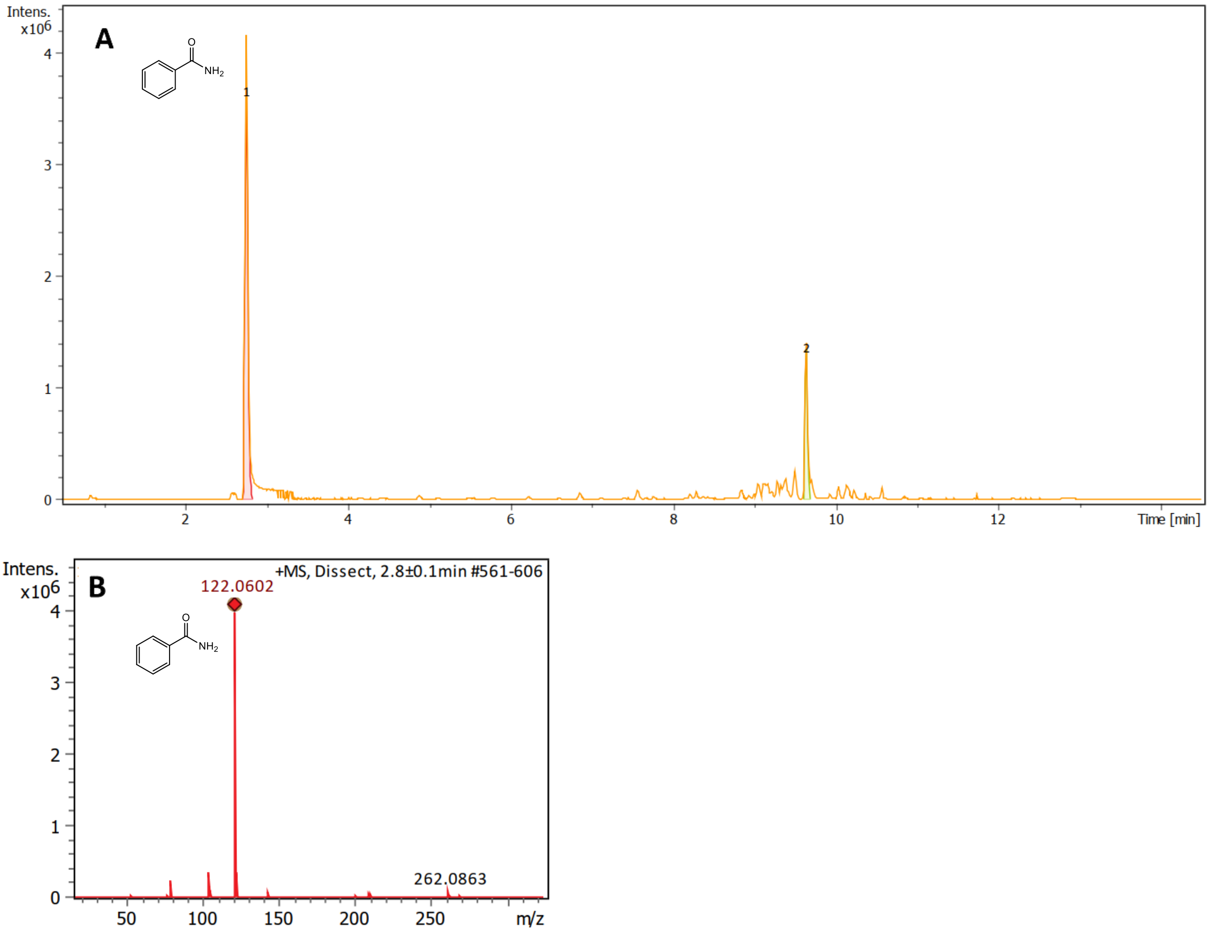

Supplementary Fig. 63. Results of LC-MS analysis of <u>trap 1 solution</u> of experiment **1.6** (cf. Supplementary **Table 26**) after 120 h of heating. **A** – chromatogram of the experiment. **B** – mass spectrum of peak #1 (R_t = 2.8 min): benzamide (C_7H_7NO).

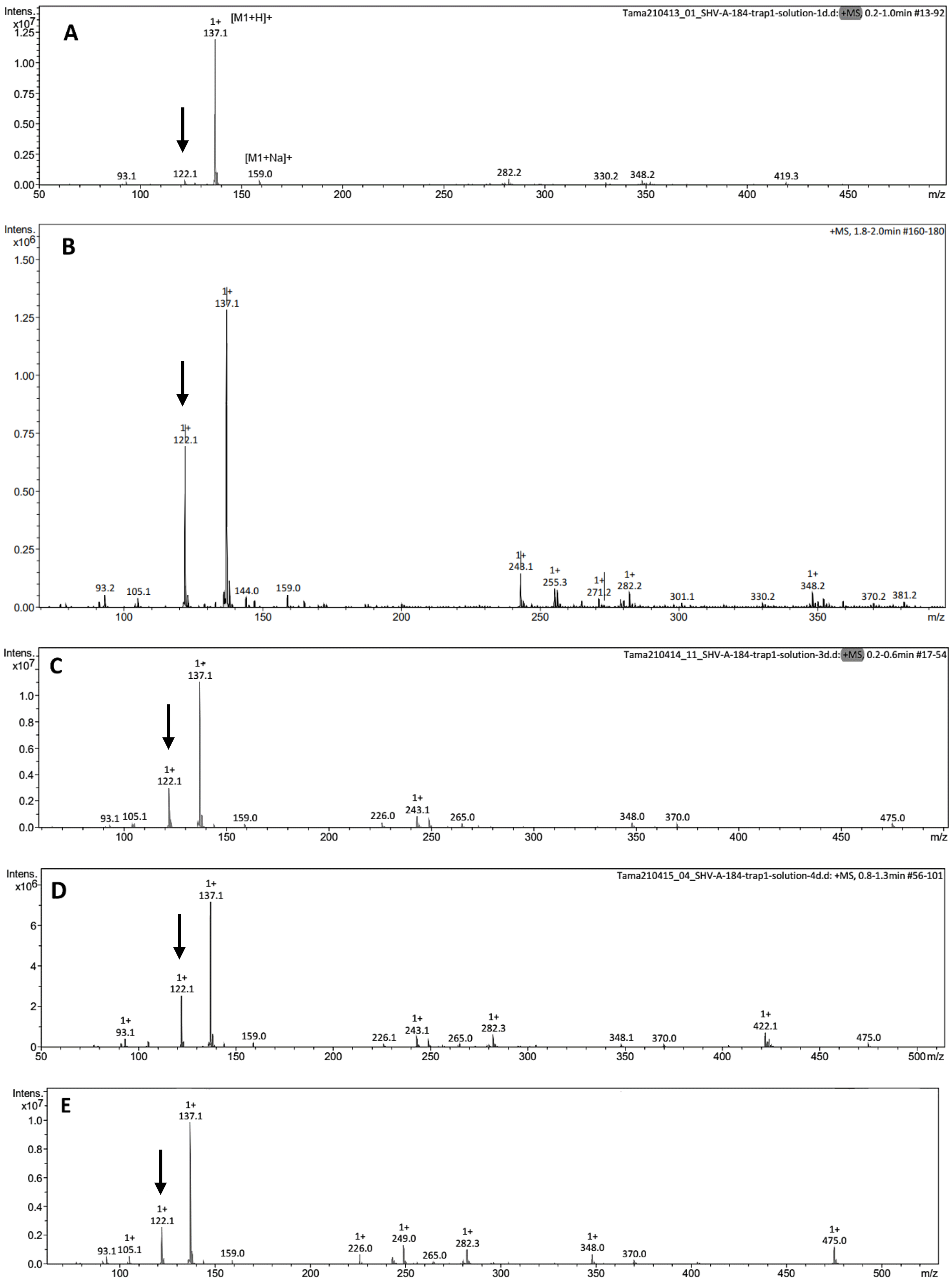

Supplementary Fig. 64. Results of low-resolution positive ion mode mass analyses of <u>trap 1</u> of experiment **1.7** (cf. Supplementary **Table 26**). **A**-**E** – the solution was sampled after 24, 48, 72, 96 and 120 h of heating (sampling directly from the trap 1 during the reaction). Arrow shows the presence of benzamide in the studied solution.

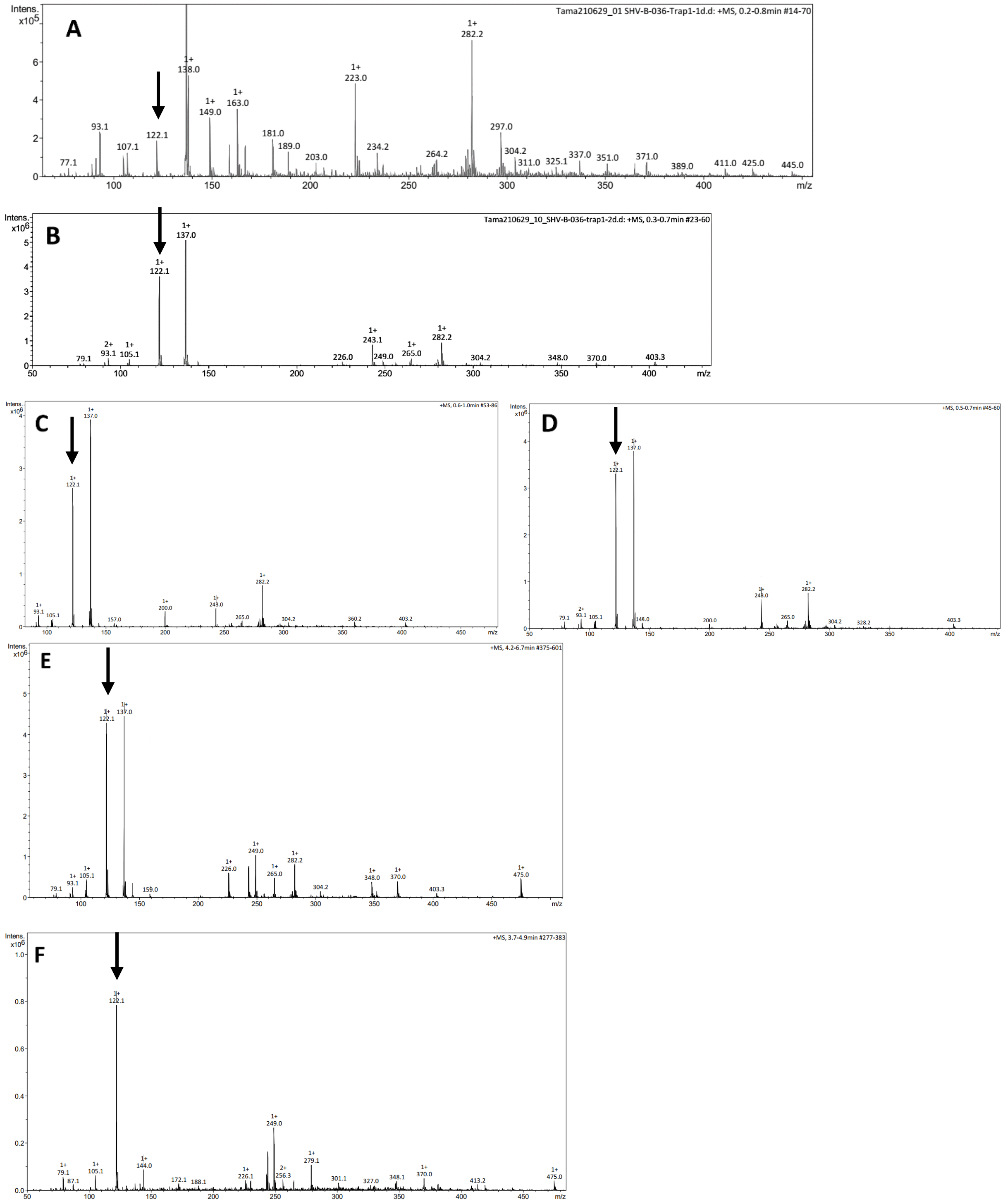

Supplementary Fig. 65. Results of low-resolution positive ion mode mass analyses of <u>trap 1</u> of experiment **1.8** (cf. Supplementary **Table 26**). **A**-**D** – the solution was sampled after 24, 48, 72 and 96 h of heating (sampling directly from the trap 1 during the reaction). **E** – sample of trap 1 solution and **F** – sample of trap 1 precipitate after the end of the reaction and filtration. Arrow shows presence of benzamide in the studied solution/precipitate.

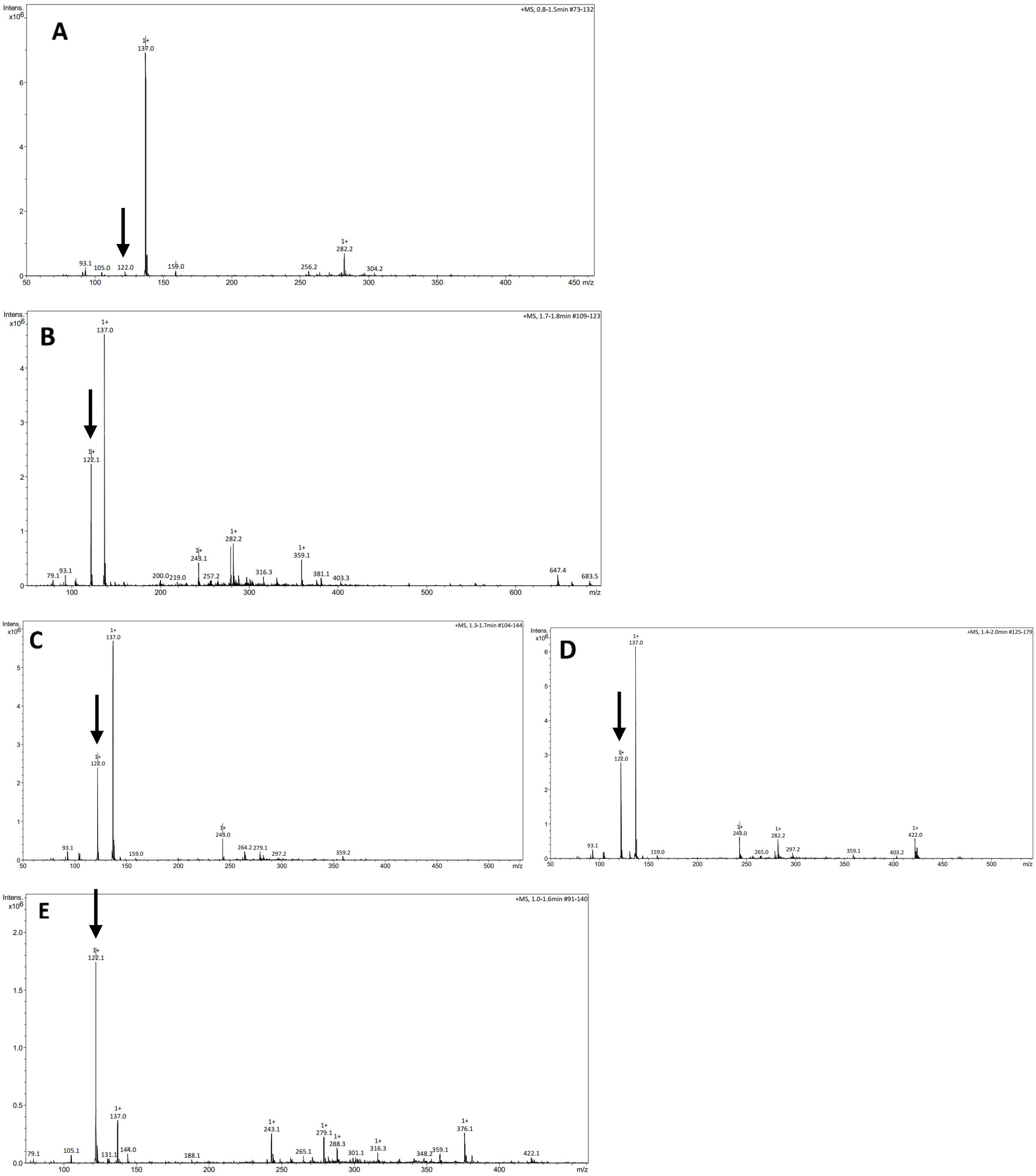

Supplementary Fig. 66. Results of low-resolution positive ion mode mass analyses of trap 1 of experiment **1.9** (cf. Supplementary **Table 26**). **A**-**C** – the solution was sampled after 24, 48 and 96 h of heating (sampling directly from the trap 1 during the reaction). **D** – sample of trap 1 solution and **E** – sample of trap 1 precipitate after the end of the reaction and filtration. Arrow shows presence of benzamide in the studied solution/precipitate.

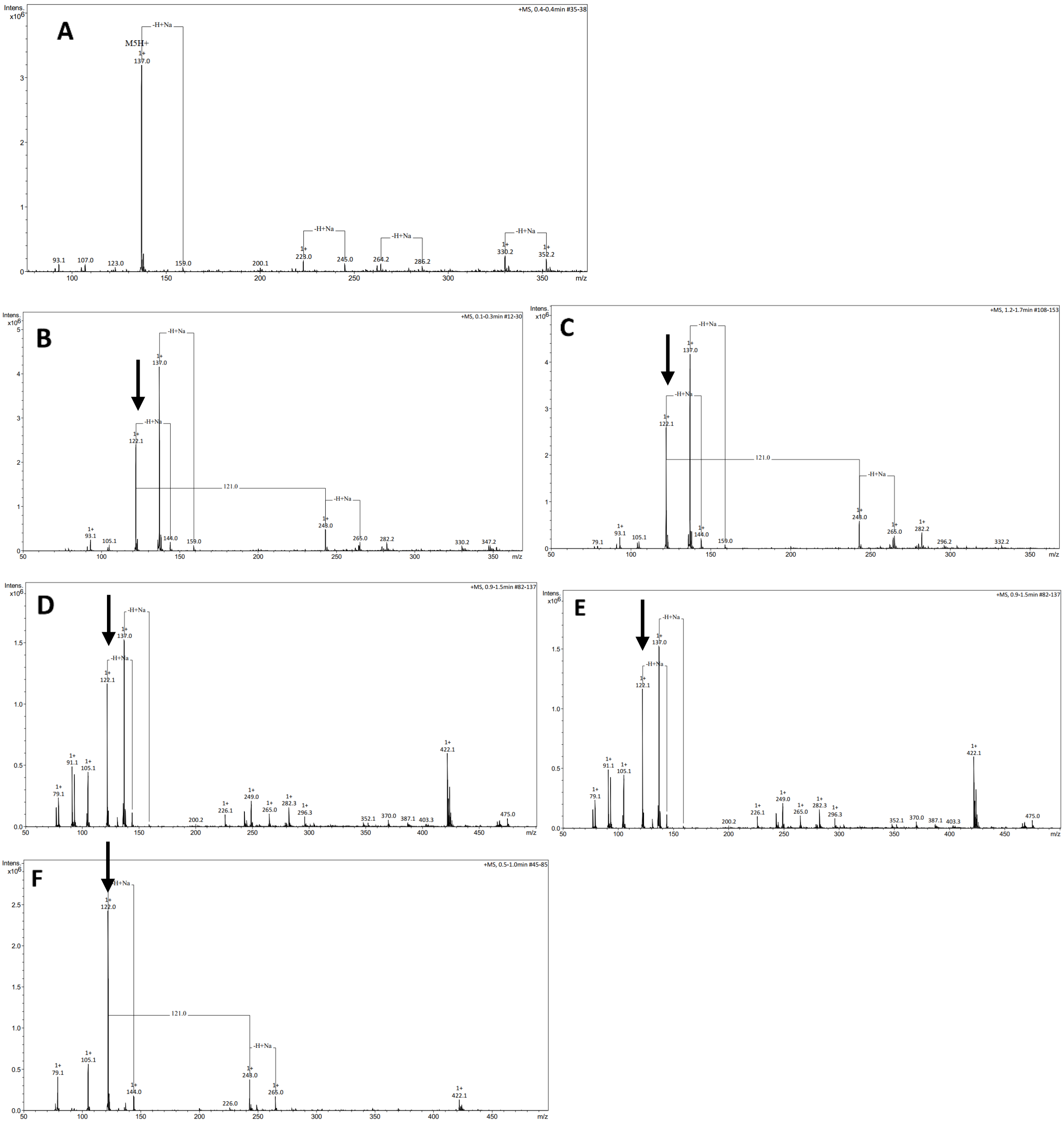

Supplementary Fig. 67. Results of low-resolution positive ion mode mass analyses of <u>trap 1</u> of experiment **1.10** (cf. Supplementary **Table 26**). **A**-**D** – the solution was sampled after 24, 48, 72 and 96 h of heating (sampling directly from the trap 1 during the reaction). **E** – sample of trap 1 solution and **F** – sample of trap 1 precipitate after the end of the reaction and filtration. Arrow shows presence of benzamide in the studied solution/precipitate.

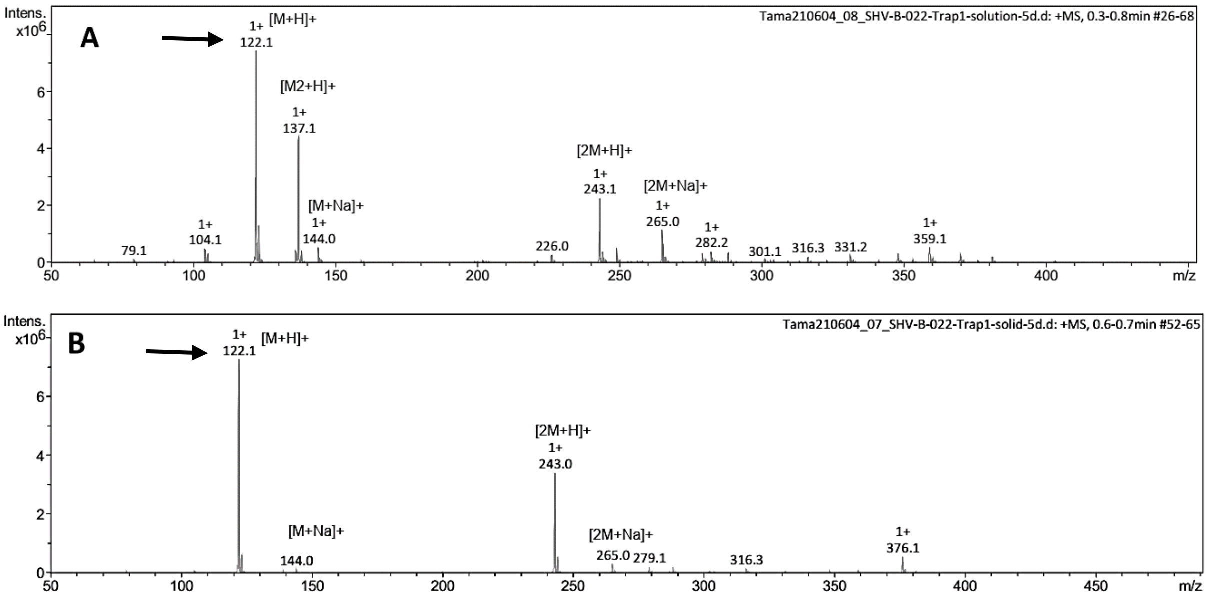

Supplementary Fig. 68. Results of low-resolution positive ion mode mass analyses of trap 1 of experiment **1.11** (cf. Supplementary **Table 26**) after 120 h of heating. **A** – sample of trap 1 solution and **B** – sample of trap 1 precipitate after the end of the reaction and filtration. Arrow shows presence of benzamide in the studied solution/precipitate.

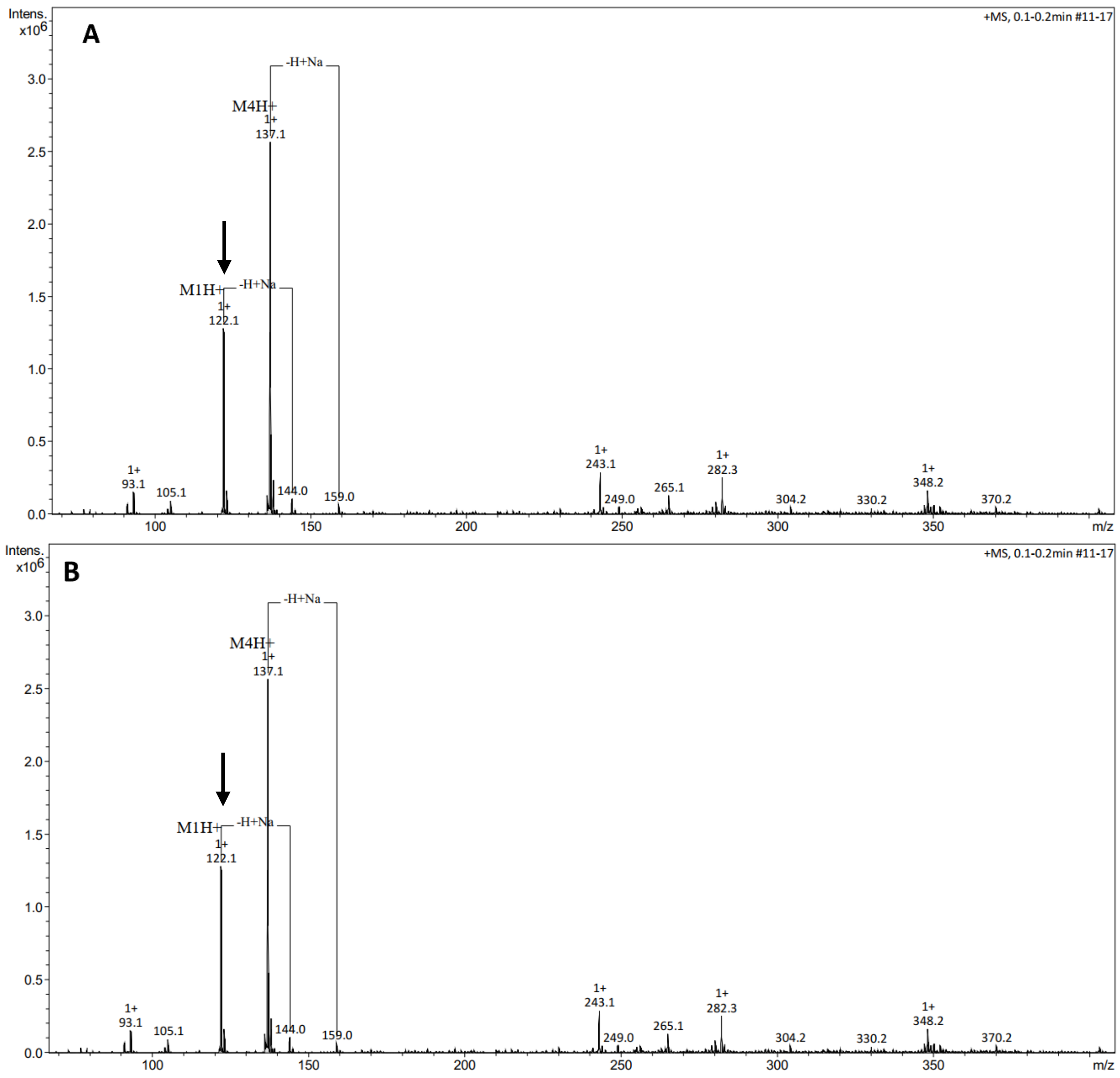

Supplementary Fig. 69. Results of low-resolution positive ion mode mass analyses of trap 1 of experiment **1.12** (cf. Supplementary **Table 26**) after 120 h of heating. **A** – sample of trap 1 solution and **B** – sample of trap 1 precipitate after the end of the reaction and filtration. Arrow shows presence of benzamide in the studied solution/precipitate.

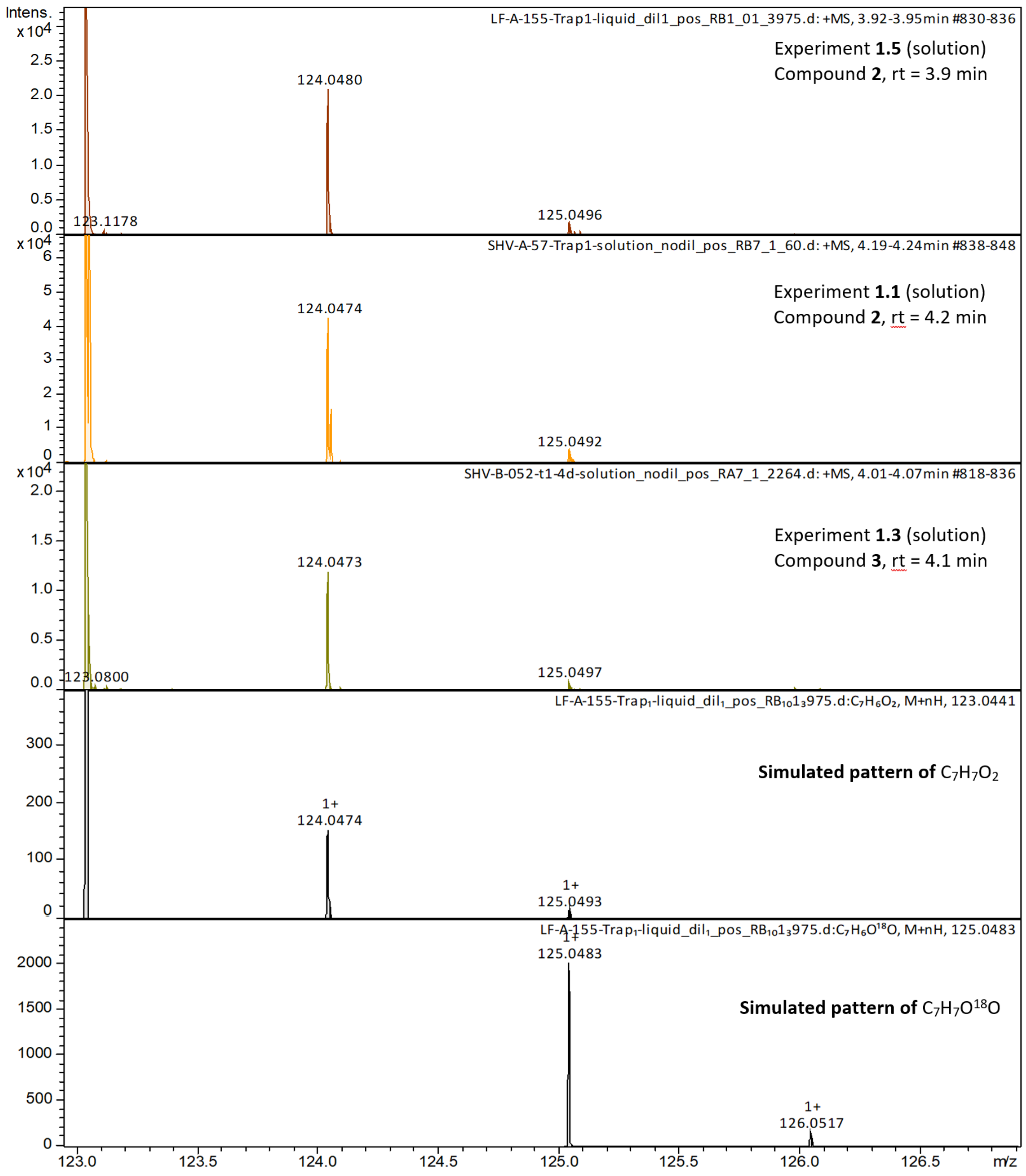

Supplementary Fig. 70. Comparison of the mass spectra of benzoic acid found in trap 1 (peak 2, R_T = 4.2 min) in the experiments **1.1**, **1.3** and **1.5** (cf. Supplementary **Table 26**) with the simulated isotopolog mass distribution of natural isotope-abundance benzoic acid and ^{18}O-labelled benzoic acid.

Supplementary Table 28. Comparison of the isotope ratio of MS signal intensities of benzoic acid found in trap 1 (peak 2, R_T = 4.2 min) in the experiments **1.1**, **1.3** and **1.5** (cf. Supplementary **Table 26**) with the simulated isotopolog mass distribution of natural isotope-abundance benzoic acid.

m/z	Formula	Intensity of selected signals, %			
		Experiment **1.5**	Experiment **1.1**	Experiment **1.3**	Natural isotopolog pattern
123.0438	$^{12}C_7{}^1H_7{}^{16}O_2$	92.07	92.05	91.89	92.25
124.0473	$^{12}C_6{}^{13}C\,{}^1H_7{}^{16}O_2$	7.36	7.36	7.53	7.13
125.0492	$^{12}C_5{}^{13}C_2\,{}^1H_7{}^{16}O_2$	0.57	0.59	0.58	0.62

To be confident that the minor signal *m/z* 125.0492 in these isotopolog patterns indeed belonged to natural isotope-abundance benzoic acid $^{12}C_5{}^{13}C_2{}^1H_7{}^{16}O_2$ (M+2 for two ^{13}C) (Supplementary **Fig. 70**) and was not *m/z* 125.0496 that would originate from ^{18}O-labelled benzoic acid, M = $^{12}C_7{}^1H_7{}^{16}O^{18}O$, we used the "automatic signal assignment" (Bruker software) tool and forced the analytical program to fit formula $C_7H_7O^{18}O$ to signal 125.0492. The obtained data summarised in Supplementary **Table 29** (assignment of benzoic acid to *m/z* 123.0444 shown as an example of good correspondence of the formula to signal). Values of mSigma (isotopic pattern fit factor) for the suggested ^{18}O-labelled benzoic acid and the error margin are above the standard deviation or are indefinable.

Supplementary Table 29. Results of using automatic tool for signal assignment of Bruker software.

Experiment	Measured *m/z*	Ion Formula	Sum Formula	error [ppm]	mSigma	Adduct
**1.5**	123.0444	$C_7H_7O_2$	$C_7H_6O_2$	−2.6	1.6	M+H
	125.0496	$C_7H_7O^{18}O$	$C_7H_6O^{18}O$	−10.5	n.a.	M+H
**1.1**	123.0438	$C_7H_7O_2$	$C_7H_6O_2$	2.2	1.4	M+H
	125.0492	$C_7H_7O^{18}O$	$C_7H_6O^{18}O$	−7.5	n.a.	M+H
**1.3**	123.044	$C_7H_7O_2$	$C_7H_6O_2$	0.1	2.7	M+H
	125.0497	$C_7H_7O^{18}O$	$C_7H_6O^{18}O$	−11.1	n.a.	M+H

Consequently, following the data of Supplementary **Fig. 70**, Supplementary **Table 28** and Supplementary **Table 29** we can safely confirm the complete absence of measurable ^{18}O-isotopes carried over from **[$^{18}O_4$]P_i** to benzoic acid found in trap 1.

6.2. Trap 2: CO_2 trapped as benzoic acid (benzamide from trapping HNCO not found by LC-MS)

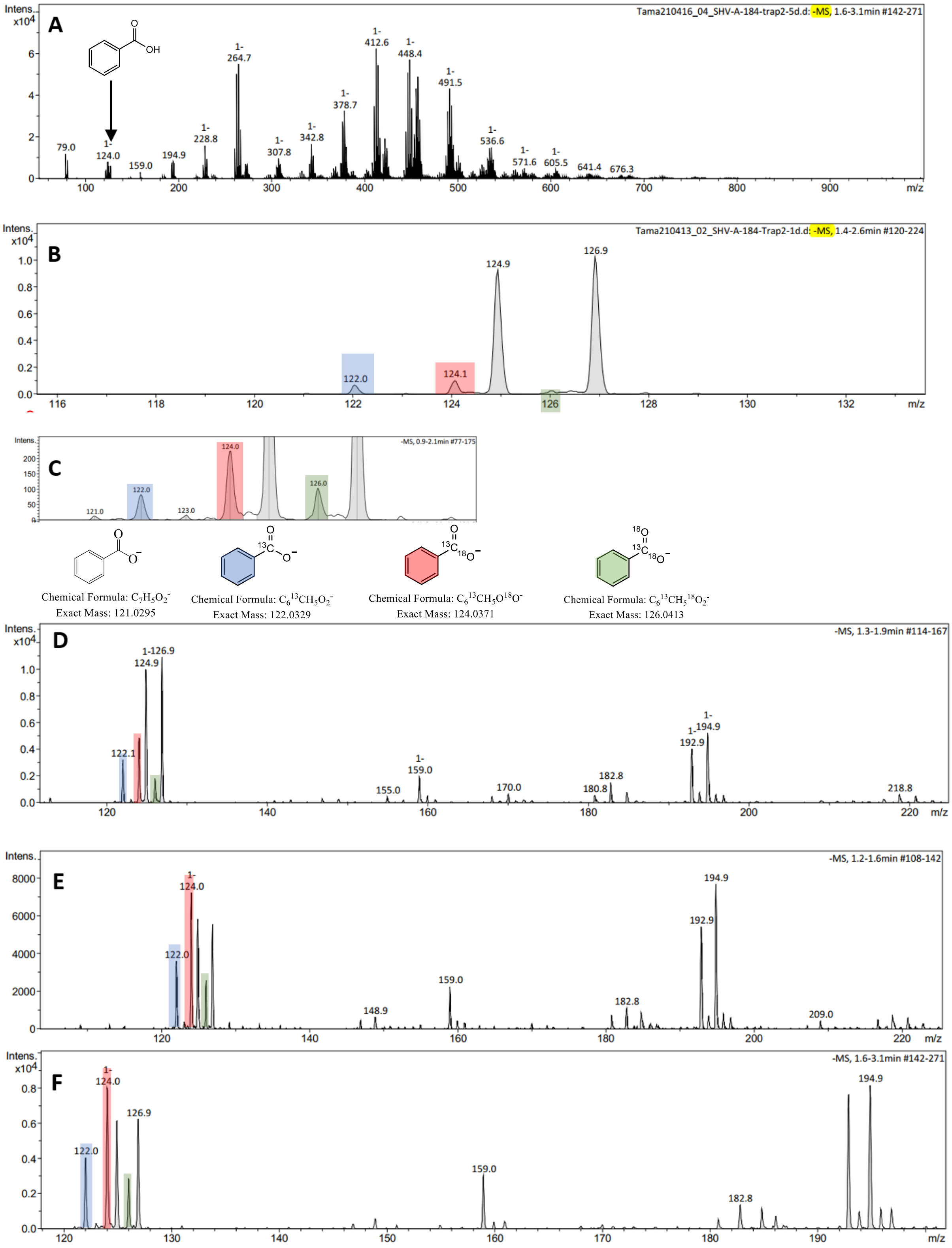

Supplementary Fig. 71. Results of low-resolution negative-ion mode mass analyses of trap 2 of experiment **1.7** (cf. Supplementary **Table 26**). **A** – total-ion chromatogram after 120 h of heating. **B-F** – sampling of trap 2 solution after 24, 48, 72, 96 and 120 hours after the start of the heating (sampling directly from the trap 2 during the reaction). [^{13}C]benzoic acid *m/z* 122.0329 [M–H]$^{-}$ marked blue, [^{13}C^{18}O]benzoic acid *m/z* 124.0371 [M–H]$^{-}$ marked red, [^{13}C^{18}O$_2$]benzoic acid *m/z* 126.0413 [M–H]$^{-}$ marked green.

Given the low concentration of benzoic acid in trap 2 solutions, the isotopolog enrichments measured over reaction time passing varied quite strongly but did not change the overall tendency of enrichment of a particular $C_7H_6O_2$-isotopolog ($^{13}C^{16}O_2$, $^{13}C^{16}O^{18}O$ and $^{13}C^{18}O_2$) over time (Supplementary **Table 30**). More data of LC-MS, HRMS and LRMS analyses of the trap 2 solution of other experiments are given in Supplementary **Fig. 73**-Supplementary **Fig. 80**.

Supplementary Table 30. Results of the study of isotopolog ratios of benzoic acid obtained from trap 2 during 5 days of experiments **1.6**, **1.8** and **1.10** (cf. Supplementary **Table 26**). The ratios were determined from the absolute intensities of signals *m/z* 121.0295 ($[C_7H_5O_2 – H]^–$), 122.0329 ($[C_6H_5O_2{}^{13}C – H]^–$), 124.0371 ($[C_6H_5O^{13}C^{18}O – H]^–$) and 126.0413 ($[C_6H_5{}^{13}C^{18}O_2 – H]^–$) in the HRMS of the selected chromatogram peak (LRMS shown in Supplementary **Fig. 71**).

**Heating time, h**	**Experiment 1.6**				**Experiment 1.8**				**Experiment 1.10**			
	$C_7H_6O_2$	$C_6H_6O_2$ [^{13}C]	C_6H_6O [$^{13}C^{18}O$]	C_6H_6O [$^{13}C^{18}O_2$]	$C_7H_6O_2$	$C_6H_6O_2$ [^{13}C]	C_6H_6O [$^{13}C^{18}O$]	C_6H_6O [$^{13}C^{18}O_2$]	$C_7H_6O_2$	$C_6H_6O_2$ [^{13}C]	C_6H_6O [$^{13}C^{18}O$]	C_6H_6O [$^{13}C^{18}O_2$]
**24**	3.51	38.72	57.77	-	-	-	-	-	5.06	82.91	12.03	-
**48**	3.07	19.58	52.83	24.53	37.53	34.27	28.20	-	-	92.83	7.17	-
**60**	-	-	-	-	-	-	-	-	6.53	85.91	7.56	-
**72**	-	32.61	49.06	18.33	17.26	36.94	39.64	6.16	14.33	74.02	11.65	-
**84**	-	-	-	-	-	-	-	-	10.42	79.39	10.19	-
**96**	-	26.89	54.00	19.11	-	50.15	49.85	-	4.74	86.45	8.80	-
**108**	-	-	-	-	-	-	-	-	15.57	66.16	18.27	-
**120**	-	27.23	53.76	19.01	11.48	40.62	41.93	5.97	9.30	90.70	-	-

Details about the identification of other signals of the mass spectra of trap 2 for the experiment **1.1** (cf. Supplementary **Table 26**) are shown in Supplementary **Fig. 81** and Supplementary **Table 31**, and the overall results summarised in Supplementary **Fig. 82**.

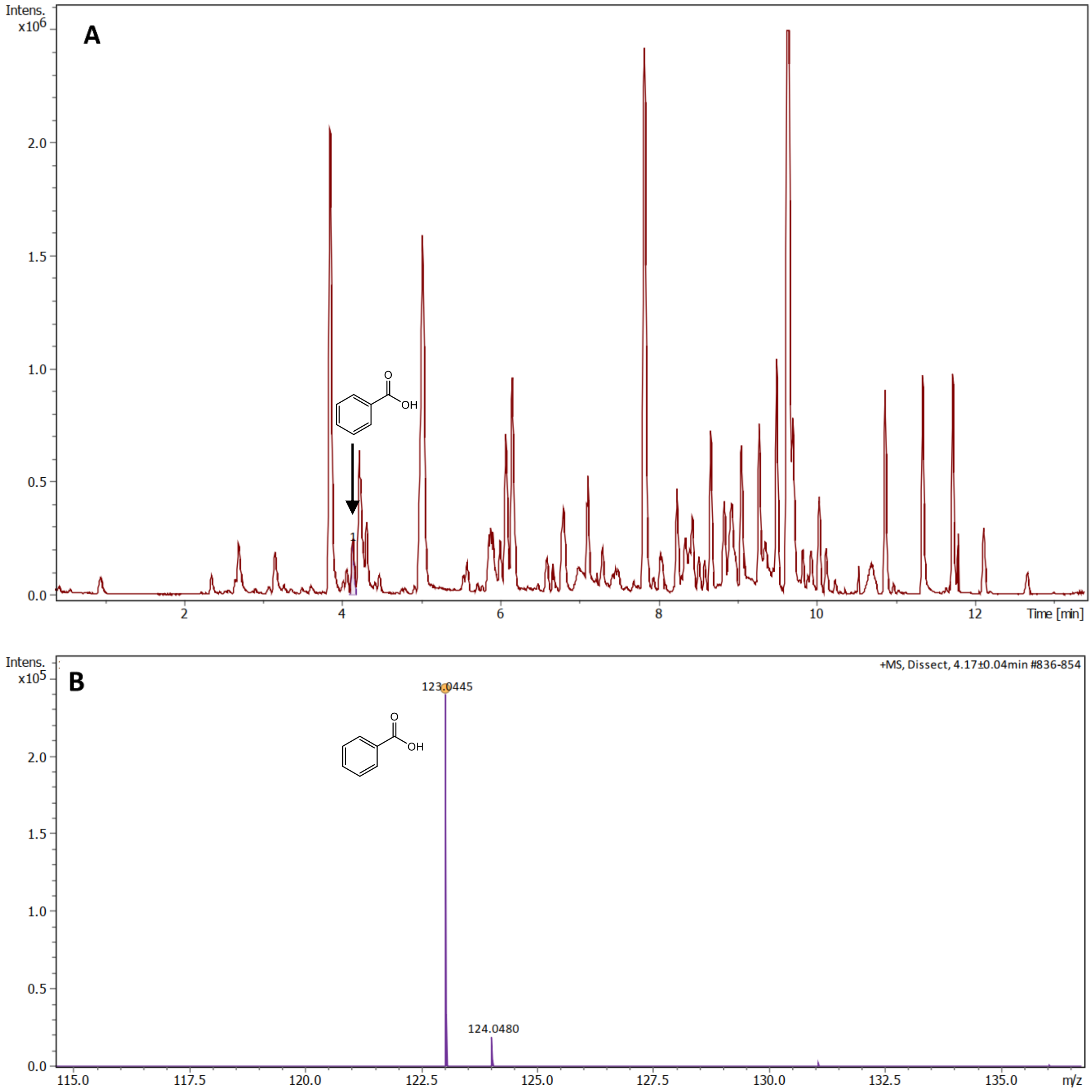

Supplementary Fig. 72. Results of LC-MS analysis of <u>trap 2</u> solution of experiment **1.2** (cf. Supplementary **Table 26**) after 120 h of heating. **A** – chromatogram of the experiment. **B** – mass spectrum of peak #1 (R_t = 4.1 min): benzoic acid ($C_7H_6O_2$). All other peaks result from over-phenylation of benzoic acid and reaction of PhMgBr with THF, cf. Supplementary **Fig. 82**.

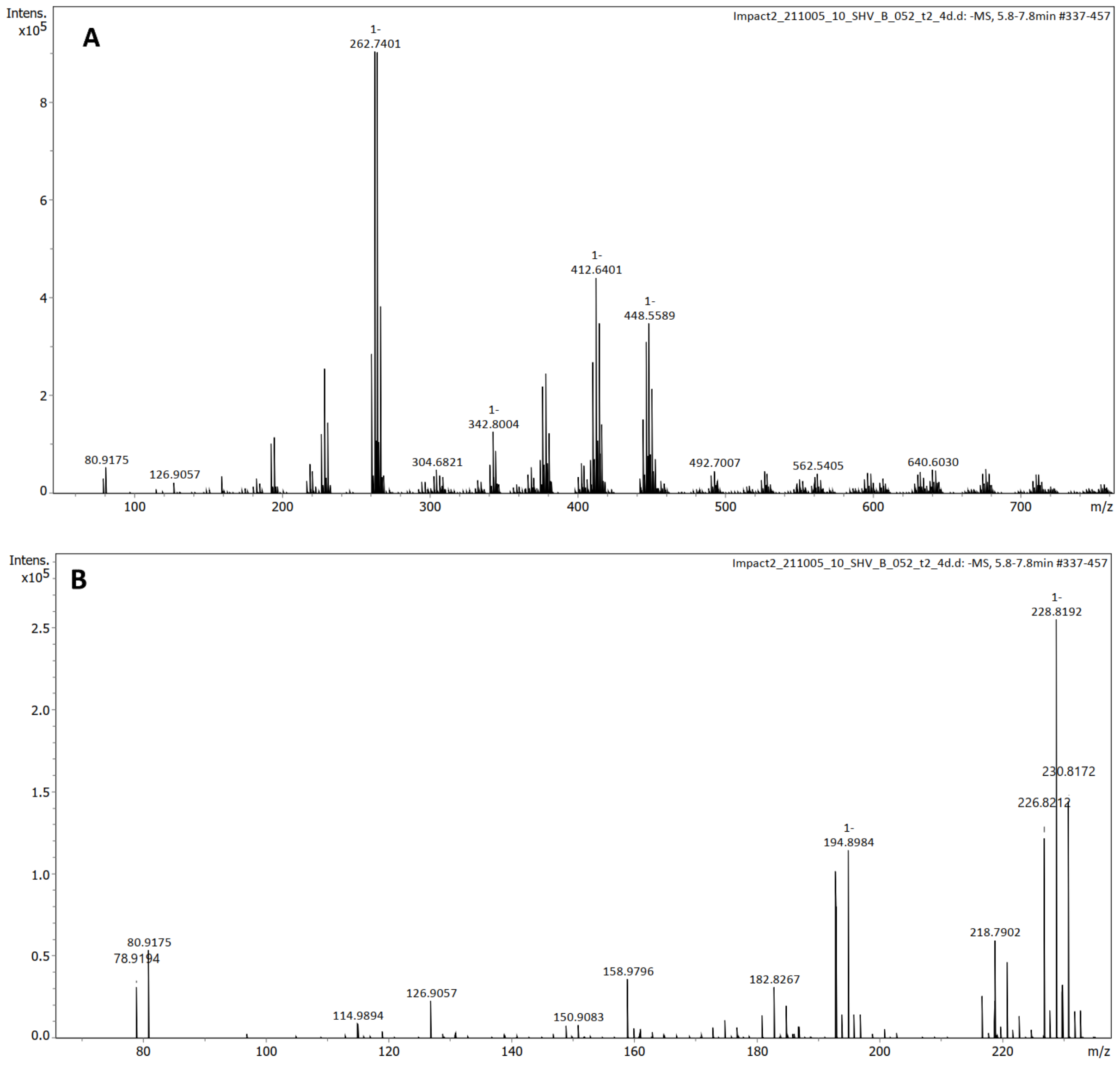

Supplementary Fig. 73. Results of high-resolution negative ion mode mass analyses of trap 2 of experiment **1.3** (cf. Supplementary **Table 26**) after 120 h of heating. **A** – chromatogram of the experiment. **B** – zoom in the area *m/z* 70-240. Benzoic acid is absent due to different phosphorylation mechanism in the reaction with formamide (**3b**).

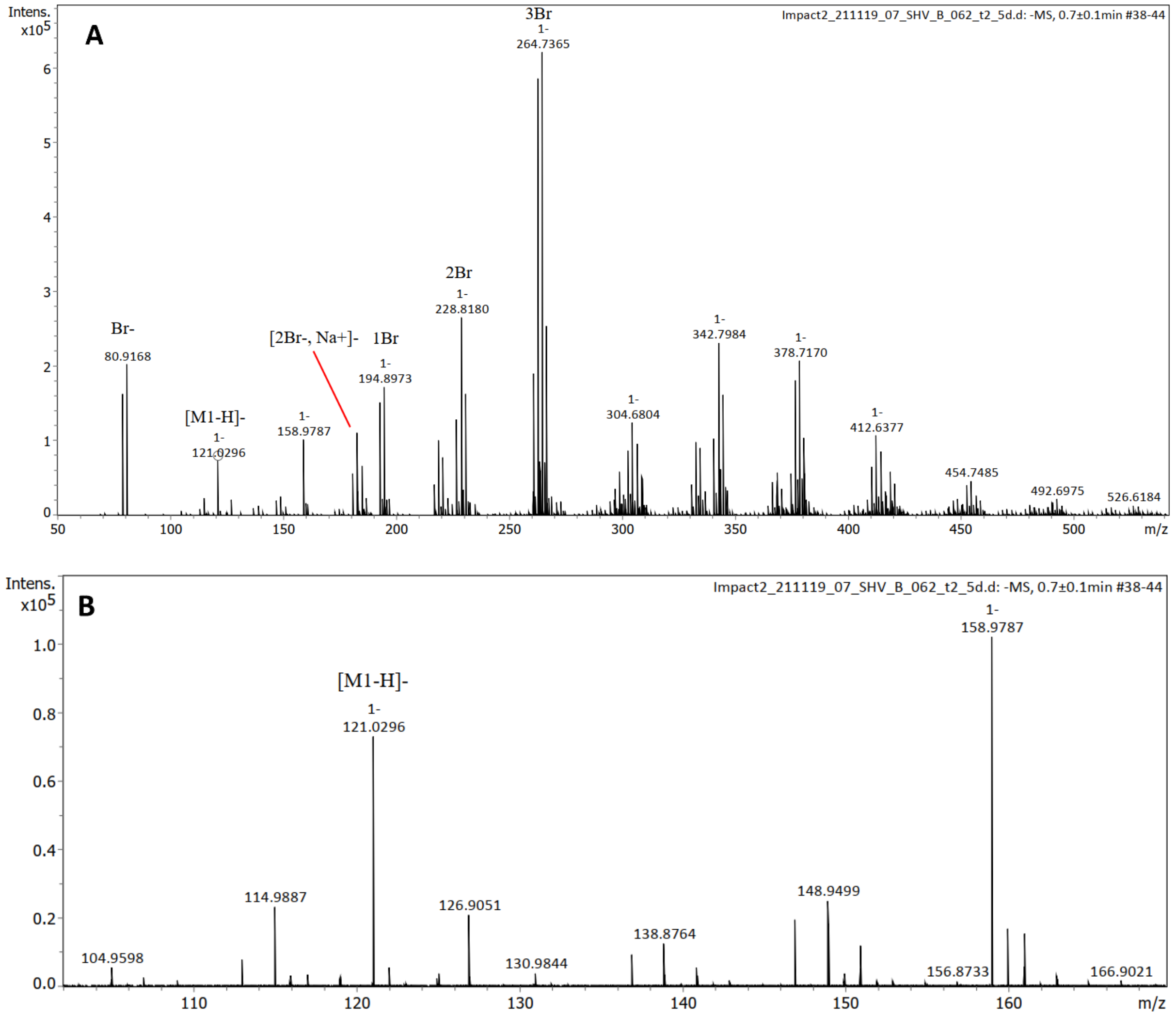

Supplementary Fig. 74. Results of high-resolution negative ion mode mass analyses of trap 2 of experiment **1.4** (cf. Supplementary **Table 26**) after 120 h of heating. **A** – chromatogram of the experiment. **B** – zoom in the area *m/z* 100-170. M1 = benzoic acid, see Supplementary **Fig. 71**.

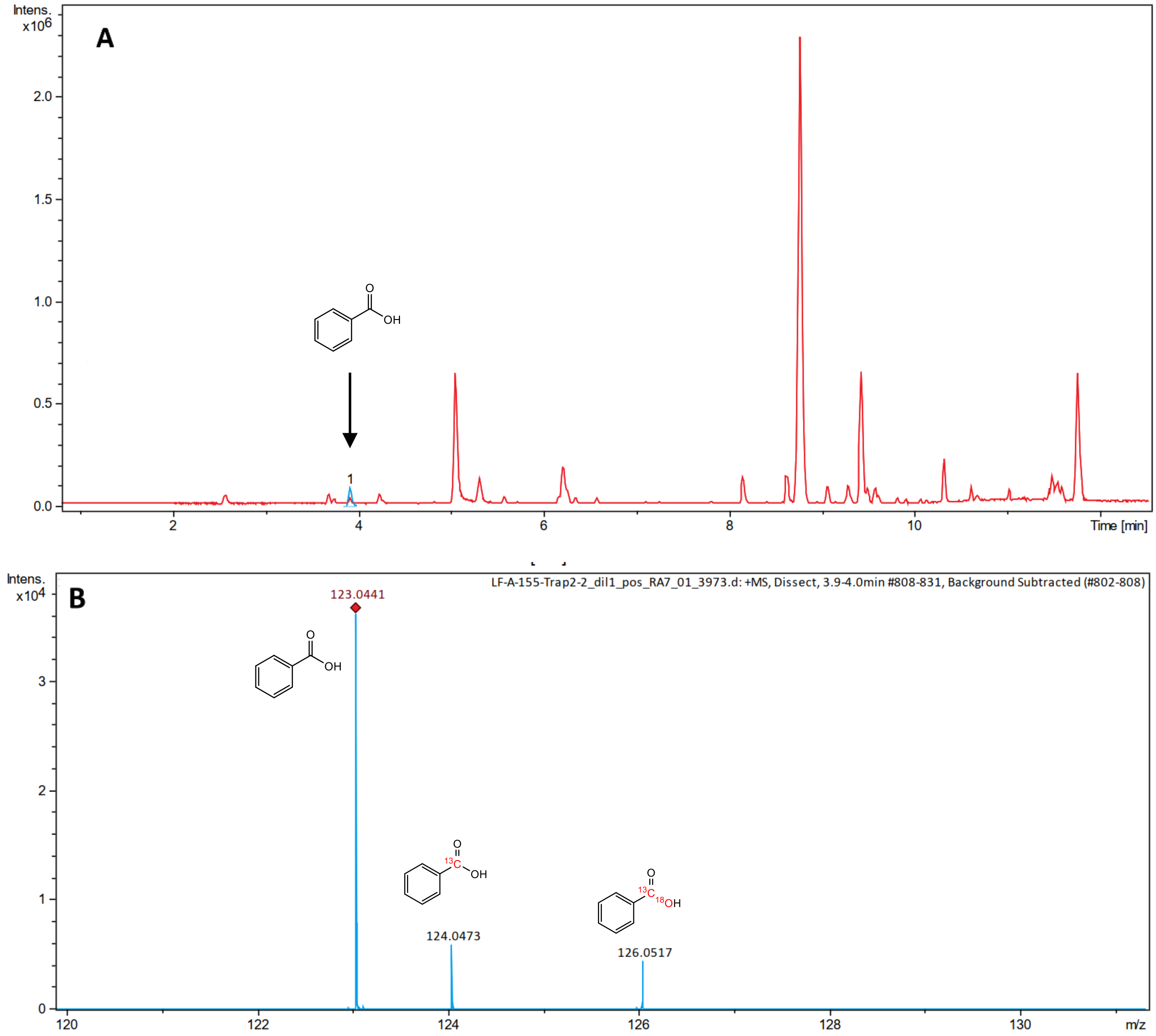

Supplementary Fig. 75. Results of LC-MS analysis of <u>trap 2</u> solution of experiment **1.5** (cf. Supplementary **Table 26**) after 120 h of heating. **A** – chromatogram of the experiment. **B** – mass spectrum of peak #1 (R_t = 4.1 min): benzoic acid (isotopologs $C_7H_6O_2$, $C_6H_6O_2{}^{13}C$ and $C_6H_6O{}^{13}C{}^{18}O$). All other peaks result from overphenylation of benzoic acid and reaction of PhMgBr with THF.

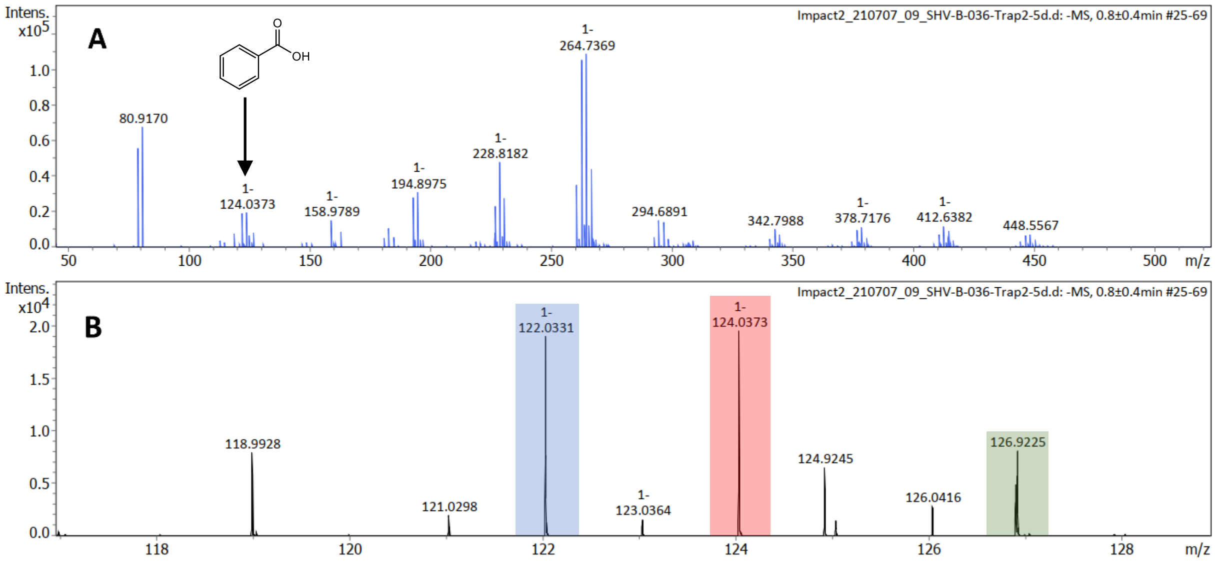

Supplementary Fig. 76. Results of high-resolution negative ion mode mass analyses of trap 2 of experiment **1.8** (cf. Supplementary **Table 26**). **A** – total ion chromatogram after 120 h of heating. **B** – zoom into benzoic acid area [^{13}C]benzoic acid [M–H]$^{-}$ *m/z* 122.0331 marked blue, [^{13}C^{18}O]benzoic acid [M–H]$^{-}$ *m/z* 124.0373 marked red, [^{13}C^{18}O$_2$]benzoic acid [M–H]$^{-}$ *m/z* 126.0416 marked green.

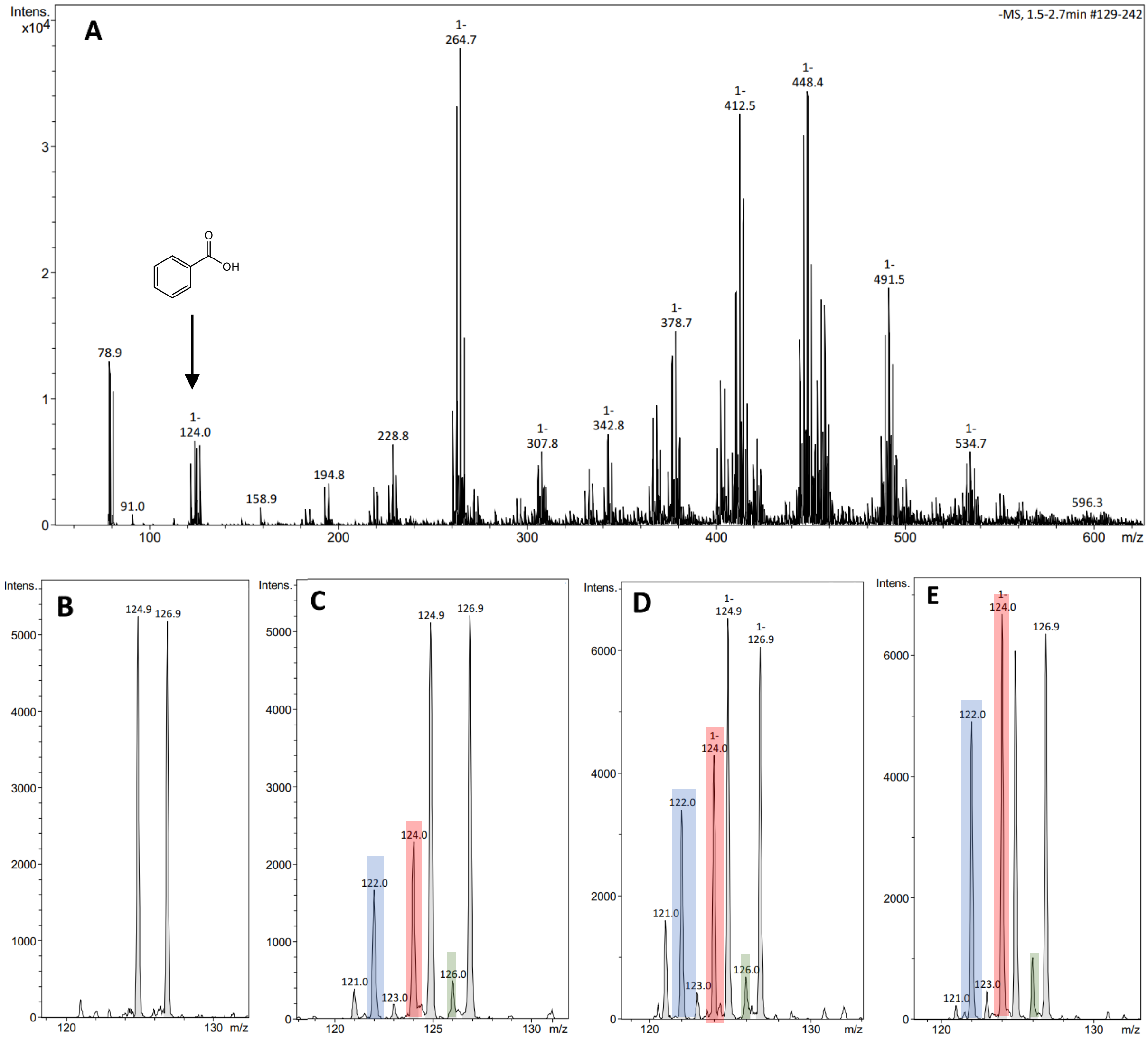

Supplementary Fig. 77. Results of low-resolution negative ion mode mass analyses of <u>trap 2</u> of experiment **1.9** (cf. Supplementary **Table 26**). **A** – total ion chromatogram after 120 h of heating. **B**-**E** – the solution was sampled after 24, 48, 96 and 120 h after the start of the heating (sampling directly from the trap 2 during the reaction). Blue color [^{13}C]benzoic acid [M–H]$^-$ *m/z* 122.0330), red [^{13}C^{18}O]benzoic acid [M–H]$^-$ *m/z* 124.0371, green [^{13}C^{18}O$_2$]benzoic acid [M–H]$^-$ *m/z* 126.0410.

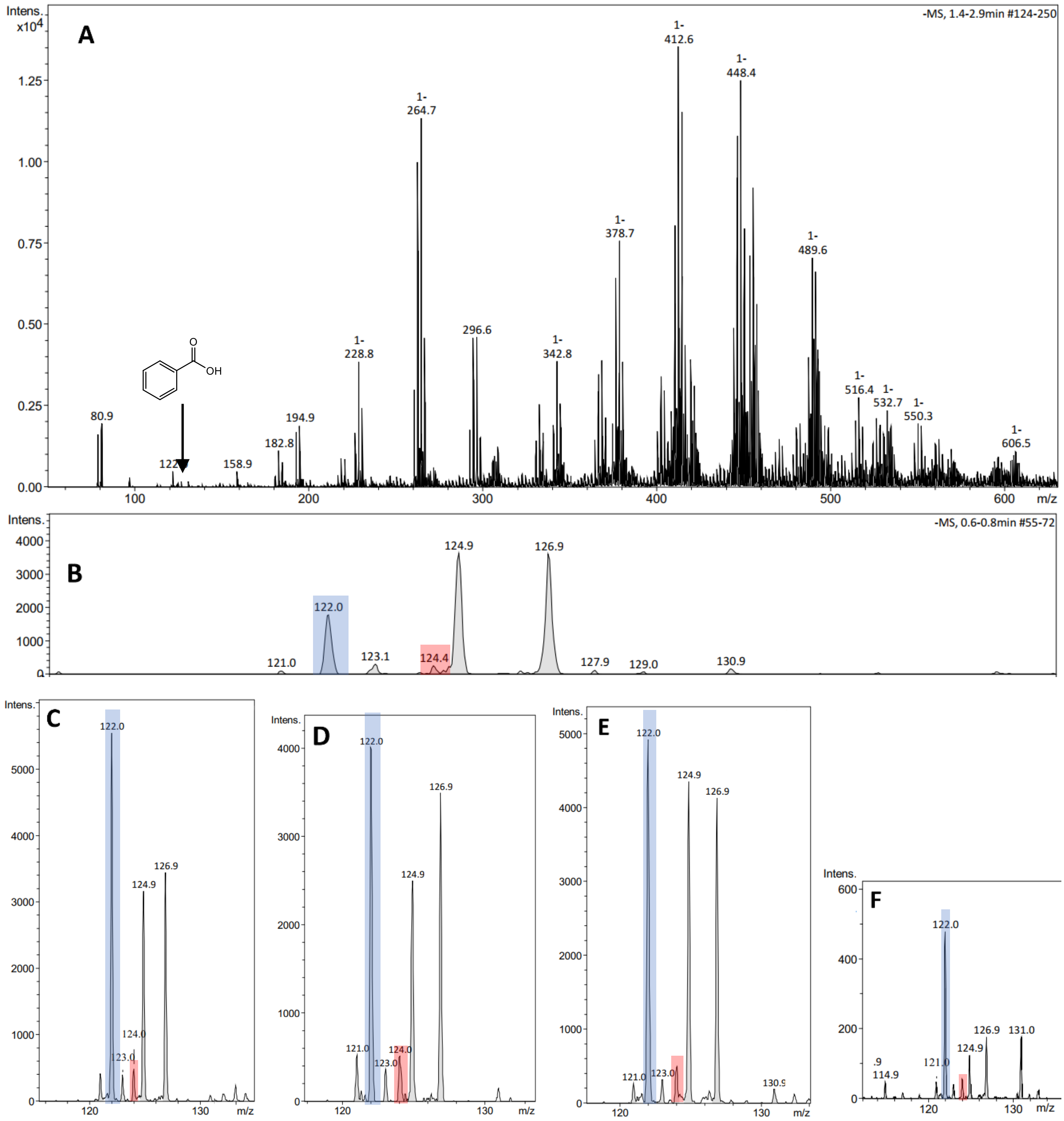

Supplementary Fig. 78. Results of low-resolution negative ion mode mass analyses of trap 2 of experiment **1.10** (cf. Supplementary **Table 26**). **A** – total ion chromatogram after 120 h of heating. **B**-**F** – the solution was sampled after 24, 48, 72, 96 and 120 h after start of the heating (sampling directly from the trap 2 during the reaction). Blue color [^{13}C]benzoic acid [M–H]$^{–}$ *m/z* 122.0330), red [^{13}C^{18}O]benzoic acid [M–H]$^{–}$ *m/z* 124.0371).

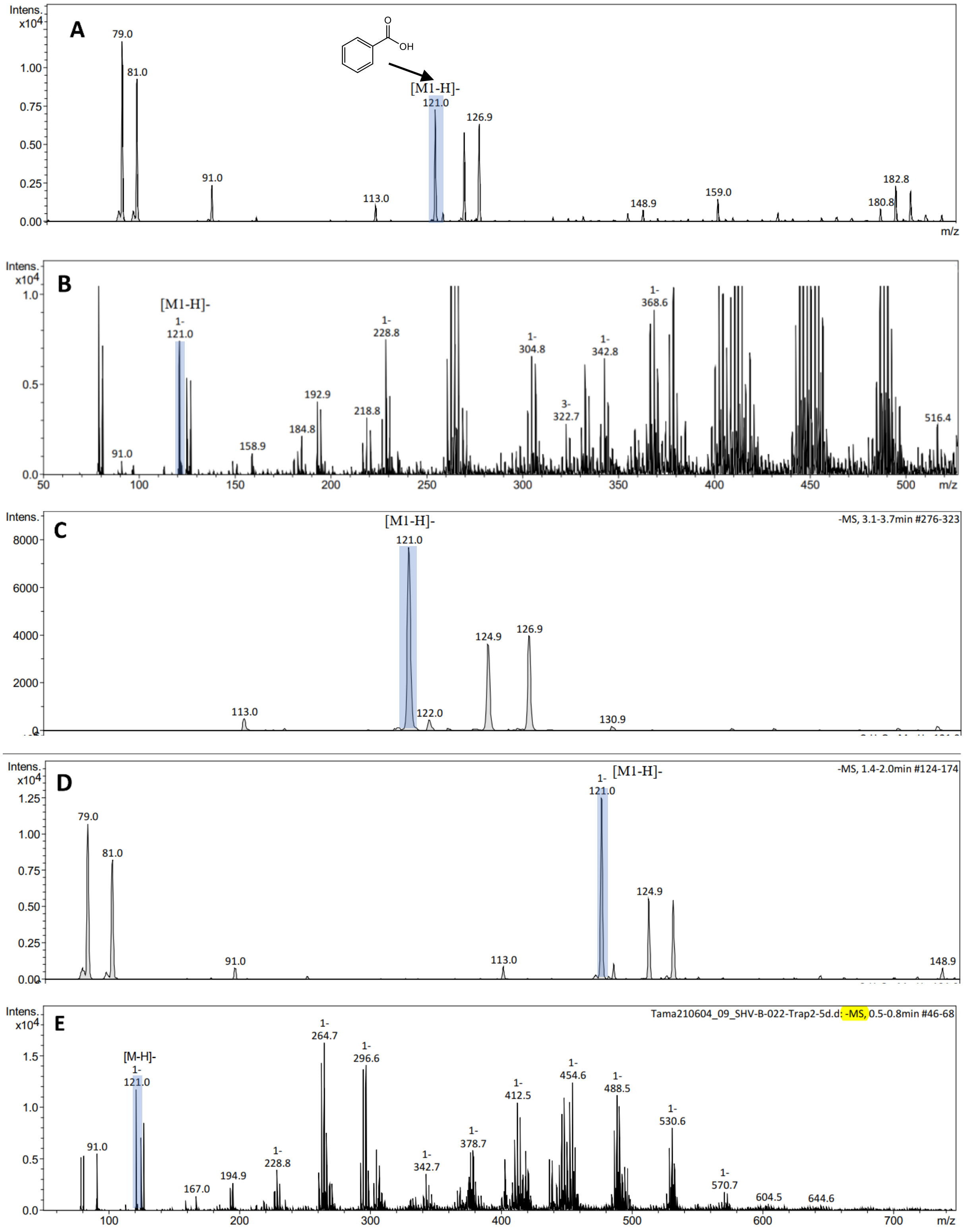

Supplementary Fig. 79. Results of low-resolution negative ion mode mass analyses of trap 2 of experiment **1.11** (cf. Supplementary **Table 26**). **A**-**E** – the solution was sampled after 24, 48, 72, 96 and 120 h after start of the heating (sampling directly from the trap 2 during the reaction). Blue color benzoic acid [M–H]$^-$ *m/z* 122.0330).

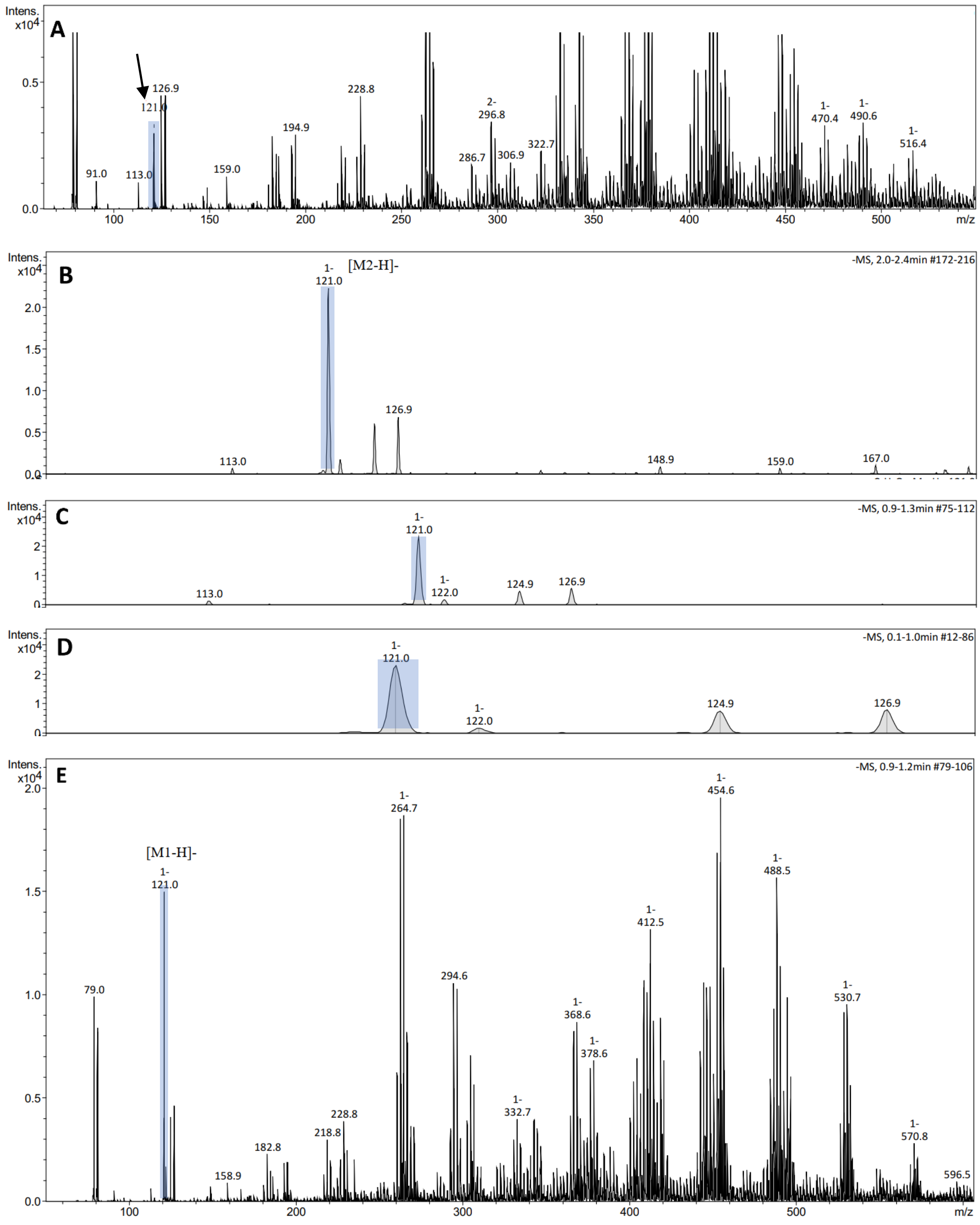

Supplementary Fig. 80. Results of low-resolution negative ion mode mass analyses of trap 2 of experiment **1.12** (cf. Supplementary **Table 26**). **A**-**E** – the solution was sampled after 24, 48, 72, 96 and 120 h after start of the heating (sampling directly from the trap 2 during the reaction). Blue color benzoic acid [M–H]$^-$ *m/z* 122.0330).

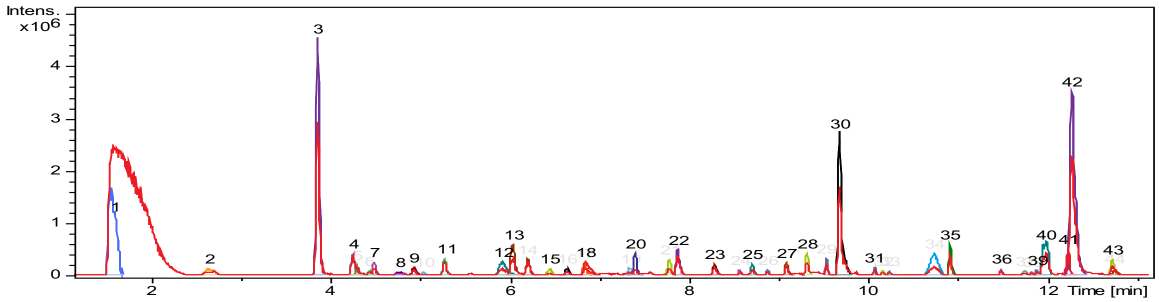

Supplementary Fig. 81. Total positive-ion chromatogram (red line) and ion-extracted peaks (numbered coloured) from LC-HRMS analysis of <u>trap 2</u> solution of experiment **1.1**, i.e., trapped electrophilic gasses evaporating from neat MPG (**6**), [$^{15}N_2$]urea (**2b**) and **[$^{18}O_4$]P$_i$** heated at 115°C for 120 h (cf. Supplementary **Table 26**).

Supplementary Table 31. List of ions identified in chromatogram shown in Supplementary **Fig. 81**. Sum formulas suggested by the "automagical signal assignment" tool (Bruker software). Natural isotope abundancies except when isotopolog ratio given. ^{18}O-enrichment derived from benzoyl. Blue formula cf. Supplementary **Fig. 82**.

Peak #	Max intens. R_T (minutes)	*m/z* of molecular ion M+H$^+$	Suggested sum formula of M	Peak #	Max intens. R_T (minutes)	*m/z* of molecular ion M+H$^+$	Suggested sum formula of M
**1**	1.70	73.0648	C_4H_8O ¶	**25**	8.74	287.1430<br>289.1480	$C_{21}H_{18}O$ 62<br>$C_{21}H_{18}{}^{18}O$ 38
**2**	2.70	117.0910	$C_6H_{12}O_2$ ¶¶	**26**	8.89	228.2322	$C_{14}H_{29}NO$ §§§
**3**	3.90	149.0961	$C_{10}H_{12}O$	**27**	9.10	254.2478	$C_{16}H_{31}NO$ §§§
**4**	4.28	105.0699<br>131.0856	C_8H_8 (major) †§<br>$C_{10}H_{10}$	**28**	9.33	280.2635	$C_{18}H_{33}NO$ §§§
**5**	4.34	167.0855<br>212.1433	$C_{13}H_{10}$ §<br>$C_{15}H_{17}N$ §§	**29**	9.55	256.2635	$C_{16}H_{33}NO$ §§§
**6**	4.47	165.0900	$C_{10}H_{12}O_2$	**30**	9.69	282.2791	$C_{18}H_{35}NO$ §§§
**7**	4.53	163.1117	$C_{11}H_{14}O$	**31**	10.08	284.2948	$C_{18}H_{37}NO$ §§§
**8**	4.80	101.0961	$C_6H_{12}O$	**32**	10.17	310.3104	$C_{20}H_{39}NO$ §§§
**9**	4.96	119.0855	C_9H_{10} §	**33**	10.24	378.3155	$C_{27}H_{39}N$ §§
**10**	5.07	177.0910	$C_{11}H_{12}O_2$	**34**	10.74	419.3156	$C_{26}H_{42}O_4$
**11**	5.30	149.0961	$C_{10}H_{12}O$	**35**	10.92	394.3468	$C_{28}H_{43}N$ §§
**12**	5.94	135.0625	not found	**36**	11.48	424.4513	$C_{28}H_{57}NO$ §§§
**13**	6.06	145.1012	$C_{11}H_{12}$ §	**37**	11.74	464.4826	$C_{31}H_{61}NO$ §§§
**14**	6.23	167.0855	$C_{13}H_{10}$	**38**	11.82	452.4826	$C_{30}H_{61}NO$ §§§
**15**	6.48	279.1356	$C_{17}H_{20}O_2$	**39**	11.88	478.4982	$C_{32}H_{63}NO$ §§§
**16**	6.66	237.1274	$C_{17}H_{16}O$	**40**	11.98	504.5139	$C_{34}H_{65}NO$ §§§
**17**	6.85	221.1536	$C_{14}H_{20}O_2$	**41**	12.23	480.5139	$C_{32}H_{65}NO$ §§§
**18**	6.87	183.0804<br>185.0848	$C_{13}H_{10}O$ 60<br>$C_{13}H_{10}{}^{18}O$ 40	**42**	12.27	506.5295	$C_{34}H_{67}NO$ §§§
**19**	7.36	253.1223<br>255.1268	$C_{17}H_{16}O_2$ 63<br>$C_{17}H_{16}O^{18}O$ 37	**43**	12.72	508.5452	$C_{34}H_{69}NO$ §§§
**20**	7.42	315.1743	$C_{23}H_{22}O$ ††	**44**	12.75	534.5608	$C_{36}H_{71}NO$ §§§
**21**	7.80	331.1693<br>333.1739	$C_{23}H_{22}O_2$ 61<br>$C_{23}H_{22}O^{18}O$ 39				
**22**	7.90	243.1168	$[C_{19}H_{15}]^+$				
**23**	8.30	271.1481	$C_{21}H_{18}$ §				
**24** ‡	8.57	354.2387	$C_{18}H_{31}N_3O_4$ 18<br>$C_{18}H_{31}N_2O_4{}^{15}N$ 82				

¶ Tetrahydrofuran (main solvent) ¶¶ 3-Methoxycyclo-pentan-1-ol (from stock solvent of PhMgBr)

† Consistent with dihydropentalene

§ Unsaturated hydrocarbon, §§ aliphatic amine and §§§ amide (related homologs) of uncertain origins

†† Deoxygenated $C_{23}H_{22}O_2$

‡ ^{15}N-carbamoyl derivative of §§ or §§§

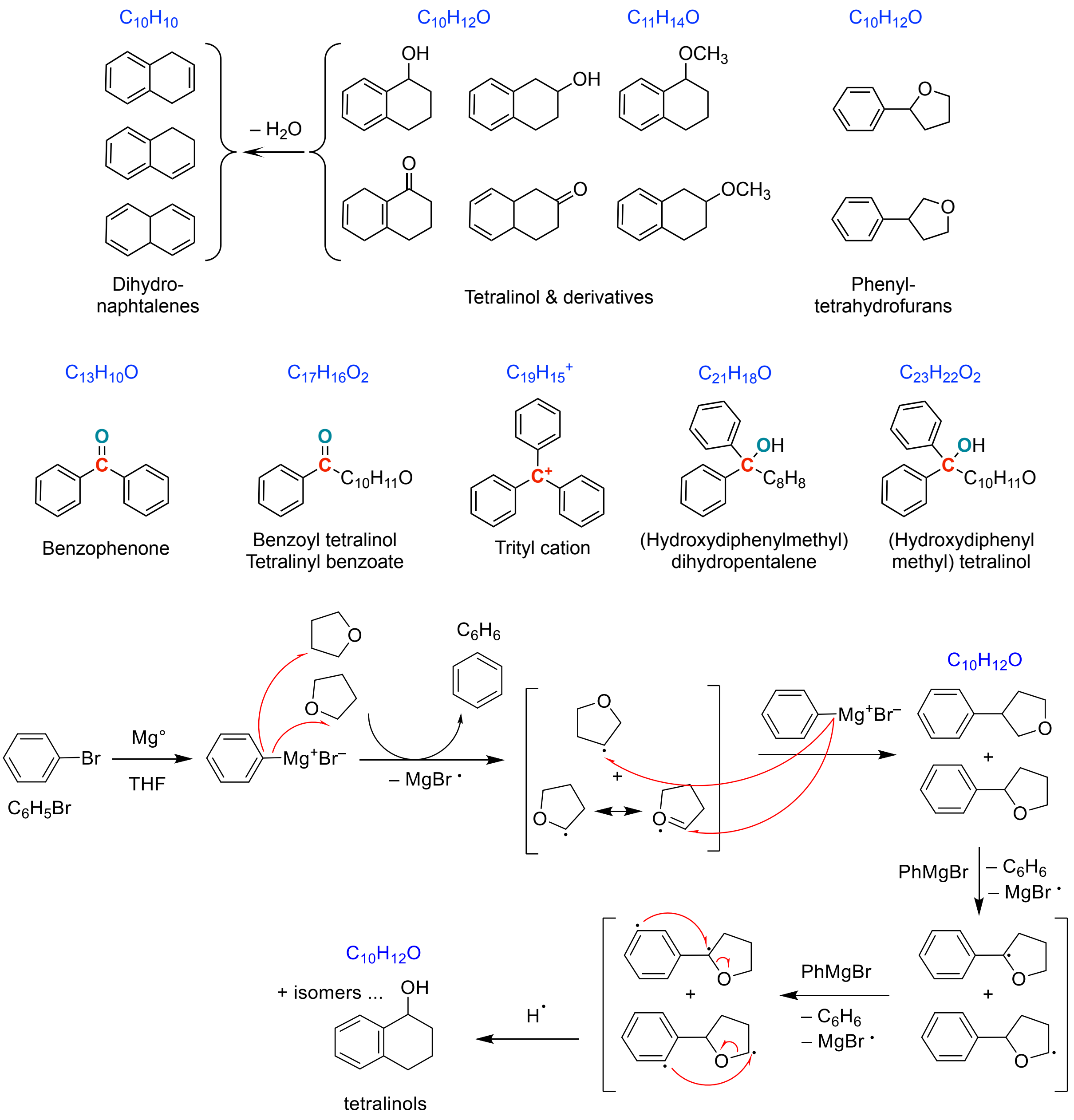

Supplementary Fig. 82. Suggested 'families' of recurrent products and proposition for the cascade of reactions that could occur in trap 2 while periodically warming from –78°C to about –50 °C for 5 days (Supplementary **Fig. 12**). ^{18}O-Enriched molecules (turquoise **O**-atom) identified in experiments where **[^{18}O$_4$]P$_i$** and [^{15}N$_2$]urea (**2b**) were used; ^{13}C-enriched molecules (red **C**-atom) were identified when [^{13}C]urea (**2c**) was used (cf. Supplementary **Table 26**).

6.3. Conclusion on tracing stable isotopes in volatiles and non-volatiles formed during the urea-assisted neat phosphorylation reactions

The isotopolog ratios of all measured compounds would allow us to determine the reaction pathway(s) of the phosphorylation reaction. In particular, if phosphate were directly dehydrated through the associative urea-catalysed mechanism, at least to some minimal extent (Supplementary **Fig. 14**), then we should obtain ^{18}O-labelled benzoic acid in the nucleophile trap 1 (Supplementary **Fig. 12A**). We have never found this compound in any test reaction that contained urea. All samples of benzoic acid from trap 1 — including neat control reactions where [^{13}C]urea had been replaced with 1-4 mol equivalents

[^{15}N]formamide (**3b**) or [^{15}N]acetamide (**4b**) to phosphorylate MPG (**6**) using $NaH_2P[^{18}O_4]$ (**[^{18}O$_4$]P**) at 115 °C giving about 7-80 % and, respectively, 5-18 % **P$_i$** consumption at 115 °C, cf. Supplementary **Table 33** in the next section — revealed a natural isotope abundance distribution within less than 0.4 % deviation from theory in each relevant *m/z* peak for $^{12}C_7{}^1H_7{}^{16}O_2$, $^{12}C_6{}^{13}C_1{}^1H_7{}^{16}O_2$ and $^{12}C_5{}^{13}C_2{}^1H_7{}^{16}O_2$ (Supplementary **Table 28**). The slightest presence of $^{12}C_7{}^1H_7{}^{16}O^{18}O$ was inconsistent with the data (Supplementary **Table 29**). This showed again that some of the residual unlabelled water, that was present in the 'dry' initial mixtures (up to 16.3 % water content by weight in MPG-containing mixtures, cf. Supplementary **Table 25**), could escape into the gas phase before reacting with cyanate and be caught in trap 1. The largest portion of benzoyl chloride (in trap 1) was however converted through ammonolysis to benzamide, or else [^{15}N]benzamide when [$^{15}N_2$]urea (**2b**), **3b** and **4b** were used. The latter two gave evidence for the thermal degradation of the carboxamides to release $^{15}NH_3$ at 115 °C (Exp. **1.3** and **1.4**, see Supplementary **Table 27**).

We did not systematically quantify the amounts of benzoic acid found in trap 2 and we never found any benzamide in this electrophile trap. This indicated that, while CO_2 would eventually be transported through trap 1 and captured in trap 2 as magnesium benzoate, later quenched with formic acid to give benzoic acid, either no isocyanic acid (bp 23.5 °C) could escape the hot reaction mixture during that time — unlike large forest wildfires or cigarette smoke[31] — or this relatively heat-resistant gas hydrolysed in the vapor phase[18,23,32]. Very low amounts HN[^{13}C]O might have remained in the basic milieu of trap 1 as ammonium salt, too difficult to get carried over to trap 2 even after 5 days, but no urea deposits from isocyanic acid and ammonia vapours could be found in the tubing after the reactor and no [^{13}C]urea (from the ammonolysis of HN[^{13}C]O) was ever detected in trap 1.

By contrast, all samples of benzoic acid from trap 2 were mixtures of different isotopologs. Only a minor fraction would contain seven ^{12}C and two ^{16}O isotopes, that is, the lightest possible isotopolog of benzoic acid, which originated from the pollution with unlabelled CO_2 from the air when taking samples from the cooled trap 2 and despite a temporarily enhanced argon flow (Supplementary **Fig. 12**). By far the largest fraction of benzoic acid in trap 2 contained one ^{13}C isotope from [^{13}C]urea (Supplementary **Table 30**). Of those, slightly less than 50 % contained two ^{16}O isotopes indicating the in situ-hydrolysis of [^{13}C]cyanate by unlabelled water that had not escaped the reaction mixture (Supplementary **Fig. 12B**). Usually, more than 40 % of [^{13}C]benzoic acid from trap 2 contained one ^{18}O isotope giving direct evidence for an operational dissociative phosphate activation mechanism through carbamoyl phosphate **CP$_i$** (Supplementary **Fig. 15**). In most of the later samples we also found lower amounts of [^{13}C,$^{18}O_2$]benzoic acid showing that, with time passing, two possible isotopomers of intermediate **CP$_i$** could eventually equilibrate with one another under these 'dry' reaction conditions (cf. pale ochre part of **Fig 4 B**). This heaviest [^{13}C,$^{18}O_2$]benzoic acid isotopolog from **CP$_i$** was absent from trap 2 in control experiments where excess unlabelled water was initially added. Nevertheless, we always observed the presence of [^{13}C,^{16}O,^{18}O]benzoic acid in trap 2 in those 'wet-and-evaporating' conditions (Exp. **1.10**, Supplementary **Table 30**).

As for the isotope-labelled non-volatiles, all major phosphorylated products in urea-assisted glycerol and MPG phosphorylation reactions showed the expected abundance of ^{18}O isotopes, i.e., mostly $R^{16}OP[^{18}O_3]^{2-}$ in phosphate monoesters and mostly $(R^{16}O)_2P[^{18}O_2]^{-}$ in cyclic and acyclic phosphodiesters (Supplementary **Table 40** and Supplementary **Table 42**). The not phosphorylated side-products, such as glyceryl carbamate and 1-(carbamoyl)glyceryl-2,3-cyclic carbonate, showed the expected natural isotope abundance but, curiously, significant ^{18}O enrichment in different non-phosphorylated side-products of dodecanol (**9**), such as dodecyl carbamate, has been found (see later, cf. Supplementary **Table 61**). This is consistent with two competing operational mechanisms under 'dry' conditions (arid environments), whereby the carbamates not only form from the direct attack of an alcohol on cyanate that had eliminated from hot urea — although, this was the only detectable pathway for fast reacting glycerol and MPG. Dodecanol was hardly phosphorylated when assisted by urea, instead some of it attacked the carbonyl group of the intermediate **CP$_i$**, see scope in Section **7.9**.

7. A scope of phosphorylation reactions in prebiotic conditions

7.1. Dependence on condensing agent of phosphorylation of glycerol (5)

First of all, we performed a “negative” control reaction in the absence of any condensing agent, where the starting alcohol was heated with phosphate. After 120 hours, we noticed that at 115 °C only glycerol (**5**) was successfully phosphorylated into acyclic phosphoric ester products (35.8 %). And even this yield is at least twice as low than in the presence of any condensing agent (60.5-96.6 %).

In the case of glycerol, we obtained organophosphates with any of the condensing agents tested at 115 °C; however, cooling down to 75 °C dramatically decreased the yield, and only reactions in the presence of urea (**2a**) and cyanamide (**1**) were significant, with slightly lower yields of 65.2 and 70.0 % after five days, respectively. A possible explanation is that high temperatures favour some thermal degradation of the carboxamides (formamides and acetamides **3a**-**4ac**) that somehow favoured phosphorylation (Supplementary **Fig. 87**). For the reaction with MPG (**6**), the presence of a condensing agent is required, and similar to glycerol, the most efficient were urea and cyanamide at both 75 and 115 °C (51.5-79.4 %), see Figures in Section **7.2**.

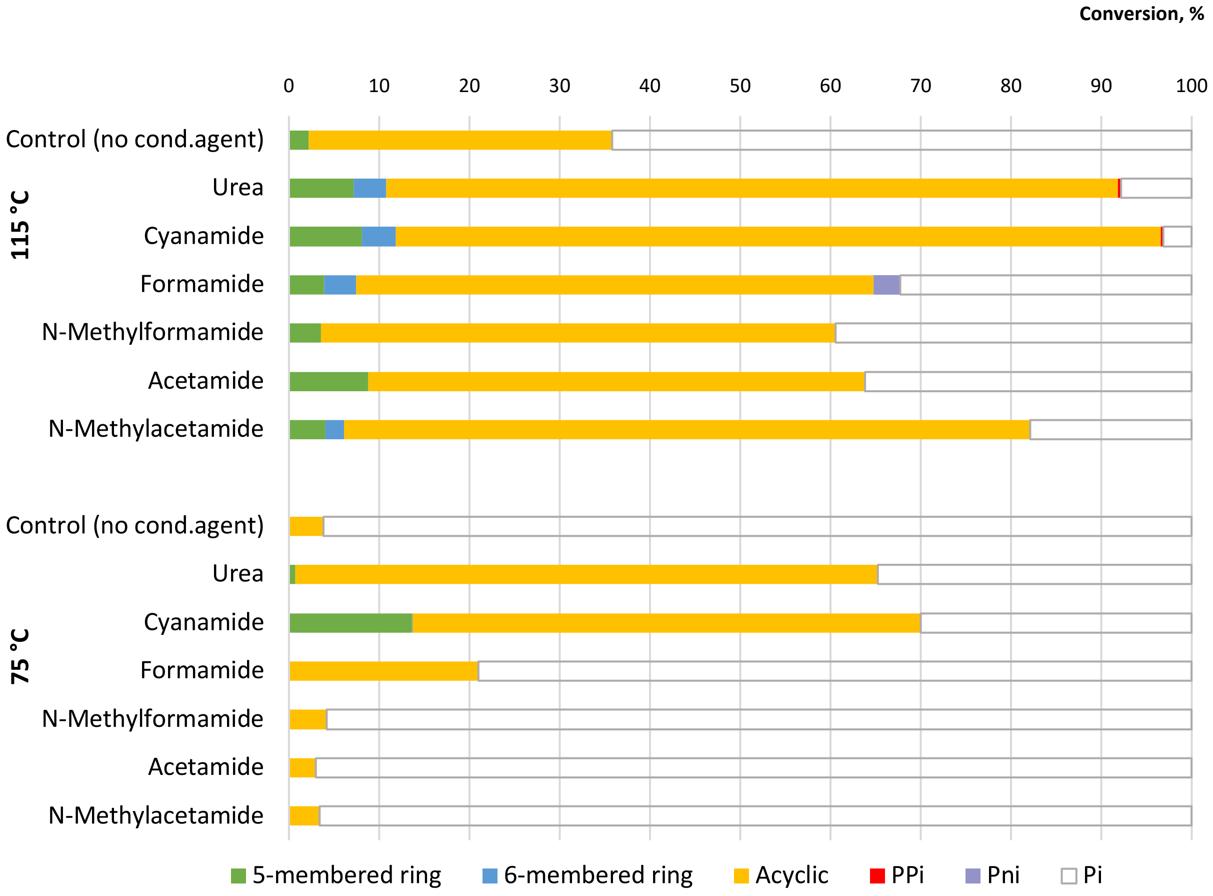

Supplementary Fig. 83. Summary of glycerol (**5**) phosphorylation after 120 h of heating at 75 and 115 °C in a 0.5 mmol scale reaction mixture containing **5, cond. agent** and $\mathbf{P_i}$ (mole ratio 1:1:1). Organic products: **5cGlP** (5-membered ring cyclic glyceryl phosphates), **GlP** (glyceryl mono-, diphosphates and di-glyceryl phosphates), **6cGlP** (6-membered ring cyclic glyceryl phosphates). Percent values from signal integration of quantitative $^{31}P\{^1H\}$ NMR spectra taken in DMSO-*d*6.

Supplementary Table 32. Data to Supplementary **Fig. 83**. Total conversion of initial amount of **P_i** to phosphorylated organic products was calculated by subtracting inorganic compound area integrals (**P_i**, **PP_i**, **P_{ni}**) from the sum of all integrated $^{31}P\{^1H\}$ NMR peak areas (in DMSO-*d*6).

**Temp. °C**	**Cond. agent**	**5cGlP %**	**6cGlP %**	**GlP %**	**P_i %**	**PP_i %**	**P_{ni} %**	**Total conversion %**
	-	2.23	-	33.58	64.19	-	-	35.81
	**2a**	7.14	3.63	81.07	7.82	0.34	-	91.84
	**1**	8.09	3.76	84.74	3.15	0.26	-	96.59
**115**	**3a**	3.93	3.50	57.30	32.29	-	2.98	64.73
	**3c**	3.53	-	57.03	39.44	-	-	60.56
	**4a**	8.79	-	55.05	36.16	-	-	63.84
	**4c**	4.03	2.10	75.96	17.91	-	-	82.09
	-	-	-	3.84	96.16	-	-	3.84
	**2a**	0.74	-	64.46	34.80	-	-	65.20
	**1**	13.60	0.07	56.31	30.02	-	-	69.98
**75**	**3a**	0.09	-	20.92	78.99	-	-	21.01
	**3c**	-	-	4.20	95.80	-	-	4.20
	**4a**	-	-	2.97	97.03	-	-	2.97
	**4c**	-	-	3.40	96.60	-	-	3.40

7.2. Dependence on condensing agent (catalyst, liquidiser) of phosphorylation of MPG (6)

Urea (**2a**) or formamide (**3a**) excess increased MPG phosphate production from 51.5 to 92.5 % and, respectively, from 6.8 to 79.7 % at 115 °C (Supplementary **Fig. 84**-Supplementary **Fig. 88**). Still higher concentrations of other carboxamides did not enhance the phosphorylation of MPG (**6**) with the maximum $\mathbf{P_i}$ consumption in the presence of 2 eq acetamide (**4a**), cf. Supplementary **Fig. 87**. Lowering the reaction temperature had a profound effect on the phosphorylation of **6**. Only either cyanamide (**1**) or urea (**2a**) allowed for significant production of MPG phosphates (almost 60-80 % $\mathbf{P_i}$ consumed) at 75 °C (Supplementary **Fig. 88**).

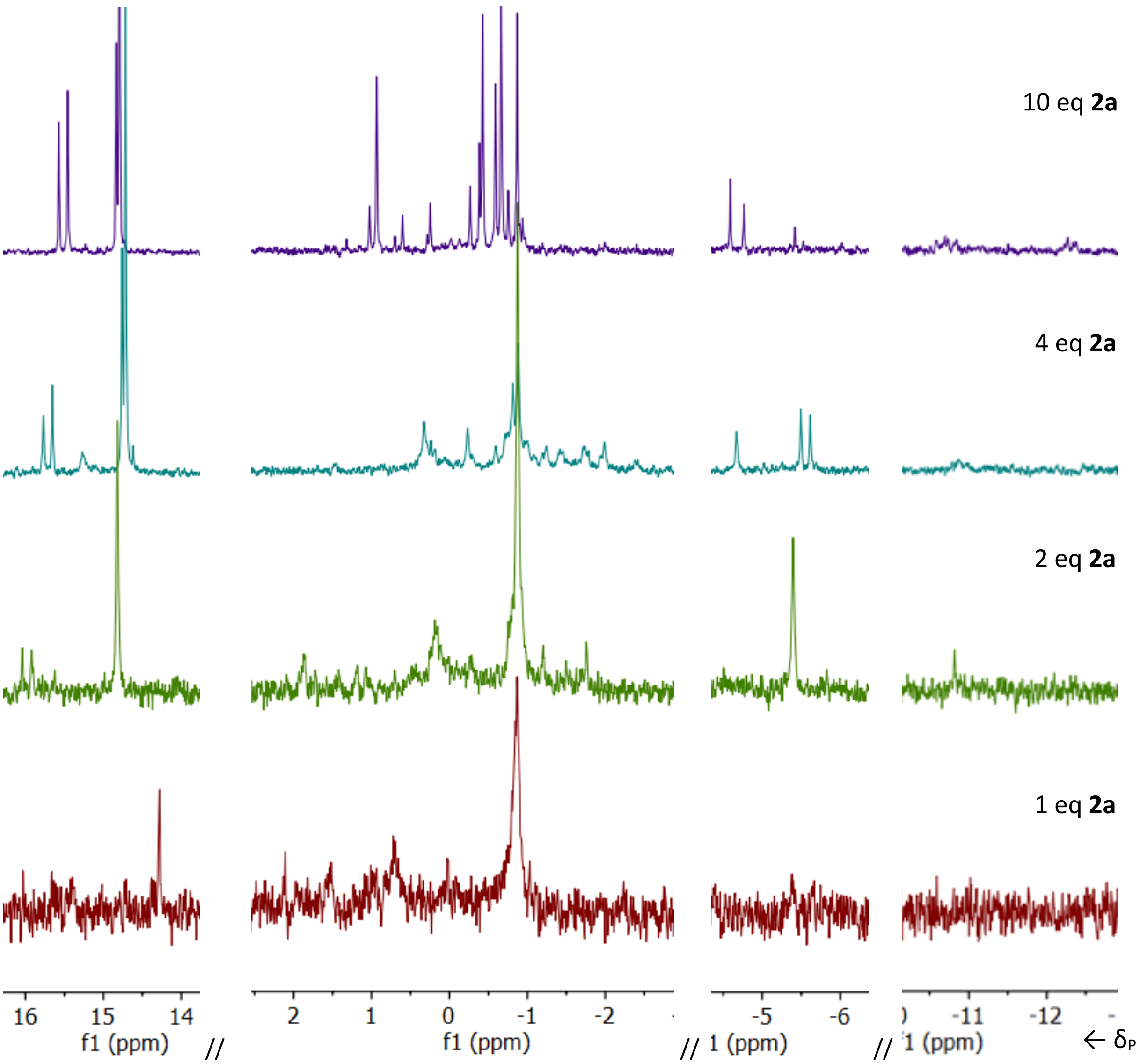

Supplementary Fig. 84. $^{31}P\{^1H\}$ NMR (202.5 MHz) of the DMSO-*d6* extracts of 0.5 mmol scale mixtures containing **6** (1 eq), **2a** (from bottom to top spectra: 1, 2, 4 and 10 eq) and $\mathbf{P_i}$ (1 eq) after heating neat at 115 °C for 120 h: δ_P = 16-14 ppm (**5cMPGP**), from 2 to –2 ppm (**MPG-1P**, **MPG-2P** and $\mathbf{[MPG]_2P}$), 0.00 ppm ($\mathbf{P_i}$), from –4.5 to –5.5 ppm (**6cMPGP**) and from –10 to –13 ppm ($\mathbf{PP_i}$). Stack centred on $\mathbf{P_i}$ singlet at δ_P = 0.00 ppm.

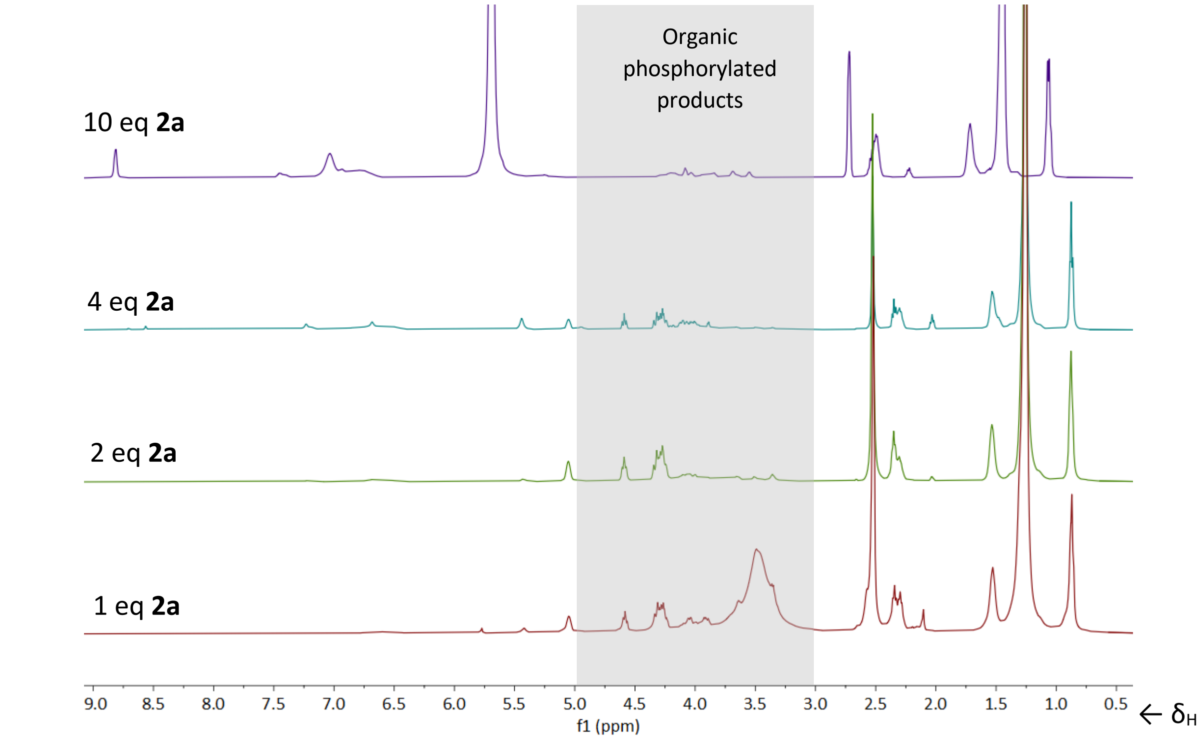

Supplementary Fig. 85. ^{1}H NMR (500 MHz) of the DMSO-*d6* extracts of 0.5 mmol scale mixtures containing **6** (1 eq), **2a** (from bottom to top spectra: 1, 2, 4 and 10 eq) and $\mathbf{P_i}$ (1 eq) after heating neat at 115 °C for 120 h: δ_H = 4.3-3.5 ppm (grey) correspond to phosphorylated products, cf. Supplementary **Fig. 86**.

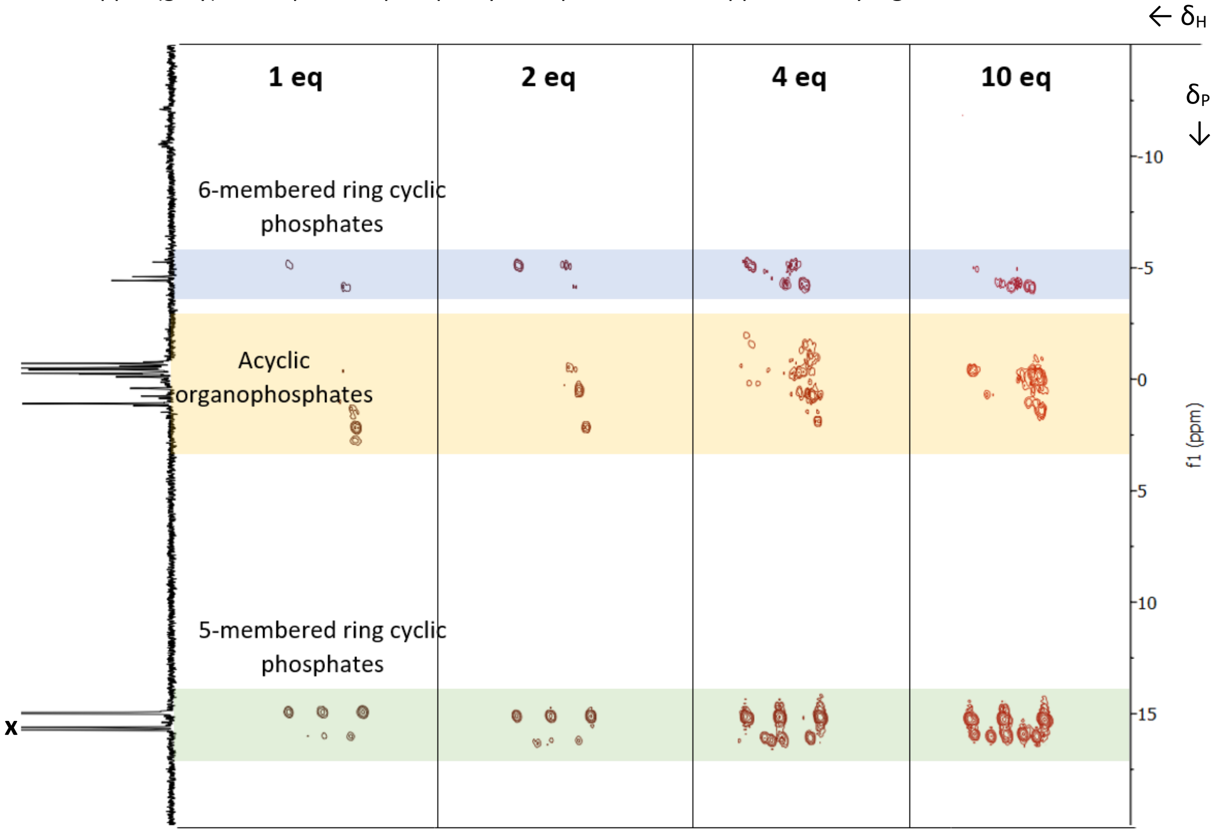

Supplementary Fig. 86. ^{1}H-$^{31}P\{^{1}H\}$ HMBC spectra (500 MHz ^{1}H [horizontal axis], δ_H = 5.0-3.0 ppm (each column), 202.5 MHz $^{31}P\{^{1}H\}$ [vertical axis], DMSO-*d6*) of the extract of mixture 0.5 mmol scale reaction of neat **6** (1 eq) : **2a** : $\mathbf{P_i}$ (1 eq) depending on molar excess of urea, reaction time 120 h at 115 °C. The ppm values of each of the 4 columns (X-axis) are depicted beneath the grey zone in Supplementary **Fig. 85**. **x** = **5cGlP**.

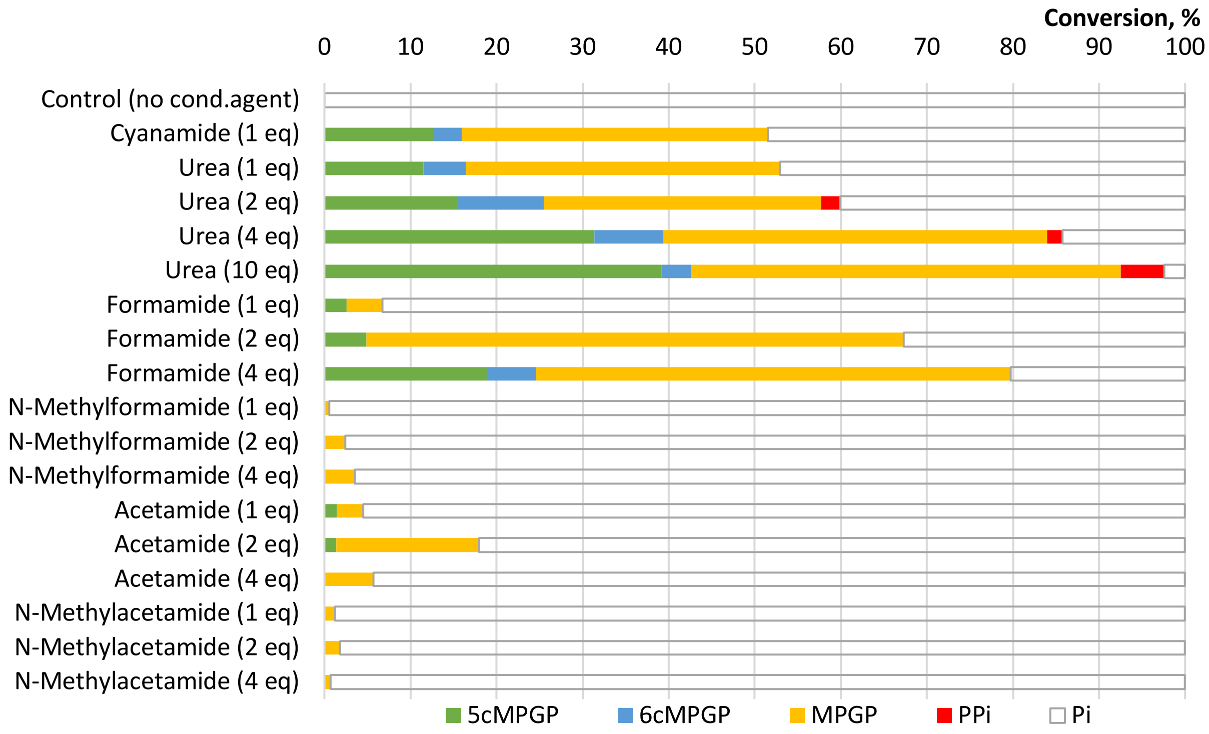

Supplementary Fig. 87. Summary of MPG (**6**) phosphorylation after 120 h of heating at 115 °C depending on molar excess of condensing agent (0-10 eq) on a 0.5 mmol scale. Molar equivalents are indicated in the diagram with respect to **6** : $\mathbf{P_i}$ (1:1 eq). Percent values from peak area integration of quantitative $^{31}P\{^1H\}$ NMR spectra (Supplementary **Fig. 84**).

Supplementary Table 33. Data to Supplementary **Fig. 87**. Total conversion of initial amount of $\mathbf{P_i}$ to phosphorylated organic products was calculated by subtracting inorganic compound integrals ($\mathbf{P_i}$, $\mathbf{PP_i}$) from the sum of all integrated $^{31}P\{^1H\}$ NMR peak areas (in DMSO-*d6*).

Cond. agent	Mol eq. cond. agent	5cMPGP %	6cMPGP %	MPGP %	P_i, %	PP_i, %	Total conversion, %
**-**	-	-	-	-	100.00	-	-
**1**	1	12.70	3.25	35.58	48.47	-	51.53
**2a**	1	11.50	4.92	36.55	47.03	-	52.97
**2a**	2	15.56	9.92	32.26	40.05	2.21	57.74
**2a**	4	31.35	8.08	44.54	14.27	1.76	83.97
**2a**	10	39.17	3.47	49.89	2.42	5.05	92.53
**3a**	1	2.61	-	4.14	93.25	-	6.75
**3a**	2	4.85	-	62.43	32.72	-	67.28
**3a**	4	18.85	5.72	55.13	20.30	-	79.70
**3c**	1	-	-	0.56	99.44	-	0.56
**3c**	2	-	-	2.45	97.55	-	2.45
**3c**	4	-	-	3.53	96.47	-	3.53
**4a**	1	1.48	-	3.06	95.46	-	4.54
**4a**	2	1.35	-	16.65	82.00	-	18.00
**4a**	4	-	-	5.68	94.32	-	5.68
**4c**	1	-	-	1.26	98.74	-	1.26
**4c**	2	-	-	1.87	98.13	-	1.87
**4c**	4	-	-	0.69	99.31	-	0.69

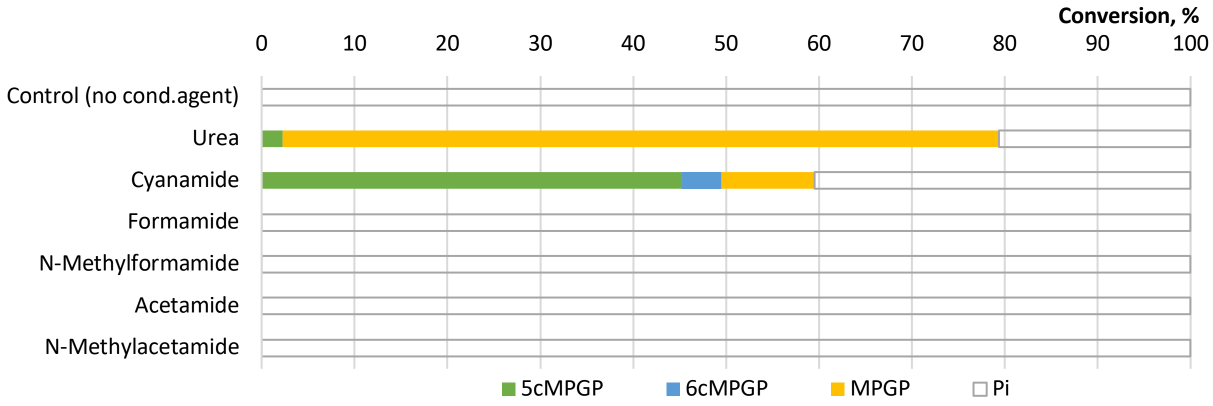

Supplementary Fig. 88. Summary of MPG (**6**) phosphorylation after 120 h of heating at 75 °C of **6**, **cond. agent** and $\mathbf{P_i}$ (1:1:1) on a 0.5 mmol scale depending on condensing agent. Organic products: **5cMPGP** (5-membered ring cyclic MPG phosphates), **MPGP** (MPG mono-, diphosphates and di-MPG phosphates), **6cMPGP** (6-membered ring cyclic MPG phosphates). Percent values from signal integration of quantitative $^{31}P\{^1H\}$ NMR spectra taken in DMSO-*d*6.

Supplementary Table 34. Data to Supplementary **Fig. 88**. Total conversion of initial amount of $\mathbf{P_i}$ to phosphorylated organic products was calculated by subtracting inorganic compound integrals ($\mathbf{P_i}$, $\mathbf{PP_i}$) from the sum of all integrated $^{31}P\{^1H\}$ NMR peak areas (in DMSO-*d*6).

Cond. agent	5cMPGP %	6cMPGP %	MPGP %	P_i %	PP_i %	Total conversion %
**-**	-	-	-	100.00	-	-
**2a**	2.24	-	77.12	20.64	-	79.36
**1**	45.20	4.28	10.03	40.49	-	59.51
**3a**	-	-	-	100.00	-	-
**3c**	-	-	-	100.00	-	-
**4a**	-	-	-	100.00	-	-
**4c**	-	-	-	100.00	-	-

7.3. Phosphorylation of MPG (6) assisted by urea and cyanamide studied by HPLC.

The efficiency of the phosphorylation reaction was compared between two condensing agents (urea and cyanamide) to see how much of the MPG reacted in both cases and quantified by evaporative light scattering detection (ELSD) after separation using reversed-phase RP-HPLC using A = H_2O + 0.1% formic acid (HCOOH) and B = acetonitrile (CH_3CN) + 0.1% HCOOH as eluants (light blue line in Supplementary **Fig. 89**).

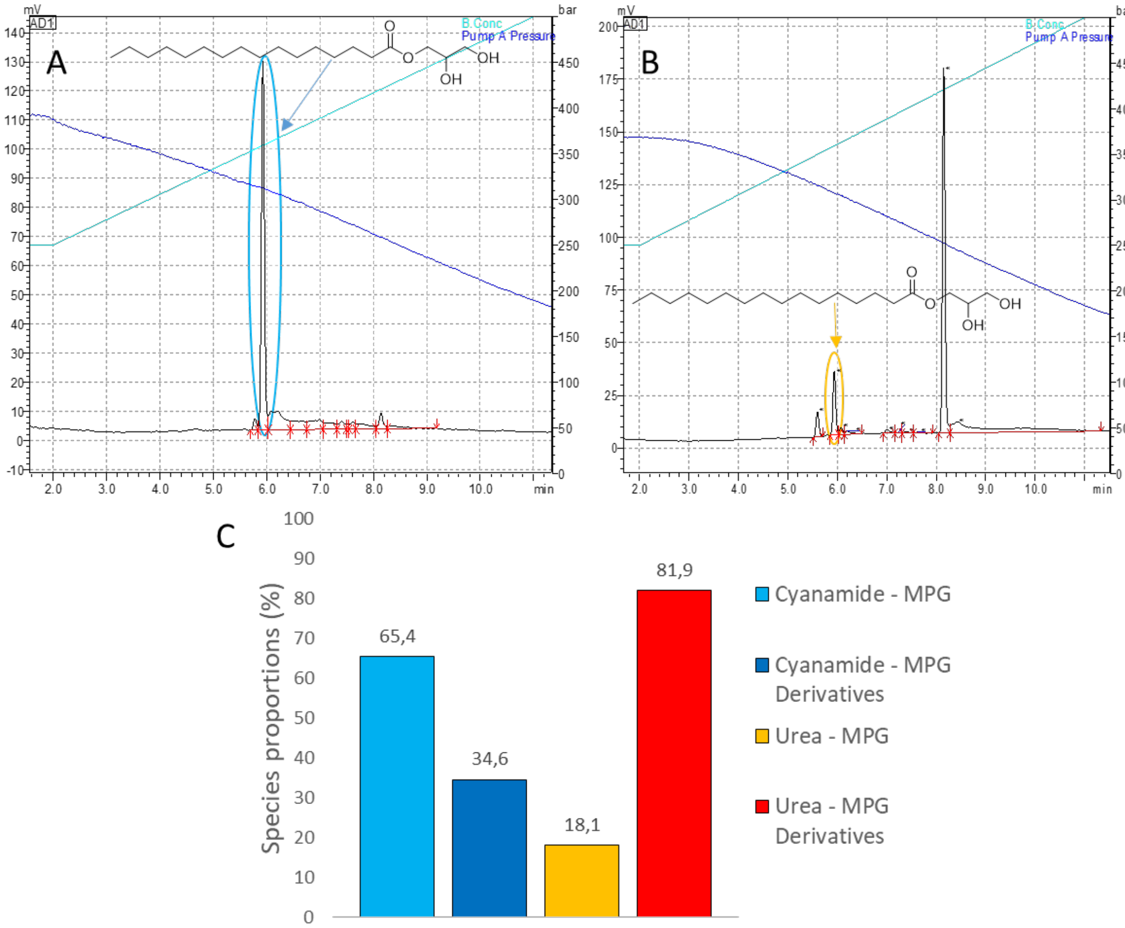

Supplementary Fig. 89. The mixtures obtained after the phosphorylation reaction of equimolar proportions of **MPG** (**6**) with **P**$_i$, in the presence of **A** – cyanamide (**1**), and **B** – urea (**2a**), were heated neat for 120 hours at 115 °C, extracted with DMSO, separated by RP-HPLC and analysed by ELSD. The peak at 6.0 min shows the starting compound (MPG). **C** – the proportions of the species obtained (MPG or MPG derivatives) at the end of the reaction were compared between both conditions (cyanamide or urea).

Using a calibration curve made for pure MPG (Supplementary **Fig. 51**), the peaks, corresponding to MPG or MPG derivatives, were integrated and quantified. In the end, the relative amounts of MPG and other derivatives could be extracted (Supplementary **Fig. 89**). It seemed clear that MPG poorly reacted in the presence of cyanamide (65 % unreacted MPG left after 5 days) when compared to urea (18 % left after 5 days). Thus, the probability to form phosphorylated species was highly increased when urea was used as a condensing agent. For this reason, further analyses were only done on reactions using urea.

The results from HPLC differ slightly from those obtained by NMR in terms of the conversion of the starting molecule. The NMR analysis was based on P$_i$, showing only the conversion of inorganic phosphate into organic phosphorylated products. However, this is different from the conversion of MPG into all other phosphorylated and not phosphorylated reaction products.

Another reason for the variation may lie in the analytical approach. While the NMR spectrum includes all molecules present in detectable quantities, HPLC depends on the type of chromatographic column used for separating reaction products. In the experiment, we used a C18 column, which is well-suited for long-chain lipids. However, it does not retain small polar glyceryl phosphates formed due to the deacylation of MPG, leading to differences in the determined quantities.

7.4. Dependence of phosphorylation of glycerol and MPG on phosphorous source

7.4.1. Effect of protonation degree of sodium orthophosphate on phosphorylation of glycerol (5)

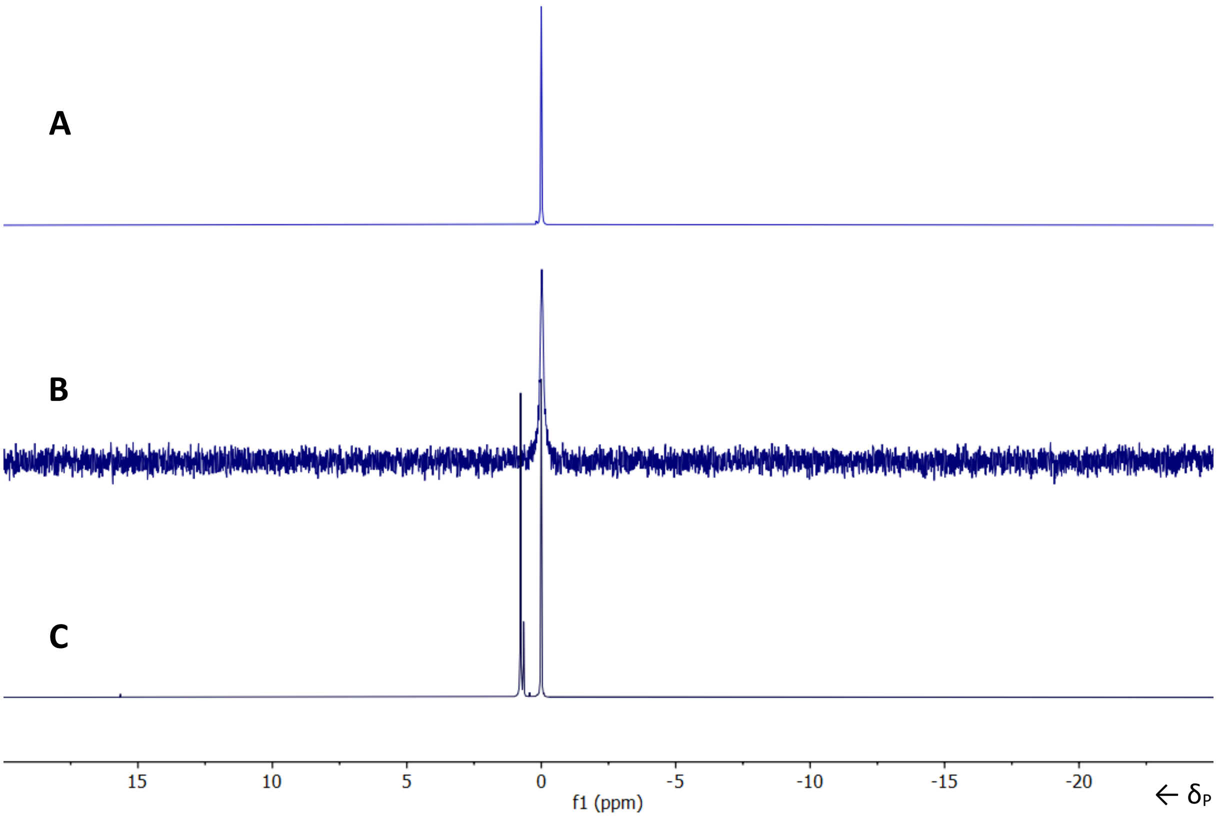

Supplementary Fig. 90. $^{31}P\{^{1}H\}$ NMR (202 MHz, DMSO-*d*6) of reaction mixture 0.5 mmol scale after 120 h of heating neat at 115°C. Spectra were referenced to the **P**$_i$ signal (0.00 ppm).

A – **5** : **Na_3PO_4** (1:1)
B – **5** : **Na_2HPO_4** = (1:1)
C – **5** : **NaH_2PO_4** = (1:1)

We compared mono-, di-, and trisodium phosphates (**P**$_i$) showing experimental pH values of equimolar mixtures with glycerol and urea in water of 4.3, 8.9 and 12.8, respectively. Significant amounts of organic phosphates were obtained only in experiments using NaH_2PO_4. The phenomenological explanation is that, the more protonated a phosphate is the more readily it can donate the phosphate group to another molecule through dehydration. Monobasic sodium dihydrogenphosphate contains more H^+ ions than the dibasic and tribasic salts. These are available to protonate the phosphate group and make it more reactive (electrophilic), for example, to produce more reactive condensed phosphates such as inorganic pyrophosphate (**PP**$_i$). In the urea-assisted phosphorylation reactions, according to the dissociative phosphate activation mechanism (Supplementary **Fig. 15**), protons are needed to eliminate ammonia from urea and carbamoyl phosphate (**CP**$_i$).

Supplementary Table 35. Results from integrating $^{31}P\{^{1}H\}$ NMR peaks of DMSO-*d*6 extract of 0.5 mmol scale mixture of **5**, **cond. agent** and $\mathbf{P_i}$ (1:1:1) after 120 h of heating neat at 115 °C depending on protonation degree of inorganic phosphate. Calculation of total conversion (conversion of initial amount of $\mathbf{P_i}$ to phosphorylated organic products) was made by subtraction of inorganic compound quantities ($\mathbf{P_i}$ and $\mathbf{PP_i}$) from the sum of all integrated peak areas.

Cond. agent	P_i	5cGlP, %	6cGlP, %	GlP, %	P_i, %	PP_i, %	Total conversion, %
**-**	$\mathbf{Na_3PO_4}$	-	-	-	100.00	-	-
**2a**	$\mathbf{Na_3PO_4}$	-	-	2.04	97.96	-	2.04
**-**	$\mathbf{Na_2HPO_4}$	-	-	-	100.00	-	-
**2a**	$\mathbf{Na_2HPO_4}$	-	-	-	100.00	-	-
**-**	$\mathbf{NaH_2PO_4}$	2.23	-	33.58	64.19	-	35.81
**2a**	$\mathbf{NaH_2PO_4}$	7.14	3.63	81.07	7.82	0.34	91.84

7.4.2. Experiments with cyclic trimetaphosphate (cTMP)

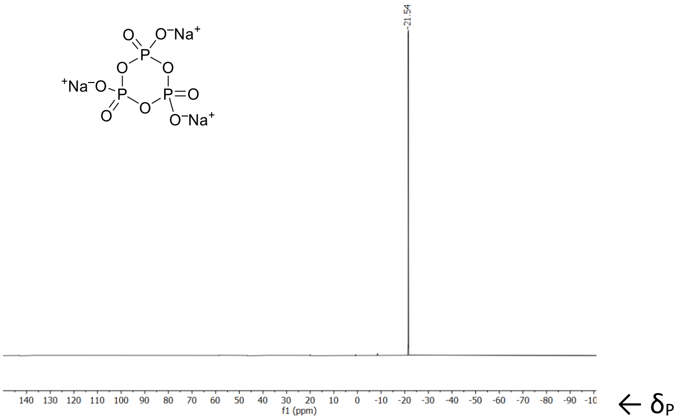

Supplementary Fig. 91. $^{31}P\{^{1}H\}$ NMR (202.5 MHz, D_2O) 0.5 mM **cTMP**. δ_P (*s*) = –21.5 ppm.

The use of equimolar amounts of **cTMP** with glycerol (**5**) gave excellent conversions to organic phosphates (71.6-98.2 %) at both temperatures 75 and 115 °C (Supplementary **Fig. 94**) even in the absence of a condensing agent. Surprisingly, heating **5** with **cTMP** and <u>no</u> added <u>urea</u> in open vessels at 115 °C provoked the formation of cyclic carbonates of diglyceryl phosphodiesters $\mathbf{diGl(CO)_2P}$ and **diGlCOP** (but not at 75 °C, see Supplementary **Fig. 92**), viz. exactly the same molecules (cf. Supplementary **Fig. 25B**) that are being generated after 8 hours of heating **5** in the presence of $\mathbf{P_i}$ and urea (**2a** and **2b**, see Supplementary **Fig. 45**, Supplementary **Fig. 46**, Supplementary **Table 12**). Since urea was lacking in this **cTMP** experiment, the only other source of carbonyl groups could have been carbon dioxide from the air. It appears that CO_2 was activated by 115 °C-hot **cTMP** and then the carbonyl group transferred to the glyceryl phosphates (Supplementary **Fig. 95**).

The presence of a condensing agent did not increase the total amount of organic phosphates very much (+ 3-8%, Supplementary **Table 36**), but more $\mathbf{diGl(CO)_2P}$, **diGlCOP** and cyclic phosphates were detected in **cTMP** reactions with cyanamide (**1**) and urea (**2a**), see Supplementary **Fig. 93**. This confirms that the additional dehydrating power provided by **1** or **2a** is used to carbamylate and then cyclise more acyclic glyceryl monophosphates into cyclic carbonates and cyclic phosphates. Owing to the fact that we registered the presence of $\mathbf{P_i}$ (0 ppm) and $\mathbf{PP_i}$ (–9.29 ppm) but hardly ever **cTMP** itself, we can conclude that the reaction conditions are always favourable for a ring-opening of **cTMP**.

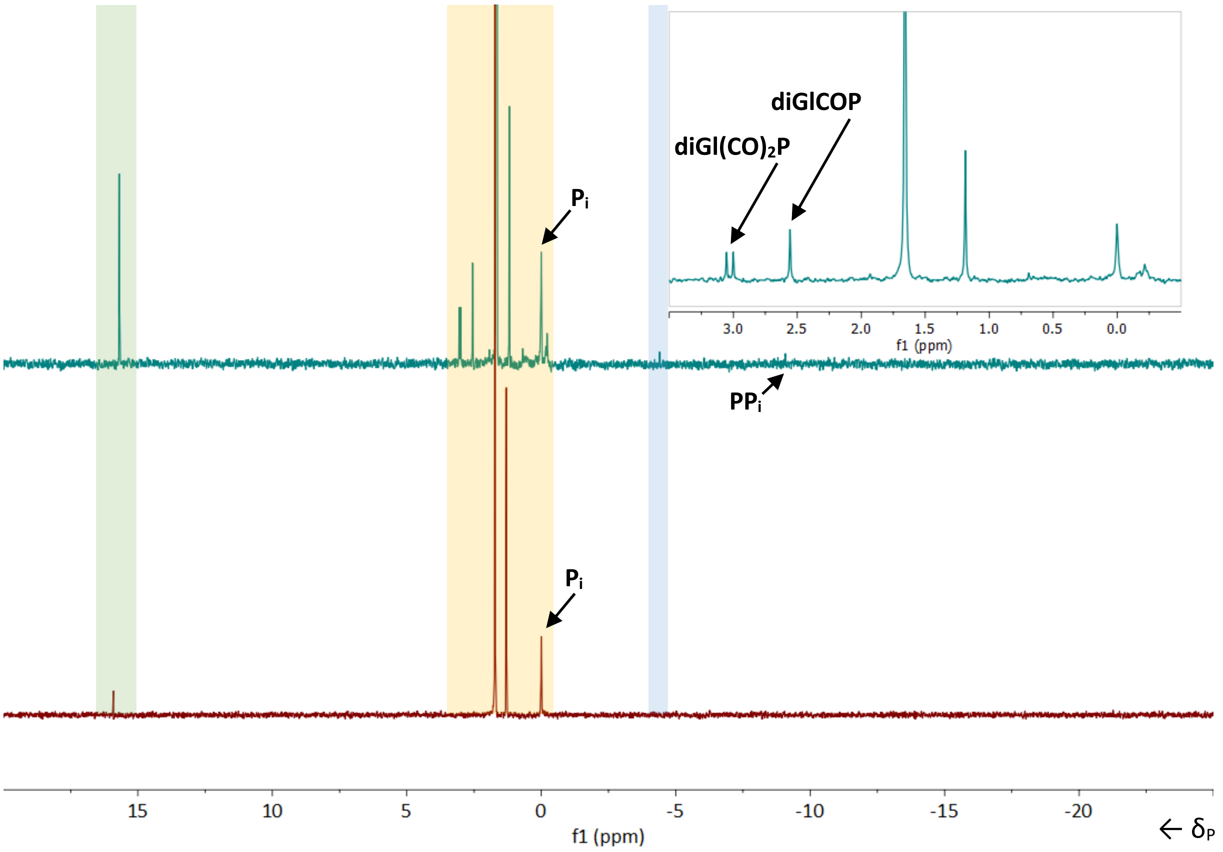

Supplementary Fig. 92. $^{31}P\{^{1}H\}$ NMR spectra (202 MHz) of crude mixture **5**:**cTMP** (1:1); 0.5 mmol scale reaction after heating for 120 h, then dissolved in DMSO-*d*6. δ_P = 16-15 ppm (**5cGlP**, green), from 3.41 to 0.54 ppm (**GlP**: glyceryl-1-phosphates, glyceryl-2-phosphates, di-glyceryl phosphates, ochre and insert), 0.00 ppm (**P$_i$**), –4.48 ppm (**6cGlP**, blue), –9.09 ppm (*s*, **PP$_i$**). **Turquoise** (upper) – heating at 115 °C; **brown** (lower) – heating at 75 °C.

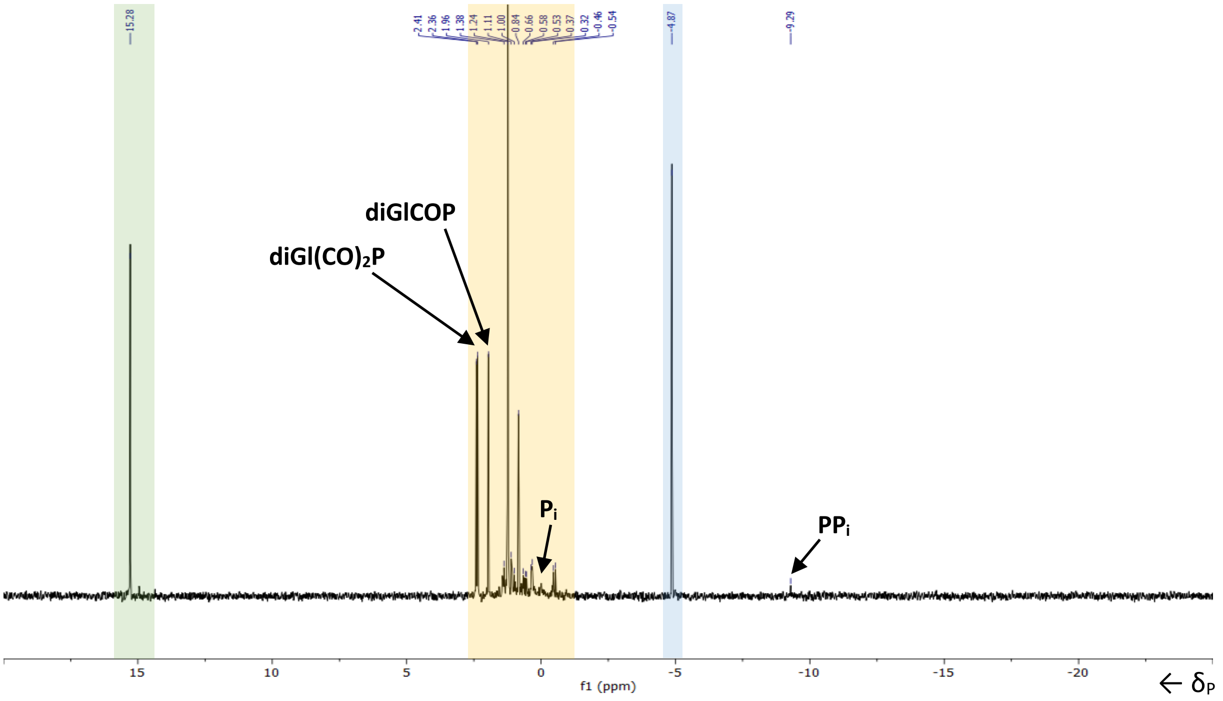

Supplementary Fig. 93. $^{31}P\{^{1}H\}$ NMR spectra (202 MHz) of crude mixture **5**:**2a**:**cTMP** (1:1:1); 0.5 mmol scale reaction after heating for 120 h at 115°C, then dissolved in DMSO-*d*6. δ_P = 15.28 ppm (**5cGlP**, green), from 2.41 to –0.54 ppm (**GlP:** di-glyceryl phosphates, glyceryl-*O*1-phosphates, glyceryl-*O*2-phosphates etc., ochre), 0.00 ppm (**P$_i$**), –4.48 ppm (**6cGlP**, blue), –9.29 ppm (*s*, **PP$_i$**).

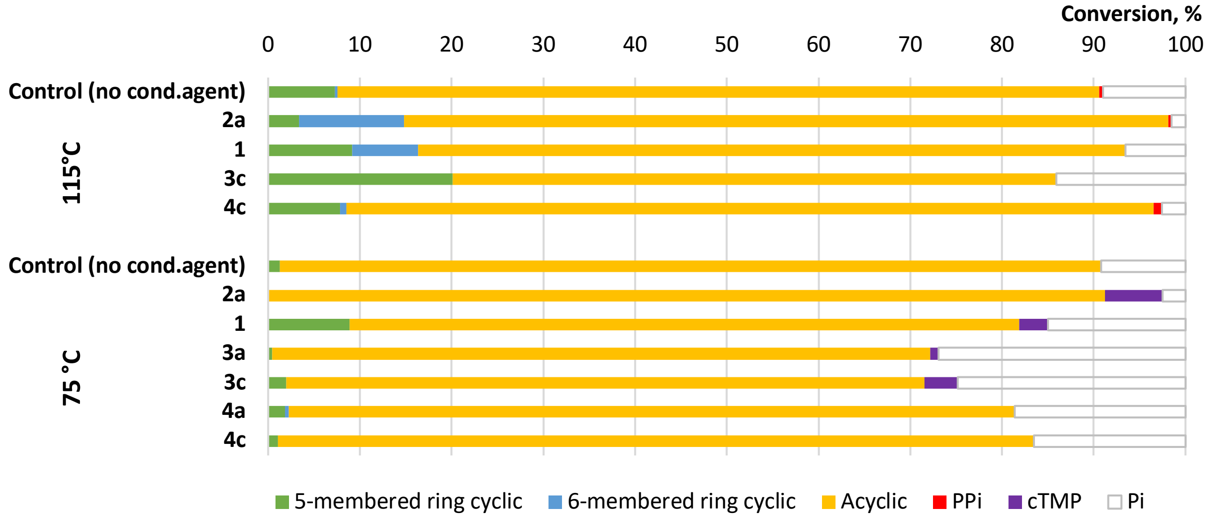

Supplementary Fig. 94. Summary of glycerol (**5**) phosphorylation after 120 h of heating at 75 and 115 °C in 0.5 mmol scale reaction mixture **5** : **cond.agent** : **cTMP** (1 : 0 or 1 : 1). Residual signal at δ_P = −20 to −25 ppm corresponds to unreacted **cTMP**.

Supplementary Table 36. Data to Supplementary **Fig. 94**. Calculation of total conversion (conversion of initial amount of **cTMP** to phosphorylated organic products) was made by subtraction of inorganic compound quantities (P_i, PP_i and **cTMP**) from the sum of all integrated $^{31}P\{^1H\}$ NMR peak areas (in DMSO-*d*6). **GlP** comprise all acyclic organic phosphate peaks except those for **diGl(CO)₂P** and **diGlCOP** (Supplementary **Fig. 92** and **67**).

Temp. °C	Cond. agent	5cGlP %	6cGlP %	diGl(CO)$_2$P %	diGlCOP %	GlP %	P_i %	PP_i %	cTMP %	Total conversion %
	-	7.25	0.30	4.60	3.99	74.43	9.01	0.41	-	90.58
	**2a**	9.38	11.40	13.02	6.57	57.78	1.49	0.36	-	98.15
**115**	**1**	9.14	7.20	18.17	10.89	48.04	6.56	-	-	93.44
	**3c**	20.11	0	7.68	0.2	57.95	14.08	-	-	85.92
	**4c**	7.87	0.66	11.99	7.02	69.02	2.55	0.89	-	96.56
	-	1.27	-	-	-	89.51	9.22	-	-	90.78
	**2a**	-	-	-	-	91.26	2.46	-	6.28	91.26
	**1**	8.85	-	-	-	73.04	14.98	-	3.13	81.89
**75**	**3a**	0.41	-	-	-	71.79	26.91	-	0.89	72.20
	**3c**	1.97	-	-	-	69.6	24.83	-	3.60	71.57
	**4a**	1.81	0.45	-	-	79.16	18.58	-	-	81.42
	**4c**	1.10	-	-	-	82.39	16.51	-	-	83.49

The fact that **cTMP** appeared to bind CO_2 in our neat 115°C-hot conditions, and transfer a carbonyl group to 1,2-diols (phosphorylated or not = P/OH), is consistent with the work of others who discovered the capacity of aqueous trisodium orthophosphate to bind CO_2[33]. We can hypothesise that neat hot **cTMP** at 115 °C activates CO_2 through a mixture of carboxylation (Supplementary **Fig. 95**, horizontal sequence) and oligomerisation of **cTMP** (vertical steps) to produce bridged through carbonyl groups (marked red) oligomers that have a (**cTMPCOcTMP**)$_n$ scaffold bearing varying degrees of peripheral carboxylation. The heat-driven carboxylation-oligomerisation process of dry **cTMP** would be accompanied by the elimination of sodium oxide from the trisodium salt of **cTMP**. The oligomeric bridging carbonyl groups could then react with nucleophiles such as 1,2-diols, whereby highly basic Na_2O would bind their protons to give water that ring-opens **cTMP** units (ring-opened **PPP**$_i$, **PP**$_i$ and **P**$_i$ not shown in the figure) while glycerol (**5**) (or MPG (**6**)) is phosphorylated and/or carbonylated:

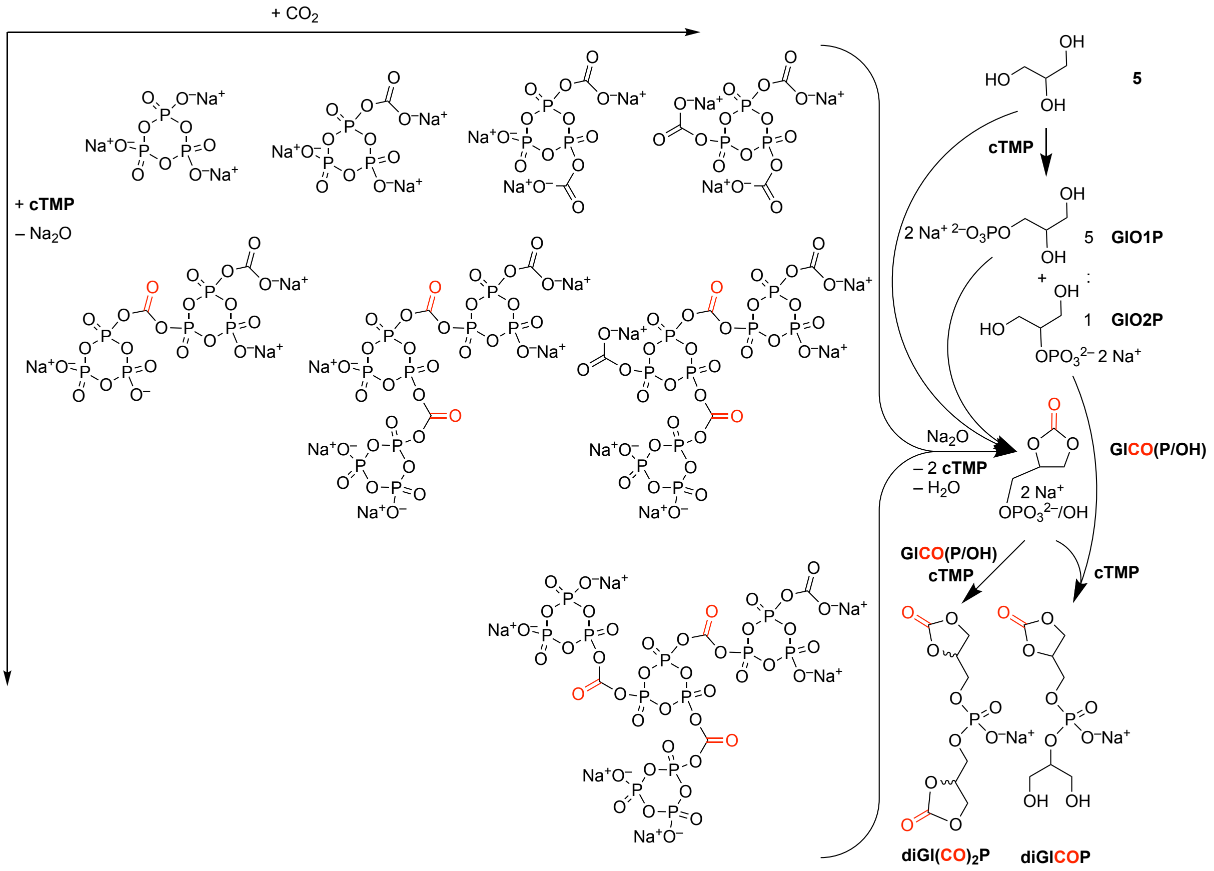

Supplementary Fig. 95. Hypothetic activation of CO_2 by **cTMP** reacting with neat glycerol (**5**) to give the observed main acyclic phosphorylated (**GlO1P**, **GlO2P**) and carbonylated products **GlCO(P/OH)**, **diGl(CO)$_2$P** and **diGlCOP**, the same as observed in the neat **P$_i$** + urea mixtures (Supplementary **Fig. 27**, Supplementary **Fig. 28**). Such carbonylated **cTMP** = (**cTMPCOcTMP**)$_n$ (n = 2 to 4 shown) should also be able to react with other 1,2-diols such as MPG (**6**, not shown here) to give carbonylated MPG (unlabelled Compound 11 shown in Supplementary **Fig. 33**).

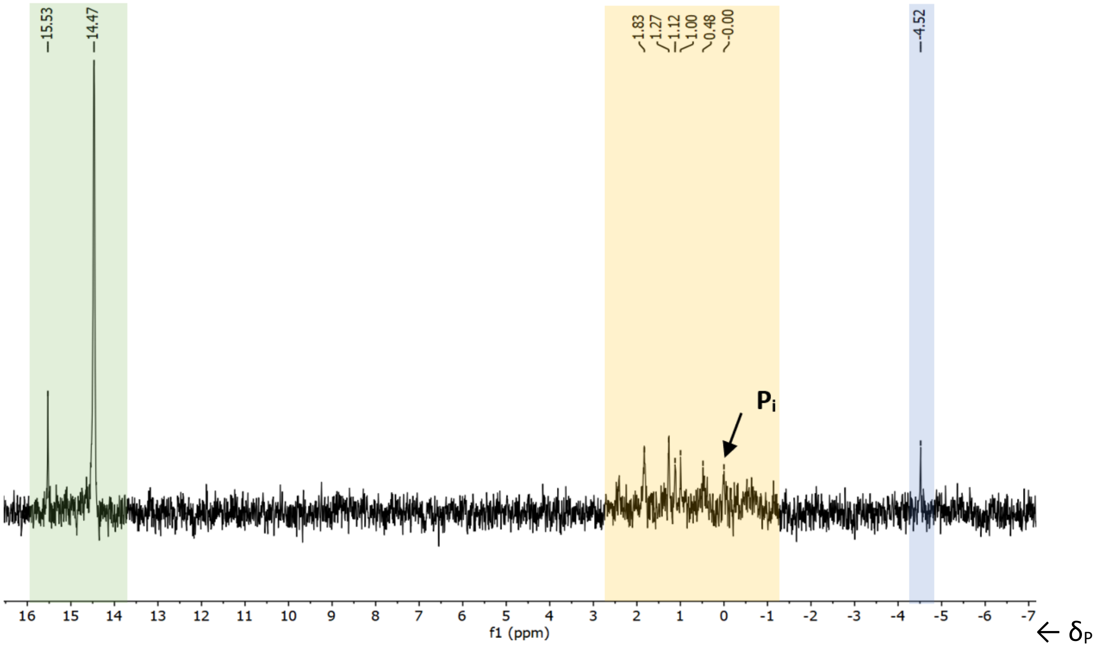

Supplementary Fig. 96. $^{31}P\{^1H\}$ NMR (202 MHz, DMSO-*d6*) of the extract of mixture 0.5 mmol scale **6**:**2a**:**cTMP** (1:1:1), reaction time 120 h at 115 °C. δ_P = 15.53 and 14.47 ppm (**5cMPGP**, **5cGlP**, green), 1.83-0.48 ppm (MPG-1-phosphates, MPG-2-phosphates, di-MPG phosphates, ochre), 0.00 ppm (**P$_i$**), –4.52 ppm (**6cMPGP**, blue).

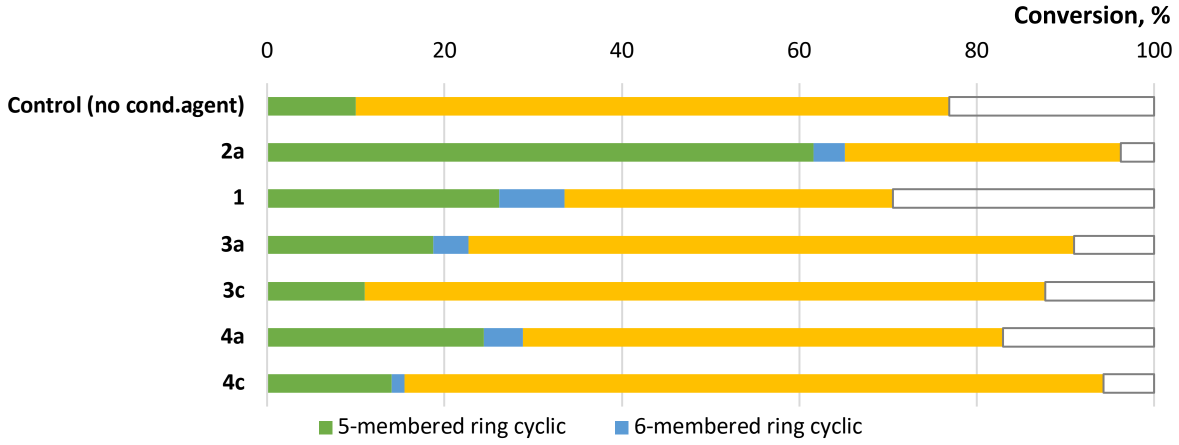

Supplementary Fig. 97. Summary of MPG (**6**) phosphorylation after 120 h of heating at 115 °C in a neat 0.5 mmol scale reaction mixture **6**:**cond.agent**:**cTMP** (1:1:1).

Supplementary Table 37. Data to Supplementary **Fig. 97**. Calculation of total conversion (conversion of initial amount of **cTMP** to phosphorylated organic products) was made by subtraction of inorganic compound quantities (P_i) from the sum of all integrated $^{31}P\{^1H\}$ NMR peak areas (in DMSO-*d*6). The weak SNR calls for caution.

Cond. agent	5cMPGP, %	6cMPGP, %	MPGP, %	P_i, %	Total conversion, %
-	9.99	-	66.89	23.12	76.88
**2a**	61.59	3.55	31.13	3.73	96.27
**1**	26.16	7.36	37.01	29.47	70.53
**3a**	18.75	3.96	68.28	9.01	90.99
**3c**	11.02	-	76.73	12.25	87.75
**4a**	24.41	4.46	54.07	17.06	82.94
**4c**	14.06	1.43	78.81	5.70	94.30

Equally good results were obtained in the phosphorylation of MPG (**6**) by **cTMP** at 115 °C (70.5-96.3%, Supplementary **Table 37** and Supplementary **Fig. 97**), as shown in the spectrum of the reaction with **6** in Supplementary **Fig. 96**. The presence of a condensing agent increased the fraction of 5- and 6-membered ring phosphates. The exact values in Supplementary **Table 37** should be taken with caution, since the ^{31}P $\{^1H\}$ and ^{31}P NMR spectra of mixtures containing MPG (**6**) often had a low SNR despite the large number of scans (cf. Section 4.2.1). In a similar series of experiments at 75 °C the highest total conversion was obtained in reaction with **2a** (88 % out of which 5-membered ring cyclic phosphates 6 % and acyclic organo-phosphates 82 %) and **1** (80 %). For all tested carboxamides we detected the presence of acyclic organo-phosphate signals, but the conversion of **cTMP** to products was not calculated.

Thus, **cTMP** appears to be an effective phosphorylation agent for glycerol and its mono-alkanoyl derivatives (as exemplified by MPG). However, it is important to note that there is still debate about whether or not **cTMP** was actually present on the early Earth in sufficient quantities to exert prebiotic phosphorylation reactions on organic molecules[33]. **cTMP** is formed from NaCl and 85 % orthophosphoric acid at 300-600 °C[35].

7.4.3. Experiments with sodium thiophosphate (SP$_i$)

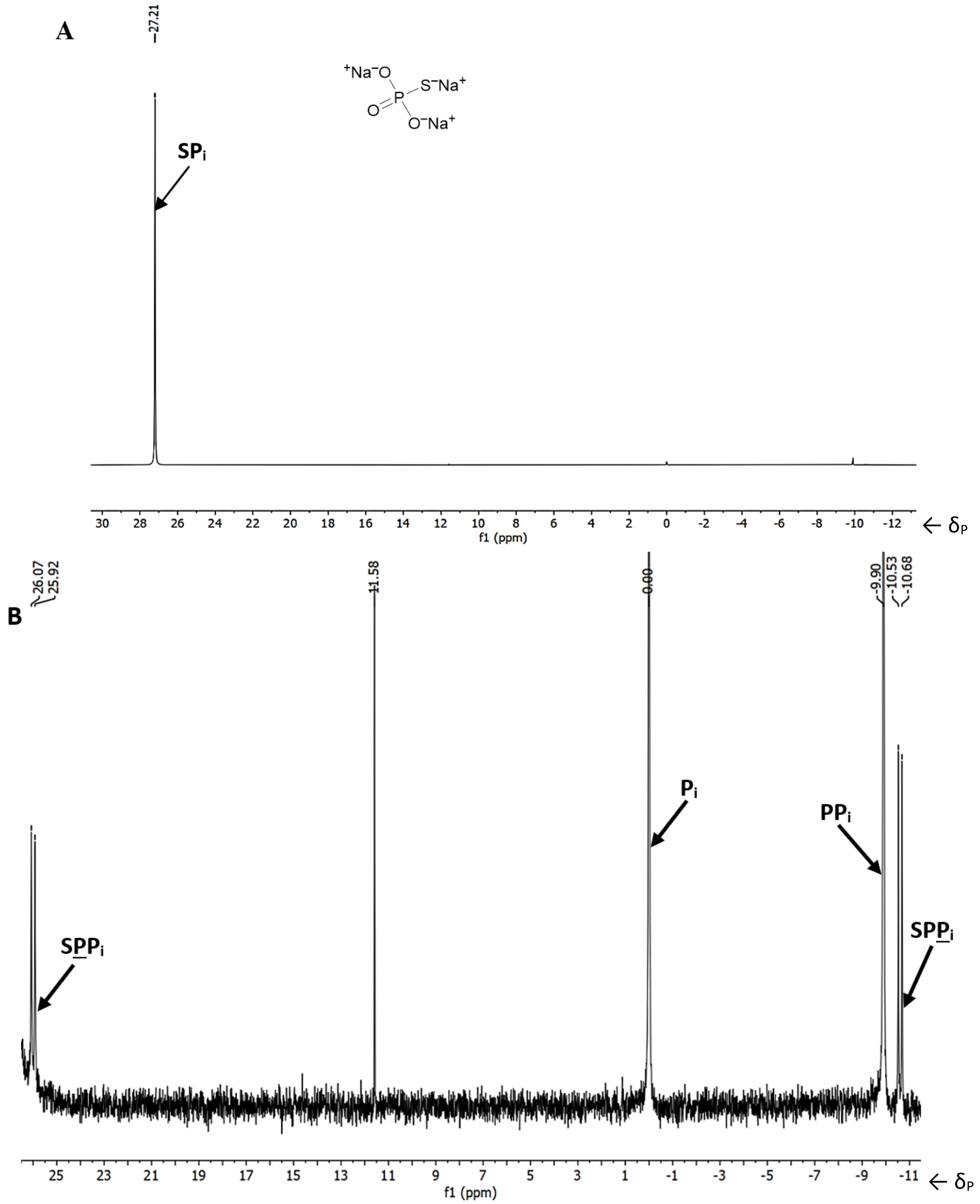

Supplementary Fig. 98. $^{31}P\{^1H\}$ NMR spectra (202 MHz) of commercial Na_3SPO_3 (**SP$_i$**) 0.5 mM in H_2O/D_2O = 9:1. δ_P (ppm) = 27.21 (*s*, **SP$_i$**), 26.00 (d, *J* = 30.7 Hz, **S$\underline{P}$P$_i$***), 11.58 (*s*), 0.00 (*s*, **P$_i$**), –9.90 (*s*, **PP$_i$**), –10.60 (d, *J* = 30.6 Hz, **SP$\underline{P_i}$**). The signal at 11.58 ppm is a singlet in the proton-coupled $^{31}P\{^1H\}$ NMR spectrum as well (not shown), its attribution unknown. Because the intensities of **SPP$_i$** signals do not change significantly during the reaction, their peak areas were not integrated and not considered in further calculations.

A – full spectrum; **B** – zoom into minor signals of **A**.

* **SPP$_i$** that originates from commercial Na_3SPO_3 (**SP$_i$**), it constitutes < 8% as indicated by the producer.

The ^{31}P {^{1}H} NMR spectrum of the **5**:**SP$_i$** reaction after 120 hours at 115 °C is shown in Supplementary **Fig. 99**. The obtained signals were identified by comparing $^{31}P\{^{1}H\}$ with ^{31}P NMR spectra as shown in Supplementary **Fig. 100**. Two main organic products are acyclic glyceryl-1- and -2-phosphates (21.70 %), along with a very small amount of glyceryl-1- and -2-thiophosphates (0.05 %).

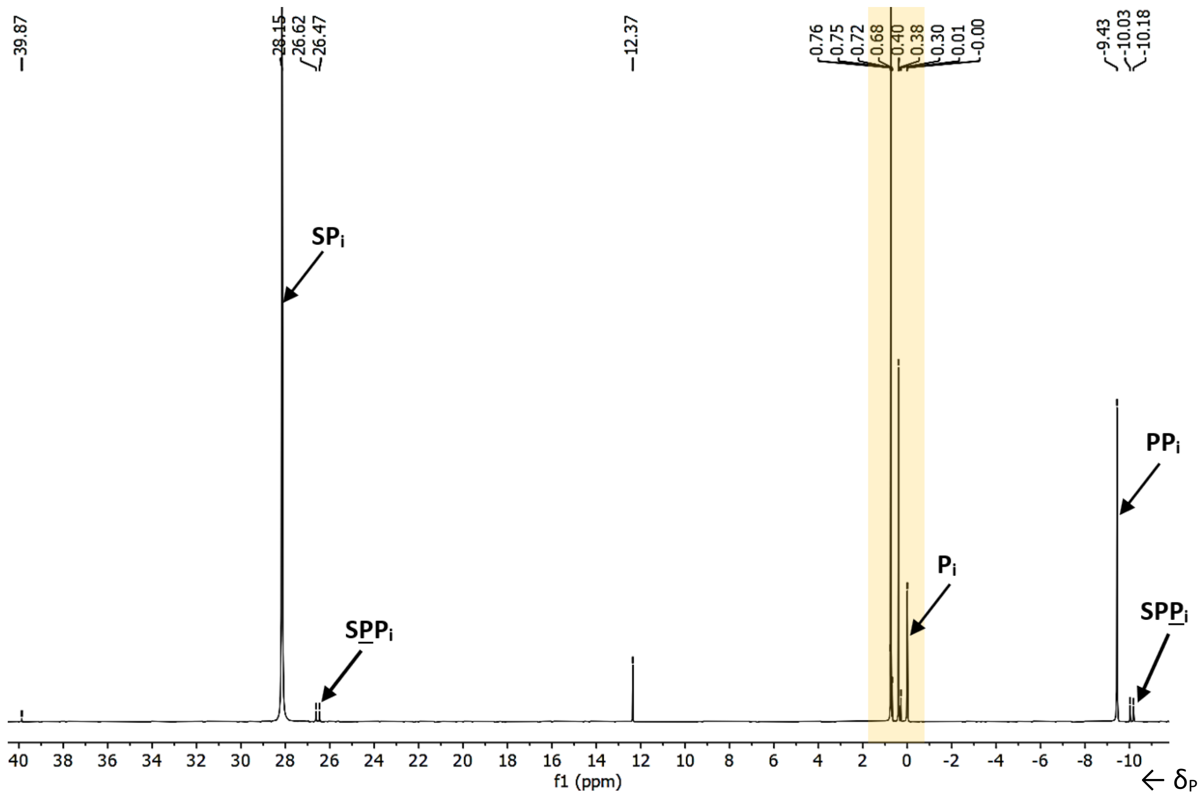

Supplementary Fig. 99. $^{31}P\{^{1}H\}$ NMR spectrum (202 MHz) of crude mixture **5**:**SP$_i$** (1:1), 0.5 mmol scale reaction after 120 h of heating at 115 °C, then dissolved in H_2O/D_2O = 9:1. δ_P (ppm) = 39.87, 39.64 (2 x *s*, glyceryl thiophosphates, **GlSP**), 28.15 (*s*, **SP$_i$**), 26.55 (*d*, J = 31.0 Hz, **S<u>P</u>P$_i$**), 12.37 (*s*), 0.75-0.63 (glyceryl-1-phosphates), 0.40-0.30 (glyceryl-2-phosphates), 0.00 (*s*, **P$_i$**), –9.43 (*s*, **PP$_i$**), –10.10 (*d*, J = 31.0 Hz, **SP<u>P</u>$_i$**). Ochre zone: acyclic glyceryl phosphates, see zooms in Supplementary **Fig. 100**.

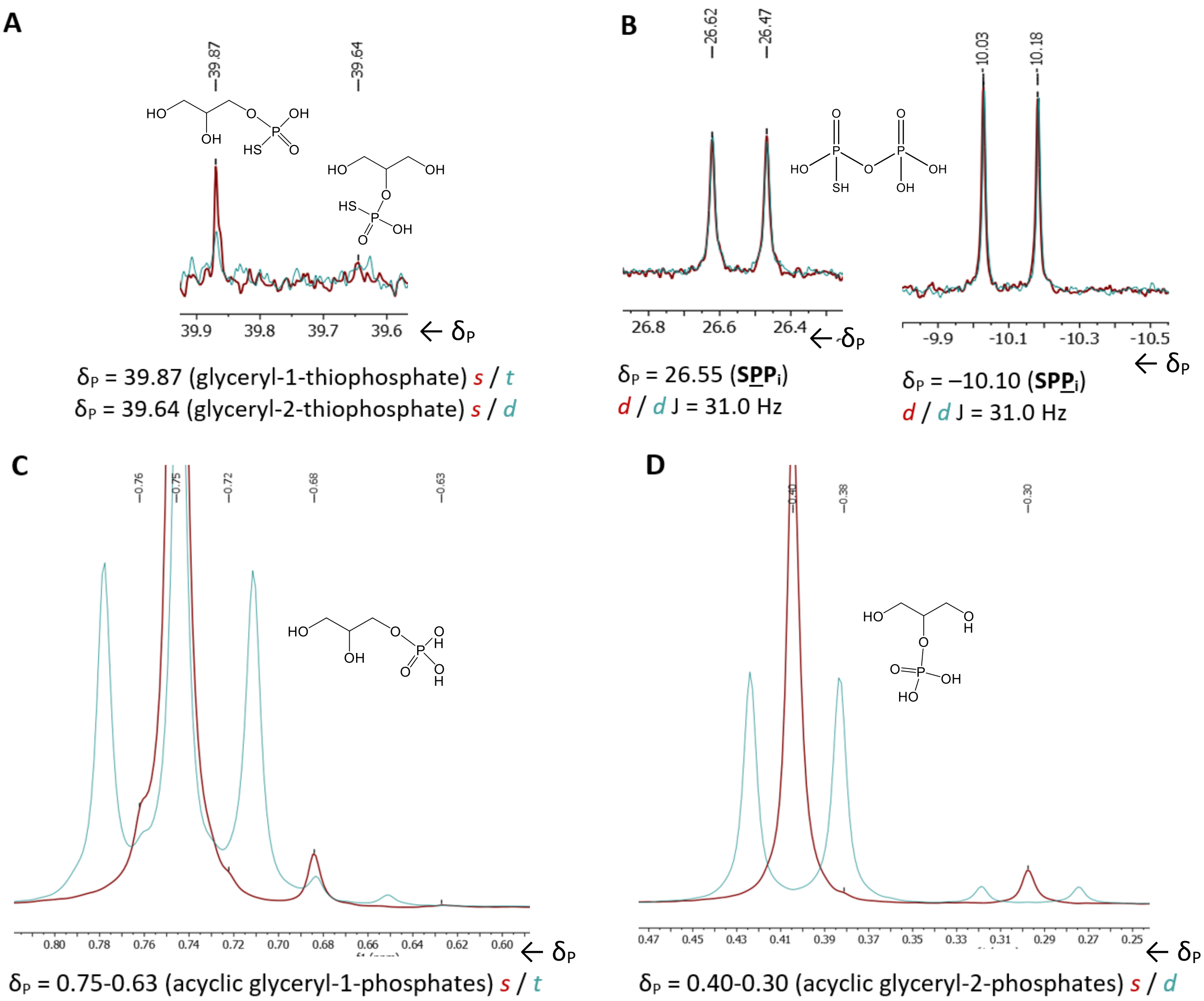

Supplementary Fig. 100. Zoomed zones of Supplementary **Fig. 99** (**brown**) superimposed on ^{31}P NMR spectrum (**turquoise**). **A** – glyceryl thiophosphates; **B** – inorganic thiopyrophosphate **SPP**$_i$; **C**, **D** – glyceryl phosphates (structure of only main compounds shown). Singlet at 12.37 ppm remains a singlet in ^{31}P NMR (not shown).

We performed several series of experiments involving reactions with condensing agents **1**, **2a** and reactions in mixtures of thio- and orthophosphate (1:1) at different protonation degrees. One of the most eventful $^{31}P\{^{1}H\}$ NMR spectra of a mixture **5**:**2a**:**SP$_i$**:**P$_i$** (1:1:0.5:0.5) on a 0.5 mmol reaction scale, after 120 h of heating at 115 °C, is shown in Supplementary **Fig. 101** with detailed signal assignment in Supplementary **Fig. 102**.

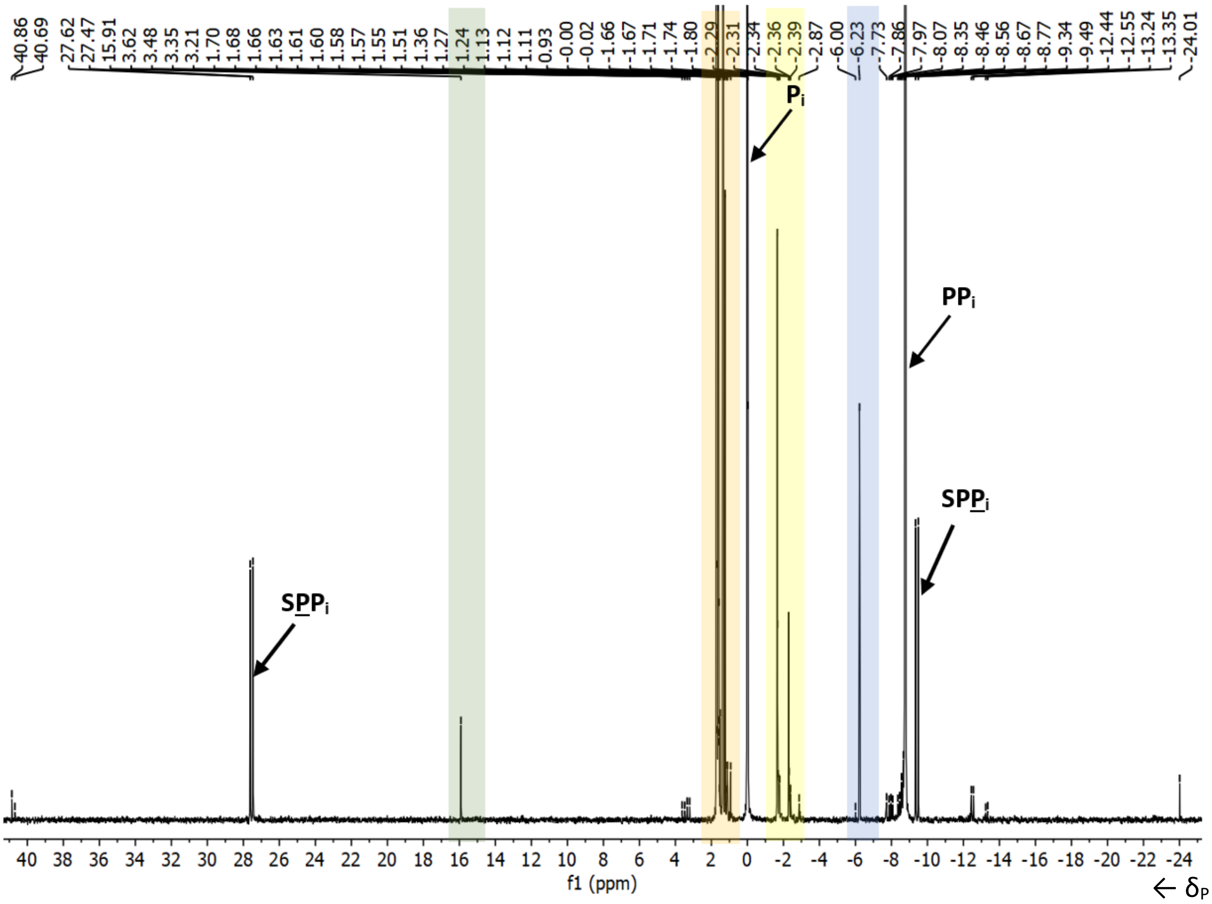

Supplementary Fig. 101. $^{31}P\{^{1}H\}$ NMR spectrum (202 MHz) of crude mixture **5**:**2a**:**SP$_i$**:**P$_i$** (1:1:0.5:0.5), 0.5 mmol scale reaction after 120 h of heating at 115 °C, then dissolved in H_2O/D_2O = 9:1. δ_P (ppm) = 40.86, 40.69 (2 x *s*, glyceryl thiophosphates, **GlSP**), 27.52 (*d*, J = 31.0 Hz, **SPP$_i$**), 15.91 (*s*, 5-membered ring cyclic phosphate **5cGlP,** green), 3.51, 3.28 (2 x *d*, J = 29.0 Hz, J = 28.0 Hz), 1.70-1.51 (acyclic glyceryl-1-phosphates, **GlO1P**, ochre), 1.36-0.92 (acyclic glyceryl-2-phosphates, **GlO2P**, ochre), 0.00 (*s*, **P$_i$**), 1.36-0.9, –1.66 to –2.87 (acyclic diglyceryl phosphates, **(Gl)$_2$P**, yellow), –6.0, –6.23 (2 x *s*, 6-membered ring cyclic phosphates **6cGlP**, blue), –7.80 (*d*, J = 27.4 Hz),–8.02 (*d*, J = 19.4 Hz), –8.41 (*d*, J = 21.9 Hz, glyceryl-2-diphosphate **GlPP**), –8.61 (*d*, J = 22.1 Hz glyceryl-1-diphosphate **GlPP**), –8.77 (*s*, **PP$_i$**), –9.41 (*d*, J = 31.0 Hz, **SPP$_i$**), –12.50 (*d*, J = 22.1 Hz, glyceryl-1-diphosphate **GlPP**), –13.29 (*d*, J = 22.1 Hz, glyceryl-2-diphosphate **GlPP**), –24.01 (*s*, **P$_{ni}$**).

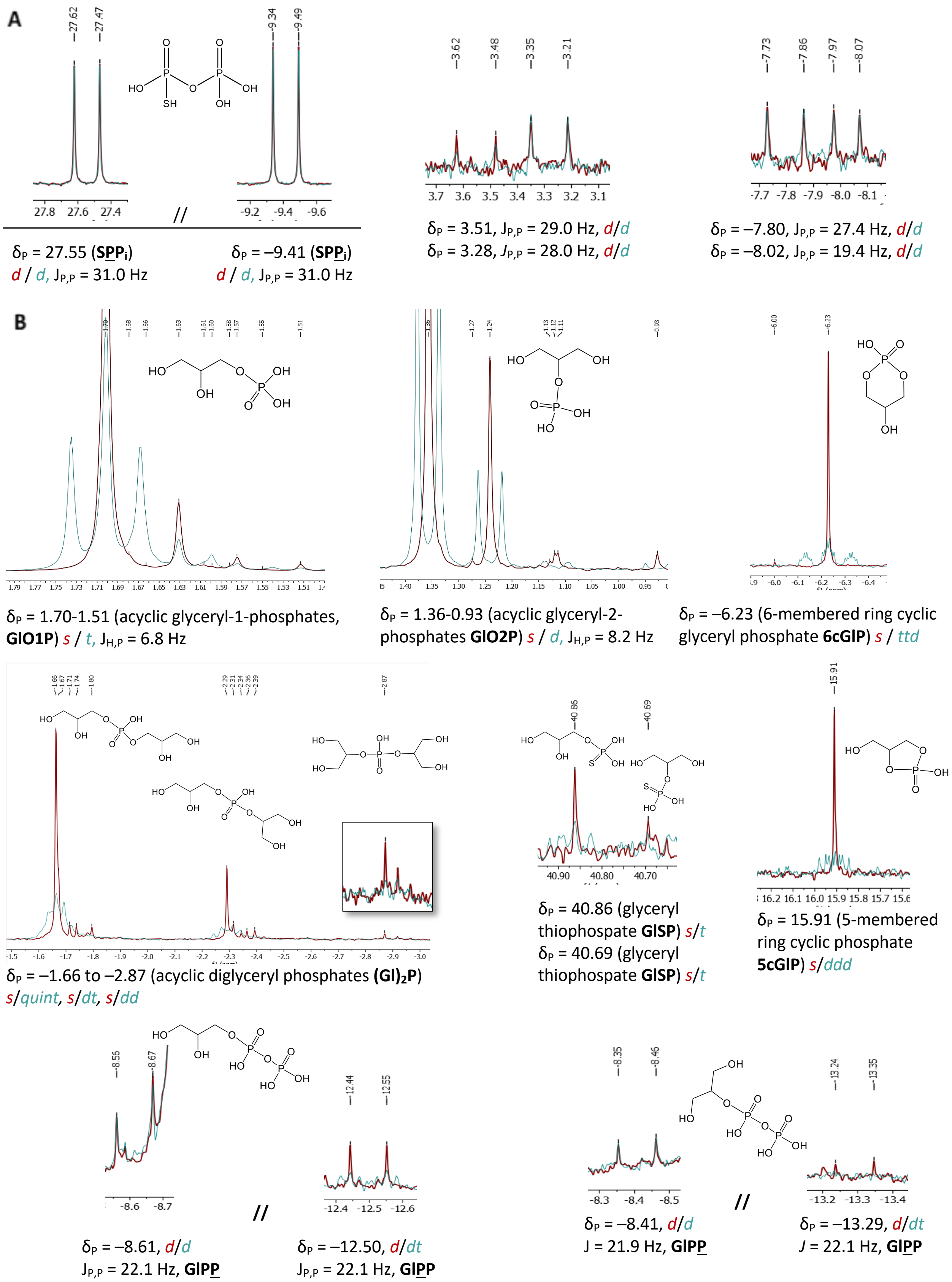

Supplementary Fig. 102. Zoomed zones of Supplementary **Fig. 101** (**brown**) superimposed on ^{31}P NMR spectrum (**turquoise**). **A** – inorganic compounds; **B** – glyceryl phosphates (compound structures of attributed signals shown). All x-axes: ^{31}P chemical shift δ_P [ppm].

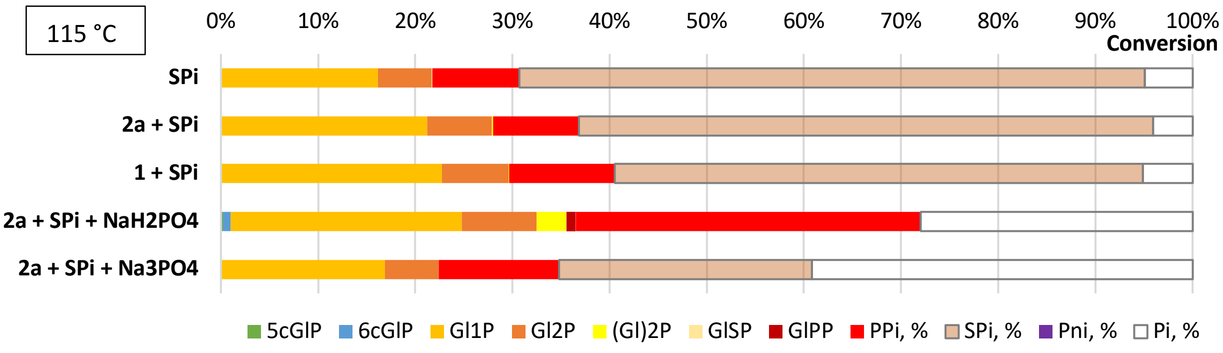

Supplementary Fig. 103. Summary of **SP$_i$**-driven phosphorylations of glycerol (**5**) after 120 h of heating at 115 °C, with and without urea (**2a**) or cyanamide (**1**), on a 0.5 mmol scale (no water added, 'dry' conditions). Molar equivalents are indicated in the diagram with respect to starting molecule **5** (1 eq). Organic products: **cGlP** (cyclic glyceryl phosphates), **Gl1P** (glyceryl-1-phosphates), **Gl2P** (glyceryl-2-phosphates), **(Gl)$_2$P** (diglyceryl phosphates), **GlSP** (glyceryl thiophosphates); **GlPP** (glyceryl diphosphates). Percent values from signal integration of quantitative $^{31}P\{^1H\}$ NMR spectra taken in H_2O/D_2O 9:1.

We also tested a milder reaction temperature (next page).

Supplementary Table 38. Data to Supplementary **Fig. 103**. Total conversion of initial amount of **SP$_i$** and **P$_i$** to phosphorylated organic products was calculated by subtracting inorganic compound integrals (**P$_i$**, **SP$_i$**, **PP$_i$**, **P$_{ni}$**) from the sum of all integrated $^{31}P\{^1H\}$ NMR peak areas.

Condensing agent*	Phosphate source		5cGlP %	6cGlP %	Acyclic organo-phosphates %					PP$_i$ %	SP$_i$ %	P$_i$ %	P$_{ni}$ %	Total conv. %
					GlO1P	GlO2P	(Gl)$_2$P	GlSP	GlPP					
-	**SP$_i$**	-	-	-	16.19	5.51	-	0.05	-	8.96	64.38	4.91	-	21.75
**2a**	**SP$_i$**	-	-	0.02	21.21	6.67	0.09	0.02	-	8.86	59.04	4.07	0.02	28.01
**1**	**SP$_i$**	-	0.03	0.08	22.63	6.83	0.09	-	-	10.88	54.33	5.13	-	29.66
**2a**	**SP$_i$** §	NaH_2PO_4	0.21	0.82	23.65	7.67	3.00	0.07	0.96	35.25	-	27.89	0.07	36.38
**2a**	**SP$_i$** §	Na_3PO_4	-	-	16.90	5.52	-	0.02	-	12.32	26.05	39.15	-	22.44

* Equimolar condensing agent with respect to phosphate source and starting compound **5** (1:1:1).

§ Equimolar ratio between two phosphate sources with respect to 1 eq of starting compound **5** and cond. agent (1:0.5:0.5).

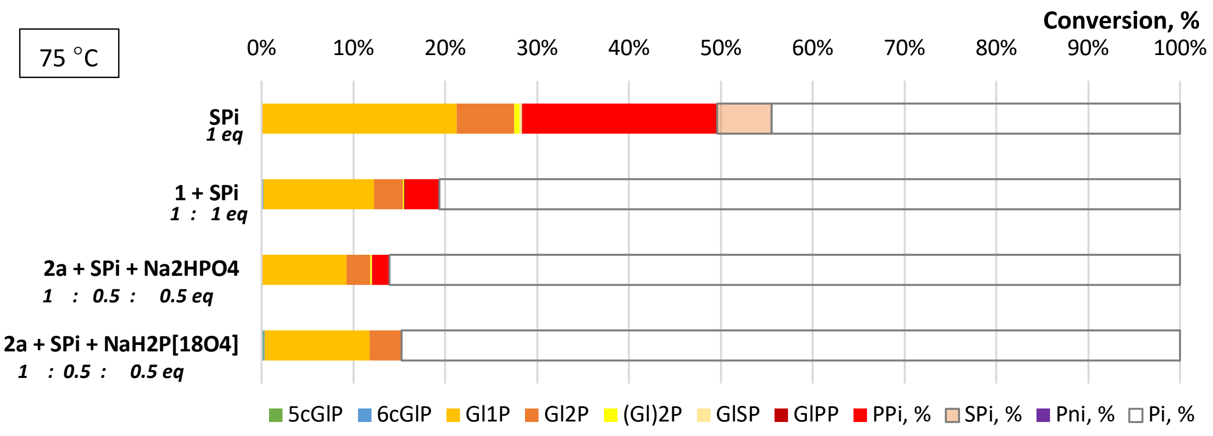

Supplementary Fig. 104. Summary of $\mathbf{SP_i}$-driven phosphorylations of glycerol (**5**) after 120 h of heating at 75 °C, with and without urea (**2a**) or cyanamide (**1**), on a 0.5 mmol scale in 1 ml D_2O ('wet and evaporating' conditions). Molar equivalents are indicated in the diagram with respect to starting molecule **5** (1 eq). Organic products: **cGlP** (cyclic glyceryl phosphates), **Gl1P** (glyceryl-1-phosphates), **Gl2P** (glyceryl-2-phosphates), **(Gl)₂P** (diglyceryl phosphates), **GlSP** (glyceryl thiophosphates); **GlPP** (glyceryl diphosphates). Percent values from signal integration of quantitative $^{31}P\{^1H\}$ NMR spectra taken in H_2O/D_2O 9:1.

Compared to the experiment without any condensing agent, the involvement of urea increased phosphorylation of **5** up to 28-29 %, which is markedly lower compared to the $\mathbf{P_i}$ phosphorylation of 91-95 % (Supplementary **Fig. 83**). The presence of orthophosphate does not help with producing organic phosphates but favours consuming $\mathbf{SP_i}$. In all experiments described here, the presence of cyclic glyceryl phosphate was detected only in the reaction with $\mathbf{SP_i}$:$\mathbf{P_i}$ (1:1). We notice that in the sequence **5**:$\mathbf{SP_i}$ > **5**:**2a**:$\mathbf{SP_i}$ > **5**:**2a**:$\mathbf{SP_i}$:$\mathbf{P_i}$ phosphorylation increased from 21.75 % through 28.01 % to 36.38 %. As expected from the mechanism of urea-assisted phosphorylation reactions necessitating the presence of sufficient protons (provided by NaH_2PO_4, for example) in order to eliminate NH_3, the same is true for the formation of H_2S as a good leaving group. In a similar experiment, where tribasic phosphate was used, no such yield increase was observed, but it is remarkable that we found > 20 % phosphorylation under such highly alkaline conditions. Lower temperatures did not help phosphorylation (< 29 % at 75 °C).

Supplementary Table 39. Data to Supplementary **Fig. 104**. Total conversion of initial amount of $\mathbf{SP_i}$ and $\mathbf{P_i}$ to phosphorylated organic products was calculated by subtracting inorganic compound integrals ($\mathbf{P_i}$, $\mathbf{SP_i}$, $\mathbf{PP_i}$, $\mathbf{P_{ni}}$) from the sum of all integrated $^{31}P\{^1H\}$ NMR peaks. Molar ratios as in Supplementary **Fig. 104**.

Condensing agent	Phosphorus source		5cGlP, %	6cGlP, %	Acyclic glyceryl phosphates, % GlO1P	GlO2P	GlPGl	GlSP	GlPP	PP_i %	SP_i %	P_{ni} %	P_i %	Total conversion, %
-	SP_i	-	0.03	0.01	21.19	6.26	0.58	0.25	0.07	21.2	5.91	0.02	44.48	28.39
1	SP_i	-	-	0.22	11.96	3.09	0.11	0.03	0.15	3.64	-	-	79.98	15.56
2a	SP_i	Na_2HPO_4	-	0.05	9.22	2.58	0.15	0.02	0.03	1.90	-	-	86.04	12.05
2a	SP_i*	$NaH_2[^{18}O_4]$	0.22	0.13	11.44	3.48	-	-	-	-	-	-	84.72	15.27

* Equimolar ratio between two phosphate sources with respect to 1 eq of starting compound **5** and cond. agent (1:0.5:0.5).

7.4.3.1. Competition between SP_i and $[^{18}O_4]P_i$ in urea-assisted phosphorylations of glycerol and MPG

The increase in yields in the reaction with mixed inorganic phosphates (**SP_i:P_i**) raised our interest in exploring the competition between them in product formation. To pursue this study, we used labelled **$[^{18}O]P_i$**; thus, we were able to track phosphorylated molecules produced by orthophosphates against unlabelled thiophosphate by using HRMS.

In Supplementary **Fig. 105**, the total-ion chromatogram in the negative-ion mode of the crude mixture **5**:**2a**:**SP_i**:**$[^{18}O]P_i$** (1:1:0.5:0.5) at a 0.5 mmol reaction scale, heated neat for 120 h at 115 °C is shown. The main signals belong to glyceryl monophosphate and its isotopologs. Along with it, we identified glyceryl carbamate (phosphorylated and not phosphorylated), cyclic monoglyceryl phosphate and diglyceryl phosphate (Supplementary **Table 40**). The ratio of unlabelled-to-labelled (i.e., in partly labelled) glyceryl phosphates was approximately 60:40. This demonstrated that **SP_i** is more efficient in the phosphorylation reaction compared to **P_i**. Protonated **SP_i**, because of the H_2S leaving group, can phosphorylate up to 69% of detected products.

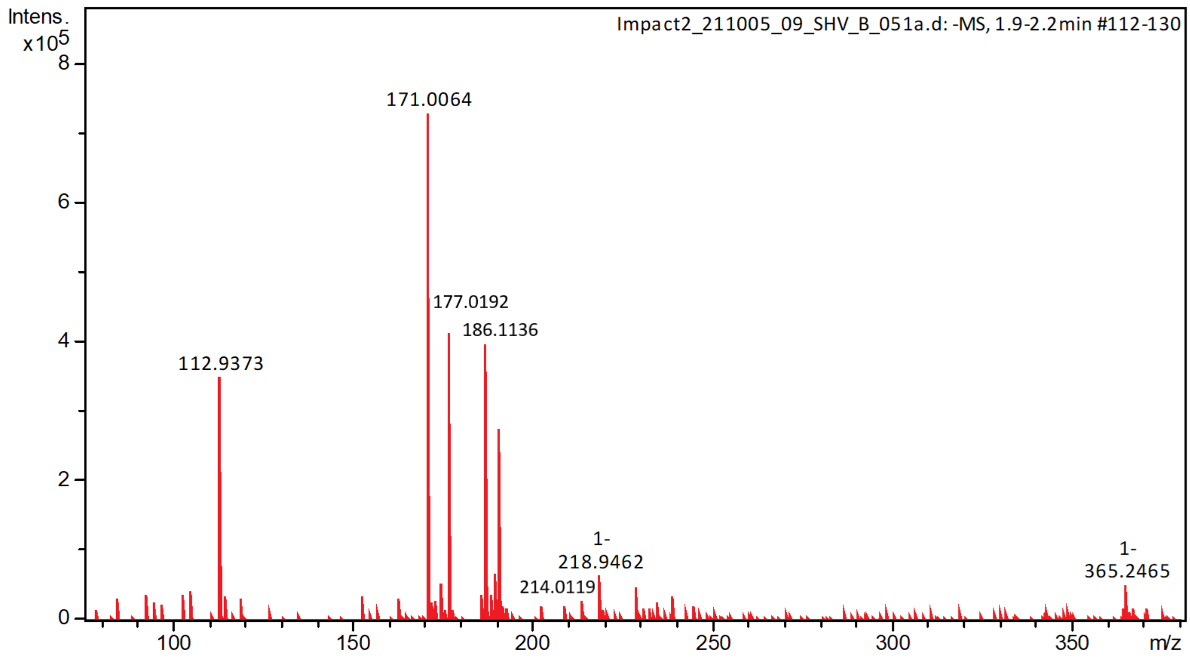

Supplementary Fig. 105. Negative ion mode HRMS of the methanolic extract of crude mixture **5**:**2a**:**SP_i**:**$[^{18}O]P_i$** (1:1:0.5:0.5) ; 0.5 mmol scale ; reaction time 120 h at 115 °C.

Supplementary Table 40. Interpretation of HRMS of Supplementary **Fig. 105** with suggested formulas, structures and measured relative isotopolog ratios per compound. The ion for all listed masses is $[M–H]^{-}$, if not specified otherwise.

Peak number	*m/z* of the main signal	Suggested chemical formula	Suggested chemical structure	Oxygen isotopolog ratio
**1**	134.0463	$C_4H_9NO_4$	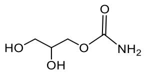	-
	152.9959	$C_3H_7O_5P$	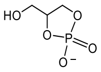	68.9
	155.0002	$C_3H_7O_4P[^{18}O]$	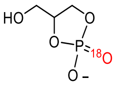	3.3
**2**	157.0045	$C_3H_7O_3P[^{18}O_2]$	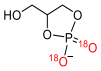	23.1
	159.0085	$C_3H_7O_2P[^{18}O_3]$	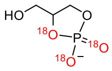 *	4.7
**3**	170.9971	$C_3H_8O_6S$	-	-
	171.0064	$C_3H_9O_6P$	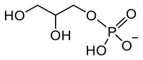	61.0
**4**	175.0150	$C_3H_9O_4P[^{18}O_2]$	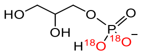	4.4
	177.0192	$C_3H_9O_3P[^{18}O_3]$	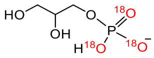	34.6
**5**	186.1136	$C_9H_{17}NO_3$	-	-
**6**	186.9741	$C_3H_8O_5S_2$		-
	214.0119	$C_4H_{10}NO_7P$	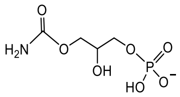	63.4
**7**	218.0205	$C_4H_{10}NO_5P[^{18}O_2]$	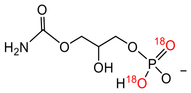	4.3
	220.0250	$C_4H_{10}NO_4P[^{18}O_3]$	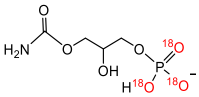	32.3
	245.0433	$C_6H_{15}O_8P$	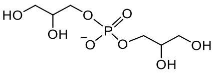	69.3
**8**	249.0510	$C_6H_{15}O_6P[^{18}O_2]$	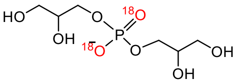	17.3
	251.0572	$C_6H_{15}O_5P[^{18}O_3]$	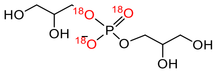 *	13.5
**9**	250.9633	$C_3H_9O_9PS$	-	65.4
	256.9761	$C_3H_9O_6PS[^{18}O_3]$	-	34.6
**10**	365.2465 $[M+Cl]^{-}$	$C_{19}H_{38}O_4$	-	-

* The position of one ^{18}O isotope is uncertain resulting probably from reversible addition-elimination at 115°C.

The phosphorylation of MPG (**6**) was more challenging than the previously described glycerol (**5**) due to the low solubility of the crude mixture in water and DMSO-*d*6. There are only two experiments where the product yields were reliably quantified: **6**:**2a**:**SP**$_i$:**P**$_i$ (1:1:0.5:0.5) on a 0.5 mmol reaction scale, after heating at 75 °C for 120 h, one in a dry mixture and another with a beforehand addition of 1 ml water that eventually evaporated during the experiment (Supplementary **Fig. 106**). The spectra and results suggest relatively high amounts of acyclic and cyclic products 61.5 and 93.9 % in 'dry' and 'wet-to-dryness' experiments, respectively (Supplementary **Fig. 107**). It should be mentioned that these results should be compared with caution to the glycerol phosphorylation data, considering the different solvent. Most likely, **SP**$_i$ as well as **SPP**$_i$ and **PP**$_i$ are less soluble in DMSO than in water, which can have an influence on the quantification by ^{31}P NMR spectroscopy.

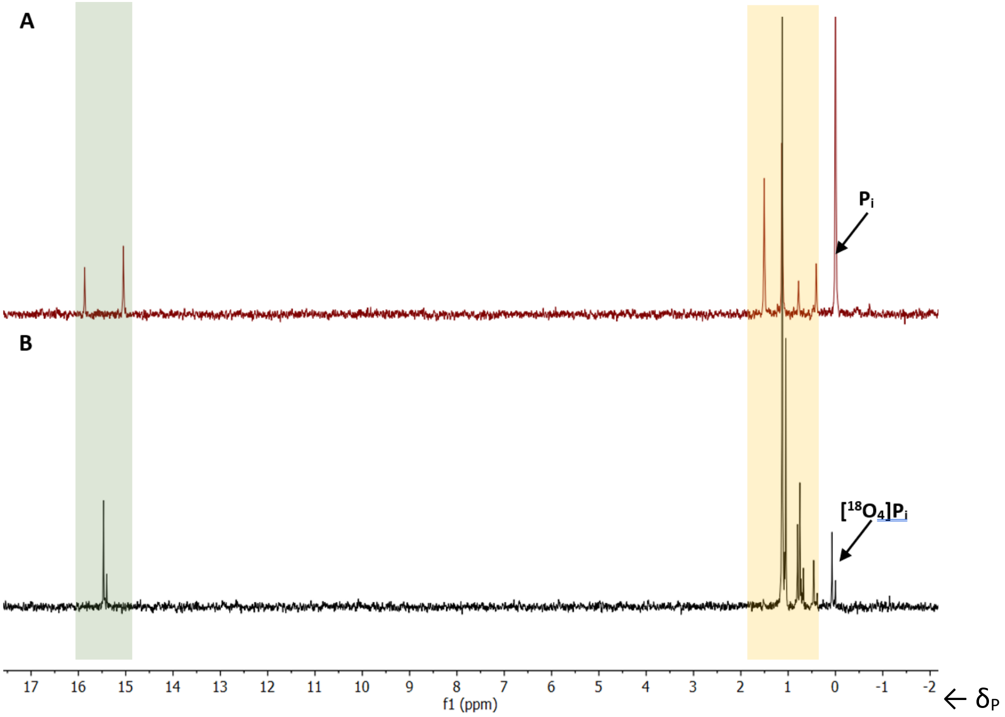

Supplementary Fig. 106. ^{31}P{^{1}H} NMR spectra (202 MHz) of crude mixture **6**:**2a**:**SP**$_i$:**P**$_i$ (1:1:0.5:0.5); 0.5 mmol scale reaction after heating for 120 h at 75 °C, then dissolved in DMSO-*d*6. δ_P (ppm) = 16-15 (green zone: 2 x *s*, 5-membered ring cyclic MPG phosphates, **5-cMPGP**), 1.7-0.3 (ochre zone: acyclic MPG phosphates = **MPG1P**, **MPG2P**, **(MPG)$_2$P**). **P**$_i$ appears in spectrum **B** as two singlets at δ_P = 0.00 and –0.09 ppm representing two isotopes of **P**$_i$, **[^{16}O$_4$]P**$_i$ from added and hydrolysed unlabelled **SP**$_i$ and **[^{18}O$_4$]P**$_i$ from added NaH$_2$P[^{18}O$_4$] (the part that did not exchange its oxygen atoms with added D$_2$^{16}O) ; isotopic shift $\Delta\delta_P$([^{16}O$_4$]P$_i$ – [^{18}O$_4$]P$_i$) = 17.3 Hz, i.e., 4.3 Hz per ^{18}O atom. **A** – without D$_2$O as a liquidiser ('dry' conditions); **B** – in 1 ml D$_2$O ('wet and evaporating' conditions).

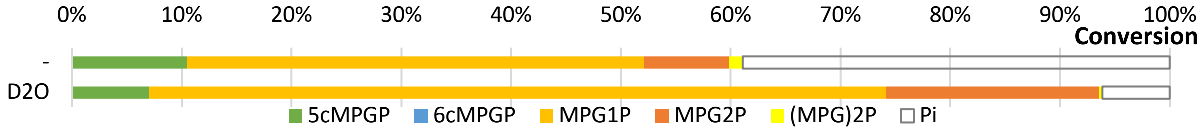

Supplementary Fig. 107. Summary of **SP**$_i$/**P**$_i$- and urea-driven phosphorylations of MPG (**6**) after 120 h of heating at 75 °C of **6**:**2a**:**P**$_i$:**SP**$_i$ (1:1:0.5:0.5) on a 0.5 mmol scale, without and with 1 ml D$_2$O (water evaporated eventually). Organic products: **5cGlP** (5-membered ring cyclic MPG phosphates), **MPG1P** (MPG-*O*1-phosphates), **MPG2P** (MPG-2-phosphates), **(MPG)$_2$P** (di-MPG phosphates). Percent values from signal integration of quantitative ^{31}P{^{1}H} NMR spectra taken in DMSO-*d*6. NMR spectra for the dry reaction mixture were measured at 40 °C to increase the solubility of the sample. Calculation of total conversion of the initial amount of **SP**$_i$ and **P**$_i$ to phosphorylated organic products was made by subtraction of inorganic compound quantities (**P**$_i$, **SP**$_i$, **PP**$_i$) from the sum of all integrated ^{31}P{^{1}H} NMR peak areas.

Supplementary Table 41. Data to Supplementary **Fig. 107**. Total conversion of initial amount of $\mathbf{SP_i}$ and $\mathbf{P_i}$ to phosphorylated organic products was calculated by subtracting inorganic compound integrals ($\mathbf{P_i}$, $\mathbf{SP_i}$, $\mathbf{PP_i}$, $\mathbf{P_{ni}}$) from the sum of all integrated $^{31}P\{^1H\}$ NMR peak areas.

Solvent	5cMPGP, %	6cMPGP, %	Acyclic organo-phosphates, %			P_i, %	SP_i, %	PP_i, %	Total conversion, %
			MPGl1P	MPGl2P	$(MPG)_2P$				
**-**	10.57	-	41.91	7.8	1.21	39.17	-	-	61.49
**D_2O**	7.07	-	67.08	25.56	0.29	6.14	-	-	93.86

All other experiments with **6** and $\mathbf{SP_i}$ were complicated by the low solubility of the crude reaction mixtures in DMSO-*d6* despite the NMR analysis being conducted at 40 °C in order to increase the solubility of the crude mixtures. We identified 5-membered ring cyclic phosphates and acyclic MPG phosphates as the main products of reactions in various ratios, but without quantification owing to too low signal-to-noise ratios (SNR). In the reactions of **6** and $\mathbf{SP_i}$ with and without condensing agent (**2a** or **1**) after heating for 120 h at 75 °C, the ratio of 5-membered ring cyclic MPG phosphates over acyclic MPG phosphates was 1:3. In experiments with a mixture of phosphate sources we measured this ratio as 1:4.5 and 1:1 in the mixtures **6**:**2a**:$\mathbf{SP_i}$:$\mathbf{Na_3PO_4}$ and, respectively, **6**:**2a**:$\mathbf{SP_i}$:$\mathbf{NaH_2PO_4}$ (1:1:0.5:0.5 moles).

Similar to the above described, the phosphorylation reaction of **6**:**2a**:$\mathbf{SP_i}$:**[^{18}O]**$\mathbf{P_i}$ (1:1:0.5:0.5) on a 0.5 mmol scale; reaction time 120 h at 115 °C, was studied by HRMS (Supplementary **Fig. 108**). The results of the experiment confirm that $\mathbf{SP_i}$ outperforms the competition with $\mathbf{P_i}$ by about 67-74 % and some products, such as **5cMPGP**, **5cGlP**, $\mathbf{Gl_2P}$ and glyceryl carbamate, were found only in unlabelled form (Supplementary **Table 42**). These results are consistent with the previous experiments and confirm that the presence of protons favours phosphorylation by thiophosphate and contributes up to 60-70 % to the total conversion to organic phosphates.

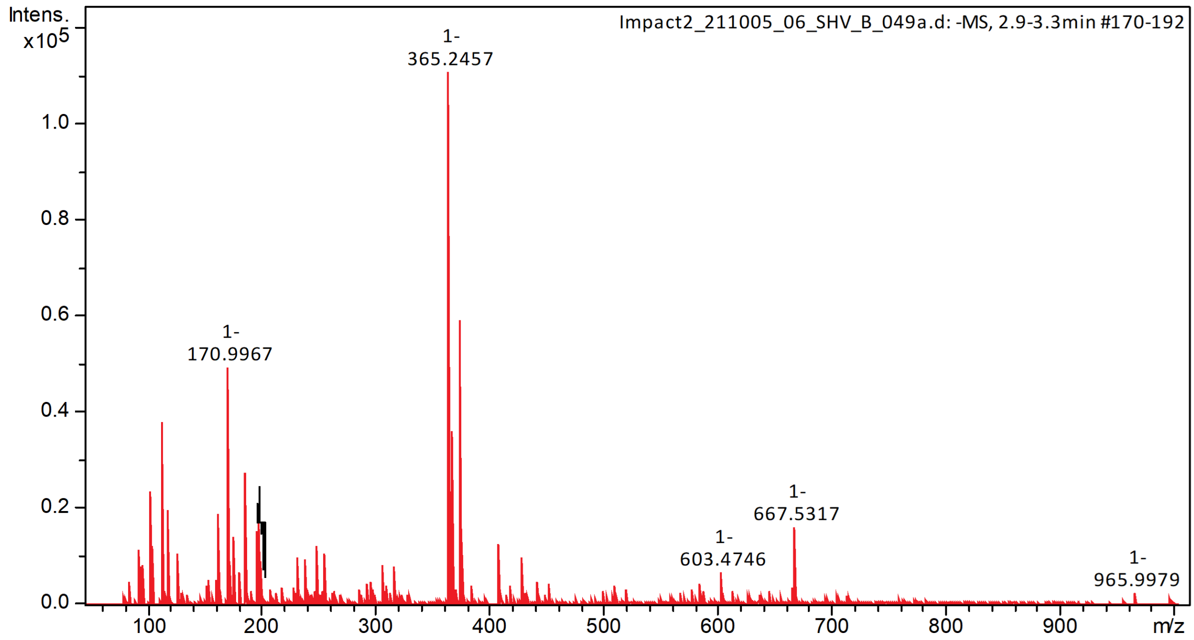

Supplementary Fig. 108. Negative ion mode HRMS of the methanolic extract of crude mixture **6**:**2a**:$\mathbf{SP_i}$:$\mathbf{P_i}$ (1:1:0.5:0.5) ; 0.5 mmol scale ; reaction time 120 h at 115 °C.

Supplementary Table 42. Interpretation of HRMS of Supplementary **Fig. 108** with suggested formulas, structures and measured relative isotopolog ratios per compound. The ion for all listed masses is $[M–H]^−$, if not specified otherwise. * The position of one ^{18}O isotope is uncertain (resulting probably from reversible addition-elimination at 115 °C).

Peak number	*m/z* of the main signal	Suggested chemical formula	Suggested chemical structure	Isotopolog ratio
**1**	152.9956	$C_3H_7O_5P$	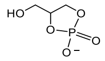	67.9
	157.0038	$C_3H_7O_4P[^{18}O]$	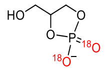	27.8
	159.0079	$C_3H_7O_3P[^{18}O_2]$	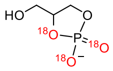 *	4.3
**2**	170.9967	$C_3H_8O_6S$	-	-
**3**	171.0058	$C_3H_9O_6P$	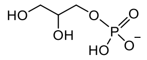	71.3
	173.0099	$C_3H_9O_5P[^{18}O]$	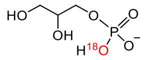	1.6
	175.0144	$C_3H_9O_4P[^{18}O_2]$	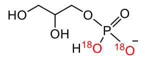	3.1
	177.0187	$C_3H_9O_3P[^{18}O_3]$	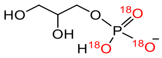	24.0
**4**	186.1132	$C_9H_{17}NO_3$	-	-
**5**	186.9735	$C_3H_8O_5S_2$	-	-
**6**	214.0113	$C_4H_{10}NO_7P$	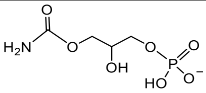	74.4
	220.0244	$C_4H_{10}NO_4P[^{18}O_3]$	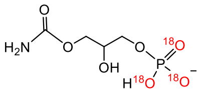	25.6
**7**	232.9616	$C_3H_8O_8P_2$	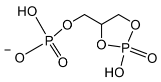	-
**8**	245.0424	$C_6H_{15}O_8P$	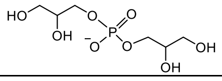	
**9**	250.9633	$C_3H_9O_9PS$	-	-
**10**	375.2743 $[M+HCOOH–H]^−$	$C_{19}H_{38}O_4$	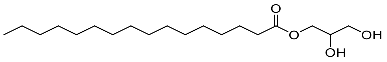	-
**11**	372.2749	$C_{20}H_{39}NO_5$	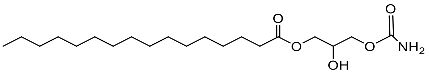	-
**12**	391.2252 $[M+Cl]^−$	$C_{20}H_{36}O_5$	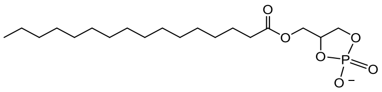	-
**13.1**	409.2287	$C_{19}H_{38}O_7S$	-	-
**13.2**	411.2421	$C_{19}H_{39}O_6P[^{18}O]$	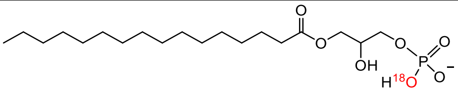	24.9
	413.2427	$C_{19}H_{39}O_5P[^{18}O_2]$	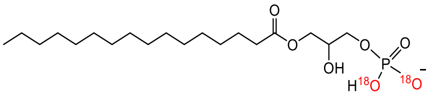	11.0
	415.2480	$C_{19}H_{39}O_4P[^{18}O_3]$	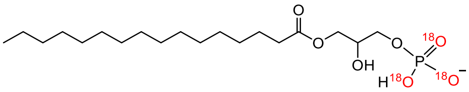	64.1
**14**	603.4746 $[M+Cl]^−$	$C_{35}H_{68}O_5$	-	-

7.4.4. Involving natural minerals and their analogues in phosphorylation of glycerol and MPG

Three commercially available and prebiotically plausible minerals were chosen to be tested: natural vivianite, that is, a blue rock grinded to powdered $Fe_3(PO_4)_2{\cdot}8H_2O$, synthetic struvite ($NH_4MgPO_4{\cdot}6H_2O$) and calcium pyrophosphate ($Ca_2P_2O_7$) as an analogue of natural canaphite ($CaNa_2P_2O_7{\cdot}4H_2O$). The results are summarised in Supplementary **Fig. 111** and Supplementary **Table 43**. We did not succeed in phosphorylating MPG (**6**) with all three tested phosphate sources; however, glycerol phosphorylation seems to be prospective.

The work with vivianite was challenging due to the presence of ferrous ions in the extracts. The initial treatment included using a saturated disodium ethylenediaminetetraacetate $Na_2[H_2EDTA]$ solution[36] to exchange Fe^{2+} ions with Na^+. However, we did not succeed in obtaining good-quality $^{31}P\{^1H\}$ NMR spectra of such solutions (and no ferrous or ferric EDTA precipitate after several weeks). Another, successful approach was to use a saturated sodium sulfide solution (Na_2S) at high pH > 10. Following this procedure, we obtained clear $^{31}P\{^1H\}$ NMR spectra that were used for the quantification. However, the phosphorylation did not appear to be very efficient: 38 % maximum yield of acyclic glyceryl phosphates in the reaction **5**:**2a**:vivianite (1:1:1) (Supplementary **Fig. 110 B**).

Both struvite and canaphite analogues demonstrated equivalent phosphoric ester production in the presence and absence of a condensing agent. A possible explanation for the efficacy of struvite is the presence of acidic ammonium cations that favour the phosphorylation reaction. Especially interesting seems to be the case of pyrophosphate-containing $Ca_2P_2O_7$. No $^{31}P\{^1H\}$ NMR resonance at low field strength, thus, no sign for the presence of **PP**$_i$ was detected at the end of phosphorylation, which indicated that the initial material was completely consumed into phosphorylated products and **P**$_i$.

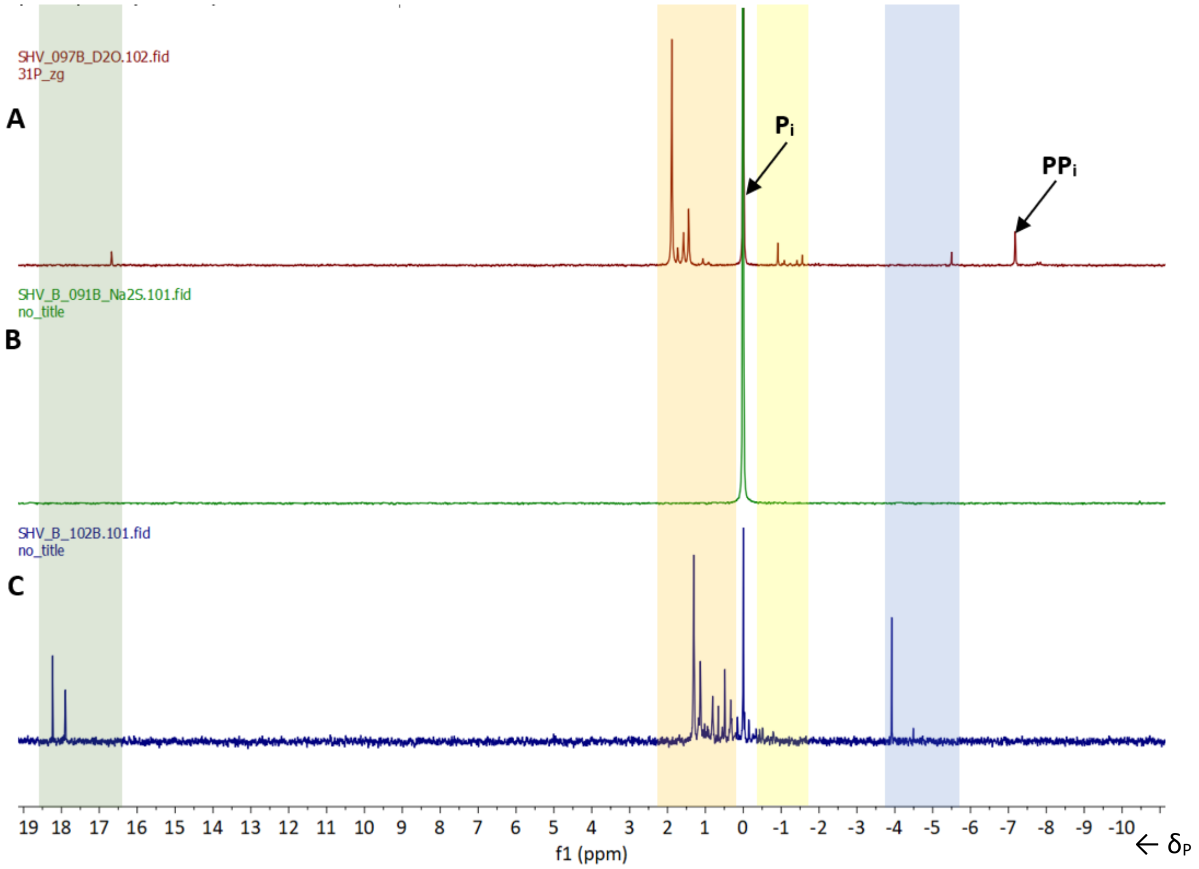

Supplementary Fig. 109. $^{31}P\{^1H\}$ NMR spectra (202.5 MHz) of crude mixture **5**:**mineral/analogue**:**2a** (1:1:1), 0.5 mmol scale reaction after 120 h of heating at 115 °C, then dissolved in D_2O. δ_P (ppm) = 19-16 (green zone: 5-membered ring cyclic phosphates, **5-GP**), 2.0-0.3 (ochre zone: acyclic phosphates **GO1P**, **GO2P**), 0.00 (*s*, **P**$_i$), –1.66 to –2.87 (acyclic diglyceryl phosphates, **(Gl)$_2$P**, yellow zone), –4 to –6 (6-membered ring cyclic phosphates **6cGlP**, blue zone), –7 (*s*, **PP**$_i$). **A** – struvite (NH_4Mg**P**$_i$); **B** – vivianite (Fe_3(**P**$_i$)$_2$); **C** – canaphite analogue (Ca_2**PP**$_i$).

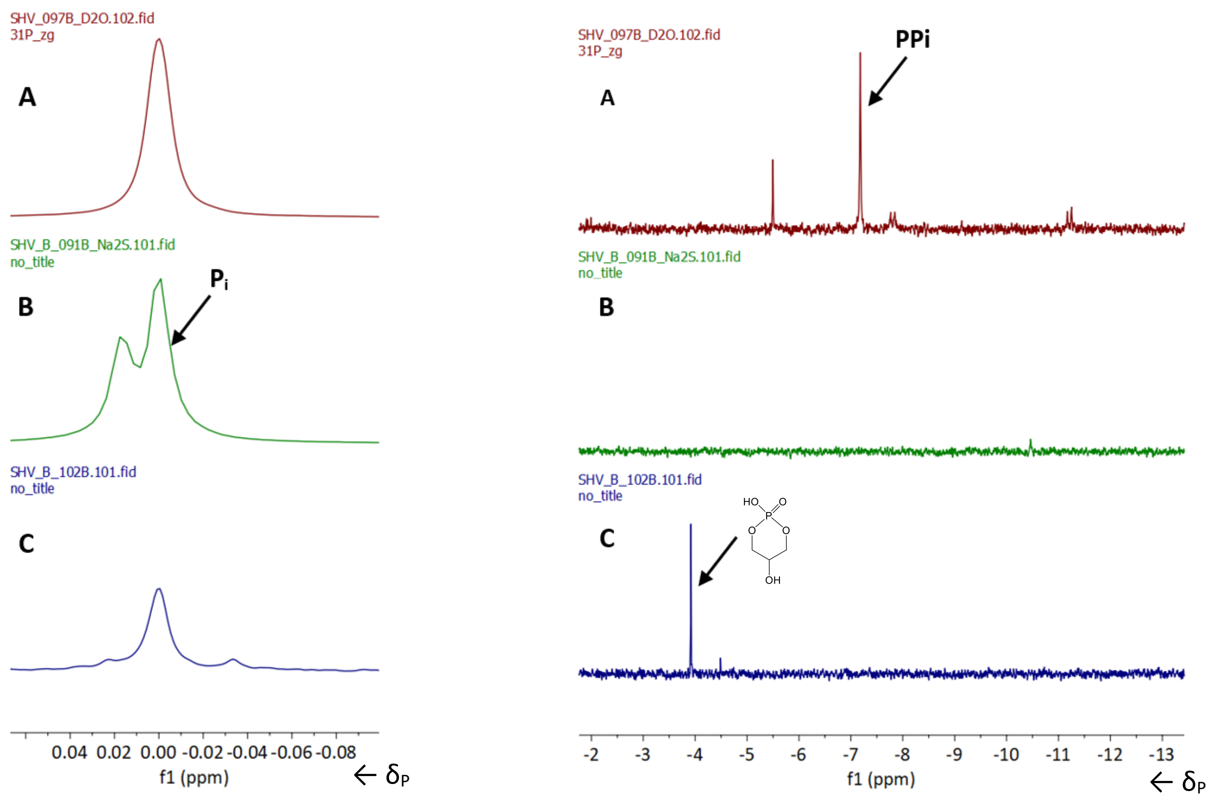

Supplementary Fig. 110. Selected zooms $^{31}P\{^{1}H\}$ NMR spectra (202 MHz) from Supplementary **Fig. 109** of crude mixture **5**:**mineral analogue**:**2a** (1:1:1), 0.5 mmol scale reaction after 120 h of heating at 115 °C, then dissolved in D_2O. **A** – struvite (NH_4Mg**P**$_i$); **B** – vivianite (Fe_3(**P**$_i$)$_2$); **C** – canaphite analogue (Ca_2**PP**$_i$).

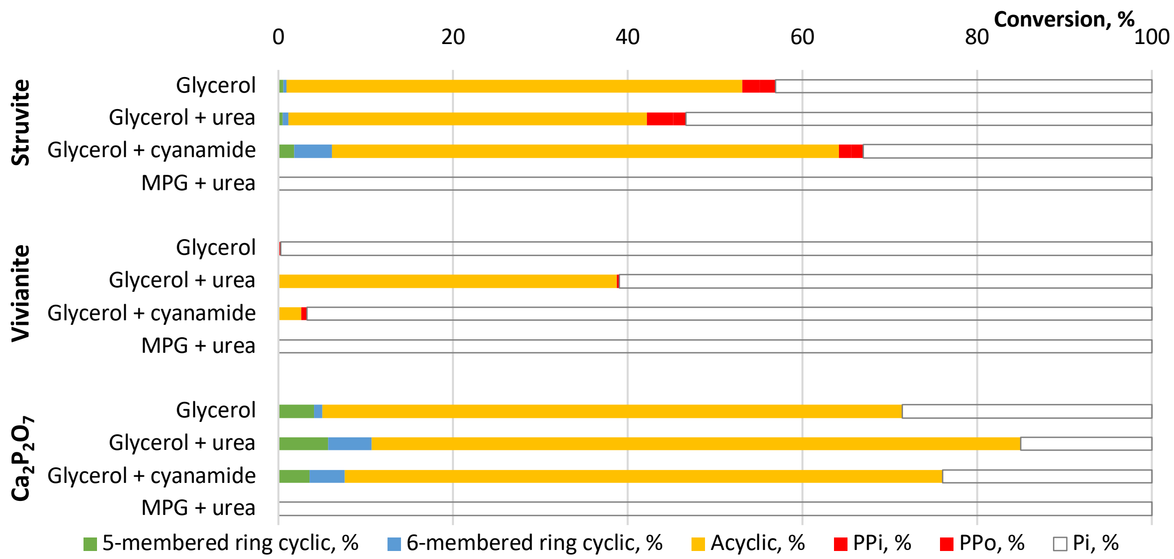

Supplementary Fig. 111. Summary of mineral-driven phosphorylations of glycerol (**5**) and MPG (**6**) after 120 h of heating at 115 °C, with and without urea (**2a**) or cyanamide (**1**), on a 0.5 mmol scale in equimolar ratios. Organic products: **cGlP** (cyclic glyceryl phosphates), **GlO1P** (glyceryl-*O*1-phosphates), **GlO2P** (glyceryl-*O*2-phosphates), **(Gl)$_2$P** (diglyceryl phosphates); sometimes **GlPP** (glyceryl diphosphates). Percent values from signal integration of quantitative $^{31}P\{^{1}H\}$ NMR spectra taken in D_2O for glycerol and DMSO-*d6* for MPG extracts (see Supplementary **Table 43**).

Supplementary Table 43. Data to Supplementary **Fig. 111**. Total conversion of initial amount of phosphates to phosphorylated organic products was calculated by subtracting inorganic compound integrals (**P$_i$**, **PP$_i$**) from the sum of all integrated ^{31}P{^{1}H} NMR peak areas.

Phosphate source	Starting molecule	Cond. agent	5-membered ring cyclic, %	6-membered ring cyclic, %	Acyclic, %	PP$_i$, %	PP$_o$, %	P$_i$, %
**Struvite $NH_4MgPO_4·6H_2O$**	**5**	-	0.56	0.33	52.23	2.00	1.81	43.07
	**5**	**2a**	0.52	0.63	41.01	3.04	1.45	53.35
	**5**	**1**	1.84	4.28	58.07	1.40	1.39	33.02
	**6**	**2a**	-	-	-	-	-	100
**Vivianite $Fe_3(PO_4)_2·8(H_2O)$**	**5**	-	-	-	-	0.24	-	99.76
	**5**	**2a**	-	-	38.75	0.26	-	60.99
	**5**	**1**	-	-	2.59	0.7	-	96.71
	**6**	**2a**	-	-	-	-	-	100
**Canaphite analogue $Ca_2P_2O_7$**	**5**	-	4.11	0.90	66.39	-	-	28.60
	**5**	**2a**	5.69	4.97	74.30	-	-	15.04
	**5**	**1**	3.57	4.05	68.45	-	-	23.93
	**6**	**2a**	-	-	-	-	-	100

7.5. Phosphorylation of nucleosides

7.5.1. Phosphorylation of adenosine in the presence of water or formamide

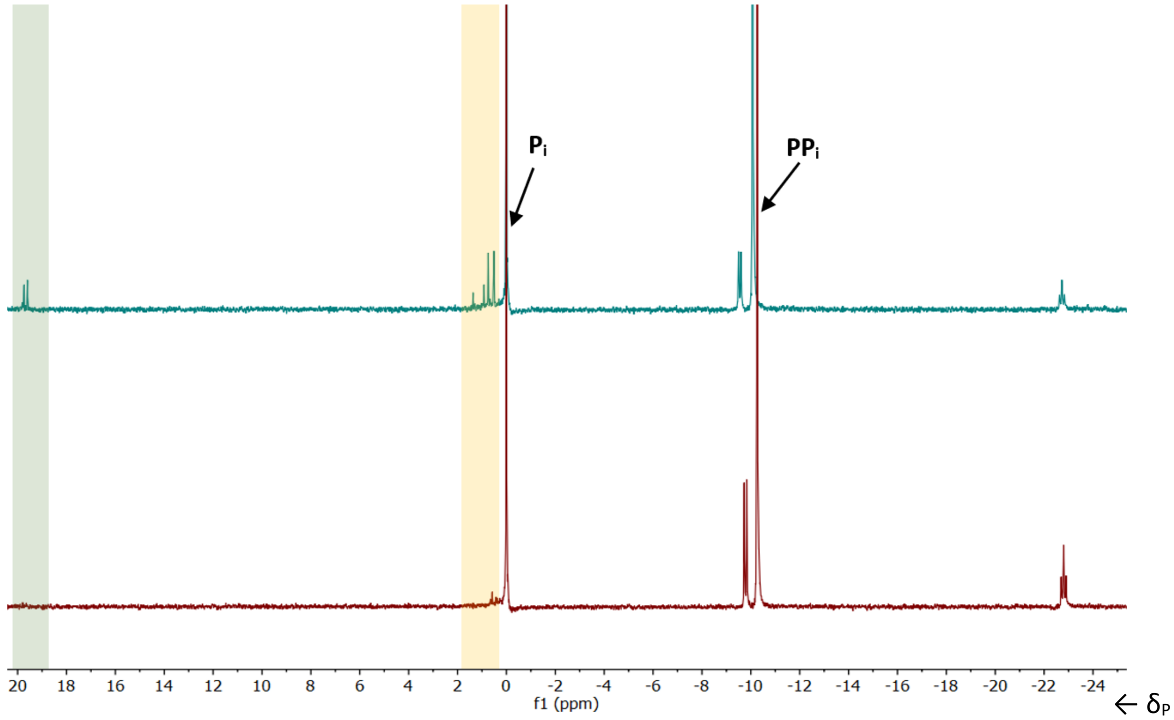

Supplementary Fig. 112. ^{31}P{^{1}H} NMR spectra (202.5 MHz, H_2O/D_2O = 9:1) 0.5 mmol scale reaction with 1 ml of H_2O added in the beginning of the reaction, after 120 h of heating at 115 °C of crude mixtures: **Lower spectrum** – **11**:**1**:**P$_i$** (1:1:1); δ$_P$ (ppm) = 0.58 (5'-, 3'- or 2'-acyclic adenosine phosphate, **Ap**, ochre), 0.00 (*s*, **P$_i$**), –9.78 (*d*, J = 20.5 Hz **PPP$_i$**), –10.26 (*s*, **PP$_i$**), –22.80 (*t*, J = 20.3 Hz **PPP$_i$**); **upper spectrum** – **11**:**2a**:**P$_i$** (1:1:1); δ$_P$ (ppm) = 19.73-19.59 (*s*, 2',3' 5-membered ring cyclic phosphates **A>p,** green), 1.36-0.51 (5'-, 3'- and 2'-acyclic adenosine phosphates, **Ap**, ochre), 0.00 (*s*, **P$_i$**), –9.55 (*d*, J = 20.5 Hz **PPP$_i$**), –10.07 (*s*, **PP$_i$**), –22.73 (*t*, J = 20.6 Hz **PPP$_i$**).

For these 'wet-to-dryness' experiments we measured ^{1}H-^{31}P HMBC spectra that did not show any cross-correlations (not shown). We concluded that all larger signals in the above ^{31}P{^{1}H} NMR spectra are due to inorganic phosphates. In both experiments the ratios of the ^{31}P{^{1}H} doublet and triplet reso-

nances integrate in a 2:1 ratio respectively, therefore, both signals belong to inorganic triphosphate. In another series of experiments water has been replaced with a small amount of formamide (two drops ≈ 0.5 mol equivalents on a 0.5 mmole scale), see the summary of results in Supplementary **Fig. 113**. In the following, all 'dry' nucleoside phosphorylations were carried out in the presence of a small amount of formamide (**3a**) as liquidiser (we use this word to distinguish physically mobilising from dissolving).

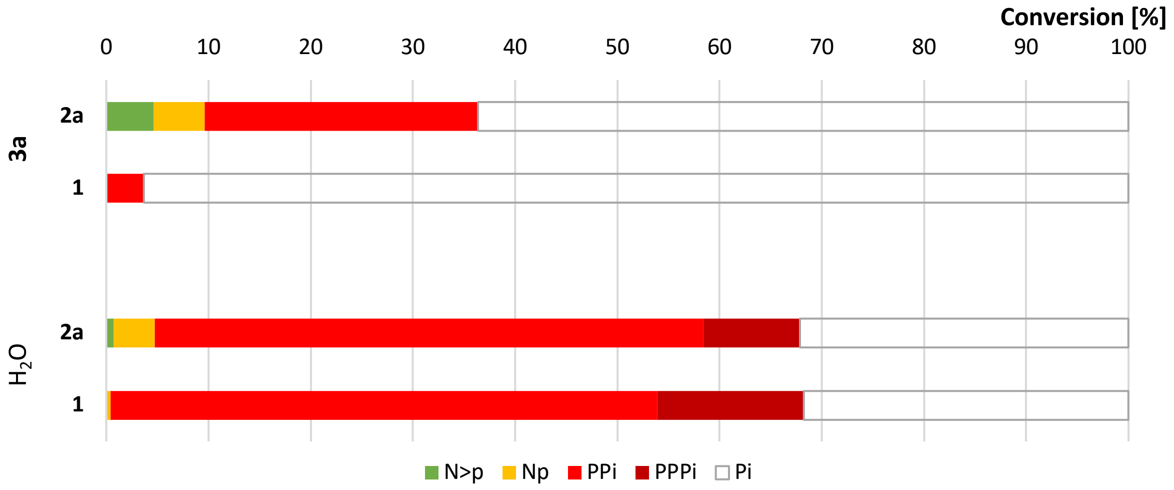

Supplementary Fig. 113. Summary of adenosine (**11**) phosphorylation in reaction **11**:**cond.agent**:**P**$_i$ (1:1:1) with 1 ml of H_2O added in the beginning of the reaction or 0.5 eq **3a** as liquidiser, 0.5 mmol scale after 120 h of heating at 115 °C, then dissolved in H_2O/D_2O = 9:1. Percent values from signal integration of quantitative $^{31}P\{^1H\}$ NMR spectrum.

Supplementary Table 44. Data to Supplementary **Fig. 113**. Total conversion of initial amount of **P**$_i$ to phosphorylated organic products was calculated by subtracting inorganic compound integrals (**P**, **PP**$_i$ and **PPP**$_i$) from the sum of all integrated $^{31}P\{^1H\}$ NMR peak areas (in H_2O/D_2O = 9:1). Products **ApA** and **p<A** (5',3'-cyclic adenosine phosphate) were not detected.

Liquidiser	Condensing agent	A>p, %	Ap, %	PP$_i$, %	PPP$_i$, %	P$_i$, %
**3a**	**2a**	4.64	4.99	26.74	-	63.63
	**1**	0.13	-	3.55	-	96.32
**H_2O**	**2a**	0.76	3.99	53.68	9.45	32.12
	**1**	-	0.40	53.51	14.34	31.75

7.5.2. Experiments with different condensing agents (cyanamide 1 and urea 2a)

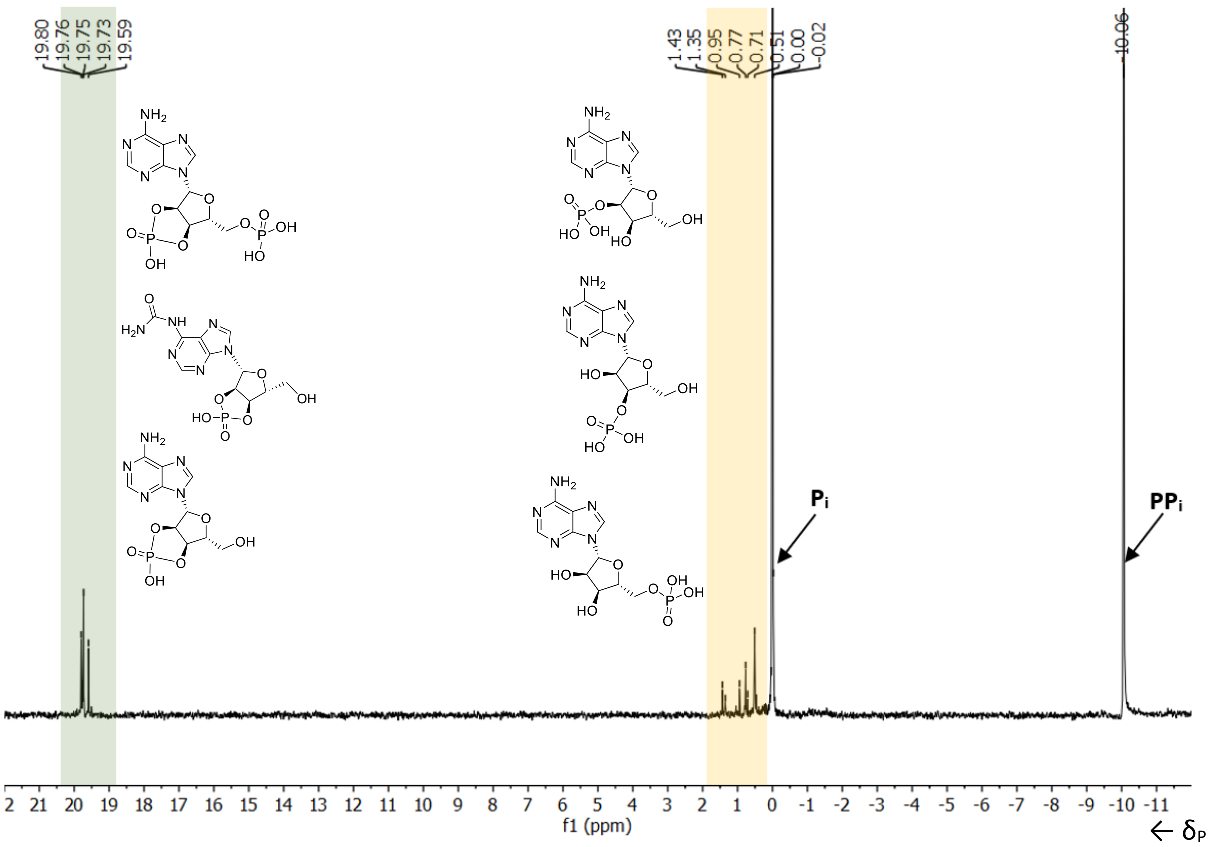

Supplementary Fig. 114. $^{31}P\{^{1}H\}$ NMR spectrum (202.5 MHz) of crude mixture containing **11**, **2a** and $\mathbf{P_i}$ (1:1:1) with 0.5 eq **3a** as liquidiser, 0.5 mmol scale reaction after 120 h of heating at 115 °C, then dissolved in H_2O/D_2O = 9:1. δ_P (ppm) = 19.80-19.59 (*s*, 2',3'-5-membered ring cyclic phosphate **A>p,** green), 1.43-0.51 (5'-, 3'- or 2'-acyclic adenosine-phosphates, **Ap**, ochre), 0.00 (*s*, $\mathbf{P_i}$), –10.06 (*s*, $\mathbf{PP_i}$). Some acyclic phosphate signals can be due to di- or tri-phosphorylated or/and carbamoylated adenosine.

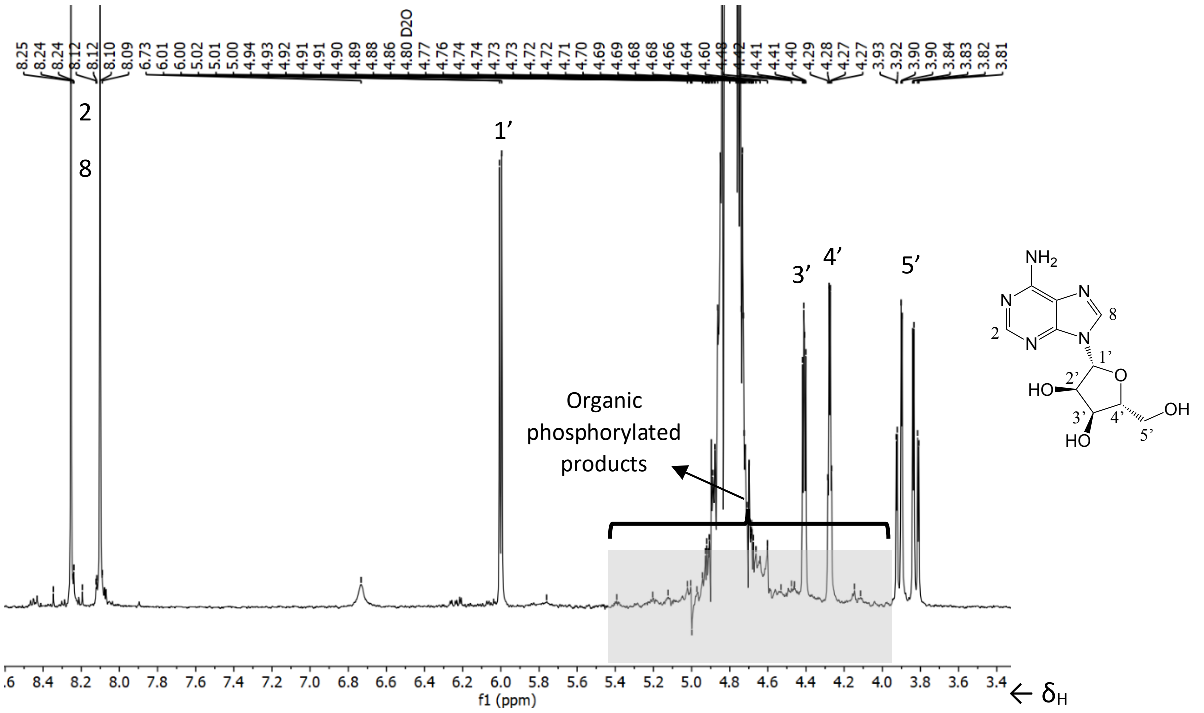

Supplementary Fig. 115. ^{1}H NMR spectrum (500 MHz) of crude mixture containing **11**, **2a** and **P**$_i$ (1:1:1) with 0.5 eq **3a** as liquidiser, 0.5 mmol scale reaction after 120 h of heating at 115 °C, then dissolved in H_2O/D_2O = 9:1. δ_H (ppm) = 8.15 (*s*, 1H), 8.00 (*s*, 1H), 5.90 (*d*, J = 6.1 Hz, 1H), 4.31 (*dd*, J = 5.2, 3.4 Hz, 1H), 4.18 (*q*, J = 3.3 Hz, 1H), 3.81 (*dd*, J = 12.9, 2.8 Hz, 1H), 3.72 (*dd*, J = 12.9, 3.6 Hz, 1H). Spectrum labelled with proton resonances of unreacted nucleoside (H2’ hidden in the solvent peak). Grey zone shows cross-correlations with ^{31}P nuclei, see Supplementary **Fig. 116**.

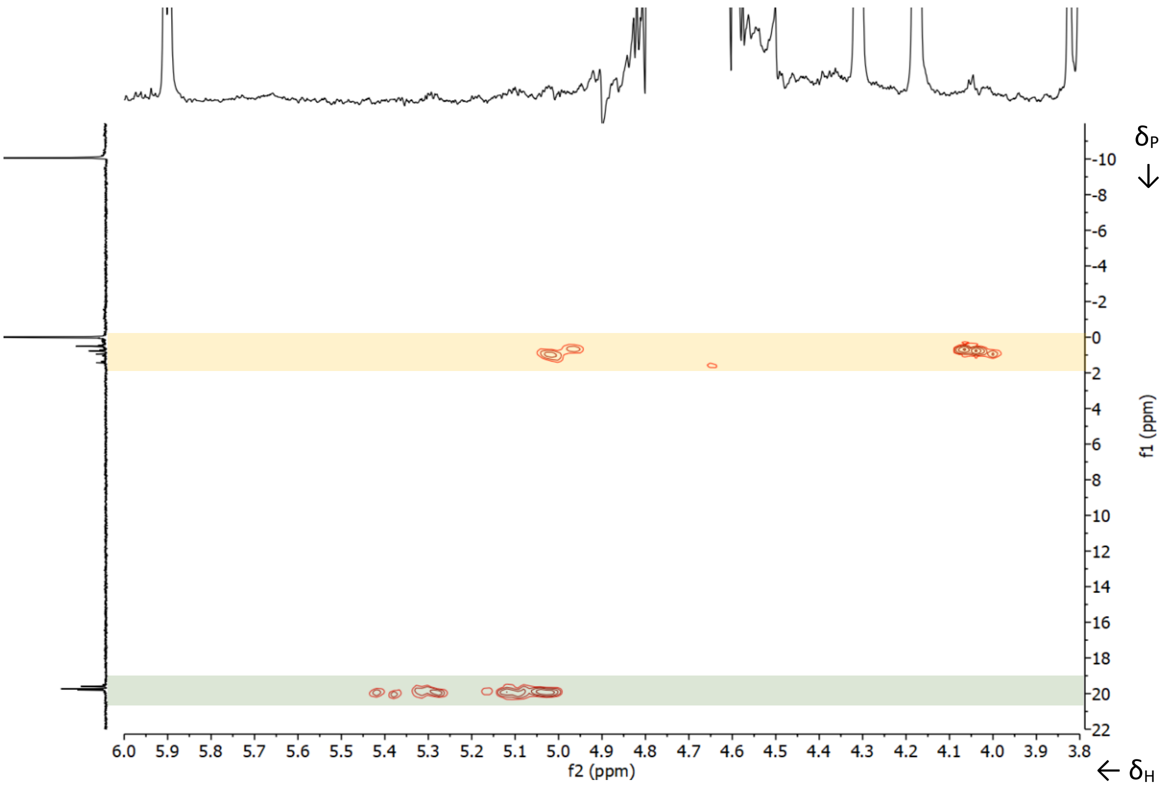

Supplementary Fig. 116. ^{1}H-^{31}P HMBC spectrum (500 MHz for ^{1}H [horizontal axis], 202.5 MHz for $^{31}P\{^{1}H\}$ [vertical axis]) of crude mixture containing **11**, **2a** and **P**$_i$ (1:1:1) with 0.5 eq **3a** as liquidiser, 0.5 mmol scale reaction after 120 h of heating at 115 °C, then dissolved in H_2O/D_2O = 9:1. Ochre (**Ap**) and green (**A>p**) zones highlight organic products of phosphorylation.

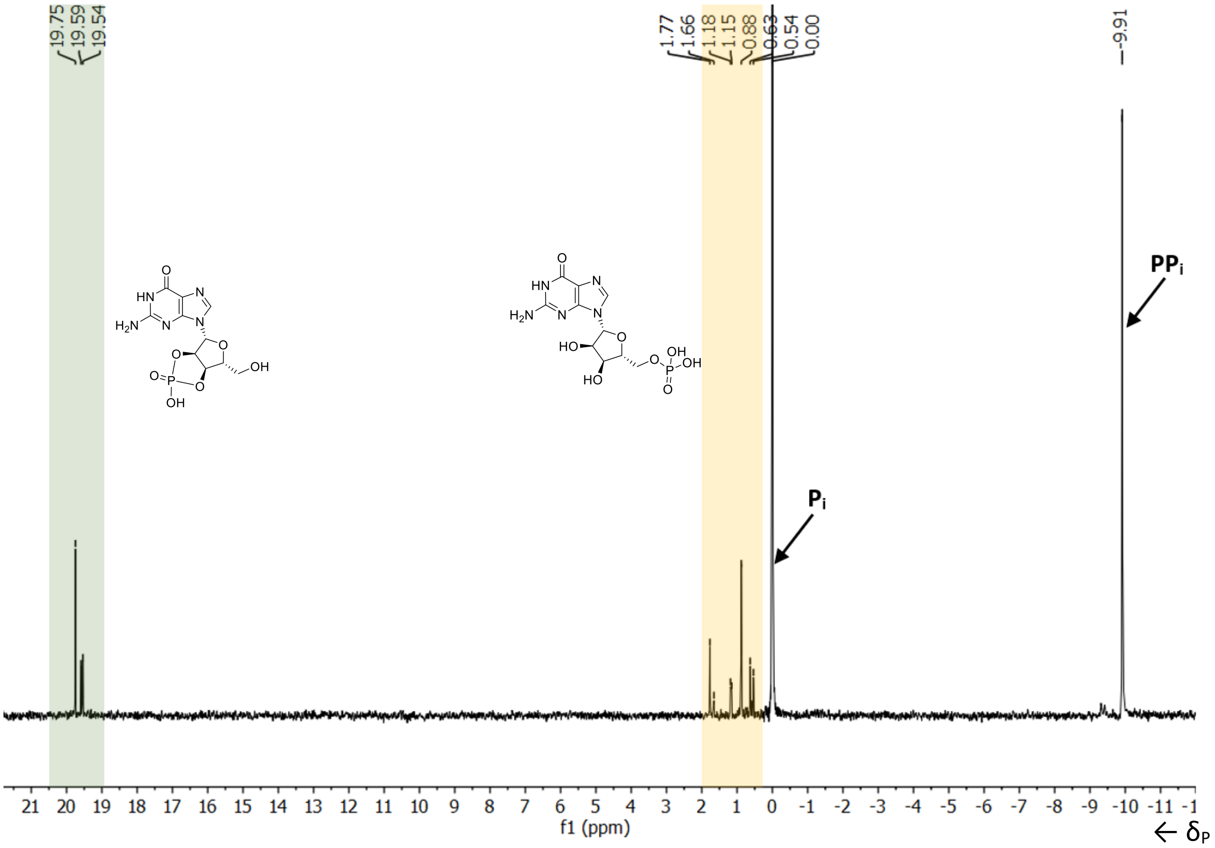

Supplementary Fig. 117. $^{31}P\{^1H\}$ NMR spectrum (202.5 MHz) of crude mixture containing **12**, **2a** and **P**$_i$ (1:1:1) with 0.5 eq **3a** as liquidiser, 0.5 mmol scale reaction after 120 h of heating at 115 °C, then dissolved in H_2O/D_2O = 9:1. δ_P (ppm) = 19.75-19.54 (*s*, 2',3' 5-membered ring cyclic phosphate **G>p,** green), 1.77-0.54 (5', 3' or 2' acyclic guanosine-phosphates, **Gp**, ochre), 0.00 (*s*, **P**$_i$), –9.91(*s*, **PP**$_i$). Structures represent main groups of organic phosphorylated products.

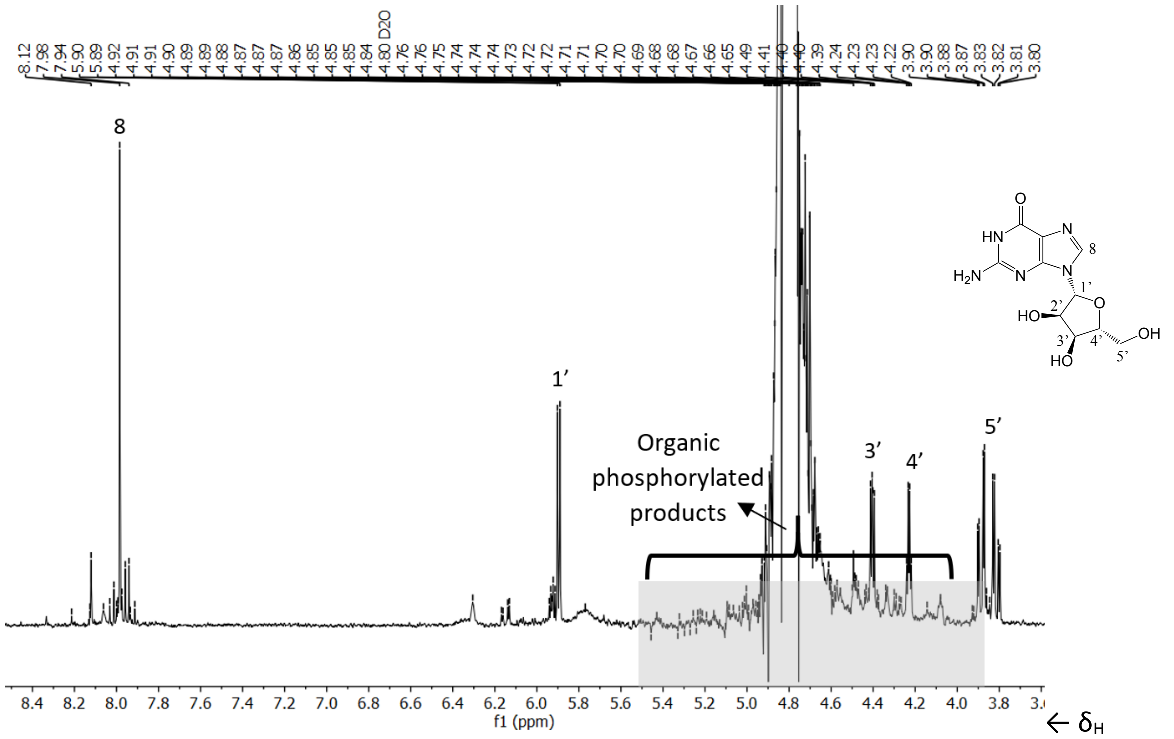

Supplementary Fig. 118. ^{1}H NMR spectrum (500 MHz) of crude mixture containing **12**, **2a** and **P**$_i$ (1:1:1) with 0.5 eq **3a** as liquidiser, 0.5 mmol scale reaction after 120 h of heating at 115 °C, then dissolved in H_2O/D_2O = 9:1. δ_H (ppm) = 7.88 (*s*, 1H), 5.80 (*d*, J = 5.9 Hz, 1H), 4.30 (*dd*, J = 5.3, 3.7 Hz, 1H), 4.13 (*d*, J = 3.5 Hz, 1H), 3.79 (*dd*, J = 12.7, 3.1 Hz, 1H), 3.71 (*dd*, J = 12.7, 4.1 Hz, 1H). Spectrum labelled with proton resonances of unreacted nucleoside (H2’ hidden in the solvent peak). Grey zone shows cross-correlations with ^{31}P nuclei, see Supplementary **Fig. 119**.

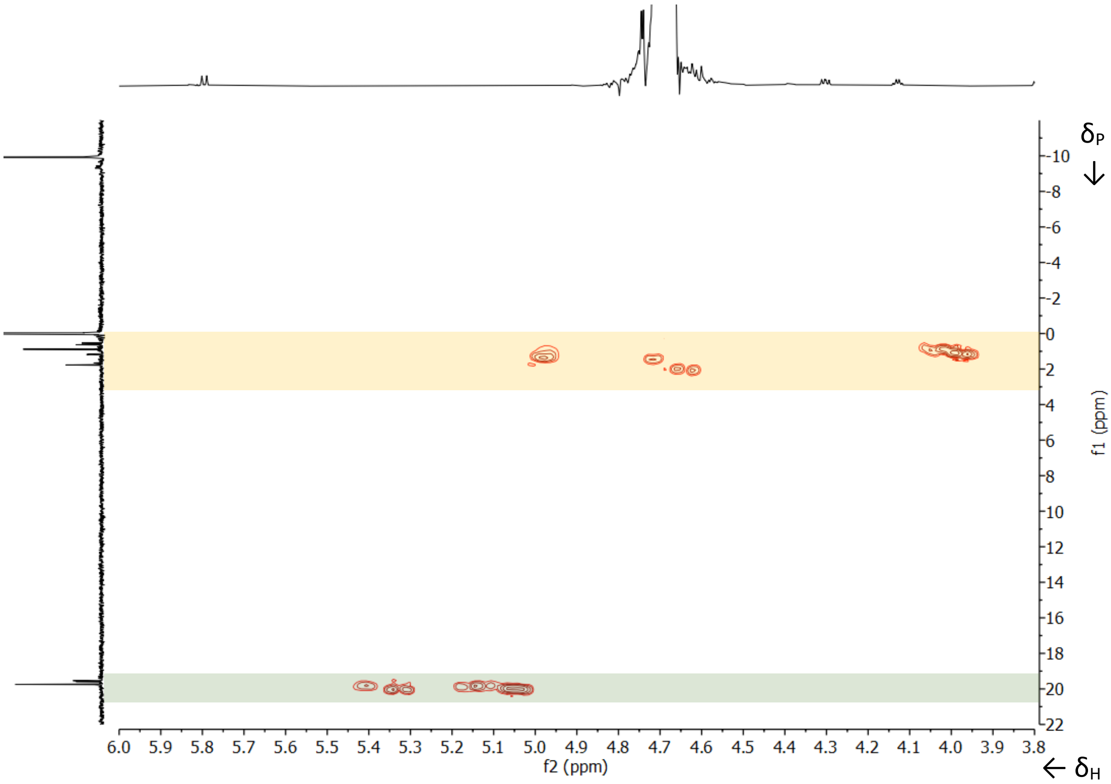

Supplementary Fig. 119. ^{1}H-^{31}P HMBC spectrum (500 MHz for ^{1}H [horizontal axis], 202.5 MHz for $^{31}P\{^1H\}$ [vertical axis]) of crude mixture containing **12**, **2a** and **P**$_i$ (1:1:1) with 0.5 eq **3a** as liquidiser, 0.5 mmol scale reaction after 120 h of heating at 115 °C, then dissolved in H_2O/D_2O = 9:1. Ochre (**Gp**) and green (**G>p**) zones highlight organic products of phosphorylation.

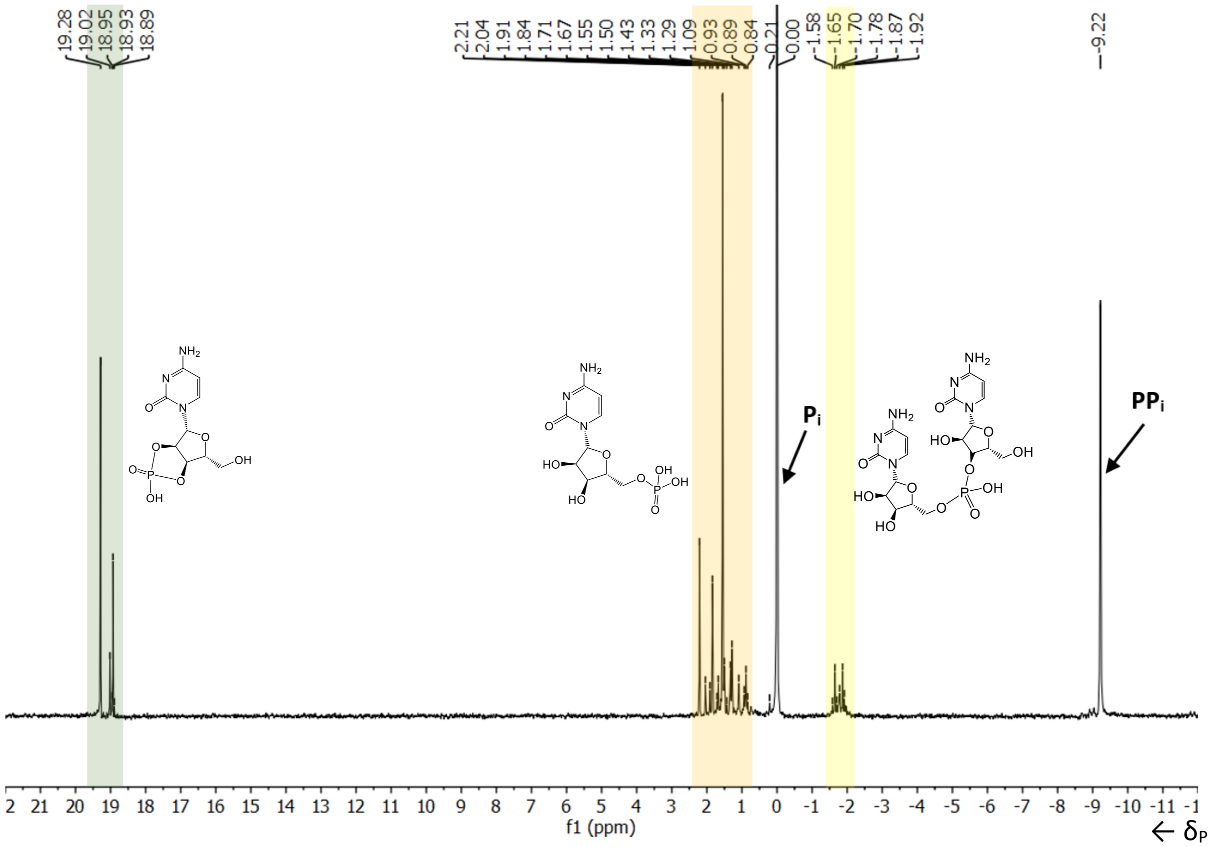

Supplementary Fig. 120. $^{31}P\{^{1}H\}$ NMR spectrum (202.5 MHz) of crude mixture containing **13**, **2a** and $\mathbf{P_i}$ (1:1:1) with 0.5 eq **3a** as liquidiser, 0.5 mmol scale reaction after 120 h of heating at 115 °C, then dissolved in H_2O/D_2O = 9:1. δ_P (ppm) = 19.28-18.89 (*s*, 2',3' 5-membered ring cyclic phosphate **C>p,** green), 2.21-0.21 (5', 3' or 2' acyclic cytidine-phosphates, **Cp**, ochre), 0.00 (*s*, $\mathbf{P_i}$), –1.58 to –1.92 (acyclic dicytidine phosphates, **CpC**, yellow), –9.22 (*s*, $\mathbf{PP_i}$). Structures represent main groups of organic phosphorylated products (isomers, more phosphorylated and carbamoylated structures not shown).

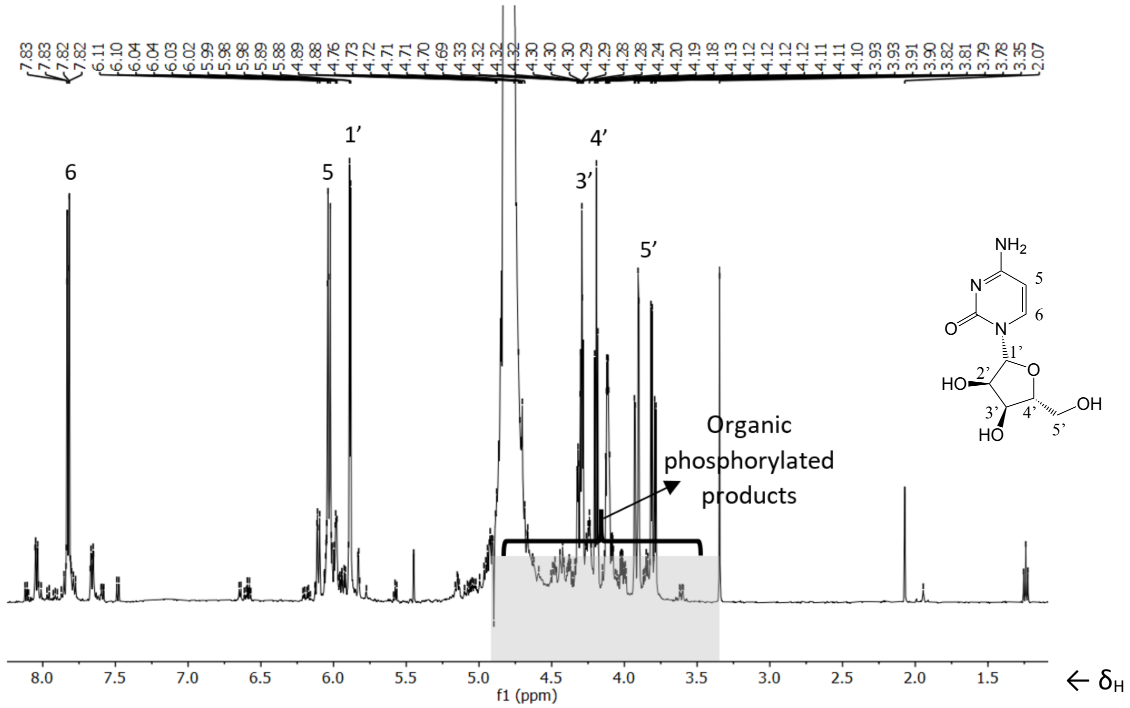

Supplementary Fig. 121. ^{1}H NMR spectrum (500 MHz) of crude mixture containing **13**, **2a** and **P**$_i$ (1:1:1) with 0.5 eq **3a** as liquidiser, 0.5 mmol scale reaction after 120 h of heating at 115 °C, then dissolved in H_2O/D_2O = 9:1. δ_H (ppm) = 7.72 (*dd*, J = 7.6, 1.1 Hz, 1H), 5.93 (*dd*, J = 7.5, 1.3 Hz, 1H), 5.79 (*d*, J = 4.0 Hz, 1H), 4.19 (*ddd*, J = 5.2, 4.0, 1.1 Hz, 1H), 4.09 (*t*, J = 5.7 Hz, 1H), 3.82 (*dd*, J = 12.8, 2.9 Hz, 1H), 3.70 (*dd*, J = 12.8, 4.5 Hz, 1H). Spectrum labelled with proton resonances of unreacted nucleoside (H2’ hidden in the solvent peak). Grey zone shows cross-correlations with ^{31}P nuclei, see Supplementary **Fig. 122**.

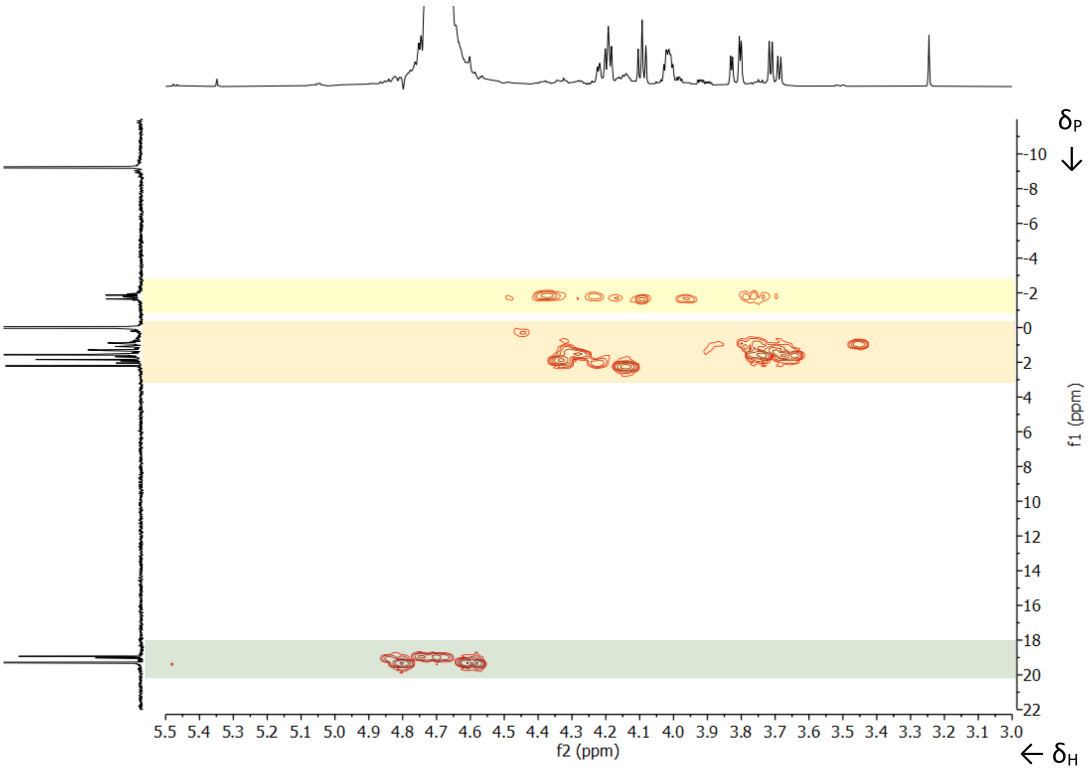

Supplementary Fig. 122. ^{1}H-^{31}P HMBC spectrum (500 MHz for ^{1}H [horizontal axis], 202.5 MHz for $^{31}P\{^{1}H\}$ [vertical axis]) of crude mixture containing **13**, **2a** and **P**$_i$ (1:1:1) with 0.5 eq **3a** as liquidiser, 0.5 mmol scale reaction after 120 h of heating at 115 °C, then dissolved in H_2O/D_2O = 9:1. Ochre (**Cp**), yellow (**CpC**) and green (**C>p**) zones highlight organic products of phosphorylation.

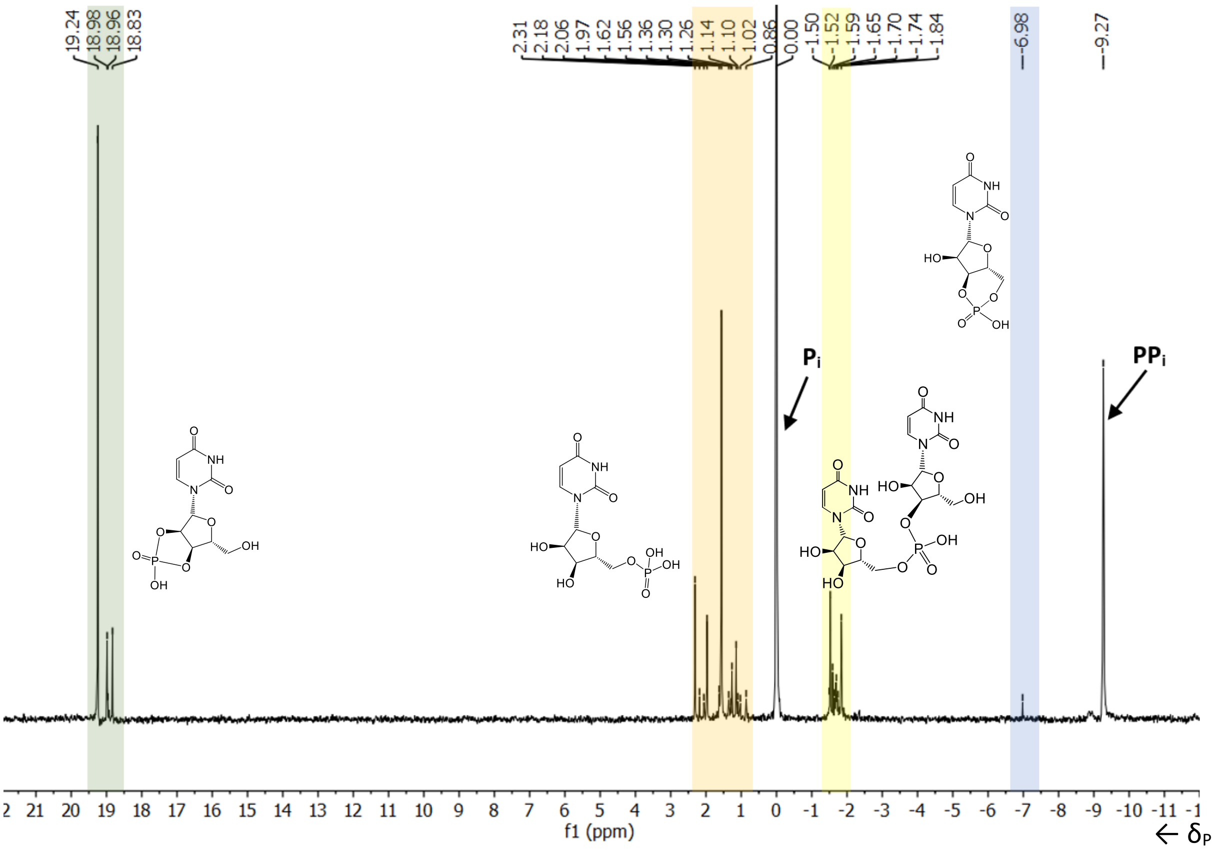

Supplementary Fig. 123. $^{31}P\{^1H\}$ NMR spectrum (202.5 MHz) of crude mixture containing **14**, **2a** and **P**$_i$ (1:1:1) with 0.5 eq **3a** as liquidiser, 0.5 mmol scale reaction after 120 h of heating at 115 °C, then dissolved in H_2O/D_2O = 9:1. δ_P (ppm) = 19.24-18.83 (*s*, 2',3' 5-membered ring cyclic phosphate **U>p,** green), 2.31-0.86 (5', 3' or 2' acyclic uridine-phosphates, **Up**, ochre), 0.00 (*s*, **P**$_i$), –1.50 to –1.84 (acyclic diuridine phosphates, **UpU**, yellow), –6.98 (*s*, 5',3' 6-membered ring cyclic phosphate **p<U**, blue), –9.27 (*s*, **PP**$_i$). Structures represent main groups of organic phosphorylated products (isomers, more phosphorylated and carbamoylated structures not shown).

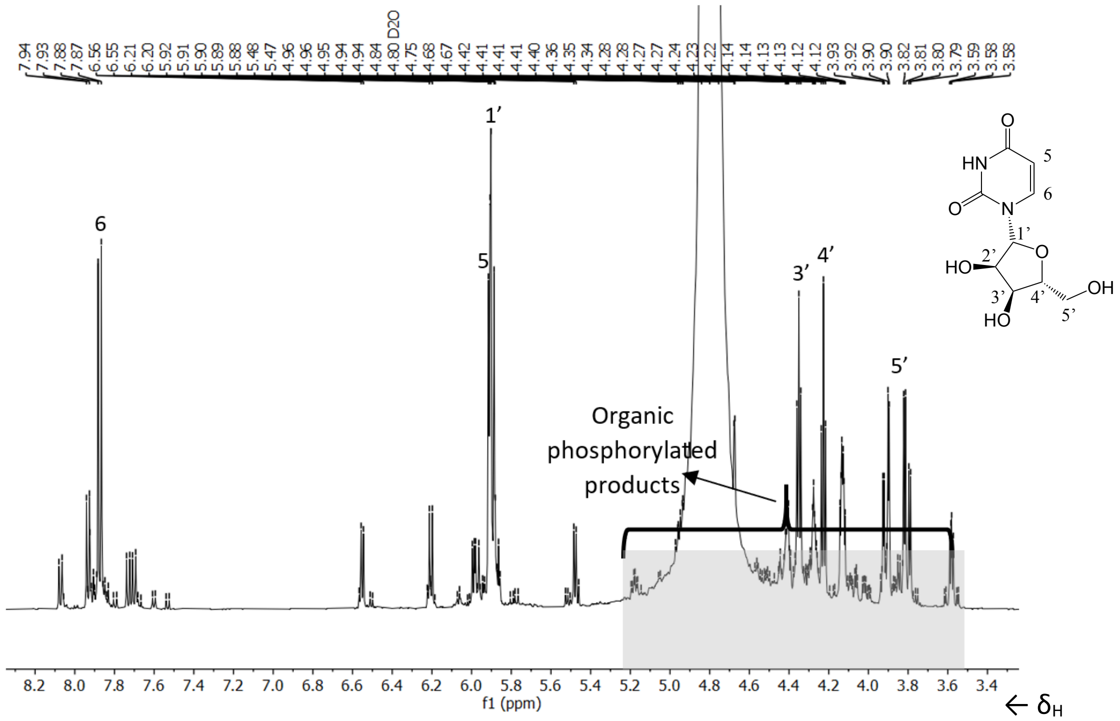

Supplementary Fig. 124. ^{1}H NMR spectrum (500 MHz) of crude mixture containing **14**, **2a** and **P$_i$** (1:1:1) with 0.5 eq **3a** as liquidiser, 0.5 mmol scale reaction after 120 h of heating at 115 °C, then dissolved in H_2O/D_2O = 9:1. δ_H (ppm) = ^{1}H NMR (500 MHz, D_2O) δ 7.87 (*d*, J = 8.1 Hz, 1H), 5.93-5.88 (*m*, 3H), 4.35 (*t*, J = 4.9 Hz, 1H), 4.23 (*t*, J = 5.5 Hz, 1H), 4.13 (*td,* J = 4.8, 2.9 Hz, 1H), 3.91 (*dd*, J = 12.8, 3.0 Hz, 1H), 3.80 (*dd*, J = 12.8, 4.4 Hz, 1H). Spectrum labelled with proton resonances of unreacted nucleoside (H2’ hidden in the solvent peak). Grey zone shows cross-correlations with ^{31}P nuclei, see Supplementary **Fig. 125**.

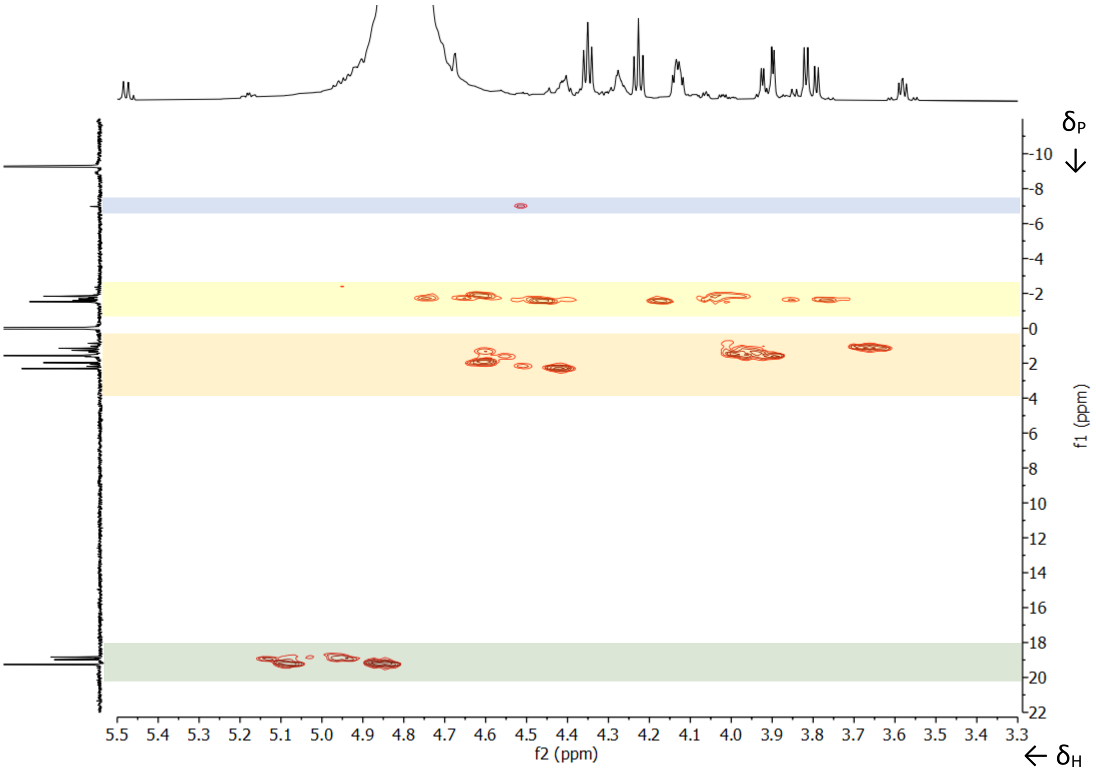

Supplementary Fig. 125. ^{1}H-^{31}P HMBC spectrum (500 MHz for ^{1}H [horizontal axis], 202.5 MHz for $^{31}P\{^{1}H\}$ [vertical axis]) of crude mixture containing **14**, **2a** and **P$_i$** (1:1:1) with 0.5 eq **3a** as liquidiser, 0.5 mmol scale reaction after 120 h of heating at 115 °C, then dissolved in H_2O/D_2O = 9:1. Blue (**U>p**), ochre (**Up**), yellow (**UpU**) and green (**p<U**) zones highlight organic products of phosphorylation.

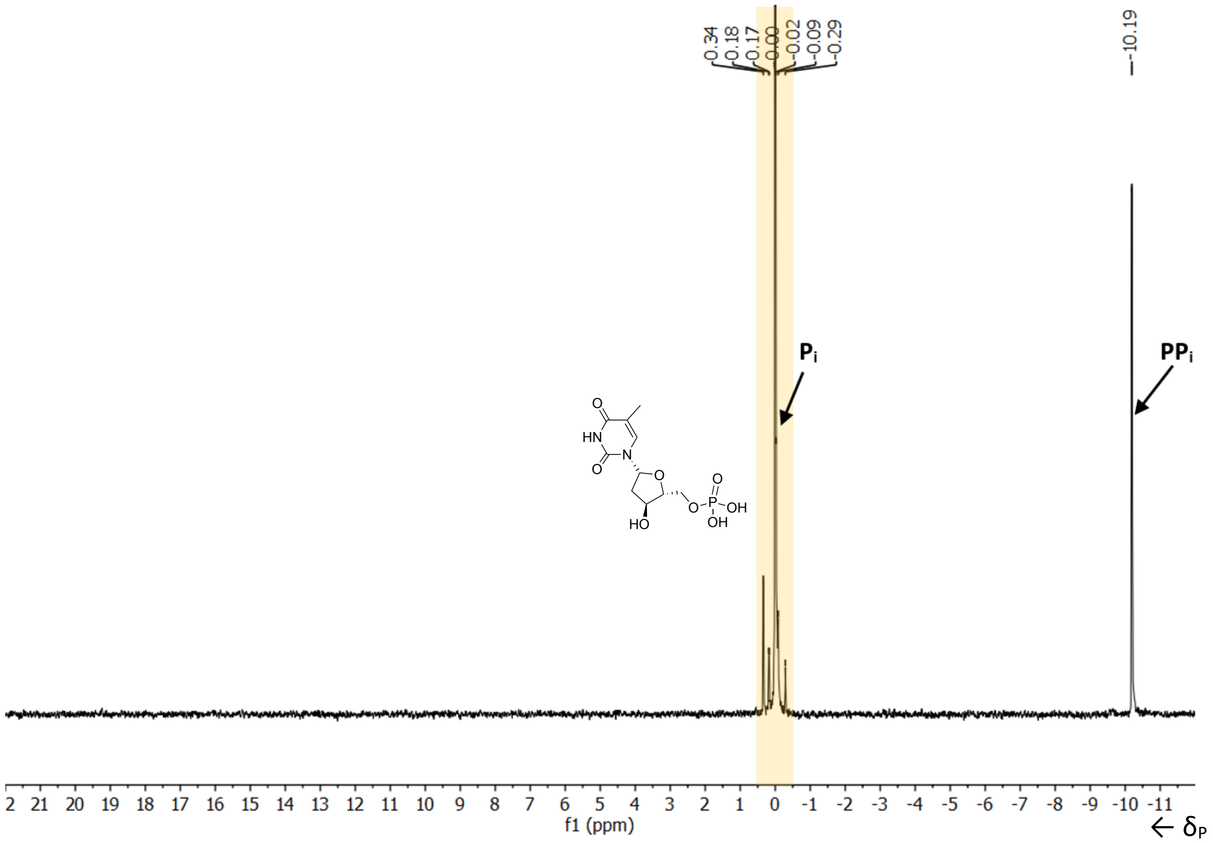

Supplementary Fig. 126. $^{31}P\{^1H\}$ NMR spectrum (202.5 MHz) of crude mixture containing **15**, **2a** and **P$_i$** (1:1:1) with 0.5 eq **3a** as liquidiser, 0.5 mmol scale reaction after 120 h of heating at 115 °C, then dissolved in H_2O/D_2O = 9:1. δ_P (ppm) = from 0.34 to –0.29 (5’-, 3’- or 2’-acyclic thymidine-phosphates, **Tp**, ochre), 0.00 (*s*, **P$_i$**), –10.19 (*s*, **PP$_i$**). Structure represents main group of organic phosphorylated products (isomers, more phosphorylated and carbamoylated structures not shown).

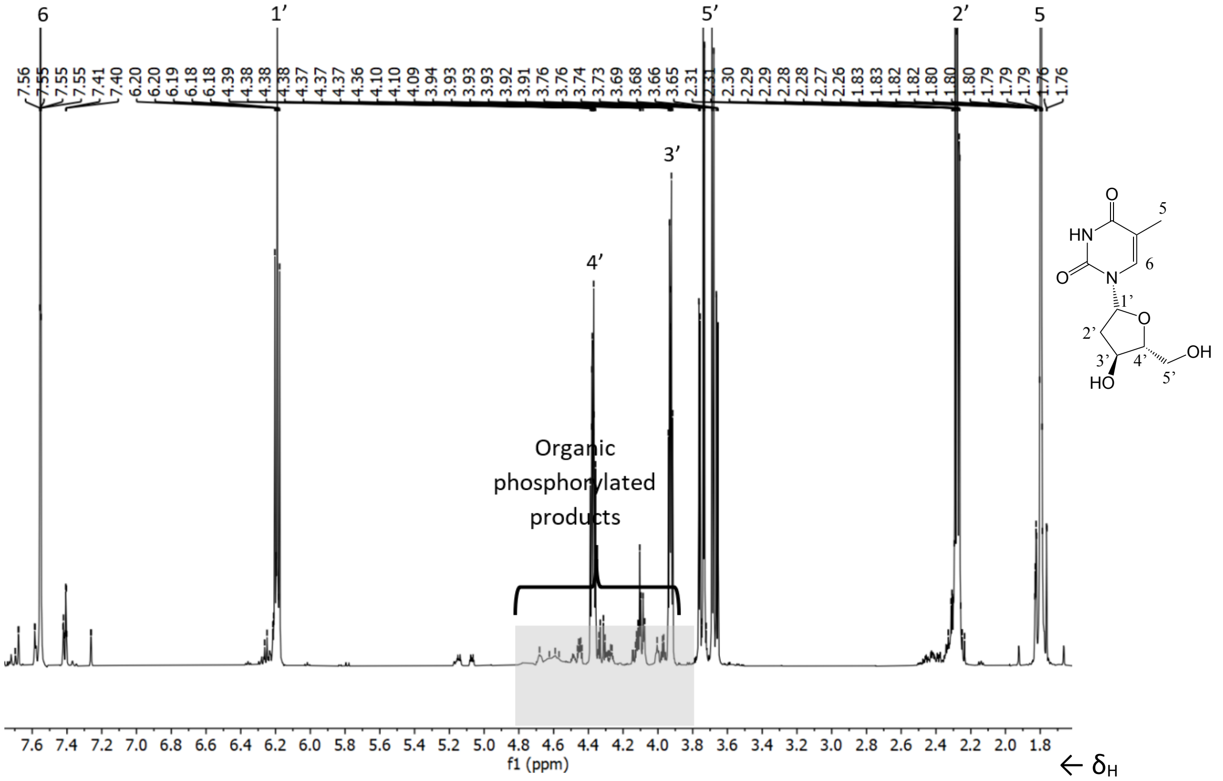

Supplementary Fig. 127. ^{1}H NMR spectrum (500 MHz, H_2O signal suppressed) of crude mixture containing **15**, **2a** and **P**$_i$ (1:1:1) with 0.5 eq **3a** as liquidiser, 0.5 mmol scale reaction after 120 h of heating at 115 °C, then dissolved in H_2O/D_2O = 9:1. δ_H (ppm) = 7.55 (*q*, J = 1.2 Hz, 1H), 6.19 (*t*, J = 6.8 Hz, 1H), 4.37 (*ddd*, J = 6.1, 4.8, 3.9 Hz, 1H), 3.93 (*dt*, J = 5.1, 3.7 Hz, 1H), 3.75 (*dd*, J = 12.5, 3.6 Hz, 1H), 3.67 (*dd*, J = 12.5, 5.0 Hz, 1H), 2.30-2.26 (*m*, 2H), 1.80 (*d*, J = 1.2 Hz, 3H). Spectrum labelled with proton resonances of unreacted 2’-deoxynucleoside. Grey zone shows cross-correlations with ^{31}P nuclei, see Supplementary **Fig. 128**.

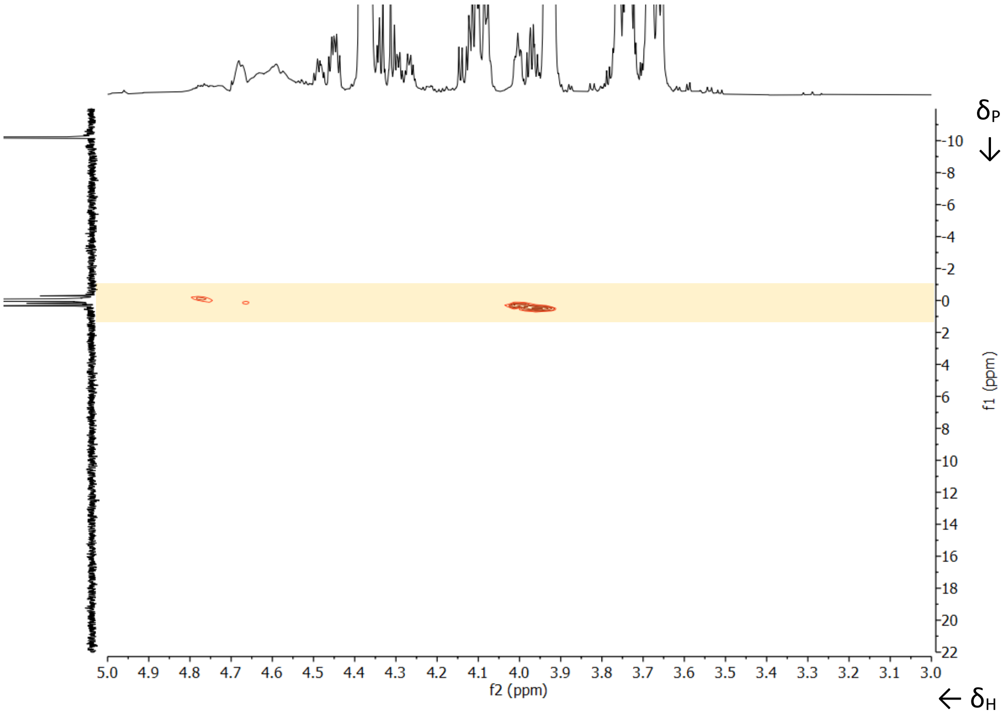

Supplementary Fig. 128. ^{1}H-^{31}P HMBC spectrum (500 MHz for ^{1}H [horizontal axis], 202.5 MHz for ^{31}P{^{1}H} [vertical axis]) of crude mixture containing **15**, **2a** and **P**$_i$ (1:1:1) with 0.5 eq **3a** as liquidiser, 0.5 mmol scale reaction after 120 h of heating at 115 °C, then dissolved in H_2O/D_2O = 9:1. Ochre (**Tp**) zone highlights acyclic organic products of phosphorylation.

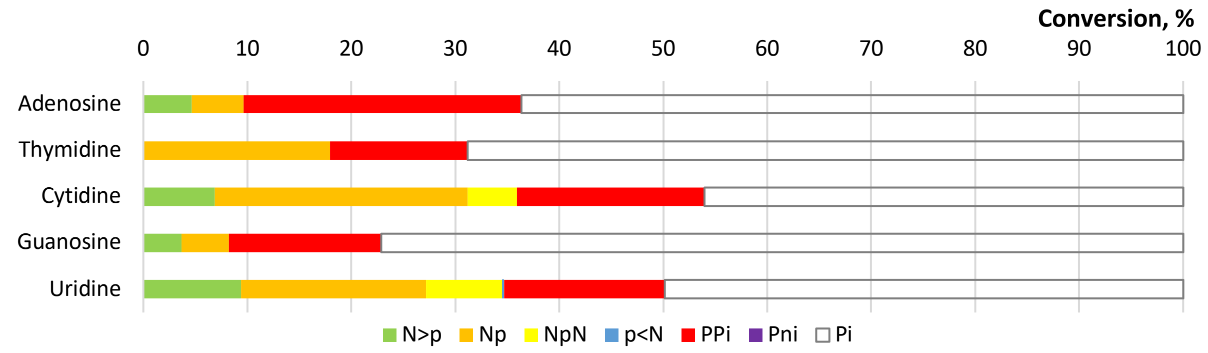

Supplementary Fig. 129. Summary of urea-assisted phosphorylation of nucleosides **N** (**11**-**15**) in **N**:**2a**:**P**$_i$ (1:1:1) with 0.5 eq **3a** as liquidiser, 0.5 mmol scale reaction after 120 h of heating at 115 °C, then dissolved in H_2O/D_2O = 9:1. Percent values from signal integration of quantitative $^{31}P\{^1H\}$ NMR spectra (Supplementary **Fig. 114**-Supplementary **Fig. 126**). N>p, p<N : 2',3'-, and resp., 5',3'-cyclic nucleos(t)ide phosphates.

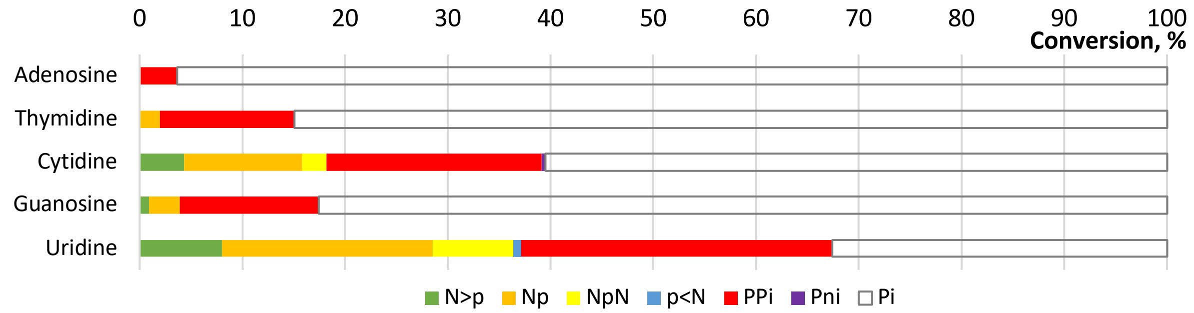

Supplementary Fig. 130. Summary of cyanamide-assisted phosphorylation of nucleosides **N** (**11**-**15**) phosphorylation in **N**:**1**:**P**$_i$ (1:1:1) with 0.5 eq **3a** as liquidiser, 0.5 mmol scale reaction after 120 h of heating at 115 °C, then dissolved in H_2O/D_2O = 9:1. Percent values from signal integration of quantitative $^{31}P\{^1H\}$ NMR spectra. N>p, p<N : 2',3'-, and resp., 5',3'-cyclic nucleos(t)ide phosphates.

Supplementary Table 45. Data to Supplementary **Fig. 129** and Supplementary **Fig. 130**. Total conversion of initial amount of **P**$_i$ to phosphorylated organic products was calculated by subtracting inorganic compound integrals (**P**$_i$, **PP**$_i$ and **P**$_{ni}$) from the sum of all integrated ^{31}P NMR peak areas (in H_2O/D_2O = 9:1). **N>p**, **p<N** : 2',3'-, and resp., 5',3'-cyclic nucleos(t)ide phosphates.

Condensing agent	Nucleoside	N>p, %	Np, %	NpN, %	p<N, %	PP$_i$, %	P$_{ni}$, %	P$_i$, %
	**11**	4.64	4.99	-	-	26.74	-	63.63
	**12**	3.70	4.54	-	-	14.62	-	77.14
**2a**	**13**	6.90	24.26	4.78	-	18.02	-	46.04
	**14**	9.41	17.78	7.32	0.19	15.46	-	49.84
	**15**	-	17.96	-	-	13.22	-	68.82
	**11**	0.13	-	-	-	3.55	-	96.32
	**12**	0.91	2.98	-	-	13.57	-	82.54
**1**	**13**	4.37	11.42	2.38	-	20.96	0.36	60.51
	**14**	8.02	20.54	7.84	0.75	30.31	-	32.54
	**15**	-	2.01	-	-	13.08	-	84.91

The phosphorylation experiments of adenosine (**11**) where urea (**2a**) was replaced with N-methylformamide (**3c**) or N-methylacetamide (**4c**) did not produce any organic products of phosphorylation at 115 °C.

7.5.3. Temperature dependence of urea- and cyanamide-assisted adenosine phosphorylation

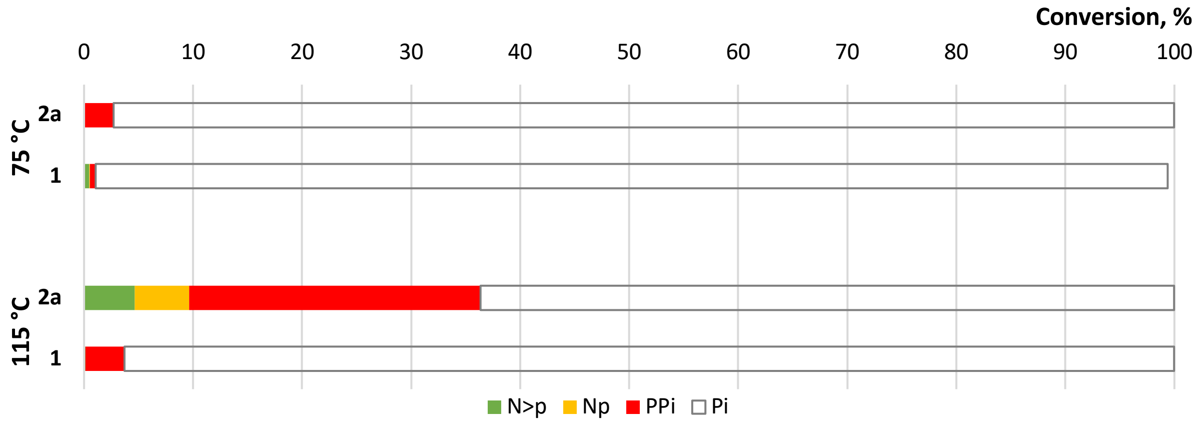

Supplementary Fig. 131. Summary of adenosine (**11**) phosphorylation in reaction **11**:**cond.agent**:$\mathbf{P_i}$ (1:1:1) with 0.5 eq **3a** as liquidiser, 0.5 mmol scale reaction after 120 h of heating at 75 or 115 °C, then dissolved in H_2O/D_2O = 9:1. Percent values from signal integration of quantitative $^{31}P\{^1H\}$ NMR spectrum. N>p : 2',3'-cyclic nucleoside phosphates.

Supplementary Table 46. Data to Supplementary **Fig. 131**. Total conversion of initial amount of $\mathbf{P_i}$ to phosphorylated organic products was calculated by subtracting inorganic compound integrals (**P**, and $\mathbf{PP_i}$) from the sum of all integrated $^{31}P\{^1H\}$ NMR peak areas (in H_2O/D_2O = 9:1). Products **ApA** and **p<A** (5',3'-cyclic adenosine phosphate) were not detected. **A>p** : 2',3'-cyclic adenosine phosphate.

Temperature, °C	Condensing agent	A>p, %	Ap, %	PP_i, %	P_i, %
75	**2a**	-	-	2.68	97.32
	**1**	0.46	0.10	0.53	98.31
115	**2a**	4.64	4.99	26.74	63.63
	**1**	0.13	-	3.55	96.32

7.5.4. Effect of urea excess on phosphorylation of nucleosides

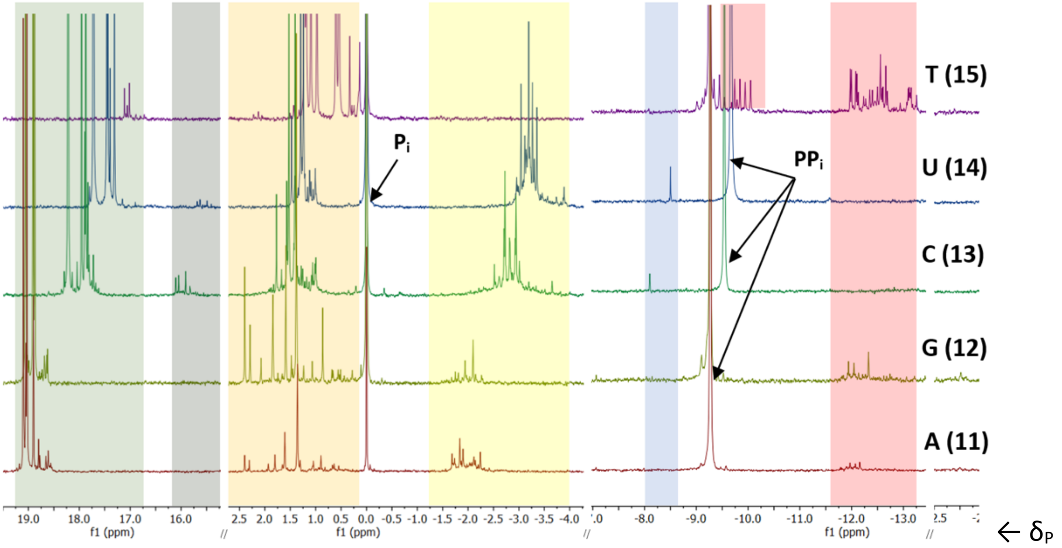

Supplementary Fig. 132. Stack of $^{31}P\{^{1}H\}$ NMR spectra (202.5 MHz, H_2O/D_2O = 9:1) of nucleoside (**N**) phosphorylations on a 0.5 mmol scale reactions of crude mixtures of **N**:**2a**:$\mathbf{P_i}$ (1:4:1) with 0.5 eq **3a** as liquidiser after 120 h of heating at 115 °C. δ_P (ppm) = 19.0-17.5 (*s*, 2',3' 5-membered ring cyclic phosphate **N>p,** green), 16.5-15.0 (*s*, 2',3' 5-membered ring cyclic diphosphate **pN>p**, grey, identified by analogy with intense acyclic dinucleotide signals), 2.5-0.5 (5', 3' or 2' acyclic phosphates, **pN** or **Np**, ochre), 0.00 (*s*, $\mathbf{P_i}$), from –1.50 to –4.0 (acyclic dinucleoside phosphates, **NpN**, yellow), from –7.0 to –8.5 (*s*, 5',3' 6-membered ring cyclic phosphate **p<N,** blue), from –9.0 to –10.0 (*s*, $\mathbf{PP_i}$); –9.5- –10.0 and –12- –13.5 (n x *d*, organic diphosphates **Npp,** pink); from –25 to –26 (*t*, organic triphosphates **Nppp,** very low quantity).

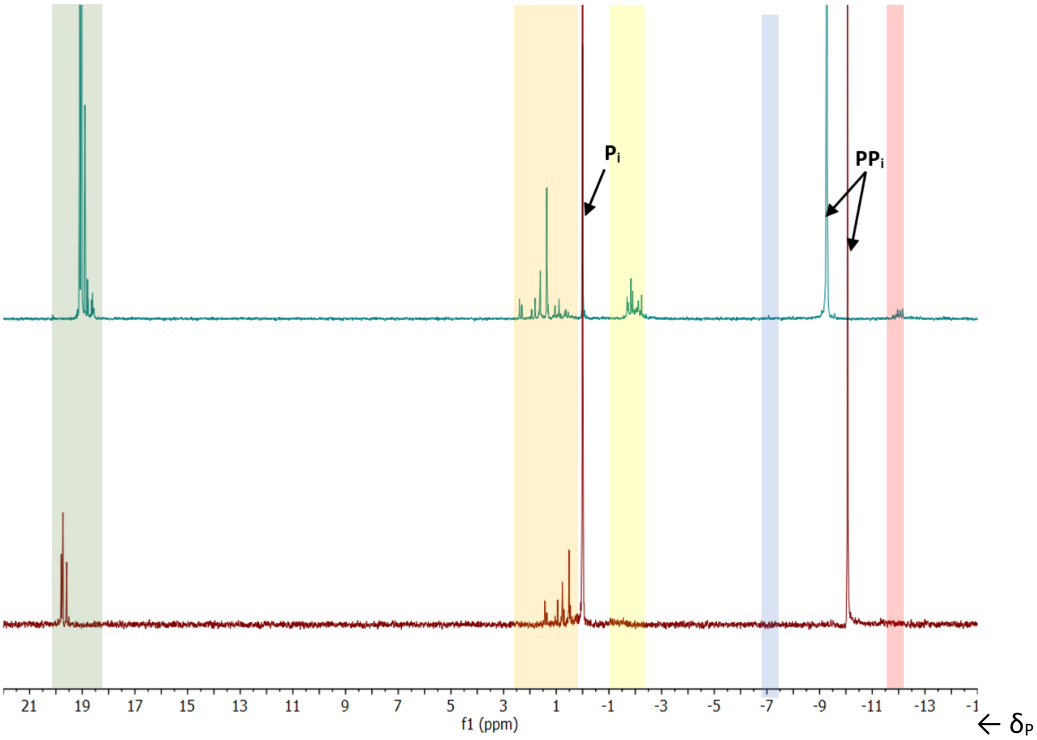

Supplementary Fig. 133. $^{31}P\{^{1}H\}$ NMR spectra (202.5 MHz, H_2O/D_2O = 9:1) 0.5 mmol scale reaction after 120 h of heating **11** at 115 °C with 0.5 eq **3a** as liquidiser of crude mixtures: **lower spectrum** – **11**:**2a**:$\mathbf{P_i}$ (1:1:1); **upper spectrum** – **11**:**2a**:$\mathbf{P_i}$ (1:4:1). δ_P (ppm) = 20.0-18.5 (*s*, 2',3' 5-membered ring cyclic phosphate **A>p,** green), 2.5-0.5 (5', 3' or 2' acyclic adenosine-phosphates, **Ap**, ochre), 0.00 (*s*, $\mathbf{P_i}$), from –1.50 to –2.5 (acyclic diadenosine phosphates, **ApA**, yellow), –7.0 (*s*, 5',3' 6-membered ring cyclic phosphate **p<A,** blue), –9.0 and –10.0 (*s*, $\mathbf{PP_i}$); –12.0 (2 x *d*, organic adenosine diphosphate **App,** pink).

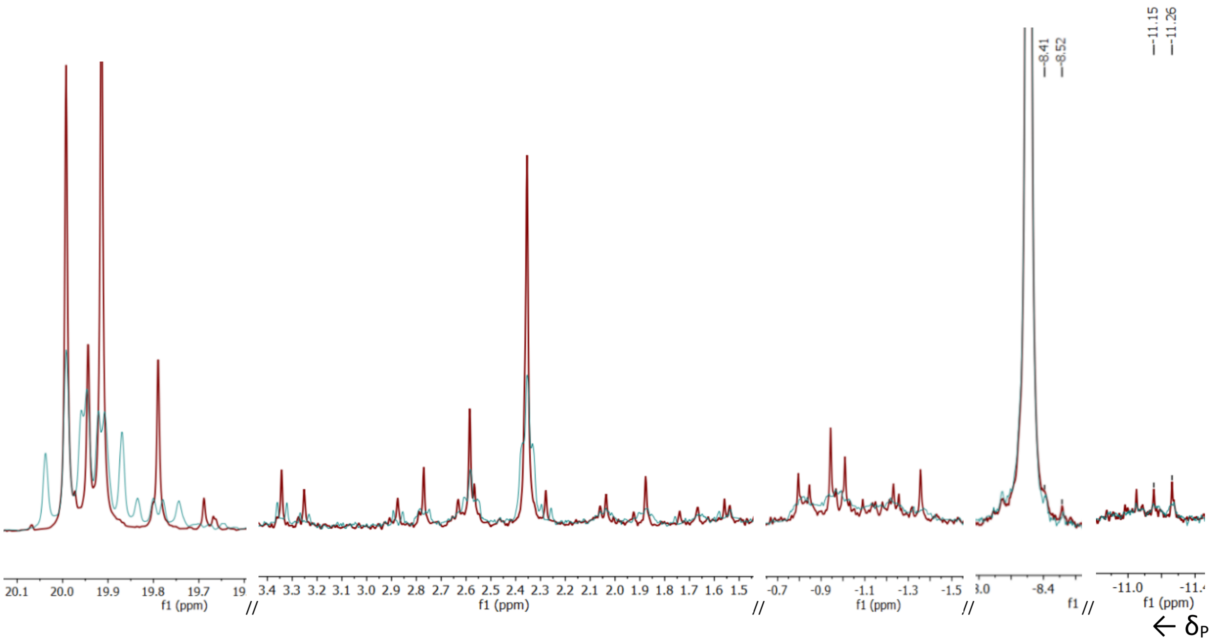

Supplementary Fig. 134. Selected $^{31}P\{^1H\}$ NMR signals (**brown**, Supplementary **Fig. 133**) and ^{31}P NMR signals (**turquoise**) (202.5 MHz, H_2O/D_2O = 9:1) of organic products of 0.5 mmol scale reaction after 120 h of heating at 115 °C of crude mixture **11**:**2a**:**P**$_i$ (1:4:1) with 0.5 eq **3a** as liquidiser.

The ^1H-coupled ^{31}P NMR multiplicity patterns (turquoise) of phosphorylated nucleosides correspond to those previous of **5** (Supplementary **Fig. 19**, Supplementary **Fig. 20**) and **6** (Supplementary **Fig. 31**). However, because of the presence of higher number of similar products, the interpretation of each of the different phosphorylated compound is complicated. δ_P (ppm) = 20.1-19.7 (s/*m*, 2',3' 5-membered ring cyclic phosphates **A>p**); 3.4-1.5 (acyclic organo-phosphates **Ap**, including s/*t* 5' and s/*d* 2' or 3' monoesters); from –0.7 to –1.5 (acyclic diester organo-phosphates **ApA**, due to many signals placed close to each other it is difficult to observe a certain multiplicity of each peak); –0.7 (not shown on the figure, 5',3' 6-membered ring cyclic phosphates **p<A**, is too small to identify multiplicity); –8.46 and –11.21 (*d*/*d*, J = 21.9 Hz and *d*/*dd*, J = 22.0 Hz, adenosine diphosphate **App**); –8.1 and –11.05 (not identified).

Up to a 4-fold molar excess of urea over neat equimolar {nucleoside + **P**$_i$} mixtures certainly helped to bring the phosphorylation level of, in particular, the purine nucleosides to that of the pyrimidine nucleosides, as is shown in the following spectra and the summary in Supplementary **Fig. 144** (for pristine nucleosides) and Supplementary **Fig. 153** (for an equimolar mixture of all four canonical ribonucleosides).

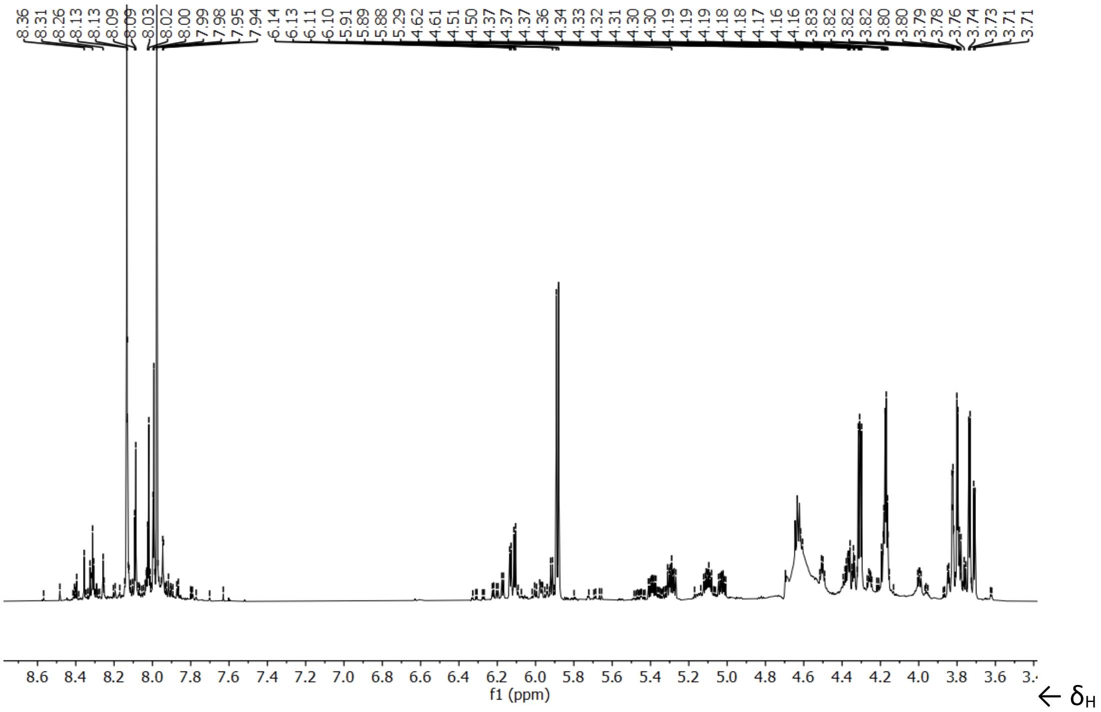

Supplementary Fig. 135. ^{1}H NMR spectrum (500 MHz, H_2O signal suppressed) of crude mixtures of reaction **11**:**2a**:**P**$_i$ (1:4:1) with 0.5 eq **3a** as liquidiser, 0.5 mmol scale reaction after 120 h of heating at 115 °C, then dissolved in H_2O/D_2O = 9:1. δ_H (ppm) = 8.13 (*s*, 1H), 8.03-7.98 (*m*, 1H), 7.98 (*s*, 1H), 6.12 (*dd*, J = 11.4, 4.0 Hz, 1H), 5.89 (*d*, J = 6.0 Hz, 1H), 4.31 (*dd*, J = 5.2, 3.4 Hz, 1H), 4.17 (*q*, J = 3.3 Hz, 1H), 3.81 (*dd,* J = 12.8, 2.7 Hz, 1H), 3.72 (*dd*, J = 12.9, 3.6 Hz, 1H). See Supplementary **Fig. 115** for nucleoside peak assignments and Supplementary **Fig. 136** for ^{1}H-^{31}P cross-correlations.

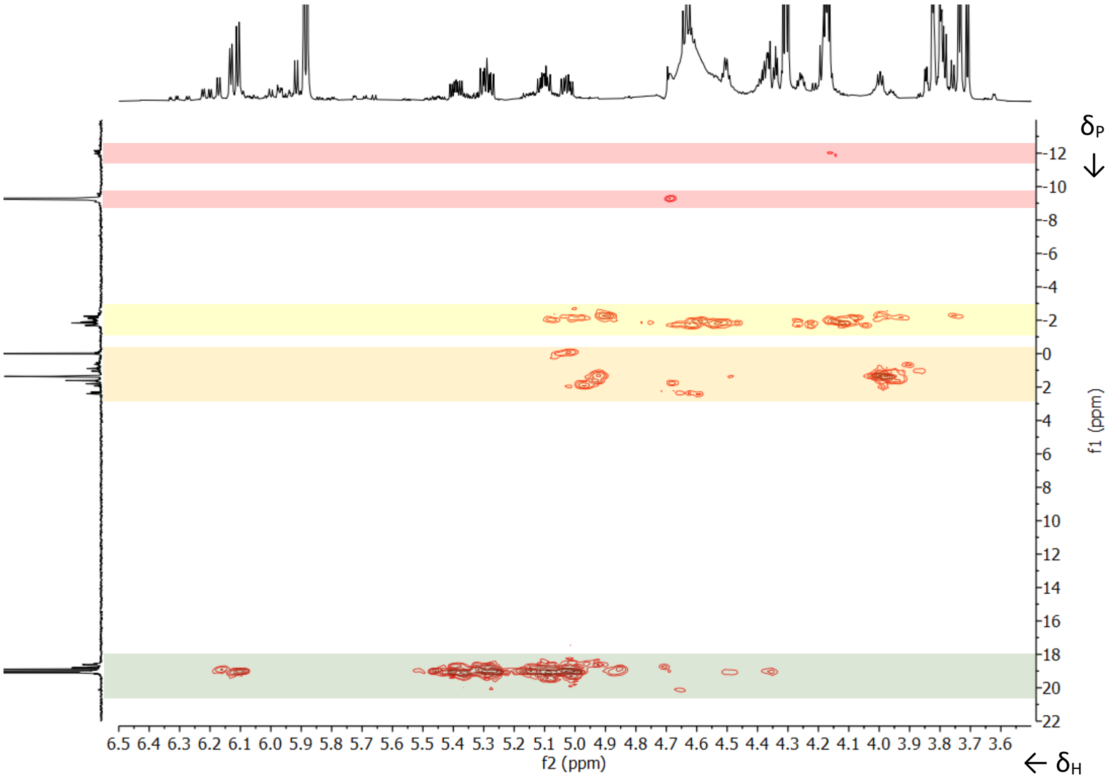

Supplementary Fig. 136. ^{1}H-^{31}P HMBC spectrum (500 MHz for ^{1}H [horizontal axis], 202.5 MHz for ^{31}P{^{1}H} [vertical axis]) of crude mixtures of reactions **11**:**2a**:**P**$_i$ (1:4:1) with 0.5 eq of **3a** as liquidiser, 0.5 mmol scale reaction after 120 h of heating at 115 °C, then dissolved in H_2O/D_2O = 9:1. Blue (**A>p**), ochre (**Ap**), yellow (**ApA**) and pink (**App**) zones highlight organic products of phosphorylation.

A

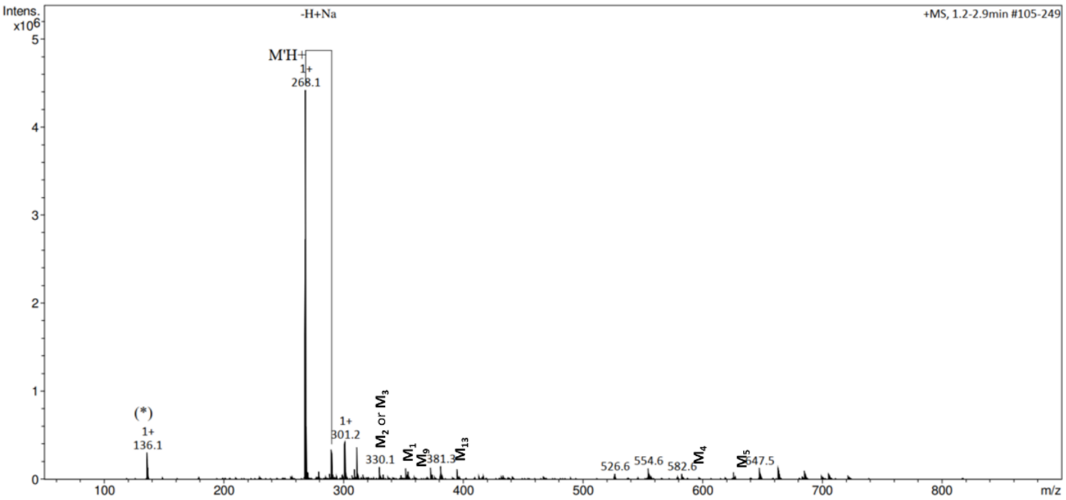

B

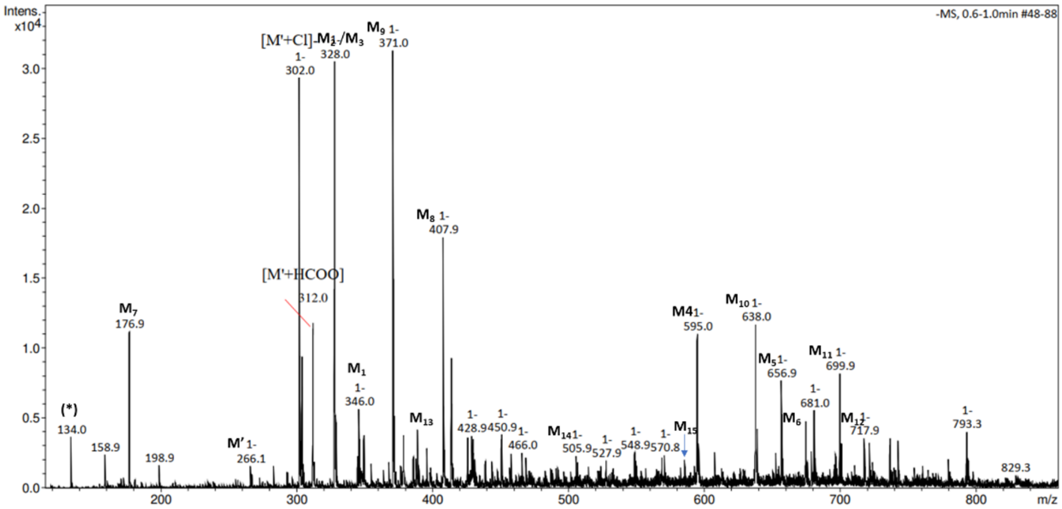

C

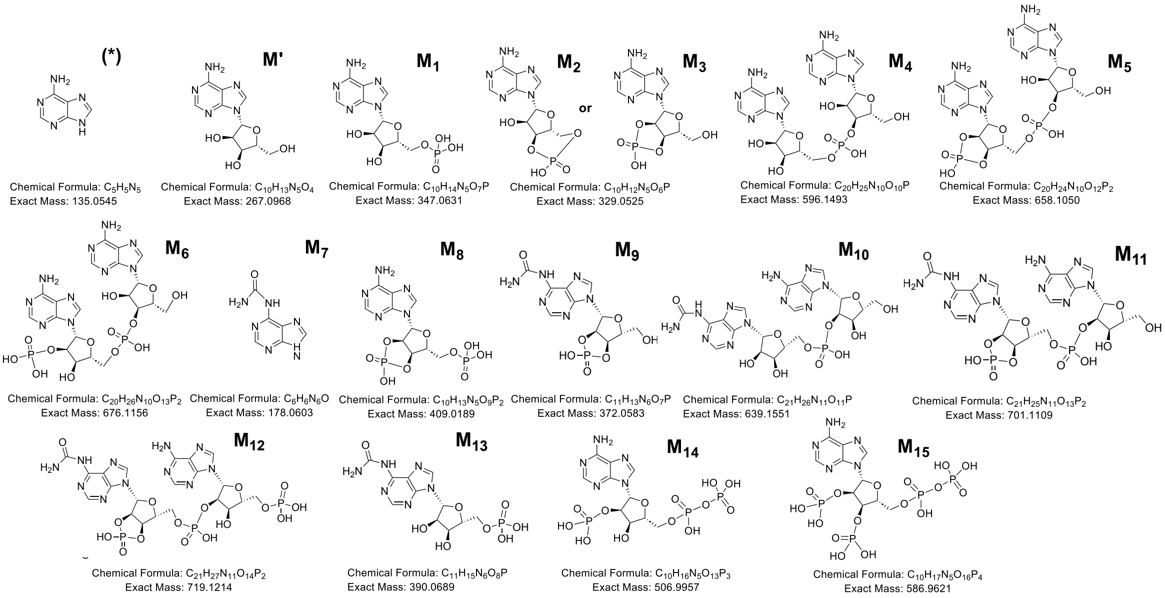

Supplementary Fig. 137. LRMS (direct injection) of the water extract of the crude mixture obtained by reacting **11**, **2a** and **P$_i$** (1:4:1) with 0.5 eq **3a** as liquidiser, 0.5 mmol scale; reaction time 120 h under 115 °C.

A – Total ion spectrum in positive-ion mode;

B – Total ion spectrum in negative-ion mode;

C – Chemical structures (only one isomer shown), formulas and exact (monoisotopic) masses of the detected molecules in their uncharged state.

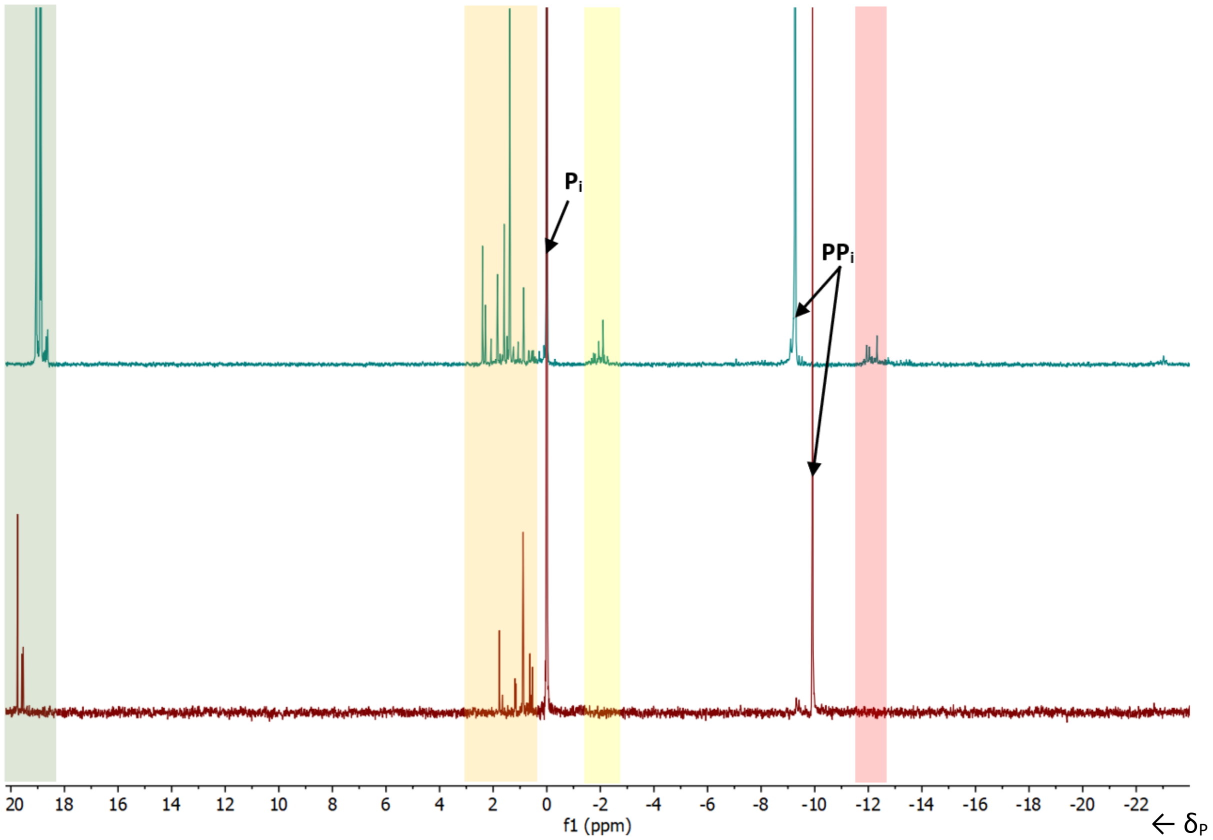

Supplementary Fig. 138. $^{31}P\{^{1}H\}$ NMR spectra (202.5 MHz, H_2O/D_2O = 9:1) 0.5 mmol scale reaction after 120 h of heating at 115 °C with 0.5 eq **3a** as liquidiser of crude mixtures: **lower spectrum** – **12**:**2a**:**P**$_i$ (1:1:1); **upper spectrum** – **12**:**2a**:**P**$_i$ (1:4:1). δ_P (ppm) = 20.0-18.5 (*s*, 2',3' 5-membered ring cyclic phosphate **G>p,** green), 2.5-0.5 (5', 3' or 2' acyclic phosphates, **Gp**, ochre), 0.00 (*s*, **P**$_i$), from –1.50 to –2.5 (acyclic diguanosine phosphates, **GpG**, yellow), –10.0 (*s*, **PP**$_i$). Signals that were identified only in experiment with 4 eq of **2a**: δ_P (ppm) = –9.47 and –11.99 (2 x *d*, J = 21.2 Hz, organic diphosphate **Gpp,** pink); –9.15, –12.68 and – 23.02 (2 x *d* and *t*, J = 21.8 Hz, organic triphosphate **Gppp**).

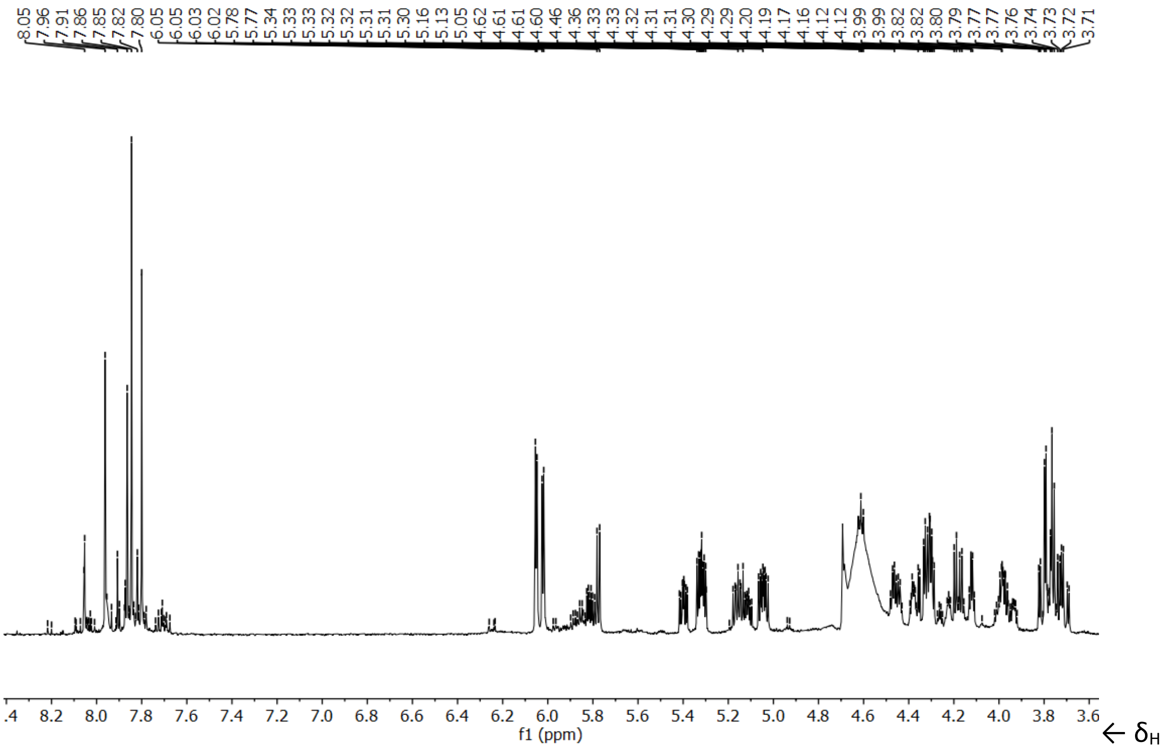

Supplementary Fig. 139. ^1H NMR spectrum (500 MHz, H_2O signal suppressed) of crude mixtures of reaction **12**:**2a**:**P$_i$** (1:4:1) with 0.5 eq **3a** as liquidiser, 0.5 mmol scale reaction after 120 h of heating at 115 °C, then dissolved in H_2O/D_2O = 9:1. δ_H (ppm) = 7.96 (*s*, OH), 7.86 (*s*, 1H), 7.85 (*s*, 1H), 7.80 (*s*, 1H), 6.04 (*dd*, J = 14.9, 3.5 Hz, 2H), 5.83-5.79 (*m*, 1H), 5.78 (*d*, J = 5.9 Hz, 1H), 5.40 (*ddd*, J = 7.8, 6.7, 3.0 Hz, 1H), 5.35-5.29 (*m*, 2H), 5.18- 5.12 (*m*, 1H), 5.04 (*ddd*, J = 9.7, 6.8, 4.7 Hz, 1H), 4.61 (*t*, J = 4.5 Hz, OH), 4.46 (*dq*, J = 12.2, 4.3 Hz, 1H), 4.34-4.28 (*m*, 2H), 4.18 (*dd*, J = 12.1, 5.1 Hz, 1H), 4.12 (*q*, J = 3.7 Hz, 1H), 3.98 (*ddd*, J = 8.2, 5.1, 4.1 Hz, 1H), 3.81-3.75 (*m*, 2H), 3.73 (*dd*, J = 8.0, 4.5 Hz, 1H). See Supplementary **Fig. 119** for nucleoside peak assignments and Supplementary **Fig. 140** for ^1H-^{31}P cross-correlations.

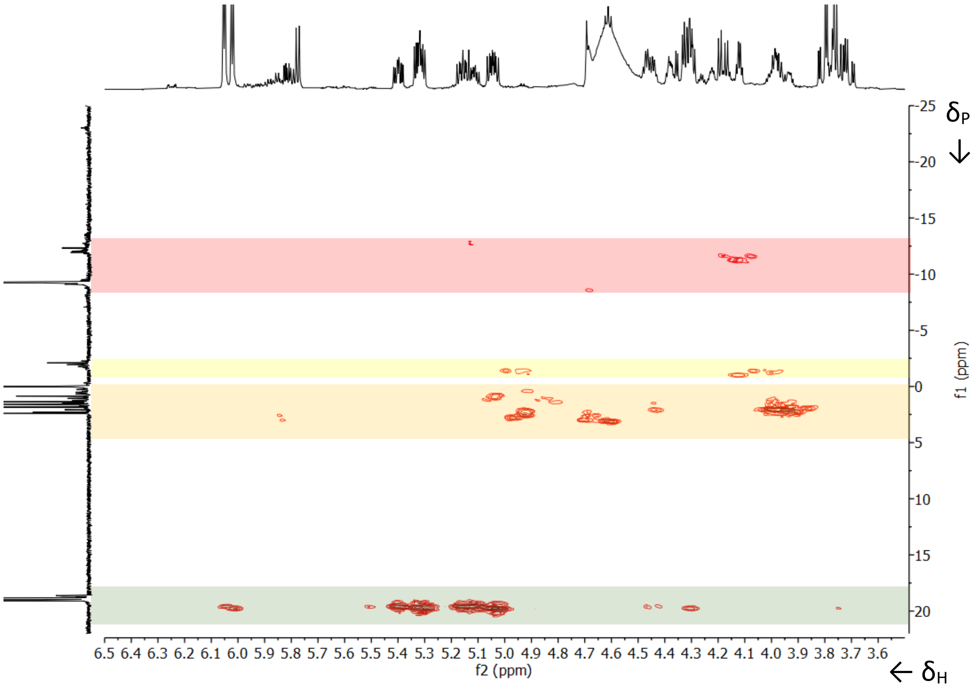

Supplementary Fig. 140. ^1H-^{31}P HMBC spectrum (500 MHz for ^1H [horizontal axis], 202.5 MHz for $^{31}P\{^1H\}$ [vertical axis]) of crude mixtures of reaction **12**:**2a**:**P$_i$** (1:4:1) with 0.5 eq **3a** as liquidiser, 0.5 mmol scale reaction after 120 h of heating at 115 °C, then dissolved in H_2O/D_2O = 9:1. Blue (**G>p**), ochre (**Gp**), yellow (**GpG**) and pink (**Gpp**) zones highlight organic products of phosphorylation.

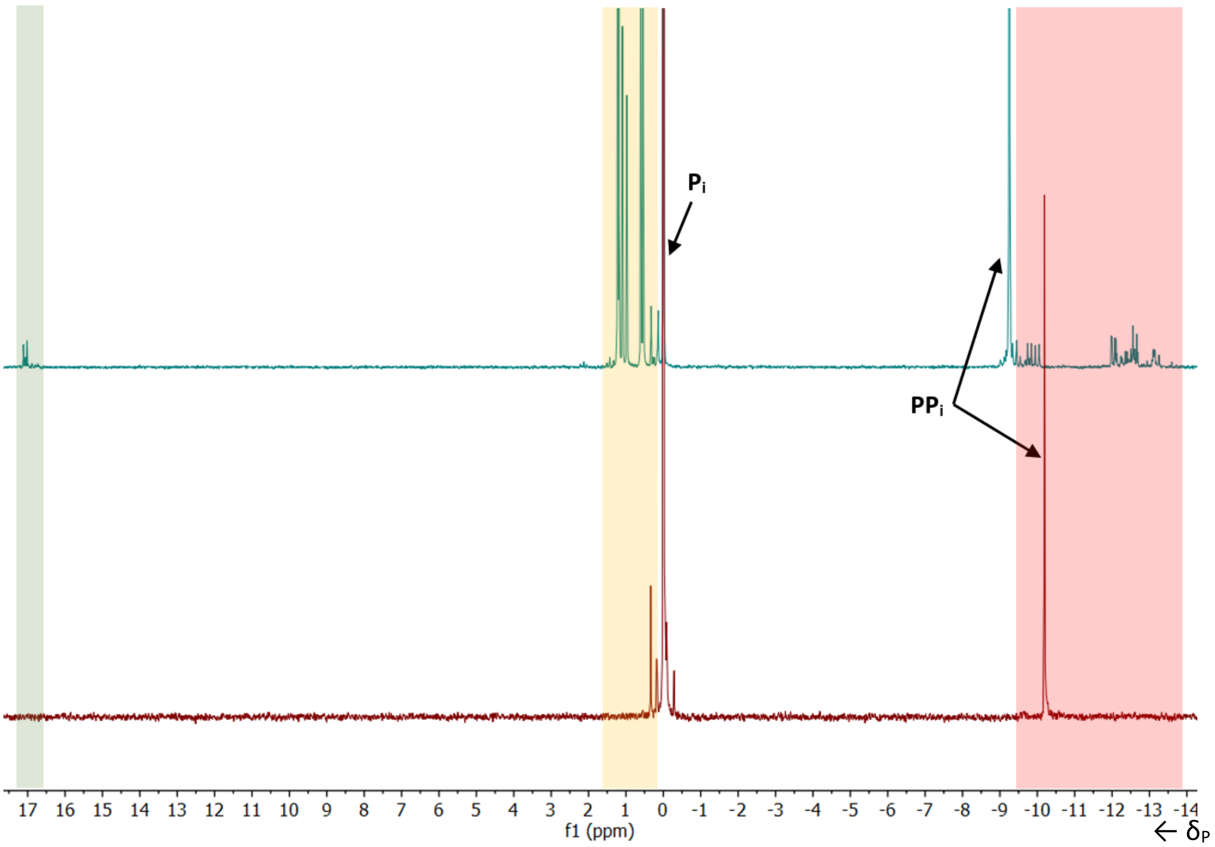

Supplementary Fig. 141. $^{31}P\{^{1}H\}$ NMR spectra (202.5 MHz, H_2O/D_2O = 9:1) 0.5 mmol scale reaction after 120 h of heating at 115 °C with 0.5 eq **3a** as liquidiser of crude mixtures: **lower spectrum** – **15**:**2a**:**P**$_i$ (1:1:1); **upper spectrum** – **15**:**2a**:**P**$_i$ (1:4:1). δ_P (ppm) = 17.5-17.0 (*s*, 5-membered ring 3,4- and 4,5-cyclic phosphates of 2-deoxyribose: **drib5>p**, green), 1.5-0.2 (5', 3' or 2' acyclic phosphates, **Tp**, ochre), 0.00 (*s*, **P**$_i$), –8 and –10.0 (*s*, **PP**$_i$). Signals that were identified only in the experiment with 4 eq of **2a :** organic diphosphates (**Tpp,** pink): δ_P (ppm) = –9.07 and –12.05 (2 x *d*, J = 21.1 Hz, **Tpp** and **Tpp**), –9.73 and –12.03 (2 x *d*, J = 21.3 Hz **Tpp** and **Tpp**), –10.00 and –12.61 (2 x *d*, J = 21.0 Hz **Tpp** and **Tpp**); other signals were not identified.

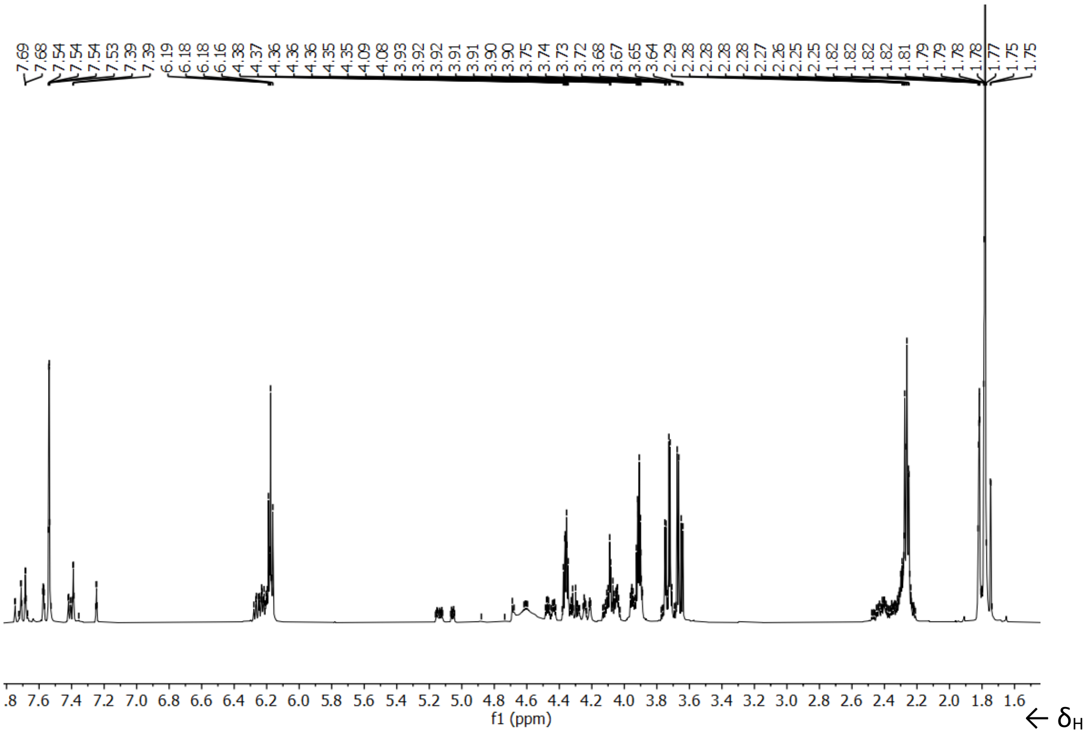

Supplementary Fig. 142. ^{1}H NMR spectrum (500 MHz, H_2O signal suppressed) of crude mixtures of reaction **15**:**2a**:**P**$_i$ (1:4:1) with 0.5 eq **3a** as liquidiser, 0.5 mmol scale reaction after 120 h of heating at 115 °C, then dissolved in H_2O/D_2O = 9:1. δ_H (ppm) = 7.54 (*q*, J = 1.2 Hz, 1H), 6.18 (*t*, J = 6.7 Hz, 1H), 4.36 (*td*, J = 5.4, 4.0 Hz, 1H), 3.93-3.89 (*m*, 1H), 3.73 (*dd*, J = 12.5, 3.6 Hz, 1H), 3.66 (*dd*, J = 12.5, 5.0 Hz, 1H), 2.29-2.24 (*m*, 2H). See Supplementary **Fig. 127** for 2'-deoxynucleoside peak assignments and Supplementary **Fig. 143** for ^{1}H-^{31}P cross-correlations.

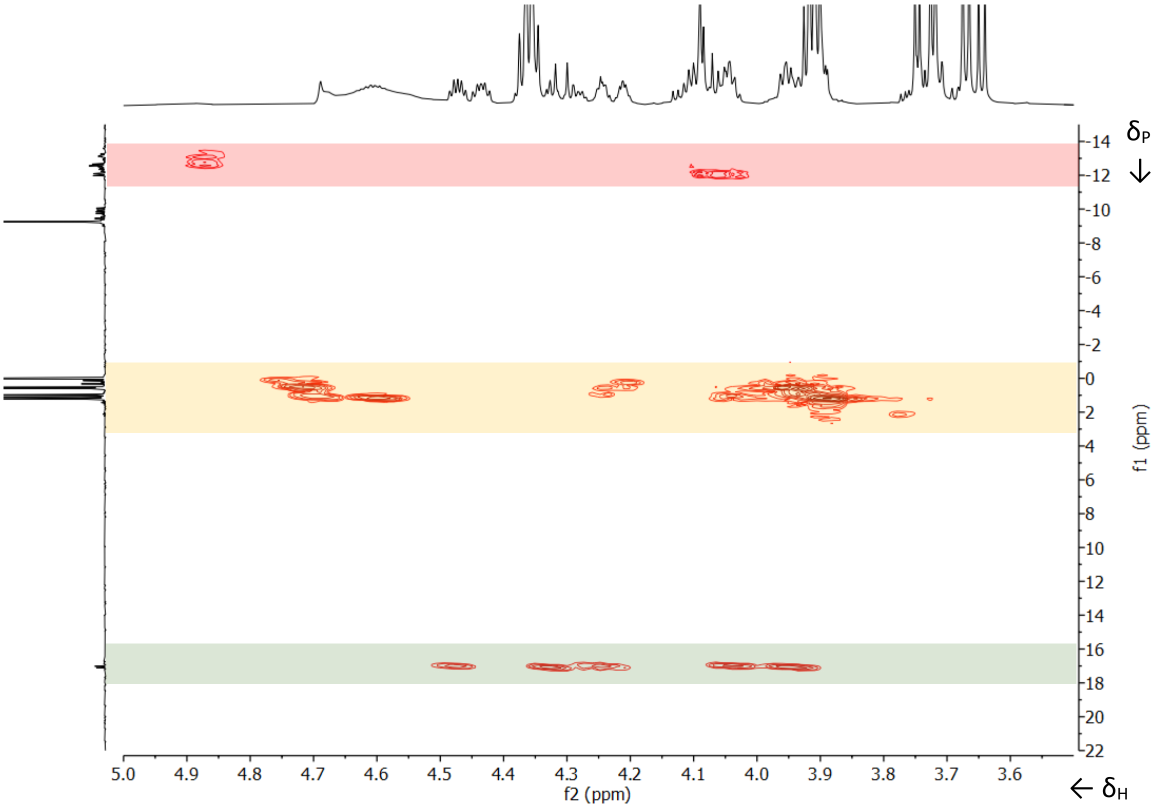

Supplementary Fig. 143. ^{1}H-^{31}P HMBC spectrum (500 MHz for ^{1}H [horizontal axis], 202.5 MHz for $^{31}P\{^{1}H\}$ [vertical axis]) of crude mixtures of reactions **15**:**2a**:**P**$_i$ (1:4:1) with 0.5 eq of **3a** as liquidiser, 0.5 mmol scale reaction after 120 h of heating at 115 °C, then dissolved in H_2O/D_2O = 9:1. Blue (**drib>p** = 2-deoxyribosyl-3,4- and -4,5-cyclic phosphates), ochre (**Tp**) and pink (**Tpp**) zones highlight organic products of phosphorylation.

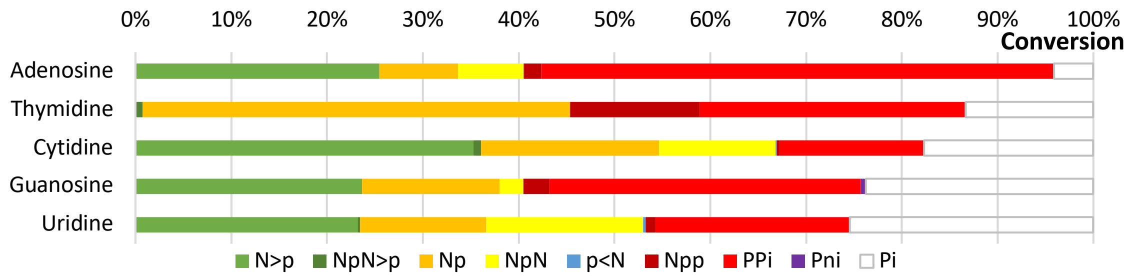

Supplementary Fig. 144. Summary of urea-assisted phosphorylation of nucleosides **N** (**11-15**) in **N**:**2a**:**P$_i$** (1:4:1) with 0.5 eq **3a** as liquidiser, 0.5 mmol scale reaction after 120 h of heating at 115 °C, then dissolved in H_2O/D_2O = 9:1. Percent values from signal integration of quantitative $^{31}P\{^1H\}$ NMR spectra (Supplementary **Fig. 132**-Supplementary **Fig. 143**). "N>p" for thymidine : **drib>p** = 2-deoxyribosyl-3,4- and -4,5-cyclic phosphates. N>p, p<N : 2',3'-, and resp., 5',3'-cyclic nucleos(t)ide phosphates.

Supplementary Table 47. Data to Supplementary **Fig. 144**. Total conversion of initial amount of **P$_i$** to phosphorylated organic products was calculated by subtracting inorganic compound integrals (**P$_i$**, **PP$_i$** and **P$_{ni}$**) from the sum of all integrated $^{31}P\{^1H\}$ NMR peak areas (in H_2O/D_2O = 9:1).

Nucleoside	N>p, %	NpN>p, %	Np, %	NpN, %	p<N, %	Npp, %	PP$_i$, %	P$_{ni}$, %	P$_i$, %
**11**	25.45	-	8.21	6.81	0.11	1.80	53.48	-	4.14
**12**	23.68	-	14.36	2.43	-	2.77	32.46	0.52	23.77
**13**	35.26	0.81	18.61	12.15	0.14	0.25	15.09	-	17.67
**14**	21.07	0.25	11.92	14.86	0.26	0.97	18.41	-	23.07
**15**	-	0.73	44.62	-	-	13.52	27.65	0.12	13.36

7.5.5. Experiments with cTMP as a phosphorous source

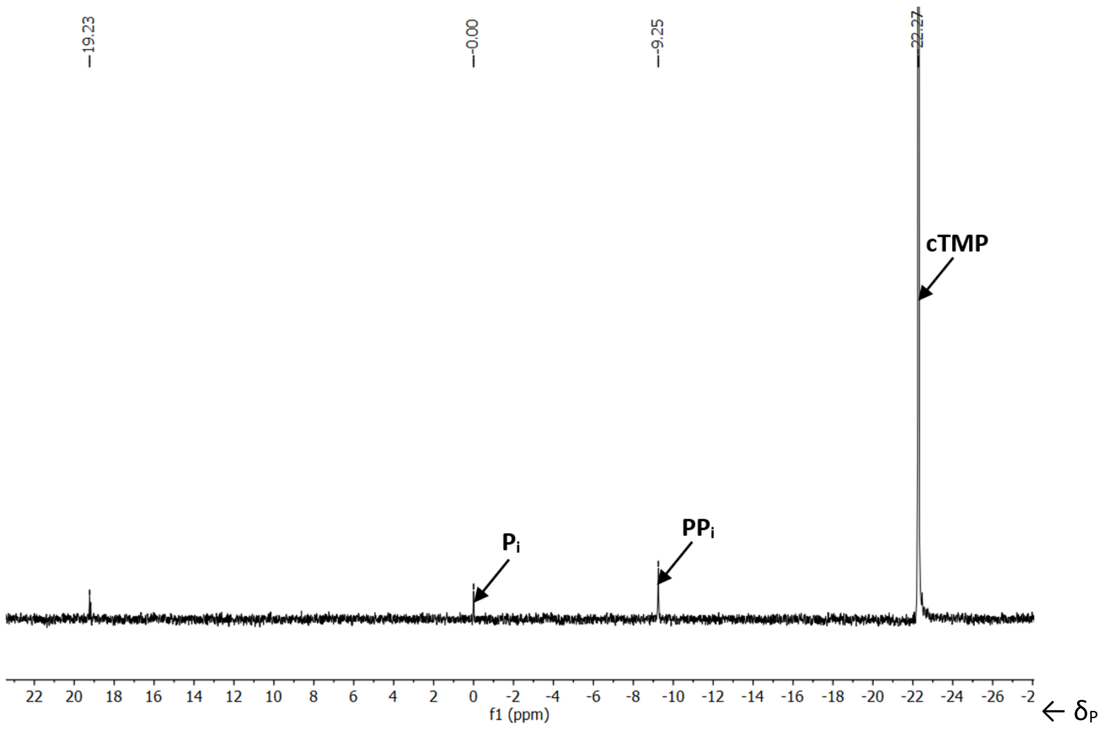

Supplementary Fig. 145. Exemplary $^{31}P\{^1H\}$ NMR spectrum (202.5 MHz) of a crude mixture of reaction **11**:**2a**:**cTMP** (1:1:1) with 0.5 eq **3a** as liquidiser, 0.5 mmol scale reaction after 120 h of heating at 115 °C, then dissolved in H_2O/D_2O = 9:1. δ_P (ppm) = 19.23 (*s*, 2',3' 5-membered ring cyclic phosphate **A>p,** green), 0.00 (*s*, **P$_i$**), –9.25 (*s*, **PP$_i$**), –22.27 (*s*, **cTMP**).

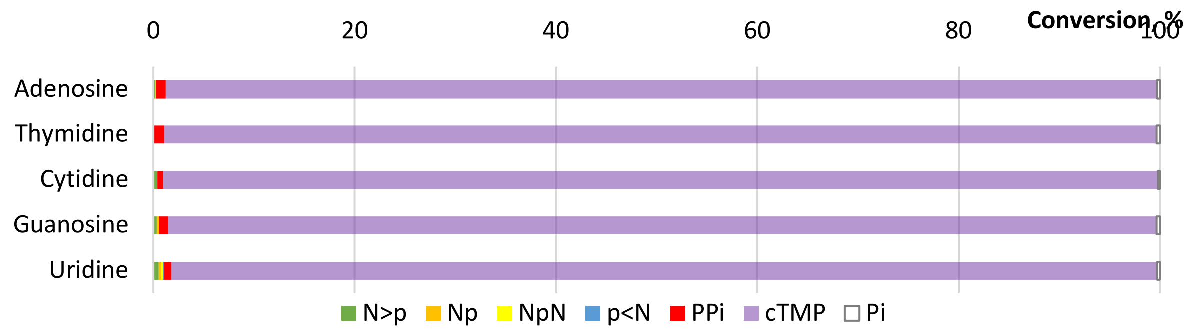

Supplementary Fig. 146. Summary of urea-assisted phosphorylation of nucleosides **N** (**11**-**15**) in **N**:**2a**:**cTMP** (1:1:1) with 0.5 eq **3a** as liquidiser, 0.5 mmol scale reaction after 120 h of heating at 115 °C, then dissolved in H_2O/D_2O = 9:1. Percent values from signal integration of quantitative $^{31}P\{^1H\}$ NMR spectra.

Supplementary Table 48. Data to Supplementary **Fig. 146**. Total conversion of initial amount of $\mathbf{P_i}$ to phosphorylated organic products was calculated by subtracting inorganic compound integrals ($\mathbf{P_i}$, $\mathbf{PP_i}$ and **cTMP**) from the sum of all integrated $^{31}P\{^1H\}$ NMR peak areas (in H_2O/D_2O = 9:1). **N>p**, **p<N**: 2',3'-, and resp., 5',3'-cyclic nucleoside phosphates.

Nucleoside	N>p, %	Np, %	NpN, %	p<N, %	PP_i, %	P_{ni}, %	P_i, %
**11**	0.19	0.07	-	-	0.99	98.47	0.28
**12**	0.34	0.23	-	-	0.94	98.17	0.32
**13**	0.42	-	-	-	0.58	98.80	0.20
**14**	0.51	0.28	0.19	0.04	0.80	97.91	0.27
**15**	-	0.11	-	-	0.97	98.58	0.34

7.5.6. Urea-assisted phosphorylation of equimolar 'dry' mixtures of mononucleotides without and with complementary nucleosides, and of all ribonucleosides

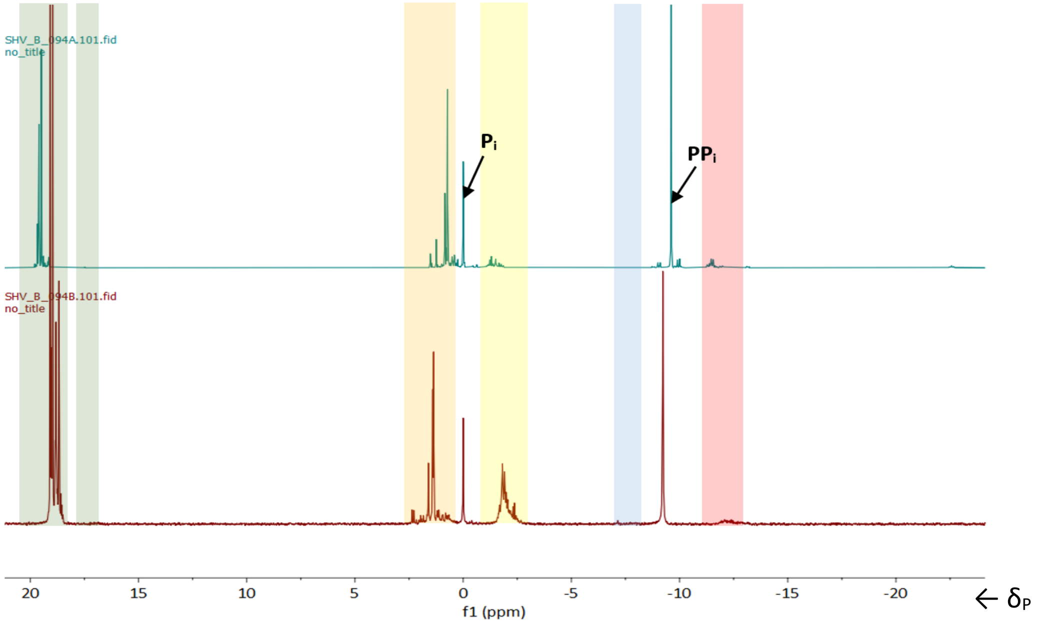

Supplementary Fig. 147. ±AMP+U: $^{31}P\{^1H\}$ NMR spectra (202.5 MHz, H_2O/D_2O = 9:1) 0.5 mmol scale reaction after 120 h of heating at 115 °C with 0.5 eq **3a** as liquidiser of crude mixtures: **lower spectrum** – **11a:14**:**2a**:$\mathbf{P_i}$ (0.5:0.5:4:1); **upper spectrum** – **11a**:**2a**:$\mathbf{P_i}$ (1:4:1). δ_P (ppm) = 20.0-18.2 (*s*, 2',3' 5-membered ring cyclic phosphates **N>p**, green), 17.6-17.2 (*s*, 2',3'-5-membered ring cyclic diphosphate **pN>p**, dark green, were was identified by analogy with acyclic dinucleotides), 2.5-0.2 (5'-, 3'- or 2'-acyclic phosphates, **Tp**, ochre), 0.00 (*s*, $\mathbf{P_i}$), from –0.3 to –3.0 (acyclic dinucleoside phosphates, **NpN**, yellow), from –6.5 to –7.5 (*s*, 5',3' 6-membered ring cyclic phosphate **p<N,** blue), –9.5 (*s*, $\mathbf{PP_i}$); –11.0 to –13.5 (n x *d*, organic diphosphates **Npp,** red).

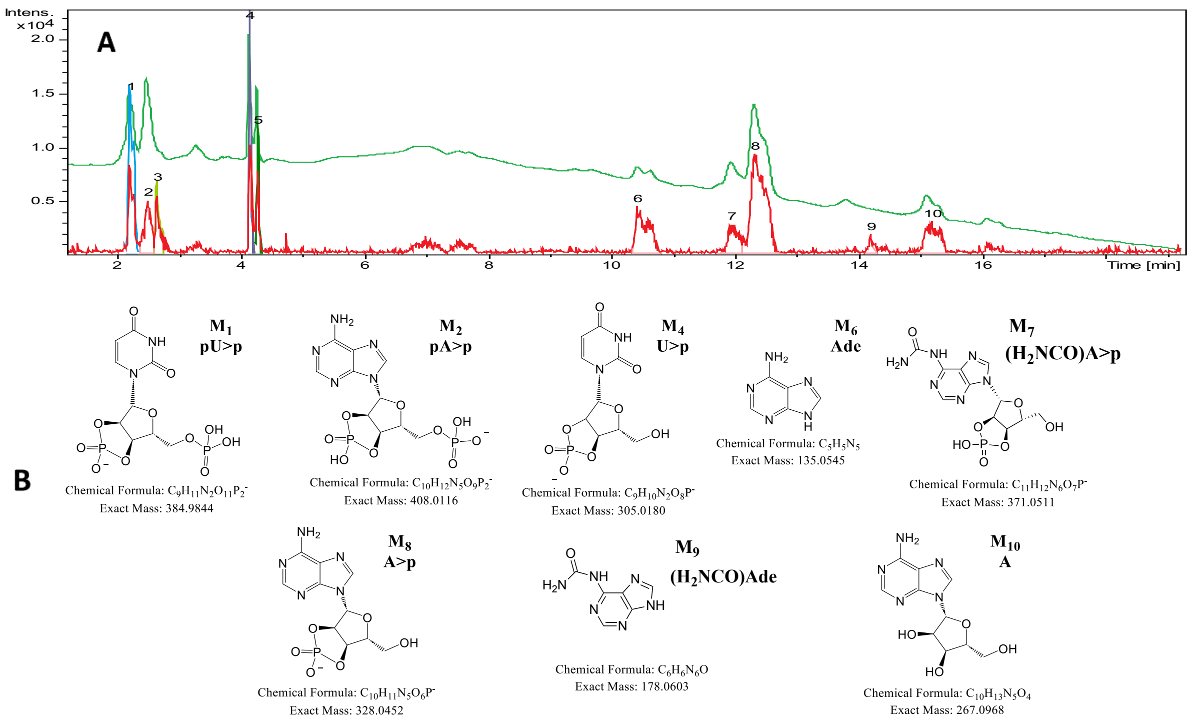

Supplementary Fig. 148. ±AMP+U: Chromatogram of H_2O extract of a 1 mmol scale mixture of **11a**:**14**:**2a**:**P$_i$** (0.5:0.5:4:1 molar ratios) with 1 eq **3a** as liquidiser, heated neat at 115 °C for 120 h. **A** – RPHPLC-HRMS in negative-ion mode; **green**: UV chromatogram (260 nm), R_T = 0-18.5 min, **red**: total-ion chromatogram, **coloured**: ion-extracted signals. **B** – Chemical structures (only the most probable isomer shown), formulas and exact (mono-isotopic) masses of the detected compounds. **M_3** signal with corresponding *m/z* 141.0172 was not identified; **M_4** and **M_5** have two similar patterns *m/z* 305.0186 of U>p and *m/z* 611.0446 of 2 x *m* of U>p or possibly UpU>p. Each signal can belong to different molecules or possibly be a 5- or 6-membered ring cyclic phosphate.

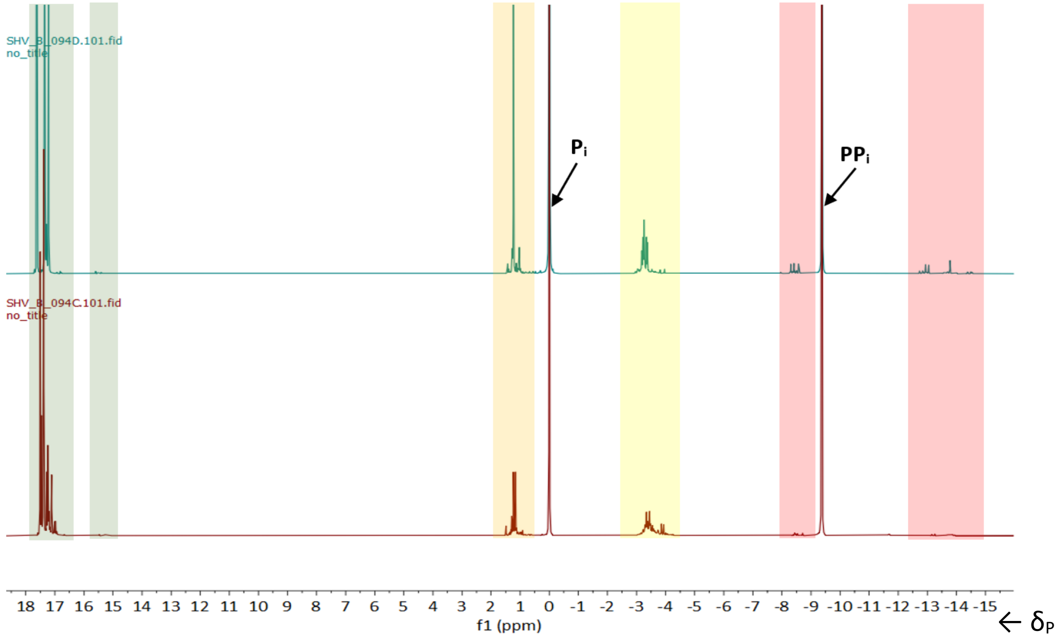

Supplementary Fig. 149. ±UMP+A: $^{31}P\{^1H\}$ NMR spectra (202.5 MHz, H_2O/D_2O = 9:1) 0.5 mmol scale reaction after 120 h of heating at 115 °C with 0.5 eq **3a** as liquidiser of crude mixtures: **lower spectrum** – **14a**:**11**:**2a**:**P$_i$** (0.5:0.5:4:1); **upper spectrum** – **14a**:**2a**:**P$_i$** (1:4:1). δ_P (ppm) = 17.8-16.5 (*s*, 2',3'-5-membered ring cyclic phosphates **N>p**, green), 15.7-15.1 (*s*, 2',3'-5-membered ring cyclic diphosphate **pN>p**, dark green, were was identified by analogy with acyclic dinucleotides), 1.6-0.2 (5'-, 3'- or 2'-acyclic phosphates, **Tp**, ochre), 0.00 (*s*, **P$_i$**), from –2.8 to –4.5 (acyclic dinucleoside phosphates, **NpN**, yellow), –9.4 (*s*, **PP$_i$**); –7.8 to –8.8 and –12.5 to –14.7 (n x *d*, organic diphosphates **Npp,** pink).

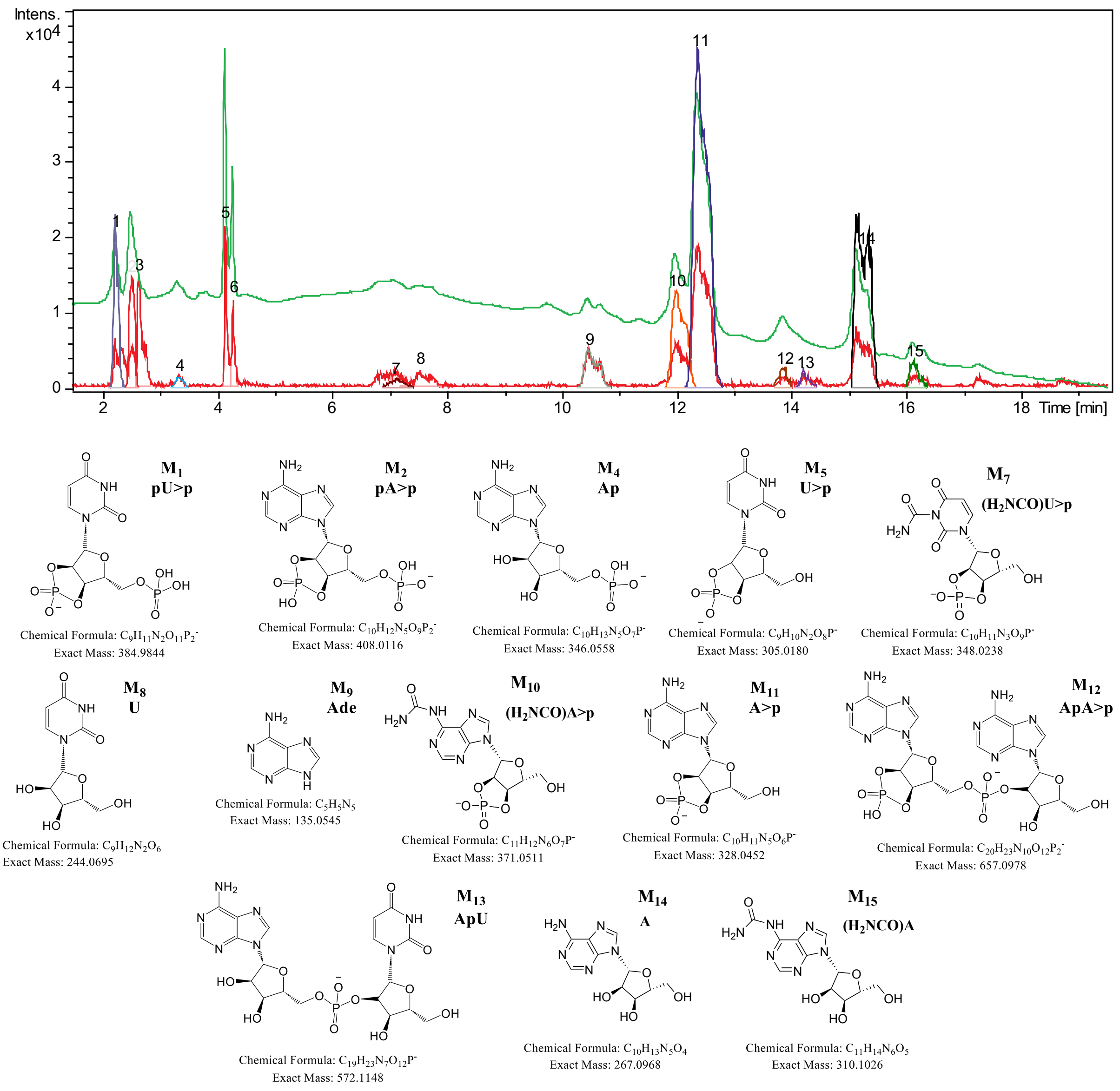

Supplementary Fig. 150 ±UMP+A: Chromatogram of H_2O extract of a 1 mmol scale mixture of **14a**:**11**:**2a:P$_i$** (0.5:0.5:4:1 molar ratios) with 1 eq **3a** as liquidiser, heated neat at 115 °C for 120 h. **A** – RPHPLC-HRMS in negative-ion mode; **green**: UV chromatogram (260 nm), R_T = 0-18.5 min, **red**: total-ion chromatogram, **coloured**: ion-extracted signals. **B** – Chemical structures (only the most likely isomer shown) and exact masses of the detected compounds. **M$_3$** signal with corresponding *m/z* 141.0168 was not identified; **M$_5$** and **M$_6$** have two similar patterns *m/z* 305.0186 of U>p and *m/z* 611.0446 of 2 x *m* of U>p or possibly UpU>p. Each signal can comprise different molecules or possibly be a 5- or 6-membered ring cyclic phosphate.

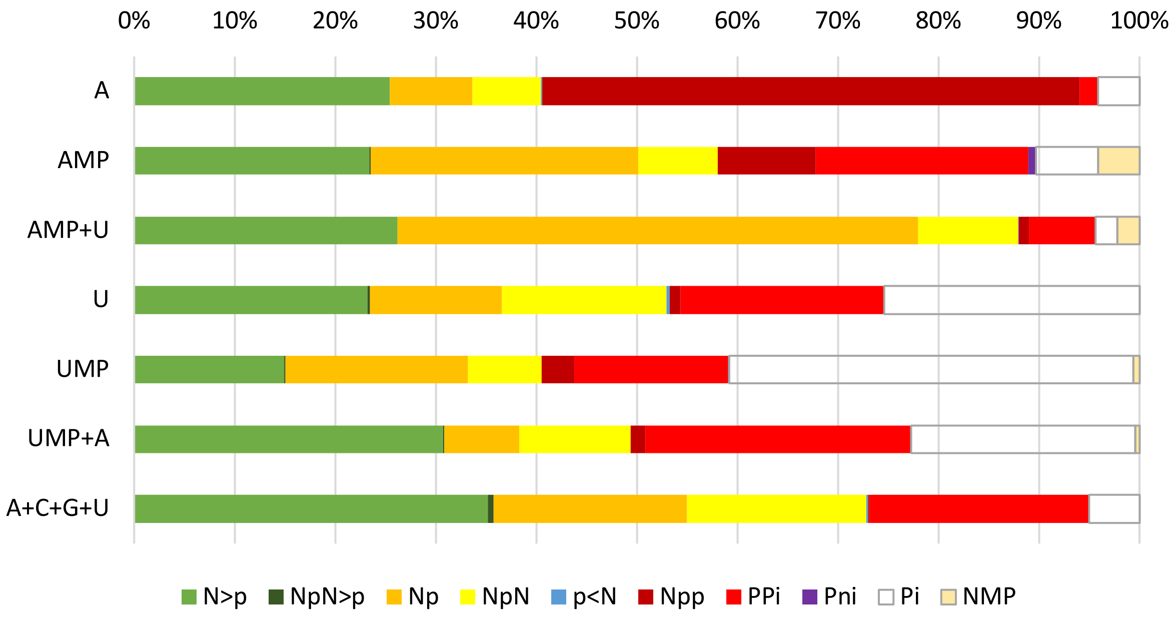

Supplementary Fig. 151. Comparison of the 'dry' phosphorylation of nucleosides (**N**), nucleoside-5'-monophosphates (**NMP**) blended as **NMP**:**N**:**2a**:P_i (1:1:4:1) or **11**:**12**:**13**:**14**:**2a**:P_i (0.25:0.25:0.25:0.25:4:1), all with 1 eq **3a** as liquidiser. 1 mmol scale reaction after 120 h of heating at 115 °C, then dissolved in H_2O/D_2O = 9:1. Percent values from signal integration of quantitative $^{31}P\{^1H\}$ NMR spectra (Supplementary **Fig. 147**-Supplementary **Fig. 152**). N>p, p<N: 2',3'-, and resp., 5',3'-cyclic nucleoside phosphates.

Supplementary Table 49. Data to Supplementary **Fig. 151**. Total conversion of initial amount of P_i or/and nucleoside-5'-monophosphates (**NMP**) to phosphorylated organic products was calculated by subtracting inorganic compound integrals (P_i, PP_i, P_{ni} and **NMP**) from the sum of all integrated $^{31}P\{^1H\}$ NMR peak areas (in H_2O/D_2O = 9:1). **N>p**, **p<N**: 2',3'-, and resp., 5',3'-cyclic nucleos(t)ide phosphates.

Start. molecule	N>p %	NpN>p %	Np %	NpN %	p<N %	Npp, %	PP_i, %	Pn_i, %	P_i, %	NMP, %
**11**	25.45	-	8.21	6.81	0.11	1.80	53.48	-	4.14	-
**11a**	23.41	0.10	26.61	7.91	-	9.74	21.17	0.79	6.14	4.13
**11a+14** (1:1)	26.19	-	51.82	9.94	0.04	0.99	6.65	-	2.14	2.23
**14**	31.07	0.25	11.92	14.86	0.26	0.97	18.41	-	23.07	-
**14a**	14.89	0.18	18.13	7.33	-	3.26	15.30	0.13	40.18	0.60
**14a+11** (1:1)	30.69	0.18	7.41	11.09	-	1.45	26.49	-	22.24	0.45

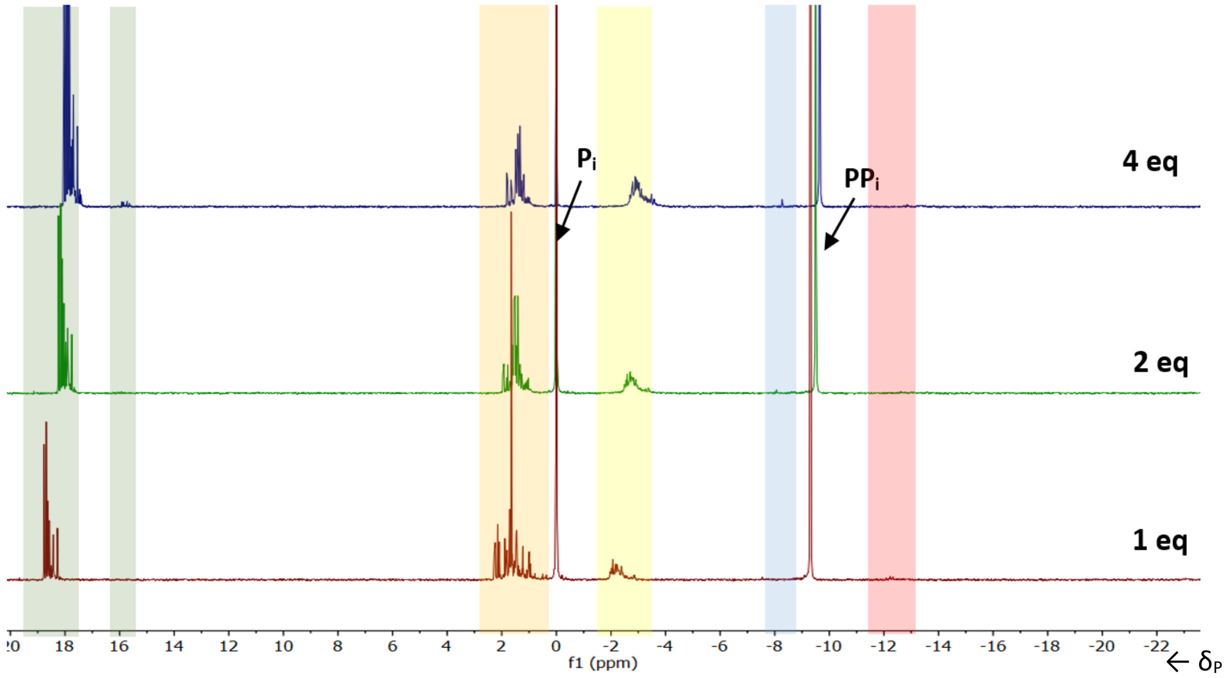

Supplementary Fig. 152. Stack of $^{31}P\{^1H\}$ NMR spectra (202.5 MHz, H_2O/D_2O = 9:1) 1 mmol scale reaction after 120 h of heating at 115 °C of crude mixtures of nucleosides **11**:**12**:**13**:**14**:**P$_i$** (0.25:0.25:0.25:0.25:1) with 1, 2 or 4 eq of **2a** with respect to **P$_i$** and 1 eq of **3a** as liquidiser. δ$_P$ (ppm) = 19.0-17.0 (*s*, 2',3' 5-membered ring cyclic phosphate **N>p,** green), 16.0-15.5 (*s*, 2',3' 5-membered ring cyclic diphosphate **pN>p**, dark green, were was identified by analogy with acyclic dinucleotides), 2.5-0.5 (5', 3' or 2' acyclic phosphates, **Np**, ochre), 0.00 (*s*, **P$_i$**), from –2.0 to –3.5 (acyclic dinucleoside phosphates, **NpN**, yellow), from –7.0 to –8.5 (*s*, 5'3' 6-membered ring cyclic phosphate **p<N,** blue), –9.5 (*s*, **PP$_i$**); –12.0 to –13.5 (n x *d*, organic diphosphates **Npp,** pink).

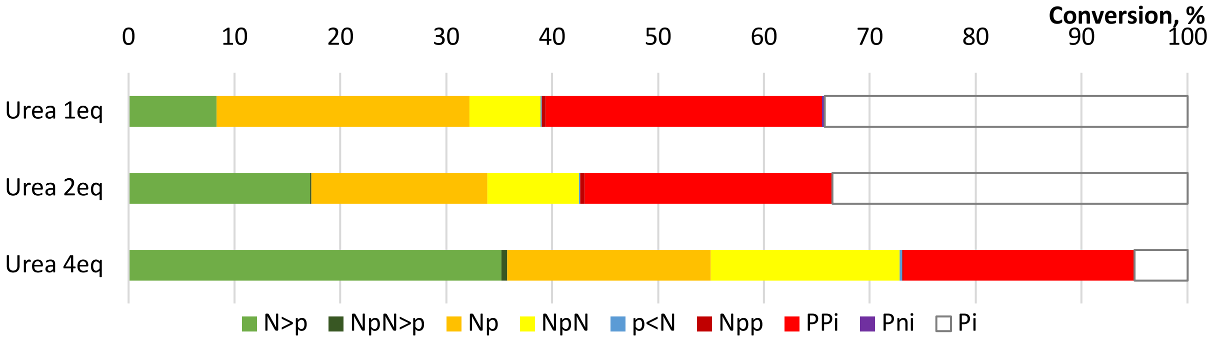

Supplementary Fig. 153. Summary of nucleosides mixture phosphorylation **11**:**12**:**13**:**14**:**P$_i$** (0.25:0.25:0.25:0.25:1) with 1, 2 or 4 eq **2a** with respect to **P$_i$** and 1 eq **3a** as liquidiser. 1 mmol scale reaction after 120 h of heating at 115 °C, then dissolved in H_2O/D_2O = 9:1. Percent values from signal integration of quantitative $^{31}P\{^1H\}$ NMR spectra (Supplementary **Fig. 152**). N>p, p<N: 2',3'-, and resp., 5',3'-cyclic nucleos(t)ide phosphates.

Supplementary Table 50. Data to Supplementary **Fig. 153**. Total conversion of initial amount of **P$_i$** to phosphorylated organic products was calculated by subtracting inorganic compound integrals (**P$_i$**, **PP$_i$** and **P$_{ni}$**) from the sum of all integrated $^{31}P\{^1H\}$ NMR peak areas (in H_2O/D_2O = 9:1). **N>p**, **p<N**: 2',3'-, and resp., 5',3'-cyclic nucleos(t)ide phosphates.

Amount of cond. agent 2a, eq	N>p, %	NpN>p, %	Np, %	NpN, %	p<N, %	Npp, %	PP$_i$, %	P$_{ni}$, %	P$_i$, %
1	8.32	-	23.86	6.72	0.11	0.37	26.1	0.26	34.26
2	17.16	0.11	16.62	8.63	0.12	0.46	23.34	-	33.56
4	35.21	0.53	19.24	17.86	0.20	0.12	21.84	-	5.00

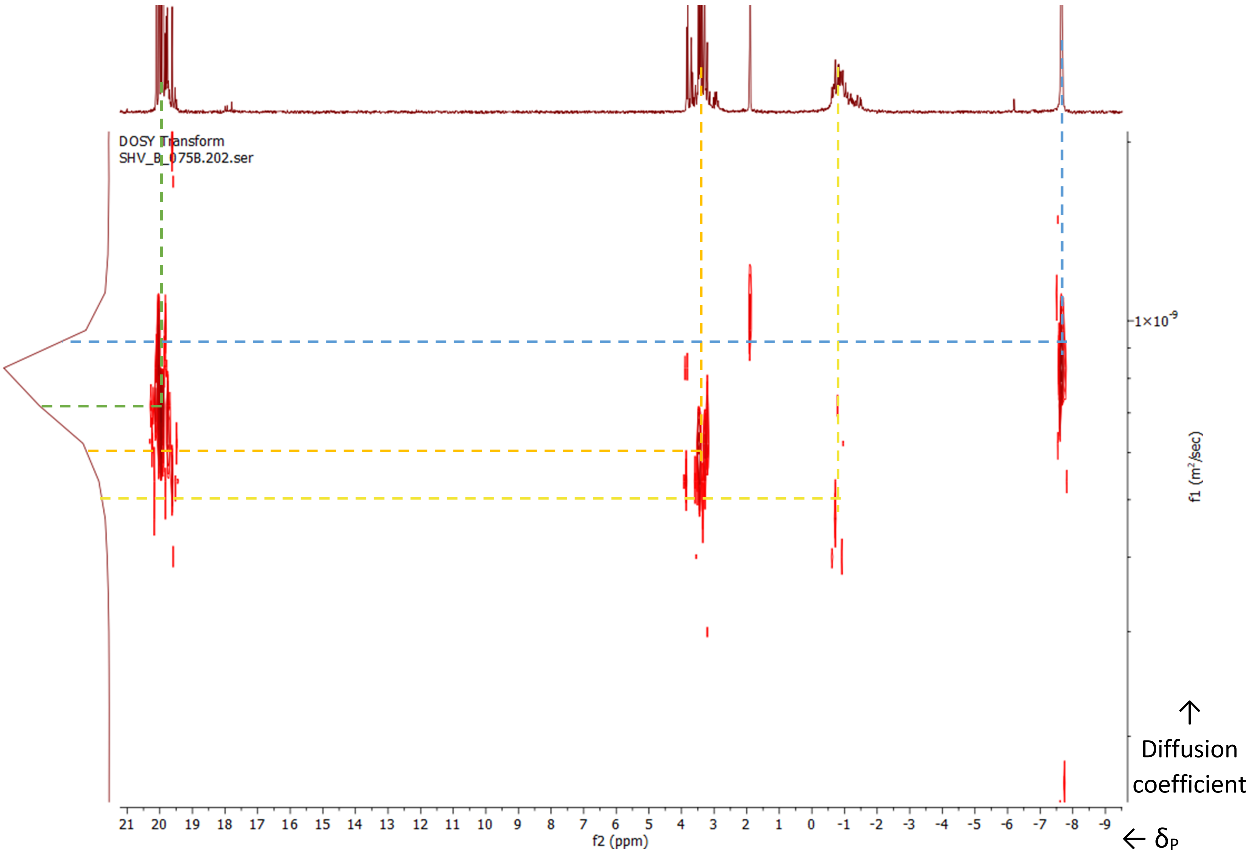

Supplementary Fig. 154. $^{31}P\{^{1}H\}$ DOSY NMR of crude equimolar mixture containing 1 equivalent nucleosides (**11**:**12**:**13**:**14** 0.25:0.25:0.25:0.25), 4 eq **2a** and 1 eq **P**$_i$., 4 mmol scale reaction after 120 h of heating at 115 °C, then dissolved in D_2O [202.5 MHz, D_2O, LED (longitudinal eddy current delay) with bipolar gradients and proton decoupling during acquisition, BBFO probe, acquisition parameters: p30 (length of the diffusion gradient) = 2.5 msec delay (δ), D20 (diffusion time) = 150 msec (Δ), 16 increments (16 spectra recorded), 128 scans each, relaxation delay = 5 sec, experiment time = 3 h, processing parameters: 2 zero fillings levels, ILT transformation]. Colour code of the dotted lines the same as the ppm areas, for example, in Supplementary **Fig. 152**.

7.5.6.1. HPLC-HRMS analysis of urea-assisted phosphorylation of a dry equimolar mixture of all ribonucleosides

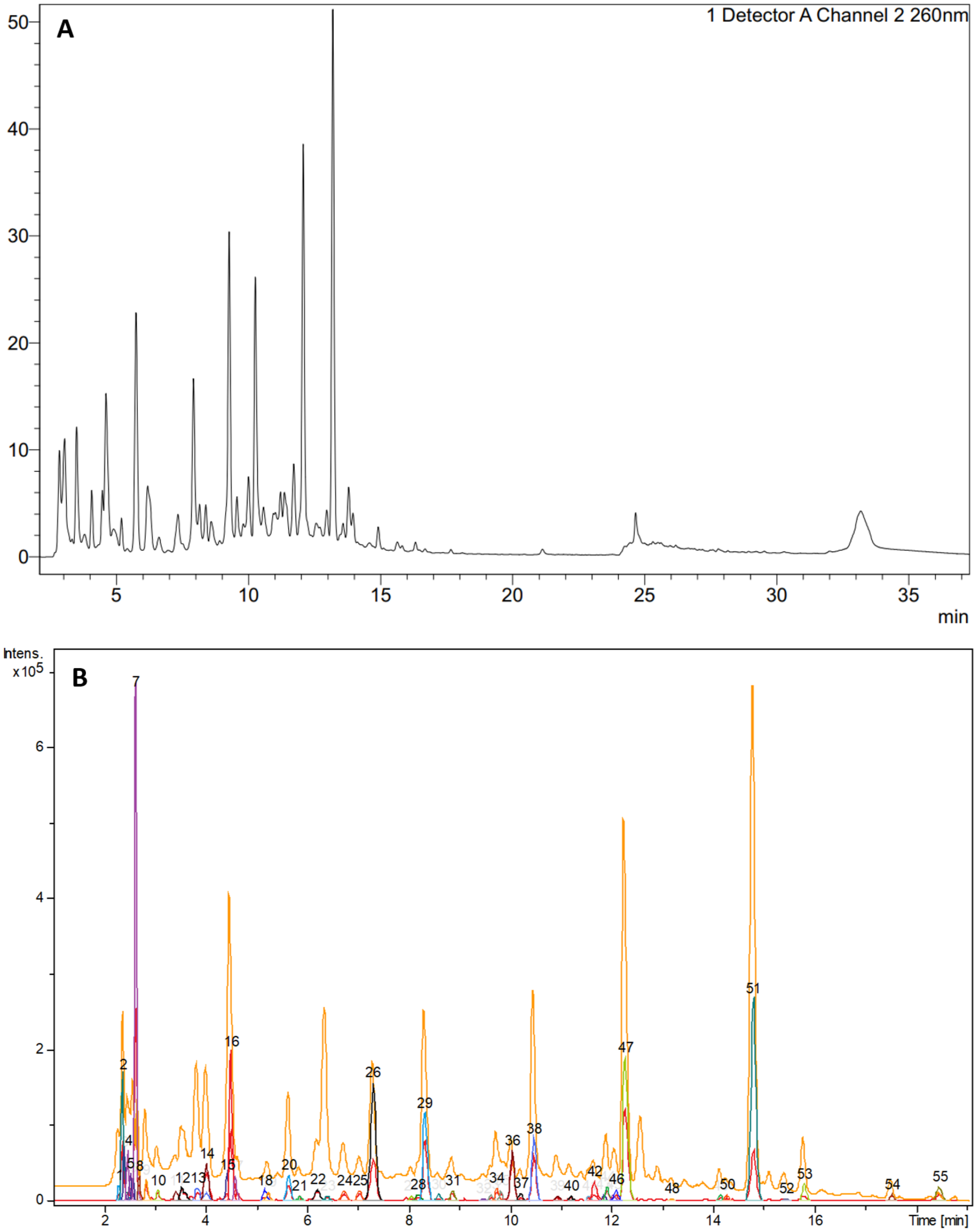

Supplementary Fig. 155. RP-HPLC of a H_2O extract of a 1 mmol scale mixture of **11**:**12**:**13**:**14**:**2a**:**P$_i$** (0.25:0.25:0.25:0.25:1:1 molar ratios) with 1 eq **3a** as liquidiser, heated neat at 115 °C for 120 h. **A** – UV(260 nm)-detected reversed-phase chromatogram, R_T = 0-37.5 min; **B** – RP-HPLC-HRMS in negative-ion mode of the same run (0-19 min); **orange**: UV chromatogram (260 nm), R_T = 0-17.5 min, **red**: total-ion chromatogram, **coloured** peaks: ion-extracted signals.

Supplementary Table 51. Interpretation of HPLC-HRMS of Supplementary **Fig. 155** with suggested formulas, retention times (**R_T**), peak areas (abs. and rel.), UV intensities and signal/noise ratio (**S/N**). The ion for all listed masses is $[M–H]^-$, if not specified otherwise. Calculations of yields (Supplementary **Fig. 157**-Supplementary **Fig. 160**) were made with taking into account the extinction coefficient for purine and pyrimidine nucleobases[37,38]. For the single peaks on the chromatogram that correspond to two or more *m/z* peaks we counted equivalent totals of all molecules found (e.g., for peak 1 having an area 1.14 and composed of 3 compounds the integrated peak area was divided by 3: 1.14/3 = 0.38). N>p : 2',3'-cyclic nucleos(t)ide phosphates ; (H_2NCO) : carbamoyl.

Number of peaks	Compound	R_T, min	Area (abs)	Area, %	Intensity (UV)	S/N
**1**	pGp, pCp, pUp	2.3	440.96	1.14	95	2038.5
**2, 3**	pC>p, pU>p, pAp,	2.4	1214.44	3.15	298	6364.8
**4**	pG>p, pGpp	2.5	804.12	2.08	179	3835.7
**5, 6**	pC, pU	2.6	815.47	2.11	178	3813.6
**8**	U>p, pA>p,	2.7	814.04	2.11	206	4401.6
**9**	pG	2.9	752.75	1.95	131	2809.9
**10**	pU	3.1	358.37	0.93	46	978.3
**11**	pU	3.4	369.23	0.96	52	1107
**12**	pA	3.6	852.38	2.21	81	1729.8
**13**	C>p	3.9	855.43	2.22	121	2593.2
**14**	C>p	4	1114.13	2.89	145	3120.5
**15, 16**	cU, U>p, UpU>p*	4.5	3127.07	8.10	586	12536.8
**17**	UpU>p	4.7	285.09	0.74	49	1056.4
**18, 19**	pI	5.3	237.37	0.62	29	620.2
**20**	C	5.7	746.52	1.93	110	2350.1
**21**	pG	5.9	287.68	0.75	30	638.3
**22**	pA	6.2	504.25	1.31	62	1320.7
**23**	Gua	6.4	680.86	1.76	76	1618.6
**24**	C>p	6.7	437.78	1.13	49	1037.6
**25**	(H_2NCO)U>p	7	498.13	1.29	49	1052.9
**26**	U	7.3	1713.94	4.44	240	5143.4
**27**	G	8.1	196.24	0.51	31	669.7
**28**	CpG>p, UpG>p,	8.2	207.25	0.54	42	909
**29**	G>p, GpG>p*	8.3	2668.66	6.92	402	8587.3
**30**	U	8.6	216.43	0.56	21	457.7
**31**	(H_2NCO)G>p	8.9	575.07	1.49	61	1302.6
**32**	-	9.5	165.79	0.43	21	457.4
**33**	UpG>p	9.6	234.9	0.61	39	827.6
**34**	I, pA, (H_2NCO)Ap, GpC	9.7	588.06	1.52	80	1697.8
**35**	U>p, G>p	9.5	296.41	0.77	104	2193.5
**36**	Ade, C	10	503.76	1.31	64	1366.8
**37**	(H_2NCO)G	10.2	225.83	0.59	26	563.6
**38**	G	10.5	2563.39	6.64	367	7839.2
**39**	ApC>p, ApU>p	10.9	539.99	1.40	51	1084.7
**40**	ApG>p	11.2	310.25	0.80	28	599.4
**41**	I, ApG>p	11.6	222.37	0.58	39	832.9
**42**	(H_2NCO)U	11.7	431.45	1.12	55	1184.8
**43, 44**	UpU, ApG, (H_2NCO)A>p	11.9	634.37	1.64	82	1756.6

(continued from last page)

Number of peaks	Compound	R_T, min	Area (abs)	Area, %	Intensity (UV)	S/N
**45, 46**	ApU>p, (H_2NCO)G, C>p	12.1	619.64	1.61	78	1650
**47**	A>p	12.3	3613.41	9.36	524	11199.5
**48**	GpU	13.2	217.35	0.56	28	592.5
**49**	A>p, ApA>p*	14.1	266.15	0.69	37	792.6
**50**	ApU	14.3	181.33	0.47	27	584
**51**	A	14.8	4870.2	12.62	715	15292.4
**52**	ApG	15.4	387.13	1.00	35	767.2
**53**	(H_2NCO)A, ApU	15.8	647.98	1.68	86	1848.6
**54**	ApA	17.5	191.68	0.50	29	612.4
**55**	(H_2NCO)Ade	18.4	104.35	0.27	11	242.7

* Dinucleotide has the same mass as the adduct of cyclic monophosphate $[2M–H]^–$

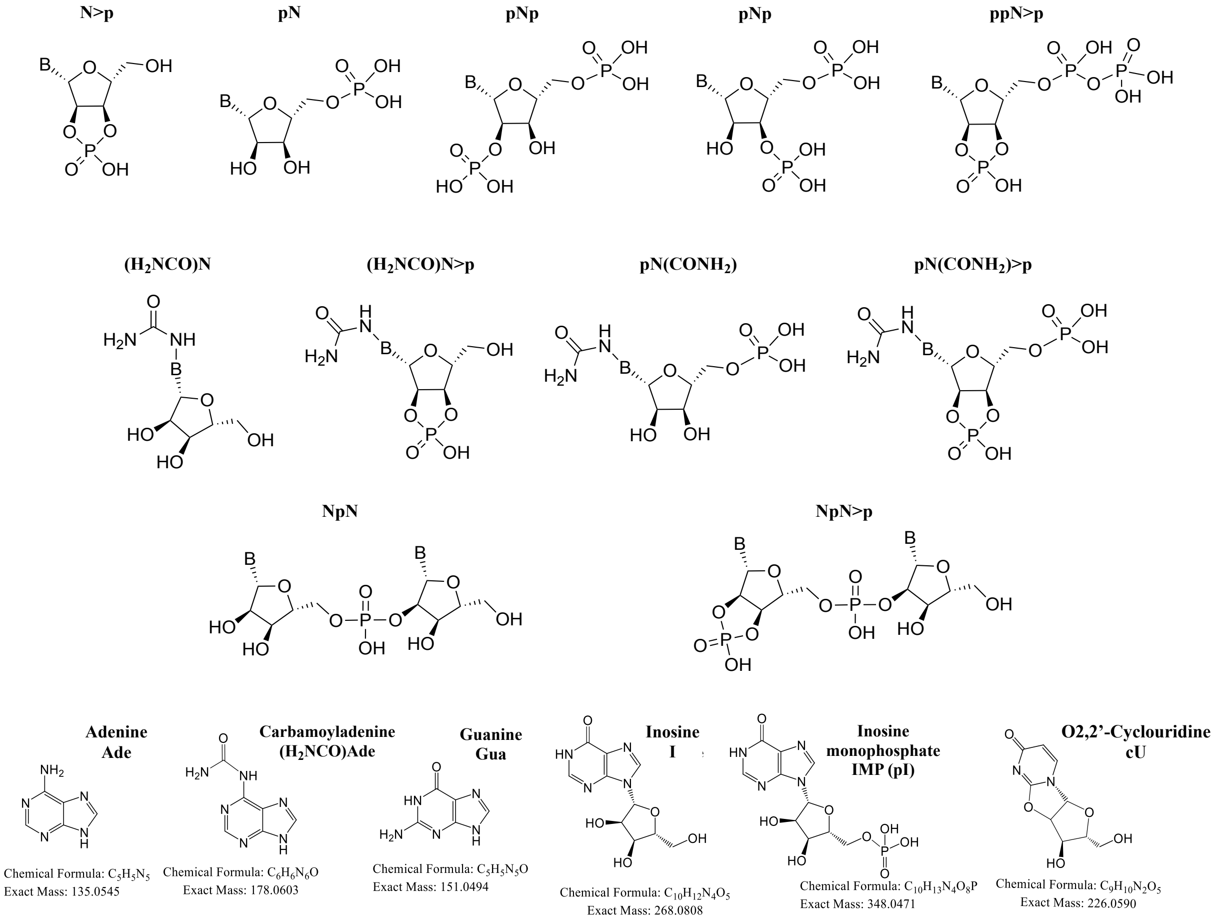

Supplementary Fig. 156. Molecular structures of the 'canonical' nucleosides N (B = Ade, Gua, Cyt, Ura), mono- and dinucleotides N>p, pN, pNp, ppN>p, NpN, NpN>p, their N-carbamoyl derivatives (only the most likely isomer shown) each representing a group of akin compounds as detected by HPLC-HRMS (Supplementary **Fig. 155**, Supplementary **Table 51**). Bottom line: chemical formulas and exact (monoisotopic) mass of the six main by-products (Ade, Carbamoyl-Ade, Gua, I, IMP, cU) denoted as "other products" in Supplementary **Fig. 157**, Supplementary **Fig. 158**, and Supplementary **Fig. 159**.

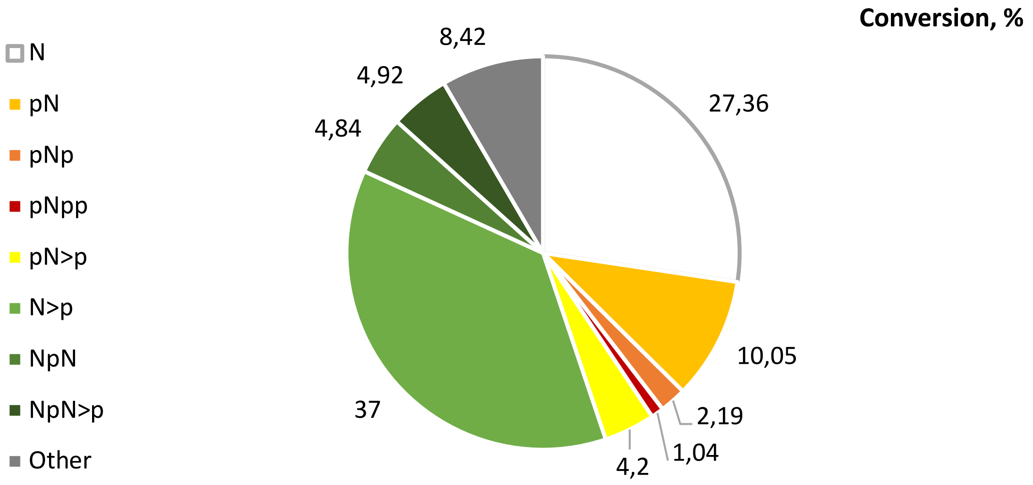

Supplementary Fig. 157. Summary of conversion of nucleosides into nucleotides obtained from the urea-assisted phosphorylation of the ribonucleoside mixture **11**:**12**:**13**:**14**:**2a**:P_i (0.25:0.25:0.25:0.25:4:1) comprising 1 eq **3a** as liquidiser. 1 mmol scale reaction after 120 h of heating at 115 °C, analysed by HPLC-HRMS. Percent values from the signal integration of the UV chromatogram (Supplementary **Fig. 155 B**).

Supplementary Table 52. Data to Supplementary **Fig. 157**. Calculations of yields were made by taking into account the difference in extinction coefficients for purine and pyrimidine nucleosides[37,38]. Total conversion of initial number of nucleosides to phosphorylated and other organic products was calculated by subtracting not reacted nucleoside integrals (**N**) from the sum of all integrated peak areas (in H_2O). **N>p** : 2',3'-cyclic nucleos(t)ide phosphates.

Type of product	N	pN	N>p	pNp	pN>p	pNpp	NpN	NpN>p	Other
%	27.36	10.05	37.00	2.19	4.20	1.04	4.84	4.92	8.42

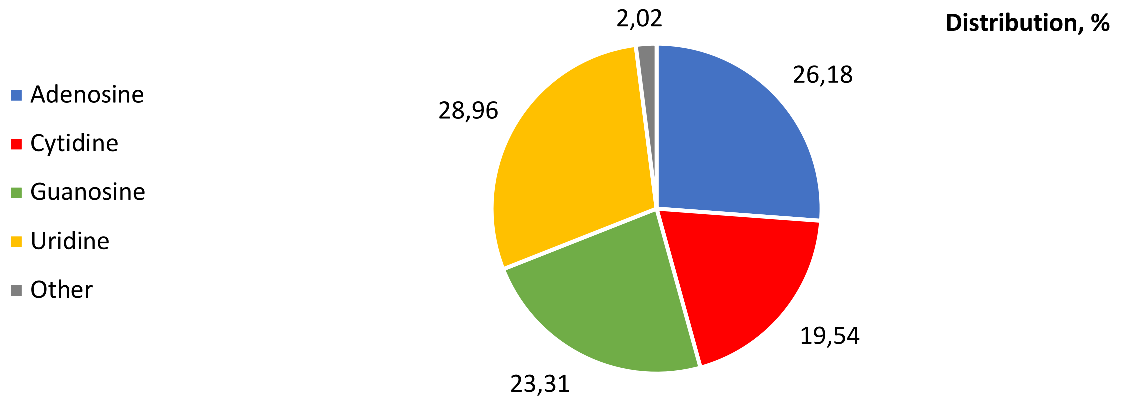

Supplementary Fig. 158. Summary of $^{31}P\{^{1}H\}$ signal intensity distribution depending on nucleobase obtained from crude mixture **11**:**12**:**13**:**14**:**2a**:**P**$_i$ (0.25:0.25:0.25:0.25:1:4) with 1 eq **3a** as liquidiser. 1 mmol scale reaction after 120 h of heating at 115 °C, analysed by HPLC-HRMS. Percent values from signal integration of UV chromatogram (Supplementary **Fig. 155 B**).

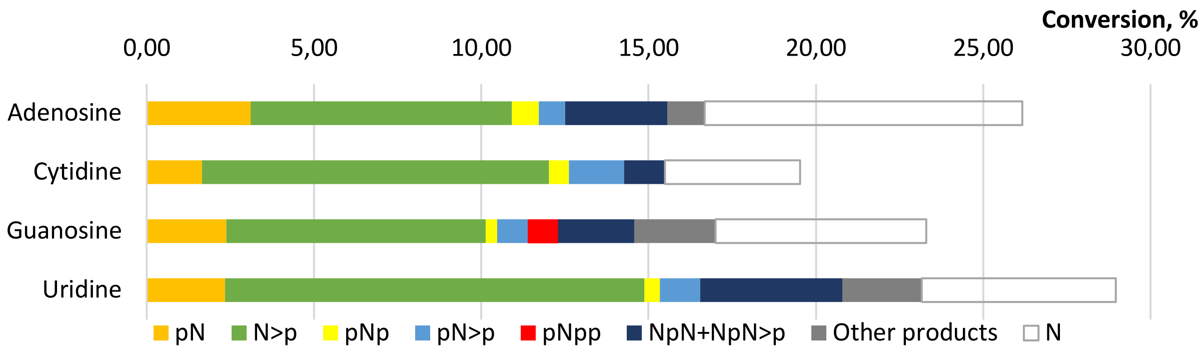

Supplementary Fig. 159. Summary of product distribution obtained from the urea-assisted phosphorylation of equimolar ribonucleoside mixture **11**:**12**:**13**:**14**:**2a**:**P**$_i$ (0.25:0.25:0.25:0.25:1:4) with 1 eq **3a** as liquidiser. 1 mmol scale reaction after 120 h of heating at 115 °C, analysed by HPLC-HRMS. Percent values from signal integration of UV chromatogram (Supplementary **Fig. 155 B**).

Supplementary Table 53. Data to Supplementary **Fig. 159**. Calculations of yields were made by taking into account the difference in extinction coefficients for purine and pyrimidine nucleosides[37,38]. Total conversion of initial number of nucleosides to phosphorylated and other organic products was calculated by subtracting not reacted nucleoside integrals (**N**) from the sum of all integrated peaks (in H_2O). **N>p** : 2’,3’-cyclic nucleos(t)ide phosphates.

Nucleoside	pN %	N>p %	pNp %	pN>p %	pNpp %	NpN+NpN>p %	Other products %	N %
**11**	4.28	10.60	1.05	1.06	-	3.45	1.49	12.62
**12**	2.70	8.80	0.38	1.04	1.04	2.58	2.75	7.15
**13**	1.06	6.78	0.38	1.05	-	0.65	-	2.59
**14**	2.02	10.83	0.38	1.05	-	3.66	2.05	5.00

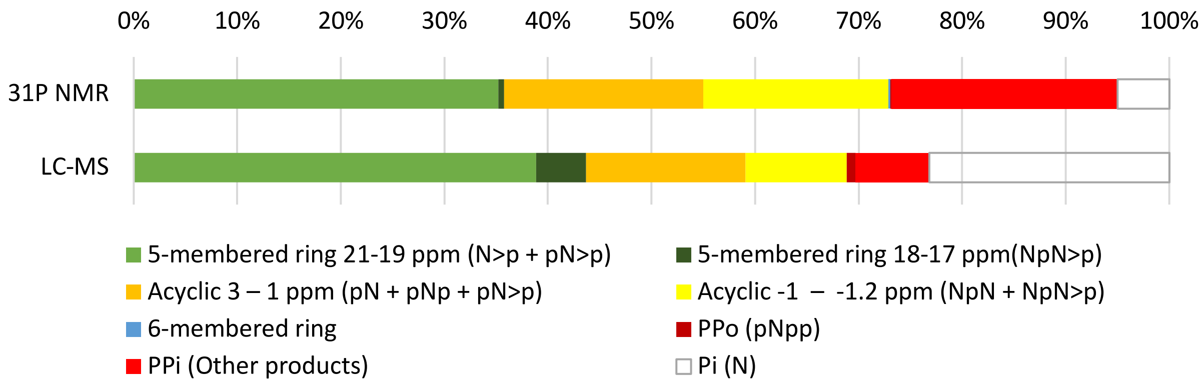

Supplementary Fig. 160. Comparison of product yields obtained by two different analytical methods, ${}^{31}P\{{}^{1}H\}$ NMR (data from Supplementary **Fig. 152**-Supplementary **Fig. 153**, Supplementary **Table 50**) and HPLC-MS (Supplementary **Fig. 155** and Supplementary **Table 52**) of reaction **11**:**12**:**13**:**14**:**2a**:**P**$_i$ (0.25:0.25:0.25:0.25:1:4) with 1 eq **3a** as liquidiser, 1 mmol scale reaction after 120 h of heating at 115 °C.

Supplementary Table 54. Data to Supplementary **Fig. 160**. **N>p**, **p<N**: 2',3'-, and resp., 5',3'-cyclic nucleos(t)ide phosphates.

	**N>p %**	**NpN>p %**	**Np %**	**NpN %**	**p<N %**	**PP$_i$ %**	**-**	**PP$_o$ %**	**P$_i$ %**
**${}^{31}P\{{}^{1}H\}$ NMR**	35.21	0.53	19.24	17.86	0.20	21.84	-	0.12	5.00
**LC-MS**	38.87	4.79	15.45	9.75	-	-	7.17	0.83	23.14
	**N>p + pN>p %**	**NpN>p %**	**pN + pNp + pN>p + pNpp %**	**NpN + NpN>p %**	**-**	**-**	**Other products, %**	**pNpp %**	**N %**

In order to compare the results obtained from two different analytical methods, we used the values from Supplementary **Table 50** and Supplementary **Table 52**. For this case, the HPLC-MS total conversion of the initial amounts of nucleosides phosphorylated and transformed to other organic products was calculated by subtracting the peak area integrals of the not reacted nucleosides (**N**) from the sum of all integrated peaks (in H_2O), which was compared to the total conversion of the initial amounts of **P**$_i$ to phosphorylated organic products as calculated by subtracting inorganic compound integrals (**P**$_i$, **PP**$_i$ and **P**$_{ni}$) from the sum of all integrated ${}^{31}P\{{}^{1}H\}$ NMR peak areas (in H_2O/D_2O = 9:1). We used the sum of products in HPLC-MS containing phosphate groups to 'match' the products in correspondence to ${}^{31}P\{{}^{1}H\}$ NMR, after scaling the total number of products to 100 %.

7.5.7. Urea-assisted phosphorylation of a mixture of ribonucleosides in the presence of valine

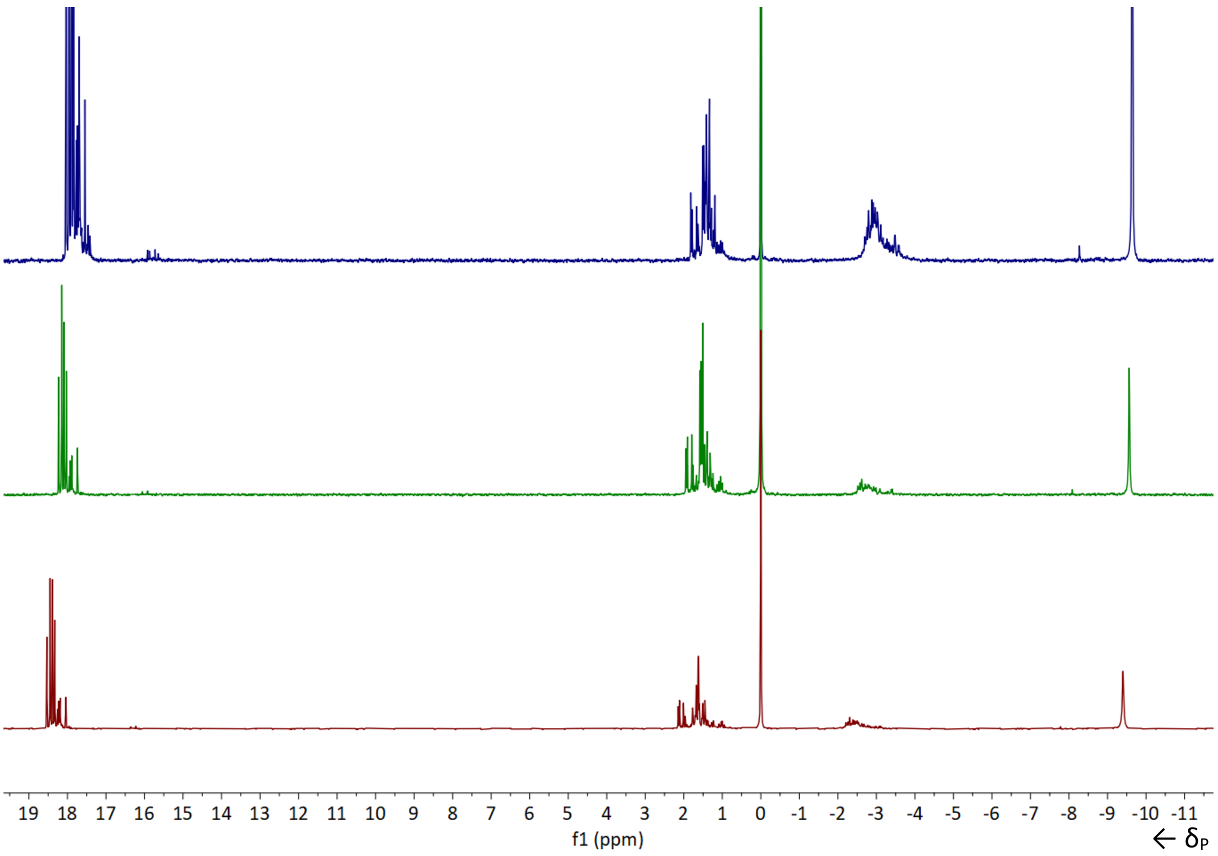

Supplementary Fig. 161. Stack of ^{31}P{^{1}H} NMR spectra (202.5 MHz, D_2O) of a 1 mmol scale reaction after 120 h of heating at 115 °C of crude mixtures of nucleosides **11**:**12**:**13**:**14**:**2a**:**P$_i$** (0.25:0.25:0.25:0.25:4:1) and 1 eq **3a** as liquidiser: **upper spectrum** – spectrum from Supplementary **Fig. 152**; **middle spectrum** – with 1 eq D-valine (**22c**) with respect to **P$_i$**; **lower spectrum** – with 1 eq L-valine (**22b**) with respect to **P$_i$** . δ_P (ppm) = 19.0-17.0 (*s*, 2',3' 5-membered ring cyclic phosphates **N>p**), 16.0-15.5 (*s*, 2',3' 5-membered ring cyclic diphosphate **pN>p**, identified by analogy with signal intensities of acyclic dinucleotides), 2.5-0.5 (5', 3' or 2' acyclic phosphates, **Np**), 0.00 (*s*, **P$_i$**), from –2.0 to –3.5 (acyclic dinucleoside phosphates, **NpN**, yellow), from –7.0 to –8.5 (*s*, 5',3' 6-membered ring cyclic phosphate **p<N**), –9.5 (*s*, **PP$_i$**).

Supplementary Table 55. Data to Supplementary **Fig. 161**. Total conversion of initial amount of **P$_i$** to phosphorylated organic products was calculated by subtracting inorganic compound integrals (**P$_i$** and **PP$_i$**) from the sum of all integrated ^{31}P{^{1}H} NMR peak areas (in D_2O). **N>p**, **p<N**: 2',3'-, and resp., 5',3'-cyclic nucleos(t)ide phosphates.

Type of product, 1eq	N>p, %	NpN>p, %	Np, %	NpN, %	p<N, %	Npp, %	PP$_i$, %	P$_i$, %
D-Valine	14.14	0.22	27.36	6.38	0.10	-	7.99	43.81
L-Valine	24.07	0.25	28.83	11.25	0.09	-	11.64	23.87

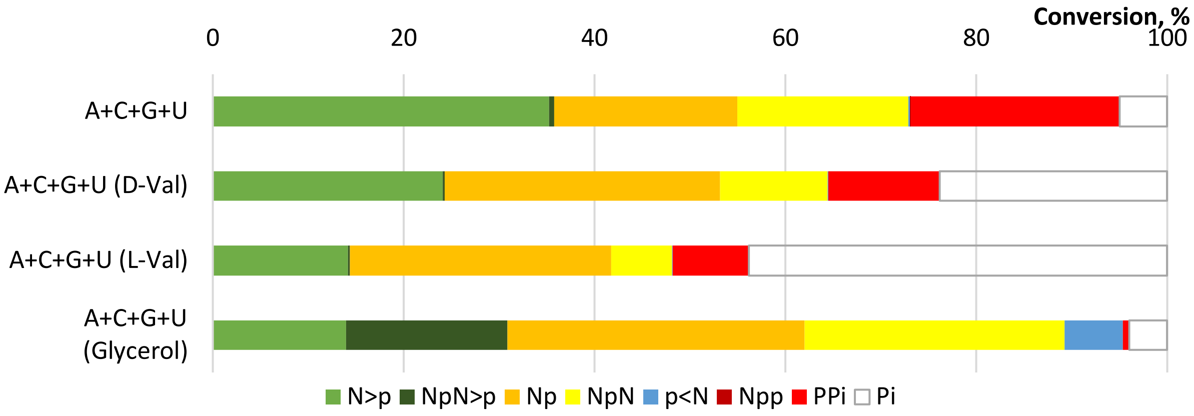

Supplementary Fig. 162. Summary of urea-assisted phosphorylations of ribonucleoside mixture **11**:**12**:**13**:**14**:**2a**:**P**$_i$ (0.25:0.25: 0.25:0.25:4:1) and 1 eq **3a** as liquidiser in absence and presence of 1 eq amino acid **22a**, **22b**, or glycerol (**5**); 1 mmol scale reaction after 120 h of heating at 115 °C, then dissolved in D_2O on a 0.5 mmol scale. N>p, p<N: 2',3'- and, respectively, 5',3'-cyclic nucleos(t)ide phosphates. Percent values from signal integration of quantitative $^{31}P\{^1H\}$ NMR spectra (Supplementary **Fig. 161** and Supplementary **Fig. 164**).

7.5.8. Supplementary conclusion on nucleoside phosphorylation

Interestingly, the phosphorylation of adenosine at 115 °C was more efficient in 'dry' mixtures that contained only a small amount of liquidising formamide (about 0.5 mol equivalent, i.e., two drops at a 0.5 mmol scale) than in 'wet and evaporating' mixtures where a large excess of water was initially added (1 ml at the same scale, cf. Supplementary **Table 44**) forming an almost clear solution that eventually dried out. Both milieus produced unusually large amounts of inorganic condensed phosphates, that is, **PP**$_i$ (inorganic pyrophosphate) and, curiously, only under wet and evaporating conditions also much **PPP**$_i$ (inorganic triphosphate). The pristine pyrimidine ribonucleosides uridine (U) and cytidine (C) were more efficiently phosphorylated than the 1,3-diol thymidine (T) and much more than the isolated purine ribonucleosides adenosine (A) and guanosine (G) that probably had an issue with sufficient mobilisation in a 'dry' 1:1:1 blend of nucleoside + **P**$_i$ + urea or + cyanamide despite the small amounts of liquid formamide that were added to all nucleoside mixtures (Supplementary **Table 45**). A 4-fold molar excess urea (nucleoside/**P**$_i$/urea 1:1:4) substantially enhanced the phosphorylation of pure A and pure G (Supplementary **Table 47**), produced more 2',3'-cyclic phosphates and significant amounts of dimerised products, thus phosphodiesters, most likely 5'-2'/5'-3'-regioisomeric mixtures containing ApA and GpG residues in amounts comparable to those of UpU and CpC.

When A was replaced with AMP, U replaced with UMP, A combined with UMP or U with AMP, along with **P**$_i$ and urea each 1:1:4 and, respectively, 1:1:1:4, most of the added UMP and AMP was consumed after 5 days at 115 °C (Supplementary **Table 49**) to give more additionally phosphorylated dinucleotides. Unlike any other alcohols tested under dry conditions, the phosphorylation of nucleosides (N) produced visible amounts of organic pyrophosphates NppN, especially from T (13 % TppT), along with predictably larger amounts of **PP**$_i$.

7.6. One-pot experiment with glycerol, the ribonucleosides, C_{10}:C_{11}:C_{12}:C_{13} alkanoic acids and urea as a condensing agent

Four natural ribonucleosides (A, C, G, U), glycerol, four short, saturated alkanoic acids ($CH_3(CH_2)_8COOH$, $CH_3(CH_2)_9COOH$, $CH_3(CH_2)_{10}COOH$, $CH_3(CH_2)_{11}COOH$), urea and NaH_2PO_4 were heated as a neat mixture at 115 °C for 2-5 days, and the products were analysed by HRMS and NMR spectroscopy. Due to the chemical diversity of the expected reaction products, we used different solvents for the highest yield of extraction, for nucleic acid derivatives water (D_2O and H_2O for NMR and MS, respectively) and for 'fatty' acids and possibly alkanoic glyceryl esters DMSO-*d*6 and MeOH for NMR and MS, respectively.

7.6.1. NMR analyses of products

The ^{1}H NMR spectrum of a D_2O extract of the crude mixture is shown together with the reference spectra of 5’-AMP (**11a**), 5’-GMP (**12a**), 5’-CMP (**13a**), 5’-UMP (**14a**) and glyceryl-1- and -2-phosphate (**5a**), cf. Supplementary **Fig. 163**. The presence of molecules similar to the suggested nucleotides was detected in the mixture of many different products. Nucleoside-containing products resonate in the range δ_H = 7.75-8.25 and 8.00 ppm (H6-pyrimidine and H2,H8-purine protons), 5.80-6.25 ppm (H5-pyrimidine and 1’-ribose protons) and 3.60-4.50 (2’,3’,4’ and 5’-ribose protons). The products of phosphorylation are identified at 4.0-5.5 ppm. Glycerol is detected at δ_H = 3.40-3.60 ppm and its phosphorylated products are found at 3.60-4.50 ppm. The signals below 3.5 ppm are all from protons in the alkanoic acid chains.

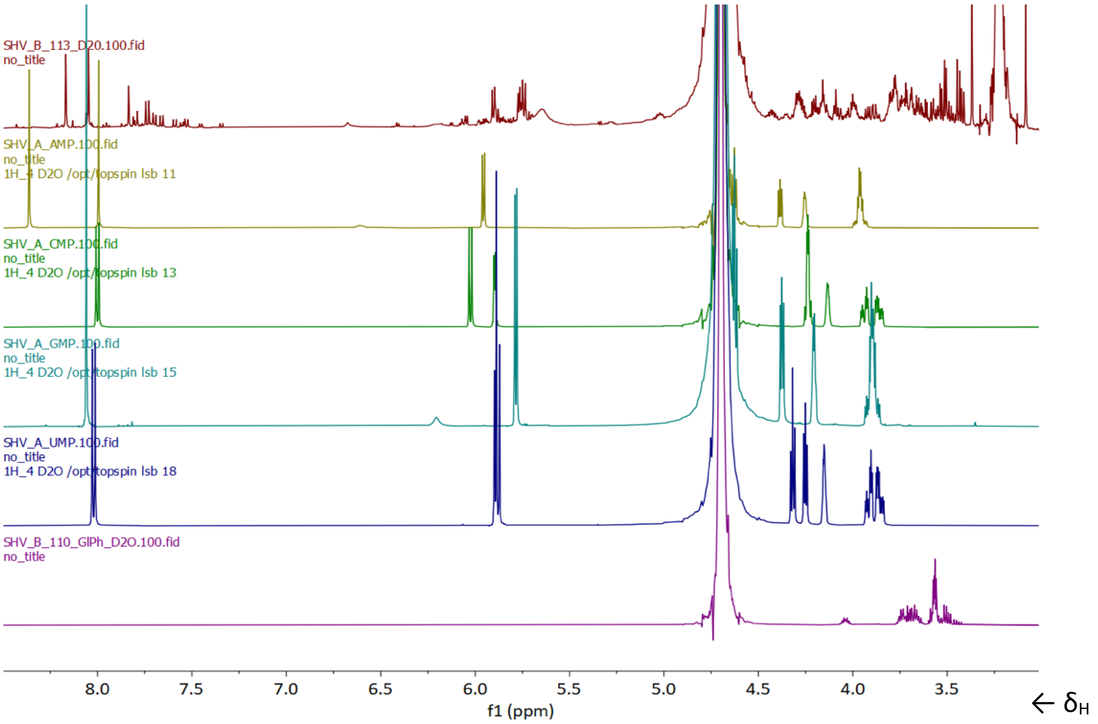

Supplementary Fig. 163. **Upper**: ^{1}H NMR spectrum (500 MHz) of the crude mixture containing 1 eq nucleosides (**11**:**12**:**13**:**14** 0.25:0.25:0.25:0.25), 1 equivalent glycerol (**5**), 1 equival. C_{10}:C_{11}:C_{12}:C_{13} alkanoic acids (**7a**:**7b**:**7c**:**7d** 0.25:0.25:0.25:0.25), 1 eq **2a** and 1 eq $\mathbf{P_i}$, 4 mmol scale reaction after 48 h of heating neat at 115 °C, then dissolved in D_2O. The stack was referenced to δ_H = 4.7 ppm (*s*, D_2O). Reference spectra: **olive** – 5’-AMP, **green** – 5’-CMP, **light blue** – 5’-GMP**, dark blue** – 5’-UMP and **violet** – glyceryl-1- and -2-phosphate.

In the $^{31}P\{^{1}H\}$ NMR spectrum shown in Supplementary **Fig. 164A** we see products similar to those of previously studied nucleoside reactions, namely, δ_P = 20-16 ppm 5-membered ring cyclic phosphates; 2.5-0.5 ppm acyclic phosphate monoesters; 0.00 ppm referenced to $\mathbf{P_i}$; from –0.2 to –2.5 ppm acyclic phosphate diesters; from –5.5 to –6.5 ppm 6-membered ring cyclic phosphates and –9.1 ppm $\mathbf{PP_i}$. The first four singlets in Supplementary **Fig. 164B**, δ_P = 18.94, 18.86, 18.83 and 18.80 ppm with relatively close intensities and integration values 2.36, 3.66, 2.54 and 2.24 %, respectively, are most likely due to 2’,3’-cyclic nucleoside phosphates corresponding to A, C, G or U. Without synthetic reference compounds, it was impossible to assign each of these resonances. Other small signals at δ_P = 18.68-18.44 ppm (2.89 %) are due to other nucleoside-containing cyclic phosphates, such as diphosphates or dinucleotides with a nucleoside residue connected to the phosphate ring.

The largest singlet at 17.32 ppm shows 13.5 % 5-membered cyclic glyceryl phosphate. We interpret it as such, because glycerol is more reactive than the nucleosides in general and this singlet is shifted aside from the other signals. The same is true for signals δ_P = 16.95-17.09 ppm (3.7 %) that are minor derivatives of 5-membered cyclic glyceryl phosphate. The data obtained from $^{31}P\{^{1}H\}$ DOSY NMR are consistent with such an interpretation (Supplementary **Fig. 165**).

Comparing this data with $^{31}P\{^1H\}$ DOSY (Supplementary **Fig. 165**) there are signals of phosphorylated monoesters at δ_P = 0.75-2.40 ppm (29.4 %) due to acyclic nucleotides and glyceryl phosphates together, and most likely dinucleotides in the range −0.2 to −2.5. The large singlet at −4.81 ppm (5.5 %) resonates in the range of 6-membered ring phosphates. Nucleoside 3',5'-cyclic phosphates also resonate in this range, but most likely this signal is due to 6-membered ring glyceryl phosphate. In general, $^{31}P\{^1H\}$ DOSY NMR demonstrates the separation of four main product groups by molecular weight (more precisely, by their diffusion rate and calculated diffusion coefficient), as identified in Supplementary **Fig. 165**.

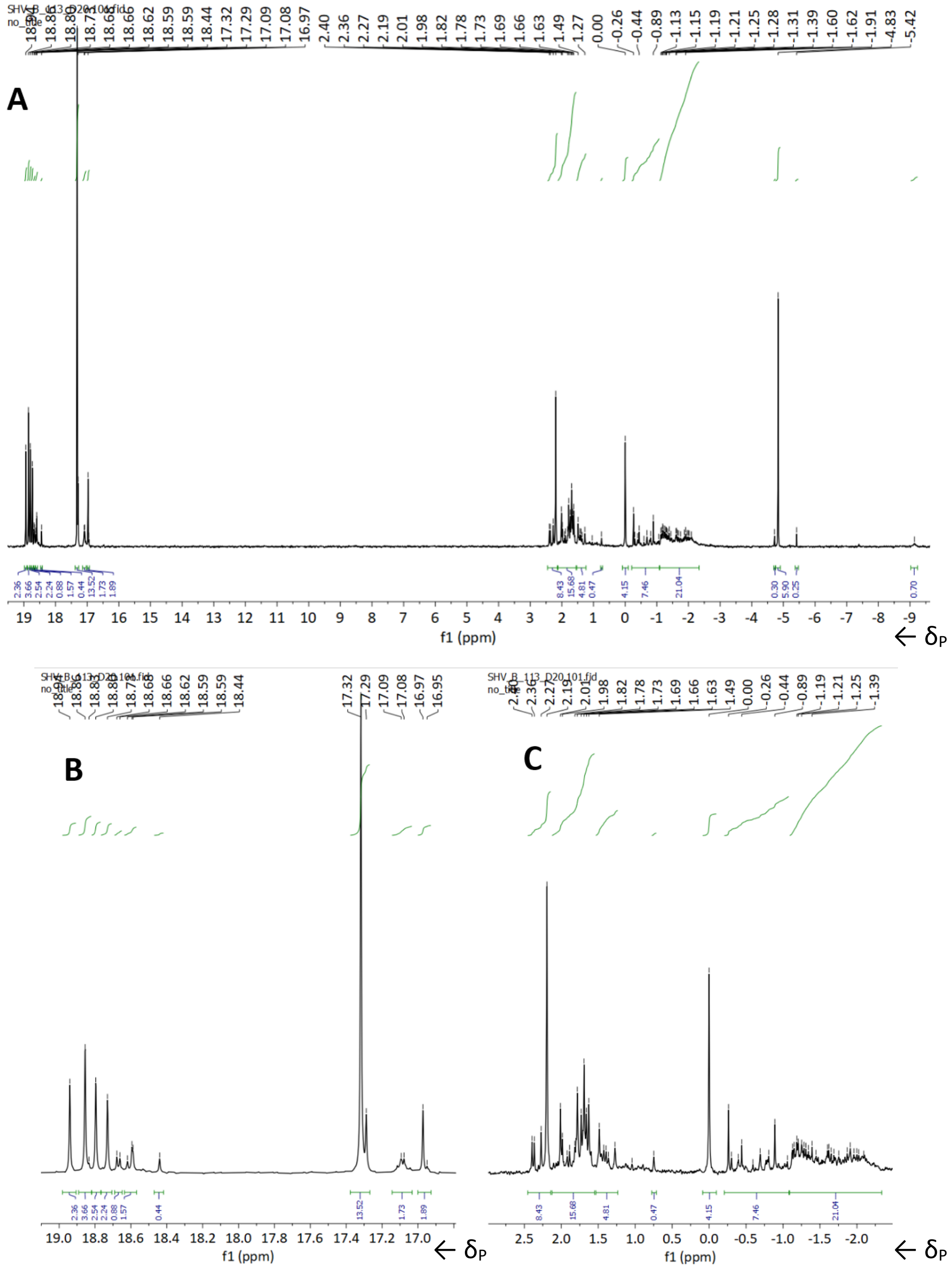

Supplementary Fig. 164. $^{31}P\{^1H\}$ NMR (202.5 MHz, D_2O) of crude mixture containing 1 equivalent nucleosides (**11**:**12**:**13**:**14** 0.25:0.25: 0.25:0.25), 1 equivalent glycerol (**5**), 1 equivalent C_{10}:C_{11}:C_{12}:C_{13} alkanoic acids (**7a**:**7b**:**7c**:**7d** 0.25:0.25:0.25:0.25), 1 eq **2a** and 1 eq $\mathbf{P_i}$, 4 mmol scale reaction after 48 h of heating at 115 °C, then dissolved in D_2O. The spectrum was referenced to δ_P = 0.00 ppm (*s*, $\mathbf{P_i}$). **A** – full spectrum, **B** – zoom on 5-membered ring cyclic phosphates (19.2-16.8 ppm), **C** – zoom on acyclic phosphates (3.0 to −2.5 ppm).

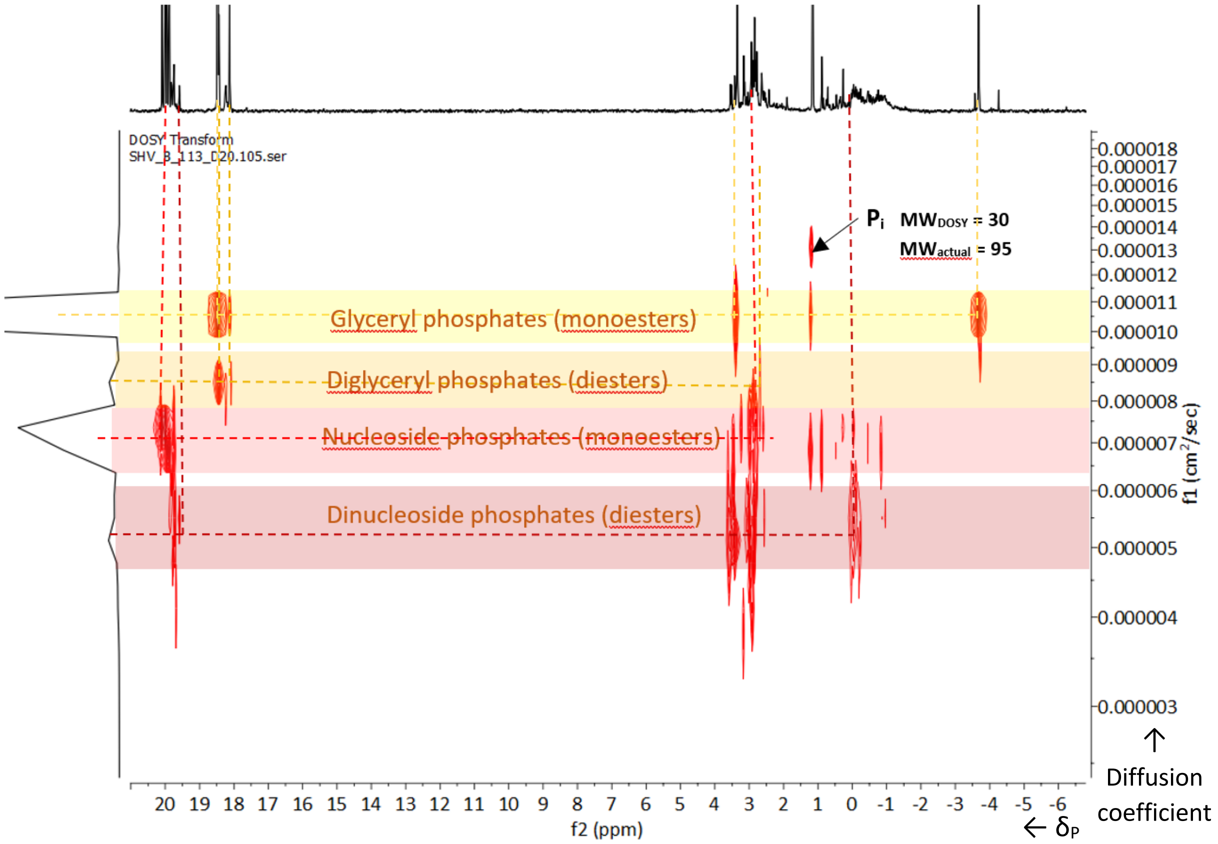

Supplementary Fig. 165. $^{31}P\{^{1}H\}$ DOSY NMR [202.5 MHz, D_2O, LED (longitudinal eddy current delay) with bipolar gradients and proton decoupling during acquisition, BBFO probe, acquisition parameters: p30 (length of the diffusion gradient) = 2.5 msec delay (δ), D20 (diffusion time) = 200 msec (Δ), 16 increments (16 spectra recorded), 128 scans each, relaxation delay = 5 sec, experiment time = 3.5 h, processing parameters: 2 zero fillings levels, ILT transformation] of crude mixture containing 1 eq nucleosides (**11**:**12**:**13**:**14** 0.25:0.25:0.25:0.25), 1 eq glycerol (**5**), 1 eq C_{10}:C_{11}:C_{12}:C_{13} alkanoic acids (**7a**:**7b**:**7c**:**7d** 0.25:0.25:0.25:0.25), 1 eq **2a** and 1 eq **P**$_i$., 4 mmol scale reaction after 48 h of heating at 115 °C, then dissolved in D_2O. The spectrum was referenced to δ_P = 0.00 ppm (*s*, **P**$_i$).

The ^{1}H NMR spectrum taken in DMSO-*d*6 of the crude mixture is shown in Supplementary **Fig. 166**. Signals at δ_H = 0.8-3.0 ppm belong to the long-chain hydrogen atoms in alkanoic acids. The overcrowded area at 3.0-4.5 ppm shows numerous glyceryl derivatives and their phosphorylated products. All other signals most likely demonstrate that some of the nucleosides and possibly nucleotides are also partly soluble in DMSO-*d*6.

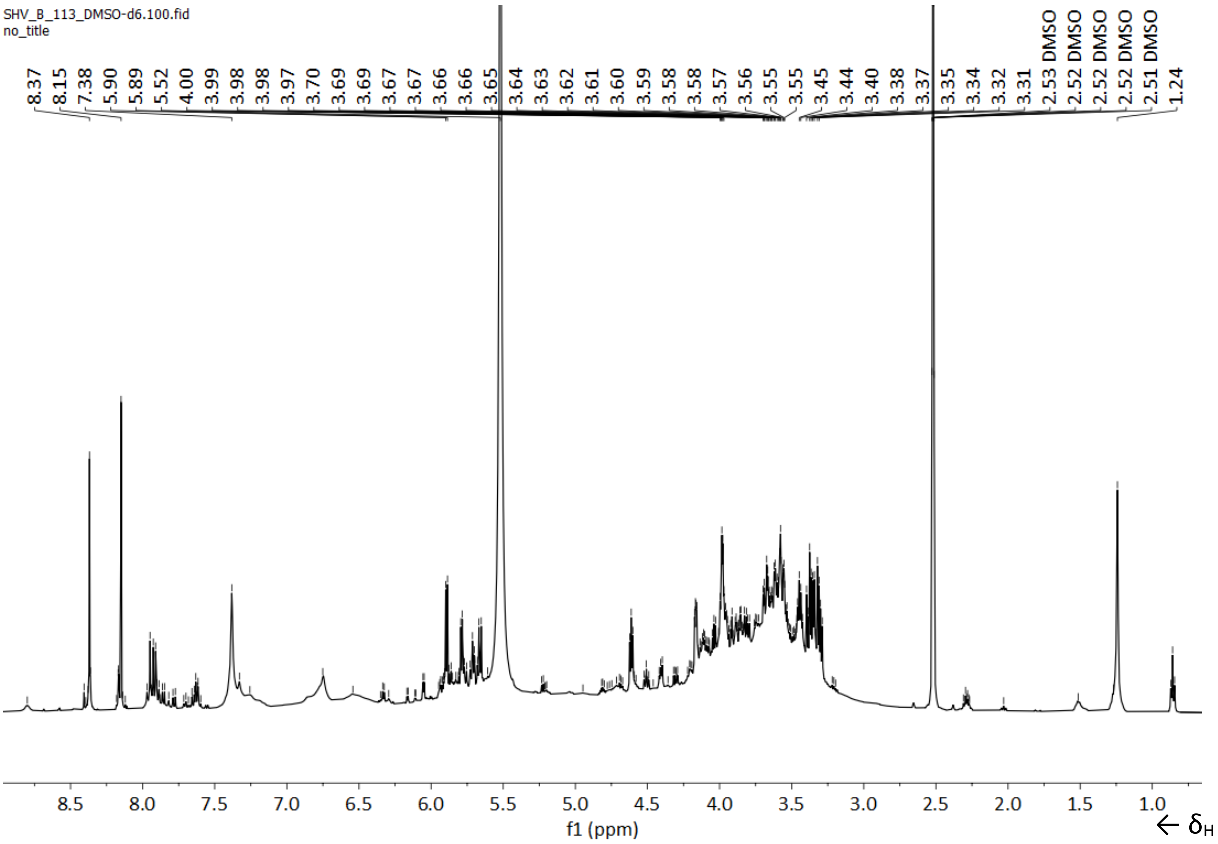

Supplementary Fig. 166. ^{1}H NMR spectrum (500 MHz) of crude mixture containing 1 equivalent nucleosides (**11**:**12**:**13**:**14** 0.25:0.25: 0.25:0.25), 1 equivalent glycerol (**5**), 1 equival. C_{10}:C_{11}:C_{12}:C_{13} alkanoic acids (**7a**:**7b**:**7c**:**7d** 0.25:0.25:0.25:0.25), 1 eq **2a** and 1 eq $\mathbf{P_i}$., 4 mmol scale reaction after 48 h of heating at 115 °C, then dissolved in DMSO-*d6*.

Although we identified in the $^{31}P\{^{1}H\}$ NMR spectrum the same groups of products, the different protons resonate too close to one another making it almost impossible to differentiate them (Supplementary **Fig. 167**). Looking into the details of the inserted zoom of 17.5-14.5 ppm range, we identify two sets of signals each with comparable intensities at 17.02 and 16.97, and at 16.83 and 16.73 ppm (total 17.6 %). These signals look like doublets; however, there is no evidence of another set of doublets in the spectrum with the same coupling constant. Therefore, we interpret them as four singlets due to 2’,3’-AMP, 2’,3’-GMP, 2’,3’-CMP and 2’,3’-UMP. In Supplementary **Fig. 164**, the signal at δ_P = 15.81 ppm (17.7 %) is due to the 5-membered ring cyclic glyceryl phosphate. The two remaining signals in the range of 15.56 and 15.11 ppm belong to more complex molecules such as phosphodiesters (2.3 %).

In the mess of signals in the acyclic area, δ_P = from 2.0 to −1.5 ppm, it is impossible to identify the $\mathbf{P_i}$ signal or confirm its absence. Most likely, we have here a mixture of different kinds of acyclic phosphoric esters that are hardly distinguishable (54.6 %). The singlet at −4.53 ppm can be attributed to 6-membered ring glyceryl phosphate (7.5 %) and another signal at −5.40 ppm, perhaps to one of its derivatives (0.4 %).

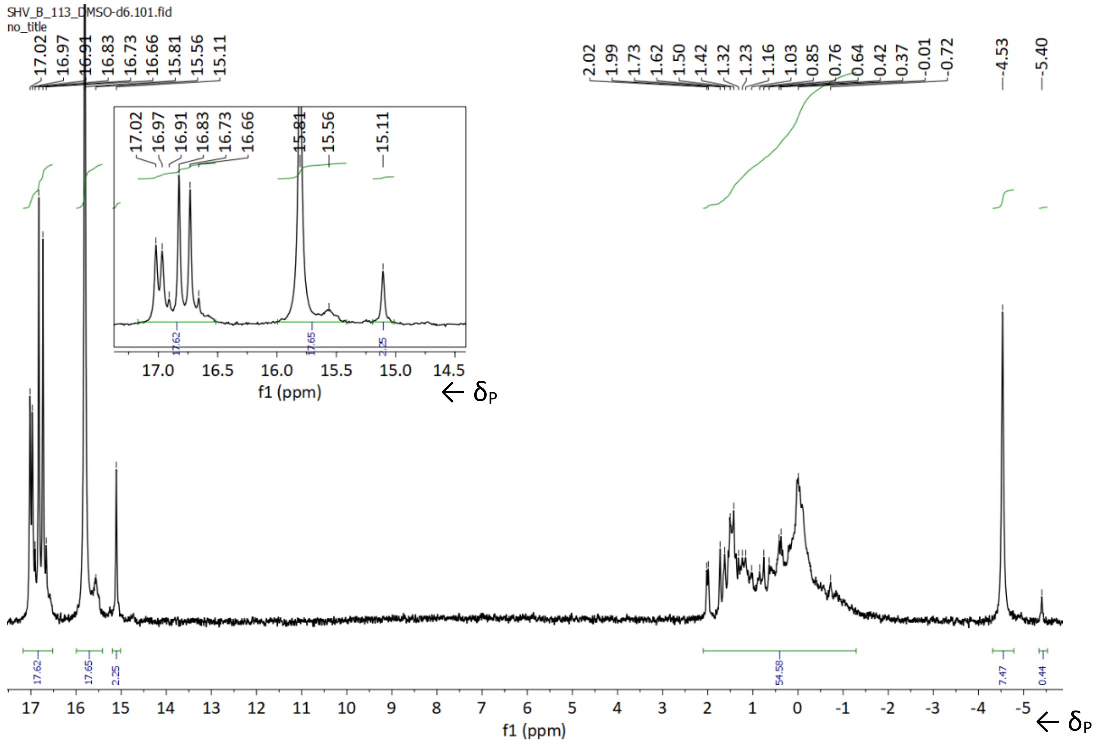

Supplementary Fig. 167. $^{31}P\{^1H\}$ NMR (202.5 MHz, DMSO-*d6*) of the crude mixture containing 1 eq nucleosides (**11**:**12**:**13**:**14** 0.25:0.25:0.25:0.25), 1 equivalent glycerol (**5**), 1 equival. C_{10}:C_{11}:C_{12}:C_{13} alkanoic acids (**7a**:**7b**:**7c**:**7d** 0.25:0.25:0.25:0.25), 1 eq **2a** and 1 eq **P**$_i$., 4 mmol scale reaction after 48 h of heating at 115 °C.

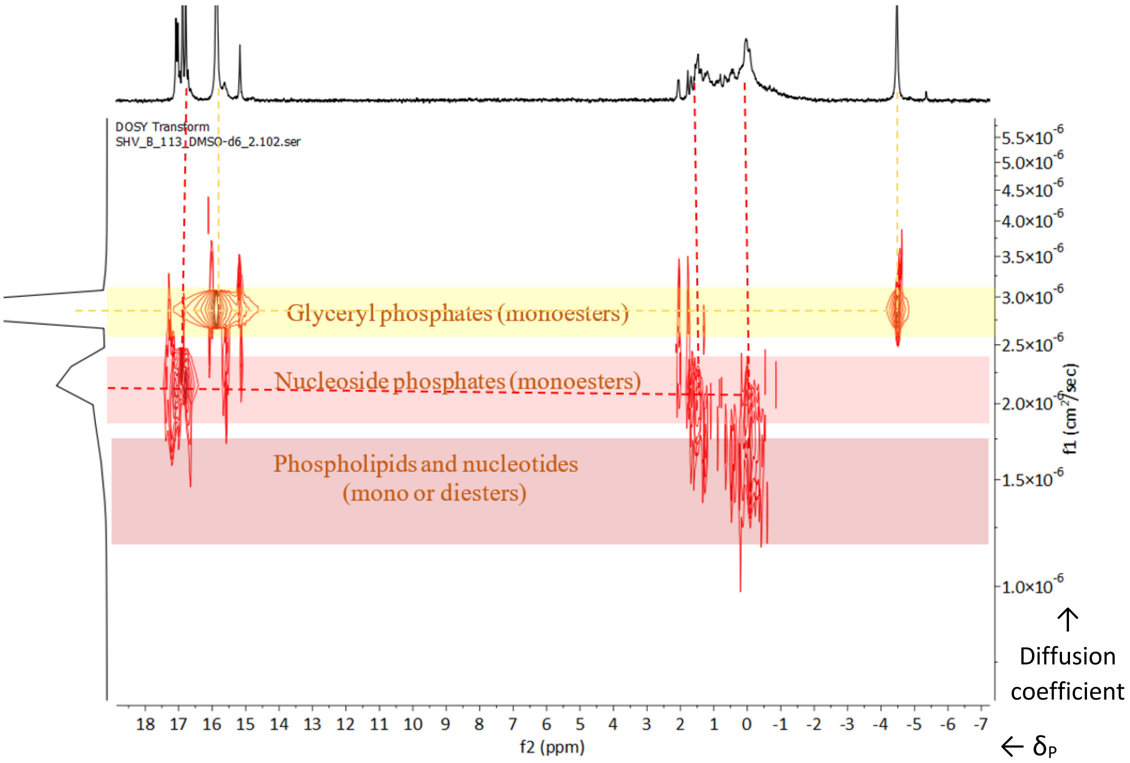

Supplementary Fig. 168. $^{31}P\{^1H\}$ DOSY NMR [202.5 MHz, D_2O, LED (longitudinal eddy current delay) with bipolar gradients and proton decoupling during acquisition, BBFO probe, acquisition parameters: p30 (length of the diffusion gradient) = 2.5 msec delay (δ), D20 (diffusion time) = 200 msec (Δ), 16 increments (16 spectra recorded), 128 scans each, relaxation delay = 5 sec, experiment time = 3.5 h, processing parameters: 2 zero fillings levels, ILT transformation] of crude mixture containing 1 eq nucleosides (**11**:**12**:**13**:**14** 0.25:0.25:0.25:0.25), 1 eq glycerol (**5**), 1 eq C_{10}:C_{11}:C_{12}: C_{13} alkanoic acids (**7a**:**7b**:**7c**:**7d** 0.25:0.25:0.25:0.25), 1 eq **2a** and 1 eq **P**$_i$, 4 mmol scale reaction after 48 h of heating at 115 °C, then dissolved in DMSO-*d6*.

The $^{31}P\{^{1}H\}$ DOSY NMR spectrum (Supplementary **Fig. 168**) confirms our suggestions for glyceryl phosphates (highlighted yellow). Part of the signals are in the same range as 2',3'-cyclyc nucleotides proposed above (highlighted pink), so we suggest that there are acyclic nucleotides present as well. In the acyclic area, we see a smooth diffusion of glyceryl monoesters towards some other heavier molecules in between the light pink and darker pink zones that are heavier (diffuse slower) than mononucleotides, possibly phospholipids and dinucleotides.

7.6.2. MS analysis of products

A mass spectrometry analysis of the crude mixture was performed in H_2O and MeOH, both in positive (LRMS) and negative (HRMS) ion modes. In water, in the positive-ion mode, we identified mostly starting molecules with the most intense signals at *m/z* 244.1, 268.1 and 284.1 that correspond to C, A and G, respectively (Supplementary **Fig. 169**). Other compounds identified are nucleobases (Ade and Ura), O- and N-carbamoyl derivatives (glyceryl carbamate, N-carbamoyl Ade and Gua) and AMP (**Ap**).

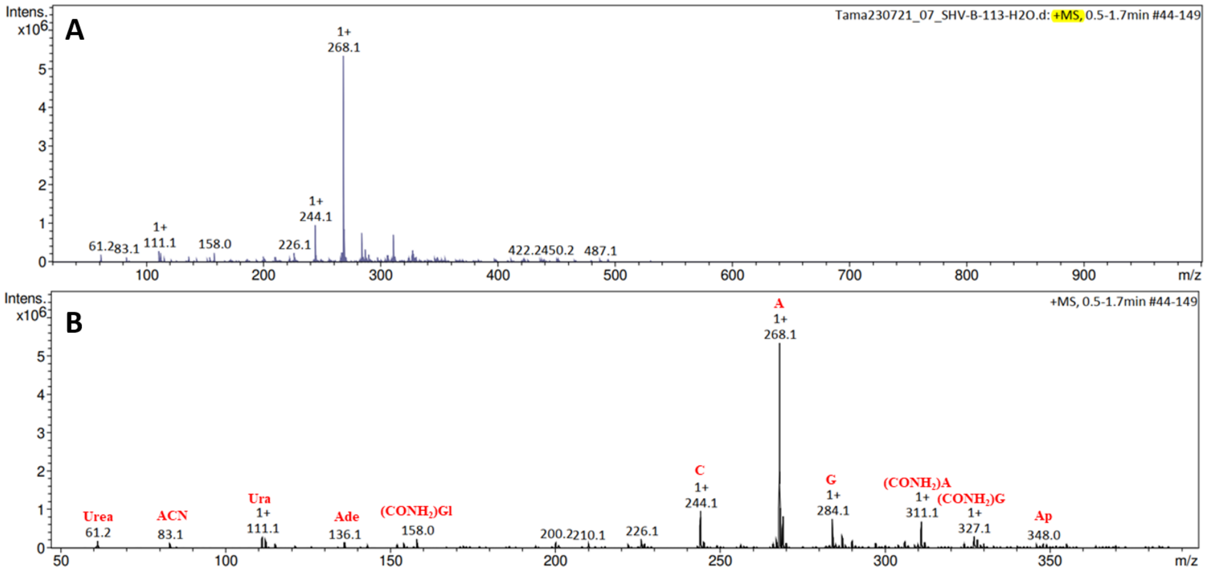

Supplementary Fig. 169. LRMS spectra (direct injection) in the positive-ion mode of the water extract of the crude mixture 1 eq nucleosides (**11**:**12**:**13**:**14** 0.25:0.25:0.25:0.25), 1 eq glycerol (**5**), 1 eq C_{10}:C_{11}:C_{12}:C_{13} alkanoic acids (**7a**:**7b**:**7c**:**7d** 0.25: 0.25:0.25:0.25), 1 eq **2a** and 1 eq $\mathbf{P_i}$., 4 mmol scale reaction after 48 h of heating at 115 °C. **A** – full spectrum, **B** – zoom into *m/z* 50-400. **Red** abbreviations are signal interpretations.

In the negative-ion mode, we identified a large diversity of organic products. In Supplementary **Fig. 170**, we show a full spectrum and its zooms dissected into *m/z* 100 ranges.

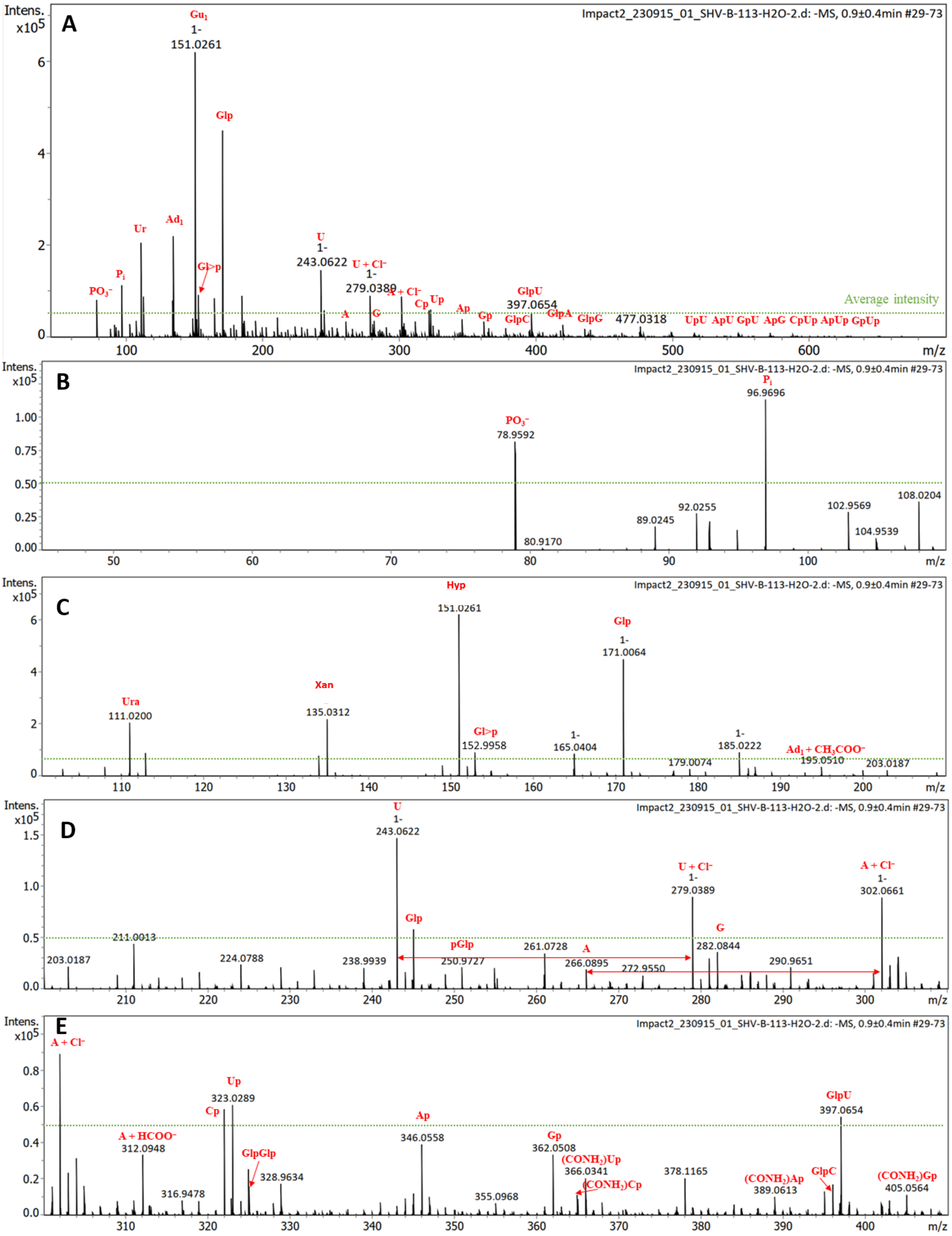

(figure continued on the next page)

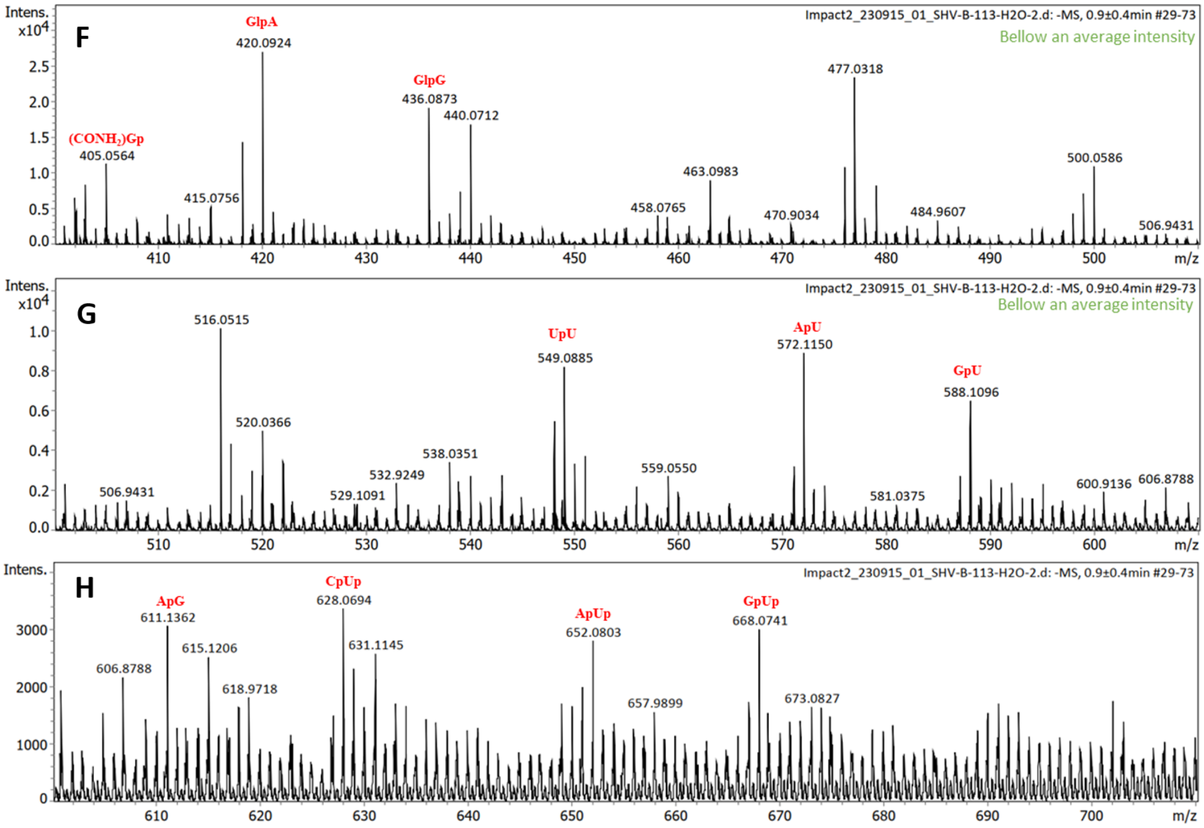

Supplementary Fig. 170. HRMS spectra (direct injection) in the negative-ion mode of the water extract of the crude mixture 1 eq nucleosides (**11**:**12**:**13**:**14** 0.25:0.25:0.25:0.25), 1 eq glycerol (**5**), 1 eq C_{10}:C_{11}:C_{12}:C_{13} alkanoic acids (**7a**:**7b**:**7c**: **7d** 0.25: 0.25:0.25:0.25), 1 eq **2a** and 1 eq **P**$_i$., 4 mmol scale reaction after 48 h of heating at 115 °C. **A** – full spectrum, **B-H** – zooms. Green dotted line is an internal gradation of the average intensity in the corresponding *m/z* range.

We were unable to identify in either the positive- or negative-ion mode the 5-membered ring nucleoside cyclic phosphates that were detected by ^{31}P{^{1}H} NMR (Supplementary **Fig. 164**). However, we confirmed the presence of doubly phosphorylated glycerol and nucleosides together with dinucleotides. There are still minor signals that were not assigned. All the identified molecules, that is, the structure of one possible isomer each are shown in Supplementary **Fig. 171**. Of note, N-carbamoyl derivatives are ureas and expectedly more stable, thus, more probable than certain isomeric exemplary O-carbamates depicted in Supplementary **Fig. 171**.[§]

In summary, the following main groups of products were identified:

- nucleobases uracil (Ura) and hydrolysed derivatives of adenine and guanine, hypoxanthine (Hyp) and, respectively, xanthine (Xan)
- nucleosides (A, G, U)
- acyclic (glyceryl, A, G, U, C) and cyclic (glyceryl) monophosphates (Glp, Gl>p, etc)
- monophosphates of the carbamoylated nucleosides A, G, C and U
- glyceryl diphosphate (pGlp)
- acyclic phosphodiesters of glycerol (GlpGl), A, G, U, C and their various combinations
- phosphorylated acyclic phosphodiesters of glycerol (GlpGlp), A, G, U, C and their combinations.

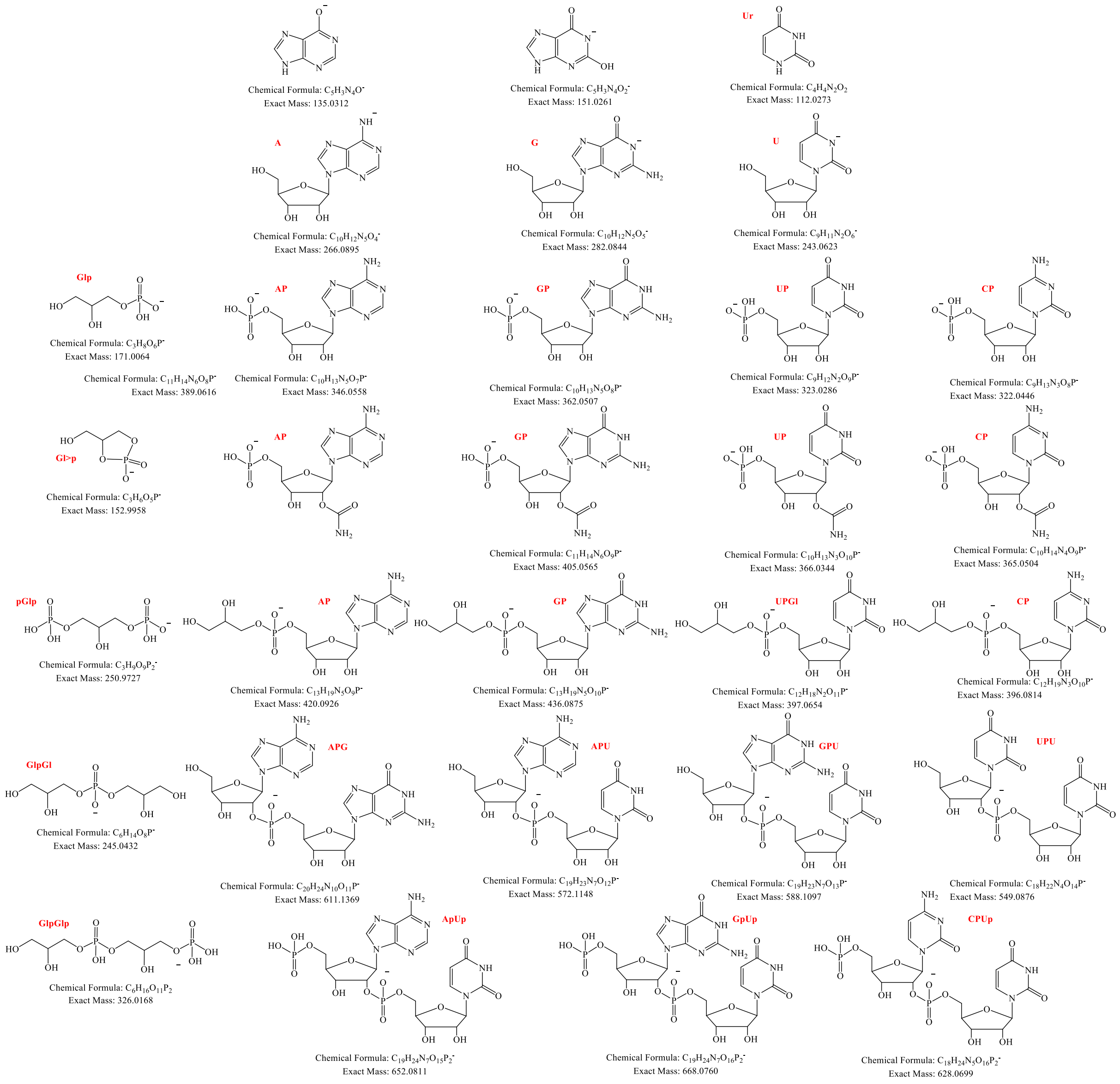

Supplementary Fig. 171. Molecular structures (only one, not necessarily the most likely isomer shown, see underlined text above§), chemical formula and monoisotopic mass of the detected compounds from the HRMS (direct injection) shown in Supplementary **Fig. 170** of the water extract of the mixture obtained by reacting 1 eq nucleosides (**11**:**12**:**13**:**14** 0.25:0.25:0.25:0.25), 1 eq glycerol (**5**), 1 eq C_{10}:C_{11}:C_{12}:C_{13} alkanoic acids (**7a**:**7b**:**7c**:**7d** 0.25:0.25: 0.25:0.25), 1 eq **2a** and 1 eq $\mathbf{P_i}$ at a 4 mmol-scale reaction after 48 h of heating at 115 °C.

As expected, we did not find in water any long-chain containing compounds other than the not esterified alkanoic acids. Hence, we performed similar MS experiments for the crude mixture dissolved in MeOH.

The positive-ion mass spectrum (Supplementary **Fig. 172**) was complicated by sodium formate clusters (pink). However, we identified signals from starting molecules A, C and G. Nevertheless, the only signal of a long-chain compound found was for the C_{12} alkanoic acid (**7c**, Supplementary **Table 1**). Of note, this spectrum has low mass resolution, so it is possible that this signal measured by HRMS would not correspond to the exact mass of the proposed formula.

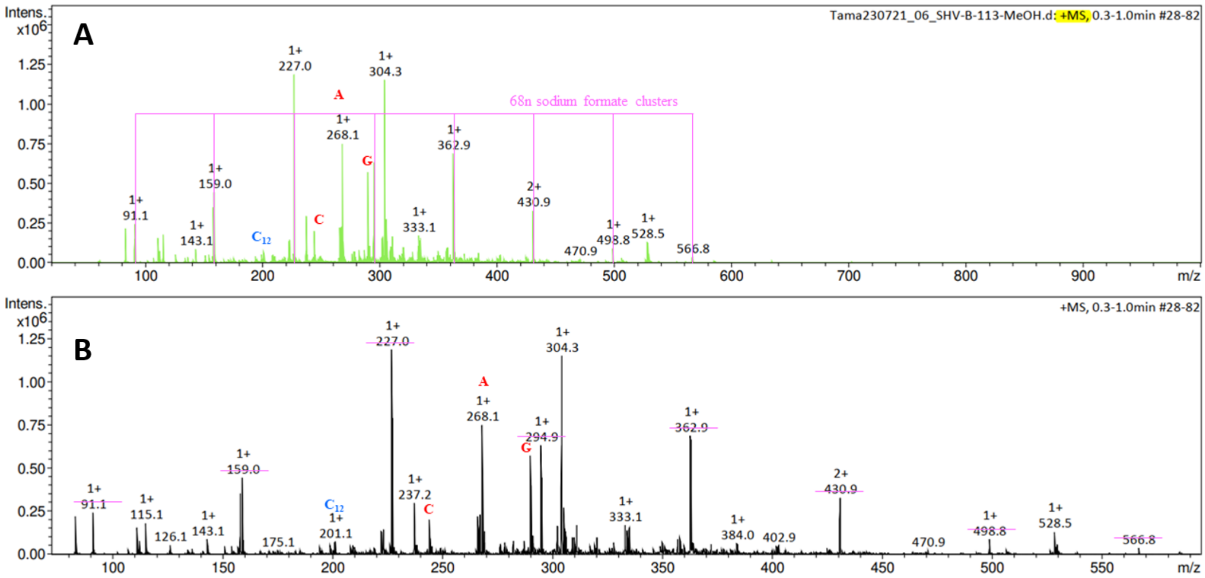

Supplementary Fig. 172. LRMS spectra (direct injection) in the positive-ion mode of the MeOH extract of the crude mixture containing 1 eq nucleosides (**11**:**12**:**13**:**14** 0.25:0.25:0.25:0.25), 1 eq glycerol (**5**), 1 eq C_{10}:C_{11}:C_{12}:C_{13} alkanoic acids (**7a**:**7b**:**7c**:**7d** 0.25:0.25:0.25:0.25), 1 eq **2a** and 1 eq **P**$_i$. 4 mmol scale reaction after 48 h of heating at 115 °C. **A** – full spectrum, **B** – zoom *m/z* 50-600. **Red** abbreviations correspond to signal interpretations that were already identified in water and **blue** signals unique for the MeOH extract.

In the negative-ion mode HRMS (Supplementary **Fig. 174**) we discovered the presence of cyclic phosphate products that were missing (Gl>p exempt) in the HRMS of the water extract. Apparently, their salts are more soluble in MeOH than in water. Although some new products were discovered (blue abbreviations), long-chain derivatives were still not found. Similar to the water extract, we did not obtain any oligomers longer than dimeric diesters. The new compounds discovered in methanol are suggested in Supplementary **Fig. 173** (the structure of the most likely isomer).

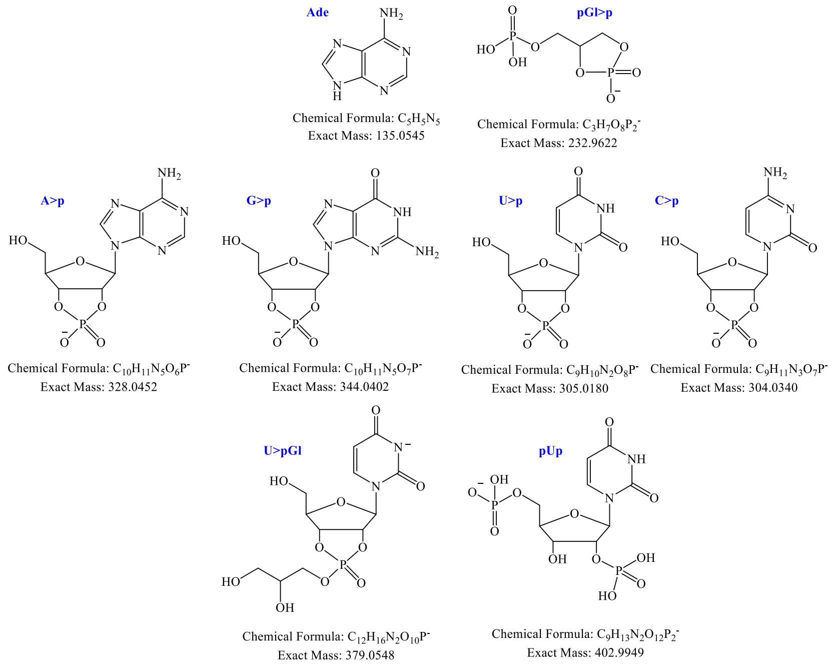

Supplementary Fig. 173. Molecular ion structures (only the most likely isomer shown), chemical formulas and monoisotopic mass of the detected compounds found by negative-ion mode HRMS of the methanol extract (shown in Supplementary **Fig. 174**) of the crude mixture obtained by reacting 1 eq nucleosides (**11**:**12**:**13**:**14** 0.25:0.25:0.25:0.25), 1 eq glycerol (**5**), 1 eq C_{10}:C_{11}:C_{12}:C_{13} alkanoic acids (**7a**:**7b**:**7c**:**7d** 0.25:0.25:0.25:0.25), 1 eq **2a** and 1 eq **P**$_i$. 4 mmol scale reaction after 48 h of heating at 115 °C.

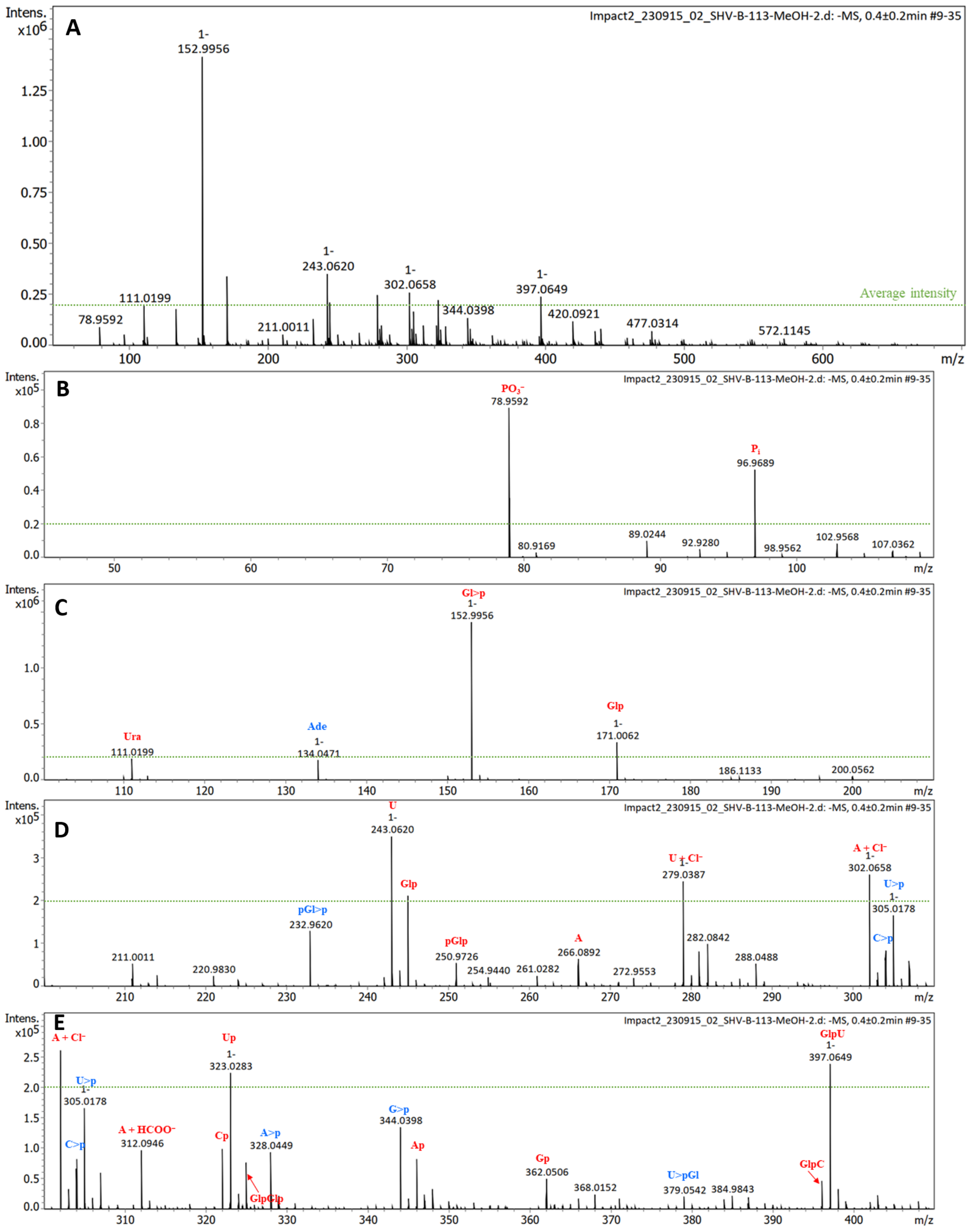

(figure continued on the next page)

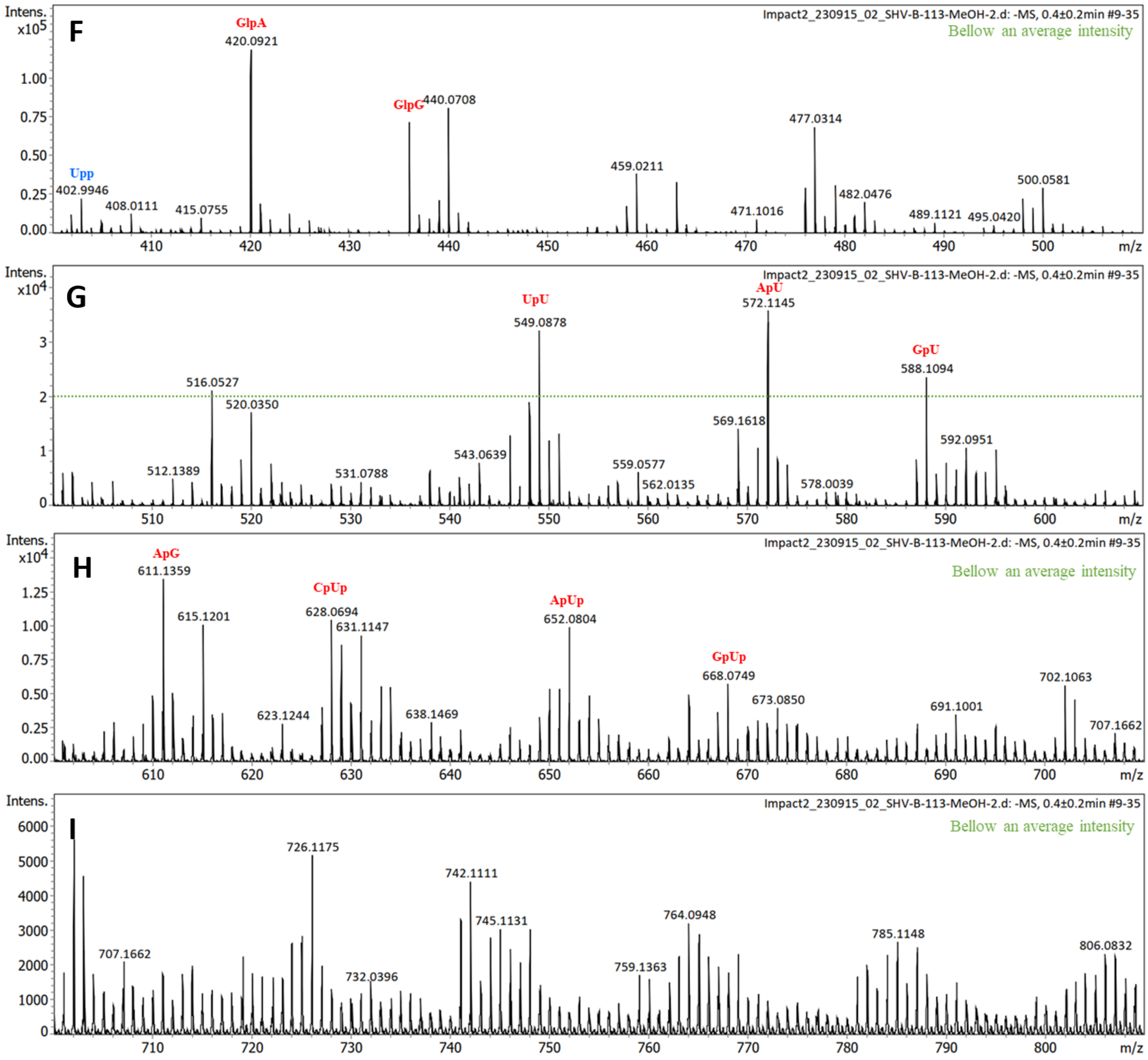

Supplementary Fig. 174. HRMS spectra (direct injection) in the negative-ion mode of the MeOH extract of the crude mixture containing 1 eq nucleosides (**11**:**12**:**13**:**14** 0.25:0.25:0.25:0.25), 1 eq glycerol (**5**), 1 eq C_{10}:C_{11}:C_{12}:C_{13} alkanoic acids (**7a**:**7b**:**7c**:**7d** 0.25:0.25:0.25:0.25), 1 eq **2a** and 1 eq **P**$_i$, 4 mmol scale reaction after 48 h of heating at 115 °C. **A** – full spectrum, **B-I** – zooms. Green dotted line is an internal gradation of the average intensity. **Red** abbreviations correspond to signal interpretation that were already identified in water and **blue** signals unique for the MeOH extract.

7.7 Phosphorylation of 2,3-*bis*-(tridecanoyl)glycerol: BTG (7)

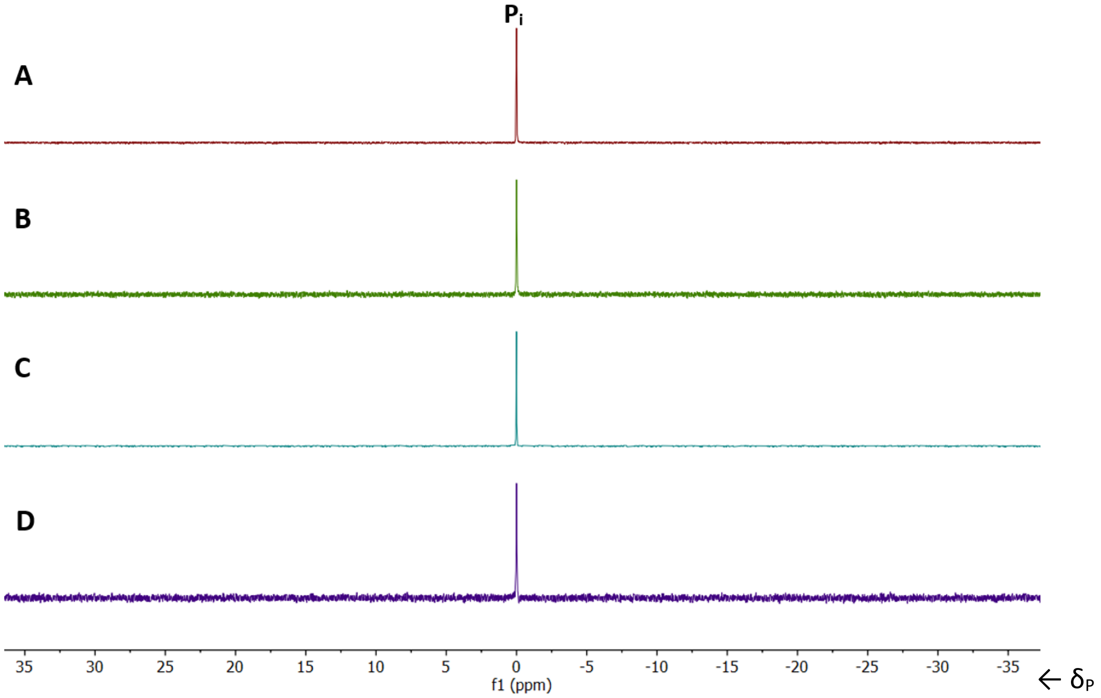

Supplementary Fig. 175. $^{31}P\{^1H\}$ NMR spectra (202.5 MHz) of crude mixtures of reactions with BTG (**7**) on a 0.5 mmol scale after 120 h of heating neat at 115 °C, then dissolved in DMSO-*d*6. On all shown spectra, we observe the presence of only one single resonance that belongs to $\mathbf{P_i}$, δ_{Pi} = 0.00 ppm:
A – reaction **7**:$\mathbf{P_i}$ (1:1); **B** – **7**:**2a**:$\mathbf{P_i}$ (1:1:1); **C** – **7**:**1**:$\mathbf{P_i}$ (1:1:1); **D** – **7**:**3a**:$\mathbf{P_i}$ (1:1:1).

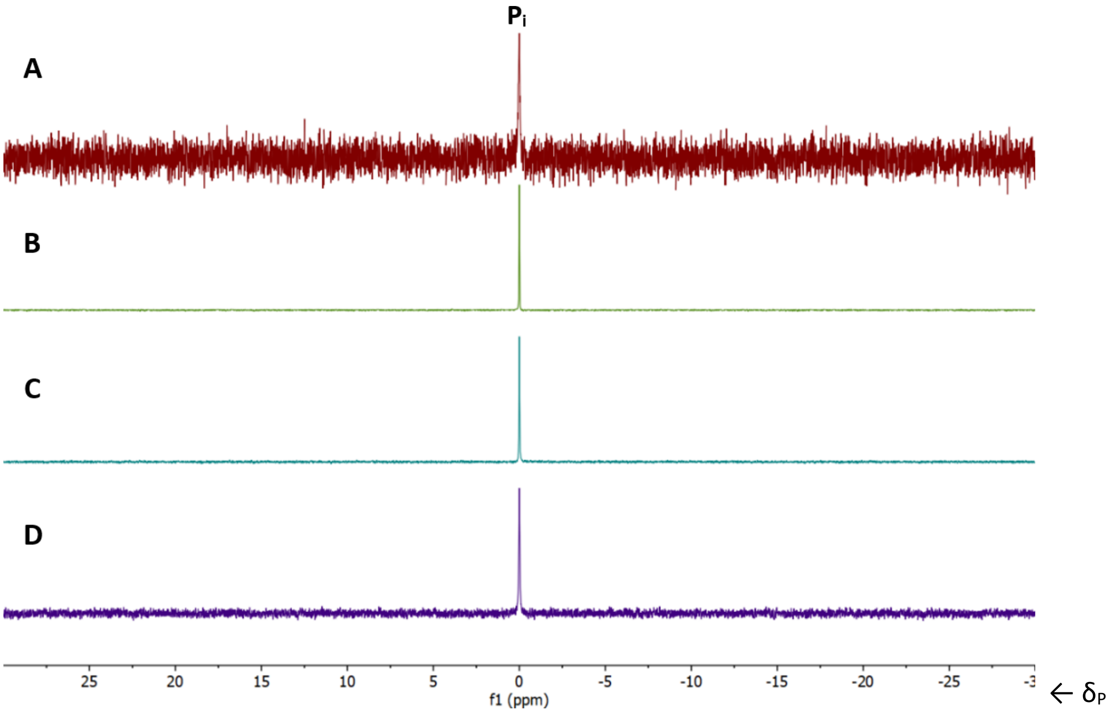

Supplementary Fig. 176. $^{31}P\{^1H\}$ NMR spectra (202.5 MHz) of crude mixtures of reactions with BTG (**7**) on a 0.5 mmol scale after 120 h of heating neat at 75 °C, then dissolved in DMSO-*d*6. On all shown spectra, we observe the presence of only one single resonance that belongs to $\mathbf{P_i}$, δ_{Pi} = 0.00 ppm:
A – reaction **7**:$\mathbf{P_i}$ (1:1); **B** – **7**:**2a**:$\mathbf{P_i}$ (1:1:1); **C** – **7**:**1**:$\mathbf{P_i}$ (1:1:1); **D** – **7**:**3a**:$\mathbf{P_i}$ (1:1:1).

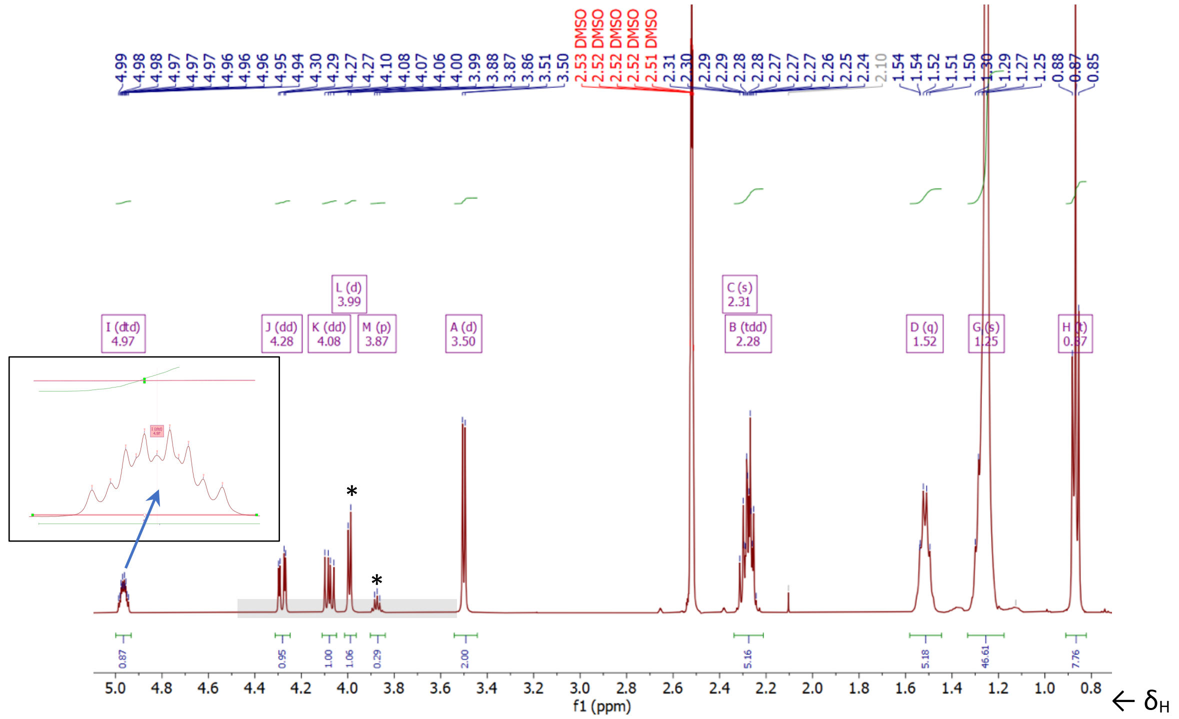

Supplementary Fig. 177. ^{1}H NMR spectrum (500 MHz) of crude reaction mixture with BTG — **7**:**1**:**cTMP** (1:1:1) on a 0.5 mmol scale after 120 h of heating neat at 115 °C, then dissolved in DMSO-*d6*. δ_H (ppm) = 5.45 (br *s*, 6H, OH, not shown), 4.97 (*dtd*, J = 10.7, 5.5, 3.0 Hz, 1H, C(2)H), 4.28 (*dd*, J = 12.0, 3.1 Hz, 1H, C(1)H_b), 4.08 (*dd*, J = 12.0, 7.2 Hz, 1H, C(1)H_a), 3.99 (*d**, J = 5.4 Hz, 2 x CH_2), 3.87 (*quint**, J = 5.4 Hz, CH), 3.50 (*d*, J = 5.6 Hz, 2H, C(3)H_2), 2.30-2.24 (*m*, 5H, C*H*$_2$COOR), 1.57-1.45 (*m*, 6H, C*H*$_2$$CH_2$COOR), 1.25 (br, 42H, CH_2), 0.86 (*t*, J = 6.9 Hz, 7H, CH_3).

The structural assignment of ^{1}H NMR resonances of the starting molecule is shown in Supplementary **Fig. 6**, new glyceryl resonances, accounting for about 22 % of all glyceryl peak areas, are marked with an asterisk*. The grey zone is shown in the ^{1}H-^{31}P HMBC spectrum (Supplementary **Fig. 178**). Out of the series of all reactions only in that of **BTG** with **cTMP** heated at 115 and 75 °C were observed small or trace amounts of organic products of phosphorylation: acyclic **BTGP** and 3-tridecanoylglyceryl-1,2-cyclic phosphate (**5cTGP**) resulting from the mono-deacylation of **BTG**(**P**).

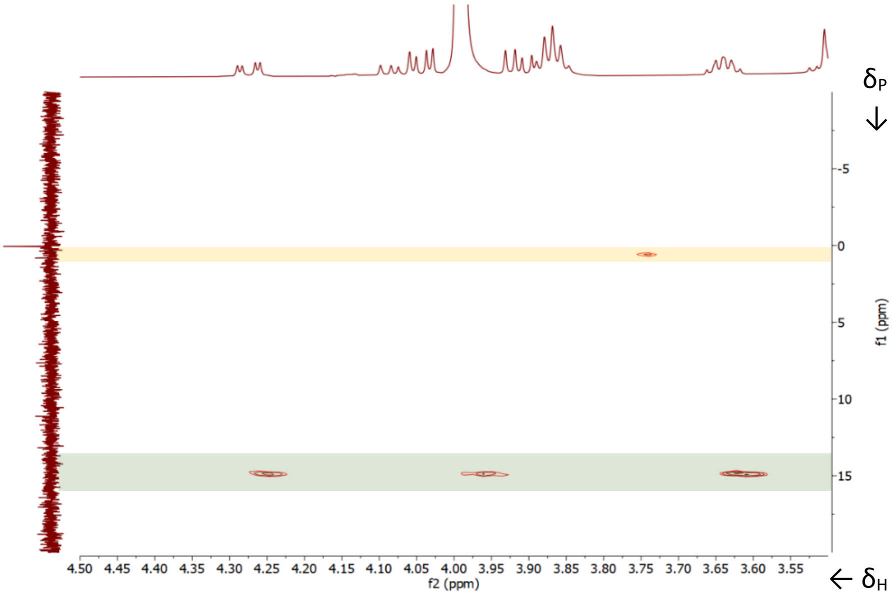

Supplementary Fig. 178. ^{1}H-^{31}P HMBC spectrum (500 MHz for ^{1}H [horizontal axis], 202.5 MHz for ^{31}P{^{1}H} [vertical axis]) of crude mixture with BTG **7**:**1**:**P**$_i$ (1:1:1) on a 0.5 mmol scale after 120 h of heating neat at 75 °C, then dissolved in DMSO-*d6*. Ochre (**BTGP**) and green (**5cTGP**) zones highlight organic products of phosphorylation.

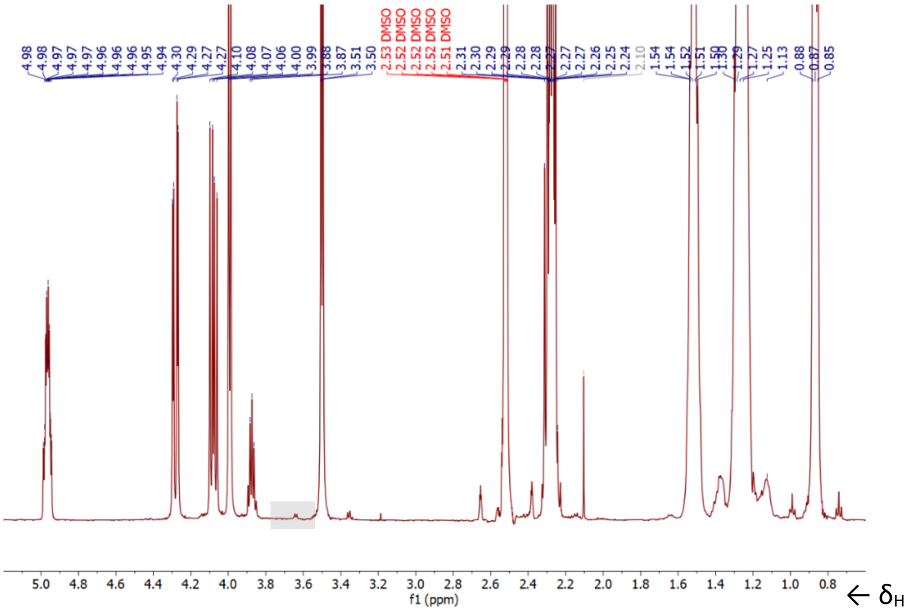

Supplementary Fig. 179. ^{1}H NMR spectrum (500 MHz) of crude reaction mixture with BTG **7**:**1**:**P**$_i$ (1:1:1) on a 0.5 mmol scale after 120 h of heating neat at 75 °C, then dissolved in DMSO-*d*6. The structural assignment of ^{1}H NMR resonances of the starting molecule is shown in Supplementary **Fig. 6**. The grey zone is an area of organic products of phosphorylation as observed in the ^{1}H-^{31}P HMBC spectrum (Supplementary **Fig. 180**).

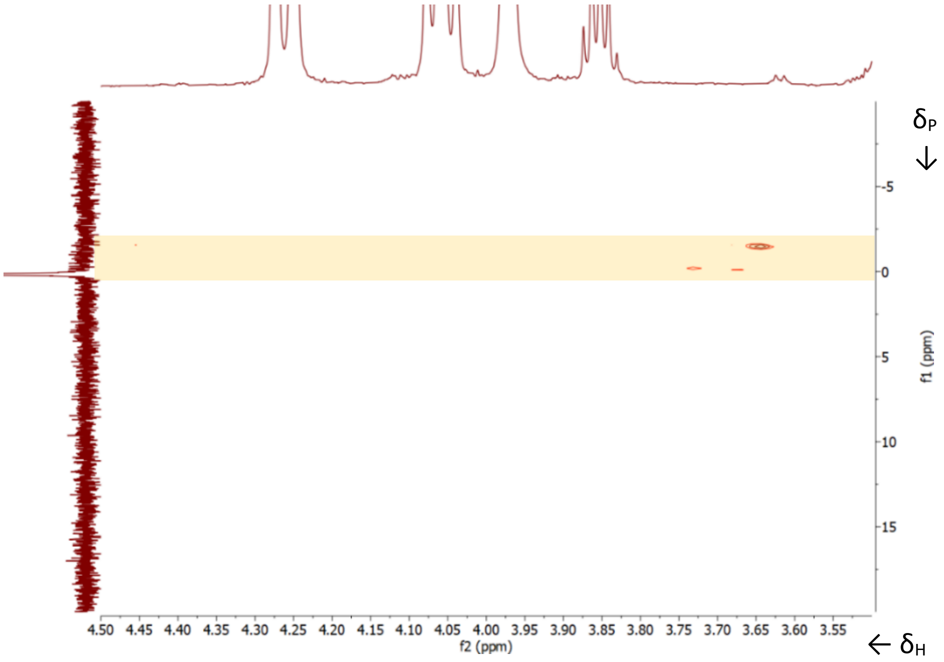

Supplementary Fig. 180. ^{1}H-^{31}P HMBC spectrum (500 MHz for ^{1}H [horizontal axis], 202.5 MHz for ^{31}P{^{1}H} [vertical axis]) of crude mixtures of reactions with BTG **7**:**1**:**P**$_i$ (1:1:1) 0.5 mmol scale reaction after 120 h of heating at 75 °C, then dissolved in DMSO-*d*6. The ^{31}P{^{1}H} NMR spectrum is presented in Supplementary **Fig. 176C.** Ochre zone highlights acyclic organic products of phosphorylation. Quantities of products are very low, consequently it is impossible to identify these traces in the ^{31}P{^{1}H} NMR spectrum.

Phosphorylated products are present in reaction mixtures in trace amounts, the signals have intensities similar to noise. The only significant signal in all ^{31}P{^{1}H} NMR spectra of reactions with **7** was δ_P = 0 ppm (**P**$_i$). However, we are able to see definitive traces of products by ^{1}H-^{31}P HMBC spectra. By cross correlation we identified product signals and integrated the corresponding peak areas (without signal broadening) in ^{31}P{^{1}H} NMR spectrum in accord to procedure described earlier (Supplementary **Fig. 181**, Supplementary **Table 56**).

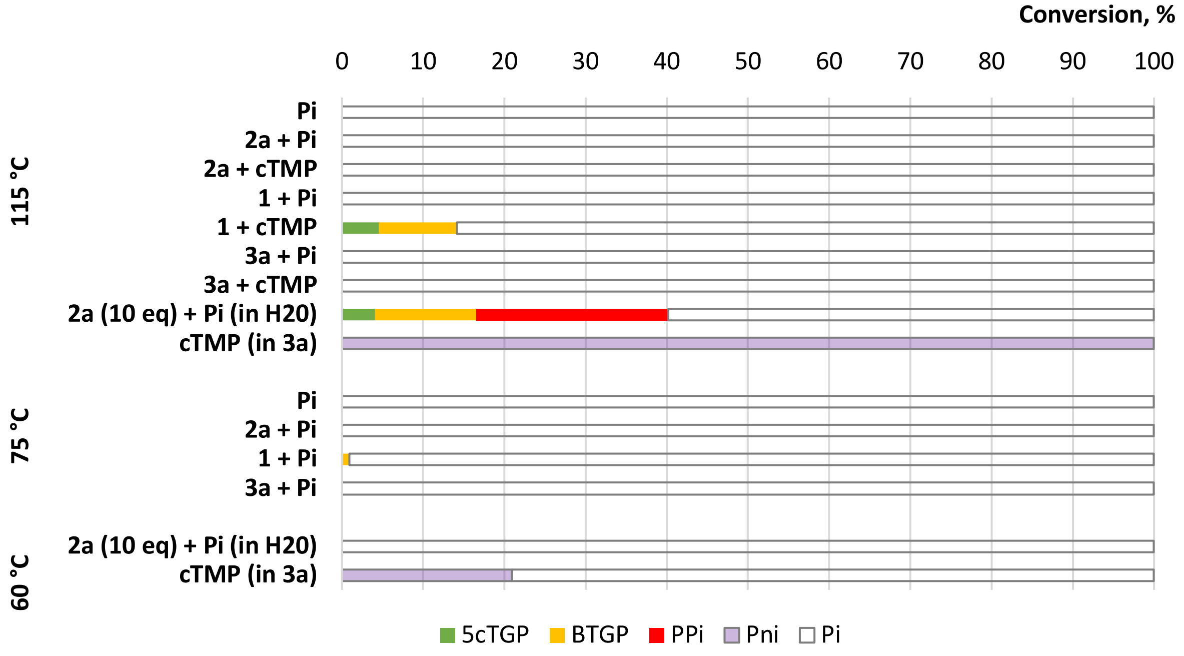

Supplementary Fig. 181. Summary of BTG (**7**) phosphorylations after 120 h of heating at 115°, 75° and 60 °C on a 0.5 mmol scale, without and with 1 ml H_2O (water evaporated eventually) or **3a** (0.5 eq with respect to starting molecule) as liquidisers. Organic products: **5cTGP** (3-tridecanoylglyceryl-1,2-cyclic phosphate), **BTGP** (BTG monophosphate). Percent values from signal integration of quantitative $^{31}P\{^{1}H\}$ NMR spectra taken in DMSO-*d6* at 40 °C to increase the solubility of the sample. Molar ratios of reaction compounds are **7** : cond. agent : phosphorous source are 1:1:1, except when mentioned otherwise (10 eq excess in several reactions with **2a**).

Supplementary Table 56. Data to Supplementary **Fig. 181**. Total conversion of initial amount of **cTMP** and $\mathbf{P_i}$ to phosphorylated organic products was calculated by subtracting inorganic compound integrals ($\mathbf{P_i}$, $\mathbf{PP_i}$, $\mathbf{P_{ni}}$) from the sum of all integrated $^{31}P\{^{1}H\}$ NMR peak areas. $\mathbf{P_{ni}}$ yield in reactions with **cTMP** is the signal of residual amount of **cTMP**. Molar ratios of reaction compounds are **7**:cond. agent:phosphorous source are 1:1:1 respectively, except if mentioned otherwise (10 eq excess in several reactions with **2a**).

Temp. °C	Cond. agent	Phosphorus source	Solvent/ Liquidiser	5cTGP %	BTGP %	Total con-version, %	PP_i, %	Pn_i, %	P_i, %
115	-	P_i	-	-	-	-	-	-	100.00
	**2a**	P_i	-	-	-	-	-	-	100.00
	**2a** (10 eq)	P_i	H_2O	4.09	12.46	16.55	23.66	-	59.79
	**1**	P_i	-	-	-	-	-	-	100.00
	**3a**	P_i	-	-	-	-	-	-	100.00
	**2a**	**cTMP**	-	-	-	-	-	-	100.00
	**1**	**cTMP**	-	4.57	9.55	14.12	-	-	85.88
	**3a**	**cTMP**	-	-	-	-	-	-	100.00
	-	**cTMP**	**3a**	-	-	-	-	100.00	-
75	-	P_i	-	-	-	-	-	-	100.00
	**2a**	P_i	-	-	-	-	-	-	100.00
	**1**	P_i	-	-	0.88	0.88	-	-	99.12
	**3a**	P_i	-	-	-	-	-	-	100.00
60	**2a** (10 eq)	P_i	H_2O	-	-	-	-	-	100.00
	-	**cTMP**	**3a**	-	-	-	-	20.91	79.09

7.8 Phosphorylation of 2,3-dioleoylglycerol: DOG (8)

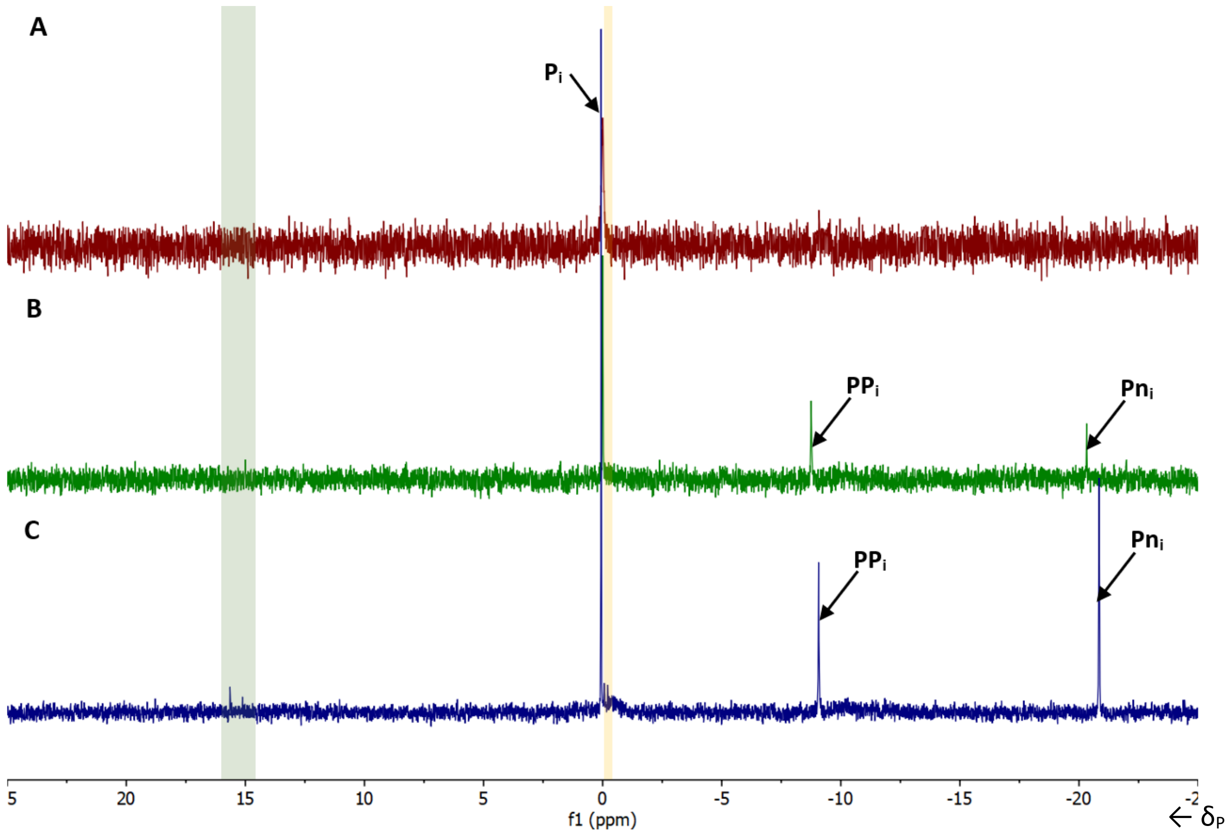

Supplementary Fig. 182. $^{31}P\{^1H\}$ NMR spectra (202.5 MHz) of reaction mixture with DOG **8**:**2a**:**P**$_i$ (1:[1/5/10]:1), on a 0.5 mmol scale after 120 h of heating at 115 °C, then dissolved in DMSO-*d*6.

A – 1 eq of **2a**; δ_P (ppm)= 0.00 (*s*, **P**$_i$).

B – 5 eq of **2a**; δ_P (ppm) = 15.01 (*s*, 5-membered ring cyclic MOG phosphate, **5cMOGP**, green zone); 0.00 (*s*, **P**$_i$); –8.78 (*s*, **PP**$_i$); –20.32 (*s*, **P**$_{ni}$).

C – 10 eq of **2a**; δ_P (ppm) = 15.58, 15.06 (2 x *s*, 5-membered ring cyclic MOG phosphate, **5cMOGP**, green zone); 0.00 (*s*, **P**$_i$); –0.13, –0.27 (2 x *s*, acyclic phosphates, **DOGP**, ochre zone), –9.13 (*s*, **PP**$_i$); –20.91 (*s*, **P**$_{ni}$).

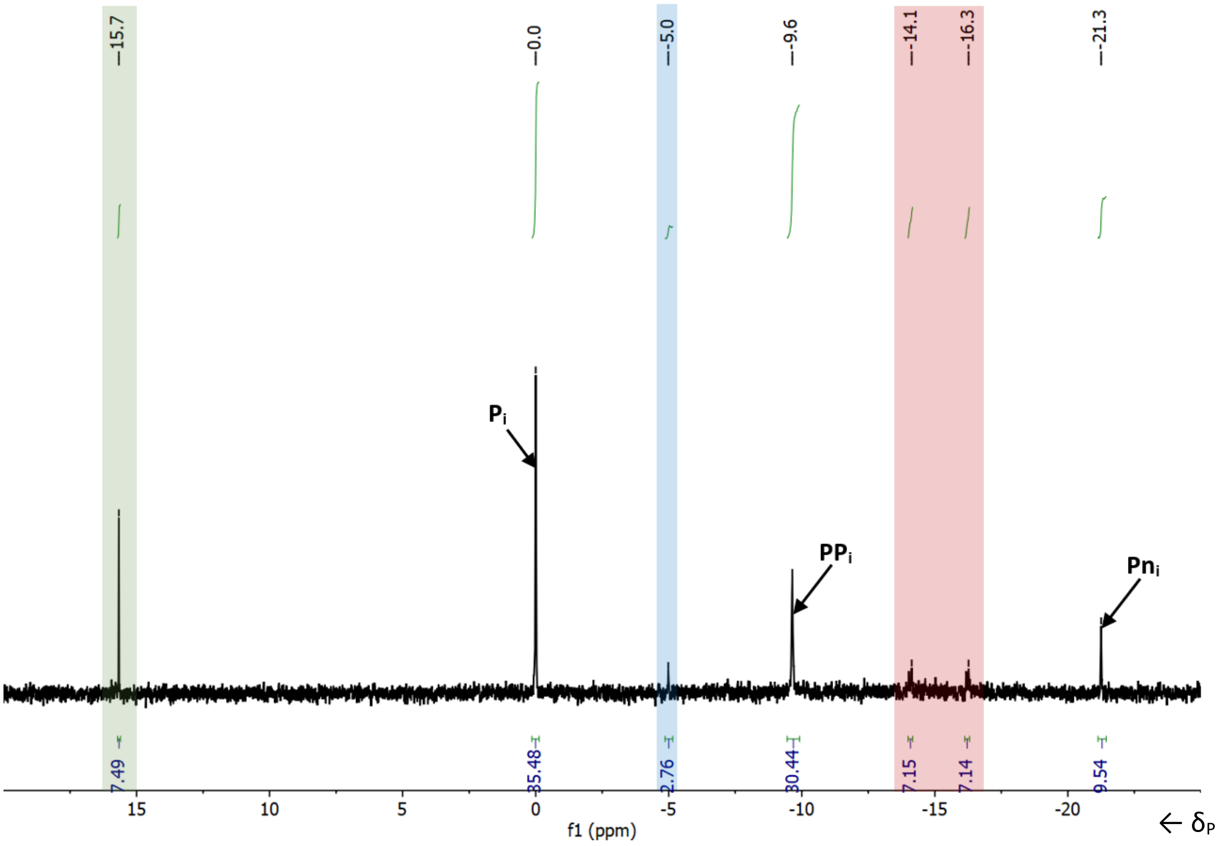

Supplementary Fig. 183. $^{31}P\{^1H\}$ NMR spectrum (202.5 MHz) of reaction mixture **8**:**1**:**[$^{18}O_4$]P$_i$** (1:1:1), on a 0.5 mmol scale after 120 h of heating at 115 °C with initially added D_2O (0.5 ml) + EtOH (0.5 ml) (water and ethanol evaporated eventually), then dissolved in DMSO-*d*6. δ_P (ppm) = 15.70 (*s*, 3-mono-oleoylglyceryl-1,2-cyclic phosphate, **5cMOGP**, green); 0.00 (*s*, **P$_i$**); –5.03 (*s*, 2-mono-oleoylglyceryl-1,3-cyclic phosphate, **6cMOGP**, blue); –9.57 (*s*, **PP$_i$**); –14.13, –16.27 (2 x *d*, DOG diphosphate, **DOGPP**, pink), –21.31 (*s*, **P$_{ni}$**).

The presence of 5-membered and 6-membered ring cyclic phosphates when cyanamide (**1**) was the condensing agent (Supplementary **Fig. 183**) can be explained in two ways, either as a result of the full deacylation to give glycerol that was phosphorylated to give **5cGlP** and **6cGlP**, or else (being the preferred option), the deacylation of the central oleoyl moiety to give after phosphorylation 3-mono-oleoylglyceryl-1,2-cyclic phosphate (5-membered ring cyclic phosphate, **5cMOGP**) and, respectively, the deacylation of the terminal oleoyl moiety to give after phosphorylation 2-mono-oleoylglyceryl-1,3-cyclic phosphate (6-membered ring cyclic phosphate, **6cMOGP**).

Much the same as for BTG, only a large molar excess of urea (10 equivalents **2a**) or 1 equivalent cyanamide (**1**) allowed for significantly detectable (> 5 %) organic phosphorylation of DOG. Neither the presence of pyruvate (1 and 10 equiv. **16**) at 60 or 115 °C nor of phosphoenolpyruvate (1 equiv. **16b**) as a potential phosphate relay compound at 60 °C could change this outcome (Supplementary **Fig. 184**).

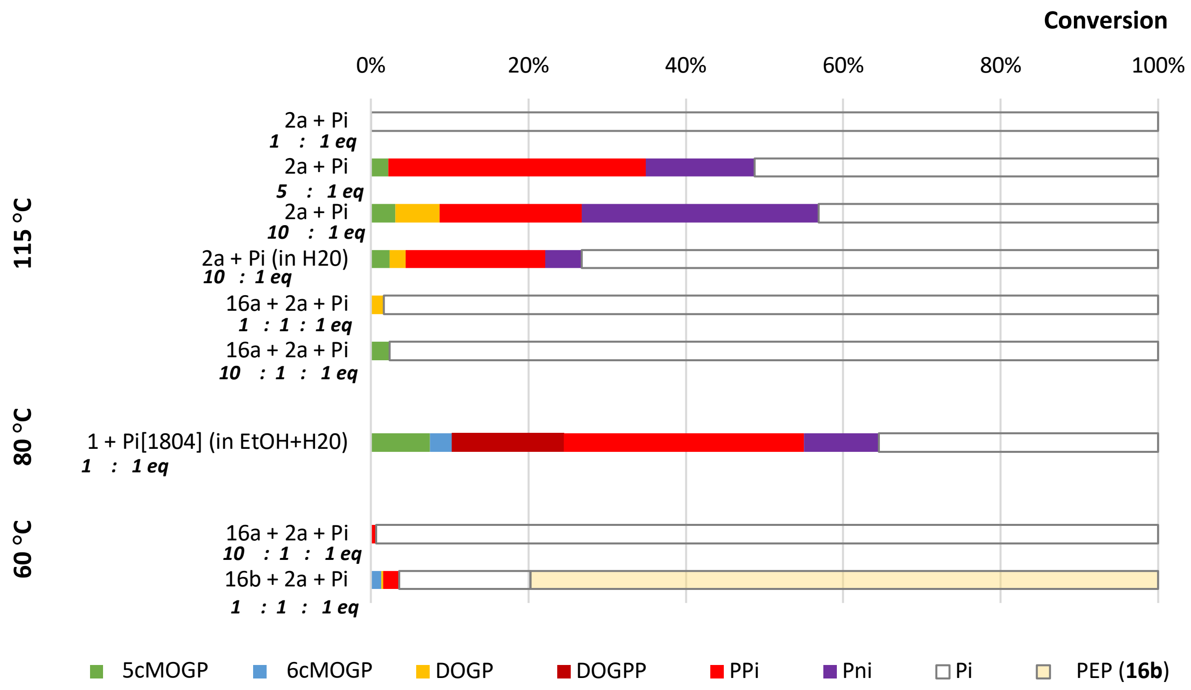

Supplementary Fig. 184. Summary of DOG (**8**) phosphorylation products after 120 h of heating at 115°, 80° and 60 °C on a 0.5 mmol scale, without solvent + **2a** and with 1 ml H_2O/EtOH + **1** (water and ethanol evaporated eventually). In the last row, added PEP (**16b**) was used to intensify the reaction where 16.7 and 79.7 % were not reacted $\mathbf{P_i}$ and **16b**, respectively. Organic products: **5cMOGP** (5-membered ring cyclic MOG phosphates), **6cMOGP** (6-membered ring cyclic MOG phosphates), **DOGP** (DOG monophosphate), **DOGPP** (DOG diphosphate). Percent values from signal integration of quantitative $^{31}P\{^1H\}$ NMR spectra taken in DMSO-*d*6. Molar ratios of reaction compounds are shown on the figure legend.

Supplementary Table 57. Data to Supplementary **Fig. 184**. Total conversion of initial amount of $\mathbf{P_i}$ to phosphorylated organic products was calculated by subtracting inorganic compound integrals ($\mathbf{P_i}$, $\mathbf{PP_i}$, $\mathbf{P_{ni}}$) from the sum of all integrated $^{31}P\{^1H\}$ NMR peaks.

**Temp., °C**	**Cond. agent**	**Phosphorus source** (1 eq)	**Liquidiser or phosphate transfer reagent**	**5cMOGP, %**	**6cMOGP, %**	**DOGP, %**	**DOGPP, %**	**PP_i %**	**P_{ni} %**	**P_i, %**	**Total conversion, %**
**115**	**2a** (1eq)	$\mathbf{P_i}$	-	-	-	-	-	-	-	100.00	-
**115**	**2a** (5 eq)	$\mathbf{P_i}$	-	2.20	-	-	-	32.75	13.80	51.25	2.20
**115**	**2a** (10 eq)	$\mathbf{P_i}$	-	3.09	-	5.60	-	18.07	30.11	43.13	8.69
**115**	**2a** (10 eq)	$\mathbf{P_i}$	H_2O	2.41	-	2.03	-	17.70	4.62	73.24	4.44
**115**	**2a** (1 eq)	$\mathbf{P_i}$	**16** (1 eq)	-	-	1.64	-	-	-	98.36	1.64
**115**	**2a** (1 eq)	$\mathbf{P_i}$	**16** (10 eq)	2.42	-	-	-	-	-	97.58	2.42
**80**	**1** (1 eq)	$\mathbf{[^{18}O_4]P_i}$	H_2O+EtOH	7.49	2.76	-	14.29	30.44	9.54	35.48	24.54
**60**	**2a** (1 eq)	$\mathbf{P_i}$	**16** (10 eq)	-	-	-	-	0.65	-	99.35	-
**60**	**2a** (1 eq)	$\mathbf{P_i}$ + **16b**	-	-	1.35	0.25	-	1.99	-	16.72<br>79.69	1.60

7.9 Phosphorylation of dodecan-1-ol (9)

In a preliminary experiment we tried to phosphorylate dodecan-1-ol (**9**) in the conditions reported by Powner et al.[39] and Albertsen et al.[40] that we refer to as ‘wet-to-dryness conditions’. The reaction scales were 0.01 mol with respect to the alcohol: urea 10 eq, alcohol 1 eq, $NH_4^+H_2PO_4^-$ 1 eq, suspended or dissolved in distilled water as 1 M solution (with respect to urea) and heated at 30 °C for 48 h then 100 °C for 48 h until complete dryness of the crude mixture was reached. ^{1}H NMR spectroscopy of the crude mixture obtained by repeating the synthesis (dodecan-1-ol replaced decan-1-ol) showed that a small amount of dodecyl phosphate formed and no traces of dodecyl carbamate were observed. We analysed this mixture also by ^{1}H, ^{13}C and ^{31}P{^{1}H} NMR spectroscopy (Supplementary **Fig. 185**). Only ^{31}P{^{1}H} analysis showed that *O*-dodecyl carbamate (**9d**) and *bis*-dodecyl diphosphate (**9c**) formed, however we were unable to precisely determine the yields from ^{1}H NMR that appeared as overestimated 3.6 %.

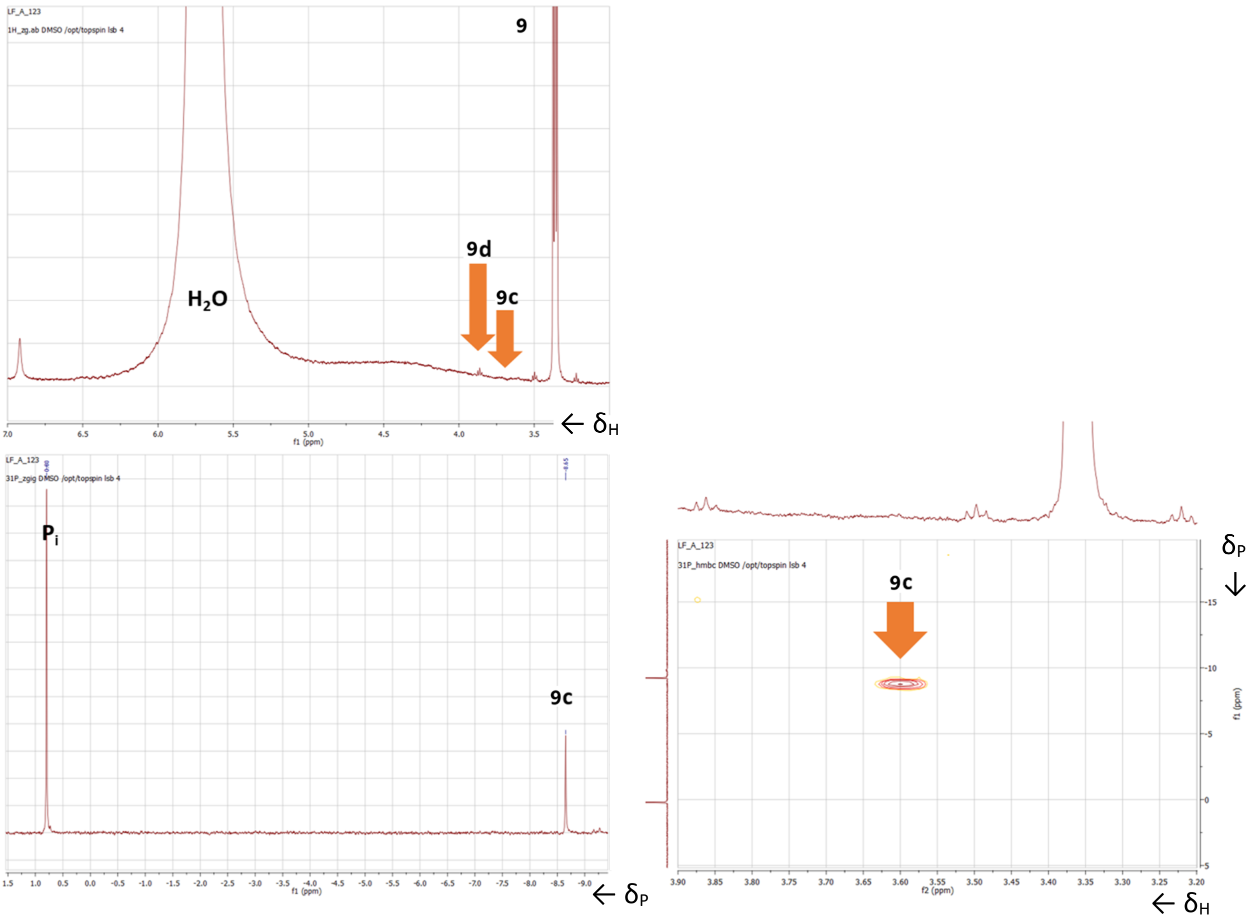

Supplementary Fig. 185. ^{1}H NMR (500 MHz), ^{31}P{^{1}H} NMR and ^{1}H-^{31}P HMBC spectra from reactions carried out in ‘wet-to-dryness’ conditions. The crude mixture contained *bis*-dodecyl pyrophosphate (**9c**) and *O*-dodecyl carbamate (**9d**) in barely detectable amounts. This experiment was carried out by using urea on a 0.1 mol scale.

We have distinguished **9b** from **9** and all the other identified compounds by spiking experiments using a commercial sample of dodecyl phosphate monosodium salt (not shown). We explored the phosphorylation of **9** in the absence of water in so-called ‘neat conditions’: urea or any other condensing agent 1 eq., alcohol 1 eq., **P**$_i$-1 eq. T= 80-130°C. 5 days (120 hrs) at a reaction scale of 0.1 mol, 0.033 mol or 0.01 mol. In addition, we tested the so-called ‘formamide conditions’ used by Powner[39] (adenosine) or Albertsen[40] (glycerol). Condition 1 (for 0.6 mmol scale of alcohol): urea 10 eq., alcohol 1 eq., $NH_4^+H_2PO_4^-$ (1 eq. **P**$_i$-1), formamide 0.6 ml. Condition 2 (for 0.1 mol scale): urea 1 eq., alcohol 1 eq., **P**$_i$-1 eq., formamide 1 or 2 eq. One experiment was carried out in the presence of L-alanine as co-reactant in a competition reaction, the found products are listed in Supplementary **Fig. 186**.

7.9.1 NMR and HRMS analyses of urea- and cyanamide-assisted reaction mixtures containing dodecan-1-ol (9), its phosphate esters and other amphiphilic products

The formation of dodecyl phosphate (**9b**) was monitored at four different temperatures 80, 100, 115 and 130°C in the presence of ammonium dihydrogenphosphate (**P**$_i$-1) or sodium dihydrogenphosphate (**P**$_i$-2) and cyanamide (**1**) or urea (**2a**) in molar ratios 1:1:1 for **9** : (**1** or **2a**) : **P**$_i$. The structures of all the molecules found, despite the different reaction conditions, are all shown in Supplementary **Fig. 186**. We based the quantification of **9b** and other dodecyl products in the untreated crude reaction mixtures by integrating the ^{1}H NMR peak areas of the methylene (CH_2) signals in alpha-position of the phosphate ester group (δ_H = 3.65 ppm) with respect to that of the methylene in alpha at the hydroxyl group of the starting material (**9**, δ_H = 3.36 ppm) together with those of diphosphate **9c** (δ_H = 3.61 ppm) and carbamate **9d** (δ_H = 3.85 ppm) and other formed compounds (**9e**-**f**).

As an example, we report here the analysis of a crude mixture obtained by heating at 115°C for 5 days a mixture containing in a 1:1:1 mol-ratio **9**, [^{13}C]urea (**2c**) and NaH_2PO_4 (**P**$_i$-2). Analysis of other mixtures gave similar or identical results. A first analysis showed that the major compound formed in the mixtures corresponded to dodecyl carbamate (**9d**, δ_H = 3.85 ppm) accompanied by minor amounts of other two compounds further identified as **9e** and **9f**. Both **9e** and **9f** possess methylene groups resonating in a close ppm range and their overlap give a multiplet (*m*) signal at δ_H = 4.06-3.99 ppm. The phosphorylation product **9b** was present in minor amounts (0.5-0.8 %, DMSO-*d*6). The presence of **9b** was further confirmed by ^{31}P{^{1}H} NMR and ^{1}H-^{31}P HMBC (δ_P = –0.63 ppm) together with diphosphate **9c** (δ_P = –8.65 ppm). ^{13}C NMR spectroscopy was used to confirm the presence of residual **9** (CH_2, δ_C = 61.2 ppm) and the formation of **9b** (CH_2, δ_C = 63.7 ppm) together with the characteristic signal of the majority product **9d** (carbamate signals δ_C = 63.3 (CH_2) and 157.3 ppm (C=O), respectively). In particular, the use of labelled [$^{15}N_2$]urea (**2b**) (not shown) and [^{13}C]urea (**2c**) (Supplementary **Fig. 187**-Supplementary **Fig. 189**) confirmed that some of the amphiphiles resulted as the product of the reaction between dodecan-1-ol and isocyanic acid or cyanate (**9d**-**9f**) and the presence of other compounds such as an oxidation product (from atmospheric oxygen) **9g.** The presence of all the mentioned compounds in the mixtures was confirmed by UHPLC-HRMS in positive-ion mode (Supplementary **Fig. 192** and Supplementary **Table 58**) whereas the presence of **9b** was confirmed by negative-ion mode MS (Supplementary **Fig. 192**-peak 4b).

Molecules found in crude mixtures

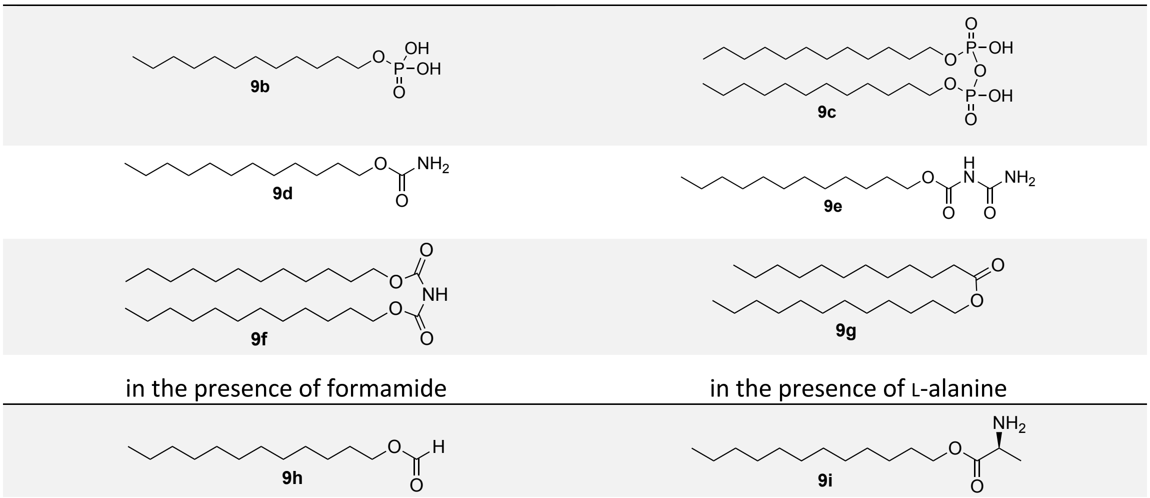

Supplementary Fig. 186. The chemical structure of some of the identified molecules present in the crude mixture obtained by heating dodecan-1-ol (**9**) in neat conditions in the presence of phosphate salts and urea or other condensing agents. Other not well identified by-products are present in the mixtures (cf. UHPLC-HRMS analysis in Supplementary **Fig. 192**). UHPLC conditions for the analysis: UHPLC (Thermo-Fisher scientific U3000) coupled with a high-resolution high-impact quadrupole mass (Bruker, Impact II), Agilent C8 column (50.0 x 2.1 x 0.27 mm), Flow 800 µL/min; mobile phase A) 0.1% formic acid in ultra-pure milliQ water, B) CH_3CN/MeOH (1:1 *v*/*v*) + 0.1% formic acid.

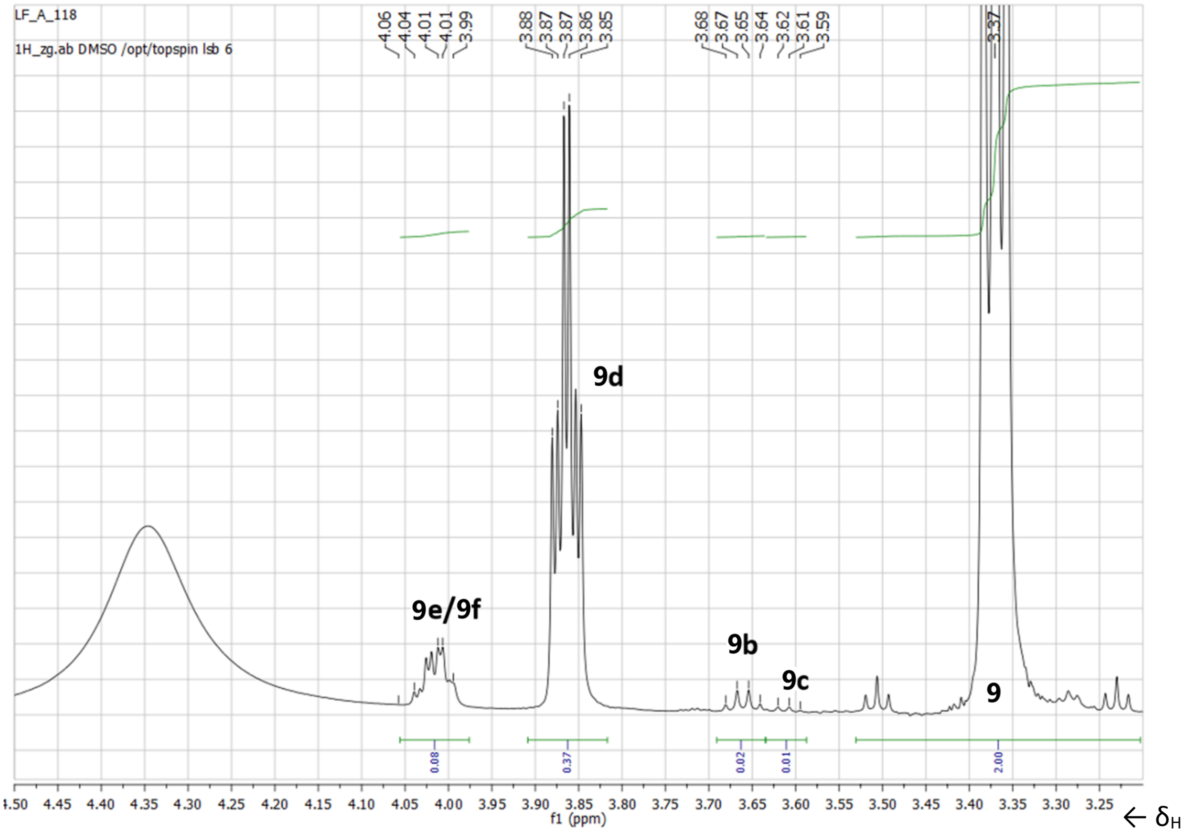

Supplementary Fig. 187. ^{1}H NMR (500 MHz) of the crude mixture from dodecan-1-ol (**9**):[^{13}C]urea (**2c**):NaH_2PO_4 (**P$_i$**-2) in 1:1:1 ratio at 115°C for 120 h. The mixture contains 1-dodecyl phosphate (**9b**), *bis*-dodecyl diphosphate (**9c**), dodecyl carbamate (**9d**) and the other by-products (**9e**-**9f**) together with unreacted dodecan-1-ol (**9**). In this case, the conversion of **9** to **9b** was 0.9 % and to **9d** it was 14.9 %.

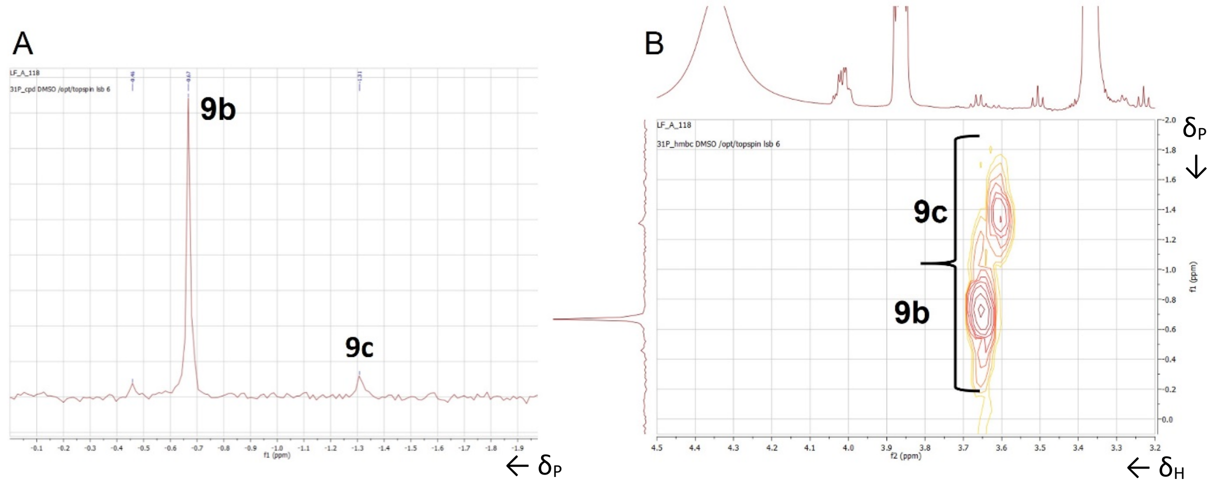

Supplementary Fig. 188. $^{31}P\{^{1}H\}$ NMR (**A**); ^{1}H-^{31}P HMBC (**B**) spectra of the crude mixture from **9**:**2c**:**P$_i$**-2 (1:1:1) at 115°C for 120 h. The mixture contains 1-dodecyl phosphate (**9b**), didodecyl pyrophosphate (**9c**) dodecyl carbamate (**9d**) and the other by-products (**9e**-**9f**) together with unreacted dodecan-1-ol (**9**).

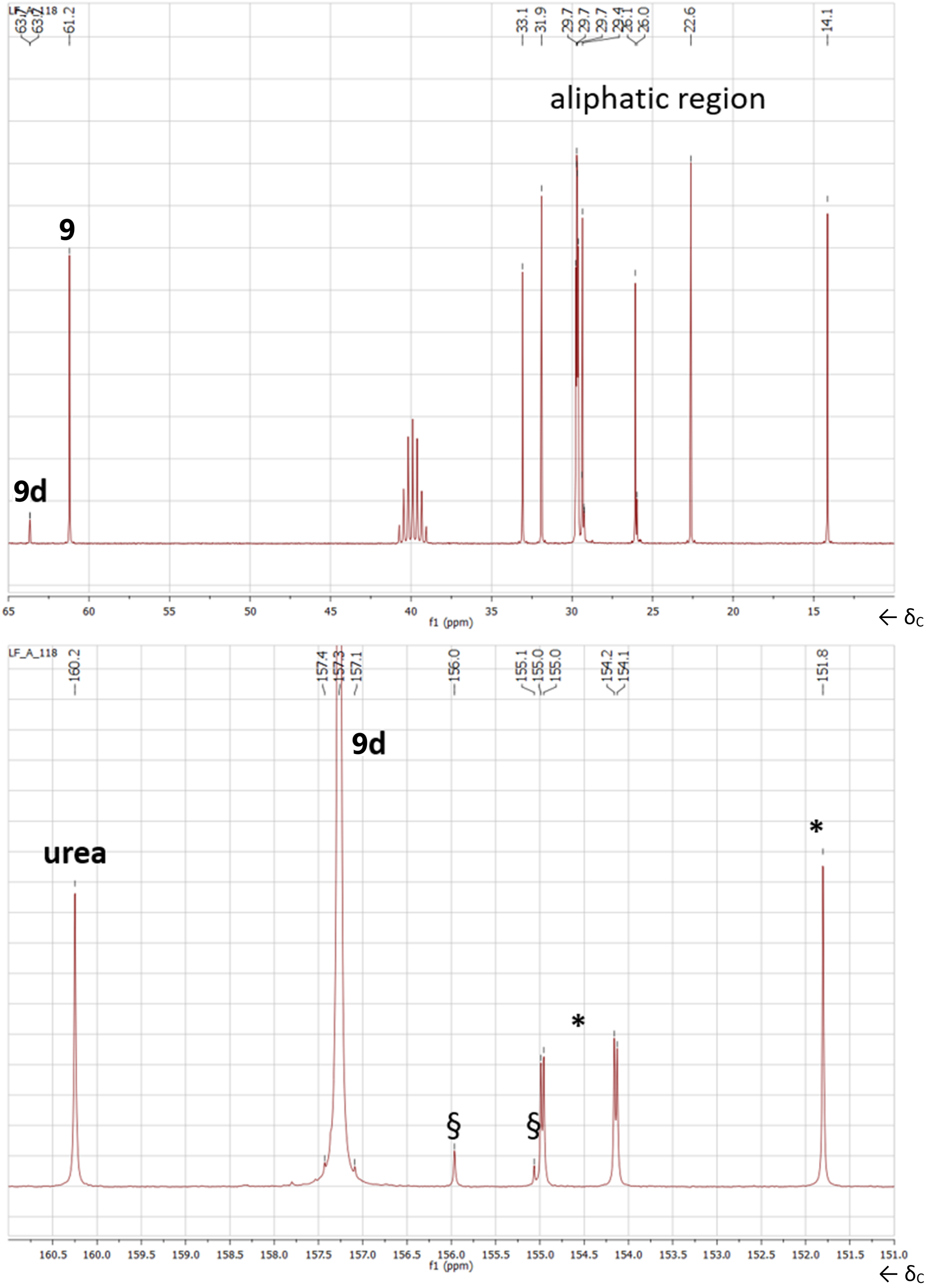

Supplementary Fig. 189. ^{13}C NMR (100 MHz) spectra and 161-151 ppm zoom of the crude mixture from dodecan-1-ol: [^{13}C]urea:NaH_2PO_4 (**9**:**2c**:$\mathbf{P}_i$-1) in 1:1:1 ratio at 115°C for 120 h. The mixture contains 1-dodecyl phosphate (**9b**), *bis*-dodecyl diphosphate (**9c**), dodecyl carbamate (**9d**) and the other by-products (**9e**-**9f**, marked * and §) together with unreacted **9**. In this case, the conversion of **9** to **9b** was 0.9 % and to **9d** it was 14.9 %.

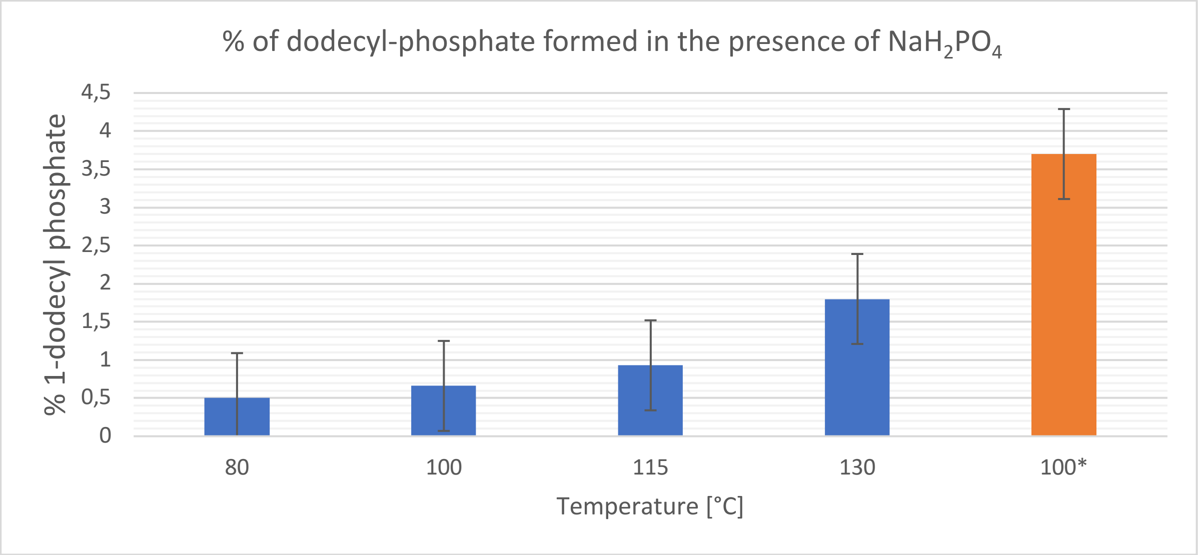

Supplementary Fig. 190. Graphical representation of the conversion of dodecan-1-ol (**9**) to 1-dodecyl phosphate (**9b**) as a function of the temperature under 'neat conditions'. Reactions were carried out for 120 h on a 0.1 mol scale under continuous nitrogen flow. (orange bar): Result obtained using 'wet-to-dryness conditions' on a 0.1 mol scale reaction at 100 °C (cf. Supplementary **Fig. 185**). Error margins represent the estimated uncertainty of peak integration. Reaction conditions detailed on page 194.

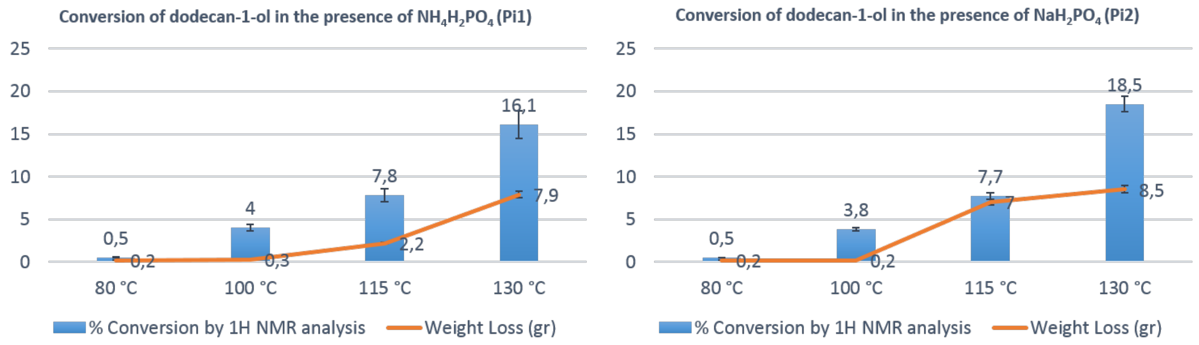

Supplementary Fig. 191. Graphical representation of the conversion of dodecan-1-ol (**9**) to dodecyl carbamate (**9d**) as a function of two different $\mathbf{P_i}$ salts (ammonium and sodium) and the temperature. Reactions were carried out for 120 h on a 0.1 mol scale under continuous nitrogen flow. The orange line represents the weight loss (Δw) in grammes measured as the difference of weight of the whole mixture at the beginning of the reaction and after 120 h.

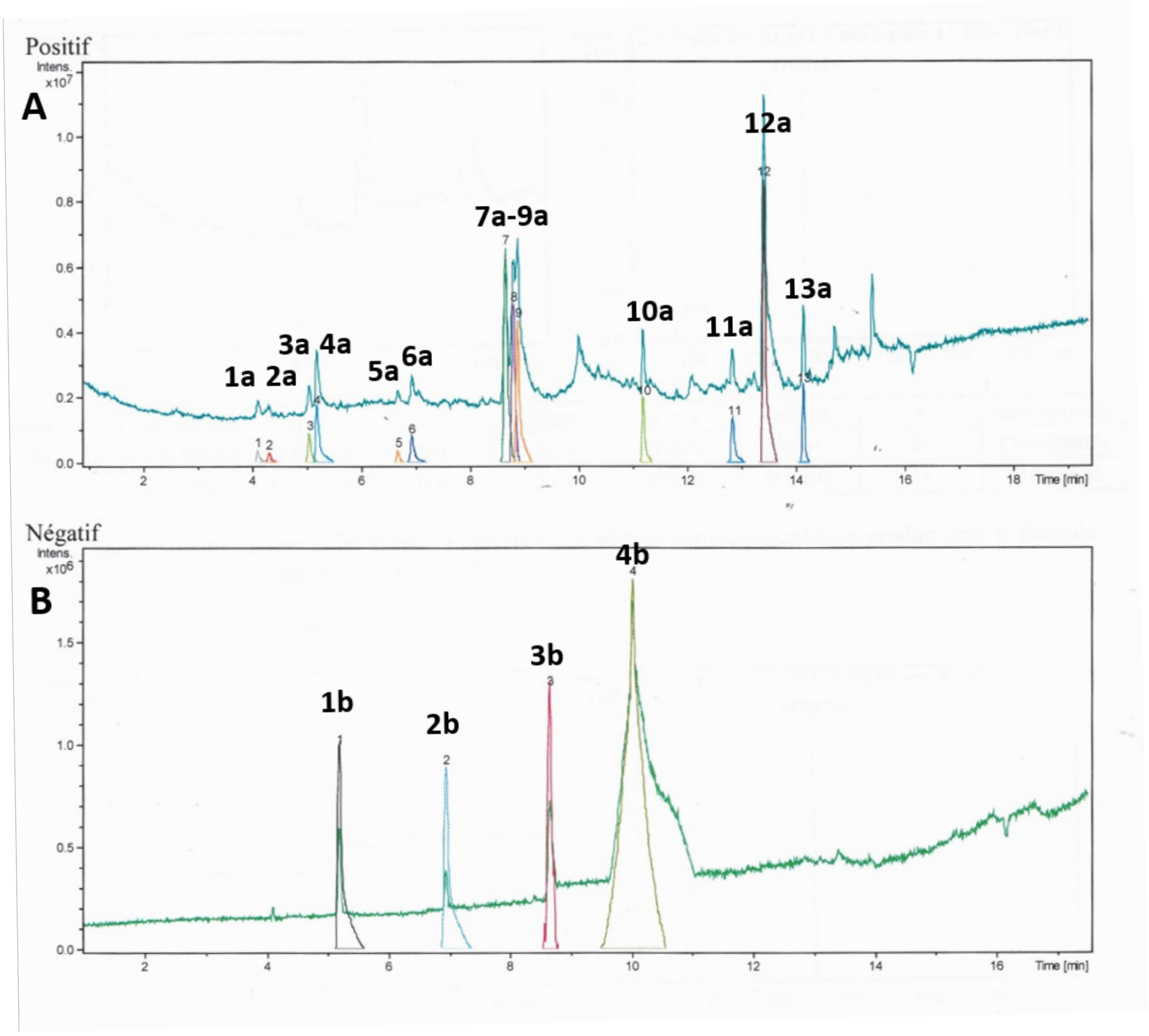

Supplementary Fig. 192. Total ion UHPLC-MS chromatograms (**A** – positive ion mode, **B** – negative ion mode, continuous total ion intensity, coloured peaks: mass-extracted ions) of the mixture obtained by heating neat **9**:**P**$_i$-2:**2a** (1:1:1) for 120 h at 115°C. The analysis of peaks is reported in Supplementary **Table 58**. The UHPLC parameters are detailed in the legend of Supplementary **Fig. 186**.

Supplementary Table 58. UPLC-HRMS data of one of the obtained crude mixtures: **9**:**P**$_i$-2:**2a** (1:1:1), 0.1 mol scale, 115°C 120 h. Peaks with bold typeset *m/z*, molecular formula and retention time have been identified as known compounds.

Peak n° (positive ion mode)	*m/z*	Molecular formula	Retention time	Compound
1a	276,2169	$C_{14}H_{29}NO_4$	4.1 min	-
2a	252,2054	$C_{12}H_{27}N_3NaO$	4.4 min	-
3a	209,1999	$C_{11}H_{26}N_2Na$	5.1 min	-
4a	264,2070	$C_{15}H_{26}N_3O$	5.2 min	-
5a	-[a]	-[a]	6.3 min	-
6a	257,2219	$C_{14}H_{29}N_3O_2$	7.0 min	-
7a	373,4043	$C_{24}H_{52}O_2$	8.7 min	-
**8a**	**230,2214**	$\mathbf{C_{13}H_{27}NO_2}$	**8.8 min**	**9d**
**9a**	**273,2174**	$\mathbf{C_{14}H_{28}N_2O_3}$	**8.9 min**	**9e**
10a	450,4054	$C_{27}H_{52}N_3O_2$	11.2 min	**-**
11a	441.4057	$C_{26}H_{52}N_2O_3$	12.9 min	-
**12a**	**442,3882**	$\mathbf{C_{26}H_{51}NO_4}$	**13.4 min**	**9f**
**13a**	**369,3716**	$\mathbf{C_{24}H_{48}O_2}$	**14.2 min**	**9g**
**Peak n° (negative mode)**	***m/z***	**Molecular formula**	**Retention time**	**Compound**
1b	264.2070	$C_{15}H_{23}N_3O$	5.2 min	-
2b	-[a]	-	6.7 min	-
3b	199,1689	$C_{10}H_{22}N_3O$	8.7 min	-
**4b**	**265,1565**	$\mathbf{C_{12}H_{27}O_4P}$	**10.0 min**	**9b**

[a] no molecular formula identified

In addition, other experiments carried out in similar conditions showed that the conversion of dodecan-1-ol (**9**) into 1-dodecyl phosphate (**9b**) is poor even when other phosphorylating agents were used together with urea. All reactions were carried out at the temperature of 115°C (Supplementary **Table 59**, entries 1-7). When formamide (Supplementary **Table 59**, entries 1-3) was used, the formation of 1-dodecyl carbamate (**9d**) was observed, the formation of **9b** was not detectable and, expectedly, the dominant presence of 1-dodecyl formate (**9h**) was detected instead. Surprisingly, the use of cyanamide (**1**), a well-known hydrolytic reservoir of urea increased the conversion of **9** to **9b** (9.1 %). In these conditions, the conversions of **9** to **9d** and **9e**/**9f** were around 11.8 and 11.1 %, respectively. We have previously observed that, when **1** was solubilised in water in the presence of 0.1 M $NH_4H_2PO_4$, its hydrolysis to urea was fast (< 12 h, monitored by ^{13}C NMR). However, when it was heated at 80 °C or higher the conversion was to cyanoguanidine and melamine in different ratios. No traces of melamine or cyanoguanidine were present in this sample, whereas urea (**2a**, δ_C = 162.9 ppm) was present together with residual **1** (δ_C = 118 ppm). In fact, cyanoguanidine signals (δ_C = 120 and 163.3 ppm) can overlap with those of **2a** and **1**, the integration of those two does not have the required 1:1 ratio. Peaks between δ_C = 158.1 ppm and 154.7 ppm correspond to those listed in Supplementary **Fig. 187**. The aliphatic region and signals of residual dodecan-1-ol are in agreement with what was previously observed for the experiment with urea as condensing agent.

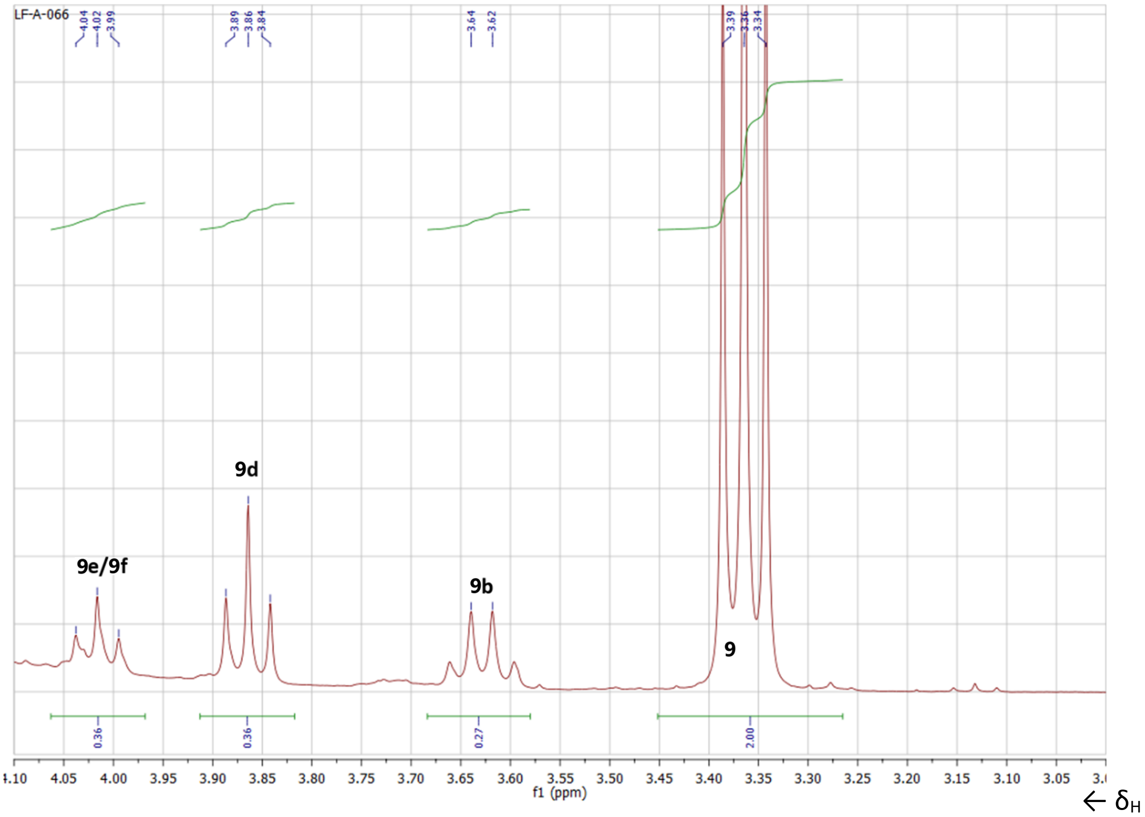

Supplementary Fig. 193.^{1}H NMR (300 MHz) of the crude mixture containing 1-dodecyl phosphate (**9b**), 1-dodecyl carbamate (**9d**) and the other by-products together with unreacted dodecan-1-ol (**9**) obtained by replacing urea with cyanamide (**1**) as condensing agent.

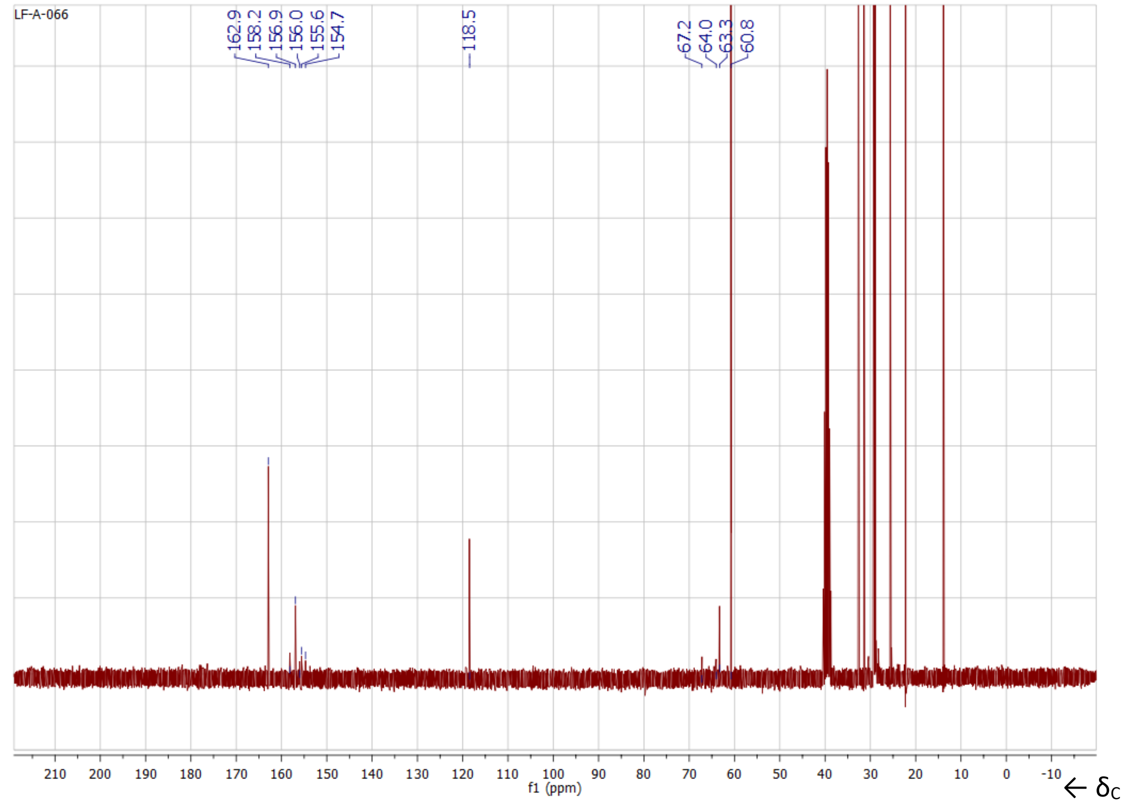

Supplementary Fig. 194.^{13}C NMR (100 MHz) of the crude mixture containing 1-dodecyl phosphate (**9b**) obtained by replacing urea with cyanamide (**1**) as condensing agent.

7.9.2 Other phosphate sources and reaction conditions for dodecan-1-ol

In contrast to the aforementioned results, when we replaced urea with sodium dicyanamide — used by Hargreaves, Mulvil & Deamer[41] for the synthesis of phospholipids under prebiotic conditions — no formation of **9b, 9c** or **9d** was observed (Supplementary **Table 53**, entry 5). The use of less soluble and alkaline phosphate sources such as naturally occurring hydroxyapatite or **cTMP** did not increase the yield of **9b**, favouring instead the formation of carbamate **9d** (entries 6-7) when compared with the reaction carried out in the presence of ammonium and sodium dihydrogenphosphates. Formamide (**3a**) directed the reaction efficiently towards dodecyl formate (**9h**) (entries 1-3). Cyanamide (**1**) favoured the formation of the carbamates **9d**, **9e** and **9f** (entry 4).

Supplementary Table 59. Product distribution after heating neat **9** at 115 °C with different condensing agents and **P**$_i$ sources.

Entry (115°C)	**P**$_i$ source	Urea **2a**	Co-condensing agent/alternative	% of found[a] **9b**	**9d**	**9e/9f**	**9h**
1	NaH_2PO_4	1 eq	**3a**, 1 eq[b]	None[a]	1.3	None[a]	9.7
2	NaH_2PO_4	1 eq	**3a**, 2 eq[b]	None[a]	1.2	None[a]	22.6
3	NaH_2PO_4	-	**3a**, 1 eq	None[a]	0.7	None[a]	70.3
4	NaH_2PO_4	-	**1**, 1 eq	9.1	11.8	11.1	-
5	NaH_2PO_4	-	Sodium dicyanamide[d], 1 eq	-	-	-	-
6	Apatite	1 eq	-	-	12.8	None[a]	-
7	**cTMP**	1 eq	-	0.8	11.7	None[a]	-

[a] ^{1}H NMR analysis and confirmed by independent HPLC analysis; [b] formamide (**3a**) used as liquidiser of neat **P**$_i$, **2a** and **9**; [d] Deamer used decanal/glycerol/sodium dicyanamide in their experiments[41].
- = no product detected, None = absent among the found product(s)

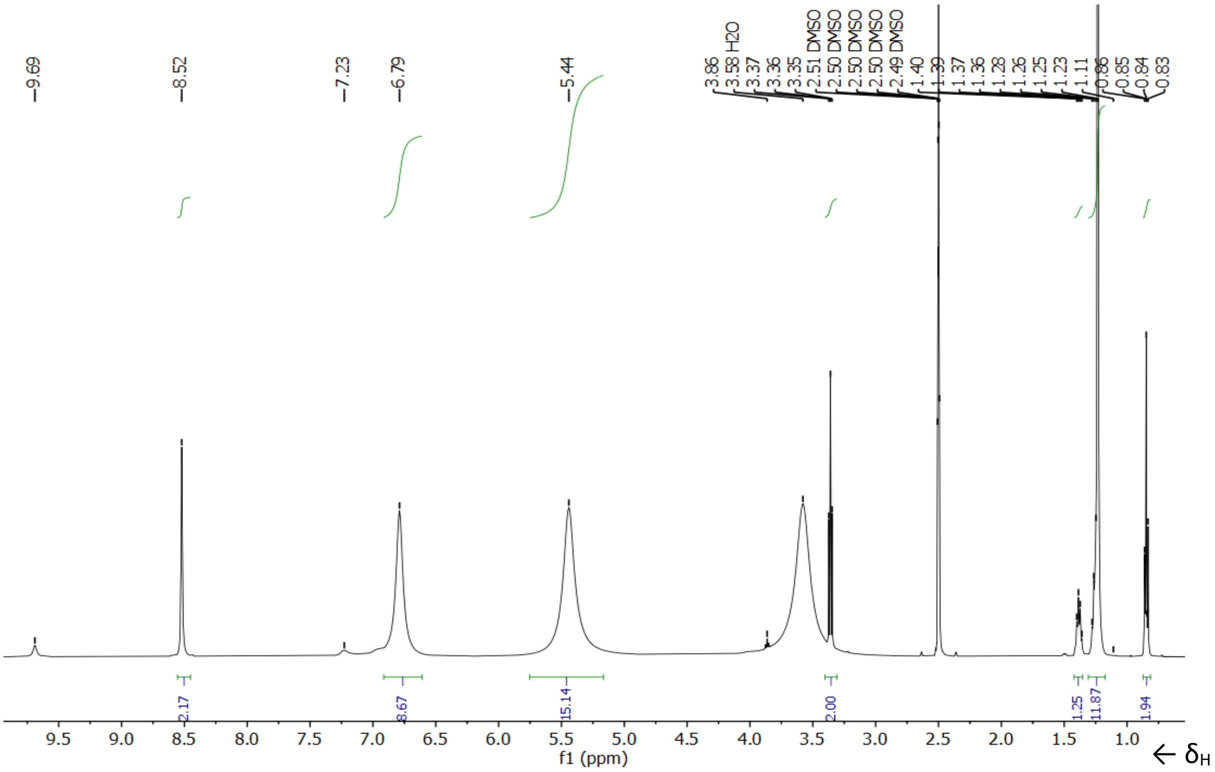

Supplementary Fig. 195. ^{1}H NMR spectrum (500 MHz, DMSO-*d6*) of crude mixture of **9**:**2a**:**P**$_i$-2 (1:10:1) 0.5 mmol scale in 1ml H_2O after 120 h of heating at 115 °C. δ_H (ppm) = 9.69 (*s*, RCONHCOR), 8.52 (*s*, 2H, ROCONH$_2$), 6.79 (br *s*, 9H, ROH), 5.44 (br *s*, CO(NH$_2$)$_2$), 3.58 (br *s*, H_2O), 3.36 (*t*, J = 6.6 Hz, 2H, $CH_3(CH_2)_9CH_2C\underline{H_2}OH$), 1.39 (*quint*, J = 6.9 Hz, 2H, $CH_3(CH_2)_9C\underline{H_2}CH_2OH$)), 1.20-1.35 (*m*, 18H, $CH_3(C\underline{H_2})_9CH_2CH_2OH$), 0.84 (*t*, J = 6.7 Hz, 3H, $C\underline{H_3}(CH_2)_9CH_2$ CH_2OH).

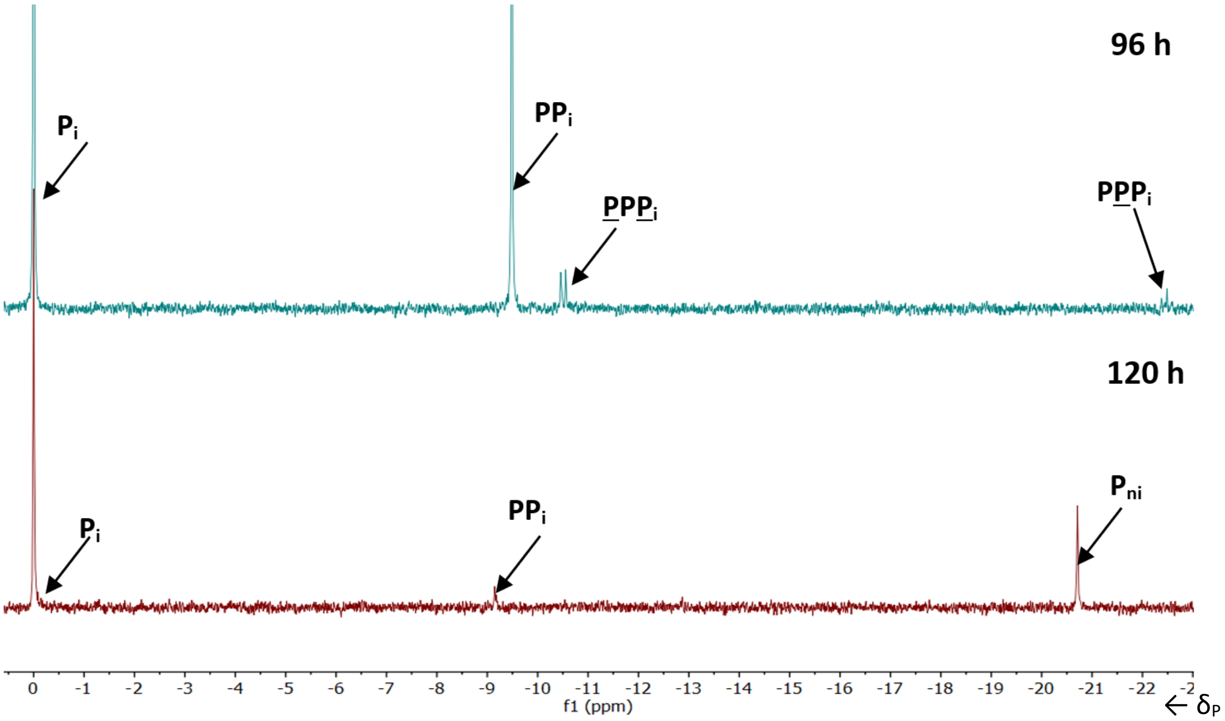

Supplementary Fig. 196. $^{31}P\{^1H\}$ NMR spectra (202.5 MHz, in DMSO-*d6*) of reaction mixture **9**:**2a**:$\mathbf{P_i}$ (1:10:1), 0.5 mmol scale at 115 °C in 1ml H_2O. **Upper spectrum** – after 96 h of heating; δ_P (ppm) = 0.00 (*s*, $\mathbf{P_i}$), –9.49 (*s*, $\mathbf{PP_i}$), –10.51 (*d*, J = 19.7 Hz $\mathbf{PPP_i}$), –22.48 (*t*, J = 19.7 Hz $\mathbf{PPP_i}$). **Lower spectrum** – after 120 h of heating; δ_P (ppm) = 0.00 (*s*, $\mathbf{P_i}$), –9.15 (*s*, $\mathbf{PP_i}$), –20.71 (*s*, $\mathbf{P_{ni}}$).

For the experiments with a large molar excess of urea under 'wet-to-dryness conditions' (reaction conditions detailed on page 194), that gave the ^1H NMR spectrum depicted in Supplementary **Fig. 195**, we performed ^1H-^{31}P HMBC analyses which did not show any cross-correlation (not shown). We concluded that all signals of the $^{31}P\{^1H\}$ NMR spectrum (Supplementary **Fig. 196**) were due to inorganic phosphates. After 96 h, we observed the (transient) appearance of ^{31}P doublet and triplet resonances, the area integration of which showed a 2:1 ratio being a signature for inorganic triphosphate $\mathbf{PPP_i}$, which could be also observed in 'wet-to-dryness conditions' involving $\mathbf{P_i}$, adenosine (**11**) and cyanamide (**1**) or urea (**2a**), see Supplementary **Fig. 112** and Supplementary **Fig. 113**.

To address the general lack of observed organic phosphorylation products, the reaction was supplemented with pyruvic acid (1 and 10 equivalents **16**). This addition was hypothesised to serve as an alternative source or activator for the phosphorylation reaction. We observed minor signals in the acyclic and 5-membered ring phosphate regions, referred to as **PEP** (phosphoenolpyruvate, **16b**) and **GAc5P** (glyceric acid-2,3-cyclic phosphate), respectively. A definitive structural assignment of these organic molecules, however, was not possible due to the low signal intensity of ^{31}P signals of organic products (cf. lower spectrum in Supplementary **Fig. 198**) with respect to the signal-to-noise ratio. Nevertheless, we can tentatively observe trace amounts of phosphorylated glyceric and pyruvic acids, **GAc5P** and **PEP**, as well as dodecyl phosphate (**9b**).

From experiments of the phosphorylation of pyruvic acid (**16**) we know that it has a tendency to polymerise as described in Section **7.11**. Owing to this process the ^1H NMR spectrum of the crude mixtures containing heated **16** is very complex (Supplementary **Fig. 197**).

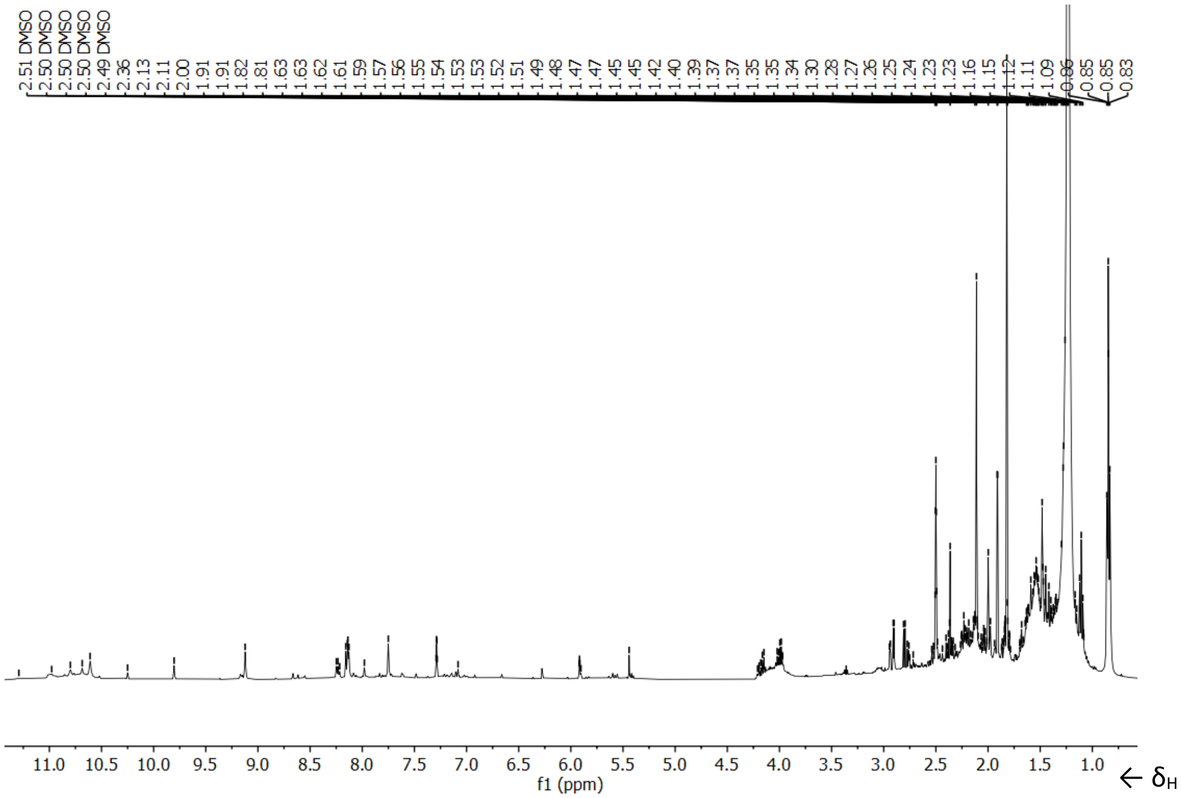

Supplementary Fig. 197. ^{1}H NMR spectrum (500 MHz, DMSO-*d6*) of crude mixtures of **9**:**2a**:**16**:$\mathbf{P_i}$ (1:1:10:1) at the 0.5 mmol reaction scale after 72 h of heating at 115 °C. δ_H (ppm) = 4.23-3.89 (*m*), 2.90 (*d*, J = 3.8 Hz), 2.11 (*s*), 1.91 (*d*, J = 1.8 Hz), 1.82 (*s*), 1.24 (*t*, J = 3.9 Hz, 8H), 1.11 (*t*, J = 7.5 Hz, 1H), 0.85 (*t*, J = 7.0 Hz, 1H).

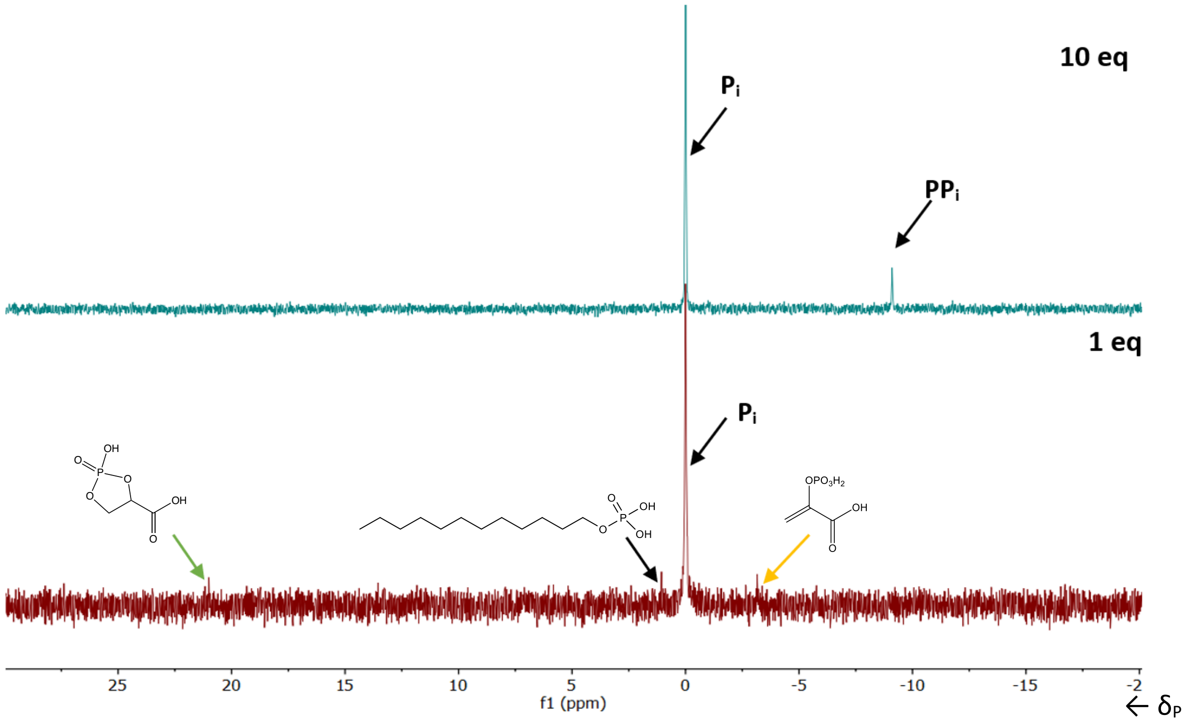

Supplementary Fig. 198. $^{31}P\{^{1}H\}$ NMR spectra (202.5 MHz, in DMSO-*d6*) 0.5 mmol scale after 72 h of heating at 115 °C of reaction mixture: **lower spectrum – 9**:**2a**:**16**:$\mathbf{P_i}$ (1:1:1:1) δ_P (ppm) = 21.00 (*s*, 5-membered ring glyceric acid-2,3-cyclic phosphate **GAc5P**), 0.00 (*s*, $\mathbf{P_i}$), –9.10 (*s*, $\mathbf{PP_i}$), 1.06 (s, dodecyl phosphate **9b**), –3.16 (s, phosphoenol pyruvate, **PEP**); **upper spectrum – 9**:**2a**:**16**:$\mathbf{P_i}$ (1:1:10:1) δ_P (ppm) = 0.00 (*s*, $\mathbf{P_i}$), –9.10 (*s*, $\mathbf{PP_i}$).

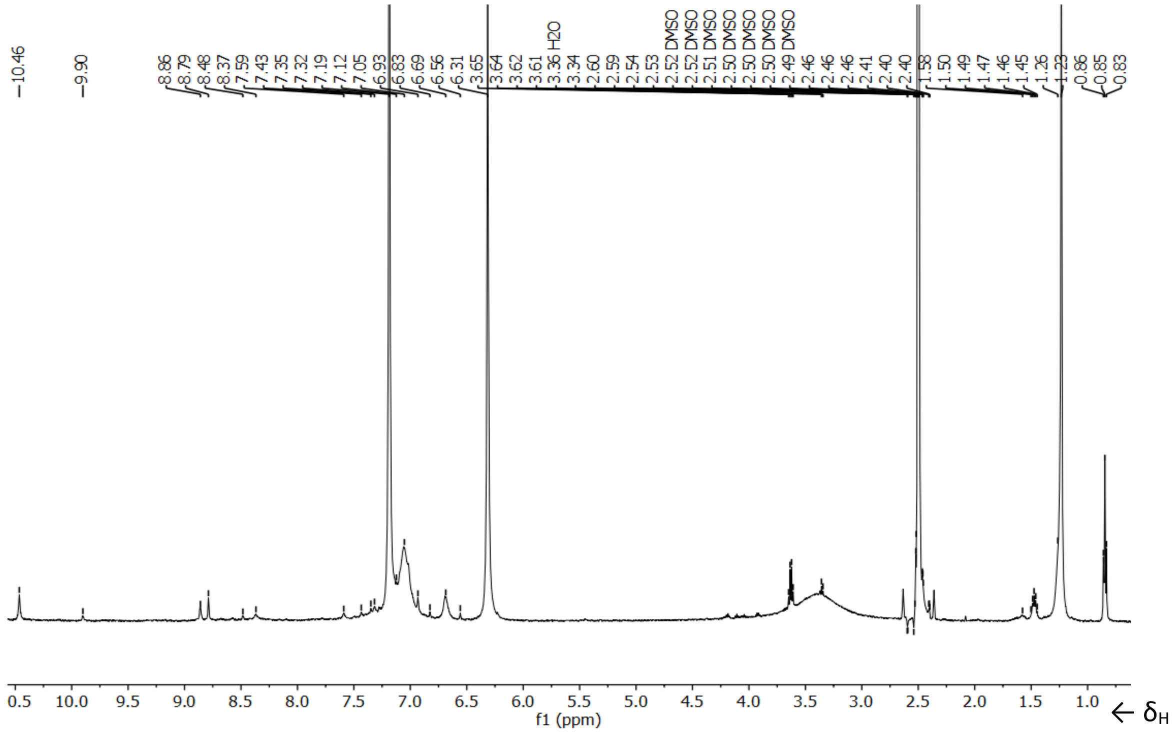

Supplementary Fig. 199. ^{1}H NMR spectrum (500 MHz, DMSO-*d6*) of crude mixtures of reaction **9**:**1**:**[$^{18}O_4$]P$_i$** (1:1:1) 0.5 mmol scale after 120 h of heating at 115 °C in 1 ml H_2O. δ_H (ppm) = 7.19 (*s*, 8H), 7.05 (*s*, 5H), 6.31 (*s*, 6H), 1.23 (*s*, 5H), 0.85 (*t*, J = 6.7 Hz, 1H).

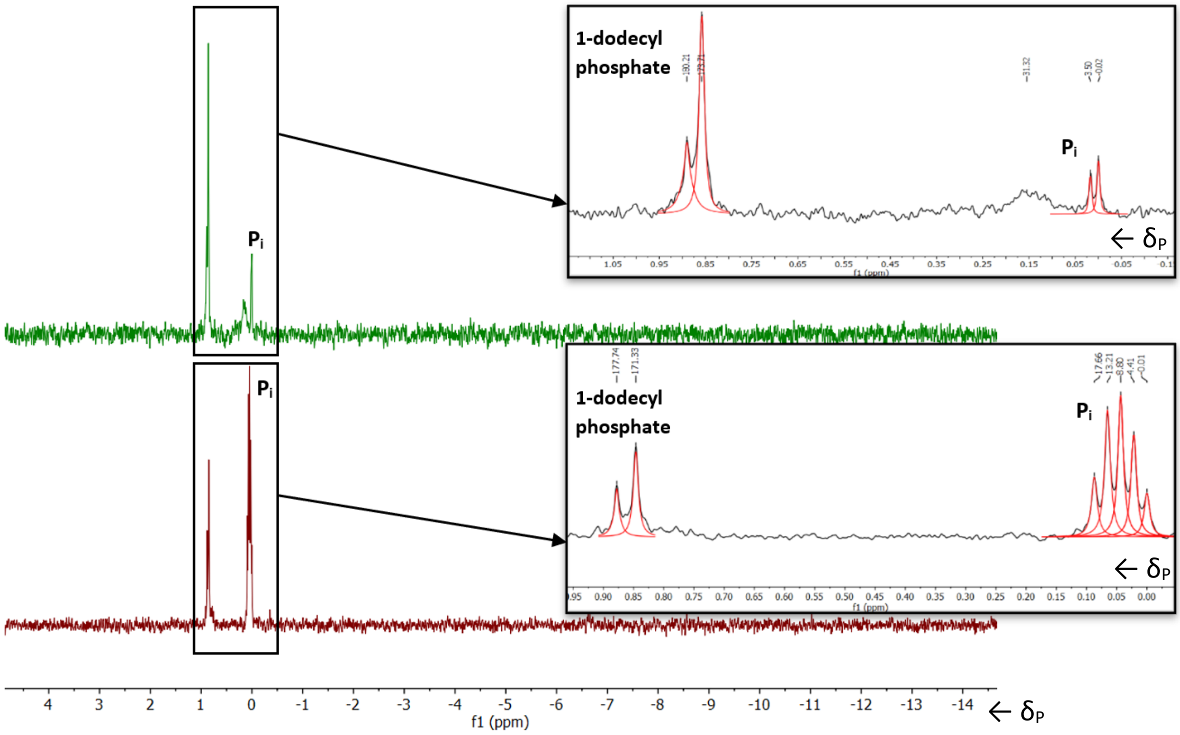

Supplementary Fig. 200. ^{31}P{^{1}H} NMR spectra (202.5 MHz, DMSO-*d6*) 0.5 mmol scale after 72 h of heating at 115 °C of reaction mixture **9**:**1**:**[$^{18}O_4$]P$_i$** (1:1:1). **Upper spectrum** — 'dry' without additional water: δ_P (ppm) = 0.88 and 0.85 (2 x *s*, $\Delta\nu$ = 6.5 Hz two main ^{18}O isotopologs of **9b**); 0.17 (broad), 0.02 and 0.00 (2 x *s*, $\Delta\nu$ ($^{18}O_1$–$^{16}O_1$) = 3.5 Hz, two main ^{18}O isotopologs of **P$_i$**). **Lower spectrum** — 'wet-to-dryness' reaction was started in 1 ml of H_2O that eventually evaporated: δ_P (ppm) = 0.88 and 0.85 (2 x *s*, $\Delta\nu$ ($^{18}O_1$–$^{16}O_1$) = 6.4 Hz, two main ^{18}O isotopologs of **9b**); 0.09, 0.07, 0.04, 0.02 and 0.00 (5 x *s*, $\Delta\nu$ ($^{18}O_1$–$^{16}O_1$) = 4.4 Hz, all five ^{18}O isotopologs of **P$_i$**). Peak frequencies in the inserts are given in Hertz. Reaction conditions detailed on page 194.

In the reaction of dodecan-1-ol (**9**) with cyanamide (**1**) and labelled **[$^{18}O_4$]P$_i$** giving 1-dodecyl phosphate (**9b**) we can observe through the isotopic shift effect[42] the exchange of O atoms from unlabelled added water with the O atoms in inorganic phosphate but not 1-dodecyl phosphate. Following the HRMS analysis of the starting phosphate salt **[$^{18}O_4$]P$_i$** (Supplementary **Fig. 10**, Supplementary **Table 3**), the two most abundant isotopologs are $NaH_2P[^{18}O_4]$ and $NaH_2P[^{16}O,^{18}O_3]$. We can assume, that the two $^{31}P\{^1H\}$ singlets of **9b** are due to $C_{12}H_{27}OP[^{18}O_3]$ (δ_P = 0.88 ppm) and $C_{12}H_{27}O_2P[^{16}O,^{18}O_2]$ (δ_P = 0.85 ppm). In the presence of natural-isotope-abundance H_2O over 72 h of the reaction, we observe five $^{31}P\{^1H\}$ singlets of inorganic phosphate in an approximately statistical intensity distribution (row 2 in Supplementary **Table 60**) that correspond to $NaH_2P[^{18}O_4]$ (δ_P = 0.09 ppm), $NaH_2P[^{16}O,^{18}O_3]$ (δ_P = 0.07 ppm), $NaH_2P[^{16}O_2, ^{18}O_2]$ (δ_P = 0.04 ppm), $NaH_2P[^{16}O_3,^{18}O]$ (δ_P = 0.02 ppm) and $NaH_2P[^{16}O_4]$ (δ_P = 0.00 ppm). Hence, in water **[$^{18}O_4$]P$_i$** was in chemical exchange with the main isotopes of oxygen brought in from water, while in the absence of added water only two **P$_i$** isotopologs were detected, **[$^{18}O_4$]P$_i$** and **[$^{18}O_3$]P$_i$** (~ 4:6) (row 1 in Supplementary **Table 60**). A similar effect was observed in Supplementary **Fig. 106B** in the reaction **6**:**2a**:**SP$_i$**:**P$_i$** (1:1:0.5:0.5).

Supplementary Table 60. Isotopolog ratio of main signals obtained from $^{31}P\{^1H\}$ NMR spectra (Supplementary **Fig. 200**). Complex signals of singlets were deconvoluted and integrated separately after extraction using the MNova software (cf. zoomed inserts of Supplementary **Fig. 200**).

	**P$_i$ isotopolog ratio**				
**Solvent**	$NaH_2P[^{18}O_4]$ %	$NaH_2P[^{16}O,^{18}O_3]$ %	$NaH_2P[^{16}O_2,^{18}O_2]$ %	$NaH_2P[^{16}O_3,^{18}O]$ %	$NaH_2P[^{16}O_4]$ %
-	41.7	58.3	-	-	-
H_2O	9.4	21.2	28.8	27.4	13.2
	**P$_o$ isotopolog ratio**				
**Solvent**	$C_{12}H_{27}{}^{16}OP[^{18}O_3]$ %	$C_{12}H_{27}{}^{16}OP[^{16}O,^{18}O_2]$ %	-	-	-
-	62.6	37.4	-	-	-
H_2O	62.6	37.4	-	-	-

The presence of water could be potentially important for the dodecanol phosphorylation in the prebiotic conditions suggested in this work. Thus, we performed HRMS of **9**, **1** and **[$^{18}O_4$]P$_i$** (1:1:1) on a 0.5 mmol scale; reaction time 72 h at 115 °C to identify differences in reaction products in 'wet-to-dryness' (Supplementary **Fig. 201**) and 'dry' conditions (Supplementary **Fig. 202**, the reaction conditions detailed on page 194). In general, the phosphorylation of dodecanol appears to be much more efficient with cyanamide than urea. The addition of water slows down phosphorylation likely due to the hydrolysis of cyanamide to urea catalysed by **P$_i$** (Supplementary **Fig. 203**). We obtained noteworthy results when equimolar, aqueous 'wet-to-dryness' or 'dry' mixtures of dodecanol, cyanamide and $NaH_2P[^{18}O_4]$ (**9**:**1**:**[$^{18}O_4$]P$_i$**) were heated for 3 days. We have found 43-80 % [$^{18}O,^{16}O$] isotopolog abundance in *O*-dodecyl carbamate. Other non-phosphorylated side-products, such as *bis*-dodecyl carbonate, *O*-dodecyl-*N*-cyanocarbamate and *O*-carbamoyl-*O*-dodecyl carbonate had also high ^{18}O contents: about 20-70 %, 67-76 % and 100 % [$^{18}O_1$], respectively (Supplementary **Table 61** and Supplementary **Table 62**). In cyanamide-assisted conditions, most dodecyl phosphates ended up as *bis*-dodecyl phosphodiester, very little dodecyl (mono)phosphate and trace amounts of *bis*-dodecyl diphosphate all showing the expected ^{18}O isotopolog abundance pattern, if not for a 19-31 % enhanced ^{16}O content and some ^{16}O-^{18}O scrambling (more $^{16}O_1{}^{18}O_3$ than expected), especially when submitted to heating in unlabelled and evaporating water. The higher ^{16}O content in dodecyl phosphates was also found in dry mixtures, which gave further evidence for the reactivity of the intermediate inorganic carbamoyl phosphate (**CP$_i$**) that slowly exchanged ^{18}O with ^{16}O isotopes prior to dodecanol attacking the carbon or phosphorous atoms of **CP$_i$** (cf. ochre zone of **Fig. 4 B**).

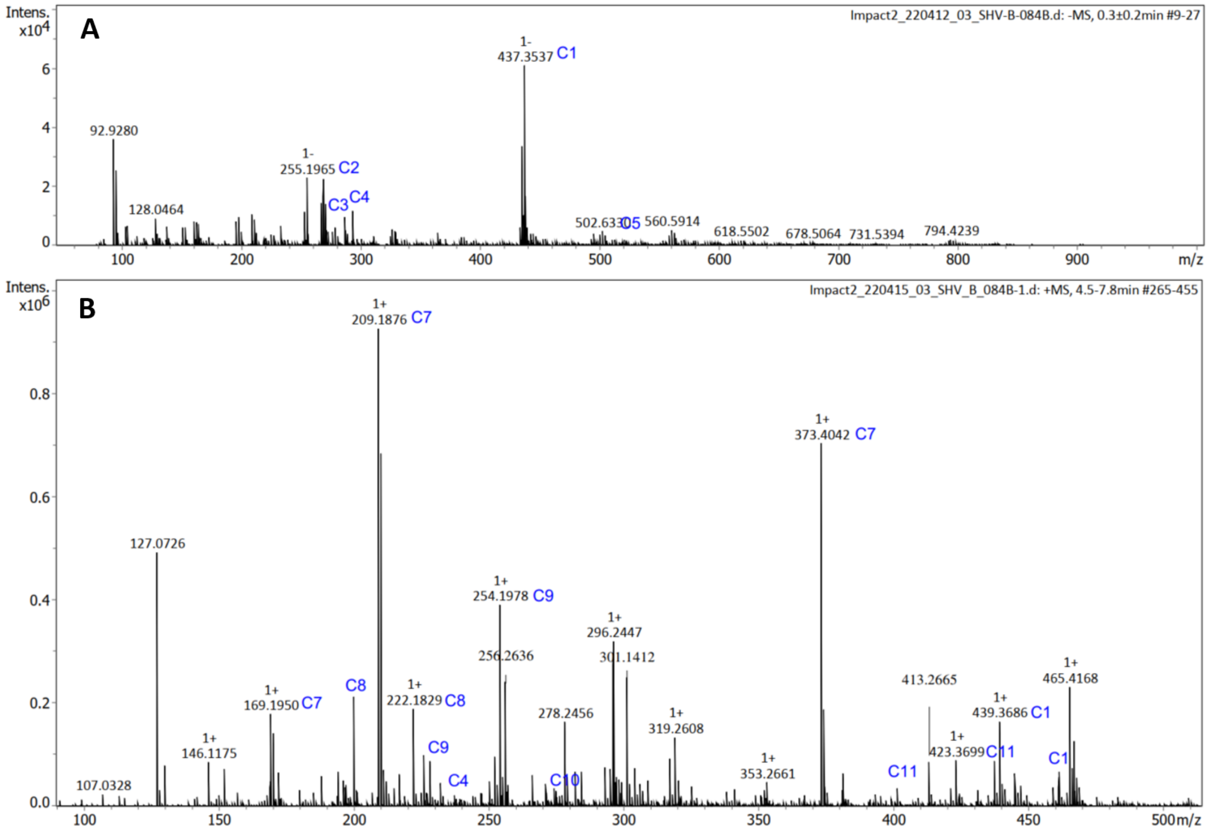

Supplementary Fig. 201. HRMS of the methanolic extract of crude mixture containing **9**, **1** and **[^{18}O$_4$]P$_i$** (1:1:1) on a 0.5 mmol scale ; reaction time 72 h at 115 °C starting in 1 ml H_2O. **A** – negative ion mode; **B** – positive ion mode. Numbers of compounds are the same as in Supplementary **Table 61**-Supplementary **Table 62** and Supplementary **Fig. 201**-Supplementary **Fig. 202**. In both figures, along with the main signals, are mentioned their adducts (same peak numbers twice).

Supplementary Table 61. Interpretation of HRMS of Supplementary **Fig. 201** with suggested formulas, structures and measured relative isotopolog ratios per compound. * The position of one ^{18}O isotope is uncertain (resulting probably from addition-elimination). Peak numbers of compounds (Supplementary **Fig. 186**) are the same for Supplementary **Table 61**-Supplementary **Table 62** and Supplementary **Fig. 201**-Supplementary **Fig. 202**.

**Peak Compound number**	***m/z* of the main signal**	**Suggested chemical formula**	**Suggested chemical structure**	**Isotopolog ratio**
C1	433.3455 $[M-H]^-$	$C_{24}H_{51}O_4P$	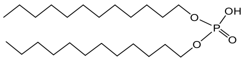	5.8
	435.3497 $[M-H]^-$	$C_{24}H_{51}O_3P[^{18}O]$	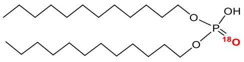	31.4
	437.3539 $[M-H]^-$	$C_{24}H_{51}O_2P[^{18}O_2]$	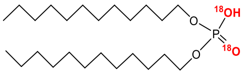	57.0
	439.3590 $[M-H]^-$	$C_{24}H_{51}OP[^{18}O_3]$	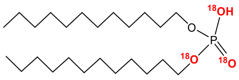 *	5.8
C2	253.1923 $[M-H]^-$	$C_{14}H_{26}N_2O_2$	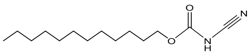	33.4
	255.1965 $[M-H]^-$	$C_{14}H_{26}N_2O[^{18}O]$	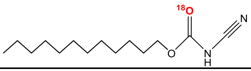	66.6
C3 (**9b**)	265.1539 $[M-H]^-$	$C_{12}H_{27}O_4P$	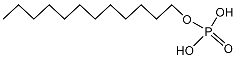	6.6
	267.1619 $[M-H]^-$	$C_{12}H_{27}O_3P[^{18}O]$	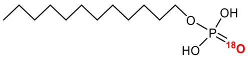	20.6
	269.1660 $[M-H]^-$	$C_{12}H_{27}O_2P[^{18}O_2]$	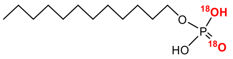	40.9
	271.1704 $[M-H]^-$	$C_{12}H_{27}OP[^{18}O_3]$	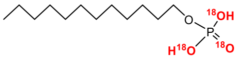	31.8
C4	267.1619 $[M+Cl]^-$	$C_{13}H_{26}O_2[^{18}O]$	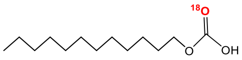	22.1
	269.1660 $[M+Cl]^-$	$C_{13}H_{26}O[^{18}O_2]$	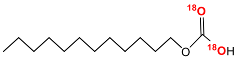	43.8
	271.1704 $[M+Cl]^-$	$C_{13}H_{26}[^{18}O_3]$	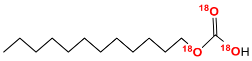 *	34.1
C5 (**9c**)	513.3126 $[M-H]^-$	$C_{24}H_{52}O_7P_2$	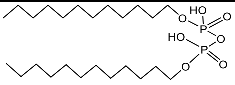	1.9
	515.3162 $[M-H]^-$	$C_{24}H_{52}O_6P_2[^{18}O]$	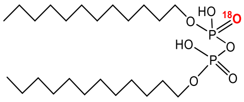	4.6
	517.3200 $[M-H]^-$	$C_{24}H_{52}O_5P_2[^{18}O_2]$	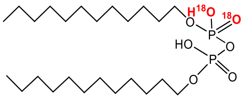	13.8
	519.3242 $[M-H]^-$	$C_{24}H_{52}O_4P_2[^{18}O_3]$	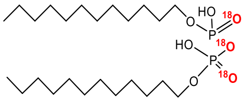	25.6
	521.3288 $[M-H]^-$	$C_{24}H_{52}O_3P_2[^{18}O_4]$	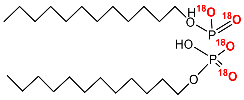	34.1
	523.3326 $[M-H]^-$	$C_{24}H_{52}O_2P_2[^{18}O_5]$	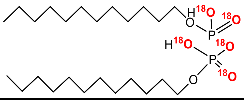	20.0
C6	-	-	-	-
C7 (**9**)	209.1876 $[M+Na]^+$	$C_{12}H_{26}O$	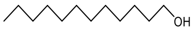	-

(continued from last page)

Peak Compound number	*m/z* of the main signal	Suggested chemical formula	Suggested chemical structure	Isotopolog ratio
C8	222.1829 [M+Na]$^+$	$C_{12}H_{25}NO$	-	-
C9 (**9d**)	252.1939 [M+Na]$^+$	$C_{13}H_{27}NO_2$	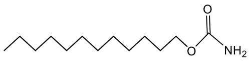	19.7
	254.1977 [M+Na]$^+$	$C_{13}H_{27}NO[^{18}O]$	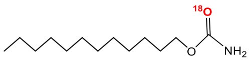	80.3
C10	276.2049 [M+H]$^+$	$C_{14}H_{27}NO_3[^{18}O]$	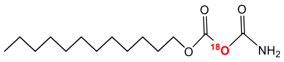	-
C11	399.3835 [M+H]$^+$	$C_{25}H_{50}O_3$	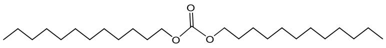	27.7
	421.3663 [M+Na]$^+$	$C_{25}H_{50}O_2[^{18}O]$	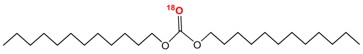	68.9
	423.3699 [M+Na]$^+$	$C_{25}H_{50}O[^{18}O_2]$	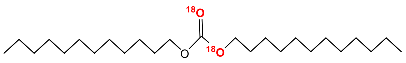 *	3.5

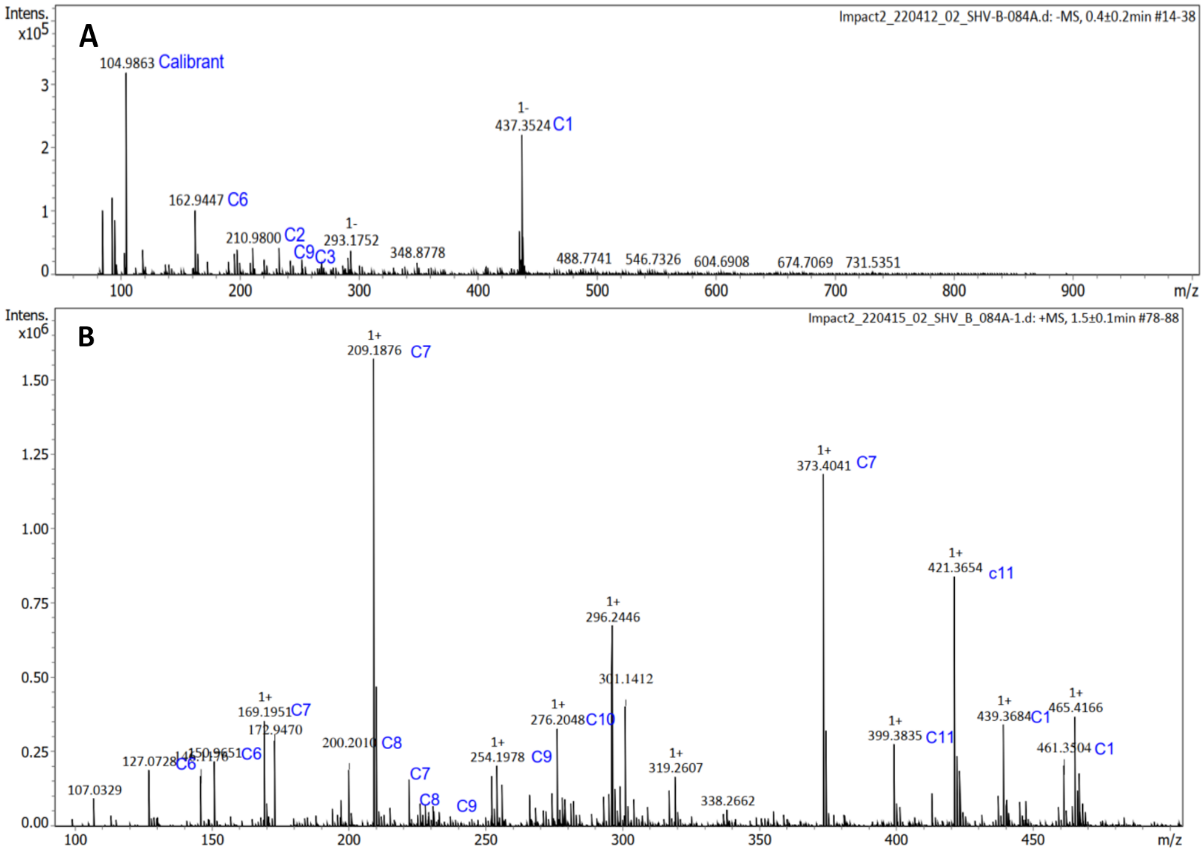

Supplementary Fig. 202. HRMS of the methanolic extract of crude mixture **9**:**1**:**[$^{18}O_4$]P$_i$** (1:1:1).; 0.5 mmol scale neat; reaction time 72 h at 115 °C. **A** – negative ion mode; **B** – positive ion mode. Numbers of compounds is the same for Supplementary **Table 61**-Supplementary **Table 62** and Supplementary **Fig. 201**-Supplementary **Fig. 202**. In both figures, along with the main signals, are mentioned their adducts (same peak numbers twice).

Supplementary Table 62. Interpretation of HRMS of Supplementary **Fig. 202** with suggested formulas, structures and measured relative isotopolog ratios per compound. * The position of one ^{18}O isotope is uncertain (resulting probably from addition-elimination). Peak numbers of compounds (Supplementary **Fig. 186**) are the same for Supplementary **Table 61**-Supplementary **Table 62** and Supplementary **Fig. 201**-Supplementary **Fig. 202**.

**Peak Compound number**	***m/z* of the main signal**	**Suggested chemical formula**	**Suggested chemical structure**	**Isotopolog ratio**
C1	433.3441 [M–H]⁻	$C_{24}H_{51}O_4P$	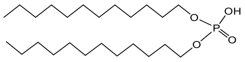	2.6
	435.3482 [M–H]⁻	$C_{24}H_{51}O_3P[^{18}O]$	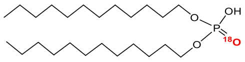	22.1
	437.3524 [M–H]⁻	$C_{24}H_{51}O_2P[^{18}O_2]$	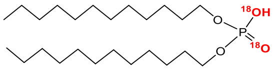	70.6
	439.3579 [M–H]⁻	$C_{24}H_{51}OP[^{18}O_3]$	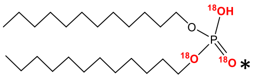 *	4.7
C2	253.1924 [M–H]⁻	$C_{14}H_{26}N_2O_2$	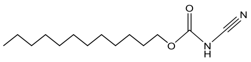	23.6
	255.1958 [M–H]⁻	$C_{14}H_{26}N_2O[^{18}O]$	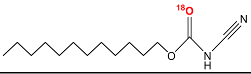	76.4
C3 (**9b**)	269.1649 [M–H]⁻	$C_{12}H_{27}O_2P[^{18}O_2]$	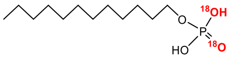	41.8
	271.1690 [M–H]⁻	$C_{12}H_{27}OP[^{18}O_3]$	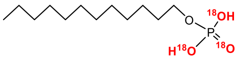	58.2
C4	-	-	-	-
C5	-	-	-	-
C6	148.9608 [M+Na]⁺	CH_3O_5P	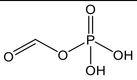	10.4
	150.9650 [M+Na]⁺	$CH_3O_4P[^{18}O]$	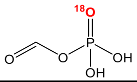	89.6
C7 (**9**)	209.1876 [M+Na]⁺	$C_{12}H_{26}O$	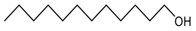	-
C8	222.1829 [M+Na]⁺	$C_{12}H_{25}NO$		-
C9 (**9d**)	252.1938 [M+Na]⁺	$C_{13}H_{27}NO_2$	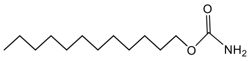	45.6
	254.1978 [M+Na]⁺	$C_{13}H_{27}NO[^{18}O]$	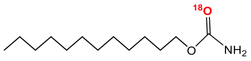	54.4
C10	274.2001 [M+H]⁺	$C_{14}H_{27}NO_4$	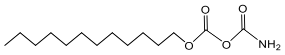	0.2
	276.2049 [M+H]⁺	$C_{14}H_{27}NO_3[^{18}O]$	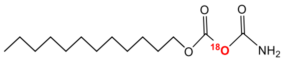	99.8
C11	399.3835 [M+H]⁺	$C_{25}H_{50}O_3$	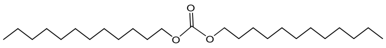	80.6
	401.3881 [M+H]⁺	$C_{25}H_{50}O_2[^{18}O]$	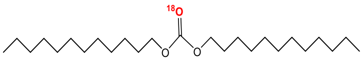	18.6
	403.3934 [M+H]⁺	$C_{25}H_{50}O[^{18}O_2]$	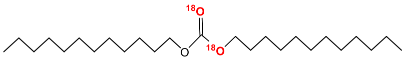 *	0.7

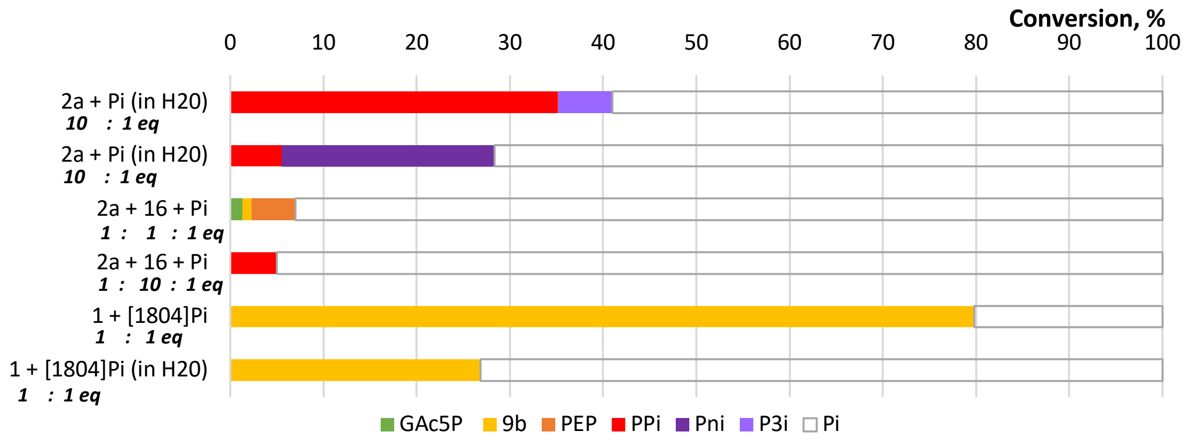

Supplementary Fig. 203. Summary of phosphorylations of dodecanol-1 (**9**) after 72-120 h of heating at 115 °C on a 0.5 mmol scale. Percent values from signal integration of quantitative $^{31}P\{^1H\}$ NMR spectra taken in DMSO-*d6*.

Supplementary Table 63. Data to Supplementary **Fig. 203**. Total conversion of initial amount of **P**$_i$ to phosphorylated organic products was calculated by subtracting inorganic compound integrals (**P**$_i$, **PP**$_i$, **P**$_{ni}$) from the sum of all integrated $^{31}P\{^1H\}$ NMR peak areas.

**Cond. agent**	**Phosphate source** (1 eq)	**Solvent**	**Reaction time, h**	**GAc5P %**	**PEP %**	**9b %**	**P$_i$ %**	**PP$_i$ %**	**P$_{3i}$ %**	**P$_{ni}$ %**	**Total conv. %**
**2a** (10 eq)	**P$_i$**	H_2O	120	-	-	-	59.01	35.16	5.83	-	-
**2a** (10 eq)	**P$_i$**	H_2O	96	-	-	-	71.64	5.53	-	22.83	-
**2a**:**16** (1:1)	**P$_i$**	-	72	1.30	4.71	1.02	92.98	-	-	-	-
**2a**:**16** (1:1)	**P$_i$**	-	72	-	-	-	95.01	4.99	-	-	-
**1**	**[^{18}O]$_4$P$_i$**	-	72	-	-	79.78	20.22	-	-	-	79.78
**1**	**[^{18}O]$_4$P$_i$**	H_2O	72	-	-	26.86	73.14	-	-	-	-

7.10 Phosphorylation of geraniol (10)

We describe our tentative and not fully explored research on the neat phosphorylation of geraniol (**10**). A few experiments revealed new products that we did not figure out completely. It is evident that the presence of unsaturated carbon-carbon bonds affects the chemistry of phosphorylation, making it different from what was studied before. In a few exemplary spectra we illustrate typical neat phosphorylation reaction products of **10** with **2a** (or **1**) and $\mathbf{P_i}$ (or $\mathbf{SP_i}$).

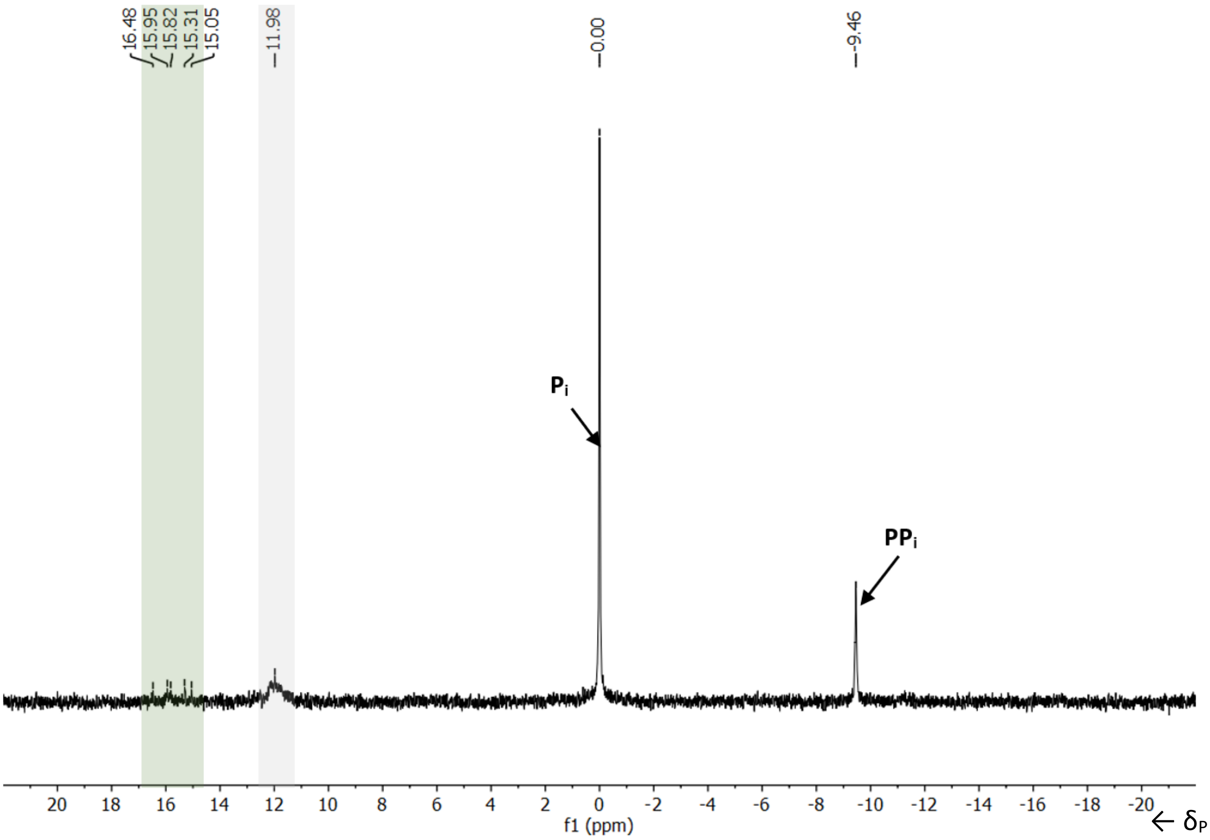

Supplementary Fig. 204. $^{31}P\{^1H\}$ NMR spectrum (202 MHz, in DMSO-*d6*) of reaction mixture **10**:**2a**:$\mathbf{P_i}$ (1:1:1), 0.5 mmol scale after 120 h of heating at 115 °C: δ_P (ppm) = 16.48-15.05 (*s*, 5-membered ring cyclic phosphates, **5cGeP**, green); 11.98 (broad *m*, $\mathbf{P_x}$, grey); 0.00 (*s*, $\mathbf{P_i}$); –9.46 (*s*, $\mathbf{PP_i}$).

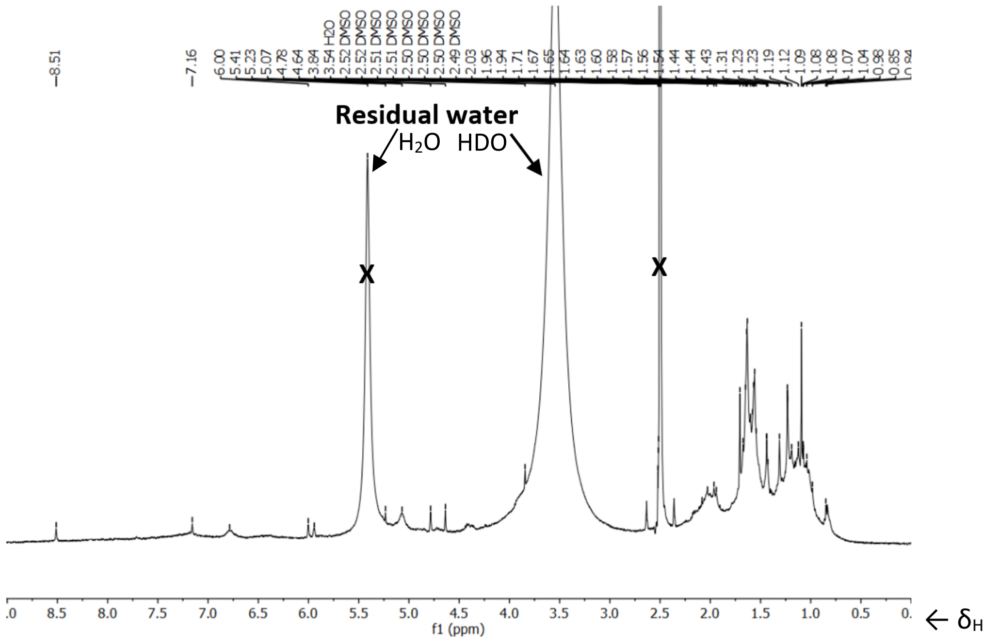

Supplementary Fig. 205. ¹H NMR spectrum (500 MHz, DMSO-*d*6) of crude mixtures of reaction **10**:**2a**:**P**$_i$ (1:1:1) 0.5 mmol scale reaction after 120 h of heating at 115 °C. **X** – residual H_2O and DMSO-*d*5.

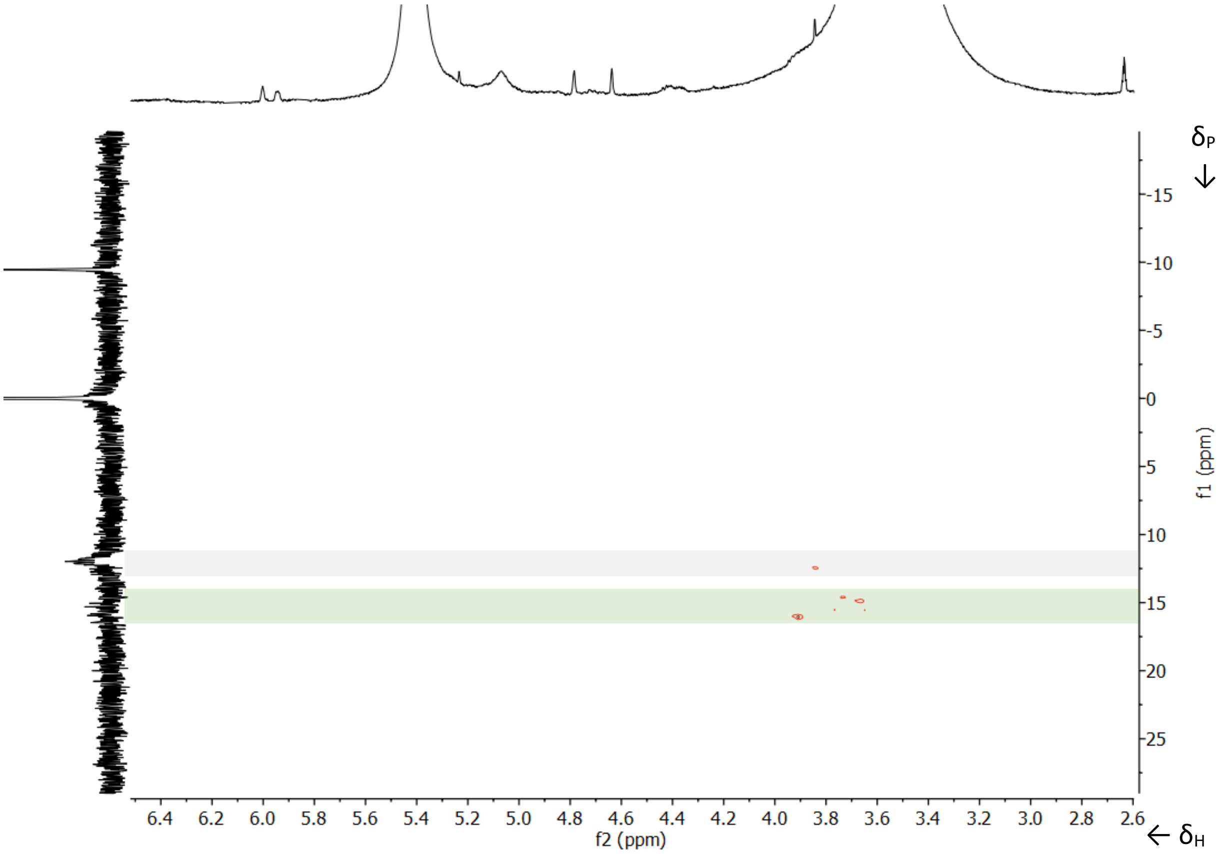

Supplementary Fig. 206. ^1H-^{31}P HMBC spectrum (500 MHz for ^1H [horizontal axis], 202.5 MHz for $^{31}P\{^1H\}$ [vertical axis], in DMSO-*d*6) of crude mixtures of reaction **10**:**2a**:**P**$_i$ (1:1:1) 0.5 mmol scale, after 120 h of heating at 115 °C. Green (**5cGeP**) and grey (**P**$_x$) zones (barely) depict organic products of phosphorylation.

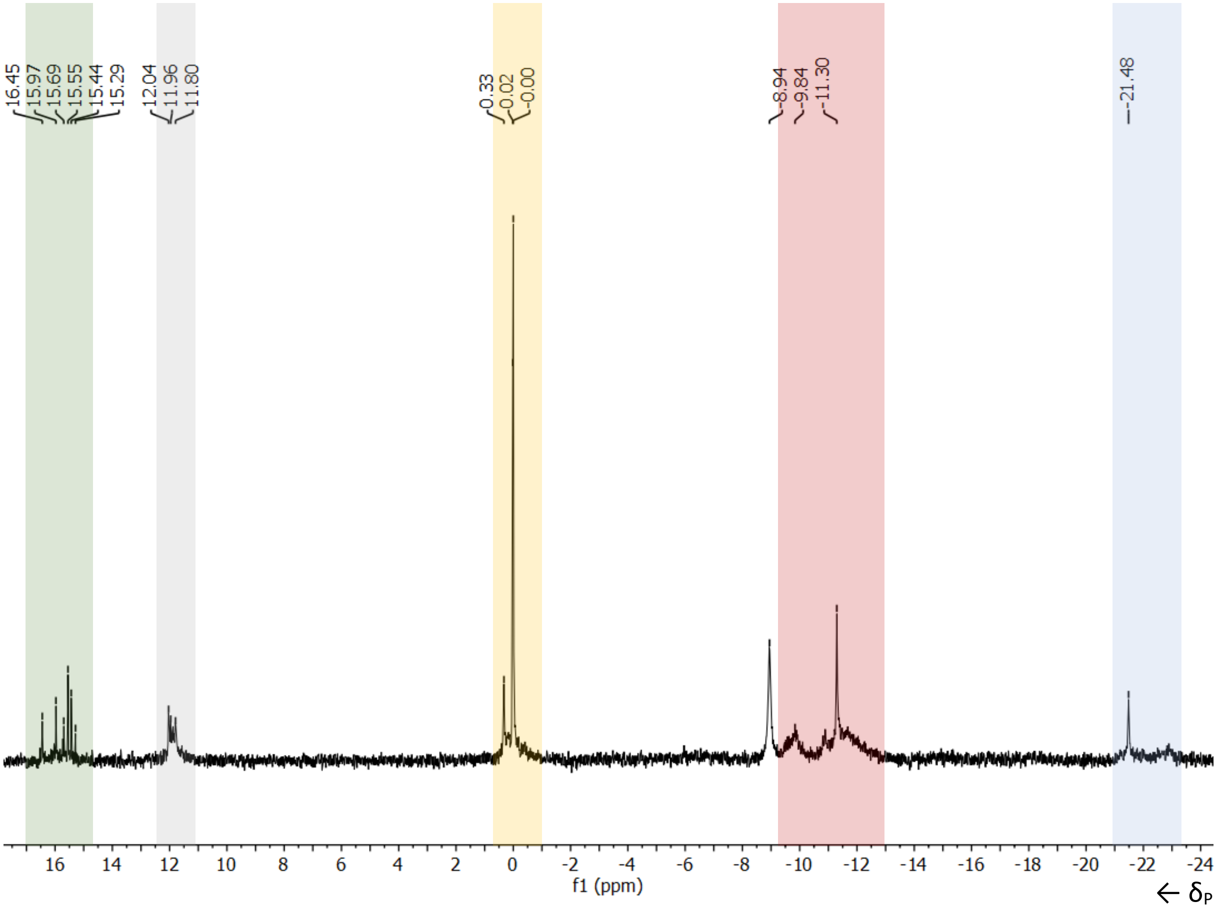

Supplementary Fig. 207. $^{31}P\{^{1}H\}$ NMR spectrum (202.5 MHz, in DMSO-*d6*) of reaction mixture **10**:**1**:$\mathbf{P_i}$ (1:1:1), 0.5 mmol scale after 120 h of heating at 115 °C: δ_P (ppm) = 16.45-15.29 (*s*, 5-membered ring cyclic phosphate, **5cGeP**, green); 12.04-11.80 (broad *m*, $\mathbf{P_x}$, grey); 0.33-0.2 (*s*, acyclic phosphate; **GeP**, ochre); 0.00 (*s*, $\mathbf{P_i}$); –8.84 and –11.30 (2 x *s*, $\mathbf{PP_i}$ + ?); from –9.2 to –13.0 (broad, digeranyl phosphates $\mathbf{Ge_2P}$, pink); –21.48 (*s*, $\mathbf{P_{ni}}$, within light blue zone).

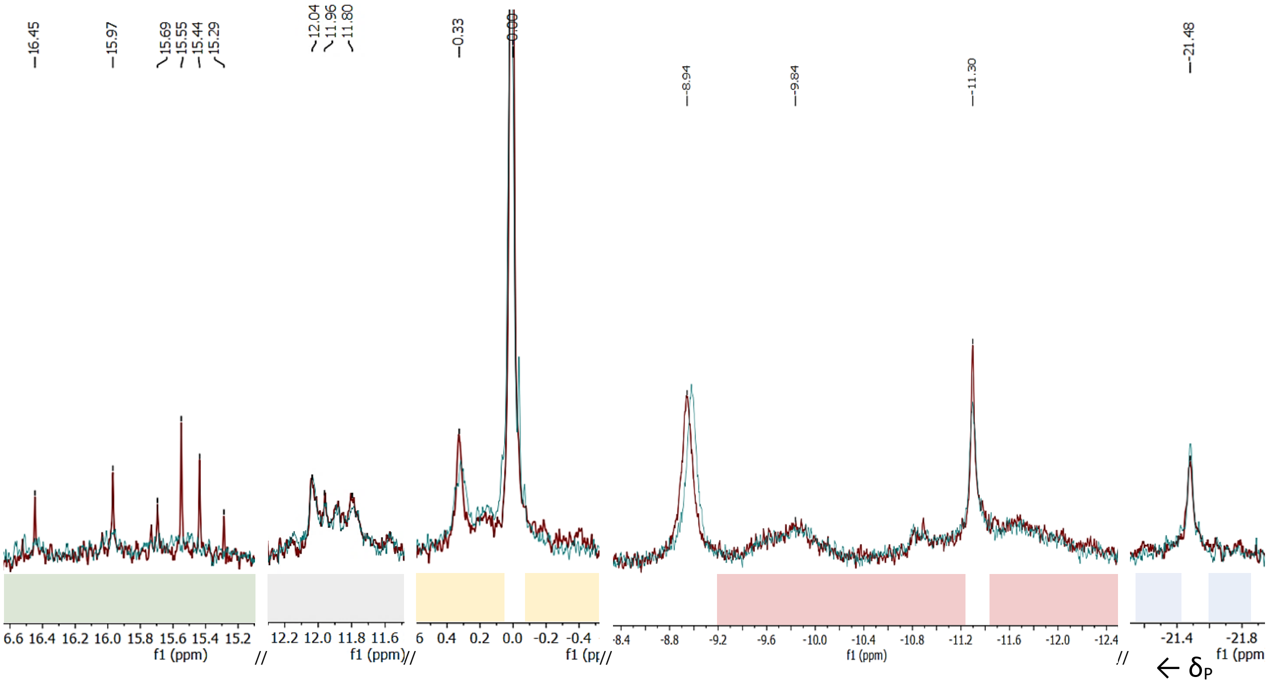

Supplementary Fig. 208. $^{31}P\{^{1}H\}$ NMR (**brown**) and ^{31}P NMR spectra (**turquoise**) (202.5 MHz, in DMSO-*d6*) of organic products **10**:**1**:$\mathbf{P_i}$ (1:1:1), 0.5 mmol scale after 120 h of heating at 115 °C. Assignments as in Supplementary **Fig. 207**.

Studying the spectra shown in Supplementary **Fig. 208** we think that the proton-coupled ^{31}P signals at δ_P = 16.5-15.3 ppm (green zone) are organic phosphates that may be the result of the phosphorylation of the C=C double-bonds of **10** creating 5-membered ring cyclic phosphates (such as **5cGeP**), which seems conceivable and consistent with published results showing that linalool was isomerised to geraniol and nerol when ortho-vanadate triesters were used[43], and taking into account that ortho-vanadate is chemically akin to ortho-phosphate. Some of the resonances at δ_P = from 0.6 to –0.6 ppm (ochre zone), should be due to geranyl phosphate and derivatives (such as **GeP**, see below and Supplementary **Fig. 207B**):

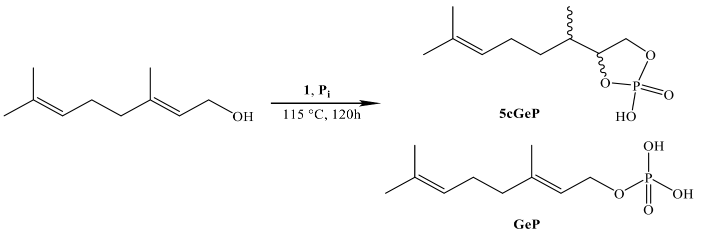

The broad signals at δ_P = 12.0-11.8 ppm (grey zone, denoted $\mathbf{P_x}$) are tentatively suggested to be associated with cyclic unsaturated monoterpene compounds that are phosphorylated on quaternary hydroxyl groups[44], which could explain the absence of any clearly visible cross-correlation between the ^{31}P and ^{1}H nuclei. The broad signals at δ_P = from –9.2 to –13.0 ppm (pink zone, denoted $\mathbf{P_o}$) could be due to phosphoramidates of geranyl cyanimide derivatives of some sort, cf. Supplementary **Fig. 209B** (**M₁** next page).

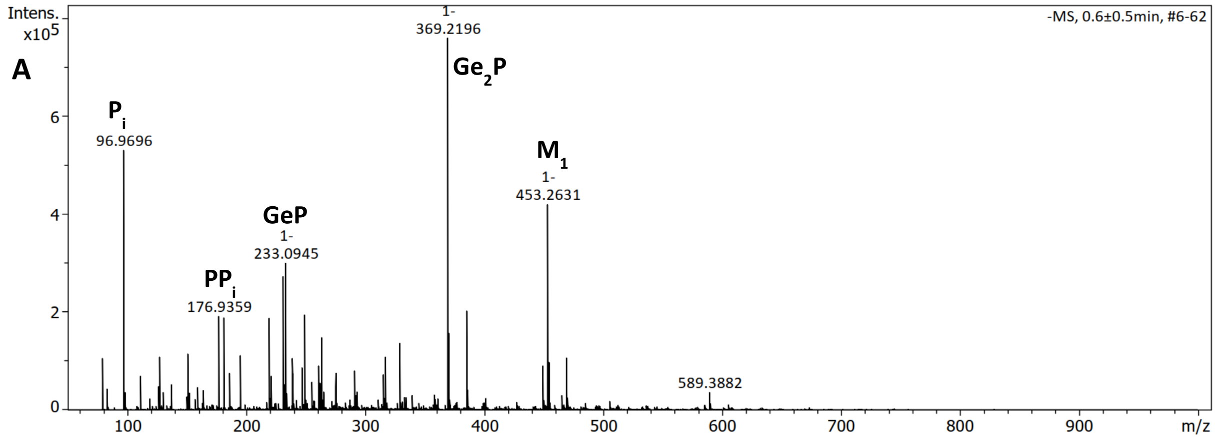

B (continued on the next page)

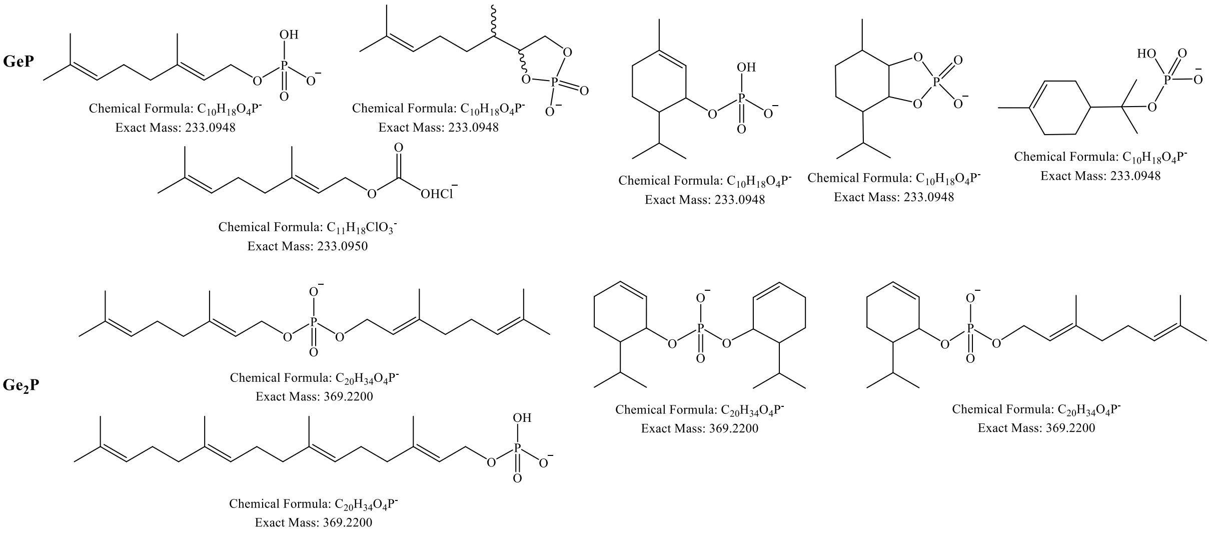

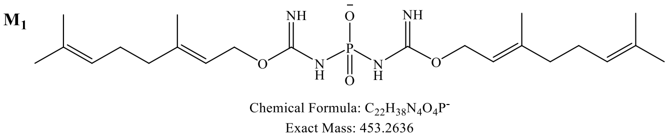

Supplementary Fig. 209. HRMS (direct injection) of the DMSO-*d6* (1 µl) extract (diluted in MeOH) of the crude mixture **10**:**1**:**P**$_i$ (1:1:1), 0.5 mmol scale after 120 h of heating at 115 °C. **A** – Total ion spectrum in negative-ion mode; **B** – Suggested molecular structures, associated chemical formulas and monoisotopic exact mass of the compounds observed using ^{31}P{^{1}H} NMR and MS spectra as shown in Supplementary **Fig. 207**, Supplementary **Fig. 208** and Supplementary **Fig. 209A**.

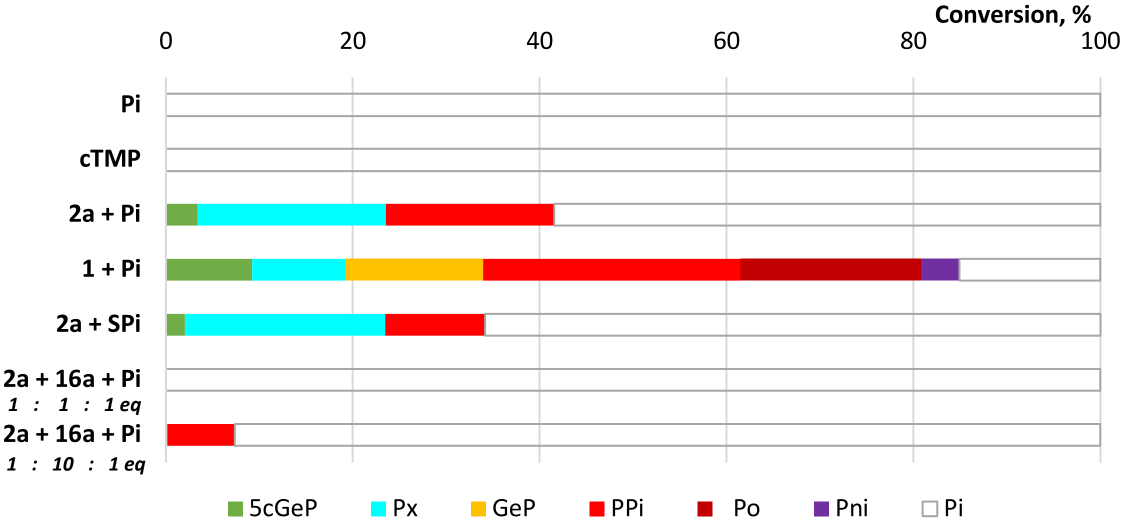

Supplementary Fig. 210. Summary of geraniol (**10**) phosphorylations after 120 h of heating at 115 °C on a 0.5 mmol scale. Percent values from signal integration of quantitative ^{31}P{^{1}H} NMR spectra taken in DMSO-*d6*. Molar ratios of reaction compounds are 1 eq with respect to starting molecule **10** if not indicated otherwise. Quantities of **P**$_x$ and **P**$_o$ unreliable (broad signals, low SNR).

In summary, heating neat equimolar mixtures of geraniol (**10**), urea (**2a**) and inorganic phosphate or thiophosphate (**P**$_i$ or **SP**$_i$) for 5 days at 115 °C did not produce any organic phosphates above 5 % (denoted **5cGeP** and **GeP**) and the presence of pyruvic acid (**16**) as an additive to **10** and **P**$_i$ totally supressed even the low amount of cyclic phosphates. The only organic phosphates from **10**, clearly identified by MS, denoted **5cGeP**, **GeP**, **P**$_x$ (difficult to quantify) and **P**$_o$ (difficult to quantify), were obtained upon the action of cyanamide (**1**) which produced compounds of suggested but yet unconfirmed structures as shown above.

Supplementary Table 64. Data to Supplementary **Fig. 210**. Total conversion of initial amount of **P**$_i$ to phosphorylated organic products was calculated by subtracting inorganic compound integrals (**P**$_i$, **PP**$_i$, **P**$_{ni}$) from the sum of all integrated ^{31}P{^{1}H} NMR peak areas. Values for **P**$_x$ and **P**$_o$ unreliable (broad signals, low SNR).

Cond. agent	Phosphorus source	5cGeP, %	P$_x$, %	GeP, %	PP$_i$, %	P$_o$, %	P$_{ni}$, %	P$_i$, %
-	**P**$_i$	-	-	-	-	-	-	100
-	**cTMP**	-	-	-	-	-	-	100
**2a**	**P**$_i$	3.39	20.15	-	17.99	-	-	58.47
**1**	**P**$_i$	9.25	9.96	14.74	27.56	19.27	4.14	15.08
**2a**	**SP**$_i$	2.01	21.47	-	10.69	-	-	65.84
**2a** + **16**	**P**$_i$	-	-	-	-	-	-	100
**2a** + **16** (10 eq)	**P**$_i$	0.17	-	-	7.20	-	-	92.63

7.11 Phosphorylation of pyruvic acid (16)

We have made much effort to involve pyruvic acid (**16**) in reactions, including different temperatures, condensing agents, phosphates and others, but we have obtained very few organic phosphorylated products. While the free enthalpies of the hydrolysis of inorganic urea (**2**), carbamoyl phosphate (**CP$_i$**), cyanate (NCO^-), inorganic pyrophosphate (**PP$_i$**), the ring-opening of cyclic trimetaphosphate (**cTMP**), or probably thiophosphate (**SP$_i$**), do not suffice to overcome the free energy of hydrolysis of phospho-enolpyruvate (PEP, **16b**), that of cyanamide (**1**) does (**Fig. 1**). And still, we discovered that any of our neat prebiotic conditions, including the use of **1** (Supplementary **Fig. 211**, Supplementary **Fig. 212**), lead most noticeably to the polymerisation of both, but each separately, **P$_i$** to inorganic condensed phosphates such as **PP$_i$** and **PPP$_i$** and condensed thiophosphates (Supplementary **Fig. 213**, Supplementary **Fig. 214**), while **16** polymerised to polyketides (Supplementary **Fig. 215**), and only in trace amounts to phosphorylated products such as phosphoenolpyruvate (PEP, **16b**) and glyceric acid-2,3-cyclic phosphate (**GAc5P**).

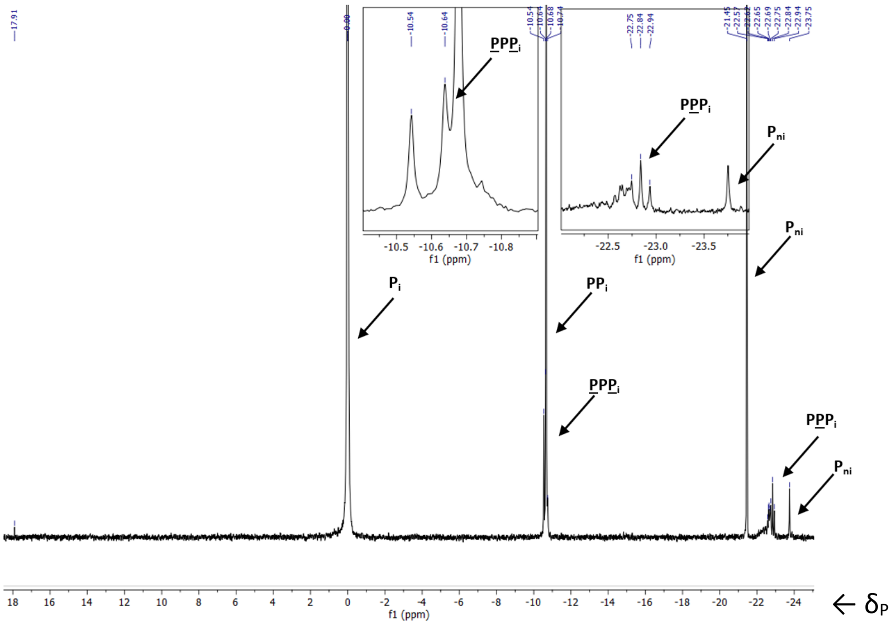

Supplementary Fig. 211. $^{31}P\{^{1}H\}$ NMR spectra (202.5 MHz, in D_2O) crude mixtures of reaction **16**:**1**:**P$_i$** (1:1:1) 0.5 mmol scale after 120 h of heating at 60 °C: δ_P (ppm) = 17.91 (*s*), 0.00 (*s*, **P$_i$**), –10.59 (*d*, *J* = 19.4 Hz, **PPP$_i$**), –10.68 (*s*, **PP$_i$**), –22.84 (*t*, *J* = 19.2 Hz, **PPP$_i$**), –21.45 and –23.75 (*s* and *s*, **P$_{ni}$**). The peak area integration of the ^{31}P doublet and triplet resonances (cf. zoomed insert) showed a 2:1 ratio, similar to Supplementary **Fig. 196**, indicating the presence of inorganic triphosphate **PPP$_i$**.

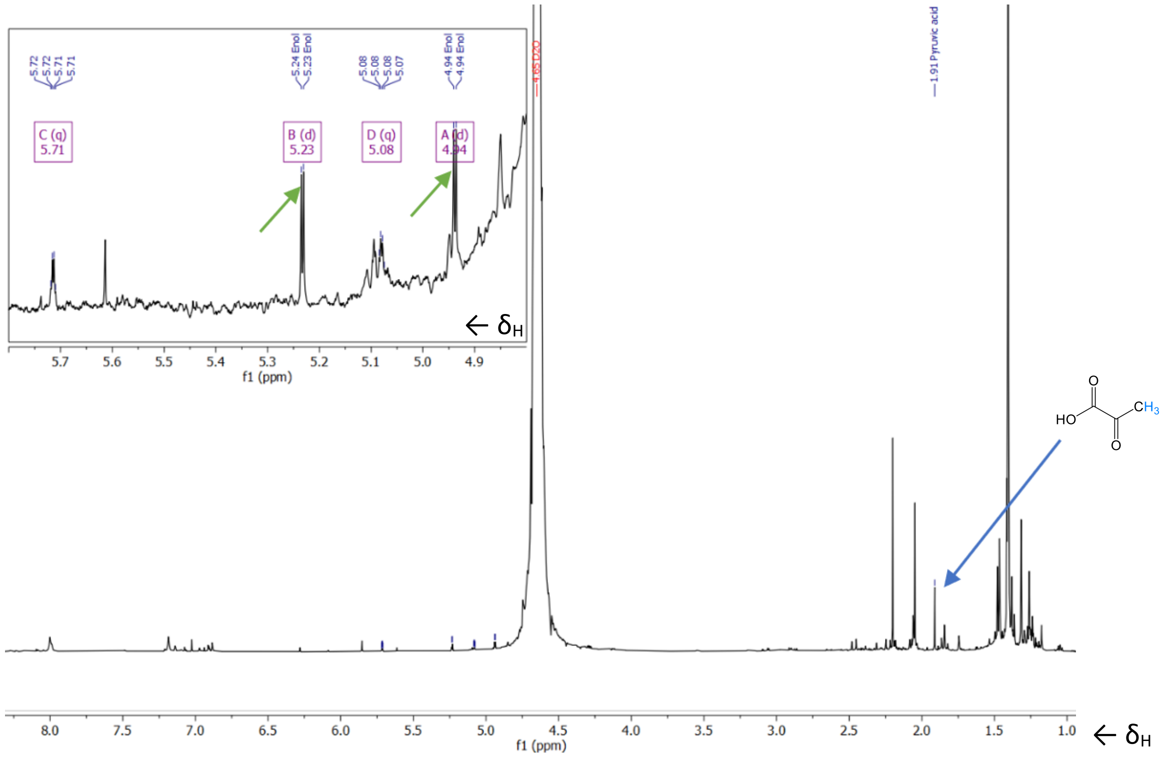

Supplementary Fig. 212. ^{1}H NMR spectra (500 MHz, D_2O): crude mixtures of reaction **16**:**1**:**P$_i$** (1:1:1) 0.5 mmol scale after 120 h of heating at 60 °C, δ_H (ppm) = 8.00 (*s*, 5H), 7.18 (*s*, 4H), 5.71 (*q*, J = 1.4 Hz, 1H), 5.23 (*d*, J = 2.3 Hz, 1H), 5.08 (*q*, J = 1.6 Hz, 2H), 4.94 (*d*, J = 2.4 Hz, 1H), 2.20 (*s*, 10H), 2.06 (*s*, 3H), 2.05 (*s*, 7H), 1.91 (*s*, 3H), 1.84 (*s*, 2H), 1.74 (*d*, J = 1.7 Hz, 2H), 1.50 (*s*, 1H), 1.48 (*s*, 5H), 1.48 (*s*, 2H), 1.47 (*s*, 14H), 1.41 (*s*, 8H), 1.41 (*s*, 20H), 1.41 (*s*, 98H), 1.38 (*s*, 3H), 1.38 (*s*, 3H), 1.36 (*s*, 3H), 1.32 (*s*, 10H), 1.29 (*s*, 1H), 1.27 (*s*, 3H), 1.26 (*s*, 7H), 1.24 (*s*, 3H), 1.23 (*s*, 1H), 1.18 (*s*, 2H). Green arrows indicate presence of enol-form molecules, derivatives of **16a**[29].

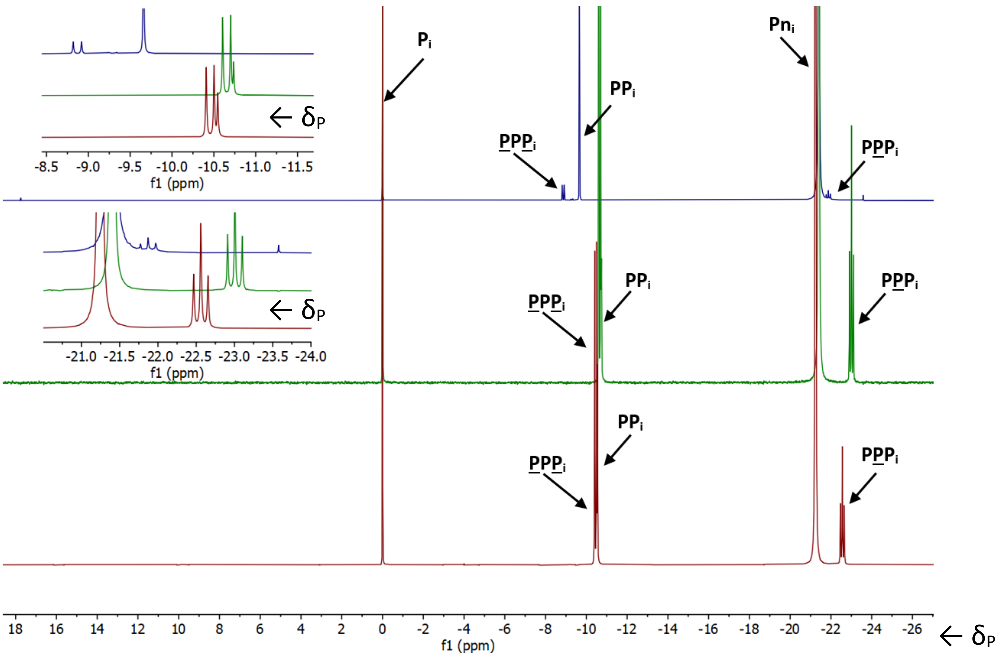

Supplementary Fig. 213. ^{31}P{^{1}H} NMR spectra (202.5 MHz, in D_2O) after 120 h of heating at 115 °C of reaction mixture (on a 0.5 mmol scale); **lower spectrum**: **16**:**cTMP** (1:1) δ_P (ppm) = 0.00 (*s*, **P$_i$**), –10.46 (*d*, J = 18.9 Hz, **PPP$_i$**), –10.55 (*s*, **PP$_i$**); –21.24 (*s*, **P$_{ni}$**), –22.56 (*t*, J = 19.0 Hz, **PPP$_i$**); **middle spectrum**: **16**:**2a**:**cTMP** (1:1:1) δ_P (ppm) = 0.00 (*s*, **P$_i$**), –10.65 (*d*, *J* = 19.3 Hz, **PPP$_i$**), –10.74 (*s*, **PP$_i$**), –21.40 (*s*, **P$_{ni}$**), –23.01 (*t*, J = 19.3 Hz, **PPP$_i$**); **upper spectrum**: **16**:**1**:**cTMP** (1:1:1) δ_P (ppm) = 17.74 (*s*), 0.00 (*s*, **P$_i$**), –8.87 (*d*, J = 20.1 Hz, **PPP$_i$**), –9.29 (*d*, *J* = 19.8 Hz), –9.66 (*s*, **PP$_i$**), –21.40 and –23.58 (*s* and *s*, **P$_{ni}$**), –21.87 (*t*, J = 20.1 Hz, **PPP$_i$**).

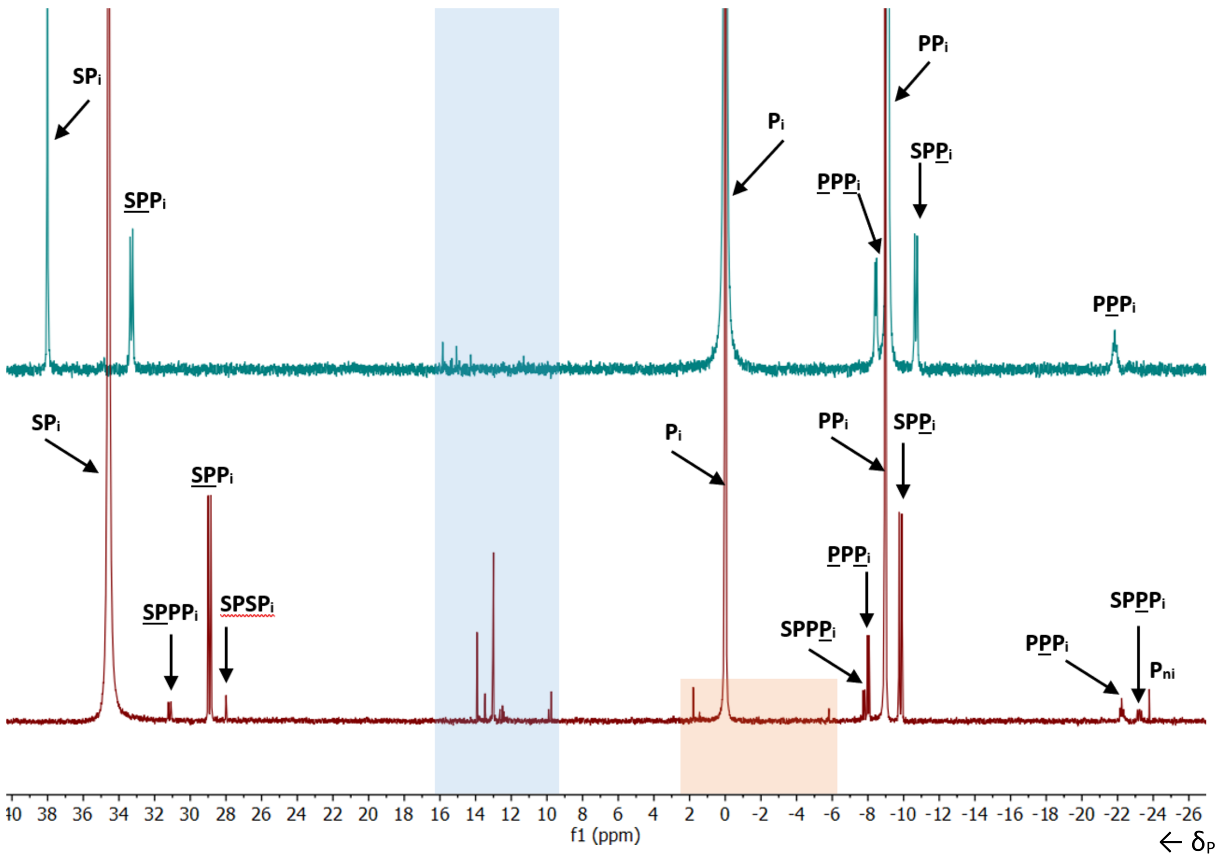

Supplementary Fig. 214. ^{31}P{^{1}H} NMR spectra (202 MHz, in D_2O) after 120 h of heating at 115 °C of reaction mixture (0.5 mmol scale); **upper spectrum**: **16**:**2a**:**P$_i$**:**SP$_i$** (1:1:0.5:0.5) δ_P (ppm) = 38.01 (s, **SP$_i$**), 33.30 (*d*, J = 27.9 Hz, **SPP$_i$**), 15.84-14.27 (unidentified cyclic 5-membered-ring phosphates, **5cPEP**, pale blue), 0.00(*s*, **P$_i$**), –8.44 (*d*, J = 20.0 Hz, **PPP$_i$**), –9.11 (*s*, **PP$_i$**), –10.69 (*d*, J = 27.7 Hz, **SPP$_i$**), –21.85 (*t*, J = 20.4 Hz, **PPP$_i$**); **lower spectrum**: **16**:**2a**:**SP$_i$** (1:1:1) δ_P (ppm) = 34.58 (s, **SP$_i$**), 31.16 (*d*, J = 28.3 Hz, **SPPP$_i$**), 28.92 (*d*, J = 29.0 Hz, **SPP$_i$**), 28.00 (*s*, **SPSP$_i$**), 13.92-9.75 (unidentified cyclic 5-membered-ring phosphates, **5cPEP**, pale blue), from 1.49 to –5.47 (**PEP**, ochre) 0.00 (*s*, **P$_i$**), –7.78 (*d*, J = 19.3 Hz, **SPPP$_i$**), –8.02 (*d*, J = 19.3 Hz, **PPP$_i$**), –8.97 (*s*, **PP$_i$**), –9.83 (*d*, J = 29.5 Hz, **SPP$_i$**), –22.23 (*t*, J = 18.8 Hz, **PPP$_i$**); –23.23 (*dd*, J = 28.7, 18.9 Hz, **SPPP$_i$**), –23.78 (*s*, **P$_{ni}$**).

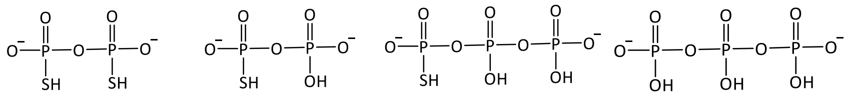

SPSP$_i$ **SPP$_i$** **SPPP$_i$** **PPP$_i$**

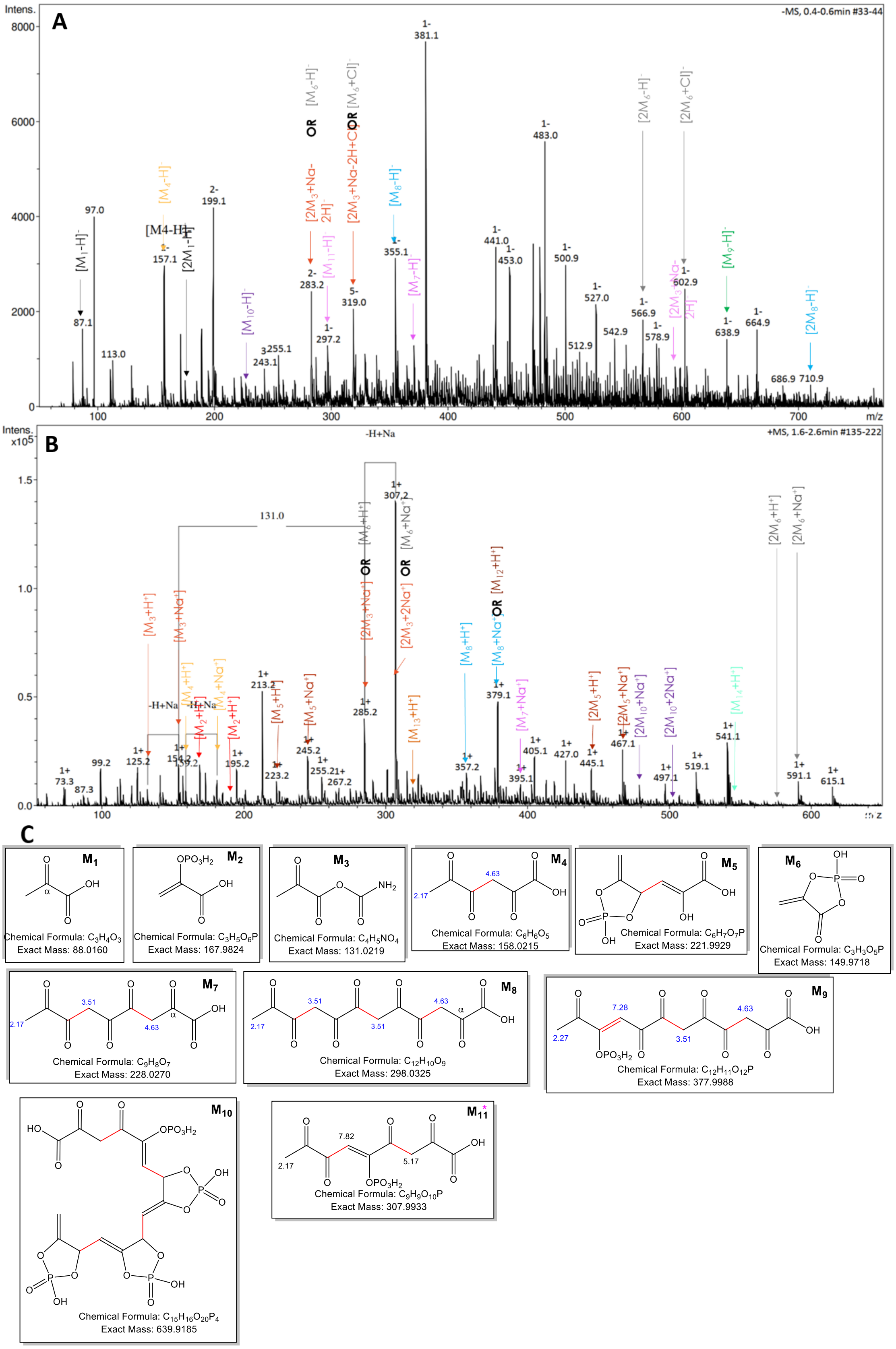

Supplementary Fig. 215. LRMS of the methanolic extract of crude mixture **16**:**2a**:**P**$_i$ (1:1:1); 0.5 mmol scale ; reaction time 120 h at 60 °C. **A** – negative ion mode; **B** – positive ion mode; **C** – Suggested molecular structures (only the most probable isomer shown), chemical formulas and monoisotopic exact masses of the compounds detected by LRMS. Values close to the main carbon chains are predicted chemical shifts [ppm] for ^{1}H NMR resonances (suggested by ChemDraw™). Red C–C bonds = newly formed from **16**.

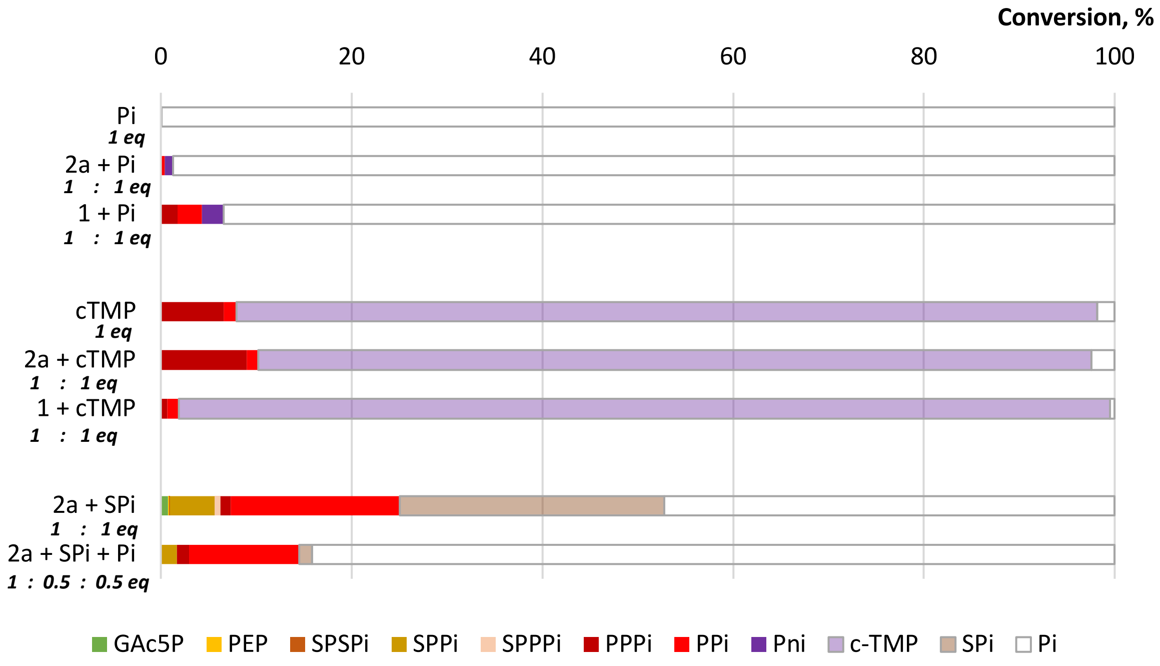

Supplementary Fig. 216. Summary of pyruvic acid (**16**) phosphorylations after 120 h of heating at 60 °C, with and without condensing agent, on a 0.5 mmol scale. Molar equivalents are indicated in the diagram with respect to starting molecule **16** (1 eq). Percent values from signal integration of quantitative $^{31}P\{^{1}H\}$ NMR spectra taken in D_2O.

As mentioned before, dry and evaporative conditions suggested in the current work are more favourable for pyruvic acid polymerisation. The phosphorylation degree in all studied experiments never exceeded about 3 %.

Supplementary Table 65. Data to Supplementary **Fig. 216**. Total conversion of initial amount of $\mathbf{P_i}$ to phosphorylated organic products was calculated by subtracting inorganic compound integrals from the sum of all integrated $^{31}P\{^{1}H\}$ NMR peak areas.

Cond. agent	Phosphate source	GAc5P %	PEP %	SPSP$_i$ %	SPP$_i$ %	SPPP$_i$ %	PPP$_i$ %	PP$_i$, %	P$_{ni}$, %	cTMP, %	SP$_i$, %	P$_i$, %
**-**	**P$_i$**	-	-	-	-	-	-	0.05	-	-	-	99.95
**2a**	**P$_i$**	-	-	-	-	-	-	0.40	0.91	-	-	98.69
**1**	**P$_i$**	0.01	-	-	-	-	1.77	2.50	2.30	-	-	93.42
**-**	**cTMP**	-	-	-	-	-	6.56	1.38	-	90.22	-	1.84
**2a**	**cTMP**	-	-	-	-	-	9.03	1.17	-	87.33	-	2.47
**1**	**cTMP**	0.01	-	-	-	-	0.69	1.15	0.03	97.61	-	0.51
**2a**	**SP$_i$**	0.73	0.12	0.10	4.68	0.65	1.06	17.67	0.04	-	27.71	47.24
**2a**	**P$_i$:SP$_i$**	0.07	-	-	1.61	-	1.25	11.61	-	-	1.28	84.18

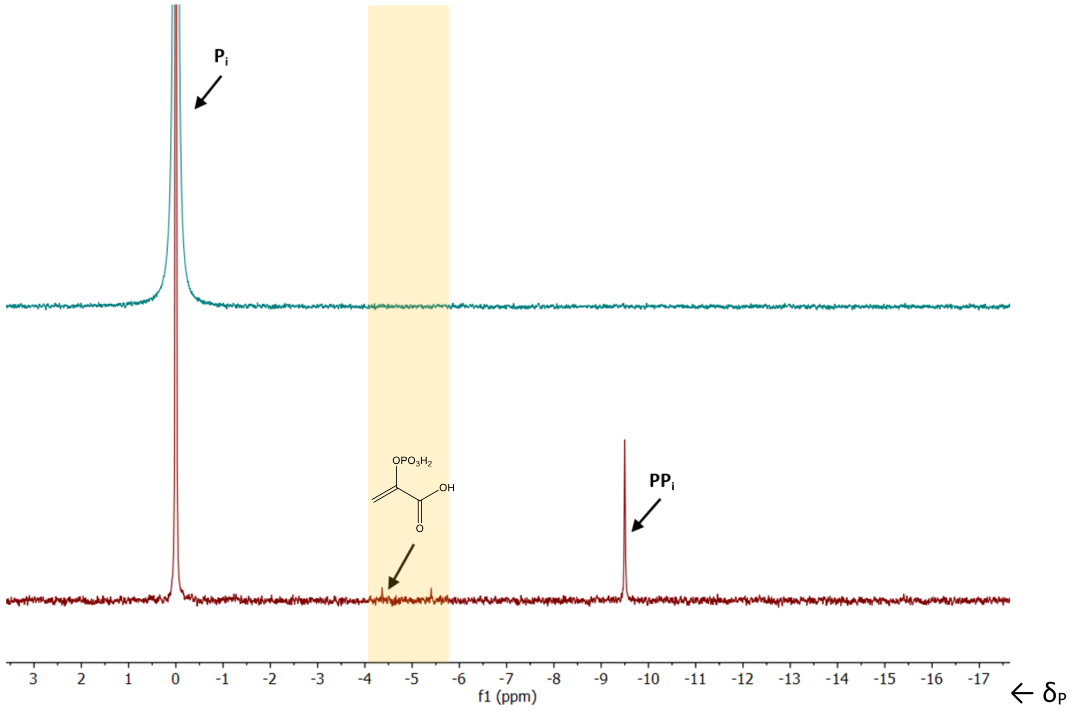

Supplementary Fig. 217. $^{31}P\{^{1}H\}$ NMR spectra (202 MHz, in DMSO-*d6*) 0.5 mmol scale after 48 h of heating at 75 °C of reaction mixture; **lower spectrum**: **16**:$\mathbf{P_i}$ (1:1): δ_P (ppm) = 0.00 (*s*, $\mathbf{P_i}$), –4.36 and –5.40 (s and s, **16b** and unknown, ochre) –9.50 (*s*, $\mathbf{PP_i}$); **upper spectrum**: **16**:**2a**:$\mathbf{P_i}$ (1:1:1): δ_P (ppm) = 0.00 (*s*, $\mathbf{P_i}$).

Signals at δ_P = –4.36 and –5.40 ppm (ochre zone) are complicated to identify with certainty. Most likely, one signal is PEP and the second is from another acyclic phosphorylated pyruvate derivative.

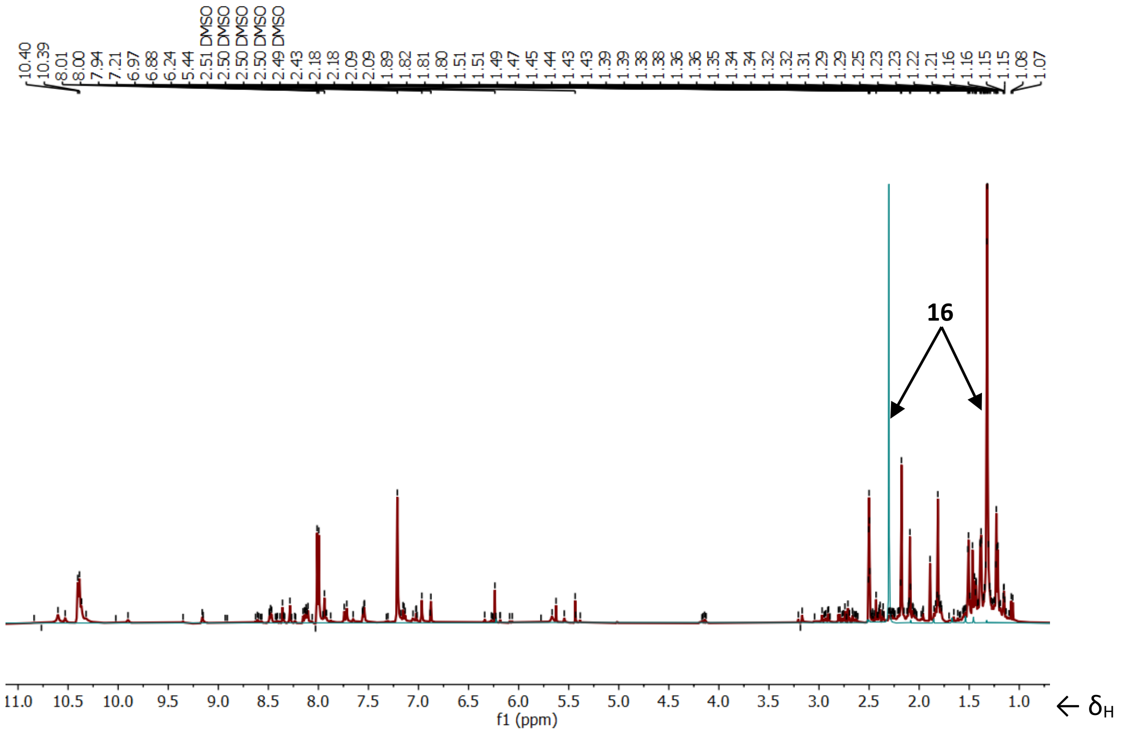

Supplementary Fig. 218. ^{1}H NMR spectra (500 MHz, DMSO-*d6*): **turquoise** – 0.5 mM **16**: δ_H (ppm) = 2.3 (*s*, 3H); **brown** – crude mixture of reaction **16**:$\mathbf{P_i}$ (1:1) 0.5 mmol scale after 48 h of heating at 75 °C, δ_H (ppm) = 10.39 (*t*, J = 9.5 Hz, 1H), 8.48 (*dd*, J = 8.8, 3.5 Hz), 8.16-8.09 (*m*), 8.00 (*d*, J = 8.6 Hz, 1H), 7.21 (*s*, 1H), 2.18 (*s*, 1H), 2.09 (*d*, J = 2.1 Hz), 1.89 (*s*), 1.81 (*s*), 1.51 (*d*, J = 3.7 Hz), 1.40-1.37 (*m*, 1H), 1.32 (*d*, J = 1.9 Hz, 3H), 1.23 (*d*, J = 2.2 Hz, 1H), 1.21 (*s*), 1.15 (*q*, J = 2.1 Hz).

Pyruvic acid (**16**), depending on the pH, can be present in two forms, as a ketoacid (oxo form) and as its hydrate[45] :

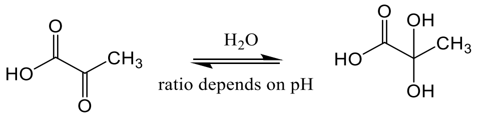

The above ^{1}H NMR spectra show at δ_H = 2.4 ppm the methyl group of the oxo form of **16** and the one at δ_H = 1.3 ppm belongs to the methyl group of pyruvic acid hydrate. We can observe after 120 h of heating **16** with **P**$_i$ in the absence of any condensing agent the main presence of the hydrated form. The occurrence of many smaller signals in the ^{1}H NMR spectrum and the absence of organic signals in the $^{31}P\{^{1}H\}$ NMR spectrum confirms the predominance of polymerisation reactions over phosphorylation.

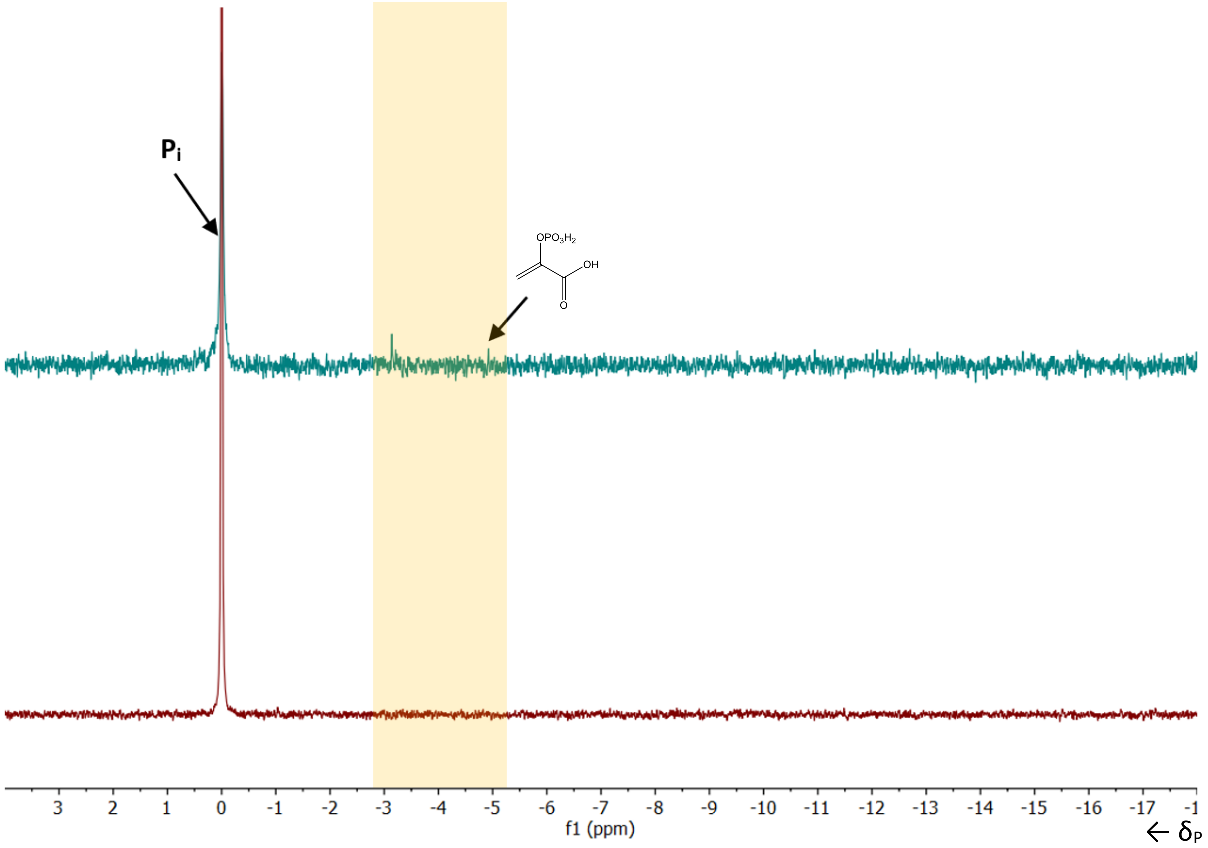

Supplementary Fig. 219. $^{31}P\{^{1}H\}$ NMR spectra (202.5 MHz, in DMSO-*d6*) after 72 h of heating at 115 °C of reaction mixture on a 0.5 mmol scale, **upper spectrum**: **16**:**2a**:**P**$_i$ (1:1:1): δ_P (ppm) = 0.00 (*s*, **P**$_i$), –3.13 and –4.92 (2 *s*, **PEP**, ochre zone), –9.50 (*s*, **PP**$_i$); **lower spectrum**: **16**:**P**$_i$ (1:1): δ_P (ppm) = 0.00 (*s*, **P**$_i$).

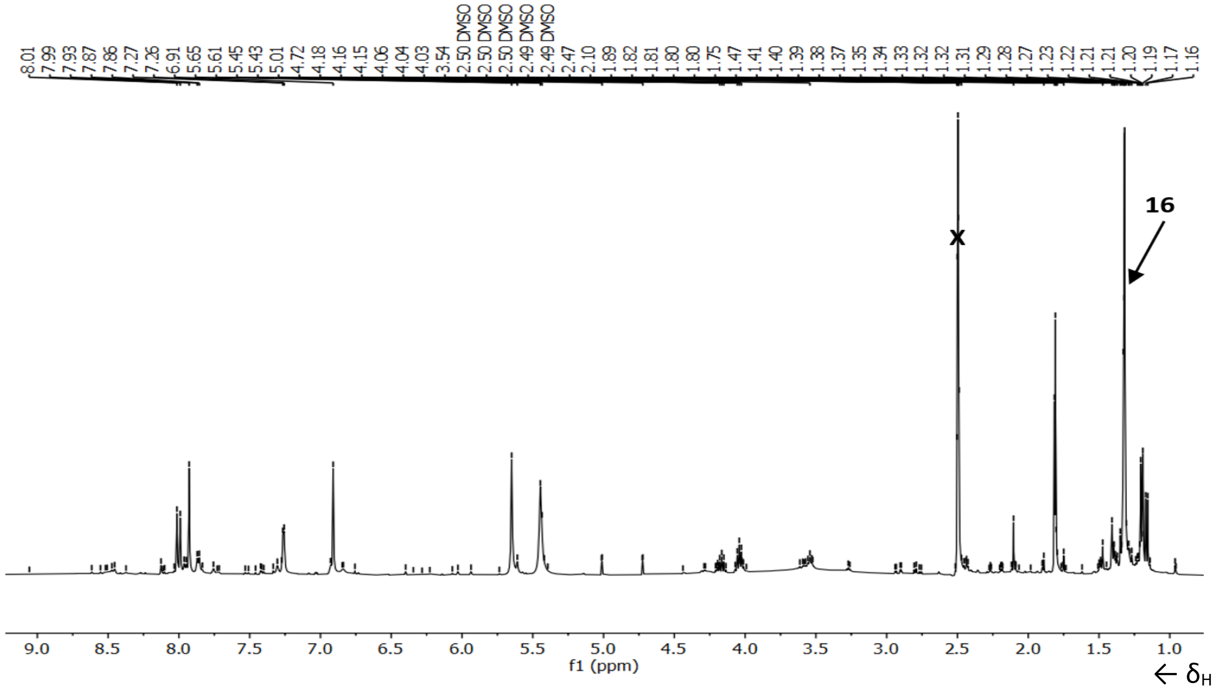

Supplementary Fig. 220. ^{1}H NMR spectrum (500 MHz, DMSO-*d*5 [**X**]) of crude mixtures of reaction **16**:**2a**:**P**$_i$ (1:1:1) 0.5 mmol scale after 72 h of heating at 115 °C: δ_H (ppm) = 8.00 (*d*, J = 11.7 Hz), 7.26 (*d*, J = 4.6 Hz), 6.91 (*s*), 5.65 (*s*, 1H), 5.44 (*d*, J = 6.2 Hz, 1H), 4.19-4.13 (*m*), 4.04 (*p*, J = 7.1 Hz), 3.57-3.49 (*m*), 2.92 (*dd*, J = 18.0, 3.8 Hz), 2.78 (*dd*, J = 17.8, 7.2 Hz), 2.10 (*s*), 1.81 (*d*, J = 3.7 Hz, 1H), 1.32 (*d*, J = 4.7 Hz, 3H), 1.18 (*dd*, J = 17.7, 7.2 Hz, 1H).

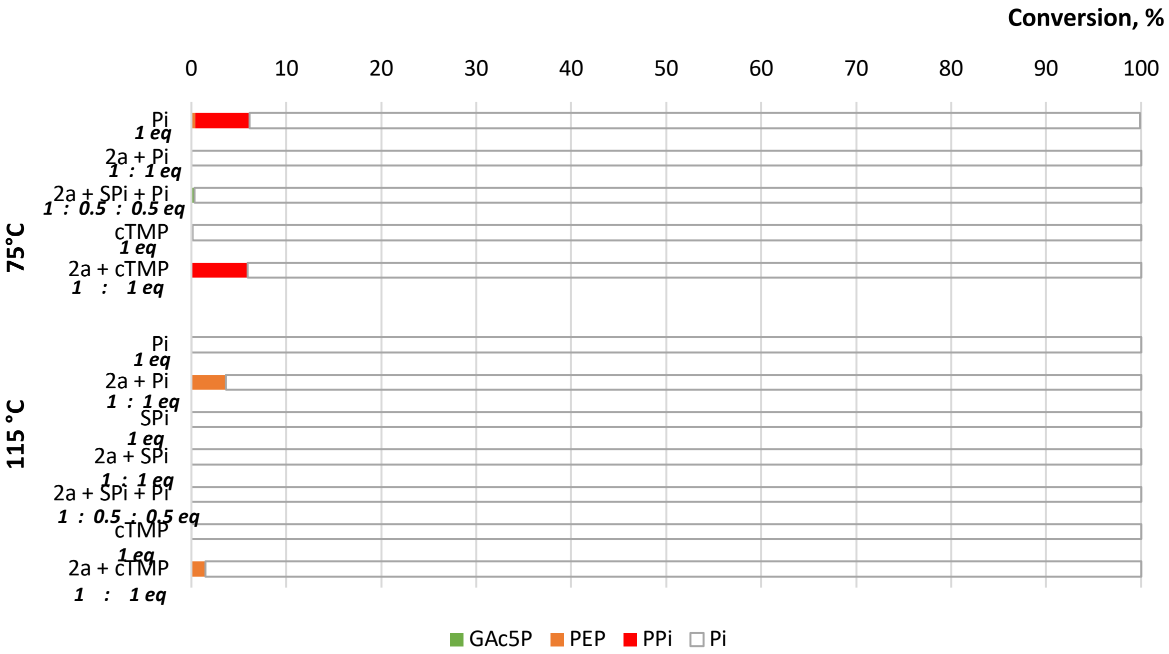

Supplementary Fig. 221. Summary of pyruvic acid (**16**) phosphorylations after 48-72 h of heating at 75/115 °C, with and without urea (**2a**), on a 0.5 mmol scale. Mol equivalents are indicated in the diagram with respect to starting comp. **16** (1 eq). Percent values from signal integration of quantitative ^{31}P{^{1}H} NMR spectra taken in DMSO-*d*6.

Thus, we can conclude that direct or condensing agent-assisted phosphorylation of neat pyruvic acid is hardly achievable in the arid prebiotic conditions studied in this work. The presence of **SP**$_i$ and **cTMP** did not accelerate the reaction; therefore, another approach for pyruvic acid phosphorylation should be investigated.

Supplementary Table 66. Data to Supplementary **Fig. 221**. Total conversion of initial amount of $\mathbf{P_i}$ to phosphorylated organic products was calculated by subtracting inorganic compound integrals ($\mathbf{P_i}$ and $\mathbf{PP_i}$) from the sum of all integrated $^{31}P\{^1H\}$ NMR peak areas.

Reaction time and temp.	Cond. agent	Phosphate source	GAc5P, %	PEP, %	PP_i, %	P_i, %
75 °C 48h	-	**P_i**	-	0.42	5.69	93.79
	**2a**	**P_i**		-	-	100
	**2a**	**P_i:SP_i**	0.34	-	-	99.66
	-	**cTMP**	-	0.14	-	99.86
	**2a**	**cTMP**	-	-	5.96	94.04
115°C 72 h	-	**P_i**	-	-	-	100
	**2a**	**P_i**		3.67	-	96.33
	-	**SP_i**	-	-	-	100
	**2a**	**SP_i**	-	-	-	100
	**2a**	**P_i:SP_i**	-	-	-	100
	-	**cTMP**	-	-	-	100
	**2a**	**cTMP**	-	1.49	-	98.51

7.11.1 Conclusion on the reactivity of pyruvic acid (16)

In spite of unfavourable thermodynamics, the minor enol tautomer (**16a**) of pyruvic acid (**PA**) was expected to be phosphorylated to some recognisable extent, through its mixed anhydride pyruvoyl phosphate (**PA-P**)[29] and the cyclic mixed anhydride of phosphoenolpyruvate (**cPEP**)[46], that could hydrolyse to **PEP** (**16b**) that might further cyclise to give the racemic 2,3-cyclic phosphate of glyceric acid (**GAc5P**, cf. black electron movements in Supplementary **Fig. 222A**). In our experiments we indeed observed detectable trace amounts of what might be **PEP** and **GAc5P**, but always in conjunction with a multitude of non-phosphorylated compounds showing quite strongly shifted ^{1}H NMR resonances (compare with software-predicted values in blue and magenta, Supplementary **Fig. 222B**). These could be oligomers of **PA** resulting from Claisen-type reactions between enol **16a** and **cPEP** (red arrows and CC-bonds, Supplementary **Fig. 222A**).

A: What could have happened first:

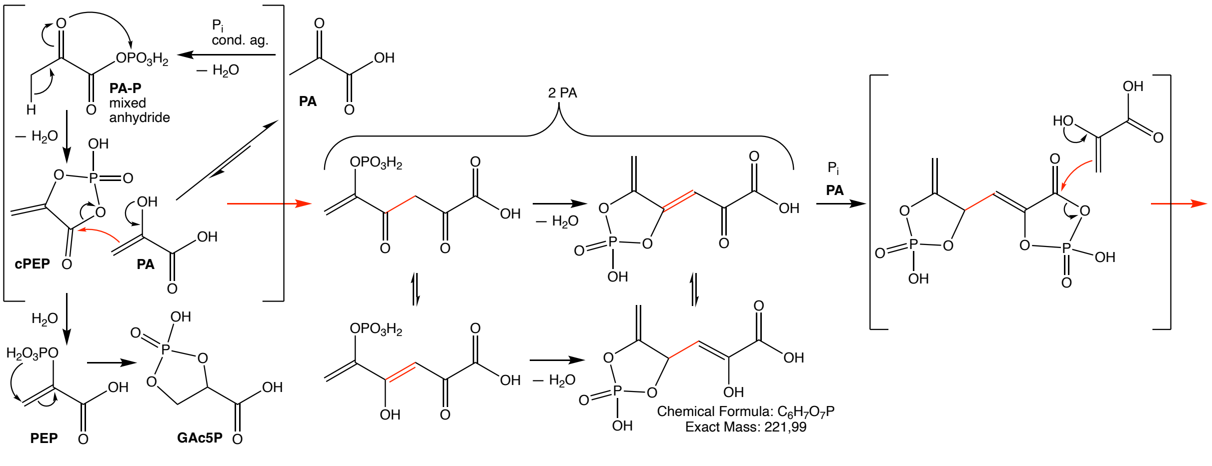

(figure continued on the next page)

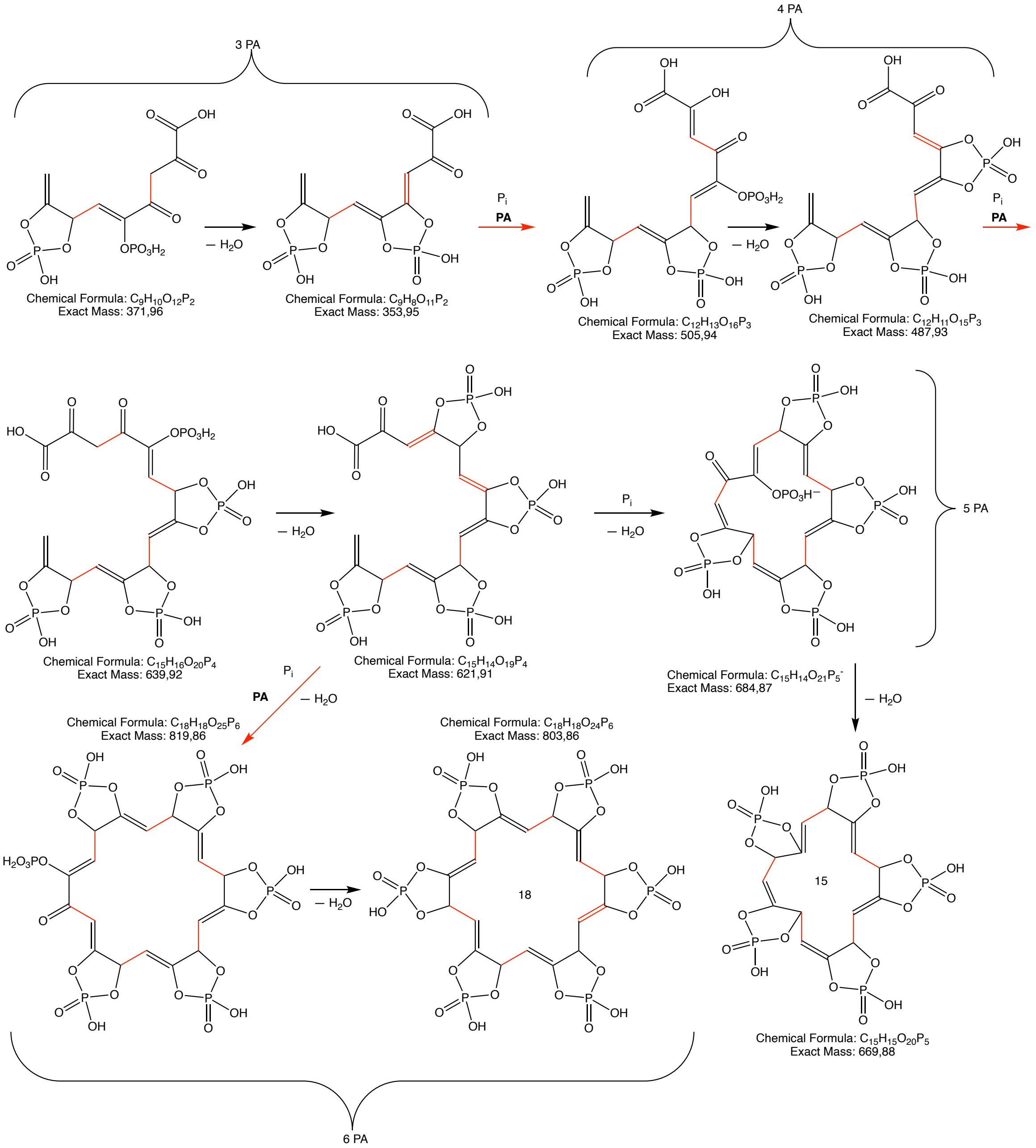

B: What could have formed later:

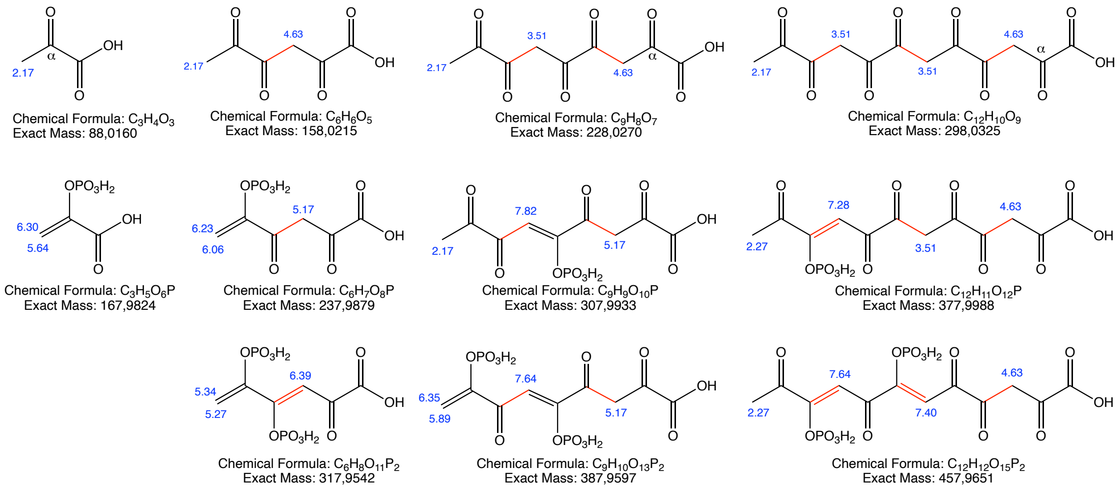

(figure continued on the next page)

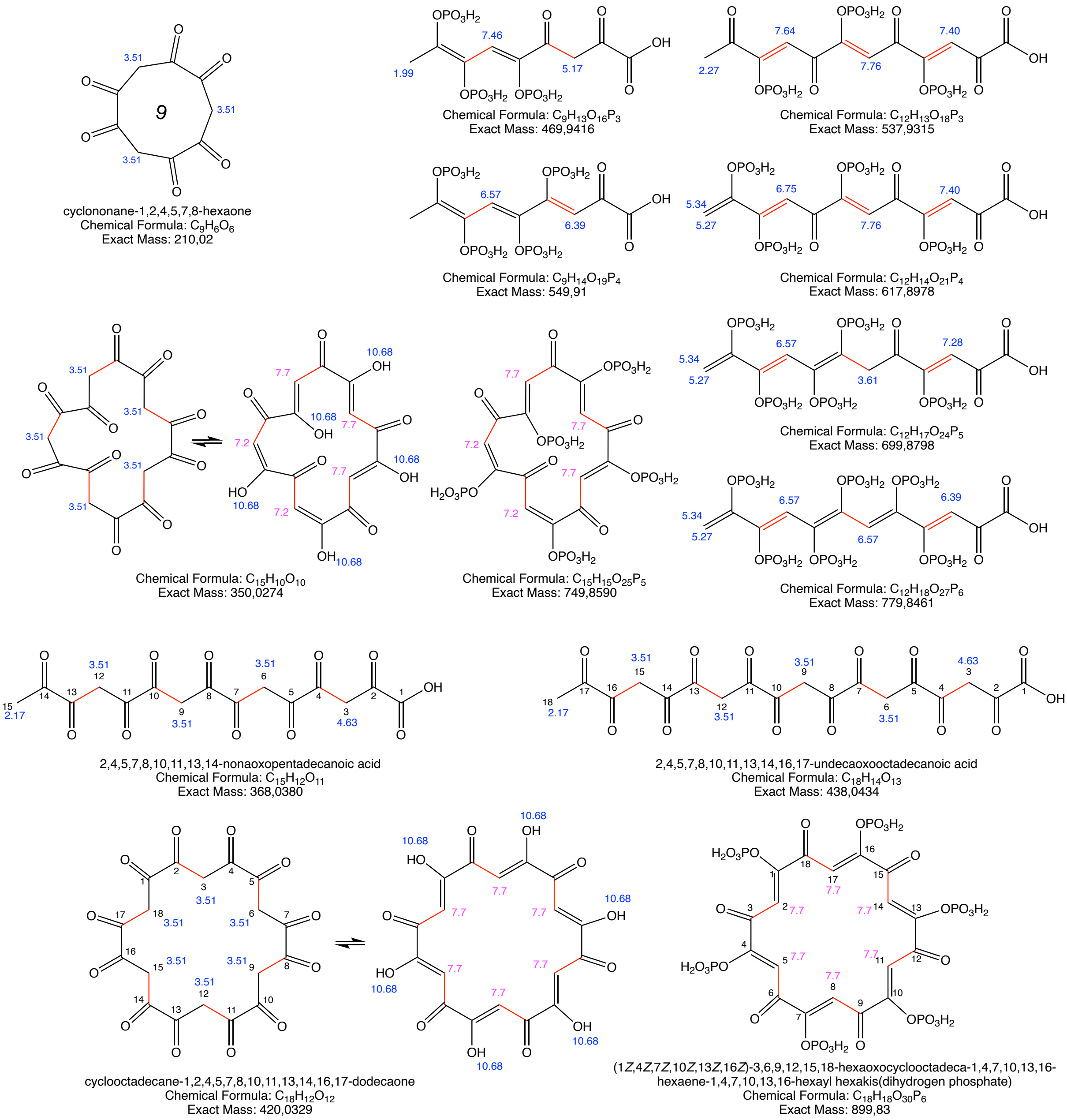

Supplementary Fig. 222. A: Suggested (hypothetic) mechanism for the phosphorylation and polymerisation of pyruvic acid (**PA**) to polyketides (n PA) under dehydrating conditions in the presence of **P$_i$** ; linear oligomers up to hexamers shown (n = 2-6). **B**: Expected fully and partly dephosphorylated PA oligomers; chemical formula, monoisotopic exact molecular masses and predicted ^{1}H NMR shifts [ppm] (in $CDCl_3$, relative to tetramethyl silane at δ_H = 0.00 ppm, blue: more certain, magenta: less certain according to ChemDraw™ Professional v 23.1.1.3).

Hence, this prebiotic pathway would lead to (cyclo)polyketides with a particular pattern of keto groups in positions (1,)2,4,5,7,8,10,11 and so forth, on the carbon chain composed of a multiple of three carbon atoms. We identified by MS oligomers up to (PA)$_5$ (Supplementary **Fig. 215**).

Finally, we tested PA (**16**) as a phosphorylation 'relay agent' that could perhaps be first phosphorylated with the help of urea (**2a**) by inorganic phosphate (**P$_i$**) to cPEP or PEP (**16b**) even in the slightest amounts, in order to further transfer it either to DOG (**8**), dodecanol (**9**) or geraniol (**10**)[47]. However, heating **16/2a/P$_i$/8** 1:1:1:1 or 10:1:1:1 at 60 °C and 115 °C for 3 days did not produce significant amounts of organic phosphate esters, and heating directly **16b/2a/P$_i$/9** 1:1:1:1 and 10:1:1:1 at 115 °C for 3 days gave maximally 1 % organic phosphates. The failed 'relay phosphorylation' of geraniol was reported at the end of Section 7.10**.**

8 Vesiculation of crude phosphorylation mixtures

A crude solid phosphorylation reaction mixture of known composition (Supplementary **Fig. 30**-Supplementary **Fig. 33**), obtained from heating MPG (**6**), NaH_2PO_4 (**P**$_i$) and urea (**2a**) in molar ratios 1:1:1 for 120 h at 115°C, was extracted using an optimised procedure. The crude mixture (2 g) was suspended in 10 ml MeOH, vortexed for 10 minutes and sonicated for the same amount of time. The suspension was filtered through a glass frit (porosity 3, SigmaAldrich®) and the residue was washed two more times with 5 ml MeOH each using the same procedure. The volume of the combined liquids was reduced through evaporation under reduced pressure to a white paste. 25 mg/ml samples were prepared. A film as thin as possible was made using 5- or 10-ml round bottom flasks according to the volume needed (usually 1 or 2 ml/mmol).* The hydrations were carried out at room temperature. Five different buffers (125 mM) have been selected among those currently used for hydration of prebiotic mixtures or in the systems chemistry or the synthetic biology domains (cf. Supplementary **Table 67**).

Supplementary Table 67. List of the buffer used, pH and results for a volume of 1 ml.

Entry	25 mg Prebiotic Mix from	Buffer Type	Concentration (MW = 408 g/mol)	Vesicles @ pH 8.0	Vesicles @ pH 9.0
1	MPG (**6**)	GlyGly	5 mM	V[a]	NV
2	MPG (**6**)	TRIS	5 mM	V	NV
3	MPG (**6**)	HEPES	5 mM	FV[b]	NV
4	MPG (**6**)	BICINE	5 mM	FV	NV
5	MPG (**6**)	GlyGly-Glycerol[d]	5 mM	NV[c]	NV

a: V = vesicles formed; b: FV = fewer vesicles, the number of vesicles was about 1/10 of V; c: NV = no vesicles found; d: 1:1 *mol/mol*, both concentrations 125 mM. * The molecular weight used for estimating the amphiphile concentration was that of MPG phosphate (MW = 408 g/mol)

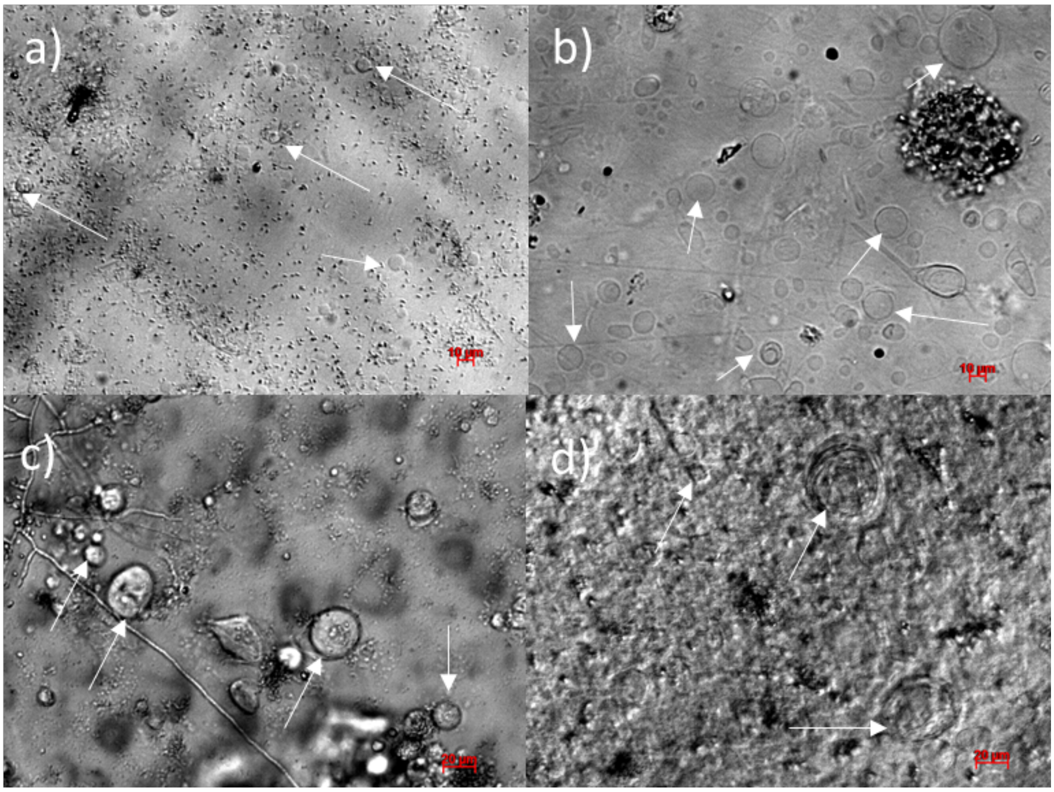

Supplementary Fig. 223. **a-d** – Bright-light microscopy images of samples of hydrated crude MPG phosphorylation mixtures, hydration conditions reported as for Supplementary **Table 67**, entries 1-4. Scale bar for **a** and **b** = 10 µm, for **c** and **d** = 20 µm. Micrographs were recorded with a Carl-Zeiss inverted microscope LSM 800 equipped with a 50x air objective and AxioCam recording. Micrographs were used without any graphical treatment and the image size was adjusted respecting the x/y pixel proportion.

9. Supplementary References

1. T. Ziegler. Approximate density functional theory as a practical tool in molecular energetics and dynamics. *Chem. Rev*. **91**, 651-667 (1991). https://doi.org/10.1021/cr00005a001.
2. J. K. Labanowski, J. Andzelm. *Density Functional Methods in Chemistry*. Eds.; Springer: New York, 1991.
3. M. J. Frisch et al. Gaussian 09, Revision C.01, Gaussian, Inc., Wallingford CT, 2010.
4. Y. Zhao, D. G. Truhlar. The M06 suite of density functionals for main group thermochemistry, thermochemical kinetics, noncovalent interactions, excited states, and transition elements: two new functionals and systematic testing of four M06-class functionals and 12 other functionals. *Theor. Chem. Acc.* **120**, 215-241 (2008). https://doi.org/10.1007/s00214-007-0310-x
5. M. J. Frisch, J. A. Pople, J. S. Binkley. Self-consistent molecular orbital methods 25. Supplementary functions for Gaussian basis sets. *J. Chem. Phys*. **80**, 3265-3269 (1984). https://doi.org/10.1063/1.447079
6. M. Walker, A. J. A. Harvey, A. Sen, C. E. H. Dessent. Performance of M06, M06–2X, and M06–HF density functionals for conformationally flexible anionic clusters: M06 functionals perform better than B3LYP for a model system with dispersion and ionic hydrogen-bonding interactions. *J. Phys. Chem.* **117**, 12590-12600 (2013). https://doi.org/10.1021/jp408166m
7. E. G. Hohenstein, S. T. Chill, C. D. Sherrill. Assessment of the performance of the M05–2X and M06–2X exchange-correlation functionals for noncovalent interactionsin biomolecules. *J. Chem. Theory Comput.* **4**, 1996-2000 (2008). https://doi.org/10.1021/ct800308k
8. R. J. Bartlett, M. Musiał. Coupled-cluster theory in quantum chemistry. *Review. Modern Phys.* **79**, 291-352 (2007). https://doi.org/10.1103/RevModPhys.79.291
9. K. B. Wiberg. Basis set effects on calculated geometries: 6-311++G** vs. aug-cc-pVDZ. *J. Comput. Chem.* **25**, 1342-1346 (2004). https://doi.org/10.1002/jcc.20058
10. K. Fukui. The path of chemical reactions — the IRC approach. *Acc. Chem. Res.* **14**, 363-368 (1981). https://doi.org/10.1021/ar00072a001
11. E. Altamura, A. Comte, A. D'Onofrio, C. Roussillon, D. Fayolle, R. Buchet, F. Mavelli, P. Stano, M. Fiore, P. Strazewski. Racemic phospholipids for origin of life studies. *Symmetry* **12**, 1108 (2020). https://doi.org/10.3390/sym12071108
12. D. Fayolle, E. Altamura, A. D'Onofrio, W. Madanamothoo, B. Fenet, F. Mavelli, R. Buchet, P. Stano, M. Fiore, P. Strazewski. Crude phosphorylation mixtures containing racemic lipid amphiphiles self-assemble to give stable primitive compartments. *Sci. Rep*. **7**, 18106 (2017). https://doi.org/10.1038/s41598-017-18053-y
13. F. H. Westheimer. Monomeric metaphosphates. *Chem. Rev.* **81**, 313–326 (1981). https://doi.org/10.1021/cr00044a001
14. S. Kamerlin, P. Sharma, R. Prasad, A. Warshel. Why nature really chose phosphate. *Quarterly Rev. Biophys.* **46**, 1-132 (2013). https://doi.org/10.1017/S0033583512000157
15. F. Duarte, J. Åqvist, N.H. Williams, S. C. Kamerlin. Resolving apparent conflicts between theoretical and experimental models of phosphate monoester hydrolysis. *J. Am. Chem. Soc.* **137**, 1081-1093 (2015). https://doi.org/10.1021/ja5082712
16. D. Petrović, K. Szeler, S. C. L. Kamerlin. Challenges and advances in the computational modeling of biological phosphate hydrolysis. *Chem. Commun.* **54**, 3077-3089 (2018). https://doi.org/10.1039/C7CC09504J

17. W. H. R. Shaw, J. J. Bordeaux. The decomposition of urea in aqueous media. *J. Am. Chem. Soc.* **77**, 4729-4733 (1955). https://doi.org/10.1021/ja01623a011
18. C. M. Allen, M. E. Jones. Decomposition of carbamylphosphate in aqueous solutions. *Biochemistry* **3**, 1238-1247 (1964). https://doi.org/10.1021/bi00897a010
19. F. Seel, F. Schinnerling. Darstellung und thermische Zersetzung von Erdalkalimetall-Carbamoylphosphaten / Preparation and thermal decomposition of carbamoyl phosphates of alkaline-earth metals. *Z. Naturforsch. B* **33**, 374-381 (1978). https://doi.org/10.1515/znb-1978-0405
20. C. Fernández-García, A. J. Coggins, M. W. Powner. A chemist's perspective on the role of phosphorus at the origins of life. *Life* **7**, 31 (2017). https://doi.org/10.3390/life7030031
21. B. Burcar, M. Pasek, M. Gull, B. J. Cafferty, F. Velasco, N. V. Hud, C. Menor-Salván. Darwin's little pond : a one-pot reaction for prebiotic phosphorylation and the mobilization of phosphate from minerals in urea-based solvent. *Angew. Chem. Int. Ed.* **55**, 13249-13253 (2016). https://doi.org/10.1002/anie.201606239
22. J. Xu, M. Tsanakopoulou, C. Magnani, R. Szabla, J. E. Šponer, J. Šponer, R. W. Góra, J. D. Sutherland. A prebiotically plausible synthesis of pyrimidine β-ribonucleosides and their phosphate derivatives involving photoanomerization. *Nature Chem.* **9**, 303-309 (2017). https://doi.org/10.1038/nchem.2664
23. N. Borduas, B. Place, G. R. Wentworth, J. P. D. Abbatt, J. G. Murphy. Solubility and reactivity of HNCO in water: insights into HNCO's fate in the atmosphere. *Atmosph. Chem. Phys*. ***16***, 703-714 (2016). https://doi.org/10.5194/acp-16-703-2016
24. M. A. Pasek. Thermodynamics of prebiotic phosphorylation. *Chem. Rev.* **120**, 4690-4706 (2020). https://doi.org/10.1021/acs.chemrev.9b00492
25. F. Tordini, A. Bencini, M. Bruschi, L. De Gioia, G. Zampella, P. Fantucci. Theoretical study of hydration of cyanamide and carbodiimide. *J. Phys. Chem. A* **107**, 1188-1196 (2003). https://doi.org/10.1021/jp026535r.
26. L. M. P. Ter-Ovanessian, B. Rigaud, A. Mezzetti, J.-F. Lambert, M.-C. Maurel. Carbamoyl phosphate and its substitutes for the uracil synthesis in origins of life scenarios. *Sci. Rep.* **11**, 19356 (2021). https://doi.org/10.1038/s41598-021-98747-6
27. O. R. Maguire, I. B. A. Smokers, W. T. S Huck. A physicochemical orthophosphate cycle via a kinetically stable thermodynamically activated intermediate enables mild prebiotic phosphorylations. *Nature Commun.* **12**, 5517 (2021). https://doi.org/10.1038/s41467-021-25555-x
28. X. Huang, F. M. Raushel. Restricted passage of reaction intermediates through the ammonia tunnel of carbamoyl phosphate synthetase. *J. Biol. Chem.* **275**, 26233-26240 (2000). https://doi.org/10.1074/jbc.275.34.26233
29. J. Zimmermann, R. J. Mayer, J. Moran. A single phosphorylation mechanism in early metabolism — the case of phosphoenolpyruvate. *Chem. Sci.* **14**, 14100-14108 (2023). https://doi.org/10.1039/D3SC04116F
30. H. Zhou, Signal-to-Noise (SNR) and uncertainty estimates. NMR protocols — Data processing (MNova) of the University of California. Updated in 2019. https://nmr.chem.ucsb.edu/protocols/SNR.html
31. J. M. Roberts, P. R. Veres, A. K. Cochran, C. Warneke, I. R. Burling, R. J. Yokelson, B. Lerner, J. B. Gilman, W. C. Kuster, R. Fall, J. de Gouw. Isocyanic acid in the atmosphere and its possible link to smoke-related health effects. *Proc. Natl. Acad. Sci. USA* **108**, 8966-8971 (2011). https://doi.org/10.1073/pnas.1103352108
32. G. Fischer, J. Geith, T. M. Klapötke, B. Krumm. Synthesis, properties and dimerization study of isocyanic acid. *Z. Naturforsch. B* **57**, 19-24 (2002). https://doi.org/10.1515/znb-2002-0103

33. H. K. Balsora, M. K. Mondal. Solubility of CO_2 in aqueous TSP. *Fluid Phase Equilibria* **328**, 21-24 (2012). https://doi.org/10.1016/j.jcou.2018.06.008

34. D. Gan, J. Ying, Y. Zhao. Prebiotic chemistry: The role of trimetaphosphate in prebiotic chemical evolution. *Front. Chem.* **10**, 941228 (2022). https://doi.org/10.3389/fchem.2022.941228

35. D. Pham Minh, J. Ramaroson, A. Nzihou, P. Sharrock. One-step synthesis of sodium trimetaphosphate ($Na_3P_3O_9$) from sodium chloride and orthophosphoric acid. *Ind. Eng. Chem. Res.* **51**, 3851-3854 (2012). https://doi.org/10.1021/ie201085b

36. L. Bindi, T. Feng, M. A. Pasek. Routes to reduction of phosphate by high-energy events. *Commun. Earth Environ*. **4**, 70 (2023). https://doi.org/10.1038/s43247-023-00736-2

37. W. A. Kibbe. OligoCalc: an online oligonucleotide properties calculator. *Nucleic Acids Res.* **35** (2007), (webserv er issue): May 25, accessed 5 Sep 2024. https://doi.org/10.1093/nar/gkm234

38. P. E. Nielsen. *Peptide Nucleic Acids, Protocols and Applications*. Horizon Bioscience, Norfolk, 2004.

39. M. W. Powner, B. Gérland, J. D. Sutherland. Synthesis of activated pyrimidine ribonucleotides in prebiotically plausible conditions. *Nature* **459**, 239-242 (2009). https://doi.org/10.1038/nature08013

40. A. N. Albertsen, C. D. Duffy, J. D. Sutherland, P.-A. Monnard. Self-assembly of phosphate amphiphiles in mixtures of prebiotically plausible surfactants. *Astrobiology* **14**, 462-472 (2014). https://doi.org/10.1089/ast.2013.1111

41. W. R. Hargreaves, S. J. Mulvil, D. W. Deamer. Synthesis of phospholipids and membranes in prebiotic conditions. *Nature* **266**, 78-80 (1977). https://doi.org/10.1038/266078a0

42. M. Cohn, A. Hu. Isotopic (^{18}O) shift in ^{31}P nuclear magnetic resonance applied to a study of enzyme-catalyzed phosphate-phosphate exchange and phosphate (oxygen)-water exchange reactions. *Proc. Natl. Acad. Sci. USA* **75**, 200-203 (1978). https://doi.org/10.1073/pnas.75.1.200

43. V. A. Semikolenov, I. I. Ilyna, R. I. Maksimovskaya. Linalool to geraniol/nerol isomerization catalyzed by $(RO)_3VO$ complexes: studies of kinetics and mechanism. *J. Mol. Catalysis A: Chemical* **204**, 201-210 (2003). https://doi.org/10.1016/S1381-1169(03)00299-1

44. V. Tsitsishvili, T. Ramishvili, I. Ivanova, I. Dobryakova, T. Bukia, N. Kokiashvili. Formation of long-chain and macrocyclic compounds during catalytic conversion of geraniol on micro- and micro-mesoporous BEA-type zeolite. *Bull. Georg. Natl. Acad. Sci*. **12**, 62-69 (2018).

45. A. Lopalco, J. Douglas, N. Denora, V. J. Stella. Determination of pK_a and hydration constants for a series of α-keto-carboxylic acids using nuclear magnetic resonance spectrometry. *J. Pharmaceut. Sci.* **105**, 664-672 (2016). https://doi.org/10.1002/jps.24539

46. G. Baccolini, C. Boga, G. Micheletti. The phosphoenolpyruvate phosphorylation: A self-organized mechanism with implications to understand the RNA transformations. *Phosphorus, Sulfur, Silicon* **185**, 2303-2315 (2010). https://doi.org/10.1080/10426501003598655

47. K. Domon, M. Puripat, K. Fujiyoshi, M. Hatanaka, S. A. Kawashima, K. Yamatsugu, M. Kanai. Catalytic chemoselective O-phosphorylation of alcohols. *ACS Central Sci.* **6**, 283-292 (2020). https://doi.org/10.1021/acscentsci.9b01272
