# Supplementary material for: A scope of prebiotic neat reaction conditions and the mechanism of urea-assisted phosphorylations of alcohols
Source: Nat Commun. 2025 Oct 8;16:8929. doi: 10.1038/s41467-025-63307-3 (PMC12508118; doi:10.1038/s41467-025-63307-3)
Supplement: Supplementary file 2 — Description of Additional Supplementary Files [file 41467_2025_63307_MOESM2_ESM.pdf]

Description of Additional Supplementary Files

Supplementary Data 1: **^{31}P {^{1}H} NMR spectra (full spectral width and zoom insert) for Suppl. Fig. 83 in the same order of appearance.** SNR = signal-to-noise ratio. X-axis: Chemical shift d_P (= f1) in parts per million (ppm). Abbreviated composition and molar ratios (left upper corner) as in the corresponding figure.

Supplementary Data 2: **^{31}P {^{1}H} NMR spectra (full spectral width and zoom insert) Fig. 87 in the same order of appearance.** SNR = signal-to-noise ratio. X-axis: Chemical shift d_P (= f1) in parts per million (ppm). Abbreviated composition and molar ratios (left upper corner) as in the corresponding figure.

Supplementary Data 3: **^{31}P {^{1}H} NMR spectra (full spectral width and zoom insert) for Suppl. Fig. 88 in the same order of appearance.** SNR = signal-to-noise ratio. X-axis: Chemical shift d_P (= f1) in parts per million (ppm). Abbreviated composition and molar ratios (left upper corner) as in the corresponding figure.

Supplementary Data 4: **^{31}P {^{1}H} NMR spectra (full spectral width and zoom insert) for Suppl. Fig. 97 in the same order of appearance.** SNR = signal-to-noise ratio. X-axis: Chemical shift d_P (= f1) in parts per million (ppm). Abbreviated composition and molar ratios (left upper corner) as in the corresponding figure.

Supplementary Data 5: **^{31}P {^{1}H} NMR spectra (full spectral width and zoom insert) for Suppl. Fig. 103 in the same order of appearance.** SNR = signal-to-noise ratio. X-axis: Chemical shift d_P (= f1) in parts per million (ppm). Abbreviated composition and molar ratios (left upper corner) as in the corresponding figure.

Supplementary Data 6: **^{31}P {^{1}H} NMR spectra (full spectral width and zoom insert) for Suppl. Fig. 104 in the same order of appearance.** SNR = signal-to-noise ratio. X-axis: Chemical shift d_P (= f1) in parts per million (ppm). Abbreviated composition and molar ratios (left upper corner) as in the corresponding figure.

Supplementary Data 7: **^{31}P {^{1}H} NMR spectra (full spectral width and zoom insert) for Suppl. Fig. 111 in the same order of appearance.** SNR = signal-to-noise ratio. X-axis: Chemical shift d_P (= f1) in parts per million (ppm). Abbreviated composition and molar ratios (left upper corner) as in the corresponding figure.

Supplementary Data 8: **Atomic coordinates (Cartesian) of all stationary states on Pathway 1_U3W0, Pathway 1_U3W2, Pathway 2_U3W0, and Pathway 2_U3W2.** Transition states (TS) and intermediate states (Int) obtained from the optimisation at the M06-2X/6-31G(d,p) level of theory.
