# Supplementary material for: A scope of prebiotic neat reaction conditions and the mechanism of urea-assisted phosphorylations of alcohols
Source: Nat Commun. 2025 Oct 8;16:8929. doi: 10.1038/s41467-025-63307-3 (PMC12508118; doi:10.1038/s41467-025-63307-3)
Supplement: Supplementary file 3 — Supplementary Data 1 [file 41467_2025_63307_MOESM3_ESM.pdf]

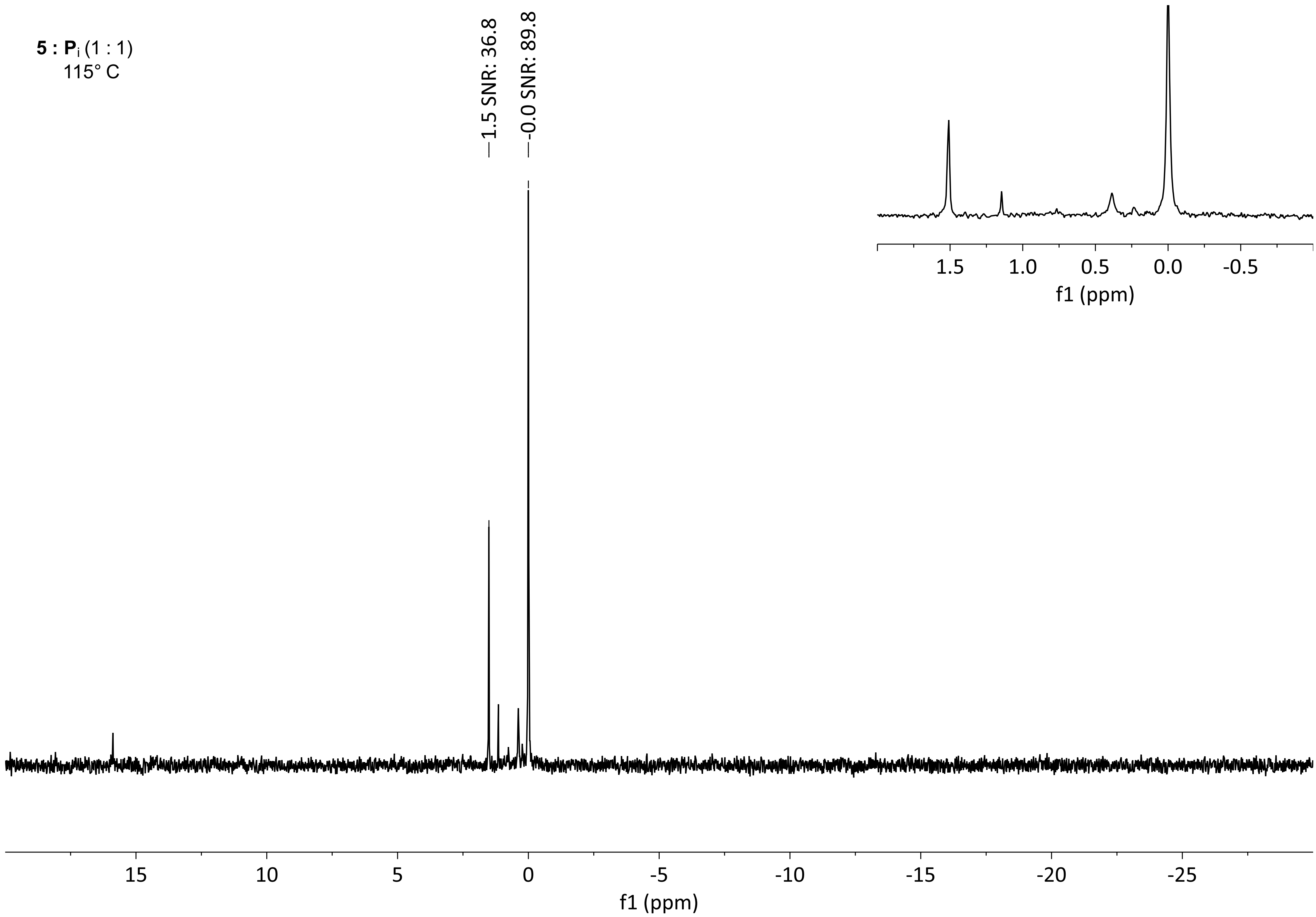

5 : Pi (1 : 1)
115° C
1.5 SNR: 36.8
-0.0 SNR: 89.8
1.5
1.0
0.5
0.0
-0.5
f1 (ppm)
15
10
5
0
-5
-10
-15
-20
-25
f1 (ppm)

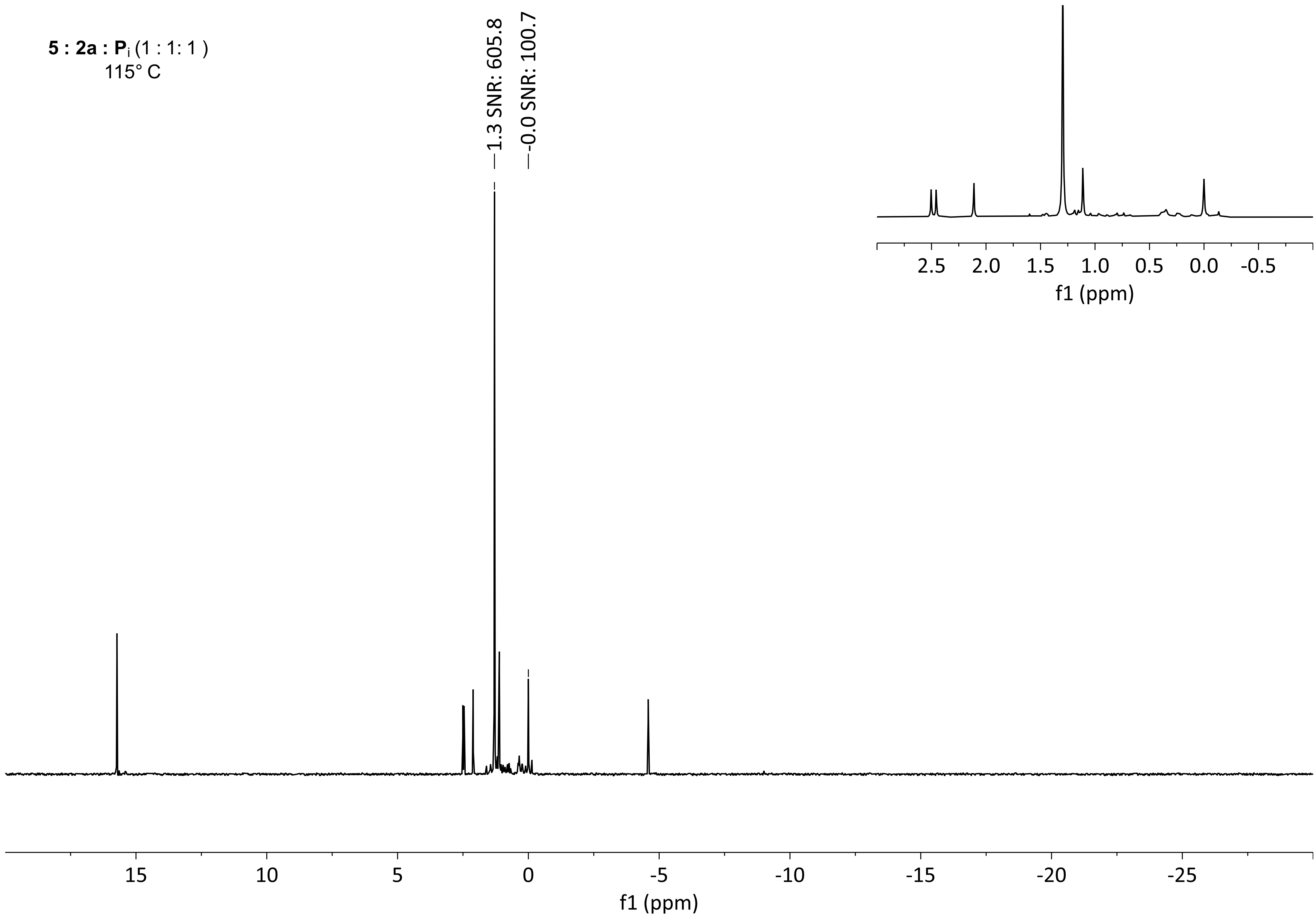
5 : 2a : Pi (1 : 1: 1)
115° C
1.3 SNR: 605.8
-0.0 SNR: 100.7
2.5 2.0 1.5 1.0 0.5 0.0 -0.5
f1 (ppm)
15 10 5 0 -5 -10 -15 -20 -25
f1 (ppm)

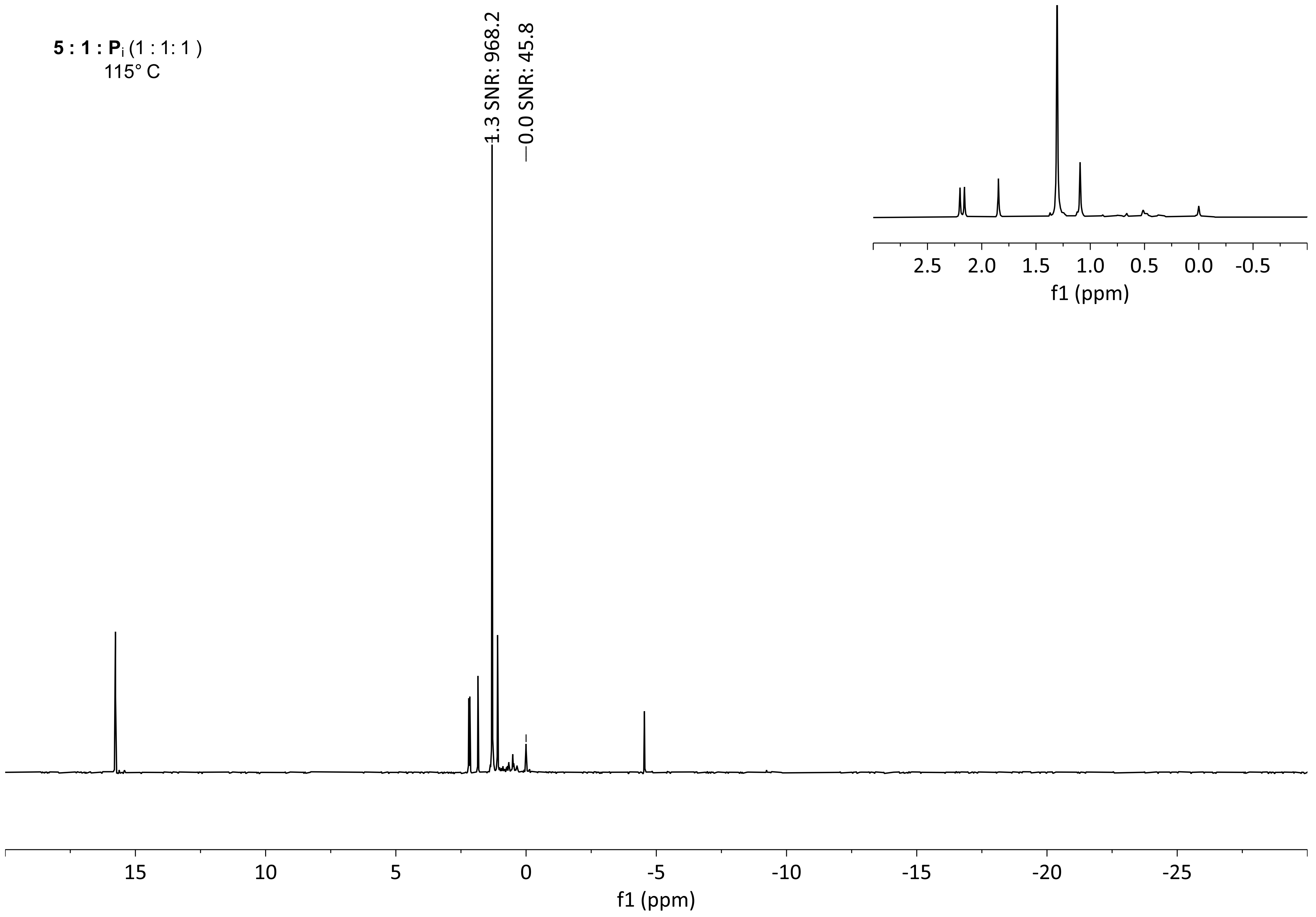

5 : 1 : P_i (1 : 1: 1)
115° C
1.3 SNR: 968.2
0.0 SNR: 45.8
2.5 2.0 1.5 1.0 0.5 0.0 -0.5
f1 (ppm)
15 10 5 0 -5 -10 -15 -20 -25
f1 (ppm)

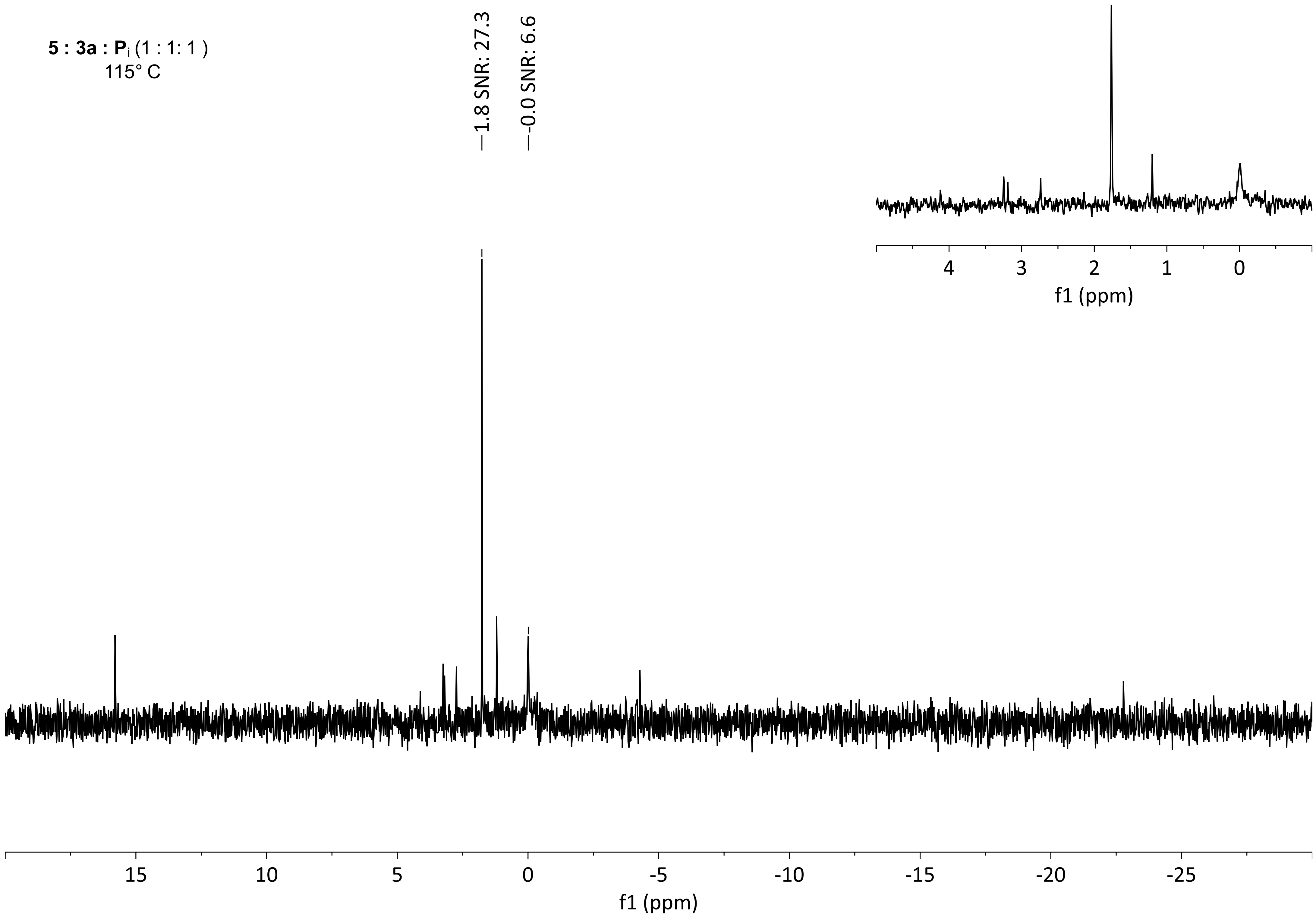

5 : 3a : P$_i$ (1 : 1: 1)
115° C
1.8 SNR: 27.3
-0.0 SNR: 6.6
4
3
2
1
0
f1 (ppm)
15
10
5
0
-5
-10
-15
-20
-25
f1 (ppm)

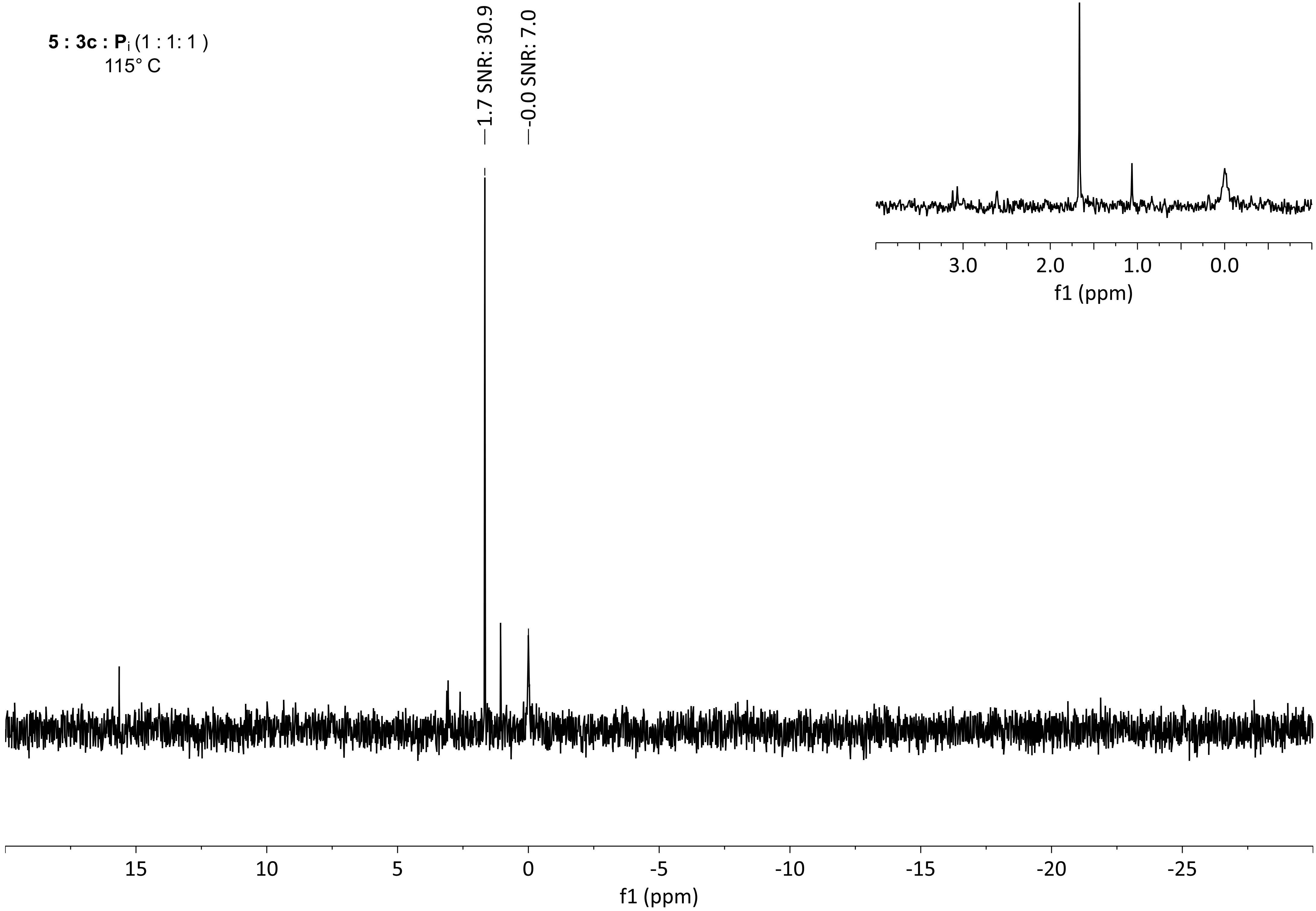
5 : 3c : Pi (1 : 1: 1)
115° C
1.7 SNR: 30.9
-0.0 SNR: 7.0
3.0
2.0
1.0
0.0
f1 (ppm)
15
10
5
0
-5
-10
-15
-20
-25
f1 (ppm)

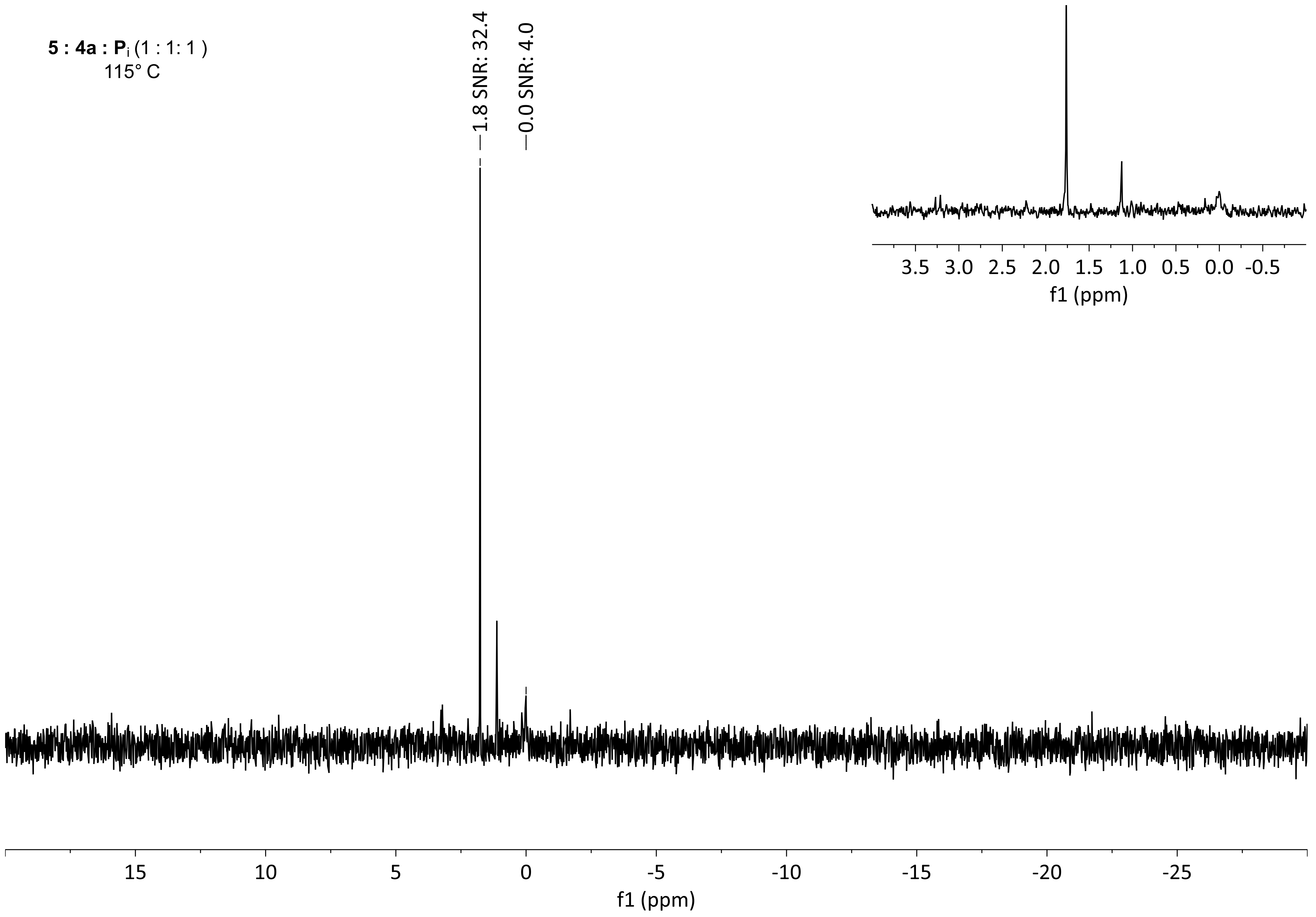

5 : 4a : Pi (1 : 1: 1)
115° C
1.8 SNR: 32.4
0.0 SNR: 4.0
3.5 3.0 2.5 2.0 1.5 1.0 0.5 0.0 -0.5
f1 (ppm)
15 10 5 0 -5 -10 -15 -20 -25
f1 (ppm)

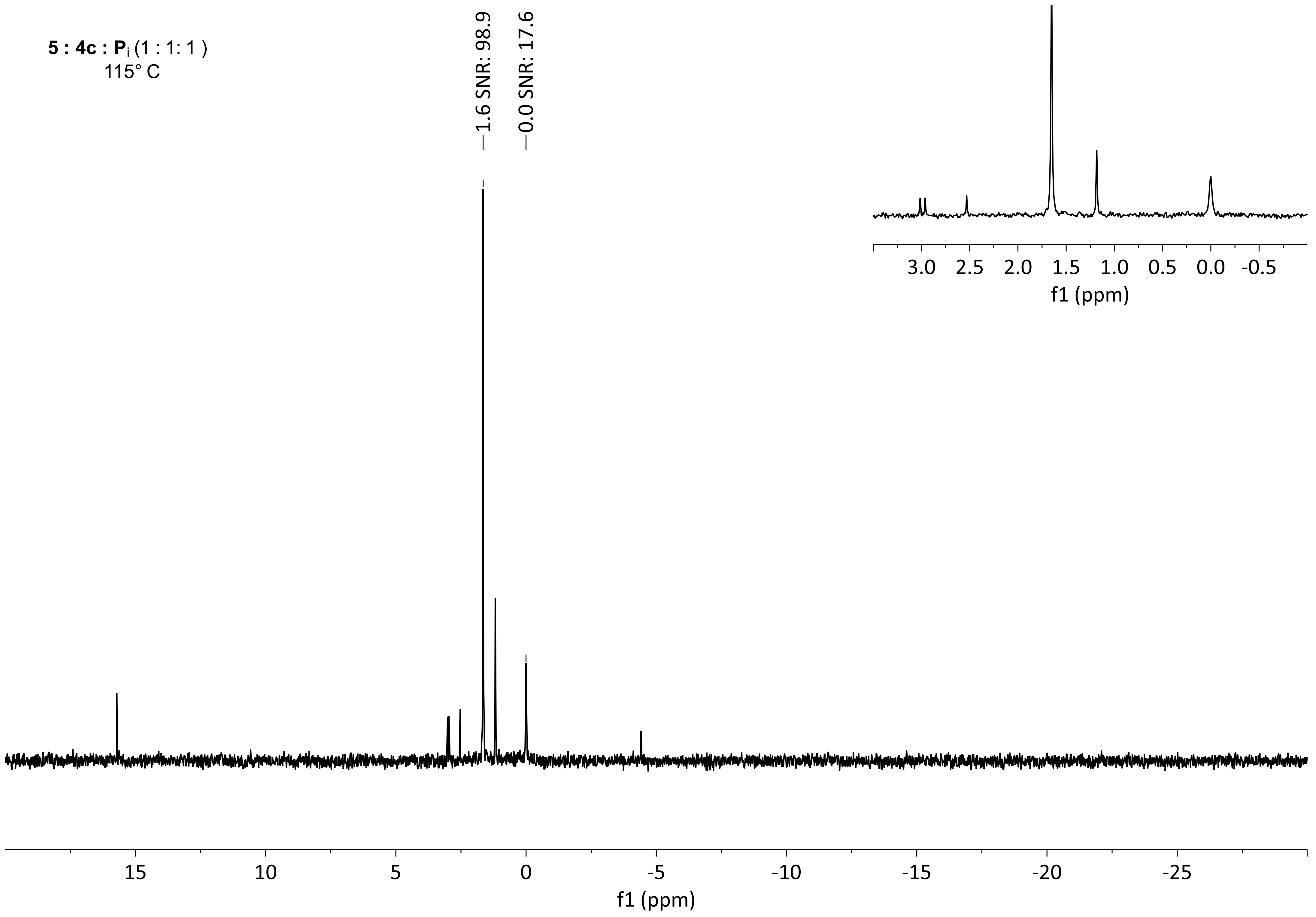
5 : 4c : P$_i$ (1 : 1: 1)
115° C
1.6 SNR: 98.9
0.0 SNR: 17.6
3.0 2.5 2.0 1.5 1.0 0.5 0.0 -0.5
f1 (ppm)
15 10 5 0 -5 -10 -15 -20 -25
f1 (ppm)

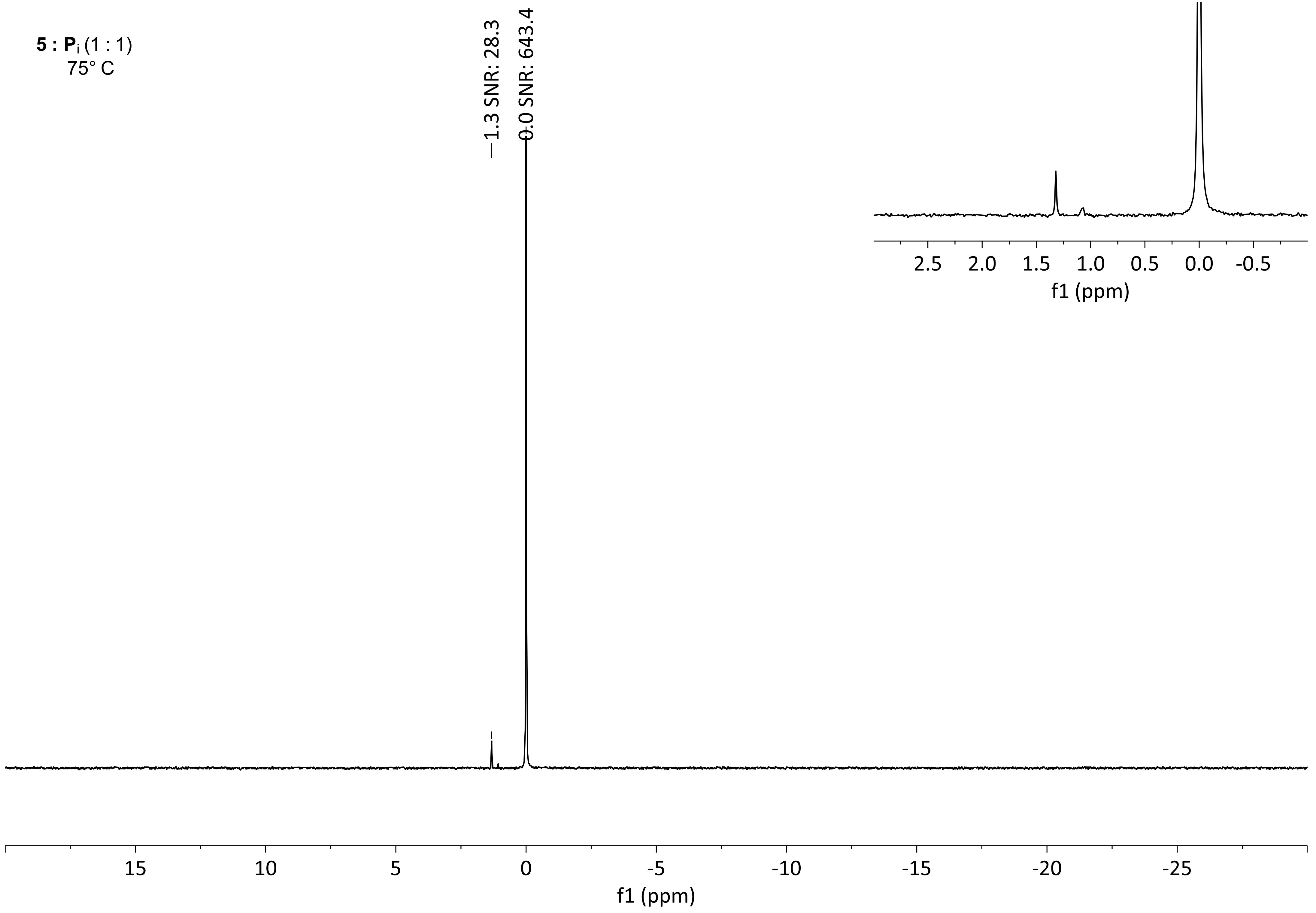

5 : Pi (1 : 1)
75° C
1.3 SNR: 28.3
0.0 SNR: 643.4
2.5 2.0 1.5 1.0 0.5 0.0 -0.5
f1 (ppm)
15 10 5 0 -5 -10 -15 -20 -25
f1 (ppm)

5 : 2a : P$_i$ (1 : 1: 1)
75° C

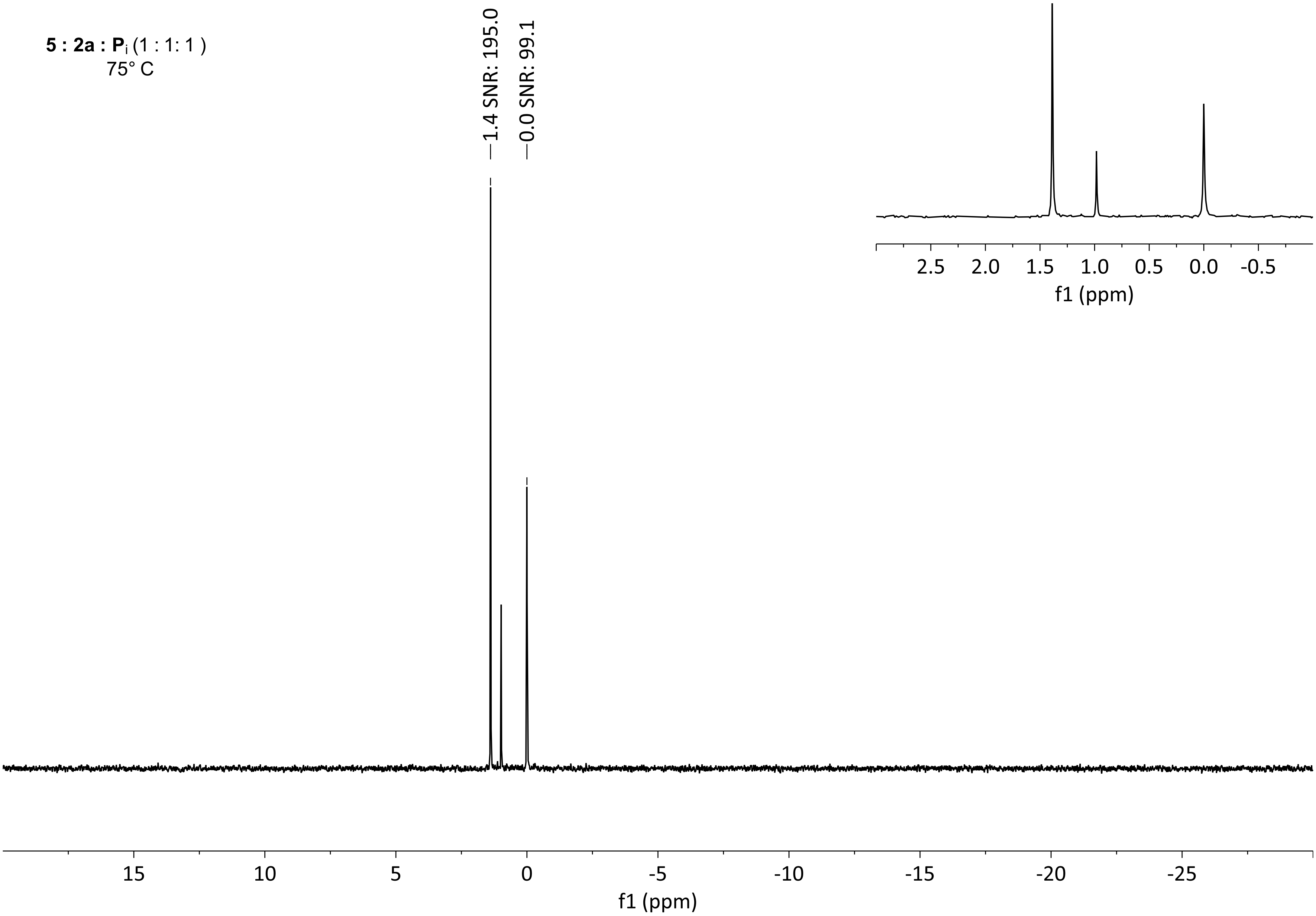

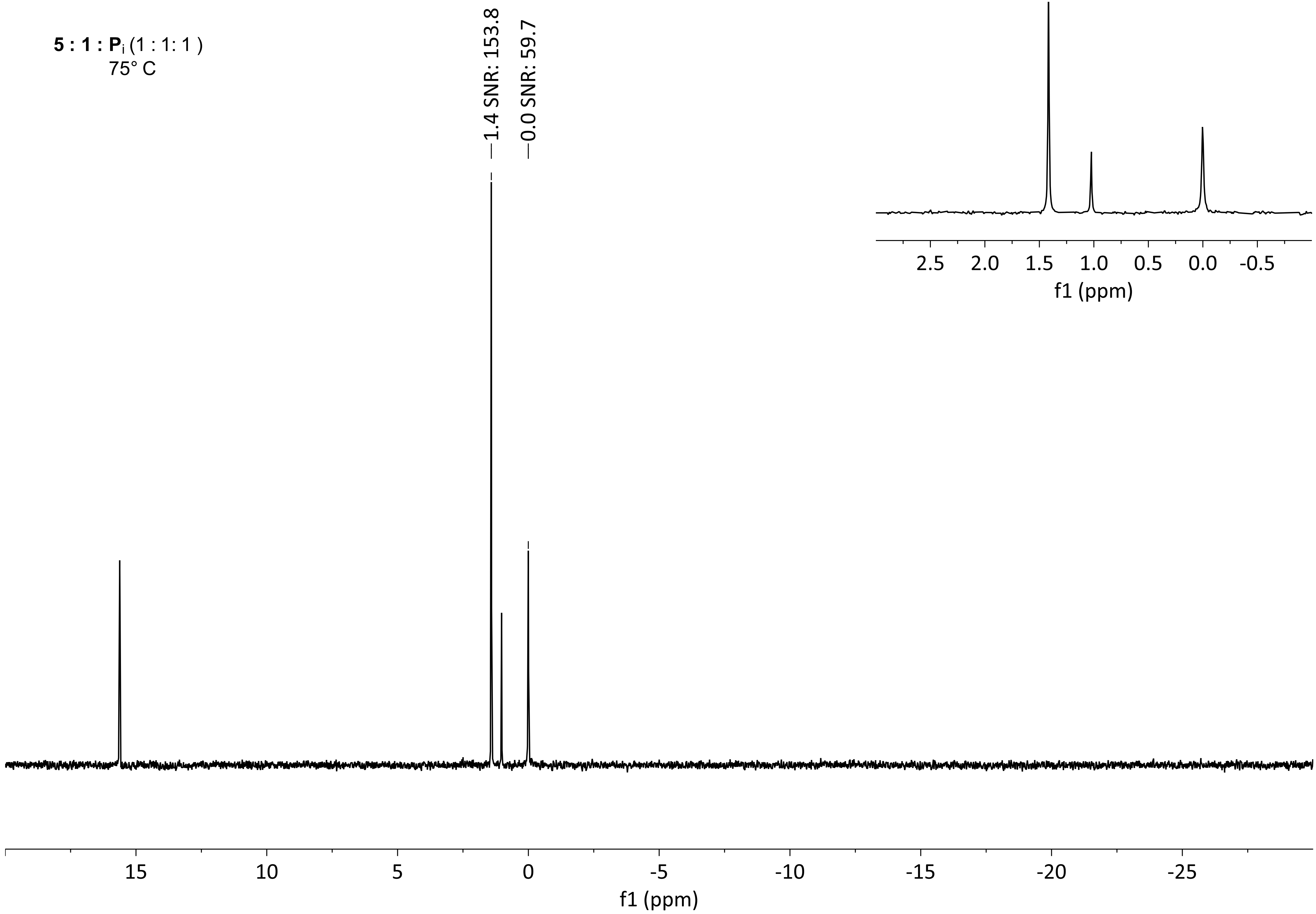

5 : 1 : P i (1 : 1: 1)
75° C
1.4 SNR: 153.8
0.0 SNR: 59.7
2.5 2.0 1.5 1.0 0.5 0.0 -0.5
f1 (ppm)
15 10 5 0 -5 -10 -15 -20 -25
f1 (ppm)

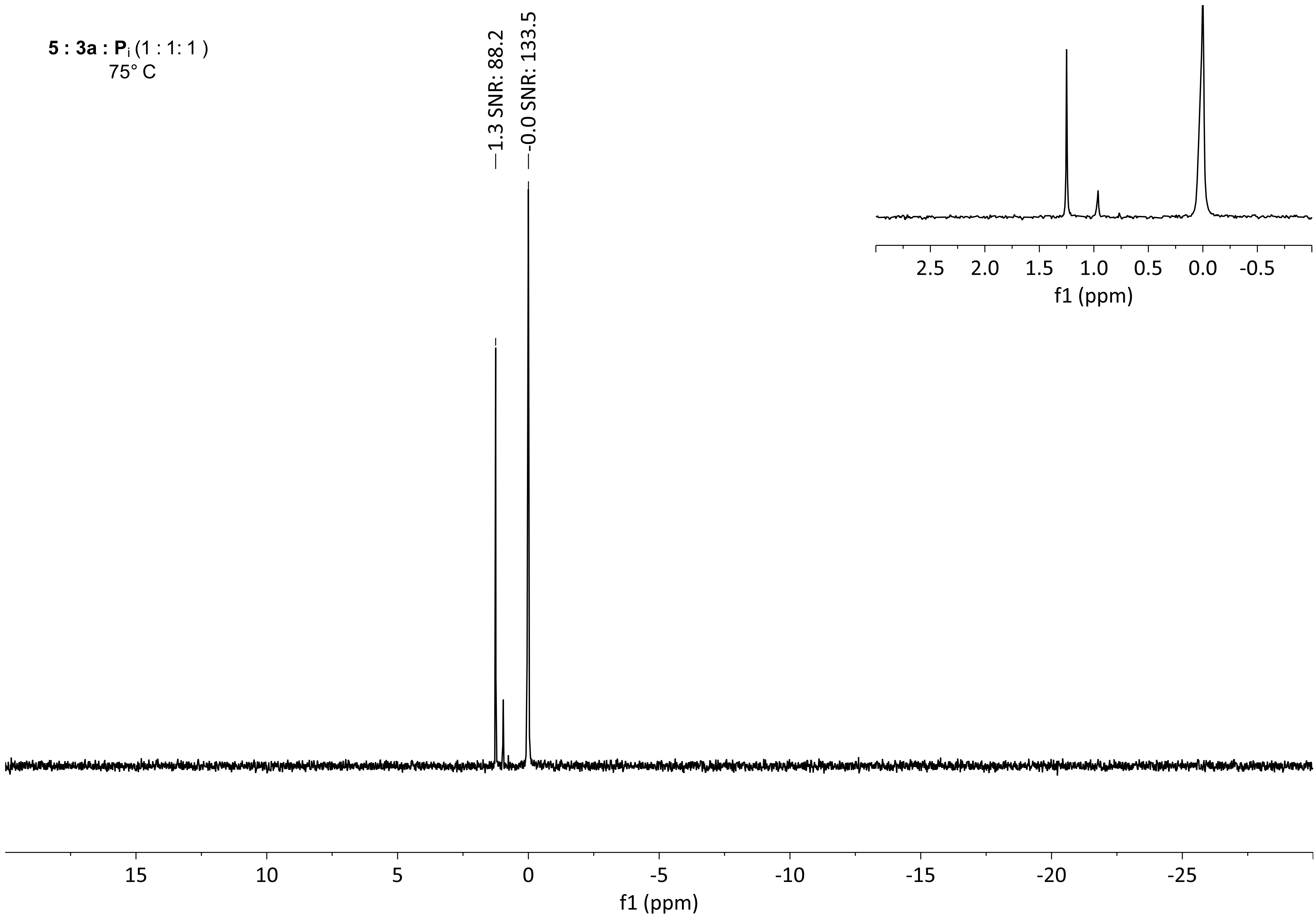

5 : 3a : Pi (1 : 1: 1)
75° C
1.3 SNR: 88.2
-0.0 SNR: 133.5
2.5 2.0 1.5 1.0 0.5 0.0 -0.5
f1 (ppm)
15 10 5 0 -5 -10 -15 -20 -25
f1 (ppm)

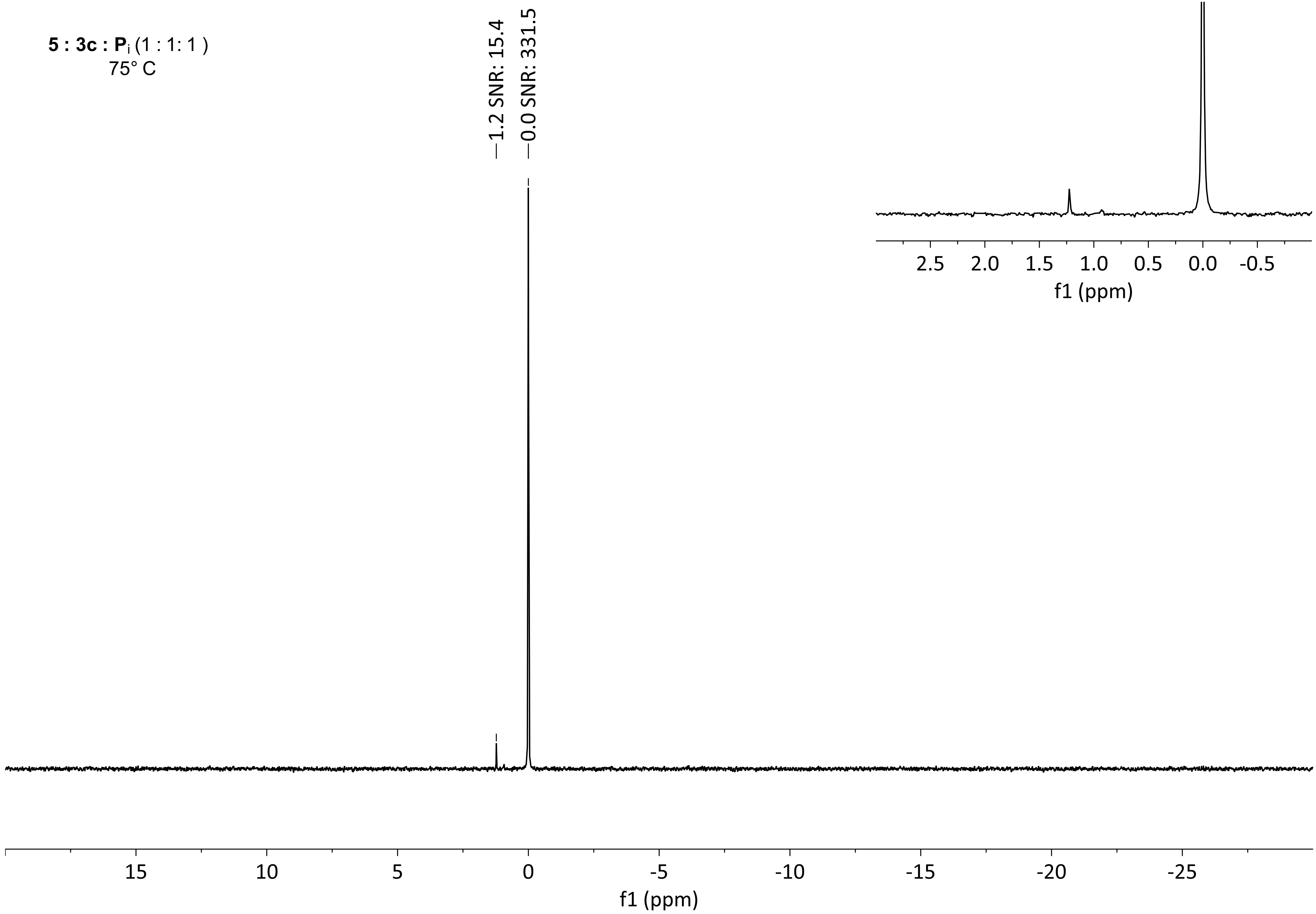

5 : 3c : Pi (1 : 1: 1)
75° C
1.2 SNR: 15.4
0.0 SNR: 331.5
2.5 2.0 1.5 1.0 0.5 0.0 -0.5
f1 (ppm)
15 10 5 0 -5 -10 -15 -20 -25
f1 (ppm)

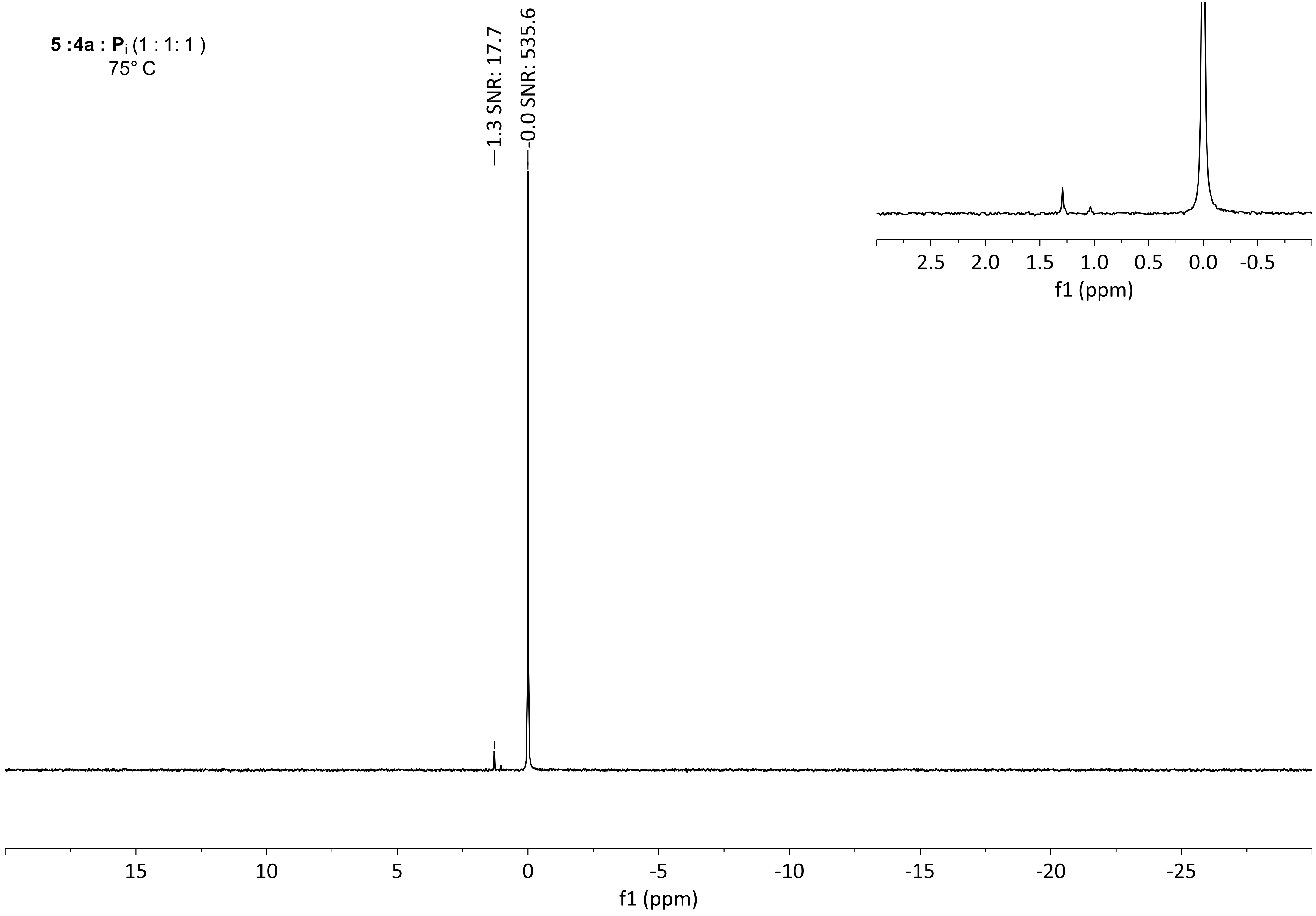
5 :4a : Pi (1 : 1: 1)
75° C
1.3 SNR: 17.7
-0.0 SNR: 535.6
2.5 2.0 1.5 1.0 0.5 0.0 -0.5
f1 (ppm)
15 10 5 0 -5 -10 -15 -20 -25
f1 (ppm)

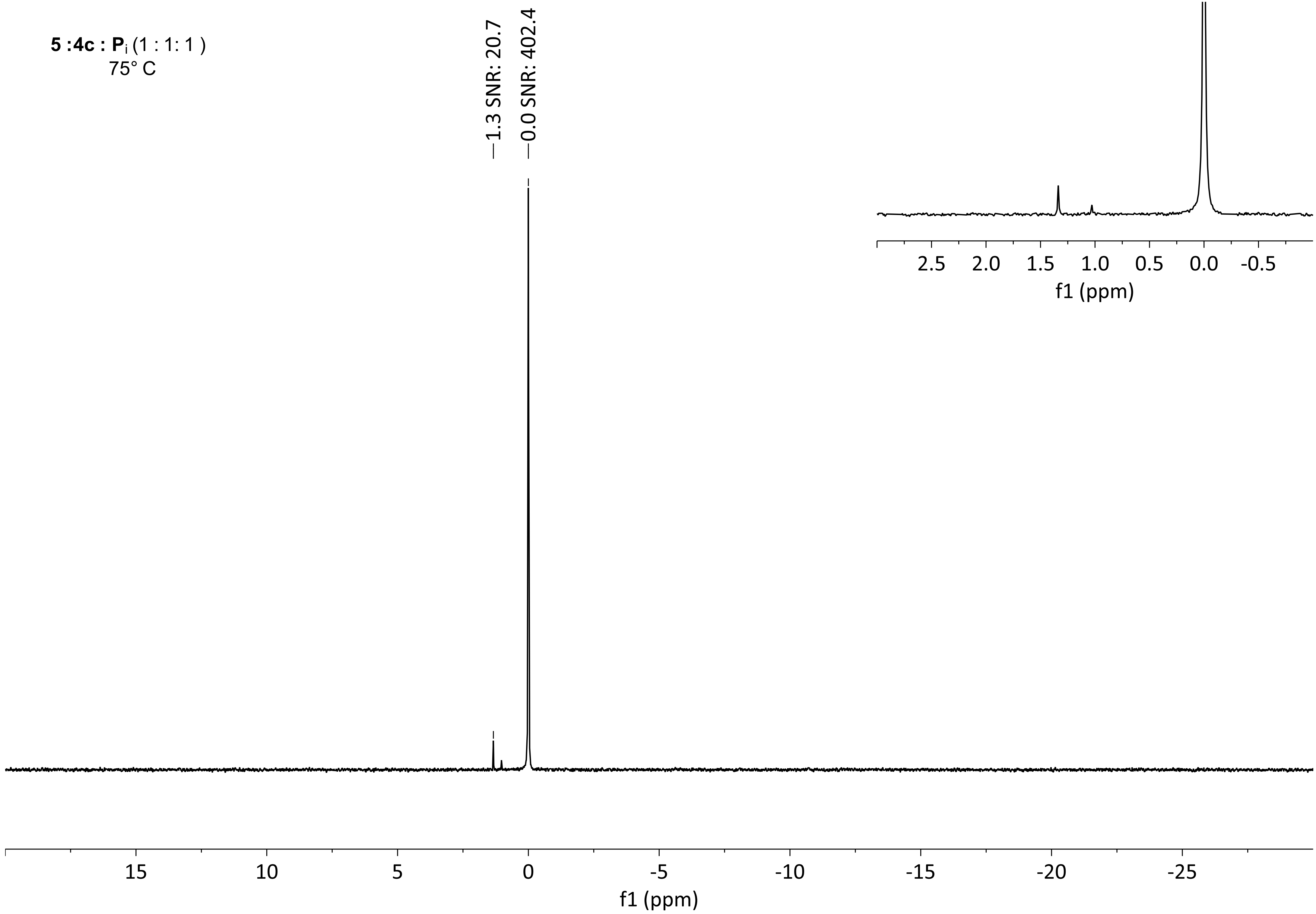

5 :4c : P_i (1 : 1: 1)
75° C
1.3 SNR: 20.7
0.0 SNR: 402.4
2.5 2.0 1.5 1.0 0.5 0.0 -0.5
f1 (ppm)
15 10 5 0 -5 -10 -15 -20 -25
f1 (ppm)
