# Supplementary material for: A scope of prebiotic neat reaction conditions and the mechanism of urea-assisted phosphorylations of alcohols
Source: Nat Commun. 2025 Oct 8;16:8929. doi: 10.1038/s41467-025-63307-3 (PMC12508118; doi:10.1038/s41467-025-63307-3)
Supplement: Supplementary file 4 — Supplementary Data 2 [file 41467_2025_63307_MOESM4_ESM.pdf]

6 : P$_i$ (1 : 1)

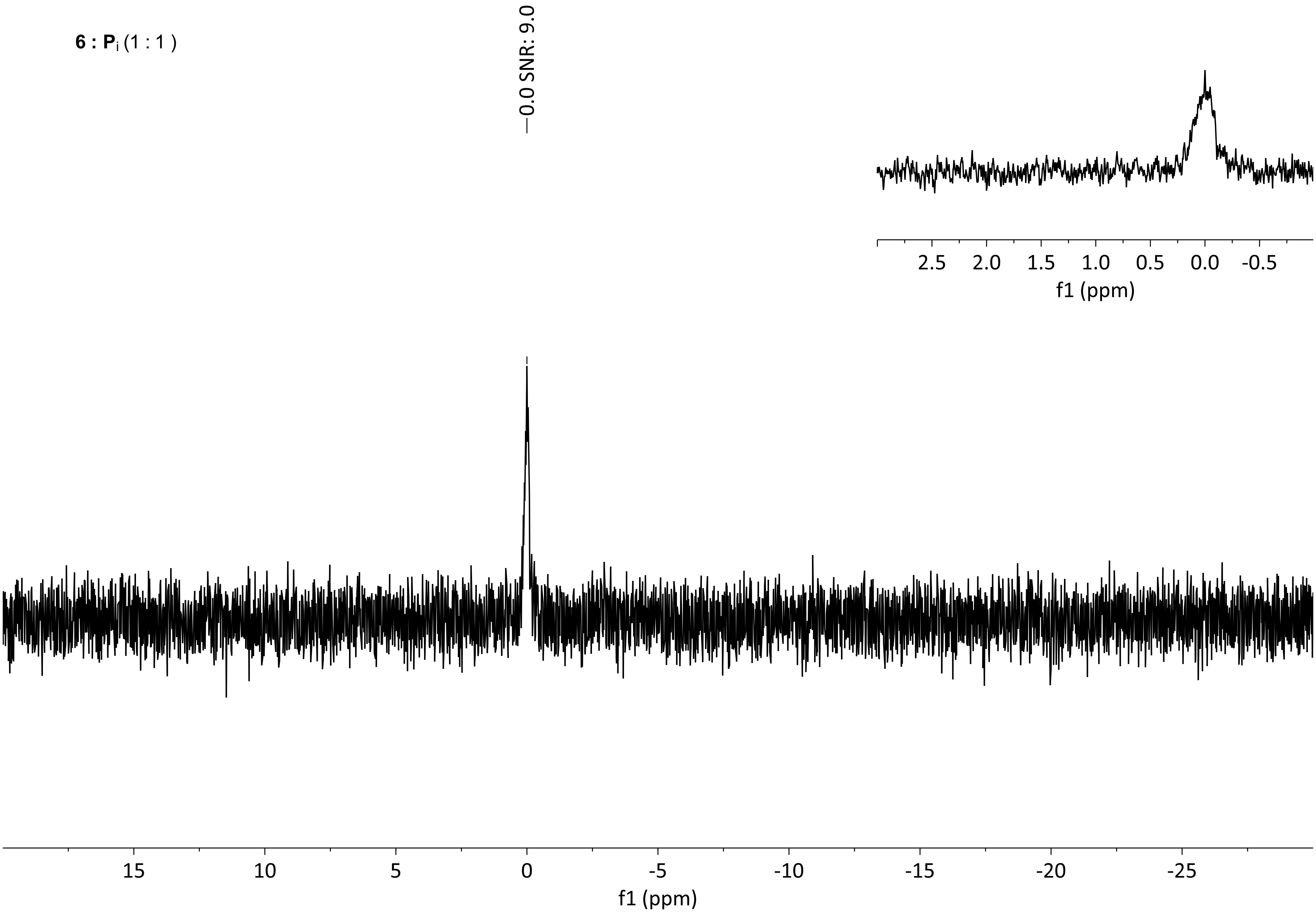

6 : 1 : P$_i$ (1 : 1 : 1)

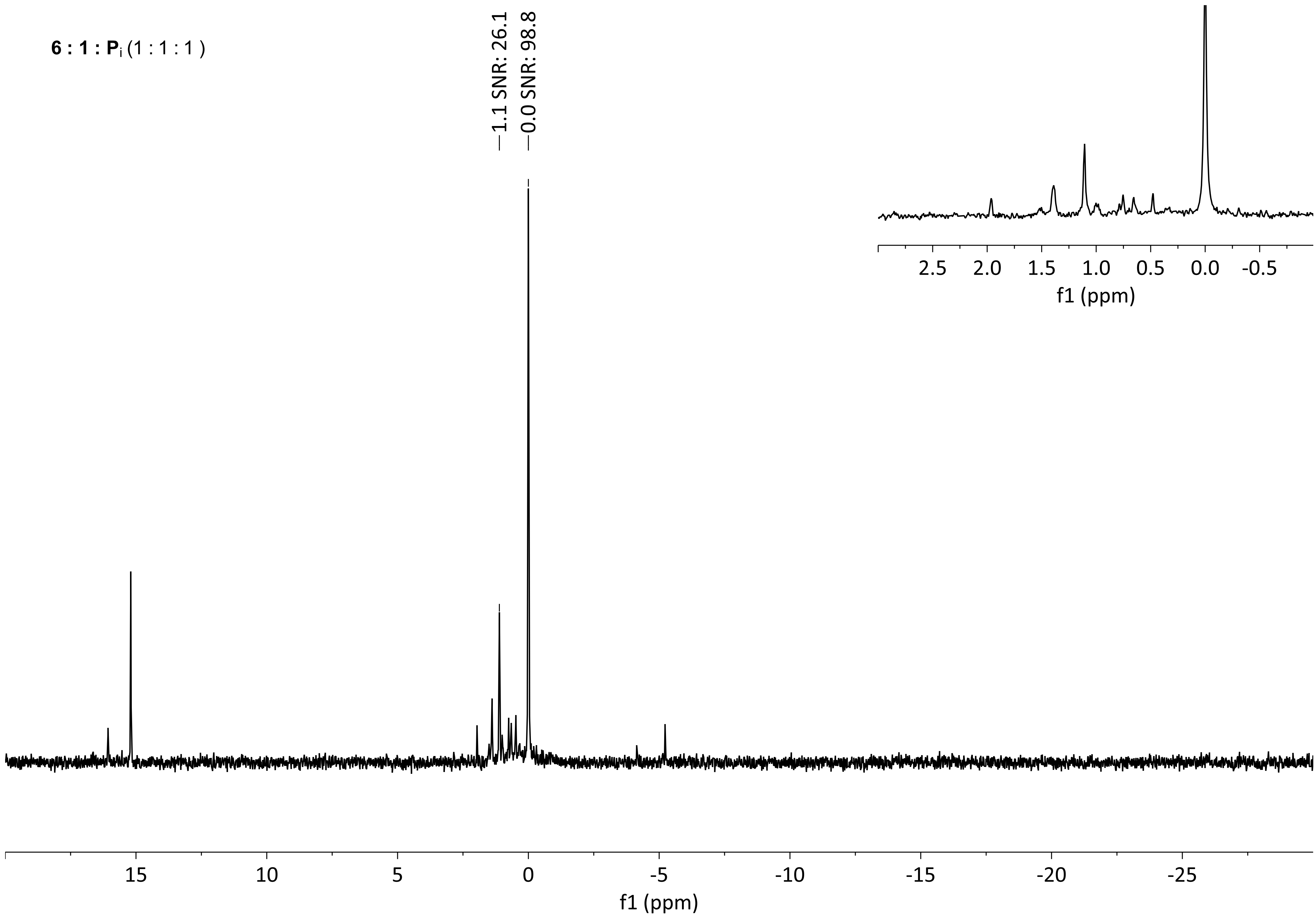

—1.1 SNR: 26.1
—0.0 SNR: 98.8
2.5
2.0
1.5
1.0
0.5
0.0
-0.5
f1 (ppm)
15
10
5
0
-5
-10
-15
-20
-25
f1 (ppm)

6 : 2a : P$_i$ (1 : 1 : 1)

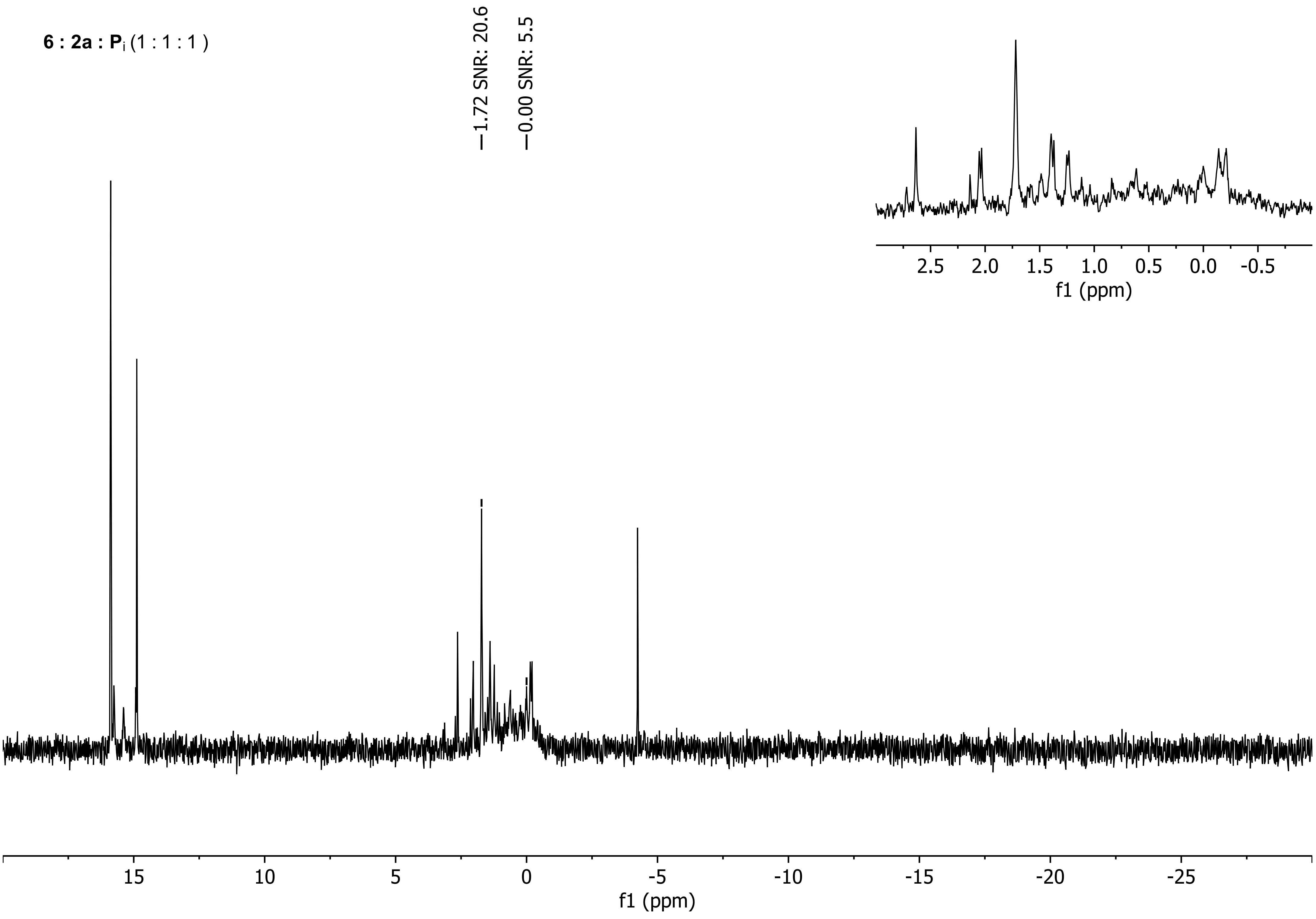

1.72 SNR: 20.6
0.00 SNR: 5.5
2.5 2.0 1.5 1.0 0.5 0.0 -0.5
f1 (ppm)
15 10 5 0 -5 -10 -15 -20 -25
f1 (ppm)

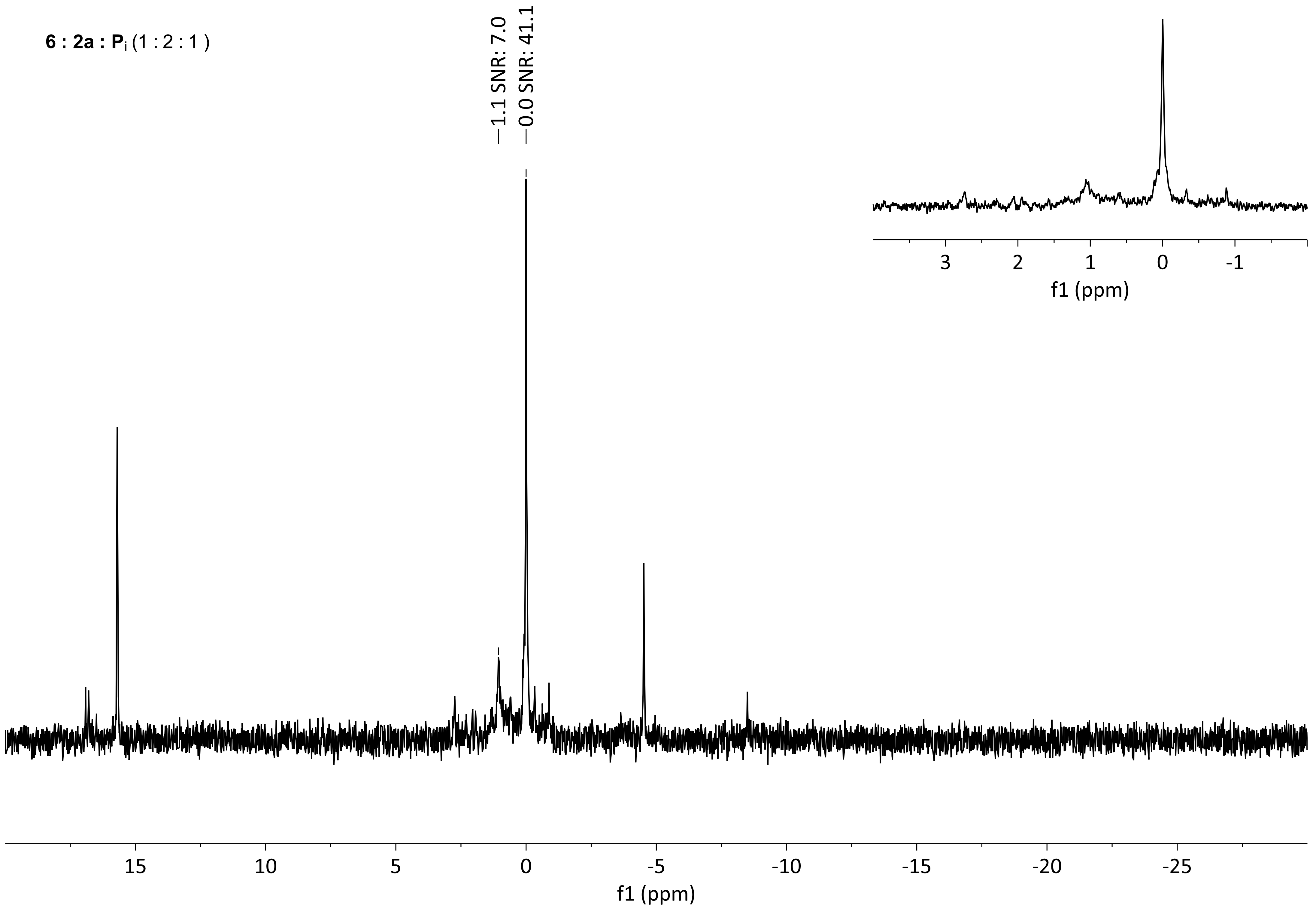

6 : 2a : Pi (1 : 2 : 1)
1.1 SNR: 7.0
0.0 SNR: 41.1
f1 (ppm)
f1 (ppm)

6 : 2a : P$_i$ (1 : 4 : 1)

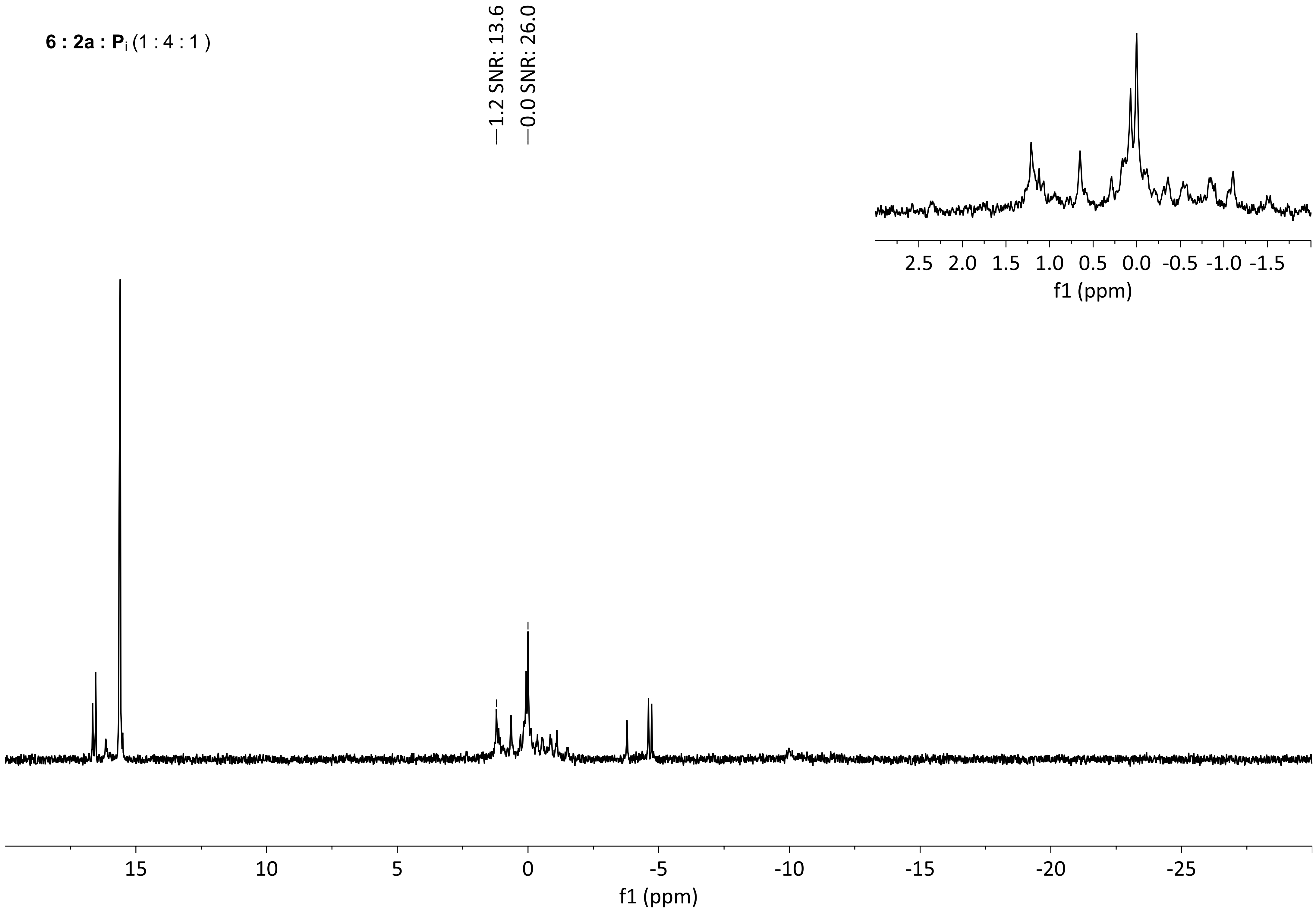

—1.2 SNR: 13.6
—0.0 SNR: 26.0
2.5 2.0 1.5 1.0 0.5 0.0 -0.5 -1.0 -1.5
f1 (ppm)
15 10 5 0 -5 -10 -15 -20 -25
f1 (ppm)

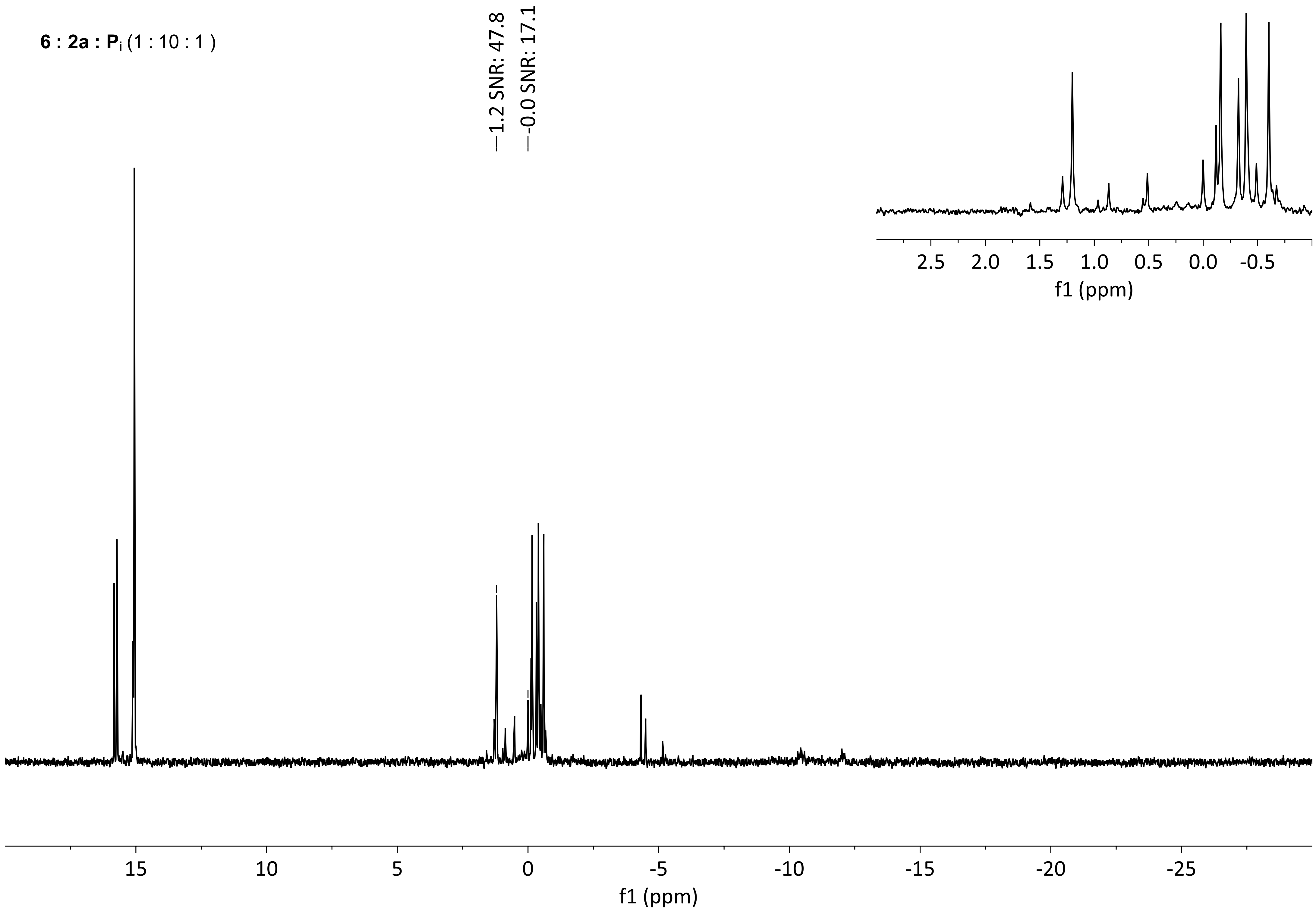
6 : 2a : P$_i$ (1 : 10 : 1)
1.2 SNR: 47.8
-0.0 SNR: 17.1
2.5 2.0 1.5 1.0 0.5 0.0 -0.5
f1 (ppm)
15 10 5 0 -5 -10 -15 -20 -25
f1 (ppm)

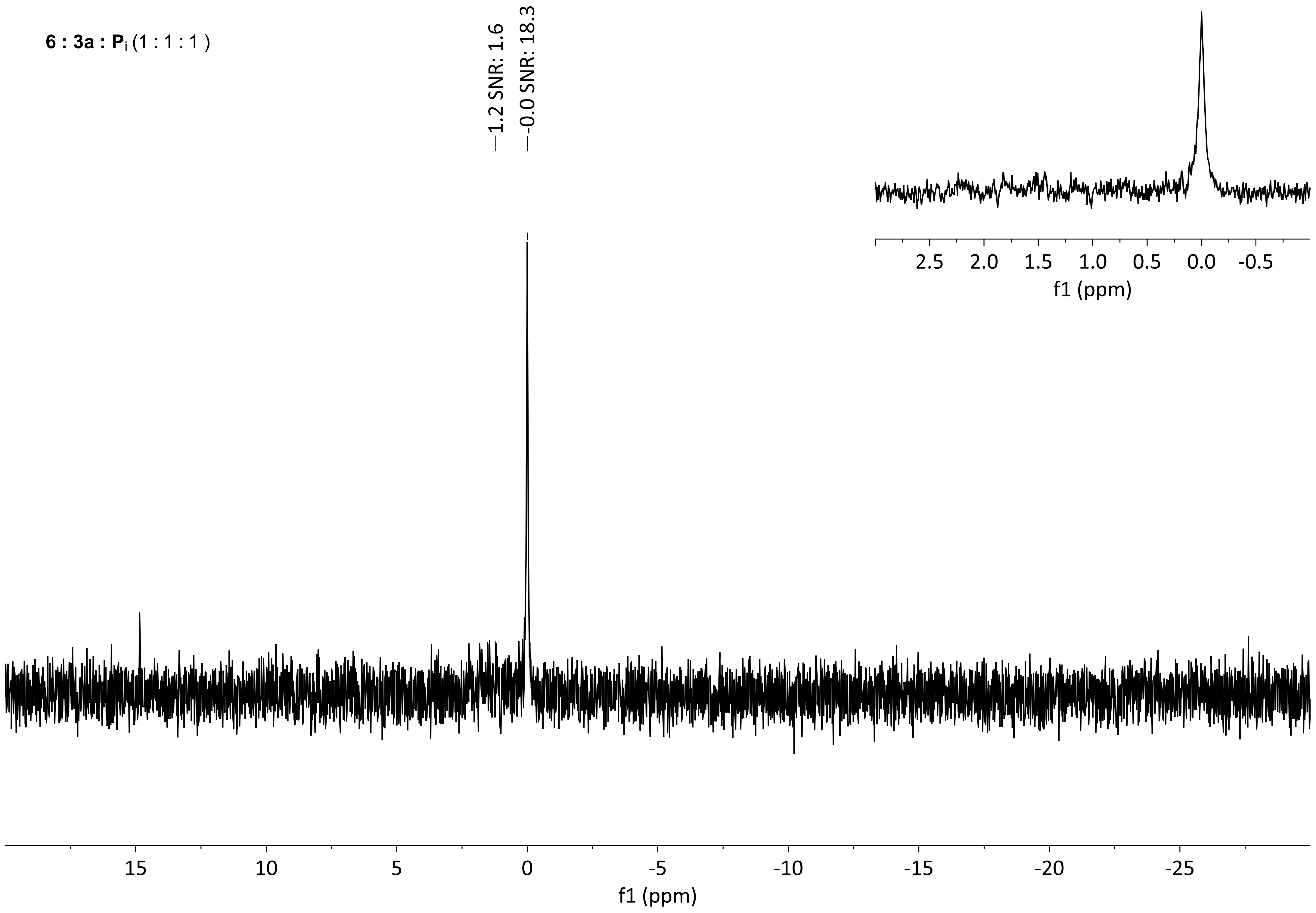
6 : 3a : Pi (1 : 1 : 1)
1.2 SNR: 1.6
-0.0 SNR: 18.3
2.5 2.0 1.5 1.0 0.5 0.0 -0.5
f1 (ppm)
15 10 5 0 -5 -10 -15 -20 -25
f1 (ppm)

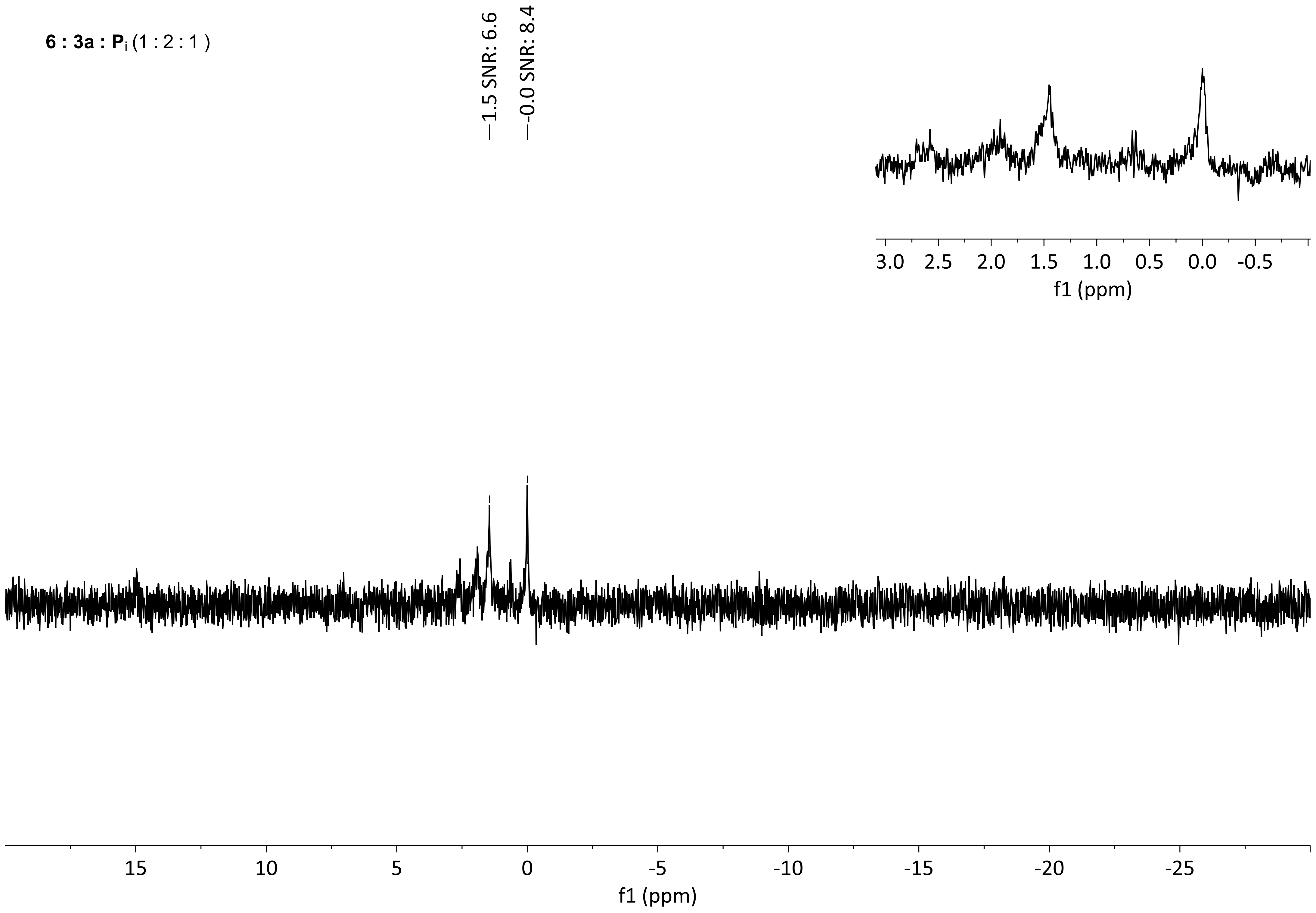
6 : 3a : P$_i$ (1 : 2 : 1)
1.5 SNR: 6.6
-0.0 SNR: 8.4
3.0 2.5 2.0 1.5 1.0 0.5 0.0 -0.5
f1 (ppm)
15 10 5 0 -5 -10 -15 -20 -25
f1 (ppm)

6 : 3a : P$_i$ (1 : 4 : 1)

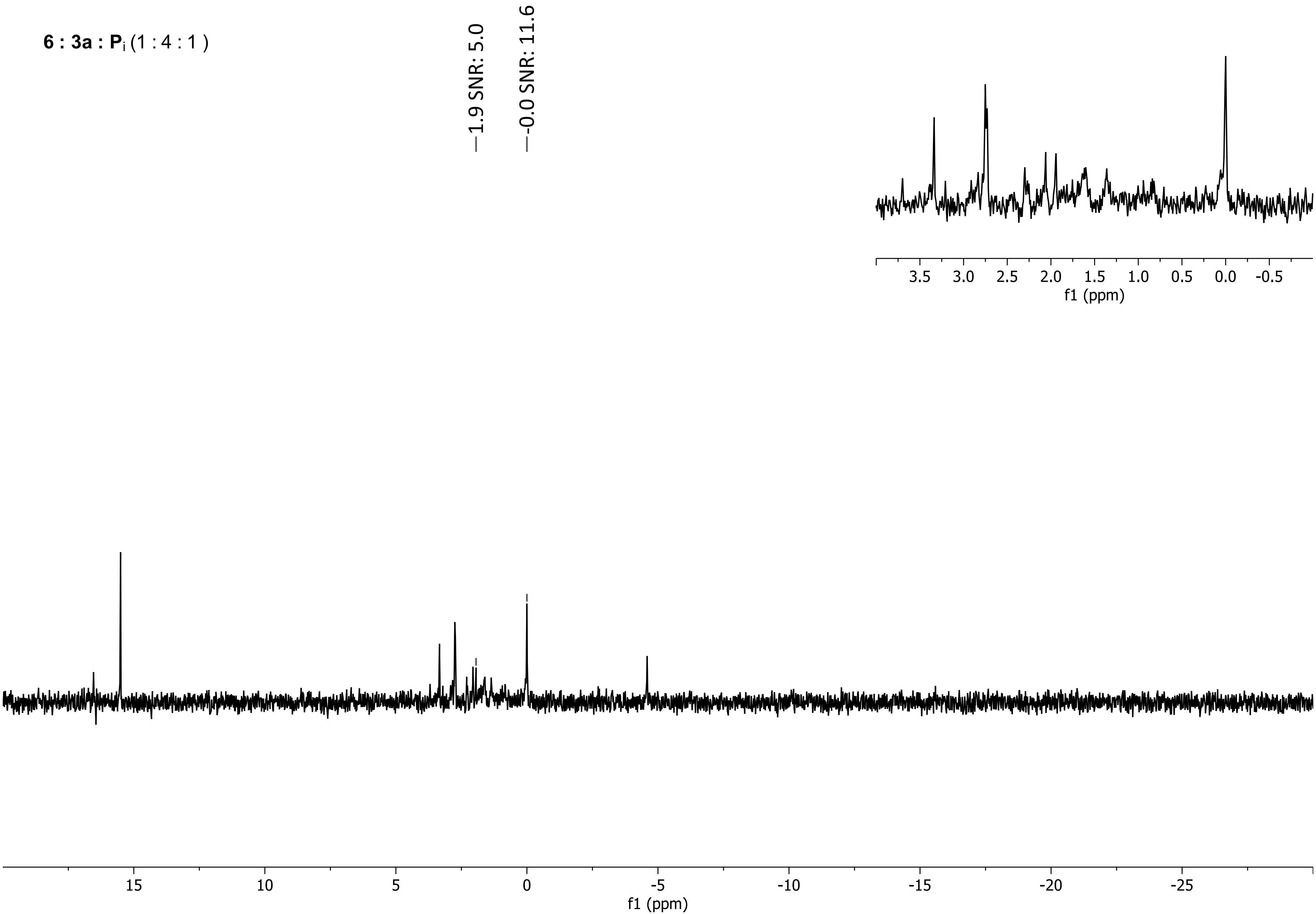

1.9 SNR: 5.0
-0.0 SNR: 11.6
3.5 3.0 2.5 2.0 1.5 1.0 0.5 0.0 -0.5
f1 (ppm)
15 10 5 0 -5 -10 -15 -20 -25
f1 (ppm)

6 : 3c : P$_i$ (1 : 1 : 1)

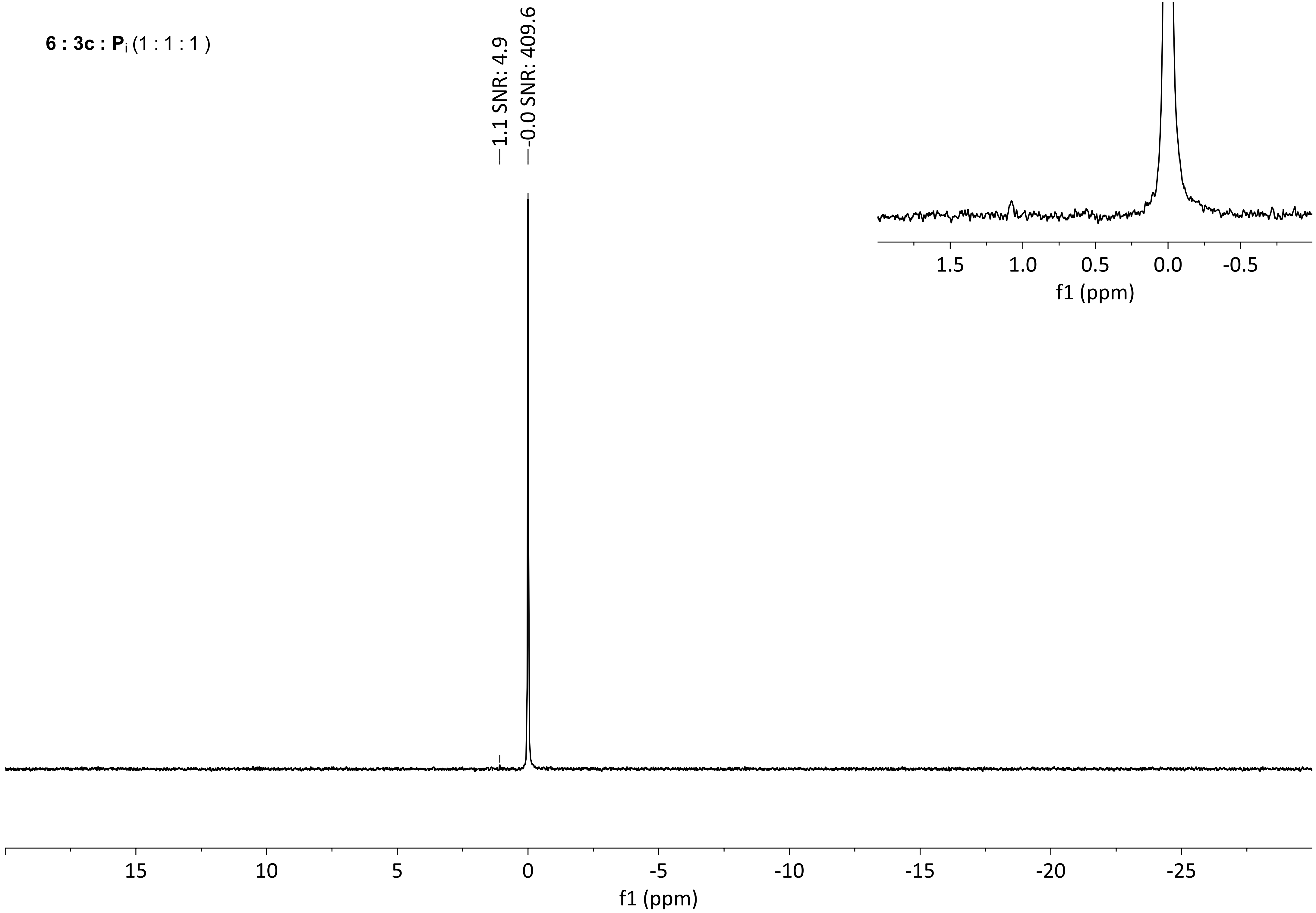

1.1 SNR: 4.9
-0.0 SNR: 409.6
1.5
1.0
0.5
0.0
-0.5
f1 (ppm)
15
10
5
0
-5
-10
-15
-20
-25
f1 (ppm)

6 : 3c : P$_i$ (1 : 2 : 1)

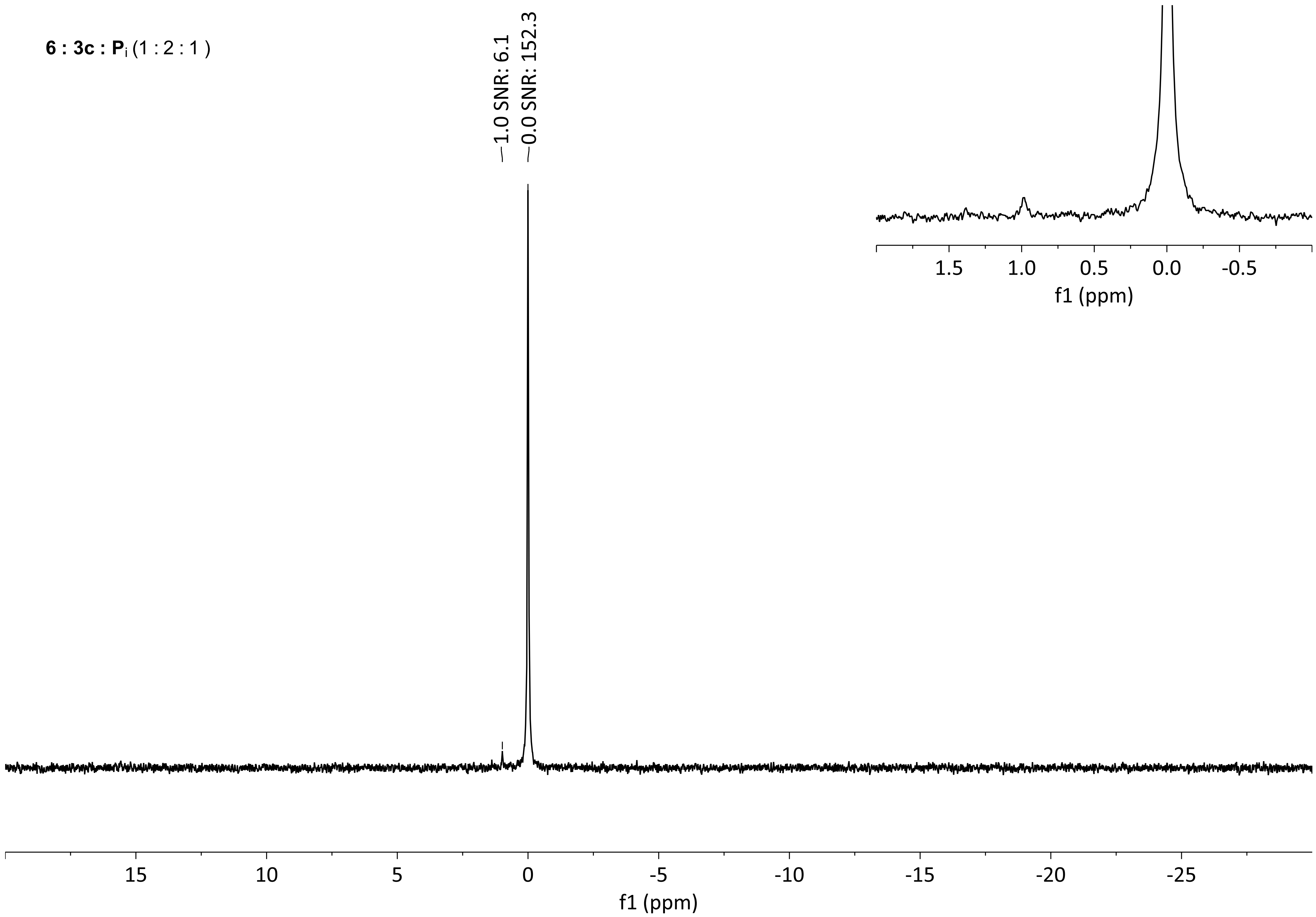

1.0 SNR: 6.1
0.0 SNR: 152.3
1.5
1.0
0.5
0.0
-0.5
f1 (ppm)
15
10
5
0
-5
-10
-15
-20
-25
f1 (ppm)

6 : 3c : P$_i$ (1 : 4 : 1)

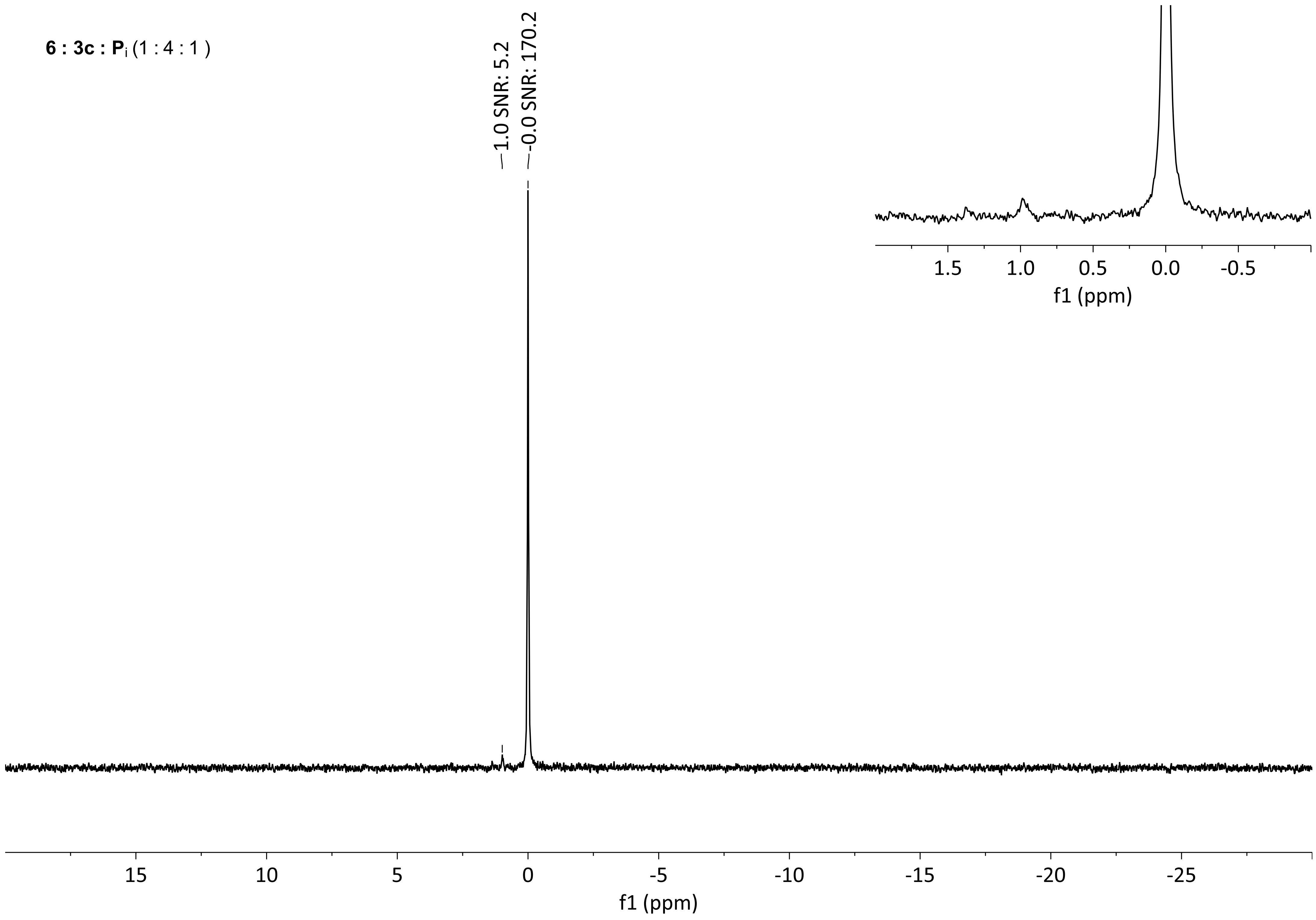
1.0 SNR: 5.2
-0.0 SNR: 170.2
15
10
5
0
-5
-10
-15
-20
-25
f1 (ppm)
1.5
1.0
0.5
0.0
-0.5
f1 (ppm)

6 : 4a : P$_i$ (1 : 1 : 1)

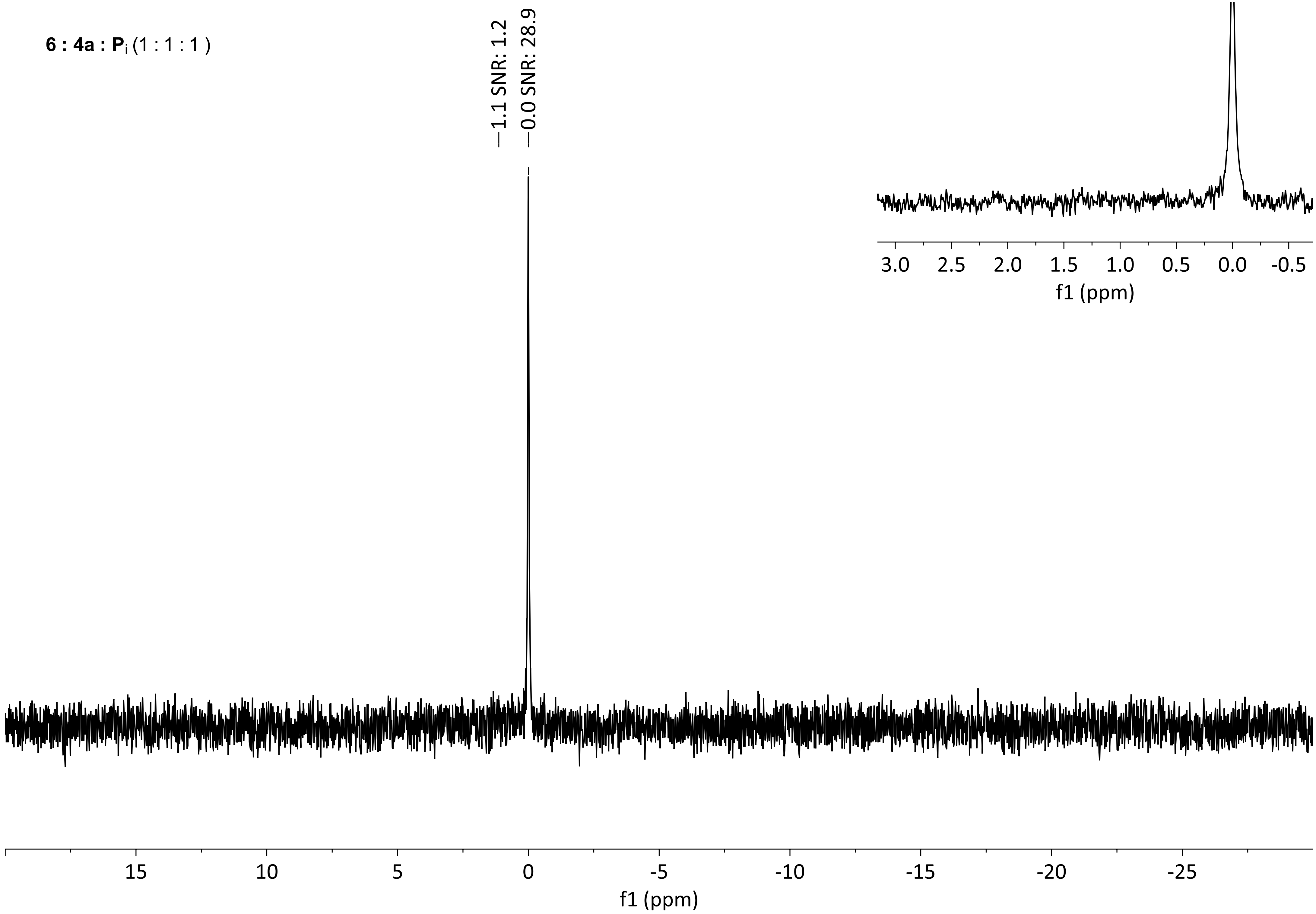

—1.1 SNR: 1.2
—0.0 SNR: 28.9
3.0 2.5 2.0 1.5 1.0 0.5 0.0 -0.5
f1 (ppm)
15 10 5 0 -5 -10 -15 -20 -25
f1 (ppm)

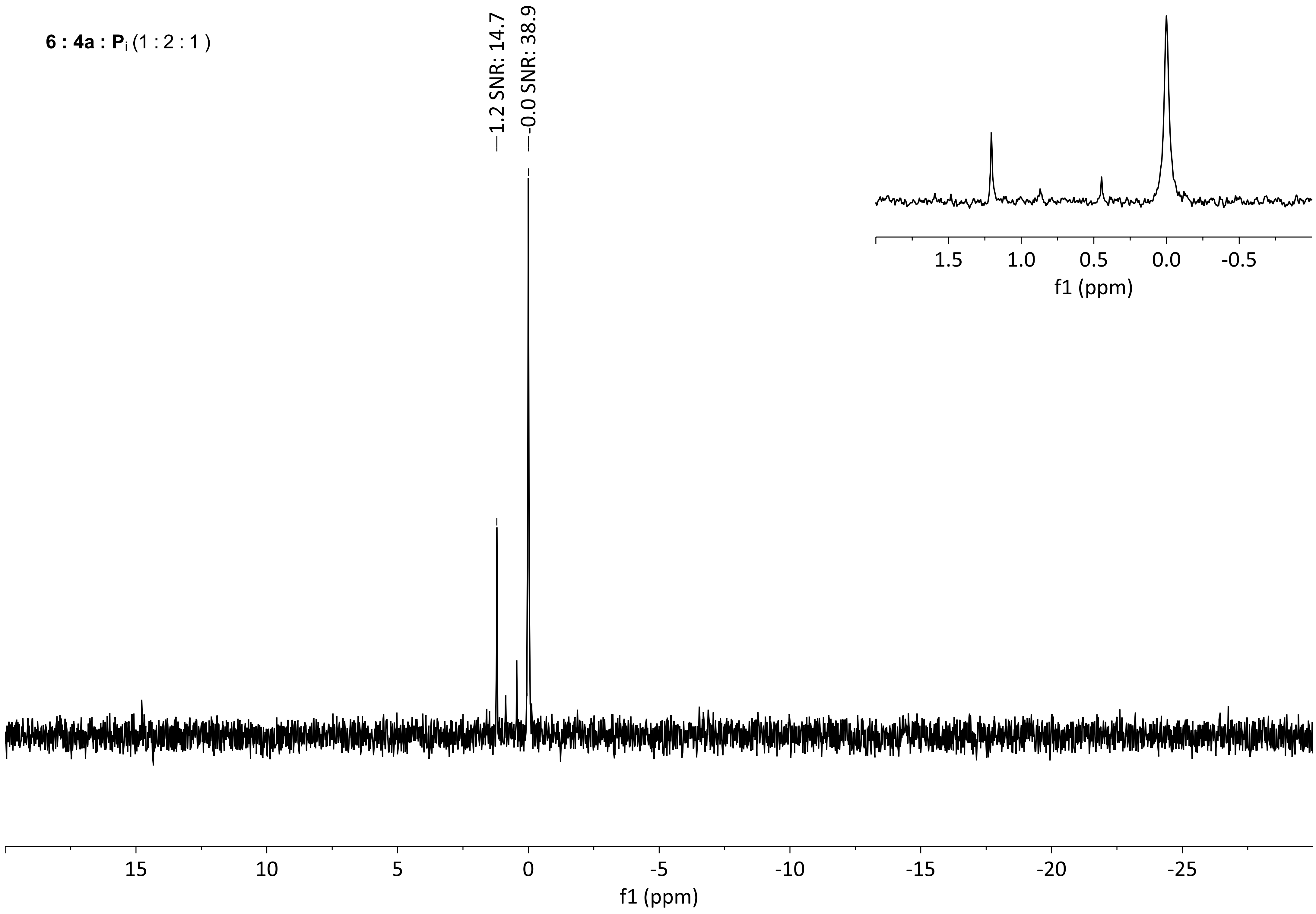
6 : 4a : Pi (1 : 2 : 1)
1.2 SNR: 14.7
-0.0 SNR: 38.9
15
10
5
0
-5
-10
-15
-20
-25
f1 (ppm)
1.5
1.0
0.5
0.0
-0.5
f1 (ppm)

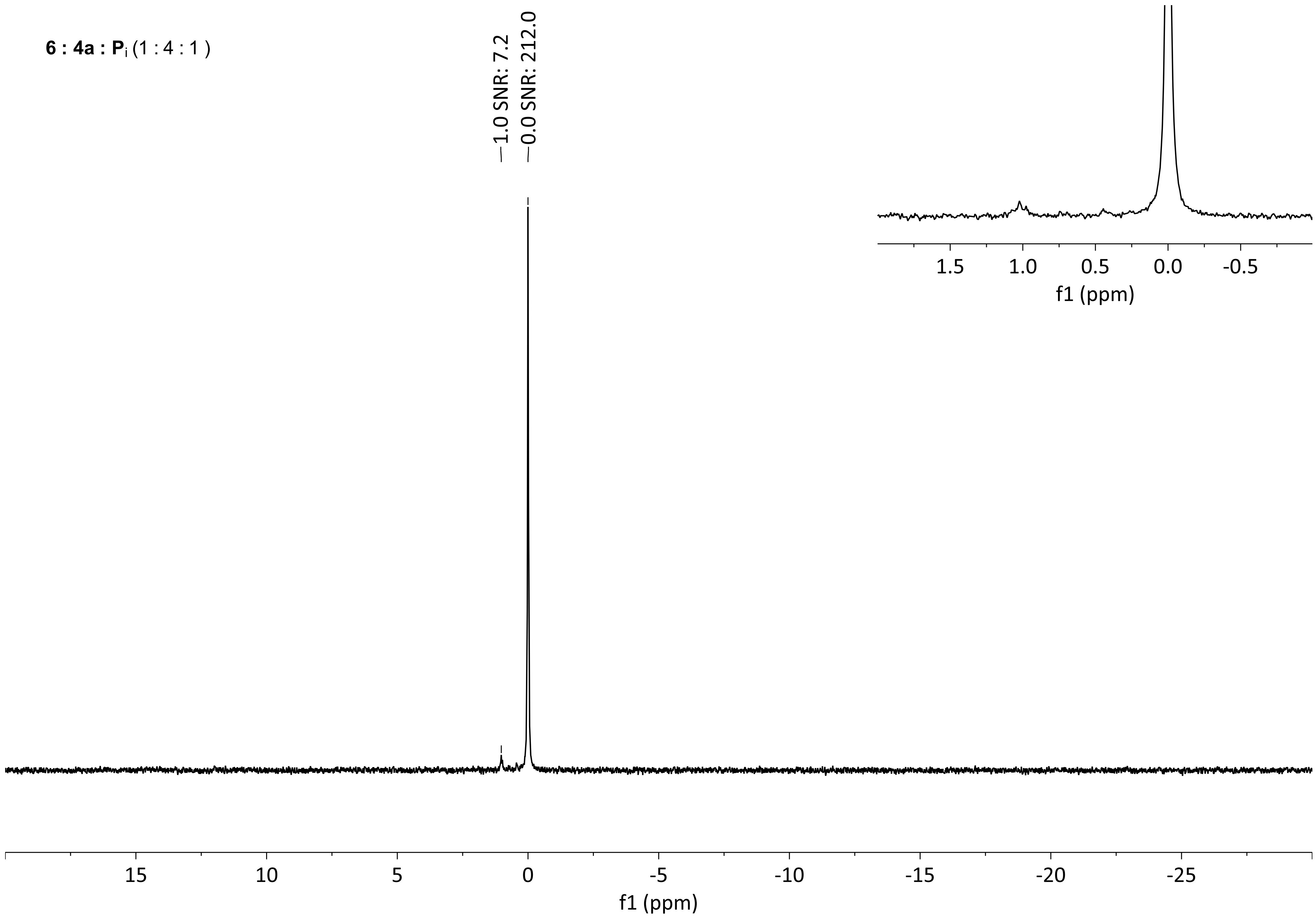

6 : 4a : P$_i$ (1 : 4 : 1)
1.0 SNR: 7.2
0.0 SNR: 212.0
1.5
1.0
0.5
0.0
-0.5
f1 (ppm)
15
10
5
0
-5
-10
-15
-20
-25
f1 (ppm)

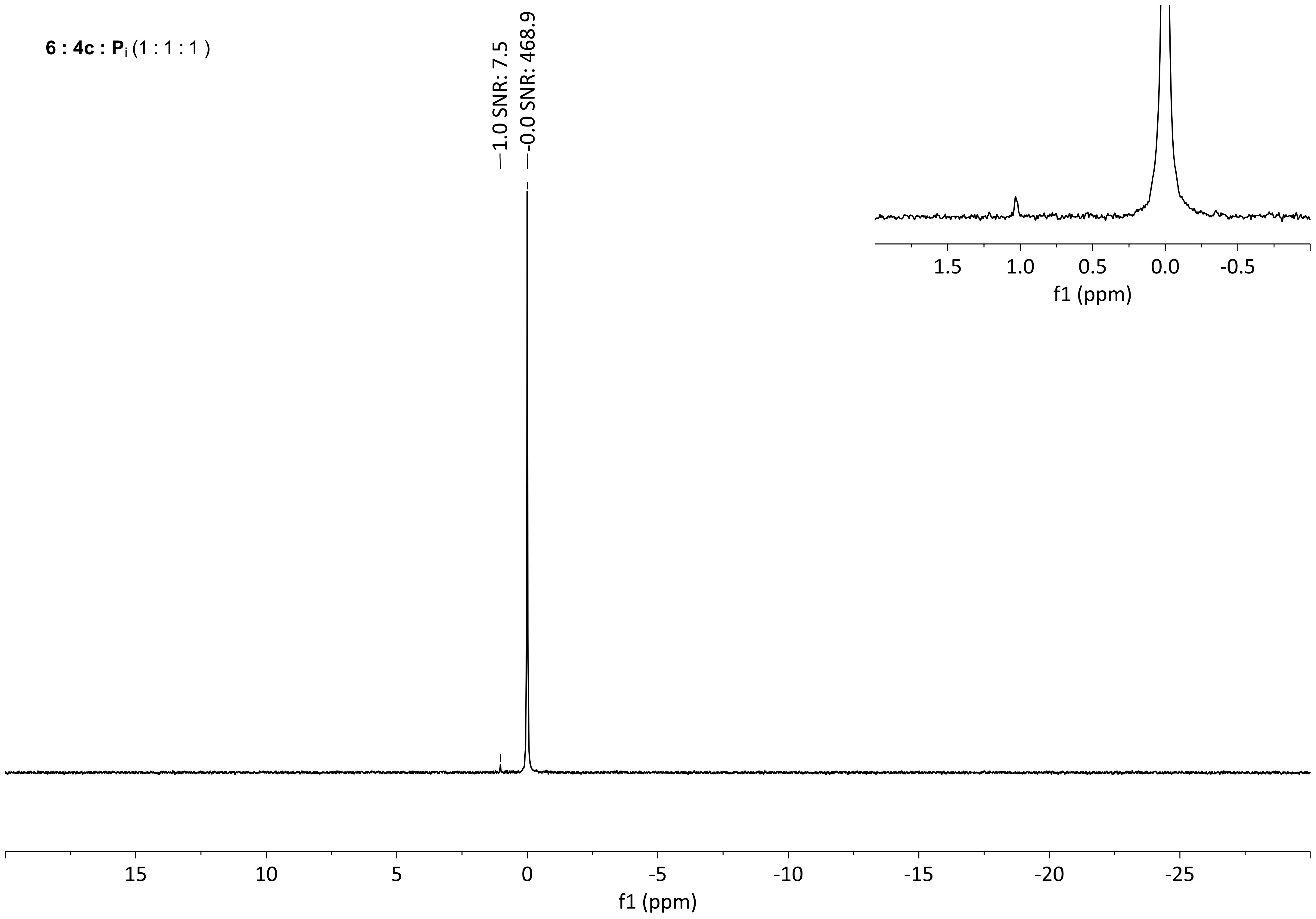

6 : 4c : Pi (1 : 1 : 1)
1.0 SNR: 7.5
-0.0 SNR: 468.9
1.5
1.0
0.5
0.0
-0.5
f1 (ppm)
15
10
5
0
-5
-10
-15
-20
-25
f1 (ppm)

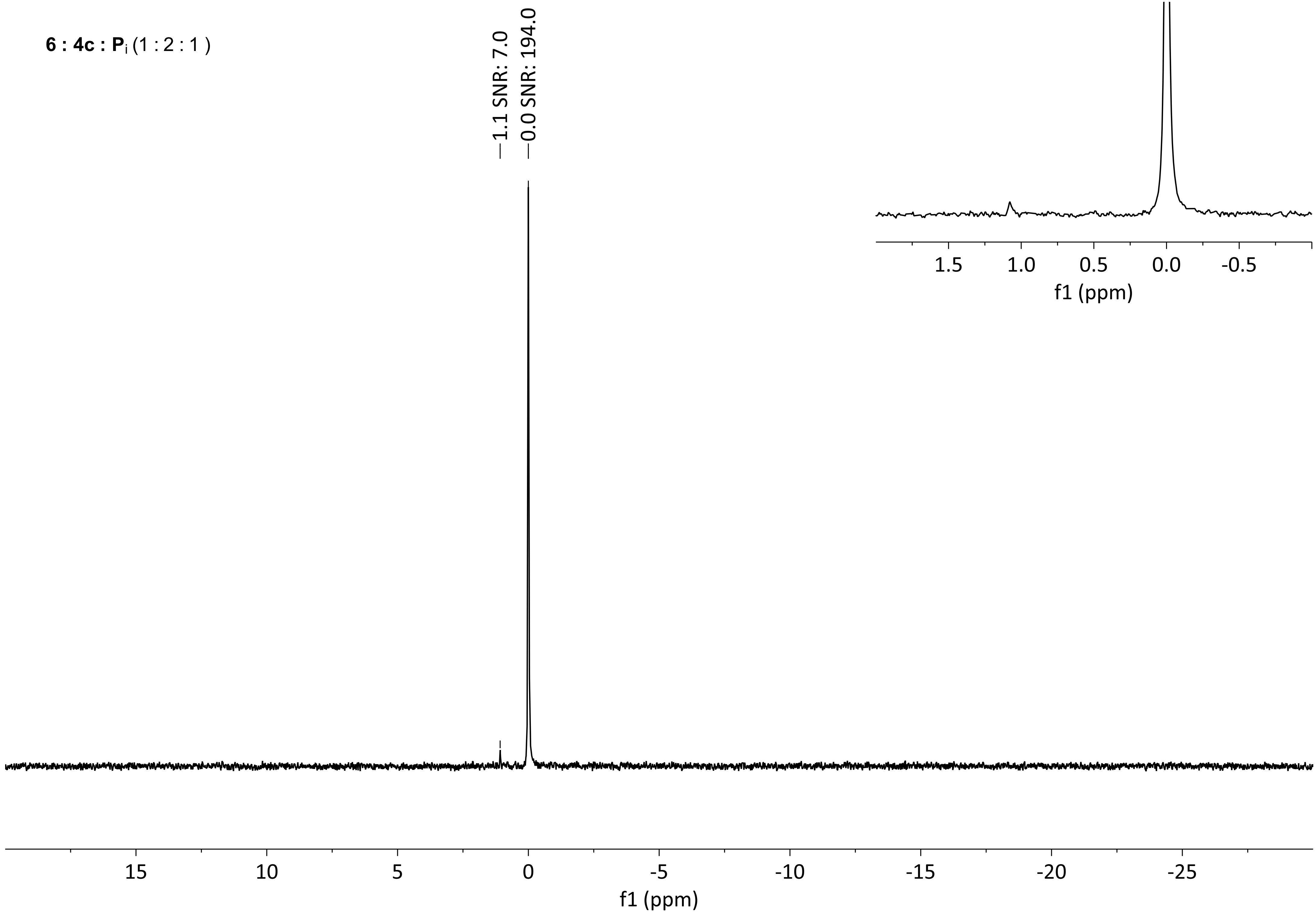
6 : 4c : Pi (1 : 2 : 1)
1.1 SNR: 7.0
0.0 SNR: 194.0
15
10
5
0
-5
-10
-15
-20
-25
f1 (ppm)
1.5
1.0
0.5
0.0
-0.5
f1 (ppm)

6 : 4c : P$_i$ (1 : 4 : 1)

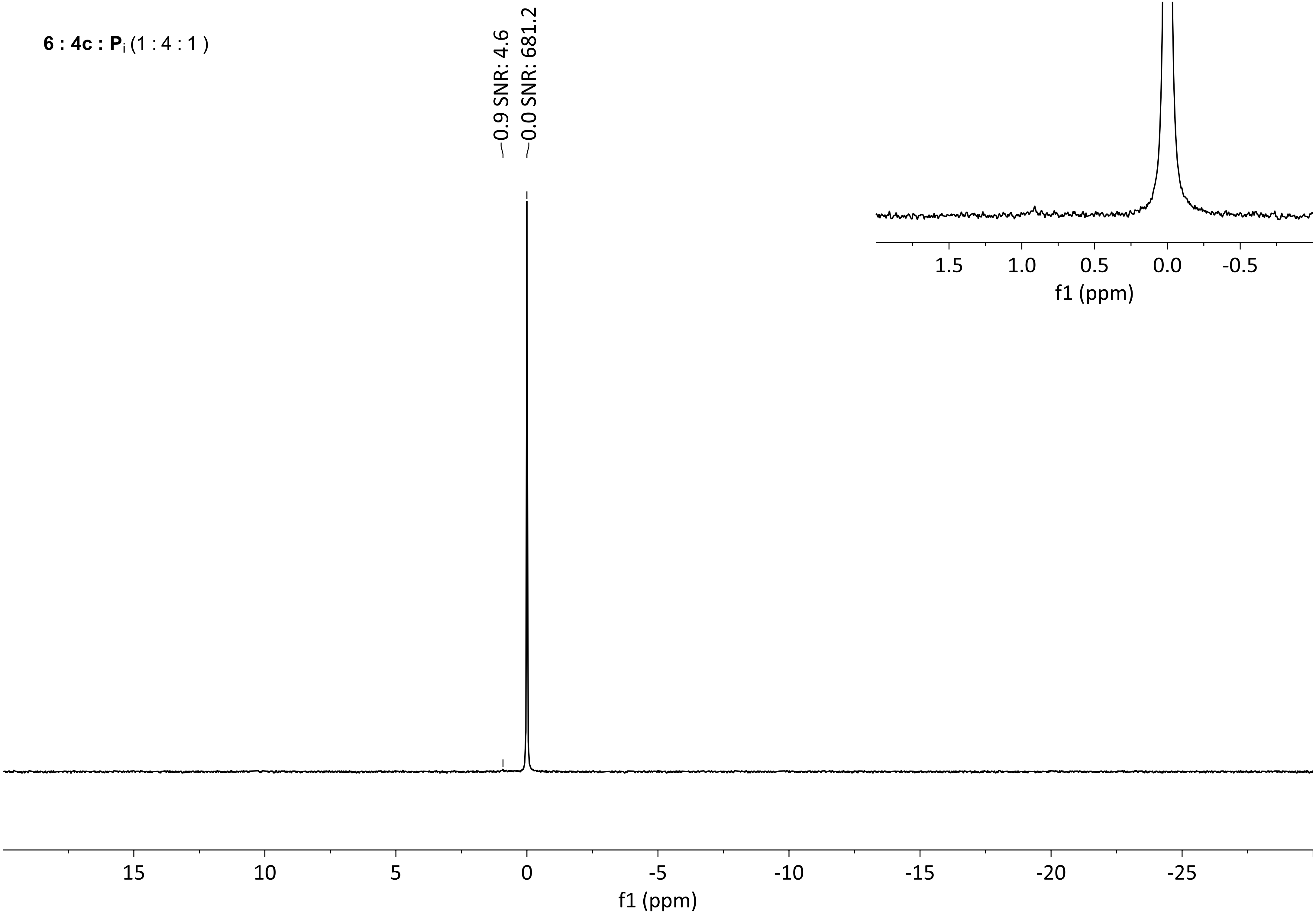
0.9 SNR: 4.6
0.0 SNR: 681.2
1.5
1.0
0.5
0.0
-0.5
f1 (ppm)
15
10
5
0
-5
-10
-15
-20
-25
f1 (ppm)
