# Supplementary material for: A scope of prebiotic neat reaction conditions and the mechanism of urea-assisted phosphorylations of alcohols
Source: Nat Commun. 2025 Oct 8;16:8929. doi: 10.1038/s41467-025-63307-3 (PMC12508118; doi:10.1038/s41467-025-63307-3)

6 : P$_i$ (1 : 1)

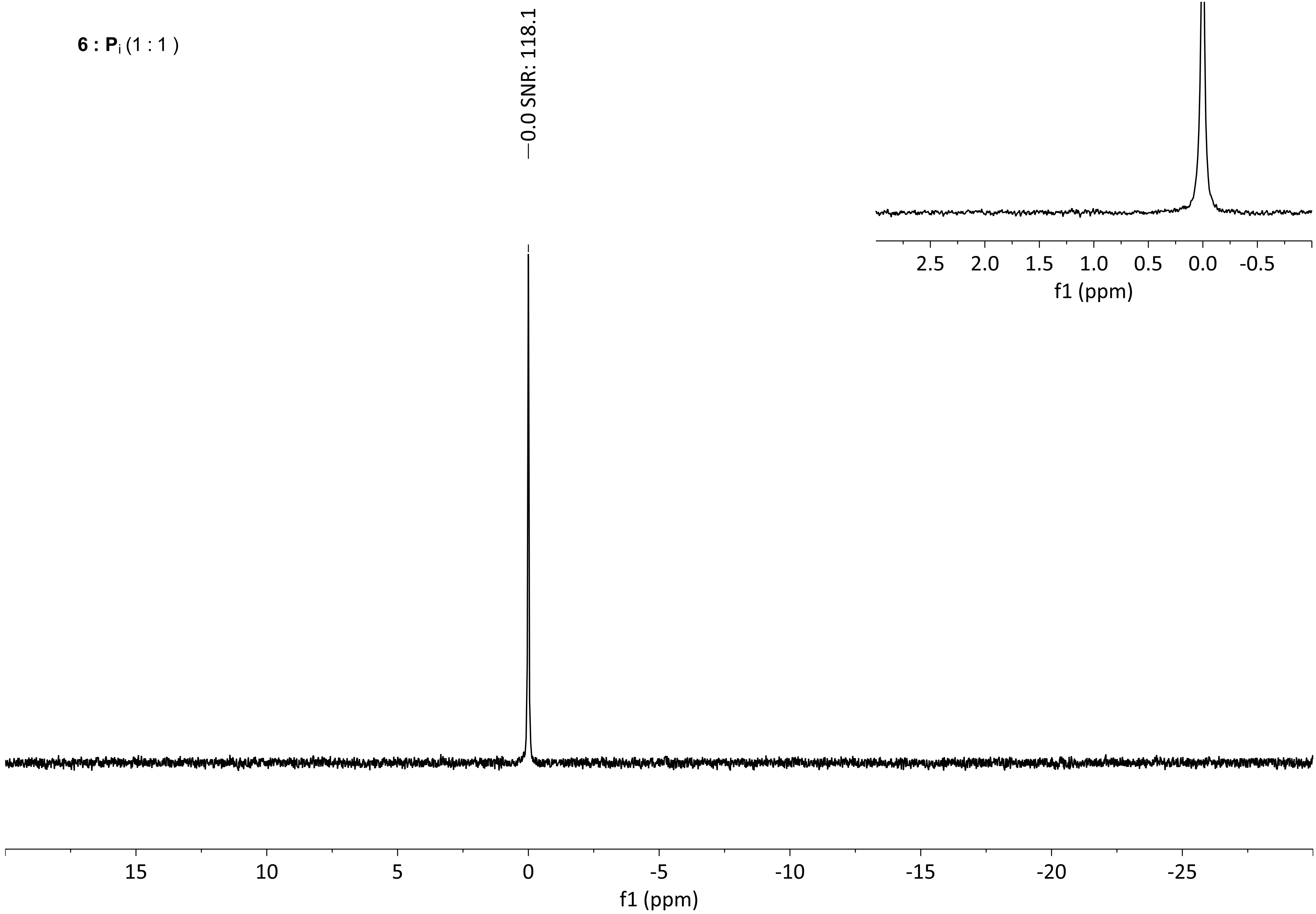

6 : 2a : P$_i$ (1 : 1 : 1)

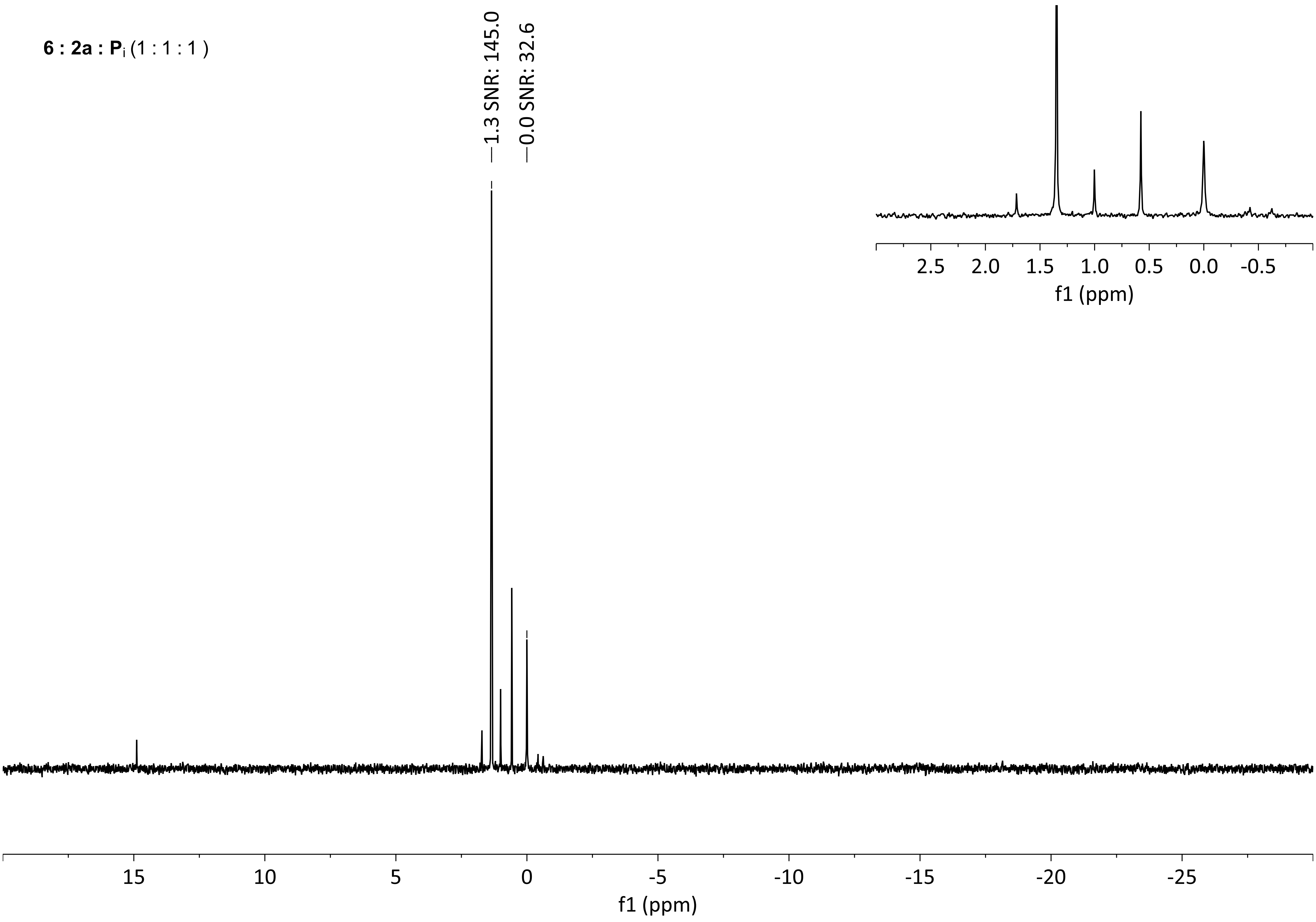
—1.3 SNR: 145.0
—0.0 SNR: 32.6
2.5 2.0 1.5 1.0 0.5 0.0 -0.5
f1 (ppm)
15 10 5 0 -5 -10 -15 -20 -25
f1 (ppm)

6 : 1 : P$_i$ (1 : 1 : 1)

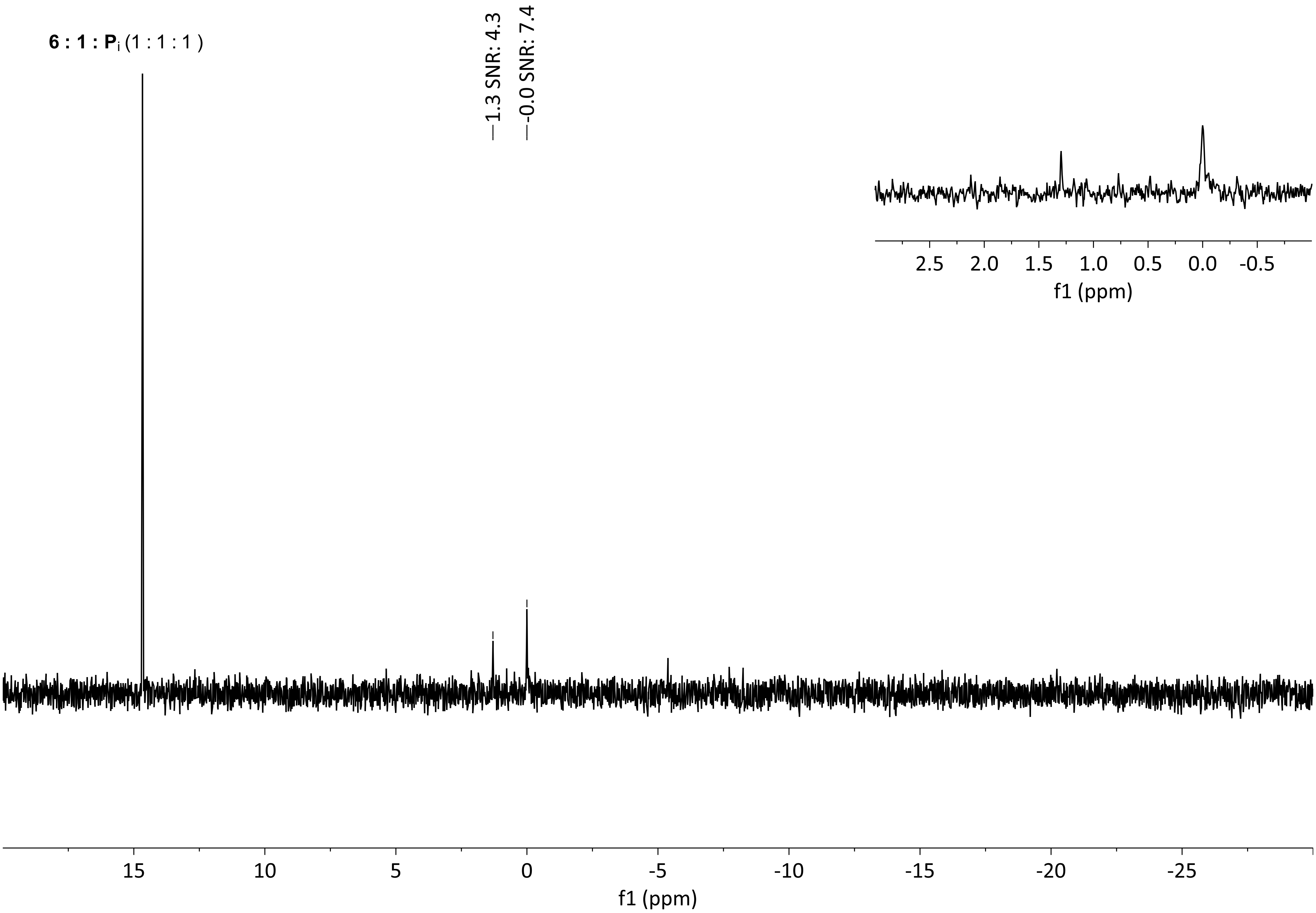

1.3 SNR: 4.3
-0.0 SNR: 7.4
2.5
2.0
1.5
1.0
0.5
0.0
-0.5
f1 (ppm)
15
10
5
0
-5
-10
-15
-20
-25
f1 (ppm)

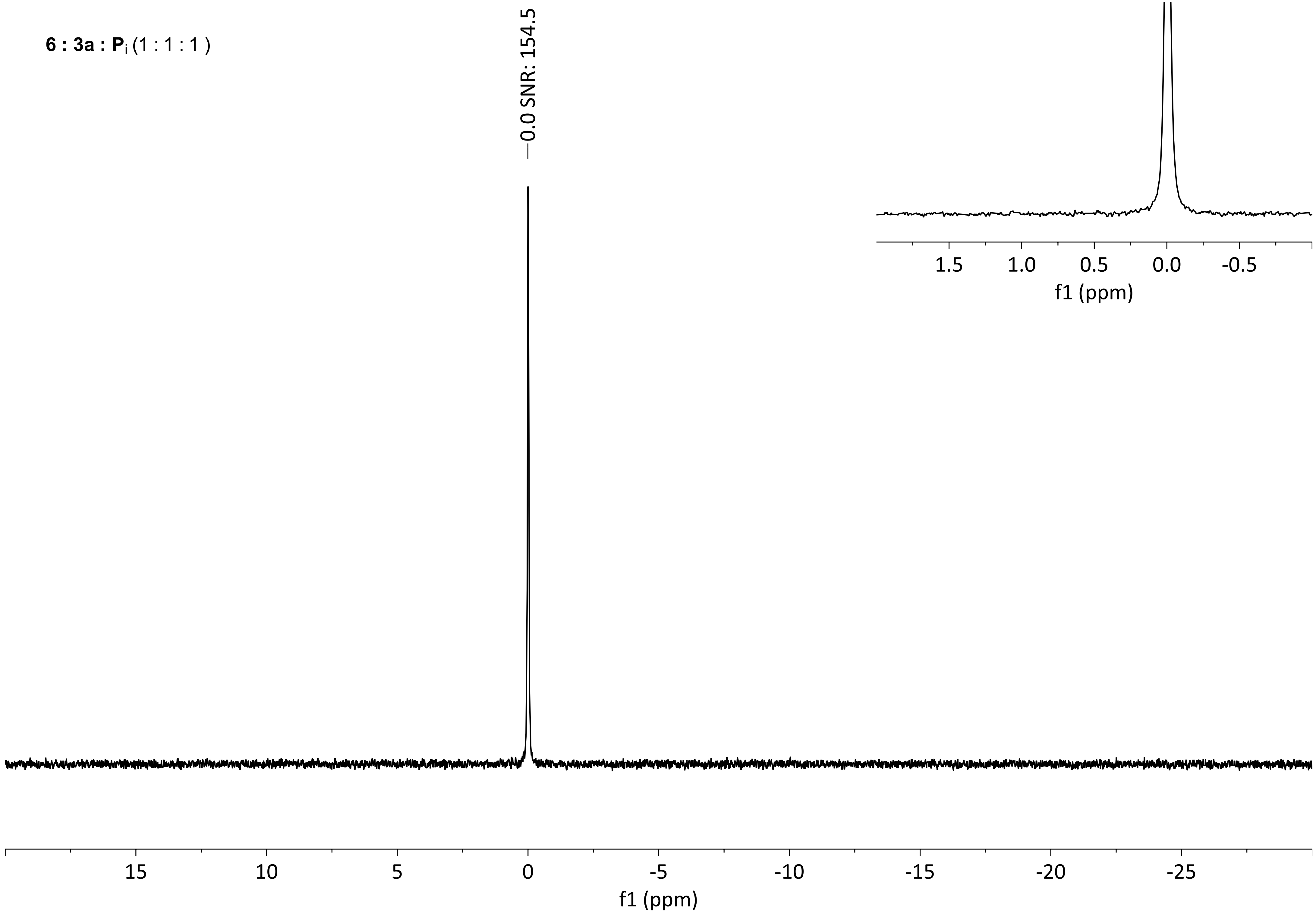
6 : 3a : Pi (1 : 1 : 1)
—0.0 SNR: 154.5
1.5
1.0
0.5
0.0
-0.5
f1 (ppm)
15
10
5
0
-5
-10
-15
-20
-25
f1 (ppm)

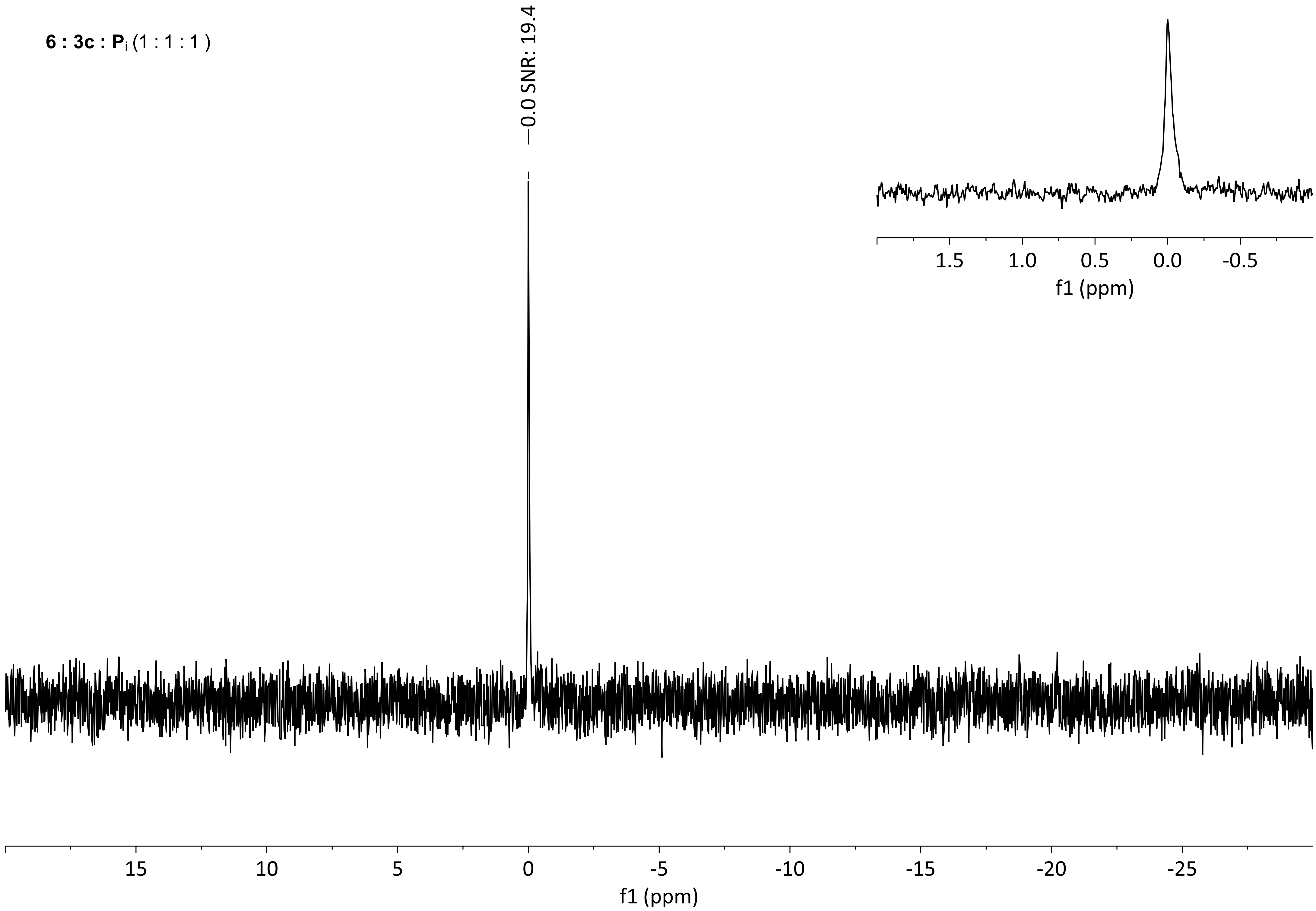

6 : 3c : P$_i$ (1 : 1 : 1)
0.0 SNR: 19.4
1.5
1.0
0.5
0.0
-0.5
f1 (ppm)
15
10
5
0
-5
-10
-15
-20
-25
f1 (ppm)

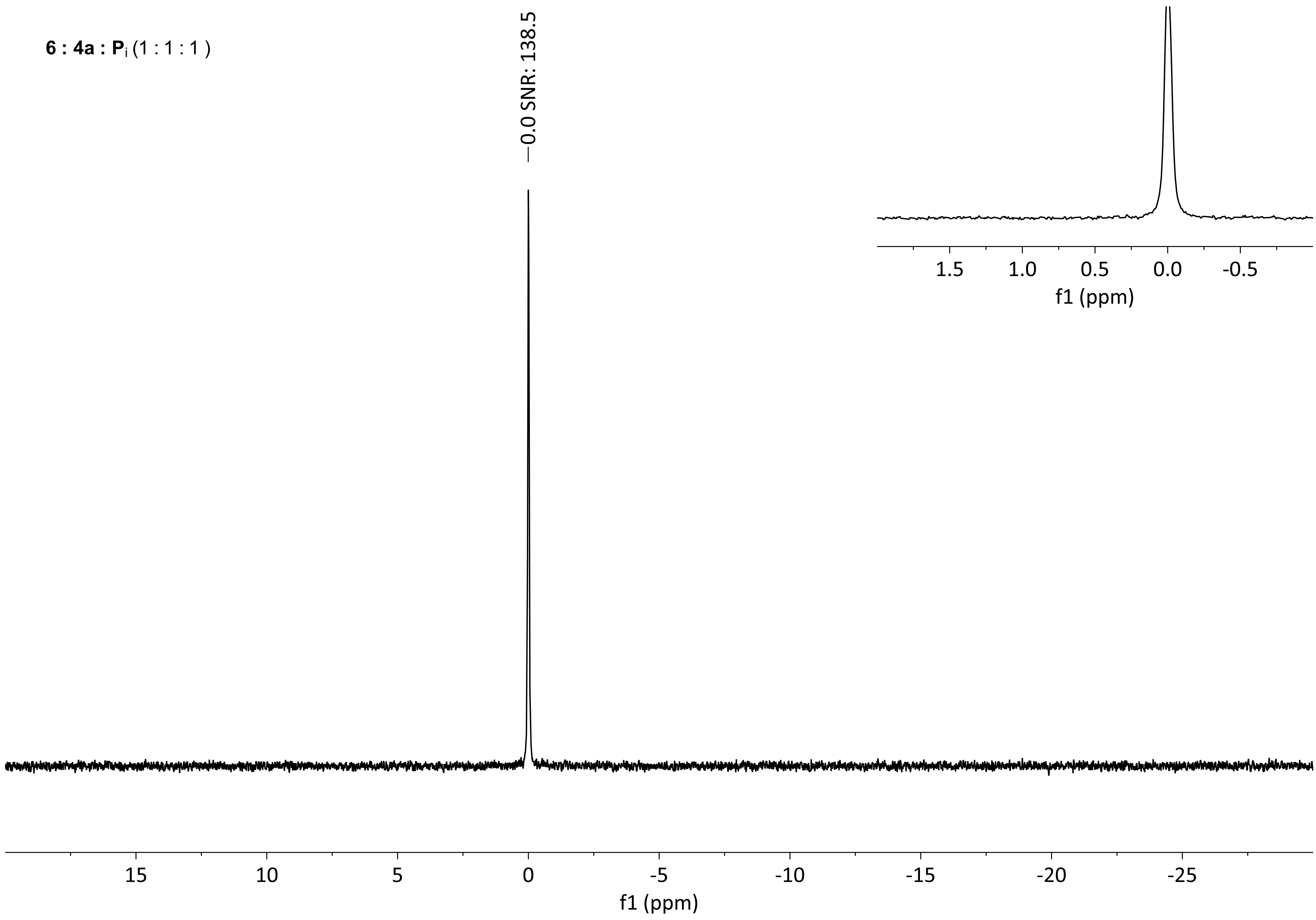
6 : 4a : Pi (1 : 1 : 1)
—0.0 SNR: 138.5
1.5
1.0
0.5
0.0
-0.5
f1 (ppm)
15
10
5
0
-5
-10
-15
-20
-25
f1 (ppm)

6 : 4c : P$_i$ (1 : 1 : 1)

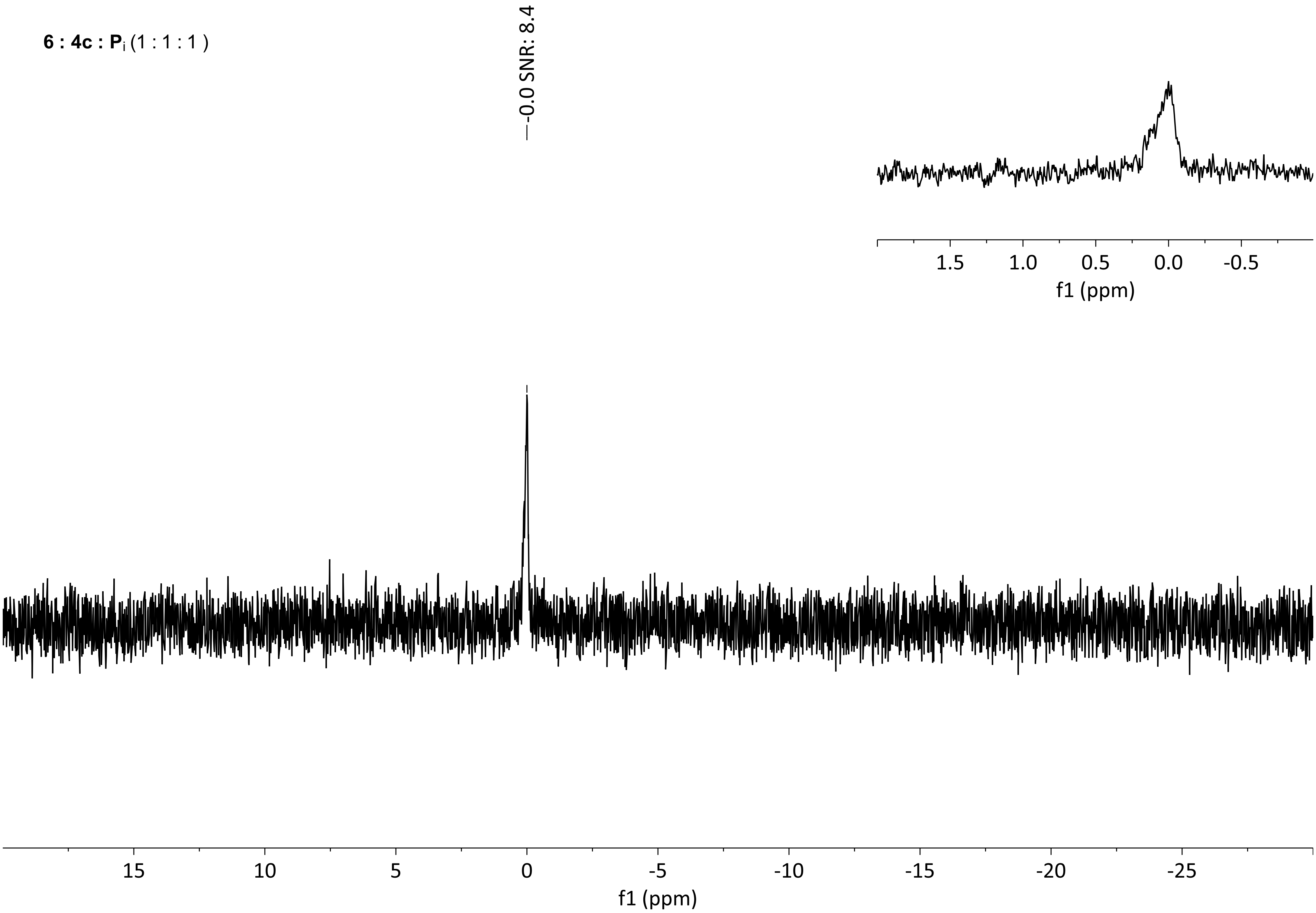

Supplement: Supplementary file 5 — Supplementary Data 3 [file 41467_2025_63307_MOESM5_ESM.pdf]
