# Supplementary figures and images for: A scope of prebiotic neat reaction conditions and the mechanism of urea-assisted phosphorylations of alcohols
Source: Nat Commun. 2025 Oct 8;16:8929. doi: 10.1038/s41467-025-63307-3 (PMC12508118; doi:10.1038/s41467-025-63307-3)

6 : cTMP (1 : 1 )

—1.8 SNR: 12.7  
—0.0 SNR: 1.9

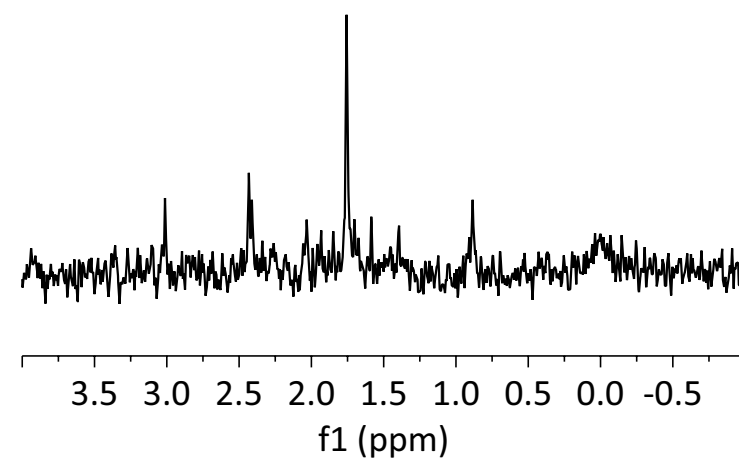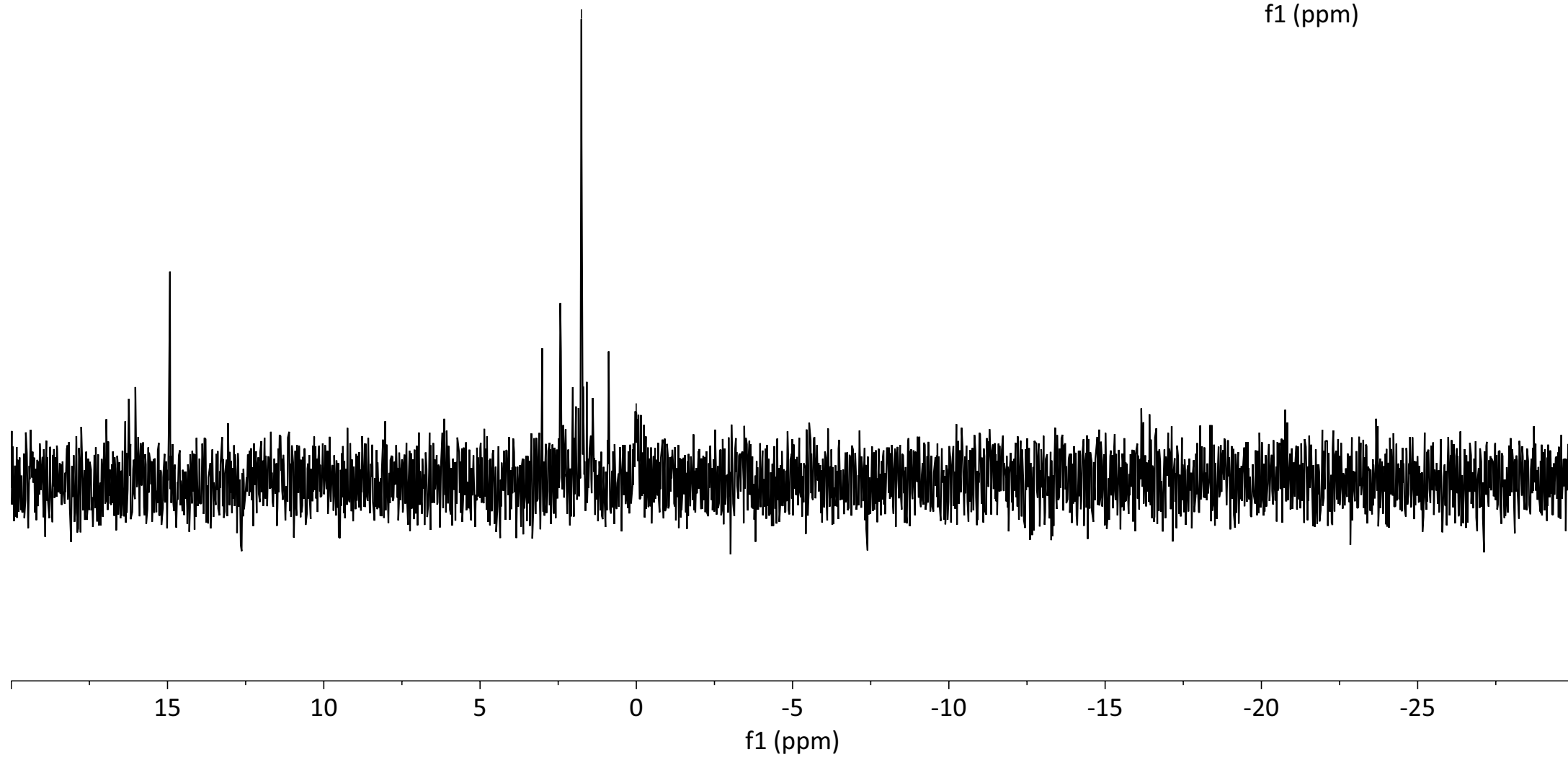

6 : 2a : cTMP (1 : 1 : 1 )

—1.3 SNR: 5.5  
—0.0 SNR: 2.5

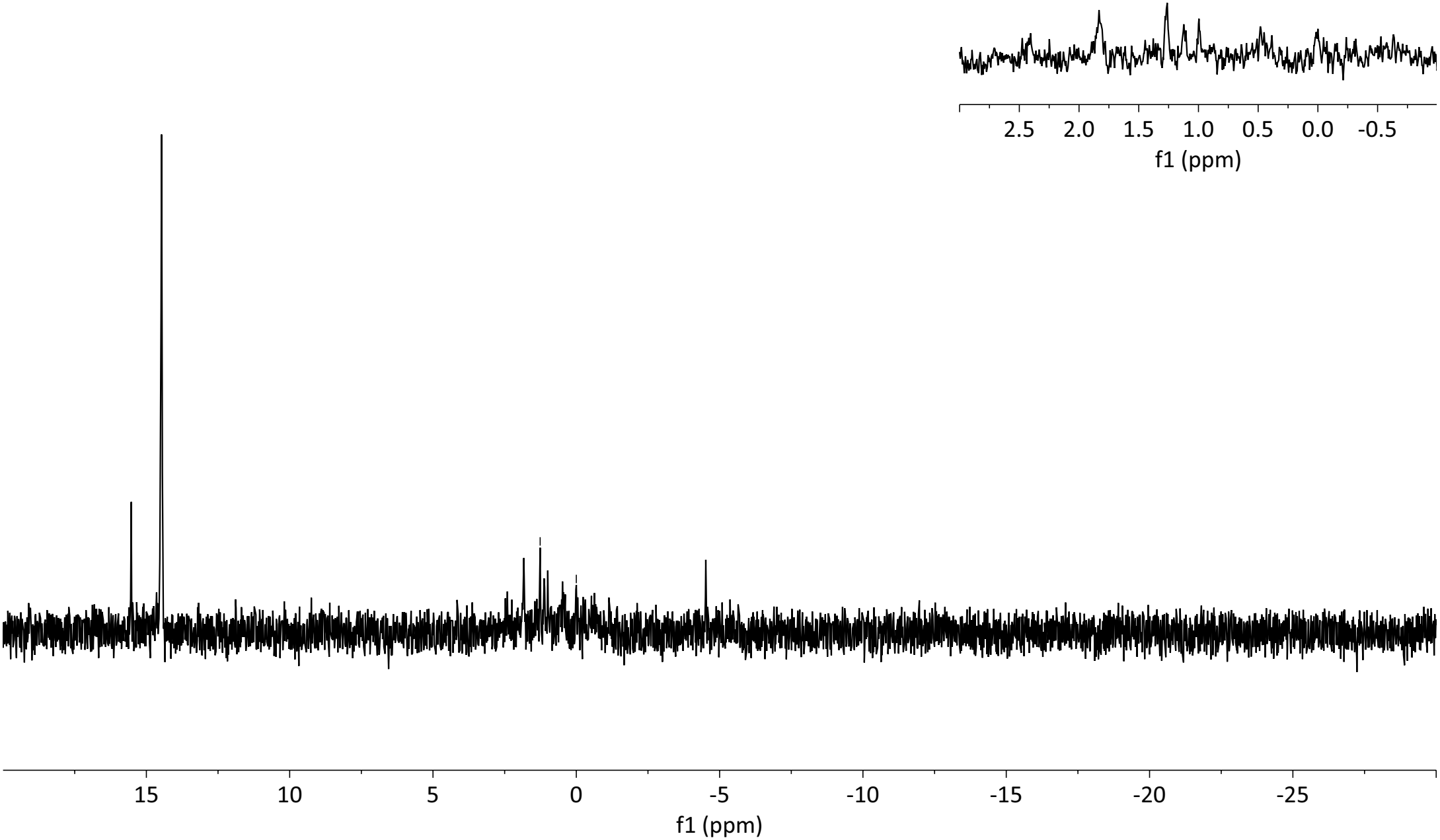

6 : 1 : cTMP (1 : 1 : 1 )

—1.7 SNR: 4.9  
—0.0 SNR: 5.8

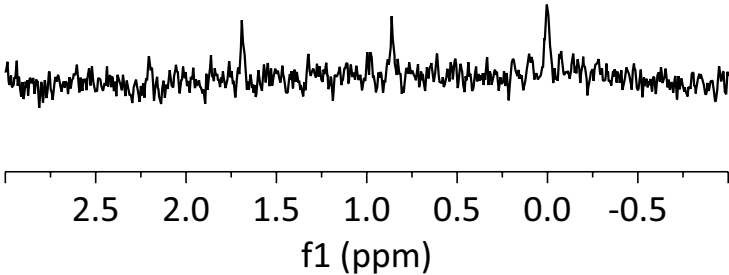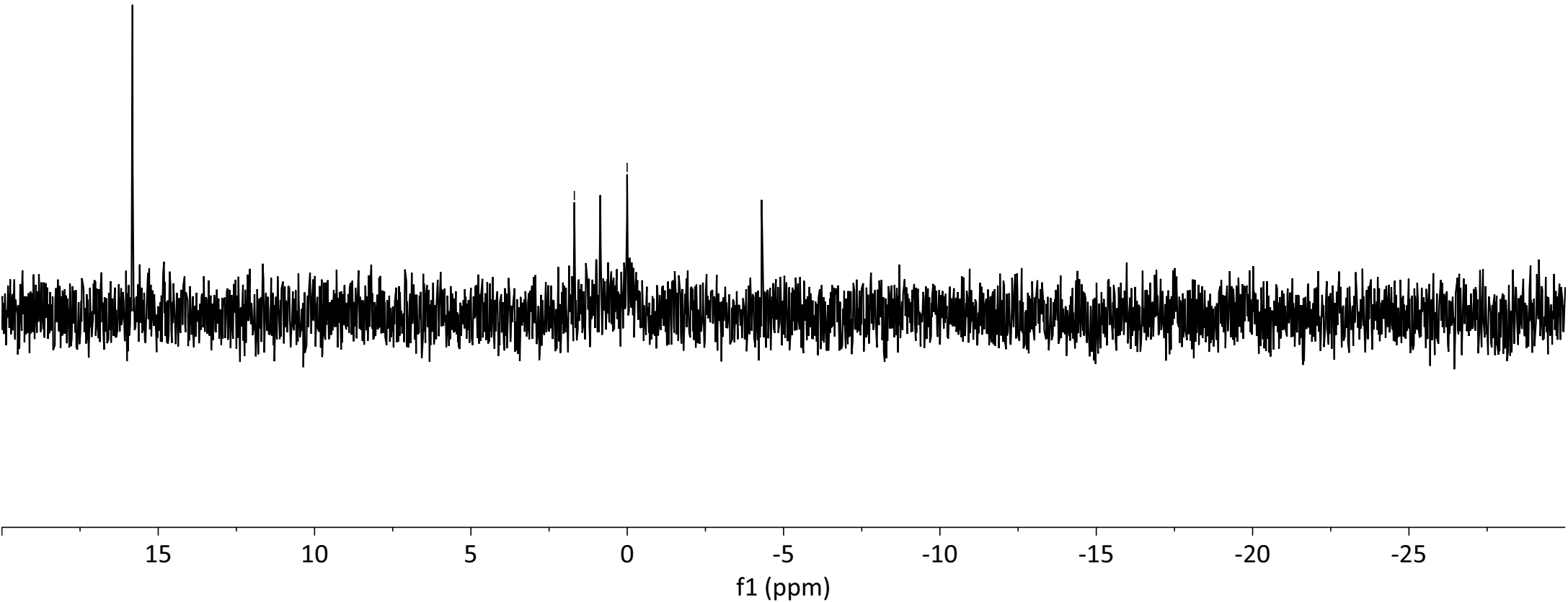

6 : 3a : cTMP (1 : 1 : 1)

—2.0 SNR: 4.8

—0.0 SNR: 3.3

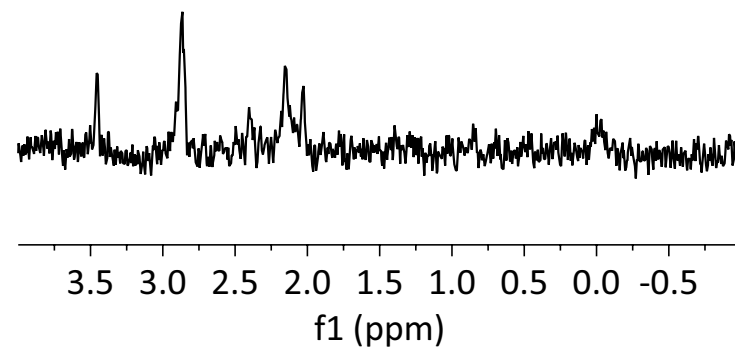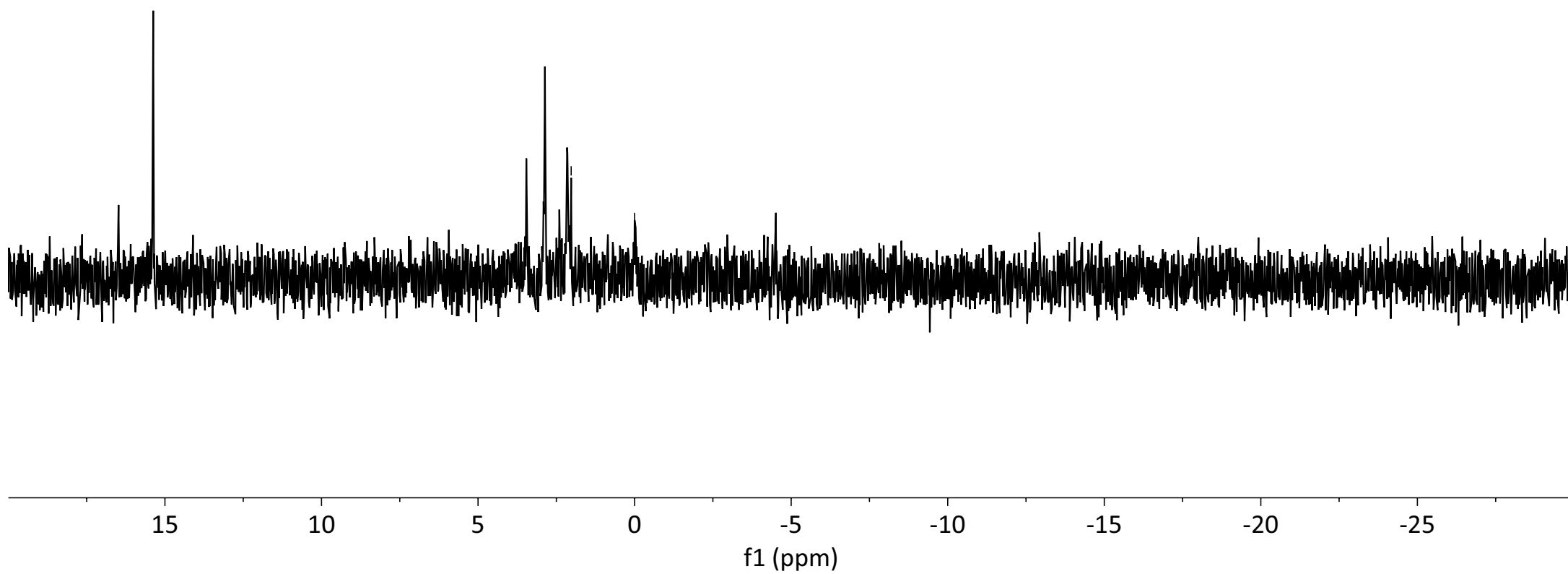

**6 : 3c : cTMP (1 : 1 : 1)**

~ 0.9 SNR: 5.6  
~ 0.0 SNR: 1.2

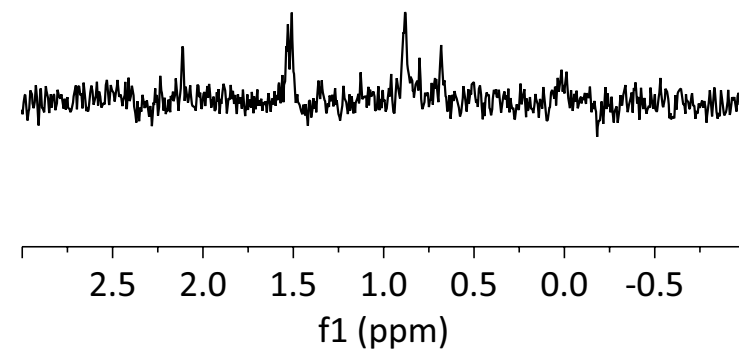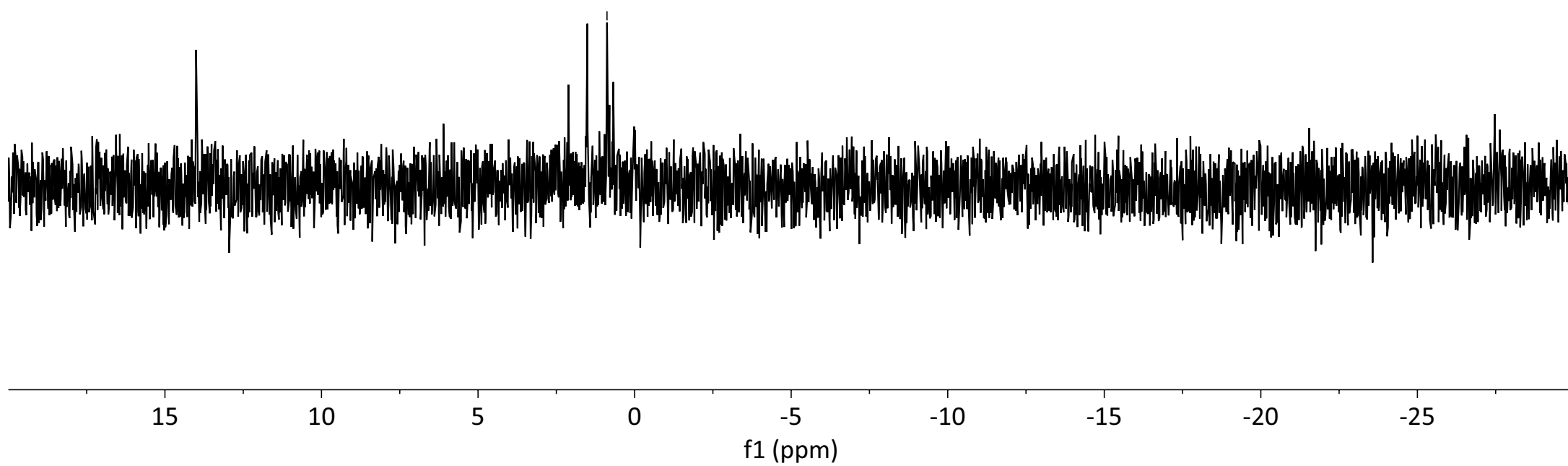

6 : 4a : cTMP (1 : 1 : 1 )

—2.1 SNR: 1.9  
—0.0 SNR: 4.4

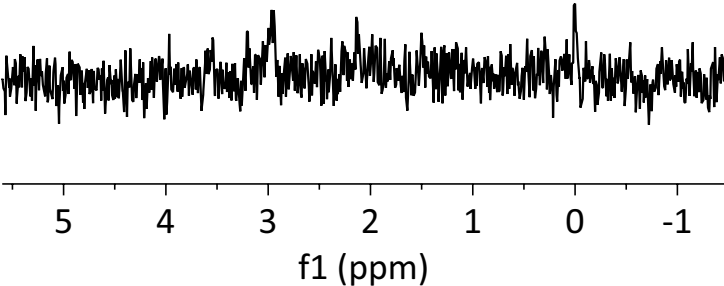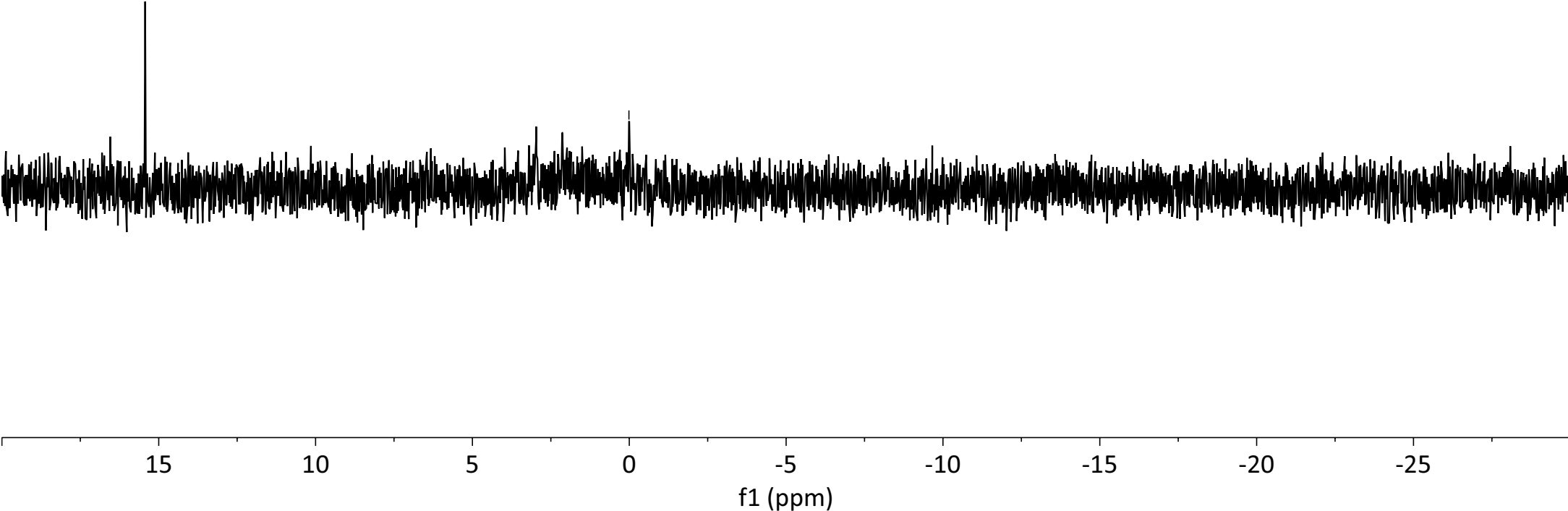

**6 : 4c : cTMP (1 : 1 : 1)**

~2.0 SNR: 13.6  
~1.1 SNR: 5.0

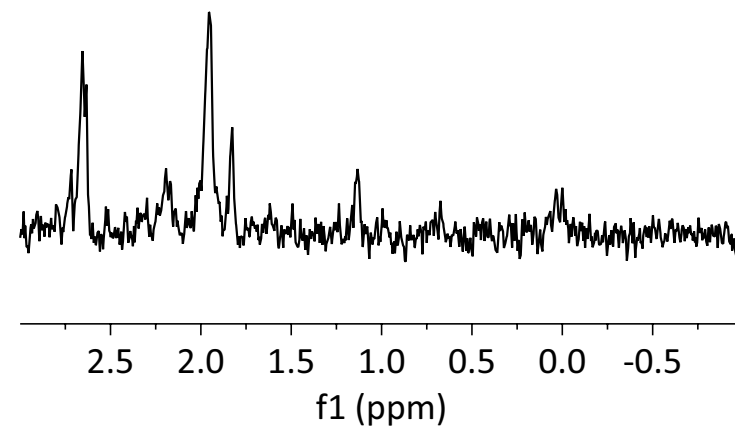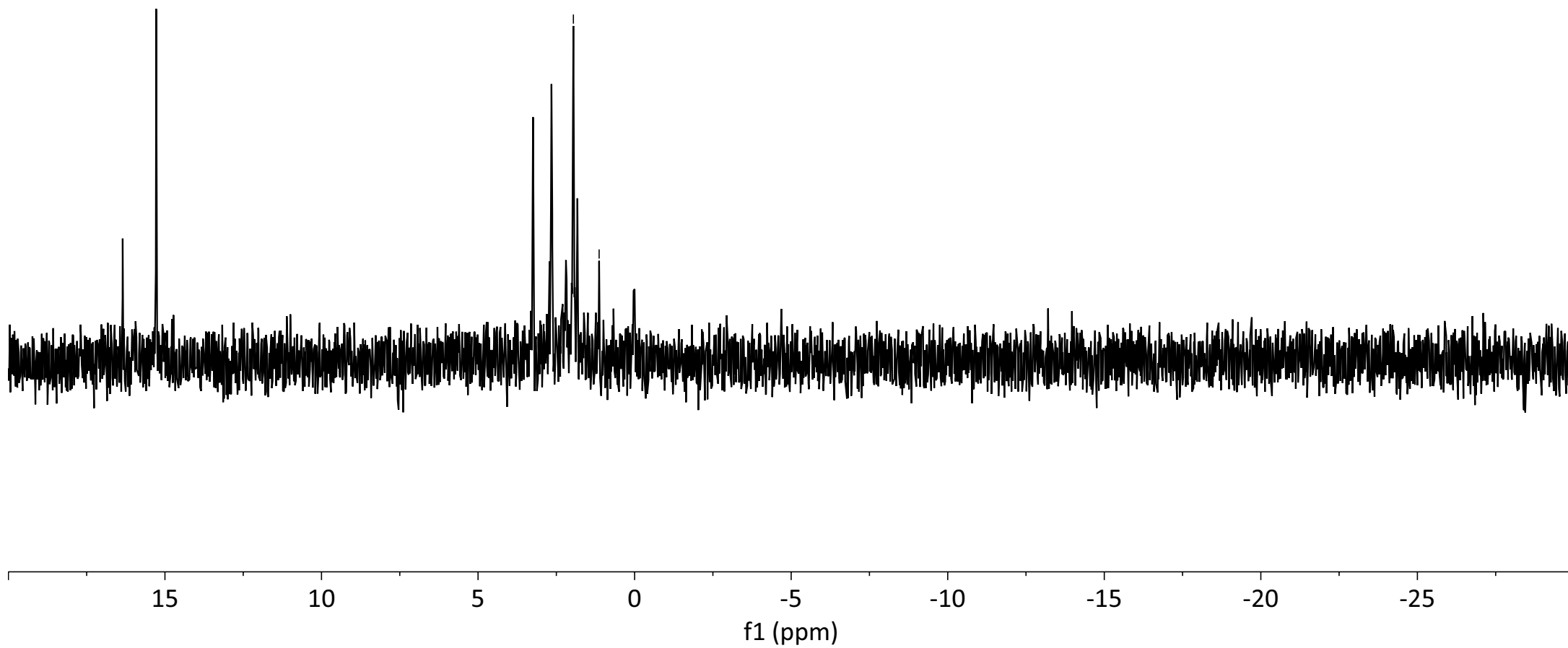

Supplement: Supplementary file 6 — Supplementary Data 4 [file 41467_2025_63307_MOESM6_ESM.pdf]
