# Supplementary material for: A scope of prebiotic neat reaction conditions and the mechanism of urea-assisted phosphorylations of alcohols
Source: Nat Commun. 2025 Oct 8;16:8929. doi: 10.1038/s41467-025-63307-3 (PMC12508118; doi:10.1038/s41467-025-63307-3)
Supplement: Supplementary file 7 — Supplementary Data 5 [file 41467_2025_63307_MOESM7_ESM.pdf]

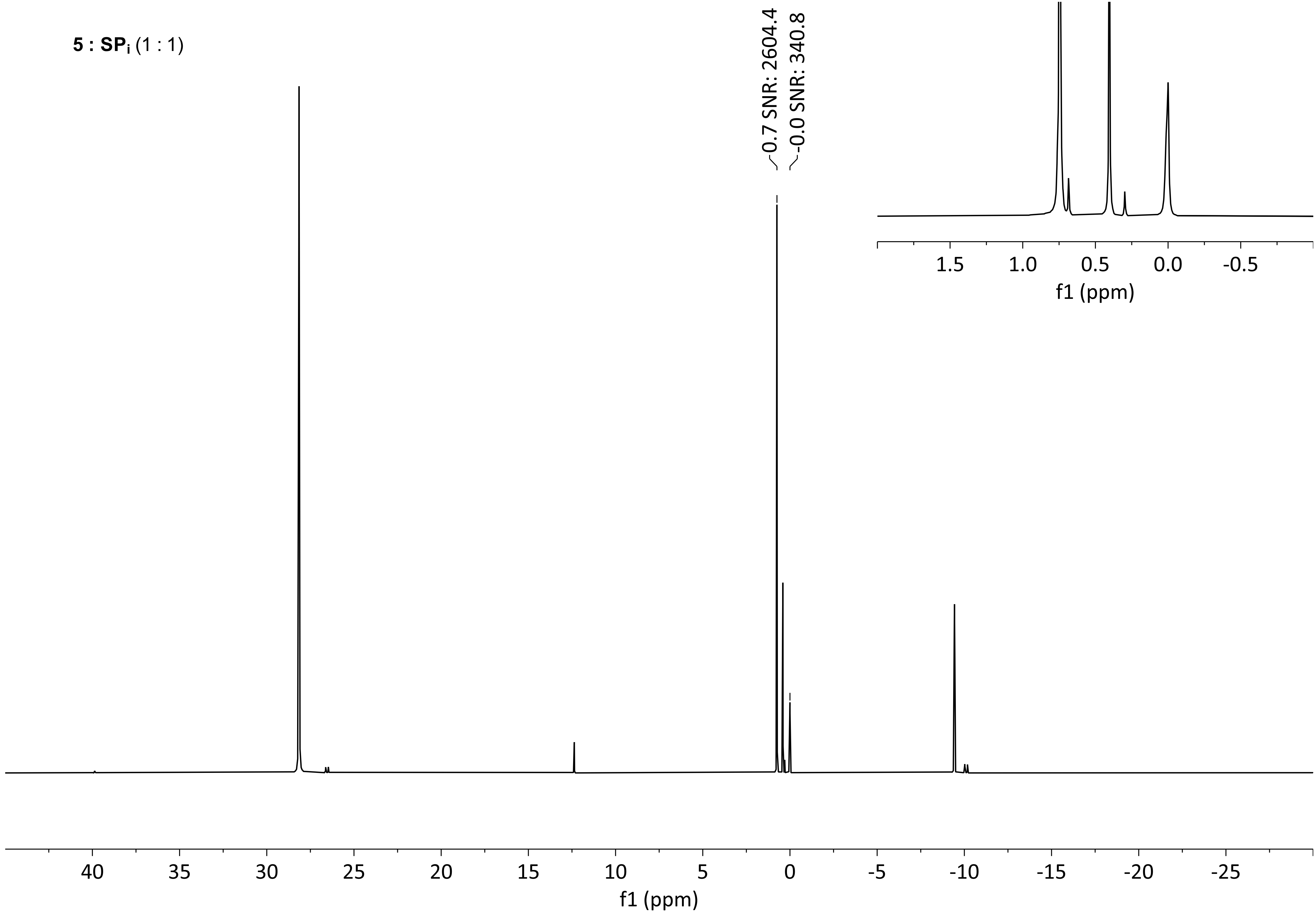
5 : SP_i (1 : 1)
0.7 SNR: 2604.4
-0.0 SNR: 340.8
1.5
1.0
0.5
0.0
-0.5
f1 (ppm)
40
35
30
25
20
15
10
5
0
-5
-10
-15
-20
-25
f1 (ppm)

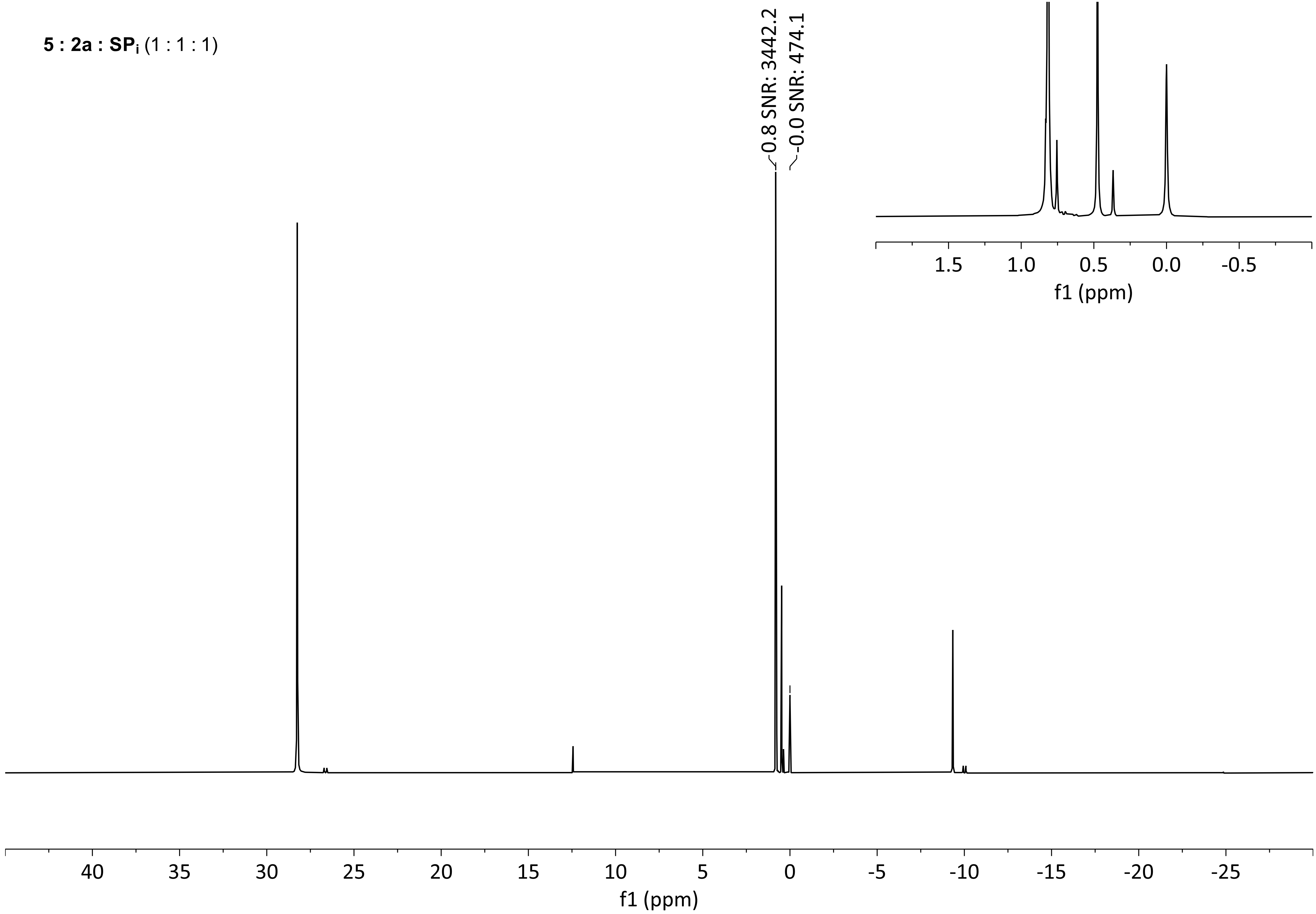
5 : 2a : SPi (1 : 1 : 1)
0.8 SNR: 3442.2
-0.0 SNR: 474.1
1.5
1.0
0.5
0.0
-0.5
f1 (ppm)
40
35
30
25
20
15
10
5
0
-5
-10
-15
-20
-25
f1 (ppm)

5 : 1 : SP$_i$ (1 : 1 : 1)

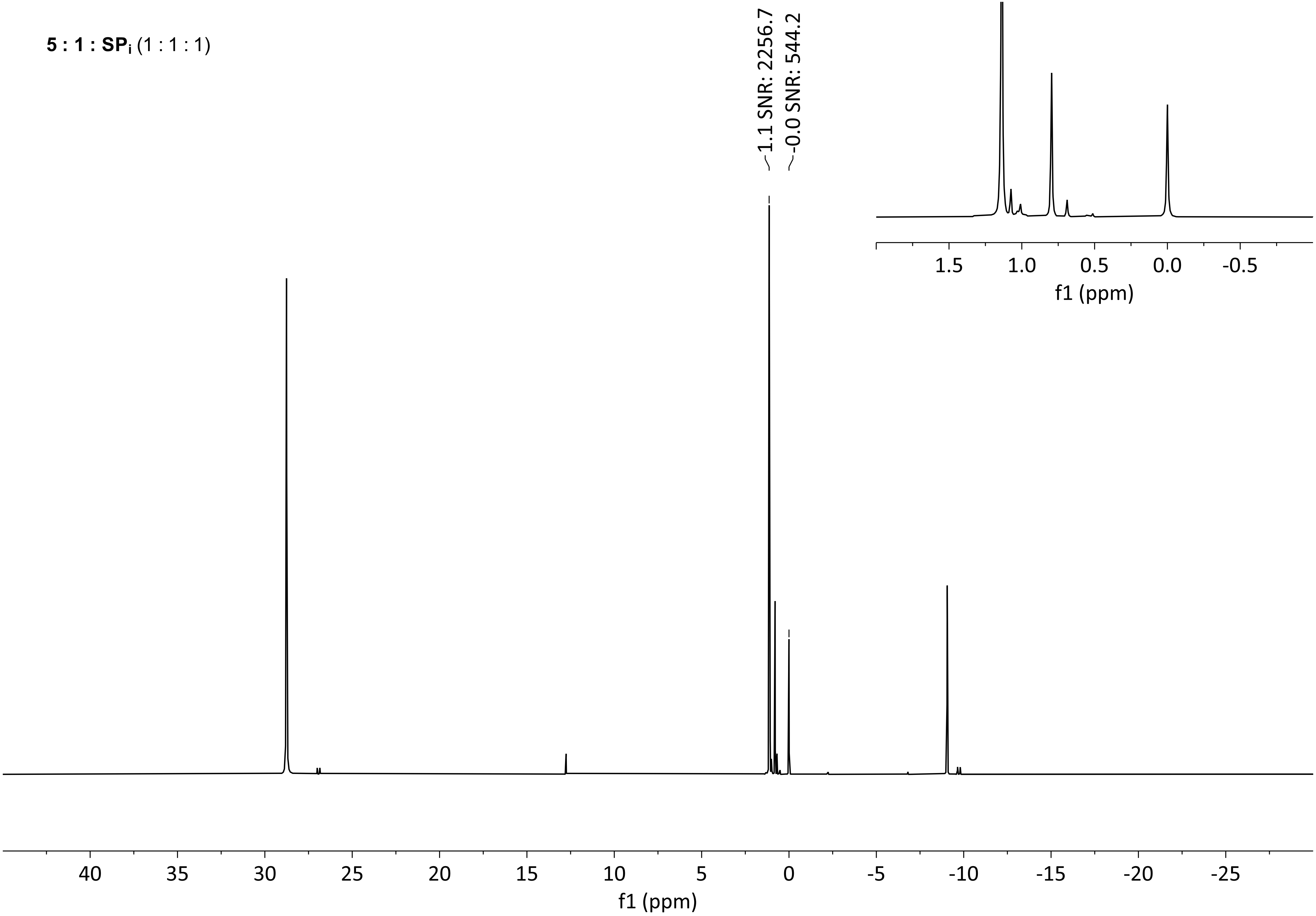
1.1 SNR: 2256.7
-0.0 SNR: 544.2
40
35
30
25
20
15
10
5
0
-5
-10
-15
-20
-25
f1 (ppm)
1.5
1.0
0.5
0.0
-0.5
f1 (ppm)

5 : 2a : NaH$_2$PO$_4$: SP$_i$ (1 : 1 : 0.5 : 0.5)

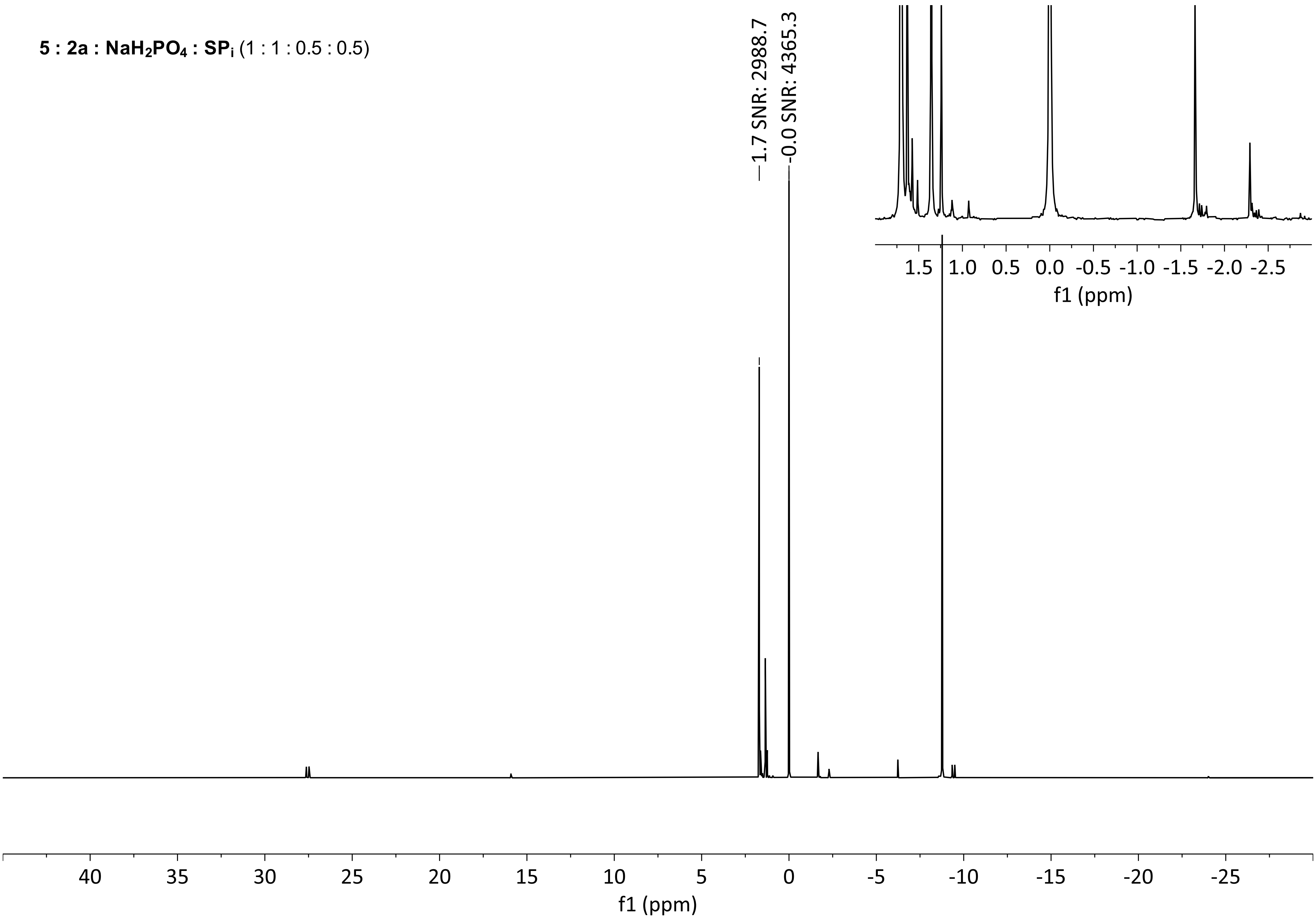

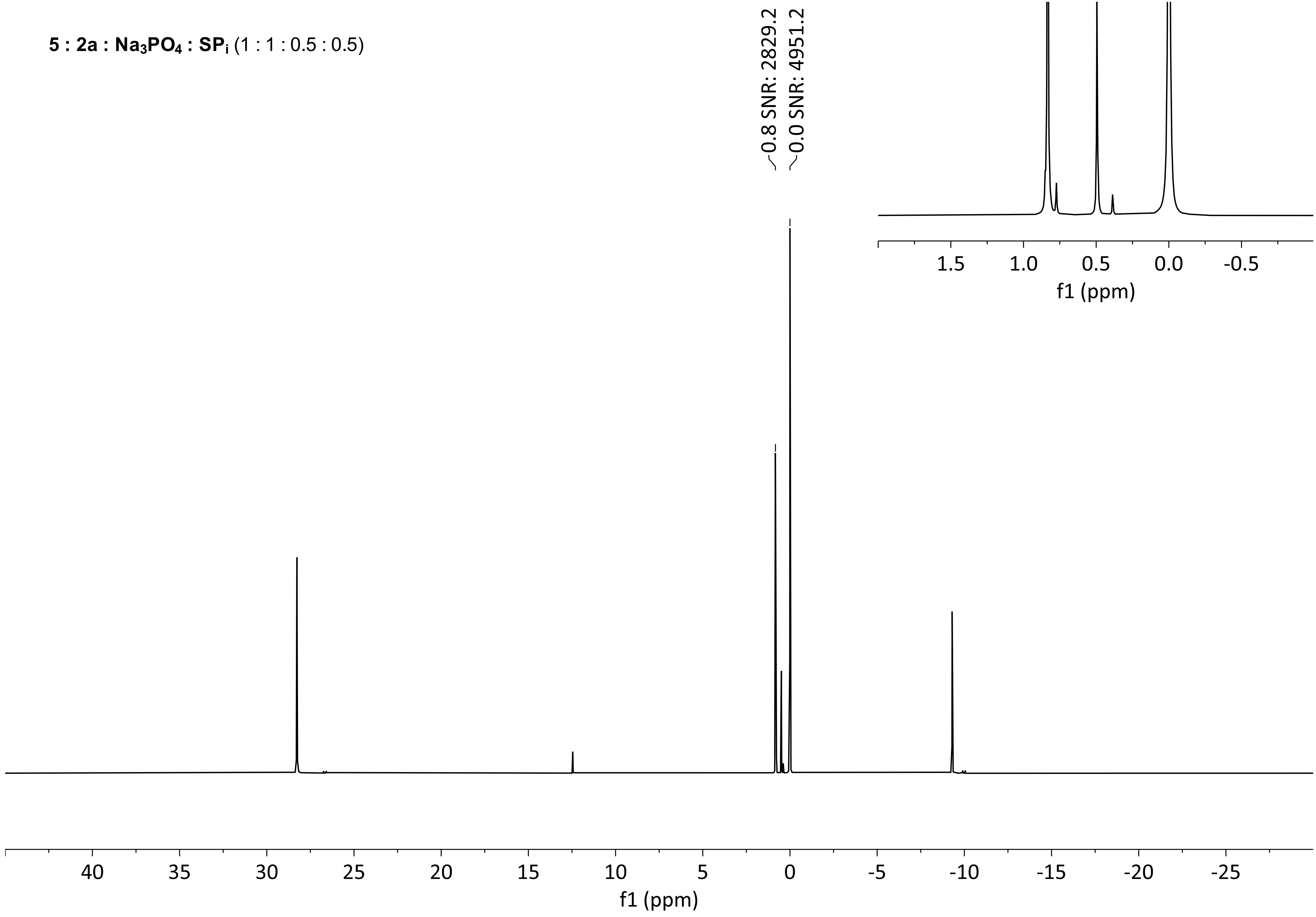

5 : 2a : Na3PO4 : SPi (1 : 1 : 0.5 : 0.5)
0.8 SNR: 2829.2
0.0 SNR: 4951.2
1.5
1.0
0.5
0.0
-0.5
f1 (ppm)
40
35
30
25
20
15
10
5
0
-5
-10
-15
-20
-25
f1 (ppm)
