# Supplementary material for: A scope of prebiotic neat reaction conditions and the mechanism of urea-assisted phosphorylations of alcohols
Source: Nat Commun. 2025 Oct 8;16:8929. doi: 10.1038/s41467-025-63307-3 (PMC12508118; doi:10.1038/s41467-025-63307-3)
Supplement: Supplementary file 8 — Supplementary Data 6 [file 41467_2025_63307_MOESM8_ESM.pdf]

5 : SP_i (1 : 1)

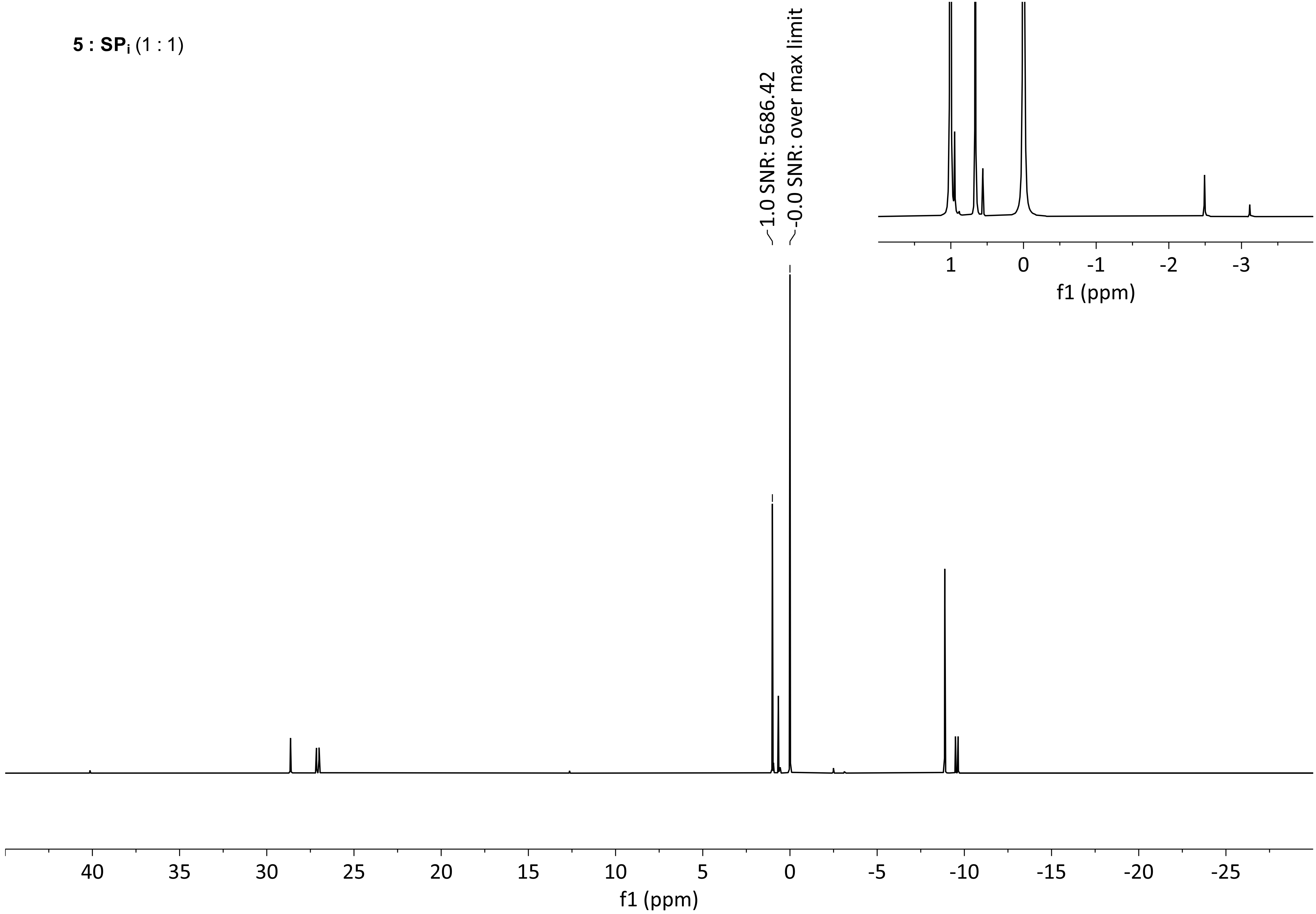

1.0 SNR: 5686.42
-0.0 SNR: over max limit
1
0
-1
-2
-3
f1 (ppm)
40
35
30
25
20
15
10
5
0
-5
-10
-15
-20
-25
f1 (ppm)

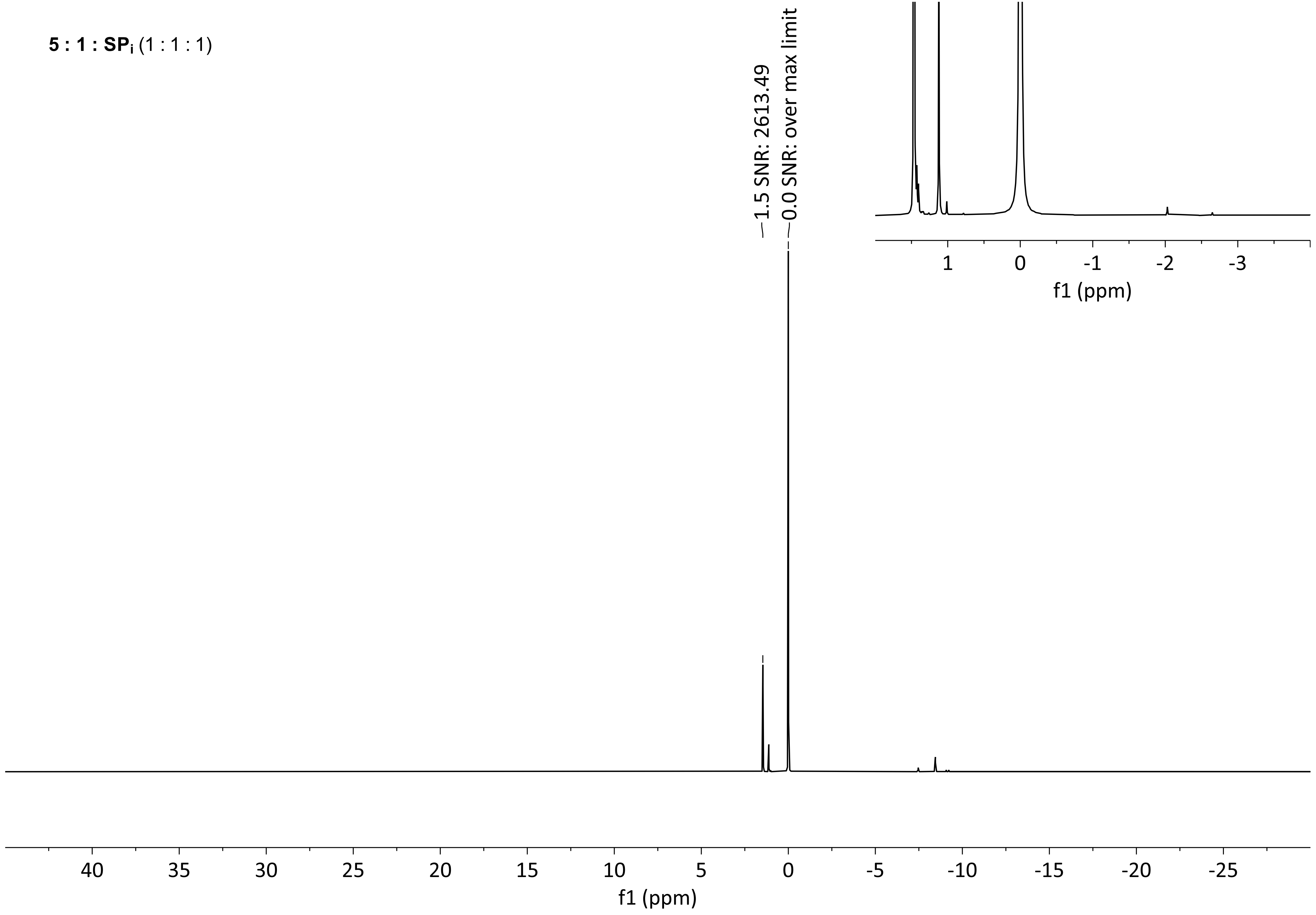
5 : 1 : SP$_i$ (1 : 1 : 1)
1.5 SNR: 2613.49
0.0 SNR: over max limit
1
0
-1
-2
-3
f1 (ppm)
40
35
30
25
20
15
10
5
0
-5
-10
-15
-20
-25
f1 (ppm)

5 : 2a : Na_2HPO_4 : SP_i (1 : 1 : 0.5 : 0.5)

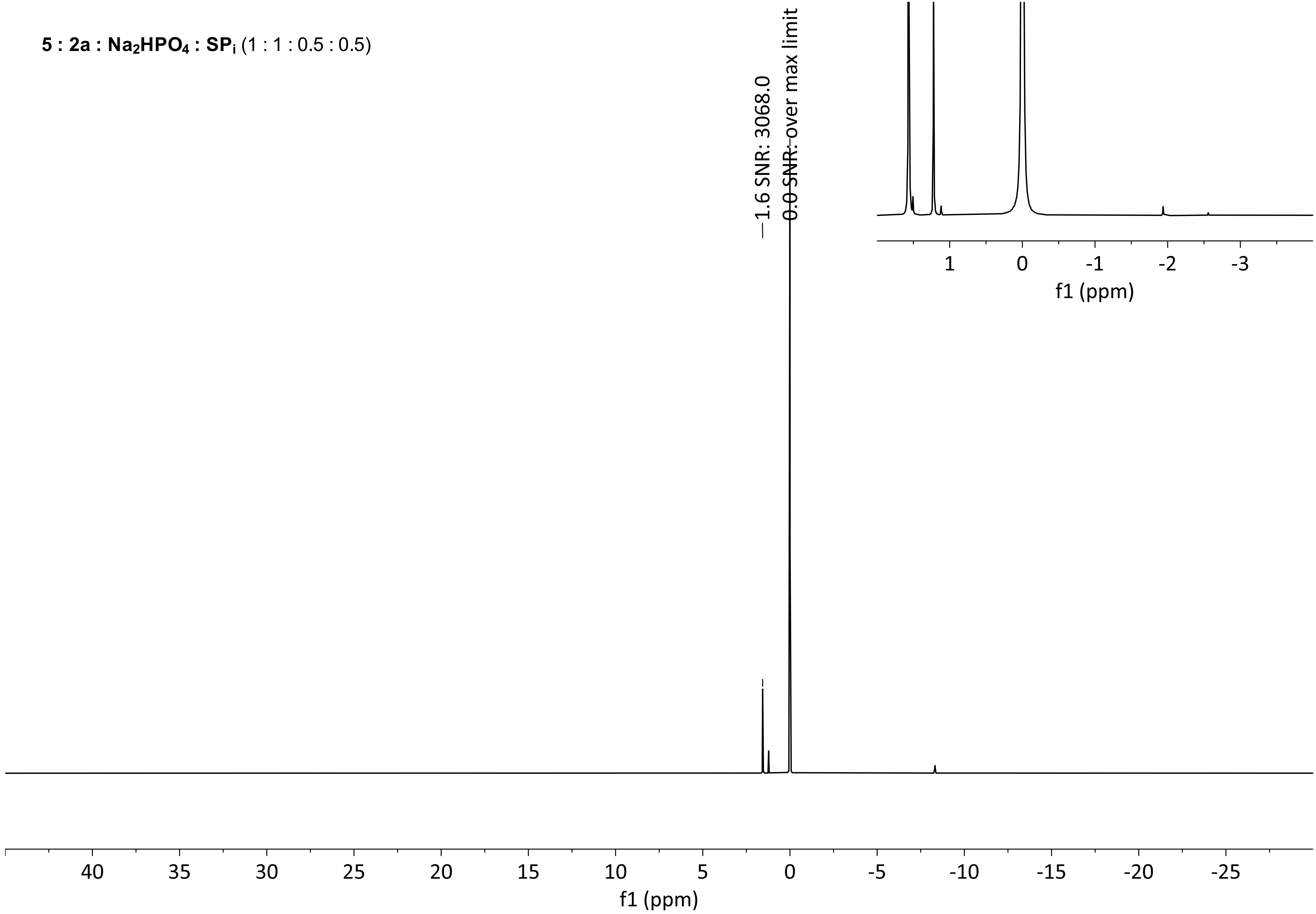

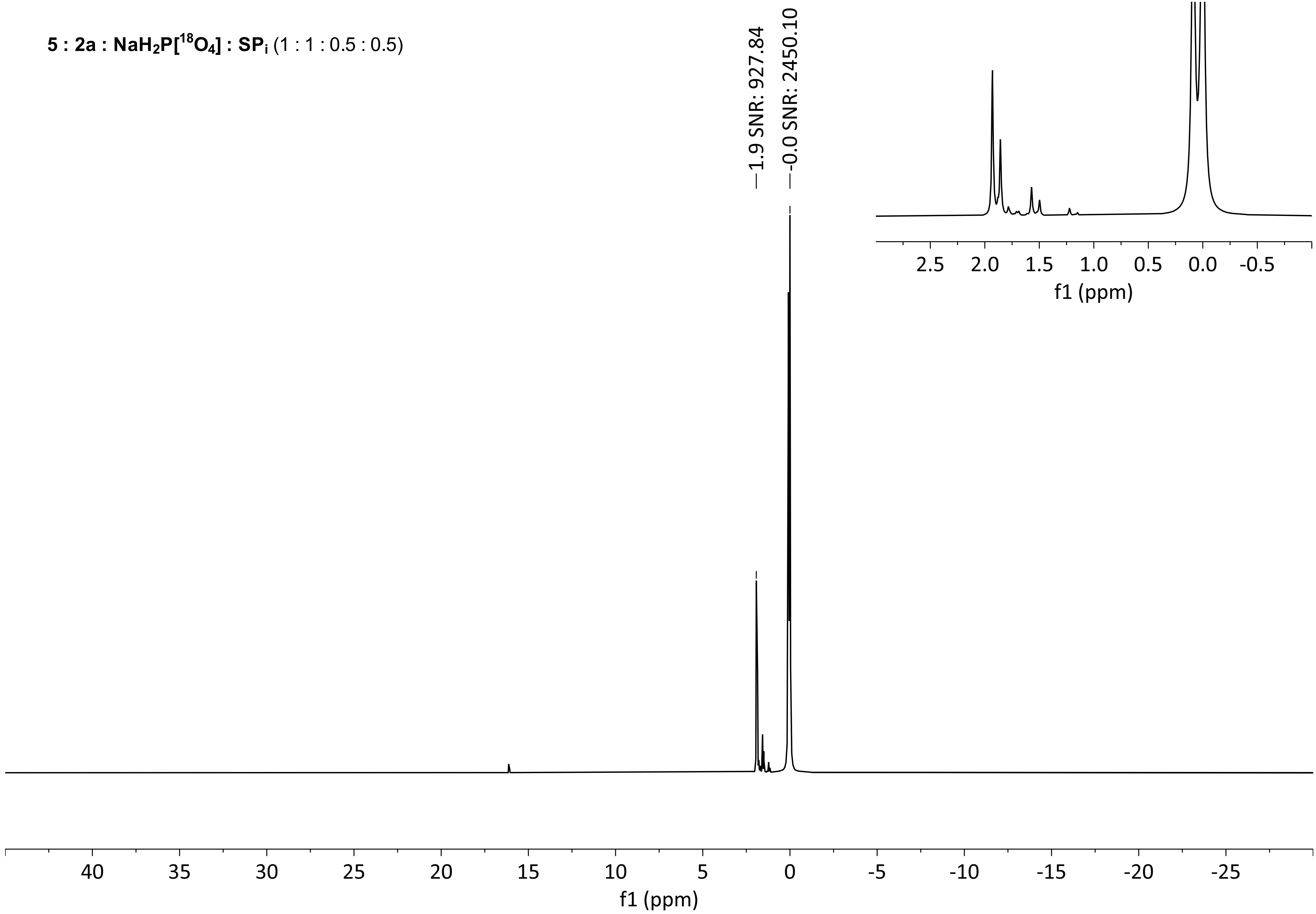
5 : 2a : NaH2P[18O4] : SPi (1 : 1 : 0.5 : 0.5)
1.9 SNR: 927.84
-0.0 SNR: 2450.10
f1 (ppm)
f1 (ppm)
