# Supplementary material for: A scope of prebiotic neat reaction conditions and the mechanism of urea-assisted phosphorylations of alcohols
Source: Nat Commun. 2025 Oct 8;16:8929. doi: 10.1038/s41467-025-63307-3 (PMC12508118; doi:10.1038/s41467-025-63307-3)
Supplement: Supplementary file 9 — Supplementary Data 7 [file 41467_2025_63307_MOESM9_ESM.pdf]

5 : Struvite (1 : 1)

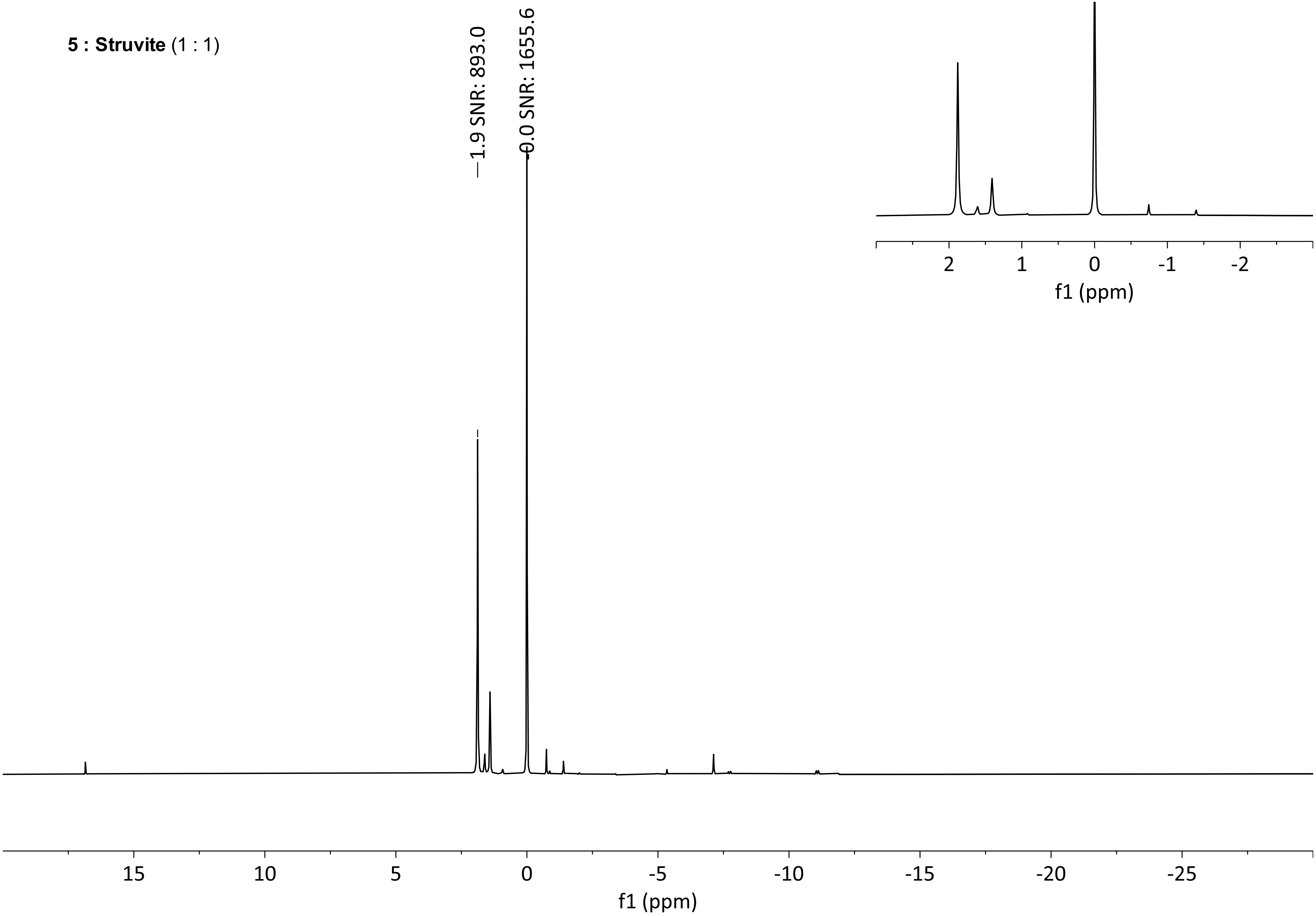

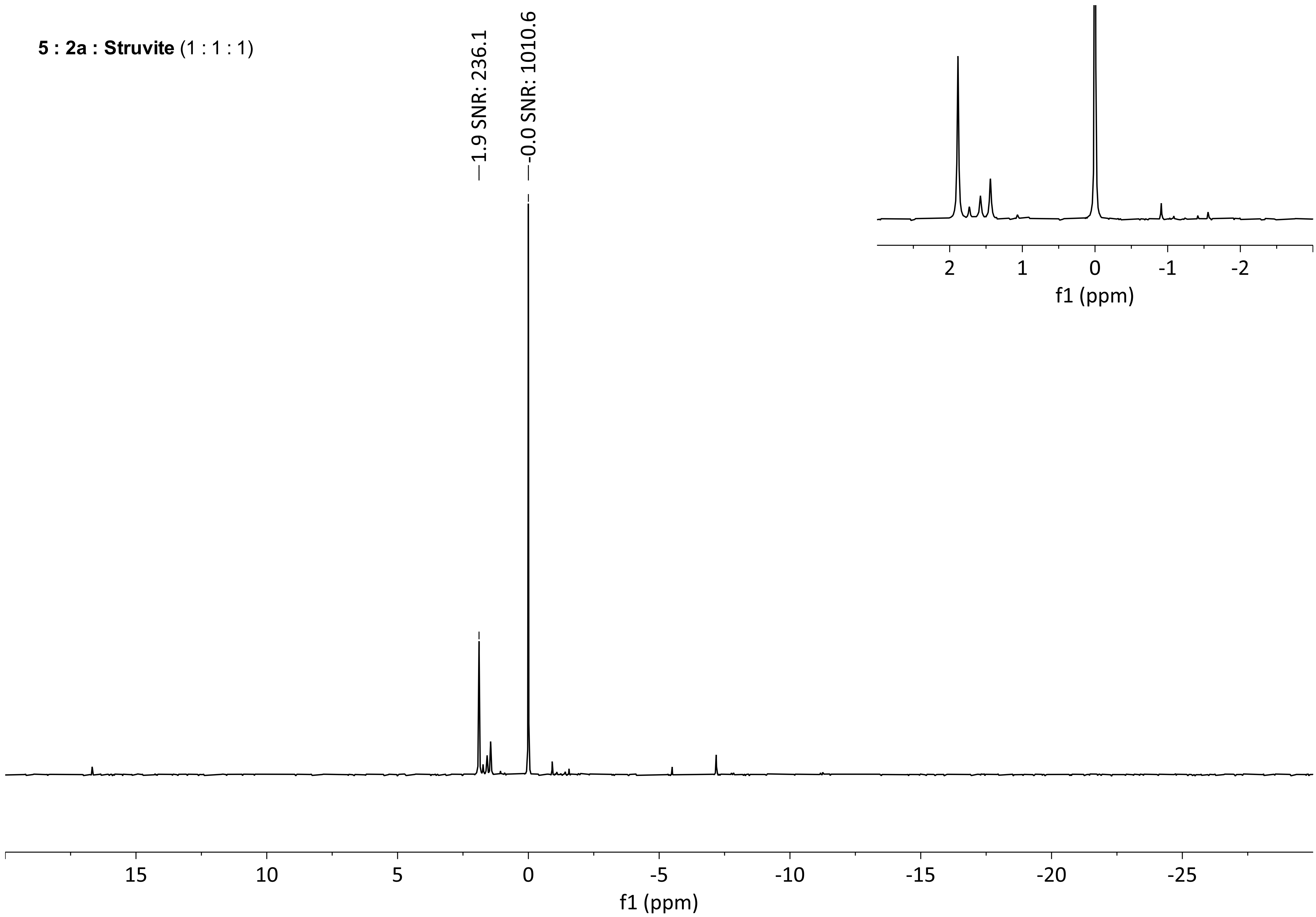

5 : 2a : Struvite (1 : 1 : 1)
1.9 SNR: 236.1
-0.0 SNR: 1010.6
f1 (ppm)

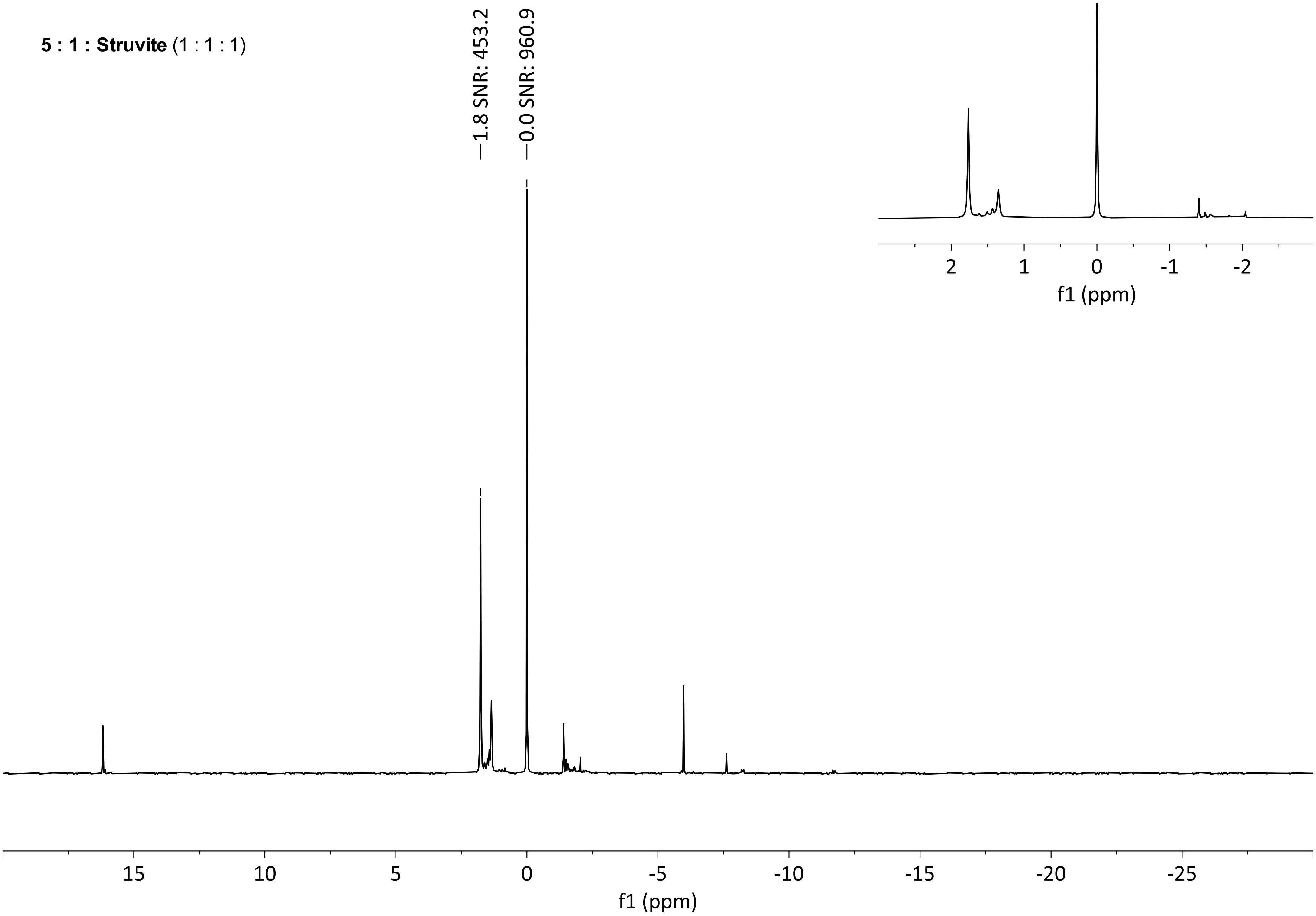

5 : 1 : Struvite (1 : 1 : 1)
1.8 SNR: 453.2
0.0 SNR: 960.9
2
1
0
-1
-2
f1 (ppm)
15
10
5
0
-5
-10
-15
-20
-25
f1 (ppm)

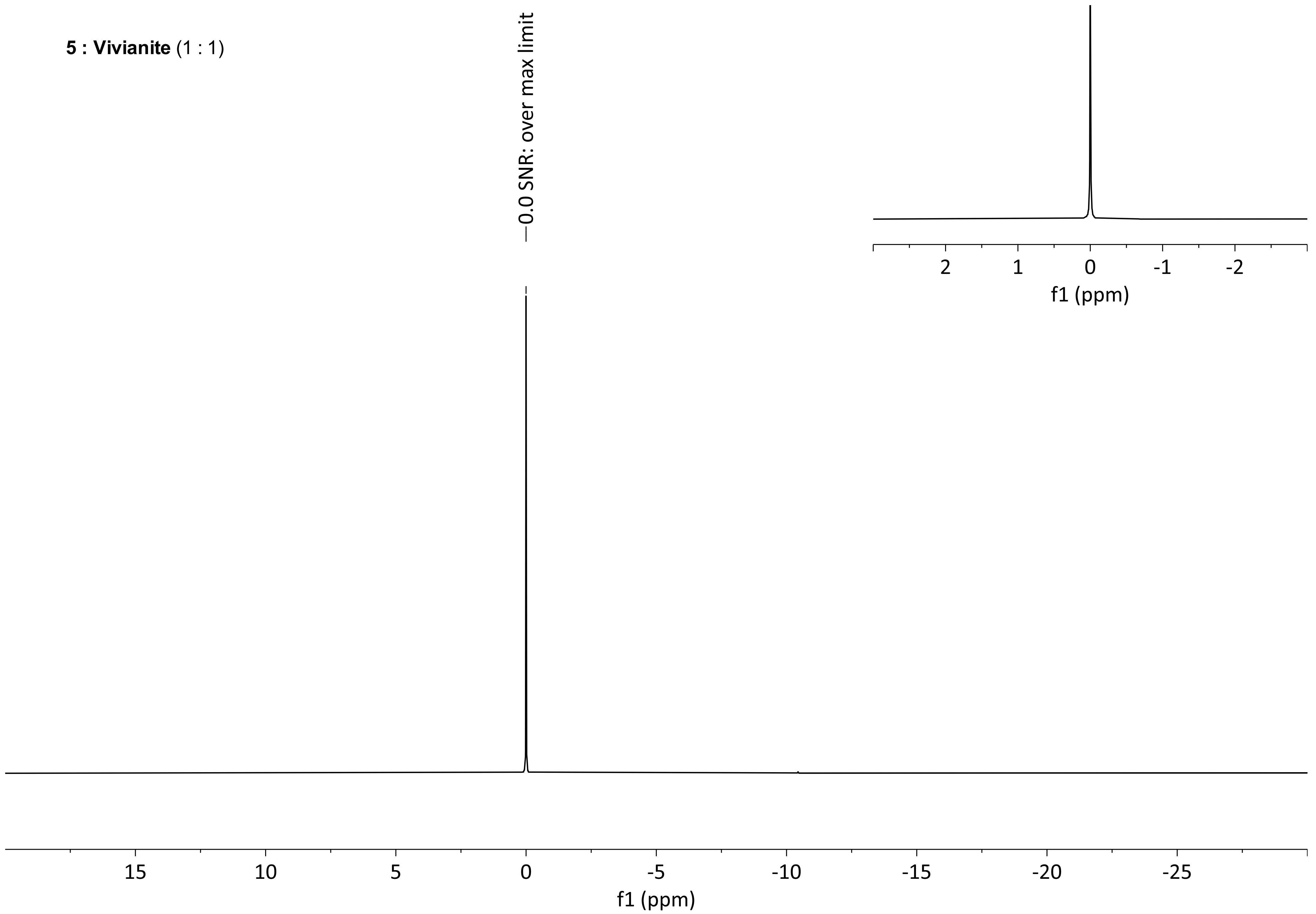

5 : Vivianite (1 : 1)
—0.0 SNR: over max limit
2
1
0
-1
-2
f1 (ppm)
15
10
5
0
-5
-10
-15
-20
-25
f1 (ppm)

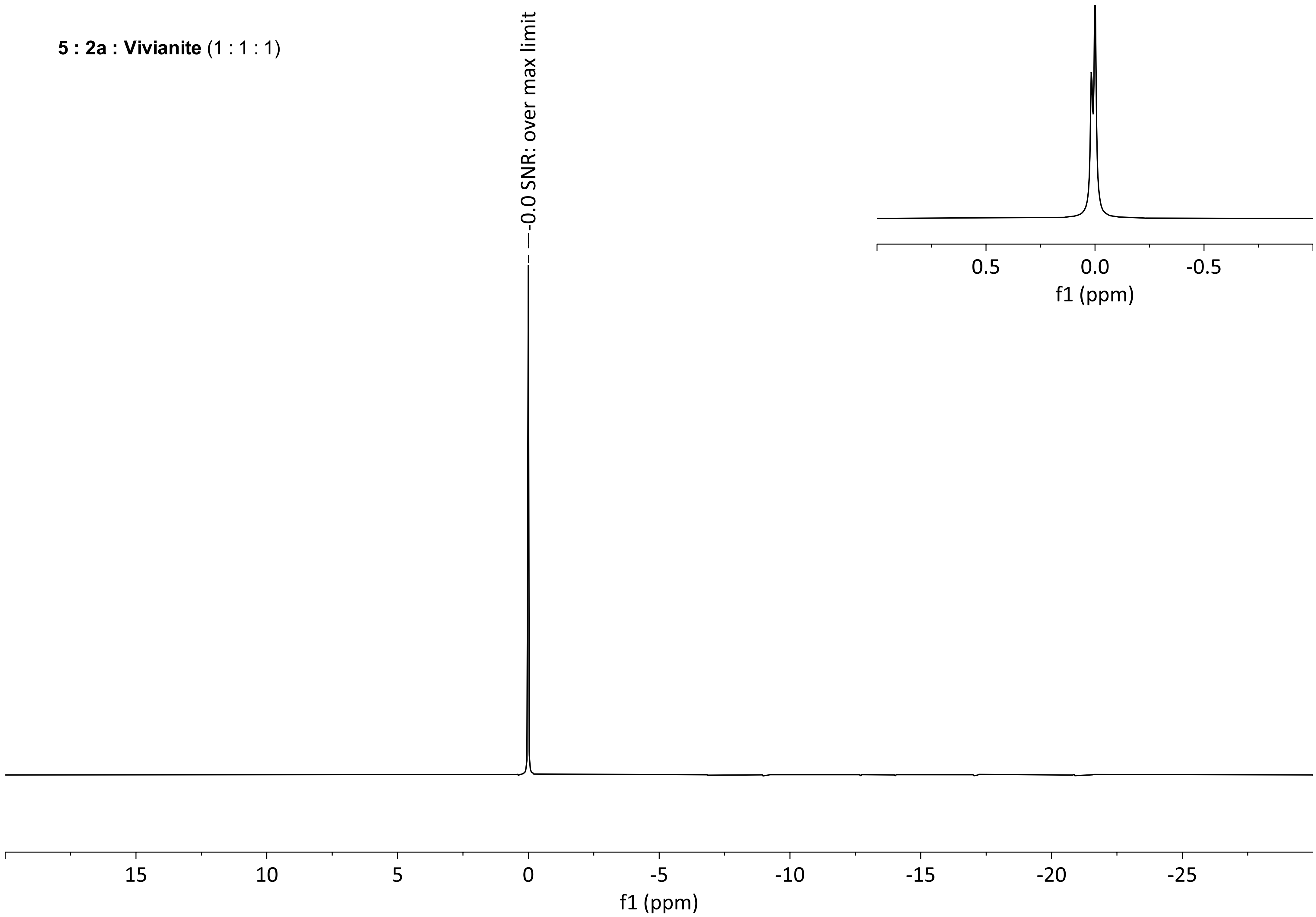

5 : 2a : Vivianite (1 : 1 : 1)
-0.0 SNR: over max limit
15
10
5
0
-5
-10
-15
-20
-25
f1 (ppm)
0.5
0.0
-0.5
f1 (ppm)

5 : 1 : Vivianite (1 : 1 : 1)

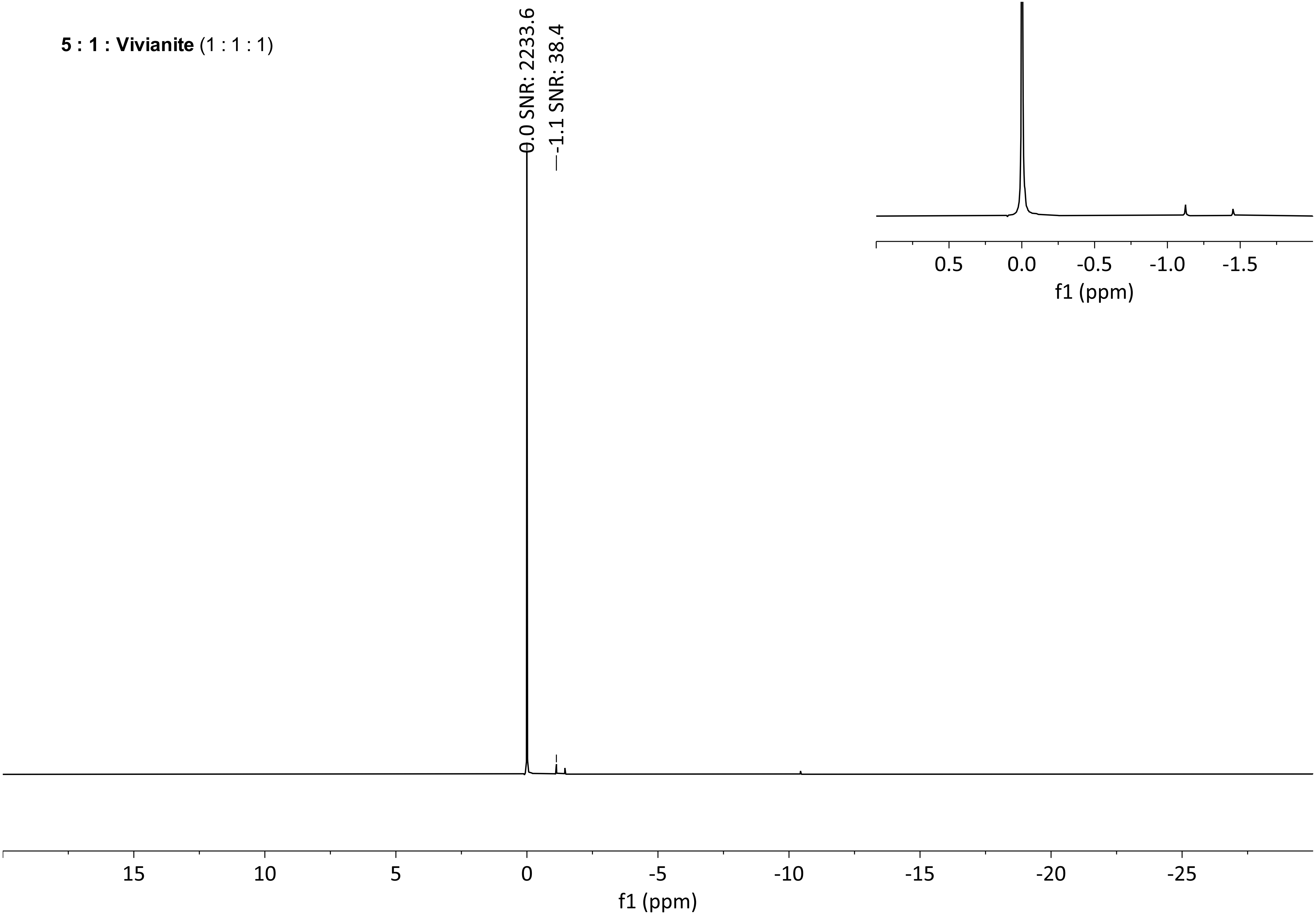

5 : Canaphite an. (1 : 1)

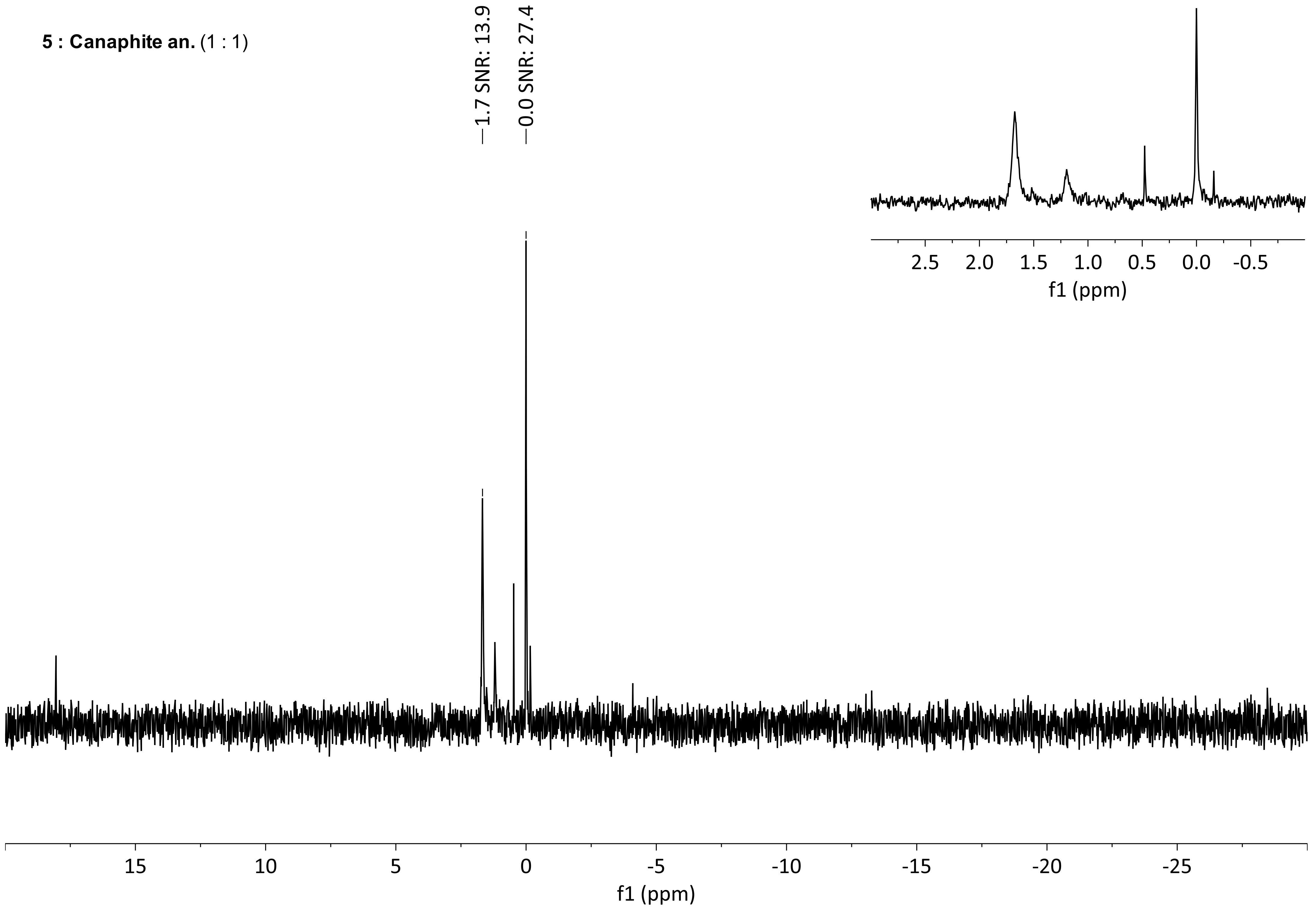

5 : 2a : Canaphite an. (1 : 1 : 1)

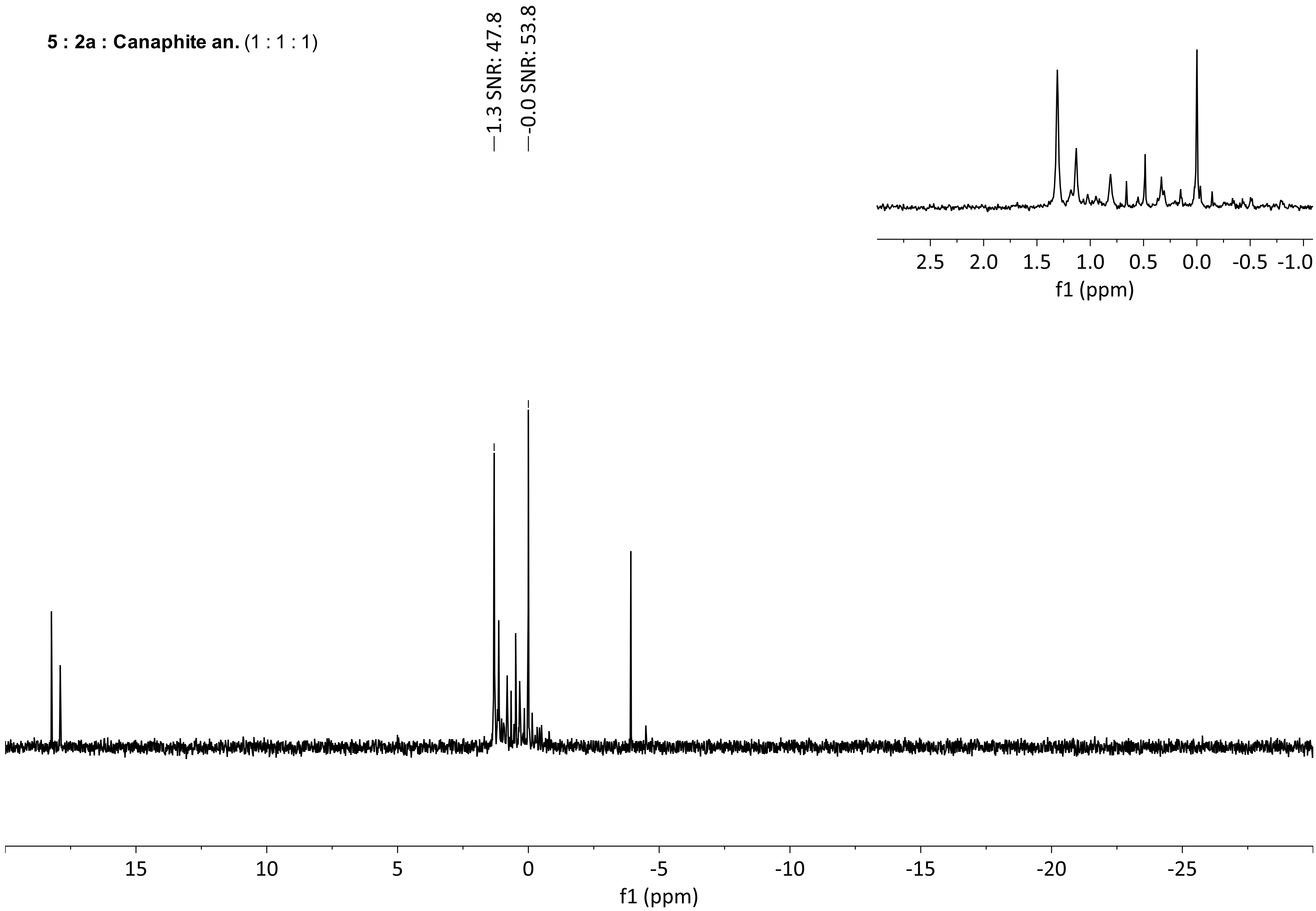

5 : 1 : Canaphite an. (1 : 1 : 1)

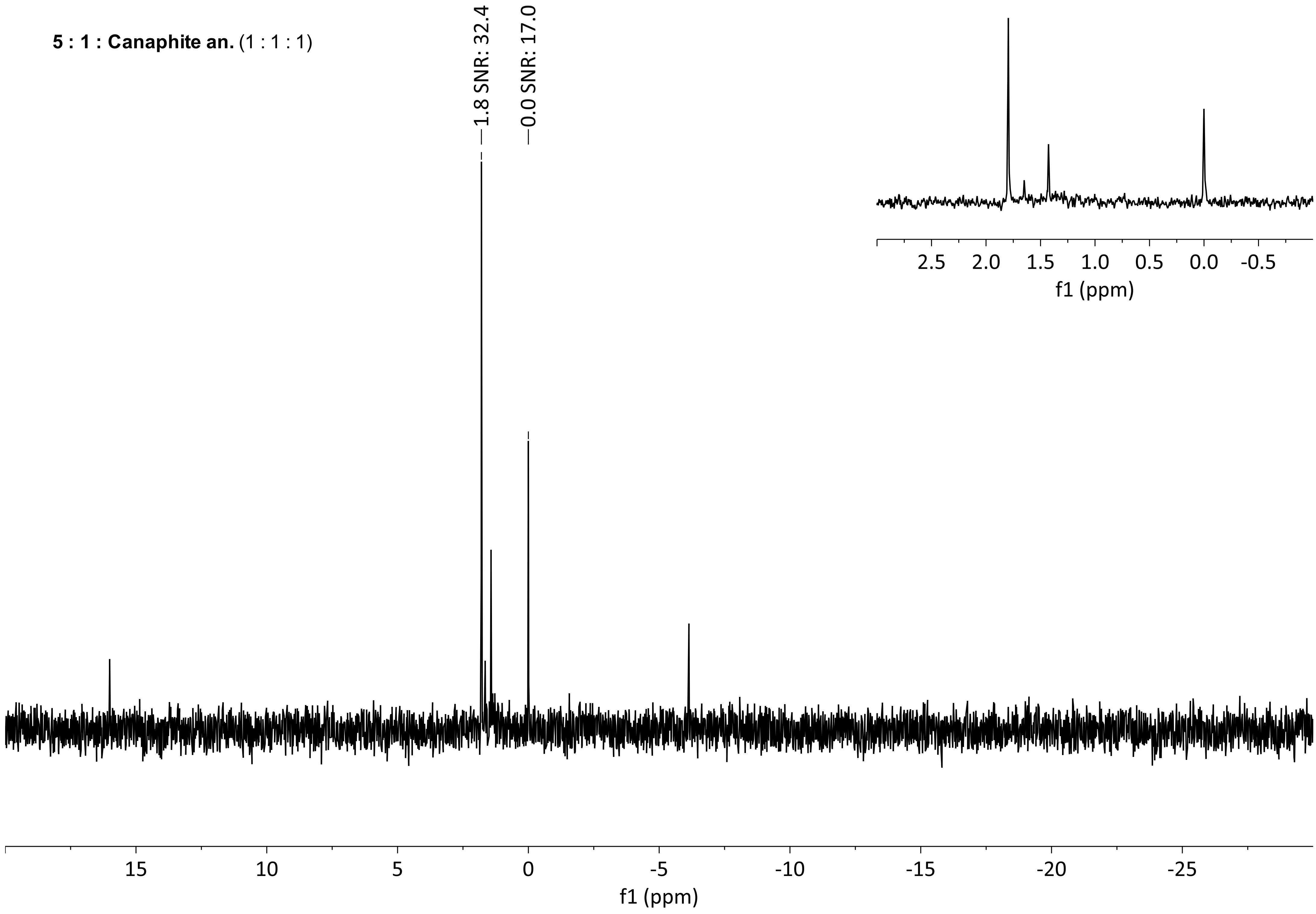

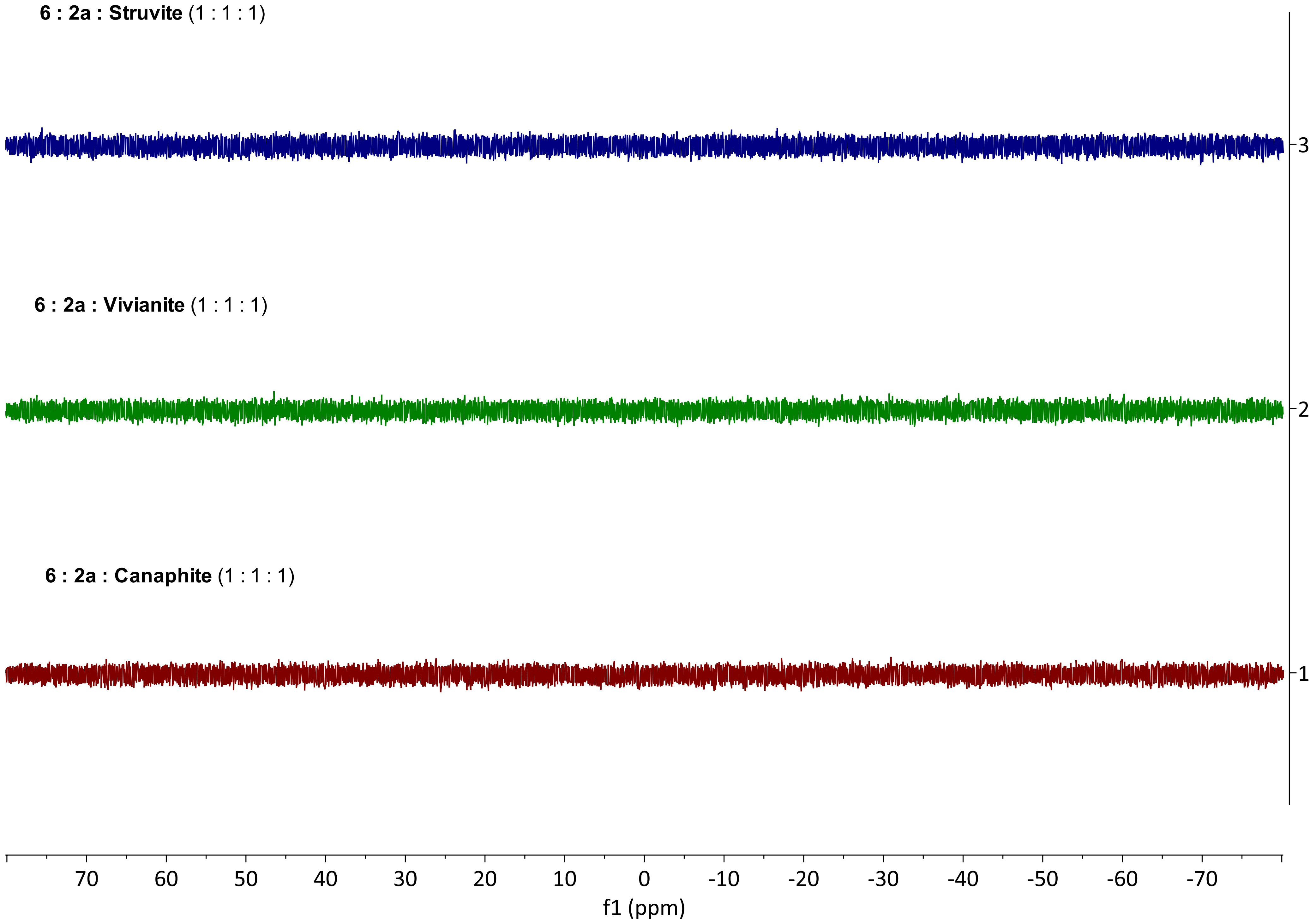

6 : 2a : Struvite (1 : 1 : 1)
6 : 2a : Vivianite (1 : 1 : 1)
6 : 2a : Canaphite (1 : 1 : 1)
3
2
1
70 60 50 40 30 20 10 0 -10 -20 -30 -40 -50 -60 -70
f1 (ppm)
